# Photoredox‐Catalyzed Hydroalkylation of C(sp^3^)–H Acids

**DOI:** 10.1002/chem.202501148

**Published:** 2025-04-21

**Authors:** Avery O. Morris, Tegan E. O'Brien, Louis Barriault

**Affiliations:** ^1^ Center for Catalysis Research and Innovation Department of Chemistry and Biomolecular Sciences University of Ottawa 10 Marie‐Curie Ottawa K1N 6N5 Canada

**Keywords:** cross‐coupling, hydroalkylation, oxidative cyclization, photoredox catalysis, spirocycles

## Abstract

We present a detailed study on a photoredox catalysis platform that directly engages 1,3‐dicarbonyl C(sp^3^)–H acids toward radical reactions. This platform enables redox‐neutral hydroalkylation and cross‐coupling, as well as oxidative transformations that demonstrably improve on the prior state of the art. Herein, we present interrogations of the underlying catalytic cycle and mechanism for this platform through kinetic, thermodynamic, and computational studies. The present investigations also demonstrate the key role of lithium trifluoroacetate under complementary Ce‐containing and Ce‐free photoredox conditions to enable ligand‐to‐metal charge transfer (LMCT) or multi‐site proton‐coupled electron transfer (MS‐PCET) activations, respectively.

## Graphical abstract

Two versatile photoredox catalysis platforms were developed to engage C(sp^3^)–H acids in hydroalkylations, cross‐couplings, and oxidative cyclizations. Extensive synthetic and mechanistic investigations demonstrate a complementarity between the activation mechanisms of these two systems.

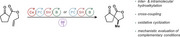

## 1 Introduction

**Scheme 1:**
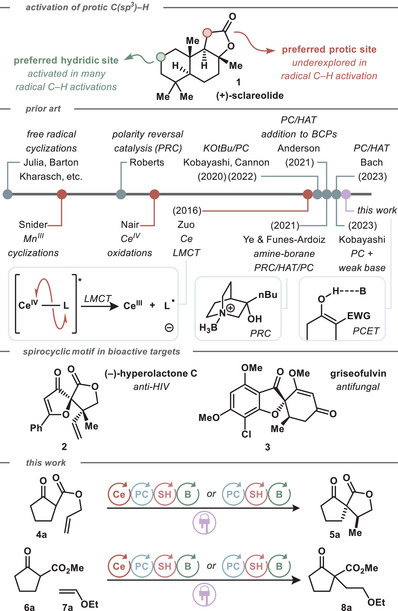
Methods for direct conversion of C(sp3)–H carbon acids to radical intermediates.

The activation of C–H bonds remains a key challenge in modern synthesis, and new methodologies that address this challenge are essential to accelerate the exploration of chemical space and the development of new chemical tools, biological probes, and pharmaceuticals.^[^ ^1^, ^2^ ^]^ C(sp^3^)–H acids are a ubiquitous functionality, but methods for their catalytic functionalization toward radical chemistry have lagged behind those aimed at hydridic C(sp^3^)–H or protic X–H bonds (Scheme 1).^[^ ^3^ ^]^ This disparity can be attributed to the relative scarcity of systems with the requisite polarity to promote efficient activation.^[^ ^4^, ^5^, ^6^ ^]^ Consequently, protic C–H bonds have been underutilized in radical chemistry despite the potential for rapid elaboration of these feedstock chemicals into complex architectures.

In contrast to the use of protic 1,3‐dicarbonyls in anionic chemistry, their use in radical chemistry is less prolific. Over 50 years ago, Julia demonstrated the feasibility of hydrogen atom transfer (HAT) radical cyclizations using C(sp^3^)–H acids.^[^ ^7^ ^]^ Despite this demonstration, a persistent sentiment in the field is that “hydrogen atom transfer cyclizations are of only limited value for organic synthesis”.^[^ ^8^ ^]^ Many early methods relied on short, inefficient radical chains with limited substrate generality, leading to their reputation as capricious systems of little synthetic value.^[^ ^9^ ^]^

Two stoichiometric strategies to activate 1,3‐dicarbonyls for radical cyclization surpassed these earlier HAT methods. The first strategy used stoichiometric single‐electron metal oxidants and its success dispelled concerns about the utility of carbon acid C–H functionalization in synthesis or radical chemistry.^[^ ^10^, ^11^ ^]^ Despite their success, these oxidative methods were limited in their product diversity and only amenable to substrates that could tolerate harsh, oxidizing conditions.

The second widespread solution was to perform reductive cyclization.^[^ ^12^, ^13^, ^14^, ^15^ ^]^ This meant that the C–H acid functionality had to be derivatized prior to radical cyclization.

The ideal functionalization of 1,3‐dicarbonyl acids would use the native C–H functionality and proceed under mild conditions without generating stoichiometric waste, ideals most readily achieved through catalysis.^[^ ^16^ ^]^ Catalytic activation would also provide a more versatile platform to allow radical intermediates to be intercepted through a variety of established mechanisms, thereby greatly expanding the scope of available transformations.^[^ ^17^, ^18^ ^]^ Despite these potential benefits, the catalytic generation of radicals from protic 1,3‐dicarbonyls remains an underexplored topic.

We reasoned that an efficient photoredox platform capable of catalyzing the Kharasch‐type hydroalkylation of olefin acceptors with 1,3‐dicarbonyl acids might provide a platform amenable to a variety of transformations that benefits from the ideals of catalysis.^[^ ^9^, ^19^, ^20^ ^]^ Recent reports on photocatalytic activation of 1,3‐dicarbonyls have employed two distinct strategies. The first, a formal proton‐transfer electron‐transfer (PTET) strategy, employed a strong Brønsted base to generate enolates that are predisposed to oxidation via photoinduced electron transfer (PET).^[^ ^21^, ^22^, ^23^ ^]^ While effective, this strategy was limited to substrates that can undergo an electron‐transfer proton‐transfer (ETPT) sequence following cyclization. To ameliorate these issues, Kobayashi later followed this work up with the application of a weaker Li‐thiophenoxide base that can also double as a hydrogen atom donor.^[^ ^24^ ^]^ The second strategy built on Roberts’ earlier polarity reversal catalysis (PRC) concept and relied on HAT activation of the 1,3‐dicarbonyl substrates.^[^ ^25^ ^]^ Funez‐Ardoiz and Ye reported an ingenious photoredox‐catalyzed hydroalkylation method employing polarity‐matched boryl radicals.^[^ ^26^ ^]^ While an excellent application of polarity matching, the reliance on HAT activation means the performance of this strategy with substrates that favor an enol tautomer is unknown.

We envisioned an alternative strategy for photocatalytic functionalization of C(sp^3^)–H acids based on ligand‐to‐metal charge‐transfer (LMCT),^[^ ^27^, ^28^, ^29^ ^]^ surmising that this would confer several advantages compared to HAT or PTET. The first anticipated advantage was that LMCT would readily port to diversifying intercept technologies like nickel metallaphotoredox,^[^ ^30^ ^]^ or oxidative terminations.^[^ ^31^, ^32^ ^]^ Additionally, we reasoned that this functionalization mode has the potential to greatly expand the scope of amenable substrates by capitalizing on the dynamics of keto‐enol tautomerism. The expected interaction with the enol tautomer allowed us to envision substrate functionalization for the entire catalog of 1,3‐dicarbonyl substrates in addition to substrates that exclusively adopt the enol form in solution. With the freedom to employ substrates irrespective of the tautomeric equilibrium, we endeavored to identify and demonstrate the utility and versatility of the LMCT functionalization strategy.

This blueprint led us to explore Ce photoredox catalysis to effect the requisite LMCT, focusing primarily on the creation of α‐keto‐spirocycles for their prevalence in bioactive compounds like hyperolactone C (**2**) and griseofulvin (**3**) (Scheme 1).^[^ ^33^, ^34^ ^]^ The wealth of literature detailing the use of Ce(IV) salts as single electron oxidants for 1,3‐dicarbonyls suggested that a system comprised of a ground‐state Ce(IV/III) cycle mediated by photocatalytic turnover might also prove viable.^[^ ^35^ ^]^ These degenerate catalytic proposals served to convince us that such a system could quickly be identified. Herein, we report two versatile photoredox catalysis platforms that exploit the keto‐enol tautomerism of 1,3‐dicarbonyls in an array of transformations. We provide evidence for LMCT and proton‐coupled electron transfer (PCET) functionalization and elucidate the reaction mechanisms using computational, kinetic, and thermodynamic studies.

## 2 Results and Discussion

### 2.1 Optimization and Scope

Toward this goal, we began with our previously identified conditions for the redox‐neutral β‐scission of lactols and hydrodecarboxylation reactions.^[^ ^36^ ^]^ Gratifyingly, violet LED irradiation of a suspension of β‐ketoester **4a**, cerium(III) chloride, bis(2,4,6‐triisopropylphenyl)disulfide ((TRIPS)_2_), tetrabutylammonium chloride (TBACl), sodium benzoate and 9,10‐diphenylanthracene (9,10‐DPA) for 16 hours under argon atmosphere delivered the desired [4.4]‐spirocyclic ketolactone **5a** in 83% yield and 3.8:1 diastereomeric ratio (Table 1, entry 1).

**Table 1:** Optimization of the redox‐neutral hydroalkylation.

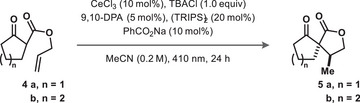				
Entry	n	Deviation	Yield [%]^[a]^	d.r.
1	1	none	83	3.8:1
2	1	no light, heated to 55 °C	n.d.	–
3	1	no (TRIPS)_2_	20	3.0:1
4	1	no TBACl	33	2.0:1
5	1	no CeCl_3_	0	–
6	1	no Ce, no TBACl	57	3.0:1
7	1	no 9,10‐DPA	8	2.0:1
8	1	no PhCO_2_Na	86	3.0:1
9	1	Ce(OTf)_3_ & CF_3_CO_2_Na in place of CeCl_3_ & PhCO_2_Na	90^[^ ^b^ ^]^	4.0:1
10	1	Ce(OTf)_3_ & CF_3_CO_2_Li in place of CeCl_3_ & PhCO_2_Na	85	3.1:1
11	1	no Ce, no TBACl, CF_3_CO_2_Li instead of PhCO_2_Na	90 (80^[^ ^b^ ^]^)	3.4:1
12	2	no Ce, no TBACl, CF_3_CO_2_Li instead of PhCO_2_Na	78	2.5:1
13	2	no Ce, no TBACl, CF_3_CO_2_Na instead of PhCO_2_Na	13	2.0:1

^[a]^ ^1^H NMR yields;

^[b]^ Isolated yield.

Control reactions showed that the reaction was photoinduced, excluding thermal Conia‐ene reactivity as the origin of product formation (Table 1, entry 2). Removal of (TRIPS)_2_ resulted in severely reduced conversion to **5a** (Table 1, entry 3). Conversion to **5a** in the absence of a disulfide co‐catalyst may arise from a HAT‐propagated background radical chain reaction. The omission of TBACl significantly reduced the yield, aligning with previous findings that high halide concentrations are imperative for efficient Ce reactivity (Table 1, entry 4).^[^ ^37^, ^38^ ^]^ Exclusion of CeCl_3_ resulted in no detectable conversion of **4a** to **5a** (Table 1, entry 5). Curiously, the simultaneous exclusion of CeCl_3_ and TBACl saw a revival in catalytic turnover toward **5a** (Table 1, entry 6). We reasoned that in the absence of both Ce(III) and excess chloride, a background PCET oxidation of **4a** by photoexcited 9,10‐DPA* is likely operative (E_1/2_(ketoester/ketoester^•+^) = +1.89 V versus SCE; E(DPA*/DPA^•–^) = +1.28 V versus SCE in MeCN).^[^ ^39^, ^40^ ^]^ The exclusion of 9,10‐DPA led to a diminished yield of 8% (Table 1, entry 7), indicating that conversion by LMCT oxidation by cerium is possible but inefficient. Removal of PhCO_2_Na led to a slight improvement in yield (Table 1, entry 8). This unexpected result prompted us to further investigate the identity of the carboxylate additive. After extensive screening (see SI for details), we identified trifluoroacetate salts as the superior additive to accomplish hydroalkylation. Screening Ce(III) sources identified Ce(OTf)_3_ as the most effective pre‐catalyst, likely a result of its superior solubility in MeCN. When used in conjunction with CF_3_CO_2_Na additive, Ce(OTf)_3_ delivered **5a** in 90% isolated yield (Table 1, entry 9). A comparison of CF_3_CO_2_Na to CF_3_CO_2_Li showed no substantial difference between the two cations (Table 1, Entries 8 & 9).

Building upon the Ce/TBACl‐free control (Table 1, entry 6), we explored the viability of cerium‐free conditions. A significant cation effect was observed, wherein CF_3_CO_2_Li, 9,10‐DPA, and (TRIPS)_2_ yielded **5b** in 78% yield (Table 1, entry 12), while the use of CF_3_CO_2_Na resulted in 13% yield (Table 1, entry 13). With two sets of optimized hydroalkylation conditions in hand, we sought to demonstrate the utility of this redox‐neutral photocatalytic hydroalkylation reaction.

**Figure 1:**
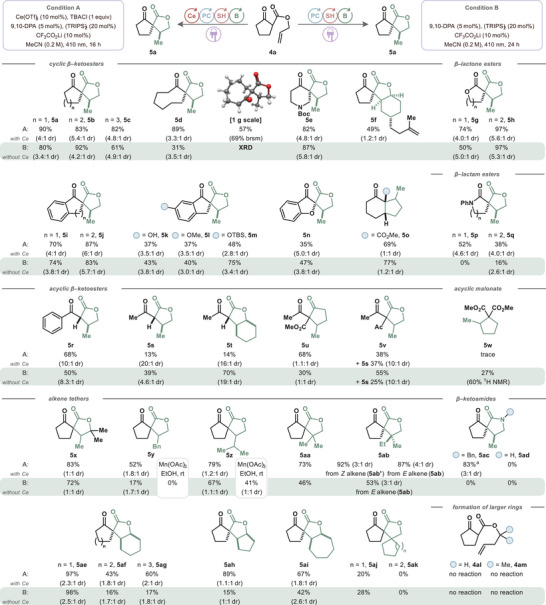
Scope of intramolecular hydroalkylation.

Application of cerium‐containing conditions (A) and cerium‐free conditions (B) to intramolecular hydroalkylation of 1,3‐dicarbonyls with tethered alkenes gave a variety of spirocyclic products (Figure 1). Cyclic β‐ketoesters of increasing ring size from 5‐ to 8‐membered gave the corresponding spirocycles **5a**–**d** in excellent yields and with good stereoselectivity. The reaction of **4d** on a gram‐scale using condition A delivered the product in 57% yield and 3.4:1 dr after 72 h (69% brsm). Spirocycle **5d** was crystalline and SCXRD analysis confirmed the *cis* relationship between methyl and ketone groups in the major diastereomer.

Boc‐protected amine **4e** was well tolerated by both conditions, yielding ketolactone **5e** in 82% (A) and 87% (B) yield. Substrate **4f** gave only singly‐cyclized tricycle **5f** under condition A with moderate yield and low diastereoselectivity. β‐Lactone esters also proved to be competent substrates; 5‐membered spiro[4.4] bis‐lactone **5** **g** was formed in good yield and the corresponding spiro[5.4] bis‐lactone **5** **h** was formed in excellent yield under both conditions.

Benzo‐fused indanone and tetralone derivatives also gave spirocyclic products **5i–m** in fair to excellent yield (35–87%). Unprotected phenol **4k** was tolerated by both conditions to give **5k**, albeit in lower yields. Methyl‐ether‐protected phenol spirocycle **5l** was produced in comparable yields, suggesting that the diminished yields observed for this substrate series are not due to the free phenol O–H. Surprisingly, silyl‐protecting groups were tolerated, with TBDMS‐protected phenol **5** **m** formed in 48% using condition A and an excellent yield of 75% using condition B. We suspect that the high chloride concentration under condition A may render the phenolic silyl ether labile. Benzofuranone **5n** was formed in lower yield, likely due to the instability of precursor **4n** to acid and oxygen that necessitated its immediate preparation and use. Fused ring systems were also accessible through both hydroalkylation conditions, with ketolactone **5o** furnished in high yield. Lactam esters were also competent substrates, with 5‐ and 6‐membered spiro lactams **5p** and **5q** formed in 52% and 38% yield, respectively, under condition A. Under cerium‐free conditions (B), the 5‐membered lactam failed to undergo conversion and the 6‐membered **5q** was obtained in only 16% yield.

Acyclic β‐ketoesters performed the hydroalkylation similarly well to their cyclic counterparts. Acetophenone‐type substrate **4r** gave lactone **5r** in good yield under both conditions, with *trans* stereochemistry due to epimerization. Cyclization of the simplest allyl β‐ketoester gave **5s** in low isolated yields due to volatility, and cyclization onto a cyclic alkene gave fused bicyclic lactone **5t** in comparable yields. Acyclic tricarbonyl substrates were also competent, though their cyclized products **5u** and **5v** were plagued by retro‐Claisen reactions, leading to diminished yields. Malonate‐derived product **5w** was formed under cerium‐free conditions, though cerium‐containing conditions gave **5w** in only trace amounts.

Varying the allyl ester substitution furnished various spiro[4.4] ketolactones through the favored 5‐*exo‐trig* cyclization. Dimethyl‐substituted lactone **5x** was produced in high yields under both conditions. Cyclization to form a secondary, benzylic radical was possible under both conditions, forming lactone **5y** in moderate yield after HAT. Similarly, cyclization to form a tertiary alkyl radical intermediate proceeded readily under both conditions to give isopropyl‐substituted lactone **5z** in high yields. In comparison, redox‐neutral Mn(OAc)_3_ cyclization conditions failed to deliver **5y**, and delivered **5z** in a diminished yield. We anticipated that these substrates would be susceptible to degradation under conditions employing stoichiometric oxidants. Geminally disubstituted products **5aa** and **5ab** were formed in high yield, demonstrating the strength of this radical cyclization in accessing sterically congested products.

Benzyl‐protected ketoamide **4ac** underwent the hydroalkylation under condition A in 83% yield with a 3:1 dr but did not react under the cerium‐free conditions (B). In contrast, secondary amide **4ad** failed to react under either condition. Esters derived from secondary, cyclic allyl alcohols performed well in the hydroalkylation. Varying ring sizes led to the formation of spirocycles **5ae**–**5ai** in low to excellent yields.

Ester **4aj** gave diaspora ketolactone **5aj** in low yields under both conditions, likely due to increased steric strain during the cyclization. Similarly, ester **4ak** failed to cyclize under either condition. Attempts at 6‐*exo‐trig* or 7‐*endo‐trig* cyclizations with substrate **4al** were unproductive under both conditions. Attempts to induce a Thorpe‐Ingold effect using substrate **4am** failed to yield product.

**Figure 2:**
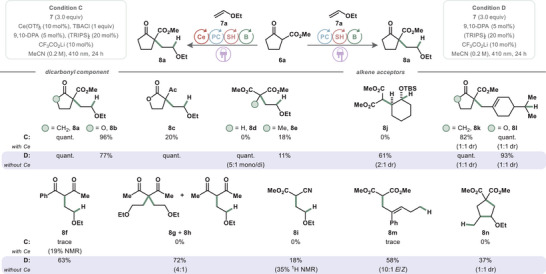
Scope of intermolecular hydroalkylation.

Intermolecular addition reactions were also achieved by this method (Figure 2). β‐ketoester **6a** and β‐lactone ester **6b** were added readily to ethyl vinyl ether (**7a**) at the terminal position to give linear adducts **4a** and **4b** in high to quantitative yields under both Ce‐containing conditions (C) and Ce‐free conditions (D). A range of dicarbonyl species was successfully added to ethyl vinyl ether (**7a**) in this unusual combination, where a nucleophilic carbon is coupled with an electron‐rich alkene in a formal cross‐nucleophile coupling. β‐ketolactone **6c** gave adduct **4c** in only 45% yield by ^1^H NMR under condition C, but cerium‐free conditions achieved quantitative yield. Dimethyl malonate (**6d**) showed a complete lack of reactivity under condition C but performed the addition to ethyl vinyl ether (**7a**) in quantitative yield under condition D. A significant difference in reactivity was observed between dimethyl malonate (**6d**) and dimethyl methylmalonate (**6e**), which forms adduct **8e** in 18% yield under condition C and 11% yield under condition D. Malonate‐derived methine substrates appear to struggle in this reaction, though it is notable that reactivity is observed under cerium‐containing conditions where no reactivity is observed for the corresponding methylene. Diketones **6f** and **6g** and methyl cyanoacetate (**6i**) exhibited limited reactivity under condition C. Under cerium‐free conditions (D), these substrates showed improved reactivity. Acetylacetone (**6g**) produced the double addition product **8g** as the major product, though decreasing the ethyl vinyl ether (**7a**) loading to 1.2 equivalents enabled isolation of the mono‐addition adduct **8h** in 42% yield (see SI). Methyl cyanoacetate (**6i**) gave adduct **8i** in 35% yield by ^1^H NMR, with reduced isolated yield due to volatility.

Additionally, intermolecular hydroalkylation was attempted with a range of alkene acceptors. Dimethyl malonate (**6d**) was able to add to silyl enol ether **7b** under condition D to give trimethylsilyl ether adduct **8j** in 61% yield as the *trans* isomer predominantly. In addition, unactivated alkenes were not productive under either condition. A notable exception was the addition of ketoester **6a** and lactone ester **6b** to β‐pinene (**7c**) to form bicyclic products **8k** and **8l** in high to quantitative yields under both conditions. We hypothesized that the formation of adduct **8k** benefits from strain‐releasing radical fragmentation upon addition. This suggests that, under the reaction conditions, addition to other unactivated alkenes may be possible but reversible, leading to unproductive regeneration of the starting material. The addition of dimethyl malonate (**6d**) to α‐cyclopropylstyrene (**7d**) yielded a fragmented product **8** **m** in 58% yield under condition D as a 10:1 *E*/*Z* mixture. A formal [3+2] reaction was achieved via an addition‐cyclization sequence between dimethyl allylmalonate (**6n**) and ethyl vinyl ether (**7a**), yielding cyclopentane **8n** in 37% yield under cerium‐free conditions.

**Scheme 2:**
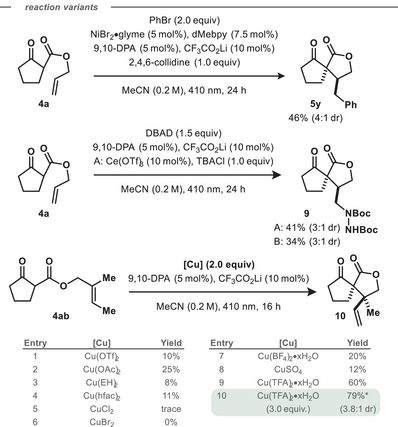
Variations of the intramolecular hydroalkylation reaction.

Beyond the redox‐neutral hydroalkylation, orthogonal transformations of the cyclized C‐centred radical intermediate were successfully demonstrated (Scheme 2). Cross‐coupling was achieved with bromobenzene, catalyzed by 9,10‐DPA, CF_3_CO_2_Li, NiBr_2_•glyme, 4,4′‐dimethyl‐2,2′‐bipyridine, and 2,4,6‐collidine to afford spiroketolactone **5y** in 46% yield, without further optimization. Amination was achieved by addition to di‐*tert*‐butyl azodicarboxylate (DBAD) in the absence of HAT catalyst, yielding amination product **9** in 41% yield under cerium‐containing conditions and 34% yield under cerium‐free conditions, without optimization. We also explored an oxidative pathway, where the cyclized radical intermediate was intercepted by a Cu(II) oxidant to form olefin **10**. Screening of Cu(II) sources identified copper(II) trifluoroacetate hydrate as a highly effective oxidant, producing spirolactone **10** in 79% isolated yield as a 3.8:1 diastereomeric mixture.

### 2.2 Mechanistic Investigations

**Figure 3:**
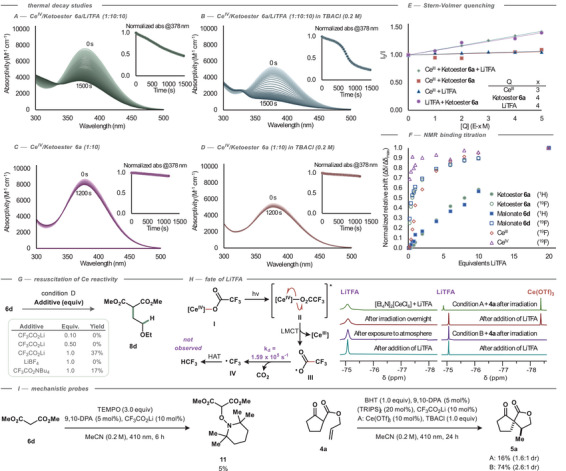
Mechanistic investigations. Thermal decay profiles of Ce^IV^ absorptivity as mixtures in MeCN at room temperature in the dark, with inset showing normalized absorbance at 378 nm over time. **A**) [Et_4_N]_2_[Ce^IV^Cl_6_]/ketoester **6a**/LiTFA (1:10:10, 0.33 mM); **B**) [Et_4_N]_2_[Ce^IV^Cl_6_]/ketoester **6a**/LiTFA (1:10:10, 0.33 mM, 0.2 M in TBACl); **C**) [Et_4_N]_2_[Ce^IV^Cl_6_]/ketoester **6a** (1:10, 0.33 mM); **D**) [Et_4_N]_2_[Ce^IV^Cl_6_]/ketoester **6a** (1:10, 0.33 mM, 0.2 M in TBACl). **E**) Stern‐Volmer fluorescence quenching of 9,10‐DPA* in MeCN (λ_excitation_ = 400 nm, λ_emission_ = 427 nm) with mixtures of reaction components as quenchers. **F**) NMR binding studies of reaction components, ^1^H and ^19^F chemical shift in MeCN when ketoester **6a**, or malonate **6d**, or [Et_4_N]_3_[Ce^III^Cl_6_] is titrated with LiTFA, relative to initial shift and normalized to maximum difference in shift. **G**) Resuscitation of malonate **6d** reactivity under Ce‐containing conditions with increased loading of LiTFA and related additives, ^1^H NMR yields. **H**) Potential decomposition pathways of LiTFA under the reaction conditions, with NMR spectra inset showing ^19^F signals after reaction tests. **I**) Mechanistic probes including TEMPO or BHT.

Next, we sought to investigate the differences between Ce‐containing and Ce‐free conditions. Given the established precedent for Ce(IV) single‐electron oxidation of β‐ketoesters in the ground state,^[^ ^35^ ^]^ we first aimed to understand the action of putative complex [Et_4_N]_2_[Ce^IV^Cl_6_] in the presence of ketoester **6a**. To this end, we examined the thermal decay of Ce(IV) by UV–vis spectroscopy under a variety of conditions (Figure 3A–D). The single‐electron reduction of Ce(IV) to Ce(III) in solution is easily observed as a disappearance of the strong absorbance band at 378 nm and the appearance of a weaker band at 328 nm.^[^ ^41^, ^42^ ^]^ Thermally promoted decay in the dark at room temperature occurred at an initial rate of 3 × 10^−7^ M s^−1^ in a mixture of ketoester **6a**/[Et_4_N]_2_[Ce^IV^Cl_6_] (10:1) (Figure 3C), which accelerated to 1 × 10^−6^ M s^−1^ with the inclusion of LiTFA (ketoester **6a**/LiTFA/[Et_4_N]_2_[Ce^IV^Cl_6_], 10:10:1) (Figure 3A). Loss of Ce(IV) absorbance was negligible under thermal conditions in solutions of [Et_4_N]_2_[Ce^IV^Cl_6_], mixtures of LiTFA/[Et_4_N]_2_[Ce^IV^Cl_6_] (10:1), or in any equimolar combination with the ketoester **6a** and LiTFA at room temperature. The pronounced effect of LiTFA on the thermal reduction of Ce(IV) in the presence of ketoester **6a** suggests an increased propensity for substrate–Ce(IV) complexation and oxidation, or potentially a stabilization of post‐oxidation products.

The addition of TBACl in the ketoester **6a**/LiTFA/[Et_4_N]_2_[Ce^IV^Cl_6_] (10:10:1) solution led to a sigmoidal depletion of absorbance at 378 nm over time. This sigmoidal behavior suggests a lag phase in which slow complexation between the components limits reduction, with an increase in rate as equilibrium approaches (Figure 3B). Near the inflection point, the rate of Ce(IV) reduction occurs more rapidly than that of the corresponding mixture without supporting chloride (Figure 3A). The greater rate of thermal decay in the presence of excess chloride could be attributed to the suppression of back electron transfer (BET) after the redox event, either by stabilizing the resulting oxidized species or by occupying the newly formed coordination site.

Having demonstrated that Ce(IV) is a competent activator of ketoester **6a**, we turned to Stern‐Volmer studies to establish its accessibility under the reaction conditions. Initial forays proved challenging due to 9,10‐DPA's short excited‐state lifetime (9 ns in MeCN)^[^ ^43^ ^]^ and insignificant quenching rates when employing individual reaction components, up to their solubility limits (see SI for details). This analysis was further complicated by the complex equilibria involved in the generation of the active quencher entities.^[^ ^44^, ^45^ ^]^ Titration of two‐component quencher mixtures such as [Et_4_N]_3_[Ce^III^Cl_6_]/ketoester **6a** (1:10) or [Et_4_N]_3_[Ce^III^Cl_6_]/LiTFA (1:10) showed no discernable quenching. In contrast, titrating a 1:1 mixture of LiTFA and ketoester **6a** caused a notable decrease in emission intensity, as did titration of a 10:10:1 mixture of LiTFA, ketoester, and Ce(III) (Figure 3E). The quenching efficiency was comparable in both cerium‐containing and cerium‐free systems.

To gain insight into the identity of the active quencher species, we studied their binding equilibria by NMR. Titration of ketoester **6a** and malonate **6d** with LiTFA revealed a nearly identical normalized response, suggesting that both substrates bind with LiTFA with a similar thermodynamic drive (Figure 3F). This finding aligns with the observation that both substrates participate in hydroalkylation in the absence of Ce. A similar response was observed in the addition of LiTFA to [Et_4_N]_3_[Ce^III^Cl_6_], though this effect was attenuated in the presence of 0.2 M tetraethylammonium chloride (see SI). Titration of [Et_4_N]_2_[Ce^IV^Cl_6_] with LiTFA produced a more immediate response, suggesting stronger binding.

Titration of Ce(III) analyte solutions with ketoester **6a** or malonate **6d** showed no discernable binding isotherm, while the corresponding Ce(IV) studies were thwarted by thermal decay (see SI). These results, taken together with the thermal Ce(IV) decay studies, suggest that substrate–Ce complexation is probable, but could not be detected by NMR spectroscopy.

In light of these mechanistic studies, substrate activation in the absence of Ce(III) could occur by a proton transfer/electron transfer (PT/ET) mechanism or by multi‐site proton‐coupled electron transfer (MS‐PCET). Given the pK_a_ difference between ketoesters and LiTFA (10.5 vs ‐0.25 in water),^[^ ^46^ ^]^ the standing concentration of lithium enolate would be infinitesimal and would imply a fluorescence quenching rate well beyond the diffusion limit, making the PT/ET sequence unlikely. This makes the MS‐PCET activation of a ketoester–LiTFA complex the more kinetically plausible quenching event. Despite the similarity in excited‐state quenching kinetics between the cerium‐free and cerium‐containing systems, our synthetic studies showed numerous instances in which the cerium‐containing conditions outperformed reactions run without it. This discrepancy suggests a subtle substrate dependence for Ce and LiTFA binding.

Since the cerium‐free conditions are a subset of the cerium‐containing conditions, it might be expected that the cerium‐free activation would operate in the background and would therefore be general to all substrates. However, the reaction scope shows that dimethyl malonate (**6d**), acetylacetone (**6g**), and methyl cyanoacetate (**6i**) are added to ethyl vinyl ether (**7a**) under cerium‐free conditions but not under cerium‐containing conditions (Figure 2). In the formation of dimethyl malonate adduct **8d** under cerium‐containing conditions (C), increasing the LiTFA loading from 10 to 50 mol% gave no trace of product. However, supplying one equivalent of LiTFA gave **8d** in 37% yield (Figure 3G). This restoration of reactivity could be attributed to the saturation of Ce with carboxylate ligand, such that unbound LiTFA is available to prime **6d** for oxidation by 9,10‐DPA*. To decouple the roles of the lithium cation and trifluoroacetate anion, we introduced each separately using salts with innocent counterions. Supplying one equivalent of LiBF_4_ resulted in no formation of **8d**, while [nBu_4_N][O_2_CCF_3_] yielded 17% of **8d**. As no reactivity was restored with stoichiometric Li^+^, and only partial reactivity returned with TFA^–^, it is clear that neither ion is wholly responsible for the effectiveness of LiTFA. Rather, Li^+^ and the weak trifluoroacetate base may act in synergy. This cooperative effect further supports a concerted MS‐PCET activation from the LiTFA•substrate complex.

We, along with others,^[^ ^30^, ^47^ ^]^ assumed that carboxylates bind to Ce, and our NMR and spectrophotometric studies suggest that trifluoroacetate indeed interacts with Ce(III) and Ce(IV). This complexation permits the possibility of Ce^IV^–O_2_CCF_3_ bond homolysis via photoinduced LMCT. The resultant CF_3_CO_2_^•^ radical could then act as an HAT catalyst by directly activating substrates (Figure 3H). Alternatively, CF_3_CO_2_^•^ could undergo decarboxylation to form a trifluoromethyl radical (^•^CF_3_), which could abstract hydrogen from the dicarbonyl (BDE(C–H)^[^ ^40^ ^]^ ≈88 kcal mol^−1^; BDE(F_3_C–H)^[^ ^48^ ^] ≈^106 kcal mol^−1^), initiating a radical chain reaction (Figure 3H). We reasoned that the catalytic application of LiTFA in our system, coupled with the slow decarboxylation of CF_3_CO_2_^•^ (1.59 × 10^5^ s^−1^ at 45 °C)^[^ ^49^ ^]^ and potential for BET (E_1/2_ (Ce^IV/III^) = +0.35 V versus SCE in MeCN, E_1/2_(CF_3_CO_2_^•^/CF_3_CO_2_^–^) =−2.4 V versus SCE in MeCN),^[^ ^50^ ^]^ would likely limit these pathways to minor or unproductive outcomes, competing with more direct substrate activation modes.

To probe the fate of trifluoroacetate, a stoichiometric photolysis of LiTFA and [Et_4_N]_2_[Ce^IV^Cl_6_] (1:1) was performed. After 5 minutes of irradiation, Ce(IV) was fully consumed, yielding only a single ^19^F resonance (Figure 3H). The same resonance was maintained throughout all subsequent manipulations, suggesting that there is no appreciable conversion of trifluoroacetate to fluoroform or any other fluorine‐containing byproduct. Given the kinetics of trifluoromethyl radical production, we reasoned that decarboxylation might still occur provided sufficient catalytic turnover events.

To determine whether ^•^CF_3_ was produced under either reaction condition (A or B), we chose to monitor the ^19^F NMR profile upon complete conversion of ketoester **4a** to ketolactone **5a**. Both conditions resulted in a ^19^F spectrum containing only a single resonance that subsequently coalesced with an additional LiTFA dose (Figure 3H), suggesting that trifluoroacetate is not appreciably deteriorated and is not a precursor to a chain‐initiating trifluoromethyl radical.

Controls omitting the disulfide co‐catalyst (TRIPS)_2_ maintained productive conversion (Table 1, entries 1 & 3). Given prior reports of formal HAT radical cyclizations proceeding via chain mechanisms, we deemed it prudent to examine whether such a process was contributing to our reaction.^[^ ^7^, ^8^ ^]^

To assess chain contribution, we used potassium ferrioxalate actinometry to find the quantum yield (Φ) for each reaction.^[^ ^51^, ^52^, ^53^ ^]^ These experiments gave quantum yields of Φ_Ce_ = 0.0028 ± 0.0009 and Φ_Ce‐free_ = 0.00125 ± 0.00009 (see SI for details). The low quantum yields (Φ ≪ 1) suggest that a chain reaction is unlikely but cannot be ruled out based on these experiments alone.^[^ ^52^ ^]^ Additionally, Julia's pioneering work on HAT cyclizations of C(sp^3^–H) acids shows that short, inefficient chains are possible.^[^ ^7^, ^13^, ^54^ ^]^

Reactions including TEMPO gave adduct **11** isolated in trace amounts that suggest the presence of radical intermediates (Figure 3I). BHT additives were found to attenuate the yield under both conditions, with a greater impact on condition A.

**Scheme 3:**
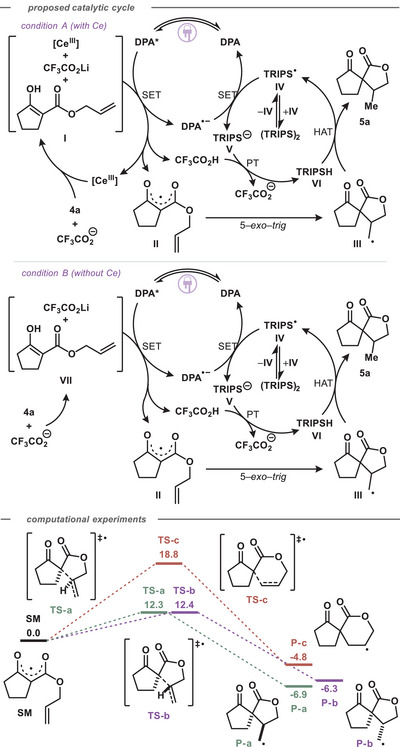
Proposed catalytic cycles for cerium‐containing and cerium‐free conditions; reaction coordinate showing cyclization transition states based on computational experiments (CBS‐QB3).

Based on these mechanistic investigations, we propose the following tentative catalytic cycles (Scheme 3). Under cerium‐employing conditions (A), we propose a ketoester•Ce(III) complex (**I**) is formed and is then subsequently oxidized by 9,10‐DPA* to give a ketoester•Ce(IV) complex and radical anion 9,10‐DPA^•–^ (E_1/2_(DPA*/DPA^•–^) = +1.28 V versus SCE in MeCN).^[^ ^39^ ^]^ The Ce(IV)•ketoester complex may then undergo thermal or photochemical LMCT, oxidizing the ketoester to furnish radical **II** and reducing Ce(IV) to Ce(III). 5‐*exo*‐*trig* cyclization gives a diastereomeric pair of [4.4]‐spirocyclic radicals (**III**). Concurrent with cyclization, 9,10‐DPA^•–^ reduces thiyl radical **IV**, simultaneously regenerating the 9,10‐DPA ground state and producing thiolate **V** (E_1/2_(DPA/DPA^•–^) = −1.80 V versus SCE in MeCN;^[^ ^39^ ^]^ E_1/2_(PhS^•^/PhS^–^) = +0.16 V vs SCE in MeCN).^[^ ^55^ ^]^ Thiolate **V** then deprotonates trifluoroacetic acid, regenerating carboxylate and yielding TRIPSH (**VI**) (pK_a_(S–H) ∼7 in water, pK_a_(CF_3_COO–H) ‐0.25 in water). HAT from TRIPSH (**VI**) to C‐centred radical **III** regenerates thiyl species **IV**, simultaneously producing closed‐shell ketolactone product **5a** (BDE(TRIPS–H) ∼83.5 kcal/mol, BDE (primary alkyl C–H) ∼100.3 kcal/mol, k_HAT_ ∼1 × 10^8^ M^−1^ s^−1^).^[^ ^56^, ^57^ ^]^

Under Ce‐free conditions, we propose that dicarbonyl substrates bind LiTFA, forming a PCET‐competent substrate•LiTFA complex **VII**. This complex (**VII**) can undergo a MS‐PCET with photoexcited 9,10‐DPA*, transferring a proton to LiTFA while reducing the photocatalyst simultaneously (BDE(PhO–H) ∼86.7 kcal/mol,^[^ ^58^ ^]^ effective BDFE ∼89.8 kcal/mol, see SI).^[^ ^59^, ^60^ ^]^ The remaining steps to complete the multi‐catalytic cycle operate identically to the cycles described for condition A.

To further understand the reaction pathway, we turned to computational experiments to better characterize the radical mechanism (see SI for details).^[^ ^61^, ^62^, ^63^, ^64^, ^65^, ^66^, ^67^, ^68^ ^]^ The key cyclization step is depicted in Scheme 3. The prohibitive 6.4 kcal/mol ∆∆G^‡^ difference between the higher‐energy 5*‐exo* transition state **TS–b** and 6*‐endo* TS **TS–c** aligns with the absence of any detectable [4.5]‐spirocyclic ketolactone product under the reaction conditions. The small 0.1 kcal mol^−1^ ∆∆G^‡^ between the two 5*‐exo* TSs (**TS–a** and **TS–b**) does not explain the experimentally obtained 4:1 dr, while the 0.6 kcal mol^−1^ ∆∆G between **P–a** and **P–b** is in good agreement.^[^ ^69^ ^]^ Of course, for the difference in **P–a** and **P–b** energies to be reflected in the product distribution there must be a negligible energy difference between the HAT transition states (see SI).

## 3 Conclusion

This photoredox catalyst platform, designed to employ 1,3‐dicarbonyl C(sp^3^)–H acids, unlocks divergent C‐centred radical reactions that offer new opportunities for building molecular complexity. In this manuscript, we delve into the wide range of substrates that can be functionalized under both cerium‐containing and cerium‐free conditions, discuss the limitations of the method, and detail the catalytic and mechanistic pathways that drive the reaction. Additionally, we highlight the various interception strategies available for these radicals.

Our scope studies revealed that subtle variations in substrate properties confer a preference for different reaction conditions. We attribute this divergence in activation to differences in binding equilibria, which lead to the formation of LMCT‐ or PCET‐competent complexes. CBS‐QB3 computational studies provided insight into reaction selectivity. We anticipate this hydroalkylation reaction finding use in accessing complex targets, particularly sp^3^‐rich scaffolds.

## 4 Experimental Section

### General procedure for intramolecular hydroalkylation

Inside a nitrogen‐filled glovebox, an 8 mL oven‐dried screw‐top Pyrex tube (12 mm diameter) was charged with lithium trifluoroacetate (0.020 mmol, 10 mol%), 9,10‐diphenylanthracene (0.010 mmol, 5 mol%), Ce(OTf)_3_ (0.020 mmol, 10 mol%, anhydrous), 2,4,6‐bis(triisopropylphenyl)disulfide (0.040 mmol, 20 mol%), tetrabutylammonium chloride (TBACl, 0.20 mmol, 1 equiv., anhydrous), and the dicarbonyl substrate (0.20 mmol, 1 equiv.). Ce(OTf)_3_ and TBACl were omitted for cerium‐free conditions (B). Once all components were added, the tube was sealed with a Teflon‐septum screw cap, transferred out of the glovebox, and immediately placed under a positive pressure of argon. Anhydrous acetonitrile (1.0 mL, 0.2 M) was added and the resulting suspension was sparged with argon for 5 minutes. After sparging, the septum cap was quickly exchanged with a hard plastic cap, and the vessel was sealed with electrical tape and then Parafilm. The reaction mixture was then sonicated (90 s) before irradiating with a violet LED (2–2.4 W, 410 nm) at a distance of 1–5 mm for 24 h. Upon complete conversion of starting material as determined by TLC, or if the conversion had stalled, the reaction solution was passed through a silica plug (3 cm^3^ in a 6 mL syringe barrel) eluting with Et_2_O (≈25 mL). The resulting solution was concentrated under reduced pressure and purified by flash column chromatography.

## Conflict of Interests

The authors declare no conflict of interest.

## Supporting information



Supporting Information

## Data Availability

The data that supports the findings of this study, including characterization data and NMR spectra for all compounds, are available in the supplemental material of this article. The authors have cited additional references within the Supporting Information.^[^ ^70^, ^71^, ^72^, ^73^, ^74^, ^75^, ^76^, ^77^, ^78^, ^79^, ^80^, ^81^, ^82^, ^83^, ^84^, ^85^, ^86^, ^87^, ^88^, ^89^, ^90^, ^91^, ^92^, ^93^, ^94^, ^95^, ^96^, ^97^, ^98^, ^99^, ^100^, ^101^, ^102^, ^103^, ^104^, ^105^, ^106^, ^107^, ^108^ ^]^ Deposition Numbers 2 416 734 (**5d**) contain the supplementary crystallographic data for this paper. These data can be obtained free of charge via the joint Cambridge Crystallographic Data Centre (CCDC).

## References

[1] L. Capaldo , D. Ravelli , Eur. J. Org. Chem. 2017, 2017, 2056. 10.1002/ejoc.201601485 PMC6099384 30147436

[2] T. Dalton , T. Faber , F. Glorius , ACS Cent. Sci. 2021, 7, 245. 33655064 10.1021/acscentsci.0c01413 PMC7908034

[3] T. E. O'Brien , A. O. Morris , L. F. Villela , L. Barriault , ChemCatChem 2023, 15, e202300989.

[4] J. J. A. Garwood , A. D. Chen , D. A. Nagib , J. Am. Chem. Soc. 2024, 146, 28034.

[5] F. Parsaee , M. C. Senarathna , P. B. Kannangara , S. N. Alexander , P. D. E. Arche , E. R. Welin , Nat. Rev. Chem. 2021, 5, 486. 37118440 10.1038/s41570-021-00284-3

[6] B. Chan , C. J. Easton , L. Radom , J. Phys. Chem. A 2015, 119, 3843. 25860917 10.1021/acs.jpca.5b01890

[7] M. Julia , Acc. Chem. Res. 1971, 4, 386.

[8] B. Giese , B. Kopping , T. Göbel , J. Dickhaut , G. Thoma , K. J. Kulicke , F. Trach , in Organic Reactions, Wiley, 2004, pp. 301–856.

[9] D. P. Curran , Synthesis 1988, 1988, 489.

[10] B. B. Snider , Chem. Rev. 1996, 96, 339. 11848756 10.1021/cr950026m

[11] J. Iqbal , B. Bhatia , N. K. Nayyar , Chem. Rev. 1994, 94, 519.

[12] D. P. Curran , M. H. Chen , E. Spletzer , C. M. Seong , C. T. Chang , J. Am. Chem. Soc. 1989, 111, 8872.

[13] D. P. Curran , C. T. Chang , J. Org. Chem. 1989, 54, 3140.

[14] D. P. Curran , T. M. Morgan , C. E. Schwartz , B. B. Snider , M. A. Dombroski , J. Am. Chem. Soc. 1991, 113, 6607.

[15] J. W. Tucker , J. D. Nguyen , J. M. R. Narayanam , S. W. Krabbe , C. R. J. Stephenson , Chem. Commun. 2010, 46, 4985. 10.1039/c0cc00981d 20512181

[16] Y. Kim , C.‐J. Li , Catal 2020, 1, 1.

[17] A. Y. Chan , I. B. Perry , N. B. Bissonnette , B. F. Buksh , G. A. Edwards , L. I. Frye , O. L. Garry , M. N. Lavagnino , B. X. Li , Y. Liang , E. Mao , A. Millet , J. V. Oakley , N. L. Reed , H. A. Sakai , C. P. Seath , D. W. C. MacMillan , Chem. Rev. 2022, 122, 1485. 34793128 10.1021/acs.chemrev.1c00383 PMC12232520

[18] J. Zhang , M. Rueping , Chem. Soc. Rev. 2023, 52, 4099. 37278288 10.1039/d3cs00023k

[19] M. S. Kharasch , E. V. Jensen , W. H. Urry , Science 1945, 102, 128. 10.1126/science.102.2640.128 17777366

[20] M. S. Kharasch , P. S. Skell , P. Fisher , J. Am. Chem. Soc. 1948, 70, 1055.

[21] K. C. Forbes , A. M. Crooke , Y. Lee , M. Kawada , K. M. Shamskhou , R. A. Zhang , J. S. Cannon , J. Org. Chem. 2022, 87, 3498. 35133155 10.1021/acs.joc.1c03055 PMC8898273

[22] S. Baś , Y. Yamashita , S. Kobayashi , ACS Catal. 2020, 10, 10546.

[23] N. Katta , Q.‐Q. Zhao , T. Mandal , O. Reiser , ACS Catal. 2022, 12, 14398. 36439036 10.1021/acscatal.2c04736 PMC9680001

[24] Y. Yamashita , Y. Ogasawara , T. Banik , S. Kobayashi , J. Am. Chem. Soc. 2023, 145, 23160. 37846890 10.1021/jacs.3c07436 PMC10603815

[25] B. P. Roberts , Chem. Soc. Rev. 1999, 28, 25.

[26] G. Lei , M. Xu , R. Chang , I. Funes‐Ardoiz , J. Ye , J. Am. Chem. Soc. 2021, 143, 11251. 34269582 10.1021/jacs.1c05852

[27] R. Zhao , L. Shi , Org. Chem. Front. 2018, 5, 3018.

[28] S. M. Treacy , T. Rovis , Synthesis 2023, 56, 1967. 38962497 10.1055/s-0042-1751518 PMC11218547

[29] F. Juliá , ChemCatChem 2022, 14, e202200916.

[30] Y. Chen , X. Wang , X. He , Q. An , Z. Zuo , J. Am. Chem. Soc. 2021, 143, 4896. 33756079 10.1021/jacs.1c00618

[31] J.‐J. Guo , A. Hu , Y. Chen , J. Sun , H. Tang , Z. Zuo , Angew. Chem. 2016, 128, 15545. 10.1002/anie.201609035 27862775

[32] J. Du , X. Yang , X. Wang , Q. An , X. He , H. Pan , Z. Zuo , Angew. Chem., Int. Ed. 2021, 60, 5370. 10.1002/anie.202012720 33259085

[33] Y. Aramaki , K. Chiba , M. Tada , Phytochemistry 1995, 38, 1419.

[34] J. F. Grove , J. MacMillan , T. P. C. Mulholland , M. A. T. Rogers , J. Chem. Soc. 1952, 762, 3977.

[35] V. Nair , A. Deepthi , Chem. Rev. 2007, 107, 1862. 17432919 10.1021/cr068408n

[36] A. O. Morris , L. Barriault , Chem. Eur. J. 2024, 30, e202400642. 38436591 10.1002/chem.202400642

[37] J. Friedrich , D. Schneider , L. Bock , C. Maichle‐Mössmer , R. Anwander , Inorg. Chem. 2017, 56, 8114. 28671467 10.1021/acs.inorgchem.7b00828

[38] H. Yin , Y. Jin , J. E. Hertzog , K. C. Mullane , P. J. Carroll , B. C. Manor , J. M. Anna , E. J. Schelter , J. Am. Chem. Soc. 2016, 138, 16266. 27936638 10.1021/jacs.6b05712

[39] R. E. Sioda , J. Phys. Chem. 1968, 72, 2322.

[40] H. P. Caldora , Z. Zhang , M. J. Tilby , O. Turner , D. Leonori , Angew. Chem., Int. Ed. 2023, 62, e202301656. 10.1002/anie.202301656 37016798

[41] L. L. Costanzo , S. Pistarà , G. Condorelli , J. Photochem. 1983, 21, 45.

[42] Q. Yang , Y.‐H. Wang , Y. Qiao , M. Gau , P. J. Carroll , P. J. Walsh , E. J. Schelter , Science 2021, 372, 847. 34016778 10.1126/science.abd8408

[43] A. Hu , Y. Chen , J. J. Guo , N. Yu , Q. An , Z. Zuo , J. Am. Chem. Soc. 2018, 140, 13580. 30289250 10.1021/jacs.8b08781

[44] G. Qiu , R. R. Knowles , J. Am. Chem. Soc. 2019, 141, 2721. 30665301 10.1021/jacs.8b13451 PMC6508549

[45] G. Qiu , R. R. Knowles , J. Am. Chem. Soc. 2019, 141, 16574. 31573194 10.1021/jacs.9b08398 PMC7206451

[46] K. Mori , M. Oshiba , T. Hara , T. Mizugaki , K. Ebitani , K. Kaneda , Tetrahedron Lett. 2005, 46, 4283.

[47] S. Shirase , S. Tamaki , K. Shinohara , K. Hirosawa , H. Tsurugi , T. Satoh , K. Mashima , J. Am. Chem. Soc. 2020, 142, 5668. 32109060 10.1021/jacs.9b12918

[48] J. C. Amphlett , J. W. Coomber , E. Whittle , J. Phys. Chem. 1966, 70, 593.

[49] H. Sawada , M. Nakayama , M. Yoshida , T. Yoshida , N. Kamigata , J. Fluorine Chem. 1990, 46, 423.

[50] S. Fernández‐García , V. O. Chantzakou , F. Juliá‐Hernández , Angew. Chem., Int. Ed. 2024, 63, e202311984. 10.1002/anie.202311984 38088503

[51] L. Buzzetti , G. E. M. Crisenza , P. Melchiorre , Angew. Chem., Int. Ed. 2019, 58, 3730. 10.1002/anie.201809984 30339746

[52] M. A. Cismesia , T. P. Yoon , Chem. Sci. 2015, 6, 5426. 26668708 10.1039/c5sc02185e PMC4676763

[53] C. Hatchard , C. A. Parker , Proc. R. Soc. London, Ser. A 1956, 235, 518.

[54] M. Julia , Pure Appl. Chem. 1974, 40, 553.

[55] A. G. Larsen , A. H. Holm , M. Roberson , K. Daasbjerg , J. Am. Chem. Soc. 2001, 123, 1723. 11456773 10.1021/ja003811b

[56] F. Dénès , M. Pichowicz , G. Povie , P. Renaud , Chem. Rev. 2014, 114, 2587. 24383397 10.1021/cr400441m

[57] Y.‐R. Luo , Comprehensive handbook of chemical bond energies, CRC press, 2007.

[58] P. Mulder , H.‐G. Korth , D. A. Pratt , G. A. DiLabio , L. Valgimigli , G. F. Pedulli , K. U. Ingold , J. Phys. Chem. A 2005, 109, 2647. 16833571 10.1021/jp047148f

[59] E. C. Gentry , R. R. Knowles , Acc. Chem. Res. 2016, 49, 1546. 27472068 10.1021/acs.accounts.6b00272 PMC5102158

[60] P. R. D. Murray , J. H. Cox , N. D. Chiappini , C. B. Roos , E. A. McLoughlin , B. G. Hejna , S. T. Nguyen , H. H. Ripberger , J. M. Ganley , E. Tsui , N. Y. Shin , B. Koronkiewicz , G. Qiu , R. R. Knowles , Chem. Rev. 2022, 122, 2017. 34813277 10.1021/acs.chemrev.1c00374 PMC8796287

[61] J. A. Montgomery, Jr. , M. J. Frisch , J. W. Ochterski , G. A. Petersson , J. Chem. Phys. 1999, 110, 2822.

[62] P. Pracht , S. Grimme , C. Bannwarth , F. Bohle , S. Ehlert , G. Feldmann , J. Gorges , M. Müller , T. Neudecker , C. Plett , S. Spicher , P. Steinbach , P. A. Wesołowski , F. Zeller , J. Chem. Phys. 2024, 160, 114110. 38511658 10.1063/5.0197592

[63] P. Pracht , F. Bohle , S. Grimme , Phys. Chem. Chem. Phys. 2020, 22, 7169. 32073075 10.1039/c9cp06869d

[64] Y. Zhao , D. G. Truhlar , Theor. Chem. Acc. 2008, 120, 215.

[65] Y. Zhao , D. G. Truhlar , Theor. Chem. Acc. 2008, 119, 525.

[66] M. J. Frisch , G. W. Trucks , H. B. Schlegel , G. E. Scuseria , M. A. Robb , J. R. Cheeseman , G. Scalmani , V. Barone , G. A. Petersson , H. Nakatsuji , X. Li , M. Caricato , A. V. Marenich , J. Bloino , B. G. Janesko , R. Gomperts , B. Mennucci , H. P. Hratchian , J. V. Ortiz , A. F. Izmaylov , J. L. Sonnenberg , Williams , F. Ding , F. Lipparini , F. Egidi , J. Goings , B. Peng , A. Petrone , T. Henderson , D. Ranasinghe , et al., Gaussian 16 Rev. C.01 (Wallingford, CT), 2016.

[67] C. Y. Legault , CYLview20 (Université de Sherbrooke), 2020.

[68] S. Grimme , C. Bannwarth , P. Shushkov , J. Chem. Theory Comput. 2017, 13, 1989. 28418654 10.1021/acs.jctc.7b00118

[69] K. Sung , Y. Y. Wang , J. Org. Chem. 2003, 68, 2771. 12662051 10.1021/jo026681t

[70] S. P. Pitre , C. D. McTiernan , W. Vine , R. DiPucchio , M. Grenier , J. C. Scaiano , Sci. Rep. 2015, 5, 16397. 26578341 10.1038/srep16397 PMC4649705

[71] G. L. Smith , A. A. Reutovich , A. K. Srivastava , R. E. Reichard , C. H. Welsh , A. Melman , F. Bou‐Abdallah , J. Inorg. Biochem. 2021, 220, 111460. 33866045 10.1016/j.jinorgbio.2021.111460

[72] Z. Marczenko , Spectrophotometric Determination of Elements, Ellis Horwood, 1976.

[73] W. Fortune , M. Mellon , Ind. Eng. Chem., Anal. Ed. 1938, 10, 60.

[74] P. Thordarson , Chem. Soc. Rev. 2011, 40, 1305. 21125111 10.1039/c0cs00062k

[75] P. Thordarson , Chem. 2012.

[76] C. Wetter , K. Jantos , K. Woithe , A. Studer , Org. Lett. 2003, 5, 2899. 12889903 10.1021/ol034994k

[77] E. Ota , H. Wang , N. L. Frye , R. R. Knowles , J. Am. Chem. Soc. 2019, 141, 1457. 30628777 10.1021/jacs.8b12552 PMC6508595

[78] C. F. Wise , R. G. Agarwal , J. M. Mayer , J. Am. Chem. Soc. 2020, 142, 10681. 32432468 10.1021/jacs.0c01032

[79] Z. Zhu , M. Odagi , C. Zhao , K. A. Abboud , H. U. Kirm , J. Saame , M. Lõkov , I. Leito , D. Seidel , Angew. Chem. Int. Ed. 2020, 59, 2028. 10.1002/anie.201912677 31710767

[80] V. V. Pavlishchuk , A. W. Addison , Inorg. Chim. Acta 2000, 298, 97.

[81] R. E. Visco , E. A. Chandross , J. Am. Chem. Soc. 1964, 86, 5350.

[82] L. R. Faulkner , A. J. Bard , J. Am. Chem. Soc. 1969, 91, 209.

[83] V. P. Bogdanov , V. A. Dmitrieva , V. A. Ioutsi , N. M. Belov , A. A. Goryunkov , J. Fluorine Chem. 2019, 226, 109344.

[84] B. B. Snider , J. E. Merritt , M. A. Dombroski , B. O. Buckman , J. Org. Chem. 1991, 56, 5544.

[85] L. Zhao , G. Huang , B. Guo , L. Xu , J. Chen , W. Cao , G. Zhao , X. Wu , Org. Lett. 2014, 16, 5584. 25325792 10.1021/ol502615y

[86] J. Cao , W. Thor , S. Yang , M. Zhang , W. Bao , L. Zhu , W. Yang , Y.‐K. Cheng , C.‐S. Lee , Org. Lett. 2019, 21, 4896. 31188619 10.1021/acs.orglett.9b01806

[87] M. W. Rathke , P. J. Cowan , J. Org. Chem. 1985, 50, 2622.

[88] D. Brillon , P. Deslongchamps , Can. J. Chem. 1987, 65, 43.

[89] W. Fang , M. Presset , A. Guérinot , C. Bour , S. Bezzenine‐Lafollée , V. Gandon , Org. Synth 2015, 92, 117.

[90] M. W. Ha , H. Lee , H. Y. Yi , Y. Park , S. Kim , S. Hong , M. Lee , M. H. Kim , T. S. Kim , H. G. Park , Adv. Synth. Catal. 2013, 355, 637.

[91] Y. Bai , W. Chen , Y. Chen , H. Huang , F. Xiao , G.‐J. Deng , RSC Adv. 2015, 5, 8002.

[92] S. K. Kristensen , S. L. Laursen , E. Taarning , T. Skrydstrup , Angew. Chem., Int. Ed. 2018, 57, 13887. 10.1002/anie.201809051 30178905

[93] A. Luque , J. Groß , T. J. Zähringer , C. Kerzig , T. Opatz , Chem.‐Eur. J. 2022, 28, e202104329. 35133690 10.1002/chem.202104329 PMC9314945

[94] M. W. Löble , J. M. Keith , A. B. Altman , S. C. E. Stieber , E. R. Batista , K. S. Boland , S. D. Conradson , D. L. Clark , J. L. Pacheco , S. A. Kozimor , R. L. Martin , S. G. Minasian , A. C. Olson , B. L. Scott , D. K. Shuh , T. Tyliszczak , M. P. Wilkerson , R. A. Zehnder , J. Am. Chem. Soc. 2015, 137, 2506. 25689484 10.1021/ja510067v

[95] F. Swarts , Bull. Soc. Chim. Belg. 1939, 48, 176.

[96] H. Helmboldt , D. Köhler , M. Hiersemann , Org. Lett. 2006, 8, 1573. 16597113 10.1021/ol060115t

[97] H. Li , J. S. Dickschat , Chem.‐Eur. J. 2024, 30, e202303560. 37947363 10.1002/chem.202303560

[98] R. Hara , T. Furukawa , H. Kashima , H. Kusama , Y. Horiguchi , I. Kuwajima , J. Am. Chem. Soc. 1999, 121, 3072.

[99] D. Young , W. Kitching , G. Wickham , Aust. J. Chem. 1984, 37, 1841.

[100] B. Wang , L. Zhang , K. Fu , Y. Luo , W. Lu , J. Tang , Org. Prep. Proced. Int. 2009, 41, 309.

[101] R. J. Mart , K. P. Liem , X. Wang , S. J. Webb , J. Am. Chem. Soc. 2006, 128, 14462. 17090021 10.1021/ja0657612

[102] M. D. Rackham , J. A. Brannigan , D. K. Moss , Z. Yu , A. J. Wilkinson , A. A. Holder , E. W. Tate , R. J. Leatherbarrow , J. Med. Chem. 2013, 56, 371. 23170970 10.1021/jm301474t PMC3601602

[103] S. Chowdhury , G. Chauhan , A. Kumar , B. Chaturvedi , C. Behera , Eur. J. Org. Chem. 2022, 2022, e202200850.

[104] R. J. Maza , E. Davenport , N. Miralles , J. J. Carbó , E. Fernández , Org. Lett. 2019, 21, 2251. 30859836 10.1021/acs.orglett.9b00531

[105] Y. Zhu , I. Colomer , A. L. Thompson , T. J. Donohoe , J. Am. Chem. Soc. 2019, 141, 6489. 30977361 10.1021/jacs.9b02198

[106] E. Rideau , H. You , M. Sidera , T. D. W. Claridge , S. P. Fletcher , J. Am. Chem. Soc. 2017, 139, 5614. 28362495 10.1021/jacs.7b02440

[107] J. A. Morales‐Serna , E. García‐Ríos , J. Bernal , E. Paleo , R. Gaviño , J. Cardenas , Synthesis 2011, 2011, 1375.

[108] A. A. Folgueiras‐Amador , A. E. Teuten , M. Salam‐Perez , J. E. Pearce , G. Denuault , D. Pletcher , P. J. Parsons , D. C. Harrowven , R. C. Brown , Angew. Chem., Int. Ed. 2022, 61, e202203694. 10.1002/anie.202203694 PMC9543573 35790060

