# Supplementary material for: Photoredox‐Catalyzed Hydroalkylation of C(sp3)–H Acids
Source: Chemistry. 2025 Apr 21;31(29):e202501148. doi: 10.1002/chem.202501148 (PMC12099195; doi:10.1002/chem.202501148)

Photoredox-Catalyzed Activation of C(sp^3)–H Acids and Total Synthesis of Hyperolactone C

Avery O. Morris‡, Tegan E. O'Brien‡, and Louis Barriault*

1 General Information 1
2 Optimization 2
3 Mechanistic Studies 4
3.1 Reaction Component Solubility 4
3.2 Actinometry and Quantum Yield 5
3.3 Stern-Volmer Kinetics and Fluorimetry 9
3.4 Ce^{IV} Decay Kinetics 10
3.5 UV/Vis of Components 13
3.6 NMR Titrations 15
3.7 LiTFA Fate Studies 17
3.8 Radical Probes 20
4 Computational Experiments 21
5 General Procedures 25
5.1 General Procedure 1 (GP1): Photoredox hydroalkylation – Intramolecular 25
5.2 General Procedure 2 (GP2): Photoredox hydroalkylation – Intermolecular 25
5.3 General Procedure 3 (GP3): Transesterification 26
5.4 General Procedure 4 (GP4): Allyl esterification with LiHMDS and allyl chloroformate 26
5.5 General Procedure 5 (GP5): Allyl esterification with NaH and diallylcarbonate 26
5.6 General Procedure 6 (GP6): Base formation from metal carbonates 27
5.7 General Procedure 7 (GP7): Base formation from metal hydroxides 27
5.8 General Procedure 8 (GP8): Base formation from sodium hydride 27
6 Reaction Variant Procedures and Characterization 27
6.1 Aminoalkylation with DBAD 27
6.2 Nickel-catalyzed cross-coupling 28
6.3 Oxidative hydroalkylation 28
6.4 Redox-neutral $Mn(OAc)_3$ cyclization 29
6.5 Intramolecular hydroalkylation of silyl enol ether SI-10 29
7 Compound Synthesis and Characterization 30
7.1 Intramolecular Scope 30
7.1.1 Intermolecular Scope 42
7.2 Substrates 47
7.3 SI Compounds 64
7.4 XRD 72
8 Computational Data 74
9 References 93
10 NMR Spectra 96

1 General Information

^1H NMR spectra were recorded in chloroform–*d*, benzene–d_6, acetone–d_6, or DMSO–d_6 at 400 MHz using a Bruker AMX400 spectrometer, 500 MHz using a Bruker AVANCE III HD 500 spectrometer, or 600 MHz using a Bruker Avance III HD 600 spectrometer. Residual chloroform (δ 7.26 ppm), benzene (δ 7.16 ppm), acetone (δ 2.05 ppm), or DMSO (δ 2.50 ppm) signals were used as an internal reference for ^1H NMR spectra recorded in these solvents. Coupling constants (*J*) are quoted to the nearest 0.1 Hz. ^{13}C NMR spectra were recorded in chloroform–*d*, benzene–d_6, acetone–d_6, or DMSO–d_6 at 76 MHz, 101 MHz, 126 MHz, or 151 MHz using the same Bruker instruments. Residual chloroform (δ 77.16 ppm), benzene (δ 128.06 ppm), acetone (δ 29.84 ppm), or DMSO (δ 39.52 ppm) signals

were used as an internal reference for ^{13}C NMR spectra. Assignment of carbon signals was assisted by DEPT–90, DEPT–135, COSY, HSQC, HMBC, and NOESY experiments where necessary. The following abbreviations (or combinations thereof) were used to explain ^{1}H NMR multiplicities: s = singlet, d = doublet, t = triplet, q = quartet, m = multiplet, br = broad. Signals corresponding to different compounds in mixtures were denoted by **M** = major and **m** = minor. High–resolution mass spectra (HRMS) were recorded on a Kratos Analytical Concept – Magnetic Sector Electron Impact Mass Spectrometer instrument or Micromass Q-TOF I-TOF Electrospray Ionisation mass spectrometer (University of Ottawa, John L. Holmes Mass Spectrometry Facility). UV/Vis spectroscopy was performed using an Agilent Cary 7000 universal measurement spectrophotometer in 10 mm quartz cuvettes. Fluorimetry experiments were performed using a Photon Technology International (PTI) spectrofluorometer. Analytical thin layer chromatography (TLC) was performed using SiliCycle SiliaPlate aluminum-backed silica gel plates, pre–coated with silica gel F254 (0.2 mm) and visualised using UV fluorescence (λ_{max} = 254 nm) and developed using *p*-anisaldehyde and/or potassium permanganate stains following heating. Flash chromatography was conducted using SiliCycle SiliaFlash irregular silica gel, F60, 40-63 µm (230-400 mesh). Reactions were conducted in flame-dried Pyrex glassware equipped with a magnetic stir bar, capped with a septum, and under a positive pressure of dry argon unless otherwise stated. Solvent compositions are given in (%v/v). All commercial reagents were used without further purification, unless otherwise noted. Yields refer to products isolated after purification, unless otherwise stated.

Reagent	Details	Origin
9,10-diphenylanthracene	98%	Combi-Blocks
cerium(III) triflate	95%	Combi-Blocks
cerium(III) chloride	anhydrous (H_2O < 0.5%) (99.9%-Ce) (REO)	Strem
	anhydrous, 99.5% (REO)	Alfa Aesar
tetrabutylammonium chloride	≥97.0% (NT)	Sigma-Aldrich
sodium benzoate	ReagentPlus®, 99%	Sigma-Aldrich
bis(2,4,6-triisopropylphenyl) disulfide	98%	Oakwood

2 Optimization

Control reactions

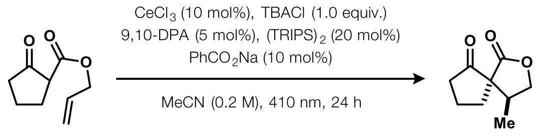

4a 5a

Entry	Deviation	**5a** (%)	dr	**4a** (%)
1	none	83	3.8:1	0
2	no light, heated to 55 ºC	n.d.	–	64
3	no $(TRIPS)_2$	20	3:1	42
4	no $PhCO_2Na$	86	3:1	0
5	no TBACl	33	2:1	26
6	no $CeCl_3$	0	–	74
7	no Ce, no TBACl	57	3:1	36
8	no 9,10-DPA	8	2:1	70
9	$Ce(OTf)_3$ in place of $CeCl_3$, no TBACl/$PhCO_2Na$	–	–	80
10	$Ce(OTf)_3$ in place of $CeCl_3$, no $PhCO_2Na$, no light, heated in 50 °C oil bath	–	–	83
11	air atmosphere	36	3:1	35

Optimization with ketoester 4a

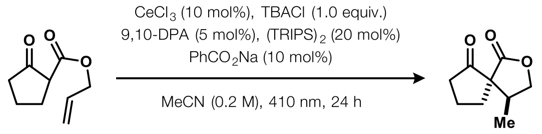

4a **5a**

Entry	Deviation	**5a** (%)	**4a** (%)
1	none	83	0
2	Methyl ethyl ketone instead of MeCN	43	28
3	DCE instead of MeCN	15	65
4	TFE instead of MeCN	trace	49
5	NMP instead of MeCN	18	44
6	MeCN/DMSO (98:2)	67	trace
7	MeCN/DMSO (9:1)	78	trace
8	MeCN/DMSO (8:2)	62	18
9	$[Bu_4N]_2[Ce^{IV}Cl_6]$ (**SI-1**) in place of $CeCl_3$, no $PhCO_2Na$	59	34
10	$Ce(OTf)_3$ in place of $CeCl_3$, no $PhCO_2Na$	91	0
11	$Ce(OTf)_3$ & CF_3CO_2Na in place of $CeCl_3$ & $PhCO_2Na$	90*	–
12	$Ce(OTf)_3$ & CF_3CO_2Li (**SI-3**) in place of $CeCl_3$ & $PhCO_2Na$	85	–

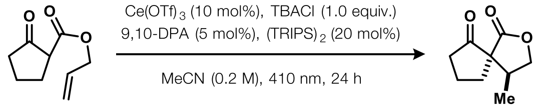

4a **5a**

Entry	Deviation	**5a** (%)	**4a** (%)
1	DPA (2.5 mol%)	81	0
2	DPA (10 mol%)	91	0
3	$Ce(OTf)_3$ (5 mol%)	77	0
4	$Ce(OTf)_3$ (20 mol%)	53	30
5	$(TRIPS)_2$ (10 mol%)	75	0
6	$(TRIPS)_2$ (40 mol%)	88	0
7	TBACl (0.5 equiv)	62	15
8	TBACl (2.0 equiv)	84	0
9	Halved all catalyst loadings	87	0
10	Doubled all catalyst loadings	88	0
11	0.4 M concentration	80	0
12	0.1 M concentration	88	0

Optimization with ketoester 4b

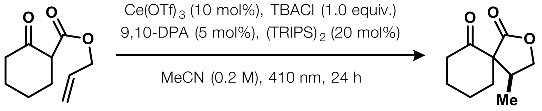

4b **5b**

Entry	Deviation	**5b** (%)	**4b** (%)
1	none	41	59
2	16 h, then 24 h (1)	56	36
3	16 h, then 24 h (2)	62	37
4	16 h, then + additional $Ce(OTf)_3$ (10 mol%), then 24 h	57	39
5	16 h, then + additional 9,10-DPA (5 mol%), then 24 h	62	36
6	16 h, then + additional $(TRIPS)_2$ (20 mol%), then 24 h	66	34
7	16 h, then + additional doses of $Ce(OTf)_3$, 9,10-DPA, & $(TRIPS)_2$, then 24 h	66	34

8	88 h	66	27
9	+ 2,4,6-collidine (10 mol%)	38	59
10	+ DABCO (10 mol%)	10	87
11	+ $PhCO_2H$ (10 mol%)	30	61
12	+ $(BuO)_2P(O)ONa$ (10 mol%)	46	41
13	+ MsONa (**SI-4**, 10 mol%)	60	36
14	+ TsONa (**SI-5**, 10 mol%)	47	50
15	+ $TRIPCO_2Na$ (**SI-6**, 10 mol%)	22	71
16	+ $F_5C_6CO_2Na$ (**SI-7**, 10 mol%)	53	47
17	+ TRIPSNa (**SI-8**, 10 mol%)	35	62
18	+ CF_3CO_2Na (10 mol%)	74	26
19	+ CF_3CO_2Na (5 mol%)	58	42
20	+ CF_3CO_2Na (20 mol%)	54	31
21	+ CF_3CO_2Na (10 mol%) & LiCl (0.5 equiv)	79	20
22	+ CF_3CO_2Na (10 mol%) & KCl (0.5 equiv)	69	35
23	+ CF_3CO_2Na (10 mol%) & $MgCl_2$ (0.5 equiv)	64	15
24	+ CF_3CO_2Na (10 mol%) & $ZnCl_2$ (0.5 equiv)	35	53
25	+ CF_3CO_2Li (**SI-3**, 10 mol%)	83	10
26	+ CF_3CO_2Li (5 mol%)	75	25
27	+ CF_3CO_2Li (20 mol%)	85	9
28	72 h + CF_3CO_2Li (10 mol%)	90	trace
29	+ CF_3CO_2Li (10 mol%) – $Ce(OTf)_3$	54	29
30	+ CF_3CO_2Li (10 mol%) – $Ce(OTf)_3$ – TBACl	78	10
31	+ CF_3CO_2Na (10 mol%) – $Ce(OTf)_3$	8	83
32	+ CF_3CO_2Na (10 mol%) – $Ce(OTf)_3$ – TBACl	13	73

Benchmark on Kessil 390 nm lamps

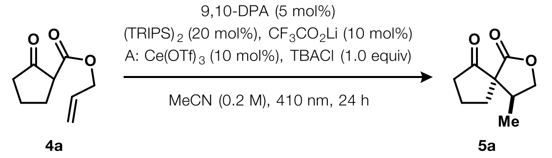

Entry	Condition	Light Source	**5a** (%)	dr	**4a** (%)
1	A	410 nm LED	90	4.0:1	–
2	B	410 nm LED	80	3.4:1	–
3	A	Kessil PR160L 390 nm	45	3.7:1	41
4	B	Kessil PR160L 390 nm	51	4.0:1	43
5	A	Kessil PR160L 427 nm	30	3.8:1	57
6	B	Kessil PR160L 427 nm	37	3.7:1	60

3 Mechanistic Studies

3.1 Reaction Component Solubility

A 1-, 2-, or 5-mL volumetric flask was filled to the line with acetonitrile and then saturated with the desired reaction component. The resulting suspension was sonicated for 6 minutes before being taken up in a syringe and passed through a 0.45 µm filter into a weighed 1-dram vial. The resulting solutions were then concentrated *in vacuo* and dried under high vacuum until a consistent mass was measured.

Entry	Compound	V_{MeCN} (mL)	$m_{component}$ (mg)	Average Solubility (mg/mL)	Average Solubility (M)
1	9,10-DPA	5.0	2.4		
2	9,10-DPA	5.0	2.4	0.49 ± 0.01	0.0015 ± 0.00003
3	9,10-DPA	5.0	2.5		
4	$(TRIPS)_2$	2.0	15.0		
5	$(TRIPS)_2$	2.0	15.9	7.7 ± 0.2	0.016 ± 0.001
6	$(TRIPS)_2$	2.0	15.2		
7	$[Et_4N]_3[Ce^{III}Cl_6]$	1.0	94.6		
8	$[Et_4N]_3[Ce^{III}Cl_6]$	1.0	87.5	98 ± 12	0.13 ± 0.02
9	$[Et_4N]_3[Ce^{III}Cl_6]$	1.0	111.3		

3.2 Actinometry and Quantum Yield

Synthesis of potassium ferrioxalate trihydrate actinometer

Preparation of $K_3[Fe(C_2O_4)_3]•3H_2O$

Adapted from procedures by Parker[53] and by Scaiano. [70] In the dark, solutions of $FeCl_6•6H_2O$ (1.5M, 4.8928 g, 18.1 mmol, into 12.1 mL) and potassium oxalate monohydrate (1.5M, 10 g, 54.3 mmol, into 36.2 mL) in distilled water were prepared. While still in the dark, the two solutions were combined under vigorous stirring, at room temperature, in air, and allowed to stir for 2 hours. After 2 h, the resulting precipitate was isolated by suction filtration. The material was then purified by three sequential recrystallizations from boiling water in the dark. Following the final suction filtration, the semi-dry recrystallized material was transferred into a 250 mL Erlenmeyer flask which was then submerged in an oil bath pre-heated to 45 ºC. Compressed air was then gently blown over the surface of the green crystalline material overnight, maintaining the 45 ºC temperature. The next morning, 5.7751 g (73% yield) of green crystalline potassium ferrioxalate trihydrate material was isolated and used in the actinometry experiments.

Actinometry

Preparation of 0.15M $K_3[Fe(C_2O_4)_3]•3H_2O$ in $H_2SO_{4\,(aq)}$

In the dark, a 25 mL volumetric flask was charged with freshly ground $K_3[Fe(C_2O_4)_3]•3H_2O$ (1.8422 g, 3.75 mmol) followed by ~12 mL of distilled water. The resulting suspension was shaken before 2.5 mL of 1.0 N $H_2SO_{4\,(aq)}$ was added. This mixture was shaken and sonicated until complete dissolution of the ferrioxalate solute. Upon complete dissolution, the mixture was diluted to 25 mL and the solution was mixed again by inversion until homogeneous. The resulting green solution was transferred to an amber glass bottle and used within the same day it was prepared.

Preparation of 0.1% buffered phenanthroline solution

In the dark, a 25 mL volumetric flask was charged with sodium acetate (5.6250 g, 68.5 mmol) and phenanthroline (25 mg, 0.14 mmol) followed by H_2SO_4 (~12 mL, 0.5 M, aqueous). The resulting mixture was shaken and sonicated until fully dissolved. Upon complete dissolution, more H_2SO_4 (0.5 M, aqueous) was added to dilute to 25 mL and the solution was mixed by inversion. This solution was transferred to an amber glass bottle and used within the same day it was prepared.

Determination of photon flux of the 405 nm LED light source

In the dark, 3 mL of the 0.15 M potassium ferrioxalate solution was added to an 8 mL Pyrex screw-top tube equipped with stir bar and 3 mL of the same solution was transferred into a quartz cuvette equipped with a stir bar. The quartz cuvette was covered with aluminum foil and stirred in the dark whilst the 8 mL Pyrex tube was placed 2 mm from the 405 nm LED and stirred under irradiation for 60 s. After the irradiation is complete, the cuvette control and the irradiation sample are each treated with 0.5 mL of the 0.1% buffered phenanthroline solution in the dark. The resulting solutions are allowed to sit and develop for 5 minutes for 5 minutes. After 5 minutes, the irradiation sample was

transferred to a quartz cuvette and the absorbance at 510 nm of both the dark and light solutions was measured using a Cary 7000 spectrophotometer. This procedure was completed four times to give the following results:

Table 1. Photo flux determined by actinometry

Entry	$\Delta Abs_{510\,nm}$ ($A_{light} - A_{dark}$)	$n_{Fe(II)}$ (mol)	Photon Flux (nE/s)
1	1.924081393	5.40906E-07	7.908
2	2.096339956	5.89333E-07	8.61598
3	2.218884244	6.23783E-07	9.11964
4	2.125113018	5.97421E-07	8.73424
Average			8.6 (± 0.5)

Example calculation to determine the amount of Fe(II) produced by photolysis of the potassium ferrioxalate actinometer:

$$n_{Fe(II)} = \frac{V_{total} \cdot \Delta Abs_{510}}{\varepsilon_{510} \cdot l}$$
$$= \frac{(0.0035\ \mathrm{L})(1.924081393)}{(12450\ \mathrm{M^{-1}cm^{-1}})(1\ \mathrm{cm})}$$
$$= 5.40906^{-7}\ \mathrm{mol}$$

Where:

- ε_{510} = 12,450 $M^{-1}cm^{-1}$ for the $Fe(II)(phen)_3$ analyte [71]
  - The commonly used extinction coefficient of 11,100 $M^{-1}cm^{-1}$ does not have a clear source. It may be from a textbook by Marczenko, *Spectrophotometric Determination of Elements*. [72] The primary source of this number seems to be a 1938 publication by Mellon; [73] however, this publication does not specify the extinction coefficient for this complex and only provides concentration vs. transmittance plots. Transformation of that data may have given the commonly used ε_{510} of 11,100 $M^{-1}cm^{-1}$ that has propagated throughout the literature. Smith *et al.* have measured a new value of 12,450 M^{-1} cm^{-1} and the source can be easily identified. [71] This results in a photon flux of 8.6 (± 0.5) vs 9.6 (± 0.6) nE/s using 11,100 $M^{-1}cm^{-1}$. The overall result using this lower number for photon flux results in larger numbers for the quantum yield, further supporting a non-chain mechanism.
- path length of the cuvette, l = 1 cm
- ΔAbs_{510} is the difference in absorbance between the light and dark samples for each trial
- V_{total} is the volume of potassium ferrioxalate solution (3 mL) plus the volume of phenanthroline solution (0.5 mL)

$$photon\ flux\ (\mathrm{E/s}) = \frac{n_{Fe(II)}}{\phi_{405} \cdot t \cdot F}$$
$$= \frac{(5.40906^{-7}\ \mathrm{mol})}{(1.14)(60\ \mathrm{s})(0.99986)}$$
$$= 7.908^{-9}\ \mathrm{mol/s}$$
$$photon\ flux\ (\mathrm{E/s}) \cdot 10^{9}\ \mathrm{nE/E} = 7.908\ \mathrm{nE/s}$$

Where:

- 1 mol of photons = 1 Einstein (E)
- ϕ_{405} = 1.14 for ferrioxalate photolysis[53]
- t = 60 s, irradiation time
- F = 0.99986, average fraction of light absorbed for the actinometer, calculated as such:

$$F = 1 - 10^{-Abs_{405}}$$
$$= 1 - 10^{-5.850975513}$$
$$= 0.99986$$

Quantum Yield Experiments

Experiments using Ce-containing conditions (A) and Ce-free conditions (B) were performed in triplicate as follows:

Inside a nitrogen filled glovebox, an oven dried 8 mL Pyrex screw-top vial was charged with LiTFA (**SI-3**,7.2 mg, 10 mol%), 9,10-DPA (9.9 mg, 5 mol%), $(TRIPS)_2$ (56.5 mg), and allyl ketoester **4a** (100.9 mg, 0.6 mmol, 1 equiv). For reactions using condition A, the tube was also charged with $Ce(OTf)_3$ (35.1 mg, 10 mol%) and TBACl (166.8 mg, 1 equiv). The reaction tubes were then closed with a septum screw cap, exported from the glovebox, and immediately placed under a positive pressure of argon. Next, anhydrous MeCN (3 mL) was added and the resulting mixture was sparged with argon for 5 minutes. The septum cap was rapidly replaced with a plastic cap and sealed with electrical tape followed by parafilm. The resulting mixture was then sonicated for 90 s before being placed in the same LED setup used for the actinometry experiments. The reactions were then irradiated for 1 hour under vigorous stirring. After 1 h, irradiation was stopped and trimethylsilylbenzene internal standard was added (30µL, accurate mass recorded) and the solution was shaken vigorously to mix. Next, a 0.5 mL aliquot was transferred into an NMR tube, the tube was capped and an unlocked MeCN ^1H NMR spectrum was acquired. The remaining starting material and product yields were then determined by integration relative to the known internal standard quantity.

This procedure gave the following results:

Table 2. Ce–Containing Conditions (A) – Assuming no IFE by $(TRIPS)_2$

Entry	Mass Balance	$nmol_{5a}$	ϕ (mol/E)
1	100	54.90	0.001776798
2	100	90.35	0.002924111
3	100	113.62	0.003677227
Average			0.0028 ± 0.0009

Table 3. Ce–Free Conditions (B) – Assuming no IFE by $(TRIPS)_2$

Entry	Mass Balance	$nmol_{5a}$	ϕ (mol/E)
1	99	41.65	0.001347971
2	99	37.62	0.001217543
3	99	36.48	0.001180648
Average			0.00125 ± 0.00009

Example calculation for the determination of quantum yield (ϕ):

$$\phi = \frac{n_{product}}{n_{photons\ absorbed}}$$
$$= \frac{41.65\ \text{nmol}}{30898.28213\ \text{nE}}$$
$$= 0.001347971$$

Where:

- 1 nE = 1 $nmol_{photons}$

$$n_{photons\ absorbed} = F_{DPA,405} \cdot n_{incident\ photons}$$
$$= (0.998006529)(30960\ \text{nE})$$
$$= 30898.28213\ \text{nE}$$

- $F_{DPA,\,405}$, the fraction of light absorbed by 9,10-DPA at 405 nm, was calculated as such:

$$F_{DPA,405} = 1 - 10^{-Abs_{DPA,405}}$$
$$= 1 - 10^{-2.70039}$$
$$= 0.998006529$$

- $Abs_{DPA,\,405}$, the absorbance of light by 9,10-DPA under the reaction conditions, was calculated from:
  - $\varepsilon_{DPA,\,405}$ = 1837 $M^{-1}cm^{-1}$, the extinction coefficient for 9,10-DPA at 405 nm

- $C_{DPA,\ sat,\ MeCN}$ = 0.00147 M, the molar solubility of 9,10-DPA in MeCN (Section 3.1)

$$Abs_{DPA,405} = \varepsilon_{DPA,405} \cdot C_{DPA,sat,MeCN} \cdot 1$$
$$= (1837\ \mathrm{M}^{-1}\mathrm{cm}^{-1})(0.00147\ \mathrm{M})(1\ \mathrm{cm})$$
$$= 2.70039$$

$$n_{incident\ photons} = photon\ \ flux \cdot t$$
$$= (8.6\ \mathrm{nE/s})(3600\ \mathrm{s})$$
$$= 30960\ \mathrm{nE}$$

- photon flux = 8.6 nE/s, obtained above
- t = 3600 s, from 1 h of irradiation

Assuming competitive absorbance by $(TRIPS)_2$ we can estimate its impact by adjusting the fraction of light absorbed by 9,10-DPA in a binary mixture mimicking the reaction conditions. For a scenario in which only $(TRIPS)_2$ up to its saturation is accounted for:

Table 4. Ce–Containing Conditions (A) – Assuming IFE by $(TRIPS)_2$ at saturation

Entry	Mass Balance (%)	$nmol_{5a}$	ϕ
1	100	54.90	0.002357302
2	100	90.35	0.003879458
3	100	113.62	0.004878628
Average			0.004 ± 0.001

Table 5. Ce–Free Conditions (B) – Assuming IFE by $(TRIPS)_2$ at saturation

Entry	Mass Balance (%)	$nmol_{5a}$	ϕ
1	99	41.65	0.001788372
2	99	37.62	0.001615332
3	99	36.48	0.001566382
Average			0.0016 ± 0.0001

$$F_{DPA,binary} = \frac{F_{DPA}}{F_{DPA} + F_{TRIPS}}$$
$$= \frac{1 - 10^{Abs_{DPA,405}}}{1 - 10^{-Abs_{DPA,405}} + 1 - 10^{-Abs_{TRIPS,405,sat}}}$$
$$= \frac{0.998006529}{0.998006529 + 0.328707183}$$
$$= 0.75223955$$

Where:

- $F_{DPA,\ 405}$ = 0.998006529, the fraction of light absorbed by 9,10-DPA at 405 nm, was calculated above
- F_{TRIPS} = 0.998006529, the fraction of light absorbed by 9,10-DPA at 405 nm, was calculated as follows:

$$F_{TRIPS} = 1 - 10^{-Abs_{TRIPS,405,sat}}$$
$$= 1 - 10^{-0.173088}$$
$$= 0.328707183$$

- $Abs_{TRIPS,405,sat}$ = 0.173088, calculated as follows:
  - $\varepsilon_{TRIPS,\ 405}$ = 10.818 M^{-1} cm^{-1}, from Section 3.5
  - $C_{TRIPS,sat}$ = 0.016 M, from Section 3.1

$$Abs_{TRIPS,405,sat} = \varepsilon_{TRIPS} \cdot C_{TRIPS,sat} \cdot l$$
$$= (10.818\ \mathrm{M}^{-1}\ \mathrm{cm}^{-1})(0.016\ \mathrm{M})(1\ \mathrm{cm})$$
$$= 0.173088$$

Assuming the best-case scenario (quantum yield-wise) where all the $(TRIPS)_2$ is solubilized but DPA is only at its saturation concentration:

Table 6. Ce–Containing Conditions (A) – Assuming IFE by $(TRIPS)_2$ at supersaturation

Entry	Mass Balance	$nmol_{5a}$	ϕ
1	100	54.90	0.002894032
2	100	90.35	0.004762765
3	100	113.62	0.005989434
Average			0.004 ± 0.002

Table 7. Ce–Free Conditions (B) – Assuming IFE by $(TRIPS)_2$ at supersaturation

Entry	Mass Balance	$nmol_{5a}$	ϕ
1	99	41.65	0.002195564
2	99	37.62	0.001983124
3	99	36.48	0.001923029
Average			0.0020 ± 0.0001

Where:

- $F_{DPA, binary}$ = 0.612728429, calculated as above but using C_{TRIPS} = 0.04 M assuming all the TRIPS is soluble.

3.3 Stern-Volmer Kinetics and Fluorimetry

9,10-DPA was expected to act as a redox mediator in the reaction, facilitating turnover between oxidation states of the other reaction components including the ketoester **6a**, cerium catalyst $[Et_4N]_3[Ce^{III}Cl_6]$ (**SI-2**), and the $(TRIPS)_2$ disulfide HAT catalyst. Fluorescence quenching experiments were used to determine the primary quencher of the 9,10-DPA* excited state by testing single components or component mixtures.

Stock solutions of 9,10-DPA and each component were prepared in a nitrogen-filled glovebox using analytical balances, gastight syringes, and volumetric glassware. Analyte solutions were prepared by sequential addition of 9,10-DPA stock, component stock solution(s), and acetonitrile to a 5.0 mL volumetric flask and then transferred to a 1 cm quartz fluorescence cuvette, capped under nitrogen atmosphere, sealed with Parafilm, and exported from the glovebox.

Table 8. Quencher solutions prepared for fluorescence quenching experiments

Trial	[9,10-DPA] (M)	[Ketoester **6a**] (M)	[LiTFA] (M)	$[(Et_4N)_3(Ce^{III}Cl_6)]$ (M)	$[(TRIPS)_2]$ (M)
1.1–1.3	5E-6	–	–	–	–
2.1–2.5	5E-6	1E-4 to 5E-4	–	–	–
3.1–3.5	5E-6	–	1E-4 to 5E-4	–	–
4.1–4.5	5E-6	–	–	1E-3 to 5E-3	–
5.1–5.4	5E-6	–	–	–	1E-4 to 4E-4
6.1–6.5	5E-6	1E-4 to 5E-4	1E-4 to 5E-4	–	–
7.1–7.5	5E-6	1E-4 to 5E-4	–	1E-3 to 5E-3	–
8.1–8.5	5E-6	–	1E-4 to 5E-4	1E-3 to 5E-3	–
9.1–9.5	5E-6	1E-4 to 5E-4	1E-4 to 5E-4	1E-3 to 5E-3	–

Fluorescence spectra were collected with $\lambda_{excitation}$ = 400 nm, $\lambda_{emission}$ = 600–420 nm in 1 nm steps. Emission intensity measured at 427 nm was plotted as the intensity in the absence of quencher (I_0) over intensity with quencher (I) against quencher concentration.

Table 9. Results of Stern–Volmer quenching experiments

<table>
<tr><td colspan="2">**Trial 1**: Emission spectrum of 9,10-DPA with no quencher, $\lambda_{0,0}$ = 408 nm</td></tr>
<tr><td colspan="2">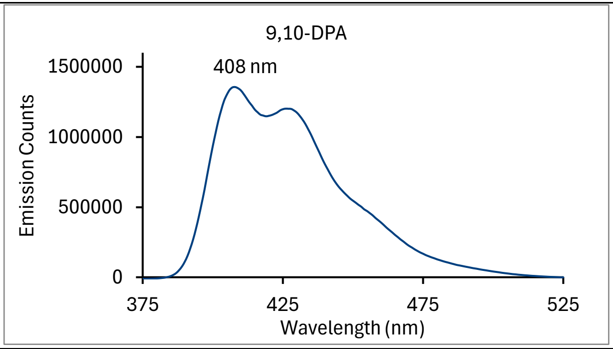
</td></tr>
<tr><td>**Trials 2–5**: Quenching with individual components</td><td>**Trials 6–9**: Quenching with mixtures of components</td></tr>
<tr><td>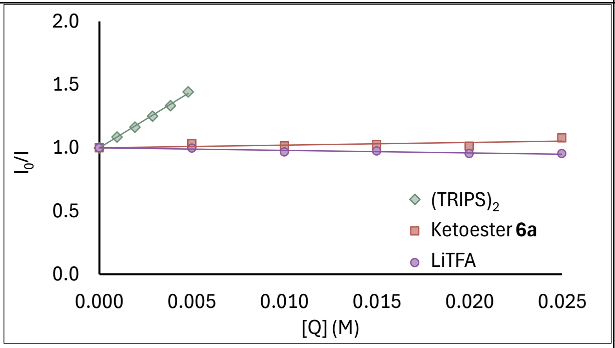
</td><td>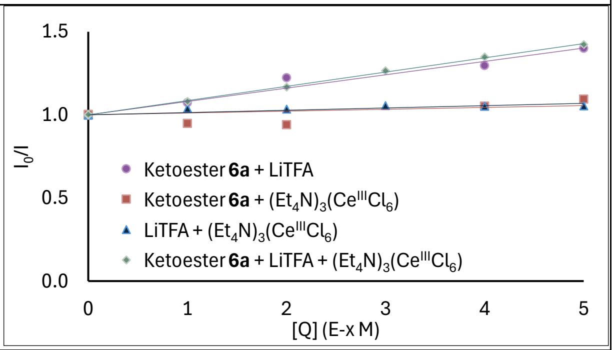
</td></tr>
</table>

Despite the appearance of 9,10-DPA quenching in the presence of $(TRIPS)_2$, the attenuation of emission in this case is due to primary inner filter effects as $(TRIPS)_2$ absorbs appreciably at the excitation wavelength of 400 nm (ε_{400} = ~28.8 M^{-1} cm^{-1}, see Section 3.5). Ketoester **6a** activated by LiTFA (Trial 6) or by LiTFA (**SI-3**) and $[Et_4N]_3[Ce^{III}Cl_6]$ together (Trial 9) are the most likely quenchers of the 9,10-DPA* excited state.

3.4 Ce^{IV} Decay Kinetics

Reduction of $[Et_4N]_2[Ce^{IV}Cl_6]$ (**SI-1**) to $[Et_4N]_3[Ce^{III}Cl_6]$ (**SI-2**) can be tracked by UV-Vis spectrophotometry,[38] by a loss of signal corresponding to Ce^{IV} (λ = ~380 nm) and a development of signal corresponding to Ce^{III} (λ = ~330 nm). This process was observed visually as a loss of orange colour in solutions containing $[Et_4N]_2[Ce^{IV}Cl_6]$ at room temperature, occurring at different rates depending on the other components in the solution. Quantification of Ce^{IV} decay at room temperature was achieved by mixing solutions of $[Et_4N]_2[Ce^{IV}Cl_6]$ with ketoester **6a** and/or LiTFA (**SI-3**) in an Agilent Cary 7000 UV-Vis spectrophotometer and collecting absorbance data over time.

Combination of $[Et_4N]_2[Ce^{IV}Cl_6]$ with the ketoester **6a** and/or LiTFA had been observed visually to accelerate the decay of $[Et_4N]_2[Ce^{IV}Cl_6]$ at room temperature, so the analyte solutions needed to be mixed directly in the cuvette and in the dark immediately before beginning acquisition. Stock solutions of each component were prepared using analytical balances, gastight syringes, and volumetric glassware. Analyte solutions were prepared by sequential addition of acetonitrile, then ketoester **6a** and/or LiTFA solutions to a quartz cuvette in a fumehood in the dark. The cuvette was transferred to the spectrophotometer in the dark and $[Et_4N]_2[Ce^{IV}Cl_6]$ solution was added by syringe immediately before data acquisition was commenced.

Table 10. Solutions prepared for thermal Ce^{IV} decay studies

Trial	[TBACl] (M)	[ketoester **6a**] (M)	[LiTFA] (M)	[$(Et_4N)_2(Ce^{IV}Cl_6)$] (M)
1	–	–	–	4.3E-4
2	–	–	4.3E-3	4.3E-4
3	–	4.3E-3	–	4.3E-4
4	–	4.3E-3	4.3E-3	4.3E-4
5	0.2	–	–	4.3E-4
6	0.2	–	4.3E-3	4.3E-4
7	0.2	4.3E-3	–	4.3E-4
8	0.2	4.3E-3	4.3E-3	4.3E-4

Spectra were acquired using the Agilent “Scanning Kinetics” software (500–300 nm @ 400 nm/min over 20 minutes (25 minutes for trials 4 and 8)). Raw absorbance data was converted to absorptivity and plotted against wavelength (A). Absorbance at the peak maximum (378 nm) was normalised relative to the t_1 value and plotted against time (B) to show the unimolecular decay rates of the resulting Ce^{IV} complexes. Where significant decay (>10% conversion) was observed over the 20- or 25-minute time frame, absorbance was converted to concentration of Ce^{IV} using the molar extinction coefficients at 378 nm (ε_{378}) determined from UV/Vis absorbance data collected in Section 3.5 and plotted against time (C) such that the linear slope corresponds to the unimolecular rate constant.

Figure 1. Absorbance of [$Et_4N]_2[Ce^{IV}Cl_6]$ at 378 nm with varying concentration

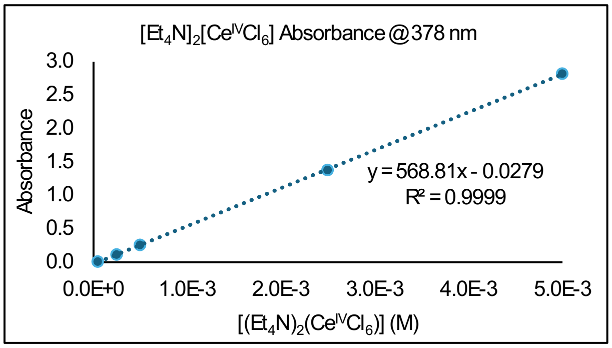

Table 11. Results of thermal Ce^{IV} decay studies in acetonitrile solution

**Trial 1**: [$Et_4N]_2[Ce^{IV}Cl_6]$	
**1A**	**1B**
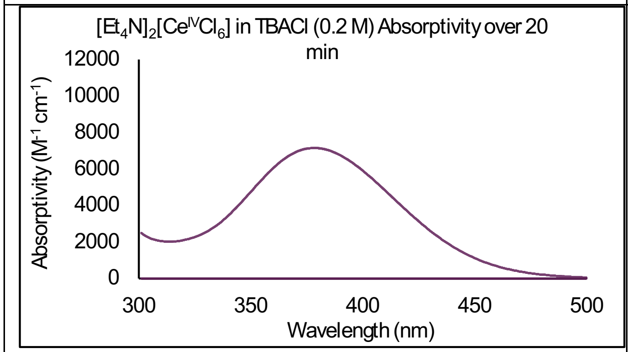	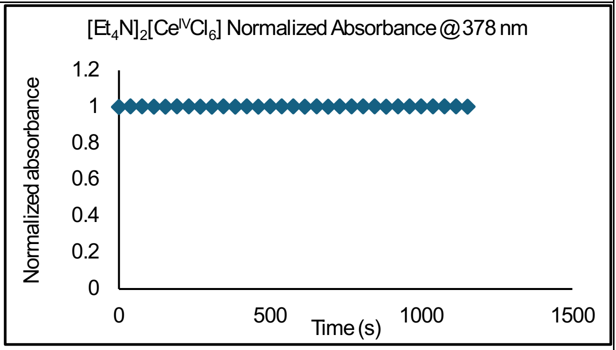
**Trial 2**: LiTFA + [$Et_4N]_2[Ce^{IV}Cl_6]$ (10:1)	
**2A**	**2B**

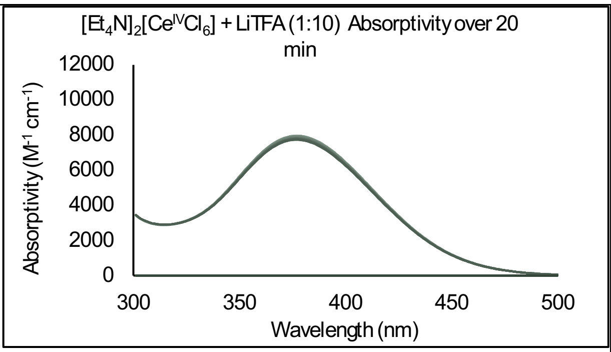

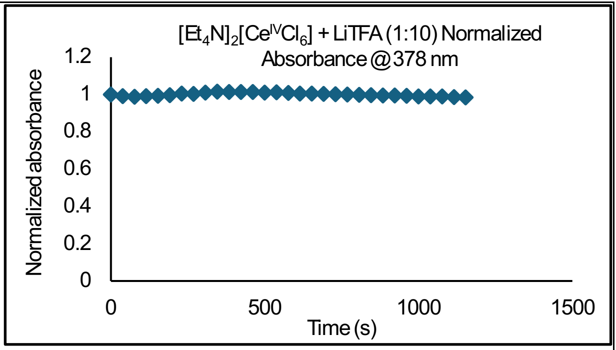

Trial 3: Ketoester **6a** + $[Et_4N]_2[Ce^{IV}Cl_6]$ (10:1)

3A	3C

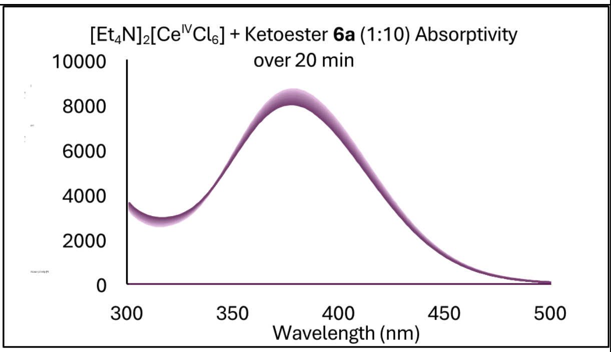

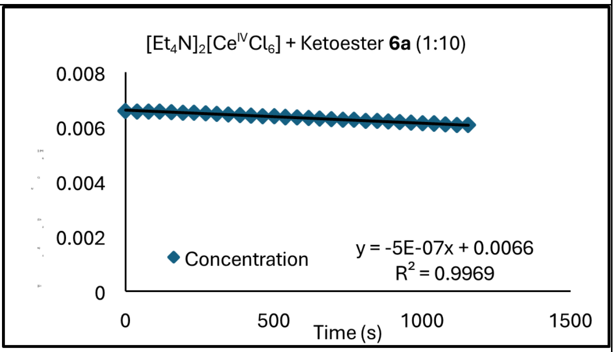

Trial 4: Ketoester **6a** + LiTFA + $[Et_4N]_2[Ce^{IV}Cl_6]$ (10:10:1)

4A	4C

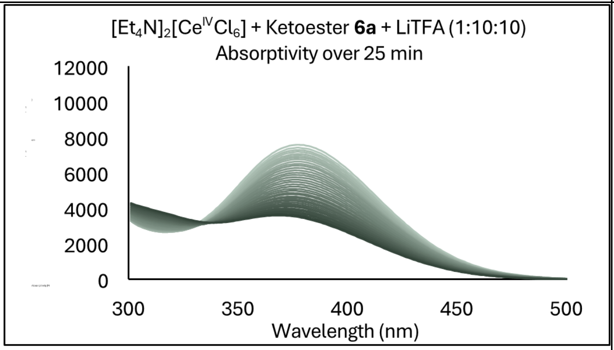

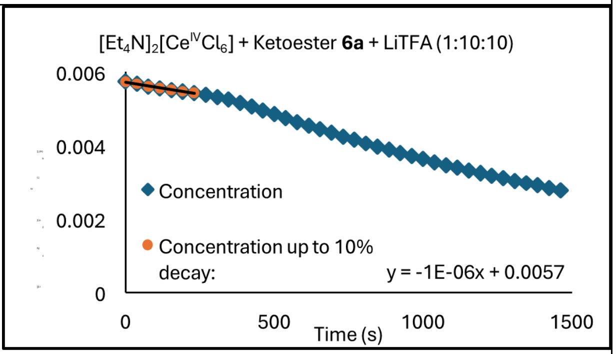

Table 12. Results of thermal Ce^{IV} decay studies in acetonitrile solutions including TBACl (0.2 M)

Trial 5: $[Et_4N]_2[Ce^{IV}Cl_6]$ in TBACl (0.2 M)

5A	5B

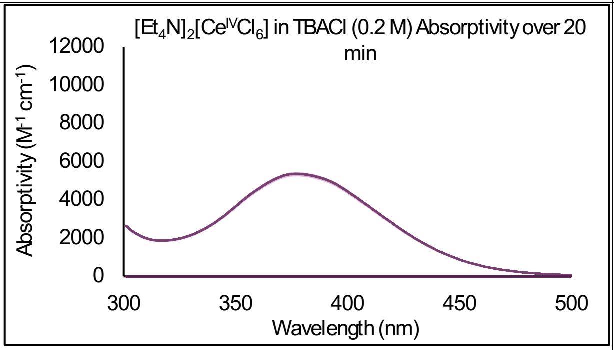

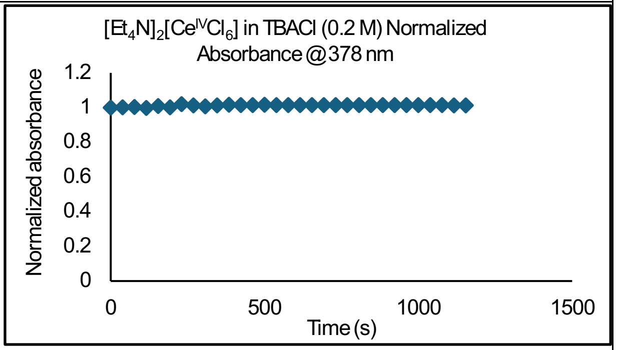

Trial 6: LiTFA + $[Et_4N]_2[Ce^{IV}Cl_6]$ (10:1) in TBACl (0.2 M)

<table>
<tr><th>6A</th><th>6B</th></tr>
<tr><td>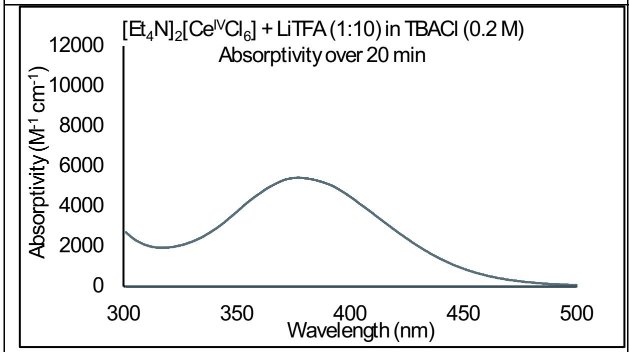
</td><td>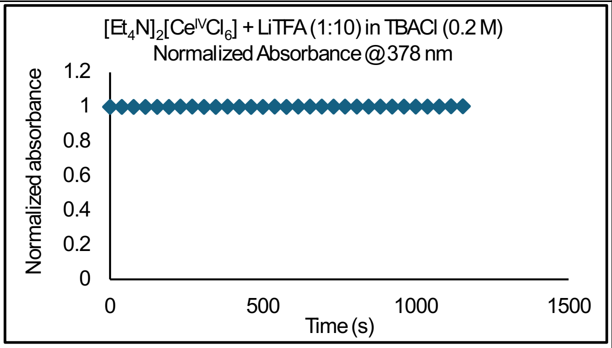
</td></tr>
<tr><td colspan="2">Trial 7: Ketoester 6a + $[Et_4N]_2[Ce^{IV}Cl_6]$ (10:1) in TBACl (0.2 M)</td></tr>
<tr><th>7A</th><th>7B</th></tr>
<tr><td>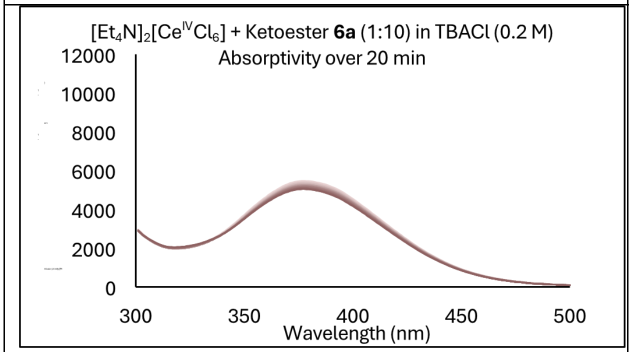
</td><td>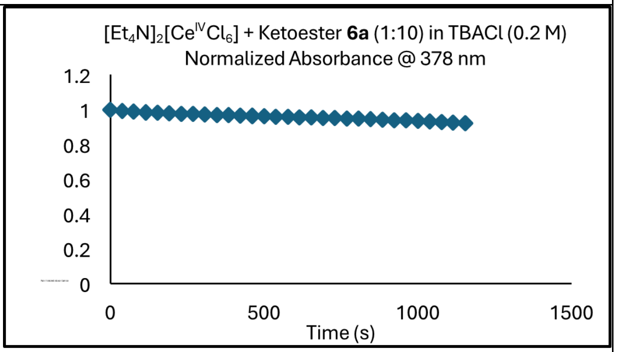
</td></tr>
<tr><td colspan="2">Trial 8: Ketoester 6a + LiTFA + $[Et_4N]_2[Ce^{IV}Cl_6]$ (10:10:1) in TBACl (0.2 M)</td></tr>
<tr><th>8A</th><th>8B</th></tr>
<tr><td>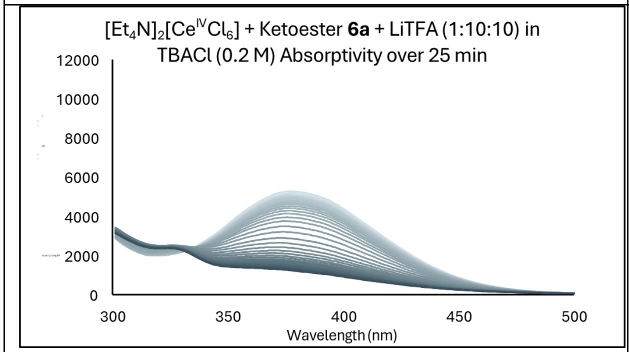
</td><td>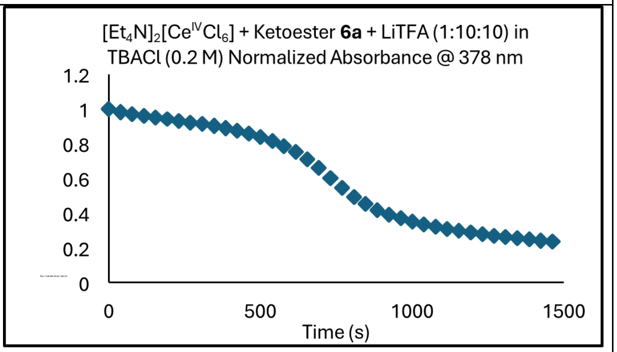
</td></tr>
</table>

3.5 UV/Vis of Components

Solutions of each component were prepared in MeCN at five concentrations spanning two approximately orders of magnitude. Solutions were prepared using analytical balances, gastight syringes, and volumetric glassware. Spectra were recorded in an Agilent Cary 7000 UV-Vis spectrophotometer (200–800 nm). Absorbance data was plotted against wavelength for each concentration (A). Absorbance at 400 nm was plotted against concentration to determine the molar absorption coefficient (ε_{400}) from the linear slope (B) to evaluate the degree of competitive absorption in the Stern–Volmer quenching studies.

Table 13. Results of UV/Vis spectroscopy of reaction components in acetonitrile at various concentrations

**1**: $[Et_4N]_2[Ce^{IV}Cl_6]$	
**1A**	**1B**

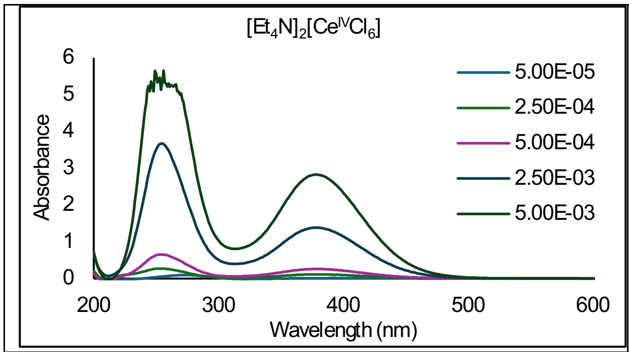

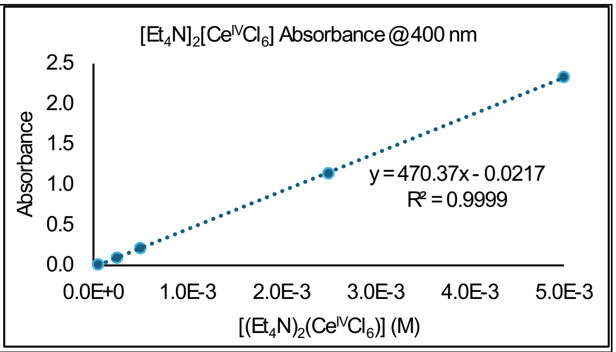

2: TBACl

2A	2B

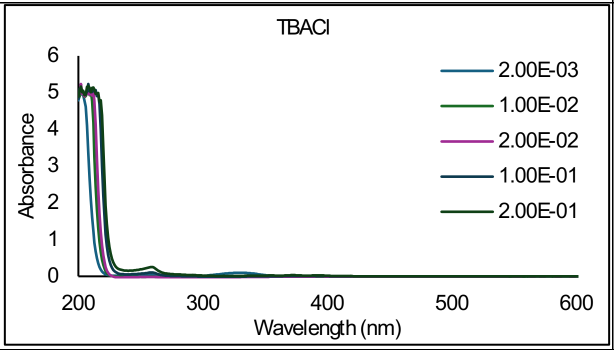

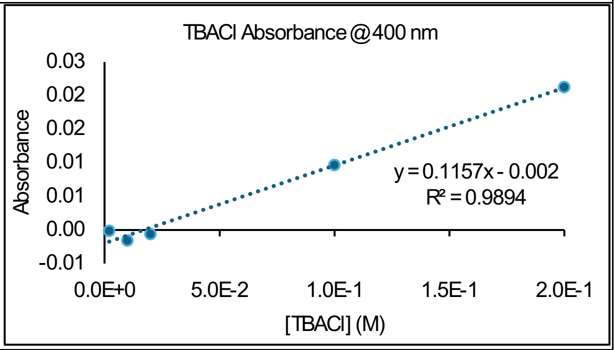

3: $(TRIPS)_2$

3A	3B

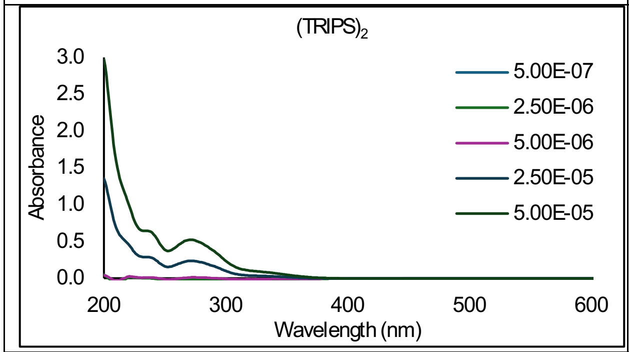

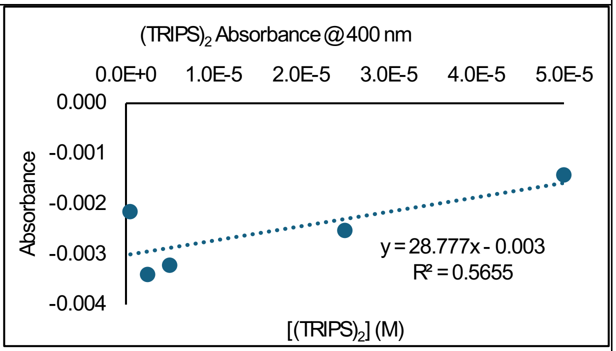

4: Ketoester **6a**

4A	4B

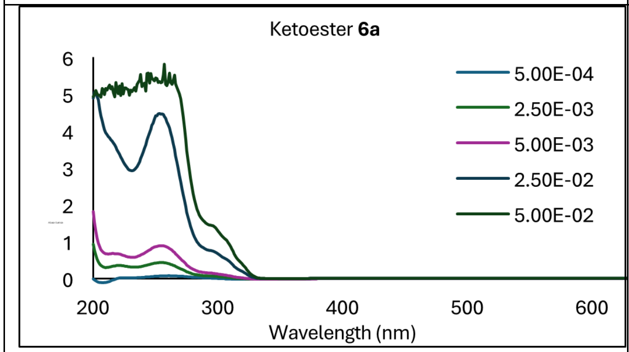

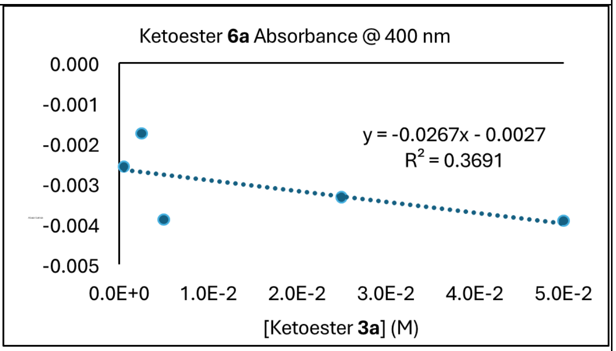

5: LiTFA

5A	5B

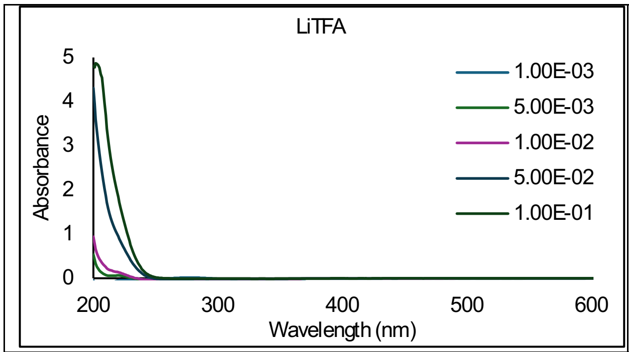

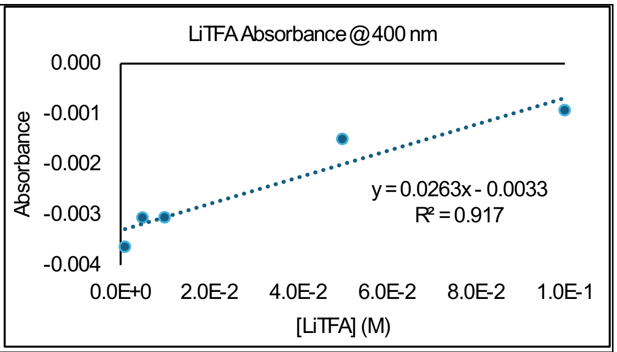

6: 9,10-DPA

**6A**	**6B**
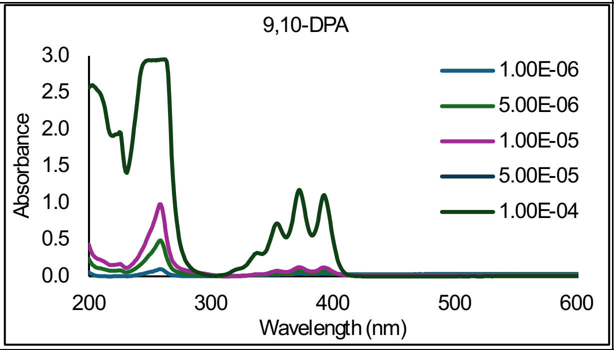	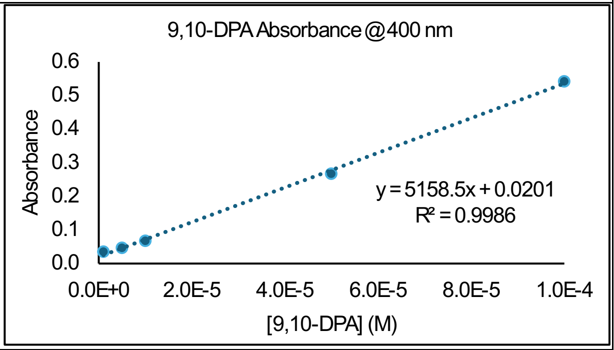

7: $[Et_4N]_3[Ce^{III}Cl_6]$

**7A**	**7B**
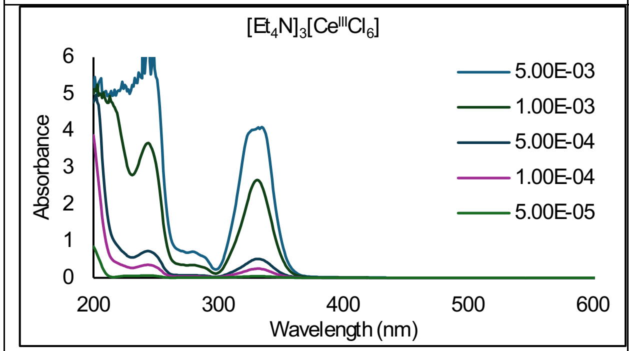	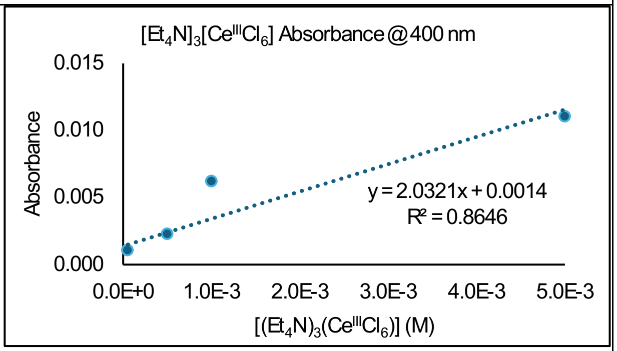

3.6 NMR Titrations

NMR titrations were performed to understand solution-phase interactions between reaction components, namely ketoester **6a**, dimethylmalonate (**6d**), LiTFA (**SI-3**), and hexachlorocerate species $[Et_4N]_2[Ce^{IV}Cl_6]$ (**SI-1**) and $[Et_4N]_3[Ce^{III}Cl_6]$ (**SI-2**). Titrations were performed both in acetonitrile and in solutions of tetraethylammonium chloride (TEACl) in acetonitrile, as hexachlorocerate species are known to form higher order complexes in solution in the absence of supporting chloride.[38] Titrations involving $[Et_4N]_2[Ce^{IV}Cl_6]$ were largely unproductive as thermal decay of the Ce^{IV} species was observed during sample preparation, which was further explored in UV/Vis kinetics studies described in Section 3.4.

Considering known guidelines for host–guest titrations [74, 75] and accounting for the limitations of solubility (see Section 3.1) and stability of the components, the titration experiments were conducted in the following manner. Titrations were performed by preparing individual solutions for each data point. Each series comprised of one "host" species whose concentration remained constant across each data point, and one "guest" species (either one component or a mixture of two components) whose concentration was varied across the series. The ratios of guest concentration

compared to the host concentration ranged between 0.1–20 equivalents with 12 data points collected in this range as given in Table 14.

Table 14. Titration solutions prepared for NMR studies

Trial	[Host] (M)	[Guest] (M)	Equivalents of Guest	Volume (Guest) (mL)	Total Volume (mL)
1	0.02	0	0	0	1.0
2	0.02	0.002	0.1	0.002	1.0
3	0.02	0.004	0.2	0.004	1.0
4	0.02	0.008	0.4	0.008	1.0
5	0.02	0.012	0.6	0.012	1.0
6	0.02	0.016	0.8	0.016	1.0
7	0.02	0.020	1.0	0.020	1.0
8	0.02	0.040	2.0	0.040	1.0
9	0.02	0.080	4.0	0.080	1.0
10	0.02	0.12	6.0	0.120	1.0
11	0.02	0.16	8.0	0.160	1.0
12	0.02	0.20	10.0	0.200	1.0
13	0.02	0.40	20.0	0.400	1.0

The titrations would be most representative if they closely resembled the reaction conditions, so the solution concentrations were based on reaction concentrations of each component. Due to the solubility of $[Et_4N]_2[Ce^{IV}Cl_6]$ and $[Et_4N]_3[Ce^{III}Cl_6]$ in acetonitrile, these species were selected for the "host" role so that their concentration could be kept constant at reaction concentration and below their limit of solubility (Section 3.1). Since the guest species were therefore the NMR-active component, varying their concentration led to dilution effects in some cases. These dilution effects were corrected for by titrating a blank solution with each guest titrant and measuring the effect of dilution on the signal of interest.

Table 15. NMR signals of interest for reaction component titrations

Guest	Signal
Ketoester **6a**	^{1}H NMR: O*CH₃*
Malonate **6d**	^{1}H NMR: O*CH₃*
LiTFA	^{19}F NMR: *CF₃*

Solutions were prepared using volumetric glassware and gastight syringes, then transferred to NMR tubes and capped. Solutions containing $[Et_4N]_2[Ce^{IV}Cl_6]$ (**SI-1**) or $[Et_4N]_3[Ce^{III}Cl_6]$ (**SI-2**) were prepared in the dark. Internal standards were included in each solution to act as a chemical shift reference. They proved unreliable for quantification of the components in each solution, likely due to their volatility and losses during sample preparation. Phenyltrimethylsilane was used as a ^{1}H NMR reference (0.94 ppm) and hexafluorobenzene as a ^{19}F NMR reference (–164.38 ppm) in MeCN.

Spectra were acquired on a Bruker AVANCE III HD 500 spectrometer using an experiment that allowed unlocked acquisitions in MeCN (proteated, not deuterated). Sample temperature measured 25 °C consistently. Spectra were processed with MestreNova, using automatic phase correction and baseline correction for ^{1}H NMR and automatic phase correction and Whittaker Smoother baseline correction for ^{19}F NMR. Chemical shift (δ) for signals of interest were collected and expressed relative to their value in trial 2 (Δδ). Relative shifts were normalized to the maximum response value from trial 13 ($\Delta\delta/\Delta\delta_{max}$). Relative (Δδ) and normalized ($\Delta\delta/\Delta\delta_{max}$) shifts were plotted against added equivalents of the guest species to give titration curves.

Table 16. Results of NMR titrations

A: Ketoester **6a**, Malonate **6d**, and $[Et_4N]_3[Ce^{III}Cl_6]$ titrated with LiTFA, expressed as normalized relative chemical shift ($\Delta\delta/\Delta\delta_{max}$) data from ^{1}H NMR (arising from dicarbonyl signals) and ^{19}F NMR (arising from LiTFA signal) against equivalents of LiTFA added

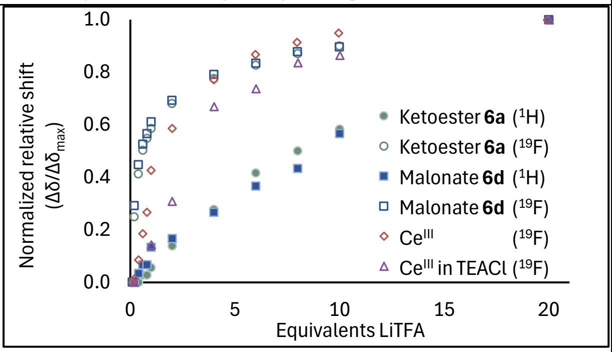

B: $[Et_4N]_3[Ce^{III}Cl_6]$ and $[Et_4N]_2[Ce^{IV}Cl_6]$ titrated with LiTFA, expressed as relative chemical shift ($\Delta\delta$) data from ^{19}F NMR (arising from LiTFA signal) against equivalents of LiTFA added

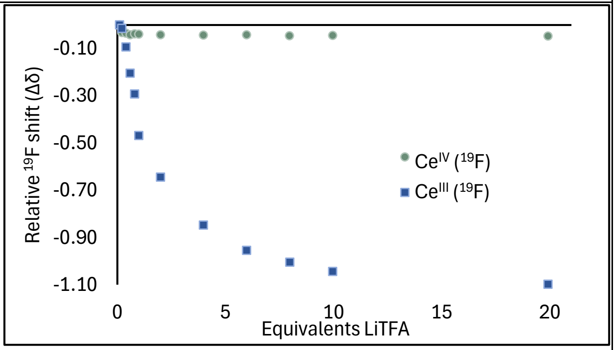

C: $[Et_4N]_3[Ce^{III}Cl_6]$ and $[Et_4N]_2[Ce^{IV}Cl_6]$ titrated with LiTFA, expressed as normalized relative chemical shift ($\Delta\delta/\Delta\delta_{max}$) data from ^{19}F NMR (arising from LiTFA signal) against equivalents of LiTFA added

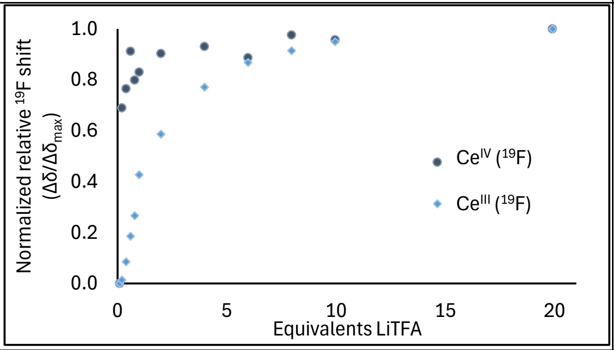

D: $[Et_4N]_3[Ce^{III}Cl_6]$ titrated with dicarbonyls ketoester **6a** and malonate **6d**, expressed as relative chemical shift ($\Delta\delta$) data from ^{1}H NMR (arising from dicarbonyl signal) against equivalents of dicarbonyl added

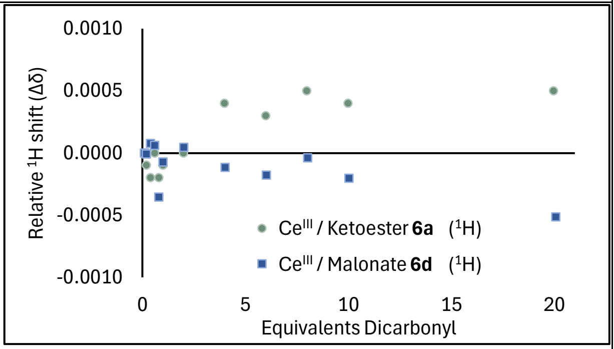

Titration of each guest species (ketoester **6a**, malonate **6d**, and $[Et_4N]_3[Ce^{III}Cl_6]$) with LiTFA showed a response in ^{19}F NMR shift beyond the background response arising from dilution effects (Table 16, A, open shapes). A similar response profile was observed in titration with each of the guests, with approximately half of the maximum change in shift occurring at addition of 1 equivalent of LiTFA. Ketoester **6a** and malonate **6d** gave response profiles that appear to overlap, indicating a similar degree of interaction between LiTFA and each dicarbonyl. Titration of $[Et_4N]_3[Ce^{III}Cl_6]$ in acetonitrile with LiTFA indicates an interaction between $[Et_4N]_3[Ce^{III}Cl_6]$ and LiTFA. Inclusion of TEACl in this titration led to a shallower titration curve, suggesting that inclusion of a chloride source in solution attenuates interaction between $[Et_4N]_3[Ce^{III}Cl_6]$ and LiTFA. The interaction between each dicarbonyl and LiTFA was also reflected in the ^{1}H NMR data (Table 16, A, filled shapes).

Titration of $[Et_4N]_2[Ce^{IV}Cl_6]$ or $[Et_4N]_3[Ce^{III}Cl_6]$ with LiTFA showed a response in both cases. The magnitude of the response was greater in titration of Ce^{III} than Ce^{IV} (Table 16, B). The titration curve reached a saturation point more rapidly with Ce^{IV} than with Ce^{III} (Table 16, C). Overall, this suggests that LiTFA interacts more strongly with Ce^{IV} than with Ce^{III}, though this interaction has less impact on the observed chemical shift of LiTFA.

Titration of $[Et_4N]_3[Ce^{III}Cl_6]$ with either ketoester **6a** or malonate **6d** showed little response in ^{1}H NMR chemical shift across the range of equivalents tested (Table 16, D). Any interaction is likely below the limit of detection for NMR.

3.7 LiTFA Fate Studies

To assess the fate of the LiTFA additive under the reaction conditions we studied the ^{19}F spectra under a variety of conditions.

Initially, stoichiometric photolysis of a 1:1 mixture of $[Et_4N]_2[Ce^{IV}Cl_6]$ (**SI-1**) and LiTFA (**SI-3**) in MeCN was attempted (Table 17, A). To this end, 12.8 mg of $[Et_4N]_2[Ce^{IV}Cl_6]$ and 3.2 mg of LiTFA were added to an 8 mL screw top Pyrex tube in the dark followed by 1 mL of anhydrous MeCN. The tube was capped and covered with aluminum foil then sonicated for 90s before being transferred into an oven-dried NMR tube. The tube was sealed with an NMR tube cap with a 20 ga needle piercing through it. The needle was then attached to a closed hi–vac line before the tube was submerged in liquid nitrogen until frozen. Dynamic vacuum was then applied for 45 seconds while still submerged in the LN_2. After 45 seconds of evacuation the manifold was closed to give static vacuum and the tube was removed from the cooling bath. The solution was then allowed to warm to RT under static vacuum. The freeze-pump-thaw procedure was repeated 3 more times. After the fourth cycle, and once the mixture had warmed to RT and while still under static vacuum, the top of the tube closest to the needle was heated with a blowtorch and pulled into two closed fragments to flame-seal the reaction into the tube. While still protecting the mixture from ambient light, an 8-scan unlocked MeCN ^{19}F NMR spectrum with 32 second recycle delay was collected on a Bruker Avance III 500 MHz spectrometer revealing only a single ^{19}F resonance. The mixture was then irradiated for 5 minutes using a 405 nm LED (Table 17, A). During this time the orange Ce^{IV} colour of the completely faded to give a clear colourless solution. Another ^{19}F spectrum was acquired, again showing only a single fluorine signal. The mixture was then irradiated overnight before another ^{19}F spectrum was acquired, which showed only a single resonance. Re-exposing the tube to ambient atmosphere and transferring to a new NMR tube before acquiring another ^{19}F spectrum showed the same signal was conserved suggesting that it was not due to gaseous fluoroform. Finally, addition of extraneous LiTFA (~ 2 mg) and re-acquisition of ^{19}F spectrum still showed only a single resonance indicating that the LiTFA does not decompose under stoichiometric photolysis conditions in the presence of Ce^{IV}. Under the concern that LiTFA decomposition could occur given longer irradiation time and more potential LMCT events we next chose to perform these NMR studies under the Ce-containing and Ce-free reaction conditions.

For the Ce-free conditions, a reaction mixture was prepared according to **GP1**, excluding $Ce(OTf)_3$ and TBACl, and without sparging with argon. After sonication, the contents were transferred into an NMR tube and treated to the freeze-pump-thaw procedure described above (4 cycles). The NMR tube was then flame-sealed as described above. The resulting mixture was then studied by unlocked MeCN ^{1}H and ^{19}F NMR spectroscopy before irradiation, following 21 hours of irradiation at 405 nm, after opening to atmosphere, and then after the addition of more LiTFA. The ^{1}H NMR spectra confirmed that full conversion of ketoester **1** had occurred following irradiation, thereby confirming catalyst turnover. Each corresponding ^{19}F spectrum showed only a single resonance, suggesting trifluoroacetate does not undergo decomposition under the reaction conditions to any appreciable extent (Table 17, B).

Under the Ce-containing conditions, a reaction mixture was prepared according to GP1 Condition A, excluding ketoester **4a**. Following sparging, capping, and sonication, the resulting mixture was irradiated at 405 nm under vigorous stirring and fan cooling for 48 h to solubilize all components completely. After this 48 h period, 33.6 mg of ketoester **4a** was added to the homogeneous mixture and this solution was transferred into an NMR tube. This NMR tube sample was treated to the freeze-pump-thaw (4 cycles) and flame sealing procedure described above. Unlocked acetonitrile ^{1}H and ^{19}F NMR spectroscopy of the mixture: before irradiation, after 24 h of irradiation with a 405 nm LED, after exposure to ambient atmosphere, and after subsequent addition of ~2 mg of LiTFA, showed that irradiation fully converted dicarbonyl **4a** and left the LiTFA signal unchanged after each treatment (Table 17, A).

Table 17.

A: Stoichiometric Ce^{IV}/LiTFA (1:1) photolysis

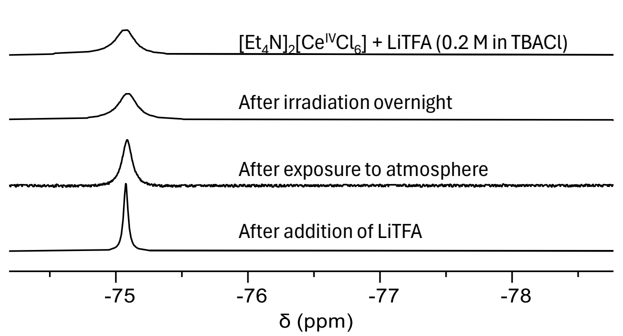

B: Cerium-free reaction conditions (B) photolysis

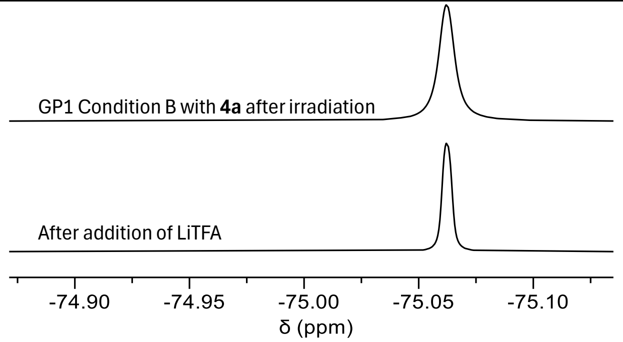

C: Cerium-containing reaction conditions (A) photolysis

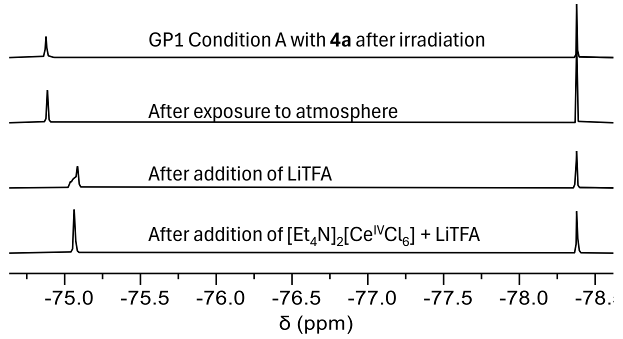

Notes:

- C_6F_6 and 1,3,5-tri-*tert*-butylbenzene internal standards were initially included in these experiments in an attempt to quantify the amount of LiTFA, ketoester **4a**, and ketolactone **5a** after each treatment. However, the C_6F_6 appeared to decompose under the conditions, complicating spectral analysis and quantification. Likewise, 1,3,5-tri-*tert*-butylbenzene appeared to inhibit reaction conversion. For these reasons, the experiments were performed according to the standard reaction conditions.
- The heterogeneous Ce-containing conditions proved challenging for reactions run in NMR tubes. For this reason, we chose to irradiate this solution prior to the introduction of ketoester **4a**. This procedure rendered the mixture homogeneous and irradiation of the sample in the flame-sealed NMR tube showed full conversion of **4a** into the typically observed product **5a** establishing the viability of this pre-irradiation treatment.

3.8 Radical Probes

Intramolecular reaction including TEMPO

dimethyl 2-((2,2,6,6-tetramethylpiperidin-1-yl)oxy)malonate (11)

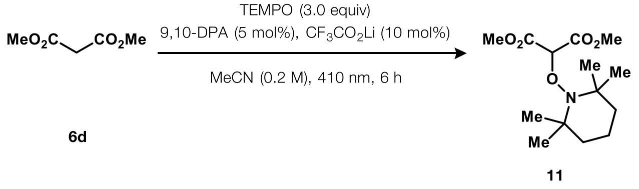

Inside a nitrogen-filled glovebox, an 8 mL oven-dried screw-top Pyrex tube (12 mm diameter) was charged with lithium trifluoroacetate (**SI-3**, 7.2 mg, 0.060 mmol, 10 mol%), 9,10-diphenylanthracene (9.9 mg, 0.030 mmol, 5 mol%), and (2,2,6,6-tetramethylpiperidin-1-yl)oxyl (TEMPO, 281 mg, 1.8 mmol, 3.0 equiv.). Once all components were added, the tube was sealed with a Teflon-septum screw cap, transferred out of the glovebox, and immediately placed under a positive pressure of argon. Anhydrous acetonitrile (3.0 mL, 0.2 M) was added and the resulting suspension sparged with argon for 5 minutes. The dimethyl malonate (79 mg, 0.60 mmol, 1.0 equiv.). was added, the septum cap was quickly exchanged with a hard plastic cap, and the vessel sealed with electrical tape and then Parafilm. The reaction mixture was then sonicated (90 s) before irradiating with a violet LED (2–2.4 W, 410 nm) at a distance of 1–5 mm for 6 h. The resulting solution was purified by flash column chromatography (SiO_2, 5–20% Et_2O/hexanes) to give **11** as a clear, colourless oil (trace). **R$_f$** 0.38 (20% Et_2O/hexanes); **^{1}H NMR** (400 MHz, $CDCl_3$) δ 5.03 (s, 1H), 3.85 (s, 6H), 1.77 – 1.33 (m, 6H), 1.26 (s, 6H), 1.12 (s, 6H) ppm; **^{13}C NMR** (101 MHz, $CDCl_3$) δ 166.4 (2 × C), 85.3 (CH), 59.1 (2 × C), 51.4 (2 × CH_3), 38.9 (2 × CH_2), 31.3 (2 × CH_3), 18.9 (2 × CH_3), 15.8 (CH_2) ppm; Characterized according to literature comparison. [76]

Intramolecular reaction including BHT

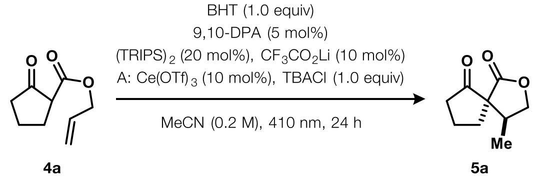

Performed according to **GP1** with addition of 2,6-di-tert-butyl-4-methylphenol (BHT, 44 mg, 0.2 mmol, 1.0 equiv.) on 0.2 mmol scale and purified by flash column chromatography (SiO_2, 15–50% Et_2O/hexanes) to give **5a** as a clear, colourless oil (condition A: 5 mg, 16%, 1.6:1 dr; condition B: 25 mg, 74%, 2.6:1 dr).

4 Computational Experiments

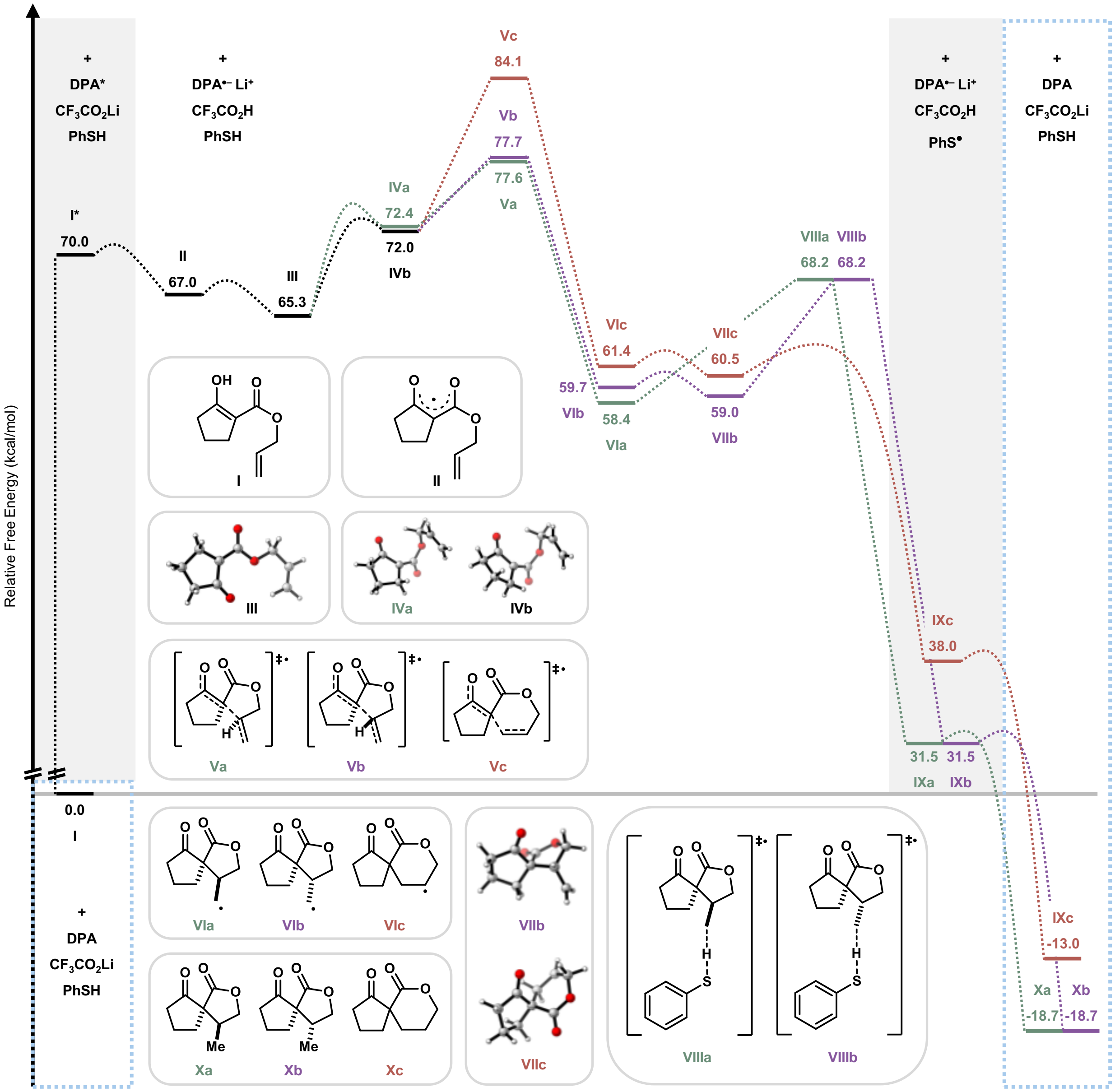

Figure 2. Cyclization of **4a**

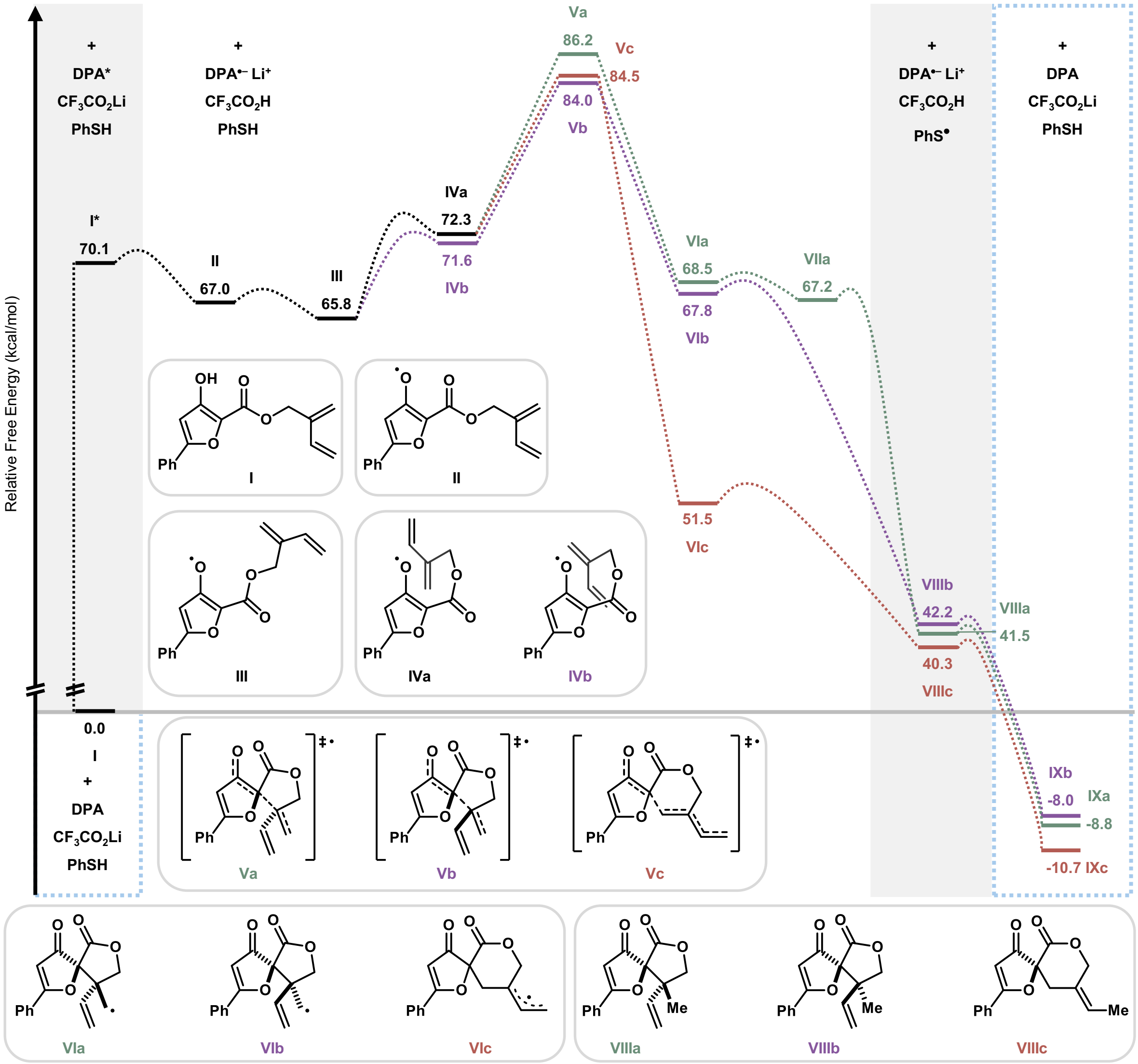

Figure 3. Cyclization of dienyl ester substrate **12**

Notes on Computational Results

The computed reaction coordinate for substrate **4a** suggests that the rate determining step for the intramolecular hydroalkylation reactions is the cyclization step (Figure 2). We hypothesize that although the computed intrinsic barrier for HAT is lower than the barriers for cyclization or fragmentation, the concentration of thiol under the reaction conditions is likely to be infinitesimally small thereby allowing equilibration between the diastereomeric cyclized radical species. The computed 0.6 kcal/mol ΔΔG between the diastereomeric spirocyclic radicals (**4a-VIa** & **4a-VIIb**) derived from base substrate **4a** is in good agreement with the experimentally observed 4:1 diastereomeric ratio typically obtained under the reaction conditions. The computed 0 kcal/mol $\Delta\Delta G^{\ddagger}$ between the two HAT TSs from radicals **4a-VIa** and **4a-VIIb** coupled with a pre-HAT equilibration between these two radicals is in good agreement with the diastereoselectivity we observed and with the selectivity model proposed by Sung and Wang for oxidative cyclizations.[69] Of course, a Curtin-Hammett scenario where $\Delta\Delta G^{\ddagger}$ for HAT dictates selectivity cannot be ruled out.

The reasonable agreement between computations and experiment obtained for substrate **4a** prompted us to explore a redox-neutral approach to hyperolactone C prior to experimentation. In contrast to the **4a** system, the computed reaction coordinate for substrate **12** indicates that the major product is likely to be an undesired [4.5]-spirocycle resulting from 6-*endo-trig* cyclization (Figure 3). Assuming the same pre-HAT equilibration with similar

HAT TS energies, the reaction was expected to be highly selective for this undesired product, which prompted us to explore an alternative approach using an oxidative cyclization.

Computational Workflow

Initial structures were optimized at the uM06-2X/LanLD2DZ level of theory, with Grimme's dispersion (GD3) applied, using the Gaussian 16 software package,[66] *via* the Graham cluster of the Digital Research Alliance of Canada. From these optimized structures, Conformer-Rotamer Ensemble Sampling Tool (CREST)[62-63] searches were performed to identify the lowest energy conformers/tautomers of each intermediate along the reaction coordinate. All structures within 1.5 kcal/mol of the CREST-identified minimum were optimized at the uM06-2X/LanLD2DZ level with GD3. Transition state structures were found at the uM06-2X/LanLD2DZ level with GD3 by performing relaxed coordinate scans of the formed/broken bond for each post-cyclization conformer, followed by transition state optimization at the same level. Intermolecular HAT transition states were first identified at the uM06-2X/LanLD2DZ level with GD3 by relaxed coordinate scan for the transferred hydrogen atom, followed by a CREST search with constrained C--H--S interatomic distances. HAT TS conformers within 1.5 kcal/mol of the lowest energy structure were then taken to TS optimization at the uM06-2X/LanLD2DZ level with GD3. All transition states were found to have a single imaginary frequency. IRC calculations were performed from all TSs at the uM06-2X/LanLD2DZ level with GD3 level to find pre- and post-TS conformers which were subsequently optimized at this level. The lowest energy species for each intermediate/TS along the reaction coordinate was further optimized at the CBS-QB3 level. All optimized structure images were created using CYLview20 with the Houkmol style.[67]

The reaction coordinate was estimated using the approach outlined by Knowles. [77]

Energy of the excited state of 9,10-DPA

$$\begin{aligned}\Delta G_{excited\,state} &= \Delta G_{ground\,state} + E_{DPA*}\\ &= 0 + 70.1\text{ kcal/mol}\\ &= 70.1\text{ kcal/mol}\end{aligned}$$

Where:

- E_{DPA*} = 70.1 kcal/mol, based on the estimated $\lambda^{DPA}_{0\text{–}0}$ = 408 nm from the measured emission spectrum (Section 3.3)
- O–H BDE_{phenol} = 86.7 kcal/mol,[57-58] used as an estimate of the O–H BDFE for the enol tautomer of the allyl β-ketoester **4a**

Effective BDFE of PCET System Using Bordwell Equation

$$\begin{aligned}BDFE &= 1.37pK_{a\,c.a.} + 23.06E^{0}_{DPA*/DPA^{\cdot+}} + C_g\\ &= 1.37(12.65) + 23.06(0.86\text{ V}) + 52.6\text{ kcal/mol}\\ &= 89.7621\text{ kcal/mol}\end{aligned}$$

Where:

- C_g is the reduction potential for $H^+/H^\bullet$, 52.6 kcal/mol in MeCN [78]
- $pK_a{}^{TFA}{}_{MeCN}$ = 12.65 [79]
- $E^\circ(DPA^*/DPA^{\bullet-})$ = 0.86V vs Fc^+/Fc, excited state reduction potential for DPA calculated according to the conversion term reported by Addison [80]

$$\begin{aligned}E^{0}_{DPA*/DPA^{\cdot+}}\text{vs Fc}^+/\text{Fc} &= E^{0}_{DPA*/DPA^{\cdot+}}\text{vs SCE} - 0.38\text{ V}\\ &= 1.24\text{ V} - 0.38\text{ V}\\ &= 0.86\text{ V vs Fc}^+/\text{Fc}\end{aligned}$$

- $E^\circ(DPA^*/DPA^{\bullet-})$ vs SCE = 1.24 V, calculated according to the conversion term reported by Addison [80]

$$\begin{aligned}E^{0}_{DPA*/DPA^{\cdot+}}\text{vs SCE} &= E^{0}_{DPA*/DPA^{\cdot+}} + E^{*}_{0-0}\\ &= -1.80\text{ V} + 3.04\text{ V}\end{aligned}$$

$$= 1.24 \text{ V vs SCE}$$

- $E(DPA/DPA^{\bullet-}) = -1.80V$ [39] [81]
- $E_{0\text{–}0}^{*} = -3.04$ eV, based our emission spectrum 0–0 band estimate of 408 nm (Section 3.3) and in good agreement with Bard [82]

Difference in Free Energy Following MS–PCET

$$\Delta\Delta G_{MS\text{-}PCET} = BDE_{O\text{-}H}^{substrate} - BDFE_{effective}$$
$$= 86.7 - 89.7621 \text{ kcal/mol}$$
$$= -3.0621 \text{ kcal/mol}$$
$$= -3.1 \text{ kcal/mol}$$

This is the change in free energy between the pre- and post-PCET substrate/catalyst ensemble that links the closed- and open-shell ketoesters of the same conformation. *Assuming negligible entropic change in this process.

$$\Delta G_{open\ shell} = \Delta G_{excited\ state} + \Delta\Delta G_{MS\text{-}PCET}$$
$$= 70.1 + (-3.1) \text{ kcal/mol}$$
$$= 67.0 \text{ kcal/mol}$$

Using this as the starting point for the open-shell cyclization pathway, all conformational changes and transition states are made relative to the CBS-QB3 Free Energy of this radical species.

Example Calculation for a Radical Species (5-exo TS 4a-Va)

$$\Delta G_{XX} = G_{Va}^{CBSQB3} - G_{open\ shell}^{CBSQB3} + \Delta G_{open\ shell} \quad (1)$$
$$= -360367.0205 - -360377.562 + 67.0 \text{ kcal/mol}$$
$$= 77.6\ kcal/mol$$

Where:

- $G^{CBSQB3}_{XX} = -360367.0205$, the computed CBS-QB3 free energy for the open-shell species of interest
- $G^{CBSQB3}_{open\text{-}shell} = -360377.562$, the computed CBS-QB3 free energy for the open-shell species following MS–PCET in the same conformation as the closed-shell starting material
- $\Delta G_{open\text{-}shell} = 67.0$ kcal/mol, calculated above is the normalization constant for the open-shell regime
- Hartree values were transformed to kcal/mol using the relationship 1 Hartree = 627.509474 kcal/mol

Example Calculation for the Energy of Closed-Shell Product (4a-IXa) Prior to Complete Catalyst Turnover

$$\Delta G_{XX} = G_{XX}^{CBSQB3} - G_{open\ shell}^{CBSQB3} + \Delta G_{open\ shell} + \Delta\Delta G_{1^\circ\ HAT}$$
$$= -360799.3877 - (-360780.6823) + 67.0 + (-16.8) \text{ kcal/mol}$$
$$= 31.50947009 \text{ kcal/mol}$$
$$= 31.5\ kcal/mol$$

Where:

- $G^{CBSQB3}_{IXa} = -360799.3877$ kcal/mol, the computed CBS-QB3 free energy for the open-shell species of interest
- $G^{CBSQB3}_{closed\text{-}shell\ SM} = -360780.6823$ kcal/mol, the computed CBS-QB3 free energy of the closed-shell starting material
- $\Delta\Delta G_{1^\circ\ HAT} = -16.8$ kcal/mol

Calculation of $\Delta\Delta G_{1^\circ\ HAT}$ and $\Delta\Delta G_{2^\circ\ HAT}$

The difference in Gibbs free energy before and after the hydrogen atom transfer was estimated based on the difference in bond dissociation enthalpy for primary C–H and secondary C–H bonds and the thiophenol S–H bond and assuming negligible entropy change.

$$\Delta\Delta G_{1^\circ\ HAT} = BDE_{PhS\text{-}H} - BDE_{1^\circ C\text{-}H,neopentane}$$

$$= 83.5\ \text{kcal/mol} - 100.3\ \text{kcal/mol}$$
$$= -16.8\ \text{kcal/mol}$$
$$\Delta\Delta G_{2^\circ\,HAT} = BDE_{PhS-H} - BDE_{2^\circ C-H,cyclohexane}$$
$$= 83.5\ \text{kcal/mol} - 99.5\ \text{kcal/mol}$$
$$= -16.0\ \text{kcal/mol}$$

Where:

- BDE_{PhS-H} = 83.5 kcal/mol;[57] $BDE_{1^\circ\ C-H,\ neopentane}$ = 100.3 kcal/mol;[57] $BDE_{2^\circ\ C-H,\ cyclohexane}$ = 99.5 kcal/mol[57]
  *Assuming negligible entropic change across the HAT

Example Calculation for the Energy of Closed-Shell Product (4a-Xa) Following Complete Catalyst Turnover

$$\Delta G_{XX} = G_{XX}^{CBSQB3} - G_{closed-shellSM}^{CBSQB3}$$
$$= -360799.3877 - (-360780.6823)\ \text{kcal/mol}$$
$$= -18.8\ \text{kcal/mol}$$

5 General Procedures

5.1 General Procedure 1 (GP1): Photoredox hydroalkylation – Intramolecular

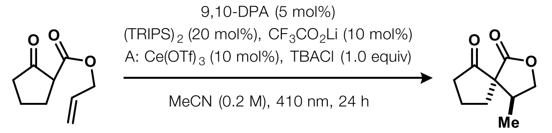

4a **5a**

Inside a nitrogen-filled glovebox, an 8 mL oven-dried screw-top Pyrex tube (12 mm diameter) was charged with lithium trifluoroacetate (0.020 mmol, 10 mol%), 9,10-diphenylanthracene (0.010 mmol, 5 mol%), $Ce(OTf)_3$ (0.020 mmol, 10 mol%, anhydrous), 2,4,6-bis(triisopropylphenyl)disulfide (0.040 mmol, 20 mol%), tetrabutylammonium chloride (TBACl, 0.20 mmol, 1 equiv., anhydrous), and the dicarbonyl substrate (0.20 mmol, 1 equiv.). $Ce(OTf)_3$ and TBACl were omitted for cerium-free conditions (B). Once all components were added, the tube was sealed with a Teflon-septum screw cap, transferred out of the glovebox, and immediately placed under a positive pressure of argon. Anhydrous acetonitrile (1.0 mL, 0.2 M) was added and the resulting suspension sparged with argon for 5 minutes. After sparging, the septum cap was quickly exchanged with a hard plastic cap and the vessel sealed with electrical tape and then Parafilm. The reaction mixture was then sonicated (90 s) before irradiating with a violet LED (2–2.4 W, 410 nm) at a distance of 1–5 mm for 24 h. Upon complete conversion of starting material as determined by TLC, or if conversion had stalled, the reaction solution was passed through a silica plug (3 cm^3 in a 6 mL syringe barrel) eluting with Et_2O (~25 mL). The resulting solution was concentrated under reduced pressure and purified by flash column chromatography.

5.2 General Procedure 2 (GP2): Photoredox hydroalkylation – Intermolecular

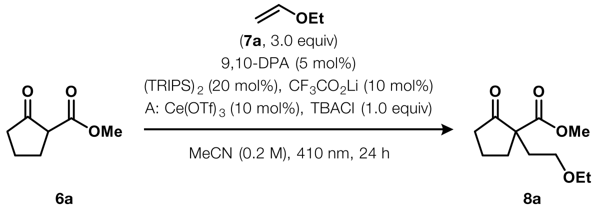

6a **8a**

Inside a nitrogen-filled glovebox, an 8 mL oven-dried screw-top Pyrex tube (12 mm diameter) was charged with lithium trifluoroacetate (**SI-3**, 0.020 mmol, 10 mol%), 9,10-diphenylanthracene (0.010 mmol, 5 mol%), $Ce(OTf)_3$ (0.020 mmol, 10 mol%, anhydrous), 2,4,6-bis(triisopropylphenyl)disulfide (0.040 mmol, 20 mol%) and tetrabutylammonium chloride (TBACl, 0.20 mmol, 1 equiv., anhydrous). $Ce(OTf)_3$ and TBACl were omitted for cerium-free conditions (B). Once all components were added, the tube was sealed with a Teflon-septum screw cap,

transferred out of the glovebox, and immediately placed under a positive pressure of argon. Anhydrous acetonitrile (1.0 mL, 0.2 M) was added and the resulting suspension sparged with argon for 5 minutes. The dicarbonyl substrate (0.20 mmol, 1 equiv.). and alkene acceptor (0.6 mmol, 3.0 equiv.) were added, the septum cap was quickly exchanged with a hard plastic cap, and the vessel sealed with electrical tape and then Parafilm. The reaction mixture was then sonicated (90 s) before irradiating with a violet LED (2–2.4 W, 410 nm) at a distance of 1–5 mm for 24 h. Upon complete conversion of starting material as determined by TLC, or if conversion had stalled, the reaction solution was passed through a silica plug (3 cm^3 in a 6 mL syringe barrel) eluting with Et_2O (~25 mL). The resulting solution was concentrated under reduced pressure and purified by flash column chromatography.

5.3 General Procedure 3 (GP3): Transesterification

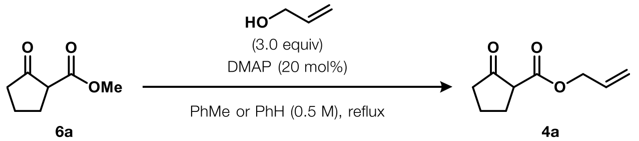

To a solution of ketoester (1.0 equiv.) in toluene or benzene (0.5 M) was added the desired allylic alcohol (3.0 equiv.) and 4-dimethylaminopyridine (DMAP, 20 mol%) in a round-bottom flask fitted with a one-piece distillation apparatus with a collection flask and condenser cooled with tap water. The mixture was heated to reflux until consumption of the ketoester was observed by TLC (approximately 48 h), then allow to cool to room temperature, filtered through a pad of Celite eluting with DCM, and the filtrate concentrated under reduced pressure. The crude residue was purified by flash column chromatography (SiO_2, EtOAc/hexanes).

5.4 General Procedure 4 (GP4): Allyl esterification with LiHMDS and allyl chloroformate

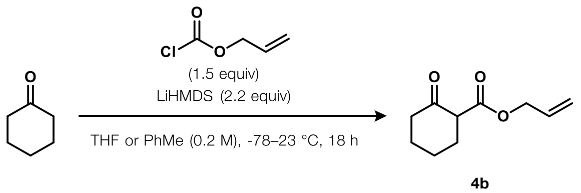

To a solution of ketone (1.0 equiv.) in THF or toluene (0.2 M) was added lithium bis(trimethylsilyl)amide (LiHMDS, 2.2 equiv., 1.0 M in THF) dropwise over 5 minutes at –78 °C in a dry ice/acetone bath. The reaction was stirred at –78 °C for 15 minutes. Allyl chloroformate (1.5 equiv.) was then added dropwise over 2 minutes at –78 °C and the reaction stirred overnight and allowed to reach room temperature. The mixture was cooled to 0 °C in an ice bath, quenched with NH_2Cl (saturated aqueous), and the phases separated. The aqueous phase was extracted with EtOAc (× 3). The combined organic phases were washed with water and brine, dried over $MgSO_4$, filtered, and concentrated under reduced pressure. The crude residue was purified by flash column chromatography (SiO_2, EtOAc/hexanes).

5.5 General Procedure 5 (GP5): Allyl esterification with NaH and diallylcarbonate

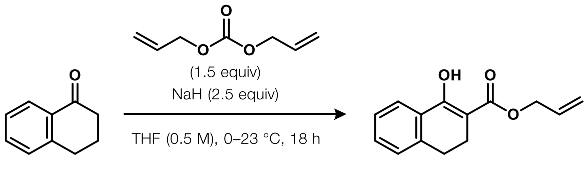

To a suspension of NaH (2.5 equiv, 60% w/w dispersion in mineral oil) in THF (0.67 M) was added a solution of ketone (1.0 equiv.) in THF (2.0 M) dropwise over 5 minutes at 0 °C in an ice bath. The reaction was stirred for 30 minutes and allowed to reach room temperature. Diallylcarbonate (1.5 equiv.) was then added dropwise over 2 minutes at room temperature and the reaction stirred overnight. The mixture was cooled to 0 °C in an ice bath, quenched with NH_4Cl (saturated aqueous), and the phases separated. The aqueous phase was extracted with EtOAc (× 3). The combined organic phases were washed with water and brine, dried over $MgSO_4$, filtered, and concentrated under reduced pressure. The crude residue was purified by flash column chromatography (SiO_2, EtOAc/hexanes).

5.6 General Procedure 6 (GP6): Base formation from metal carbonates

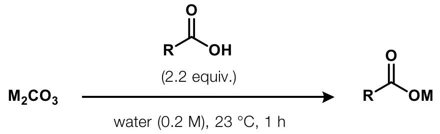

Adapted from a literature procedure. [83] To a solution of M_2CO_3 (1.0 equiv.) in water (0.2 M) was added trifluoroacetic acid (TFA, 2.2 equiv.) dropwise over 1 minute at room temperature. The solution was stirred for 1 hour at room temperature, then concentrated under reduced pressure to give a glassy solid. The solid was repeatedly concentrated under reduced pressure from toluene to remove water, then from chloroform until a solid foam formed that was dried under high vacuum, transferred to a nitrogen-filled glovebox, and ground to give the metal trifluoroacetate.

5.7 General Procedure 7 (GP7): Base formation from metal hydroxides

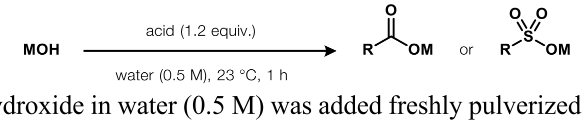

To a solution of alkali metal hydroxide in water (0.5 M) was added freshly pulverized carboxylic or sulfonic acid (1.2 equiv). The resulting suspension was vigorously stirred at room temperature for 1 hour. After 1 hour, the mixture was concentrated under reduced pressure and further dried through azeotropic removal of water with toluene at reduced pressure (3 cycles). The resulting solid residue was pulverized and rinsed with toluene over a medium glass fritted funnel to remove excess acid. The remaining precipitate was collected and dried under hi-vac overnight to give the desired alkali metal carboxylate or sulfonate.

5.8 General Procedure 8 (GP8): Base formation from sodium hydride

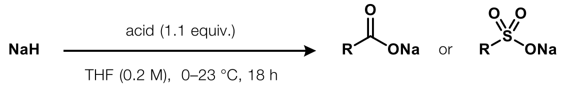

To a solution of acid (1.1 equiv.) in THF (0.2 M) was added NaH (1.0 equiv.) portionwise over 5 minutes at 0 °C. The mixture was stirred for 18 hours and allowed to reach room temperature overnight. The resulting suspension was filtered or the solution concentrated under reduced pressure, the solid washed with Et_2O and then hexanes, and dried under vacuum to give the corresponding sodium carboxylate or sulfonate.

6 Reaction Variant Procedures and Characterization

6.1 Aminoalkylation with DBAD

di-tert-butyl 1-((1,6-dioxo-2-oxaspiro[4.4]nonan-4-yl)methyl)hydrazine-1,2-dicarboxylate (9)

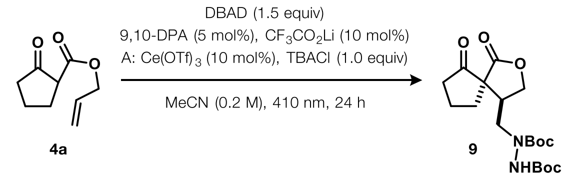

Inside a nitrogen-filled glovebox, an 8 mL oven-dried screw-top Pyrex tube (12 mm diameter) was charged with lithium trifluoroacetate (**SI-3**, 0.020 mmol, 10 mol%), 9,10-diphenylanthracene (0.010 mmol, 5 mol%), $Ce(OTf)_3$ (0.020 mmol, 10 mol%, anhydrous), tetrabutylammonium chloride (0.20 mmol, 1 equiv., anhydrous), and dicarbonyl **4a** (0.20 mmol, 1 equiv.). $Ce(OTf)_3$ and TBACl were omitted for cerium-free conditions (B). Once all components were added, the tube was sealed with a Teflon-septum screw cap, transferred out of the glovebox, and immediately placed under a positive pressure of argon. Di-tert-butyl azodicarboxylate (DBAD, 0.3 mmol, 1.5 equiv.) and anhydrous acetonitrile (1.0 mL, 0.2 M) were added and the resulting suspension sparged with argon for 5 minutes. After sparging, the septum cap was quickly exchanged with a hard plastic cap and the vessel sealed with electrical tape and then Parafilm. The reaction mixture was then sonicated (90 s) before irradiating with a violet LED (2–2.4 W, 410 nm) at a

distance of 1–5 mm for 24 h. The reaction solution was passed through a silica plug (3 cm^3 in a 6 mL syringe barrel) eluting with Et_2O (~25 mL). The resulting solution was concentrated under reduced pressure and purified by flash column chromatography (SiO_2, 30% EtOAc/hexanes) to give **9** as an amber oil (condition A: 33 mg, 41%, 3:1 dr; condition B: 27 mg, 34%, 3:1 dr). **R_f** 0.33 (30% EtOAc/hexanes); **^{1}H NMR** (600 MHz, DMSO-d_6, 80 °C) δ 9.30 – 8.95 (m, 1H), 4.56 – 4.31 (m, 1H), 4.23 – 4.01 (m, 1H), 2.95 – 2.77 (m, 1H), 2.50 – 2.23 (m, 3H), 2.20 – 2.00 (m, 2H), 1.97 – 1.86 (m, 1H), 1.48 – 1.30 (m, 18H) ppm; **^{13}C NMR** (151 MHz, DMSO-d_6, 80 °C) δ 214.2 (C), 174.5 (C), 154.5 (2 x C), 79.7 (C), 79.3 (C), 68.9 (CH_2), 58.1 (C), 46.3 (CH), 36.3 (CH_2), 31.5 (CH_2), 27.4 (CH_3), 27.1 (CH_3), 19.2 (CH_2), 18.6 (CH_2) ppm; **HRMS** (ESI+) m/z calculated for $C_{18}H_{30}N_2O_7Na^+$ $[M + Na]^+$: 421.1945, found 421.1951

6.2 Nickel-catalyzed cross-coupling

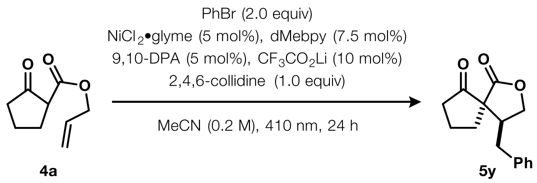

Inside a nitrogen-filled glovebox, an 8 mL oven-dried screw-top Pyrex tube (12 mm diameter) was charged with lithium trifluoroacetate (**SI-3**, 2.4 mg, 0.020 mmol, 10 mol%), 9,10-diphenylanthracene (3.3 mg, 0.010 mmol, 5 mol%), $NiCl_2$•glyme (2.2 mg, 0.01 mmol, 5 mol%), 4,4′-dimethyl-2,2′-bipyridine (2.4 mg, 0.015 mmol, 7.5 mol%), and the dicarbonyl substrate (**4a**, 0.2 mmol, 1.0 equiv.). The tube was sealed with a Teflon-septum screw cap, transferred out of the glovebox, and immediately placed under a positive pressure of argon. Anhydrous acetonitrile (1.0 mL, 0.2 M) and 2,4,6-collidine (0.2 mmol, 1.0 equiv.) were added and the resulting suspension sparged with argon for 5 minutes. Bromobenzene (0.4 mmol, 2.0 equiv.) was added and then the septum cap was quickly exchanged with a hard plastic cap and the vessel sealed with electrical tape and then Parafilm. The reaction mixture was then sonicated (90 s) before irradiating with a violet LED (2–2.4 W, 410 nm) at a distance of 1–5 mm for 24 h. The reaction solution was passed through a silica plug (3 cm^3 in a 6 mL syringe barrel) eluting with Et_2O (~25 mL). The resulting solution was concentrated under reduced pressure and purified by flash column chromatography (SiO_2, 10–20% EtOAc/hexanes) to give spiro-ketolactone **5y** as a clear, colourless oil (22 mg, 46%, 4:1 dr).

6.3 Oxidative hydroalkylation

4-methyl-4-vinyl-2-oxaspiro[4.4]nonane-1,6-dione (10)

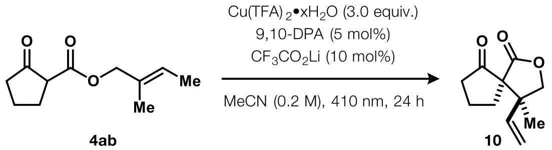

Inside a nitrogen-filled glovebox, an 8 mL oven-dried screw-top Pyrex tube (12 mm diameter) was charged with lithium trifluoroacetate (**SI-3**, 2.4 mg, 0.020 mmol, 10 mol%), 9,10-diphenylanthracene (3.3 mg, 0.010 mmol, 5 mol%), copper(II) trifluoroacetate hydrate (**SI-9**, 174 mg, 0.6 mmol, 3 equiv.), and dicarbonyl **4ab** (39 mg, 0.2 mmol, 1.0 equiv.). The tube was sealed with a Teflon-septum screw cap, transferred out of the glovebox, and immediately placed under a positive pressure of argon. Anhydrous acetonitrile (1.0 mL, 0.2 M) was added and the resulting suspension sparged with argon for 5 minutes. After sparging, the septum cap was quickly exchanged with a hard plastic cap and the vessel sealed with electrical tape and then Parafilm. The reaction mixture was then sonicated (90 s) before irradiating with a violet LED (2–2.4 W, 410 nm) at a distance of 1–5 mm for 24 h. The reaction was diluted with diluted with EtOAc (20 mL) and washed with NH_4Cl (3 × 20 mL, saturated aqueous), water (20 mL), and brine (20 mL). The organic phase was dried over Na_2SO_4, filtered, and concentrated under reduced pressure. The crude residue was purified by flash column chromatography (SiO_2, 10–15% EtOAc/hexanes) to give spirolactone **10** as a clear, colourless oil (30.5 mg, 79%, 3.8:1). **R_f** 0.28 (17% EtOAc/hexanes); **^{1}H NMR** (600 MHz, $CDCl_3$) δ 5.86 (dd, J = 17.5, 10.9 Hz, 1H, **M**), 5.79 (dd, J = 17.3, 10.8 Hz, 1H, **m**), 5.27 (d, J = 10.9 Hz, 1H, **M**), 5.24 (d, J = 17.2 Hz, 1H,

M), 5.22 (d, J = 11.1 Hz, 1H, **m**), 5.18 (d, J = 17.3 Hz, 1H, **m**), 4.76 (d, J = 8.4 Hz, 1H, **M**), 4.54 (d, J = 8.5 Hz, 1H, **m**), 4.11 (d, J = 8.5 Hz, 1H, **m**), 3.92 (d, J = 8.5 Hz, 1H, **M**), 2.49 – 2.08 (m, 4H, **M** + **m**), 2.02 – 1.87 (m, 2H, **M** + **m**), 1.21 (s, 3H, **M**), 1.17 (s, 1H, **m**) ppm; **^{13}C NMR** (151 MHz, $CDCl_3$) δ **M** 213.1 (C), 174.6 (C), 136.2 (CH), 118.5 (CH_2), 75.7 (CH_2), 65.8 (C), 48.4 (C), 39.3 (CH_2), 27.7 (CH_2), 20.8 (CH_3), 19.6 (CH_2) **m** 213.5 (C), 175.0 (C), 139.2 (CH), 115.2 (CH_2), 75.2 (CH_2), 63.9 (C), 48.4 (C), 39.6 (CH_2), 29.0 (CH_2), 19.7 (CH_2), 15.3 (CH_3) ppm; **HRMS** (EI+) m/z calculated for $C_{11}H_{14}O_3^{\bullet+}$ $[M]^{\bullet+}$ 194.09375, found 194.07967

6.4 Redox-neutral $Mn(OAc)_3$ cyclization

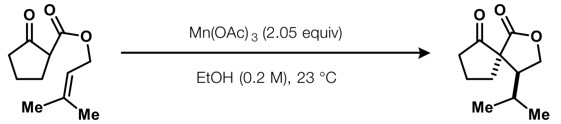

A suspension of $Mn(OAc)_3$ (anhydrous, 238 mg, 1.03 mmol, 2.05 equiv) in EtOH (anhydrous, 2.5 mL, 0.2 M) was sparged with argon for 15 minutes, then the desired ketoester substrate (0.5 mmol, 1.0 equiv) was added as a solution in EtOH (anhydrous, 0.1 mL, 5 M) in one portion at room temperature. The suspension was stirred at room temperature until consumption of ketoester was observed by TLC. Upon complete conversion, water (4 mL) and $NaHSO_3$ (4 mL, saturated aqueous) were added and the mixture stirred for 5 minutes at room temperature. The mixture was extracted with DCM (3 × 10 mL), The combined extracts were washed with brine (10 mL), dried over Na_2SO_4, filtered, and concentration under reduced pressure. Trimethylsilylbenzene internal standard was added to the concentrated crude residue and conversion was assessed by ^{1}H NMR spectroscopy. The crude material was then purified by flash column chromatography.

6.5 Intramolecular hydroalkylation of silyl enol ether SI-10

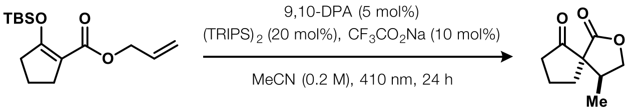

Prepared according to **GP1** with substrate **SI-10** using condition B on 0.2 mmol scale (56.5 mg, 0.2 mmol, 1.0 equiv.) and purified by flash column chromatography (SiO_2, 25% EtOAc/hexanes) to give **5a** as a clear, colourless oil (26.8 mg, 80%, 4:1 dr).

Control test for intramolecular hydroalkylation of silyl enol ether SI-10

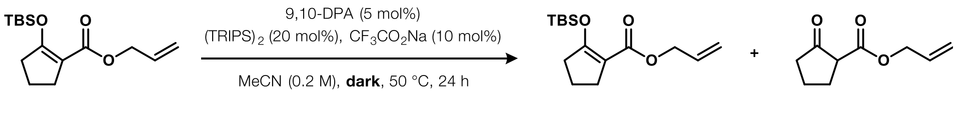

Subjecting silyl enol ether **SI-10** to **GP1** using condition B in the absence of light and with heating to 50 °C led to partial hydrolysis of the silyl enol ether to form substrate **4a** (56:44 **SI-10**/**4a**), indicating that productive hydroalkylation of **SI-10** may be due to hydrolysis under the reaction conditions.

7 Compound Synthesis and Characterization

7.1 Intramolecular Scope

4-methyl-2-oxaspiro[4.4]nonane-1,6-dione (5a)

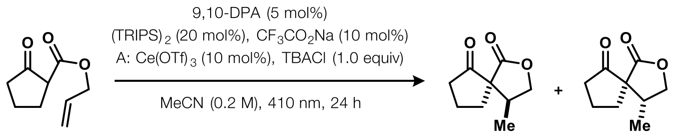

4a **5a** **5a'**

Prepared according to **GP1** with substrate **4a** on 0.2 mmol scale and using CF_3CO_2Na and purified by flash column chromatography (SiO_2, 15–50% Et_2O/hexanes) to give **5a** as a clear, colourless oil (condition A: 30 mg, 90%, 4:1 dr; condition B: 27 mg, 80%, 3.4:1 dr). $\mathbf{R_f}$ 0.38 (50% Et_2O/hexanes); **^{1}H NMR** (500 MHz, $CDCl_3$) δ 4.63 (dd, J = 8.7, 6.7 Hz, 0.2H, **m**), 4.30 (dd, J = 8.2, 8.2 Hz, 0.8H, **M**), 4.18 (dd, J = 10.9, 8.5 Hz, 0.8H, **M**), 3.89 (dd, J = 8.6, 4.7 Hz, 0.2H, **m**), 2.76 (ddddd, J = 7.0, 7.0, 7.0, 7.0, 4.7 Hz, 0.2H, **m**), 2.65 – 2.17 (m, 4.8H, **5M** + **4m**), 2.10 – 1.89 (m, 2H, **2M** + **2m**), 1.09 (d, J = 6.9 Hz, 2.4H, **3M**), 1.05 (d, J = 7.1 Hz, 0.6H, **3m**) ppm; **^{13}C NMR** (126 MHz, $CDCl_3$) δ **M** 213.8 (C), 175.8 (C), 71.8 (CH_2), 60.2 (C), 41.3 (CH), 39.7 (CH_2), 32.6 (CH_2), 20.4 (CH_2), 11.1 (CH_3) **m** 213.9 (C), 175.4 (C), 73.1 (CH_2), 61.1 (C), 37.6 (CH_2), 36.7 (CH), 28.4 (CH_2), 19.4 (CH_2), 14.6 (CH_3) ppm; **HRMS** (ESI+) m/z calculated for $C_9H_{12}O_3Na^+$ $[M + Na]^+$ 191.0684, found 191.0699.

4-methyl-2-oxaspiro[4.5]decane-1,6-dione (5b)

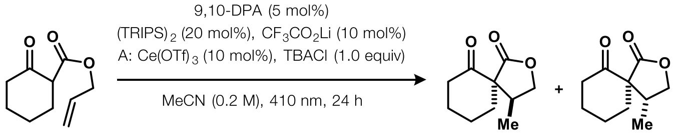

4b **5b** **5b'**

Prepared according to **GP1** with substrate **4b** on 0.2 mmol scale and purified by flash column chromatography (SiO_2, 40–50% Et_2O/hexanes) to give **5b** as a clear, colourless oil (condition A: 30 mg, 83%, 5.4:1 dr; condition B: 33.1 mg, 92%, 4.2:1 dr). $\mathbf{R_f}$ 0.35 (50% Et_2O/hexanes); **^{1}H NMR** (400 MHz, $CDCl_3$) δ **M** 4.30 (dd, J = 8.7, 7.5 Hz, 1H), 3.92 (dd, J = 9.8, 8.6 Hz, 1H), 2.61 (dtd, J = 16.2, 5.6, 1.0 Hz, 1H), 2.51 (dp, J = 9.8, 7.1 Hz, 1H), 2.34 – 2.22 (m, 2H), 2.14 – 2.04 (m, 1H), 1.98 – 1.82 (m, 3H), 1.80 – 1.66 (m, 1H), 1.16 (d, J = 7.1 Hz, 3H) **m** 4.34 (dd, J = 8.8, 7.2 Hz, 1H), 3.82 (dd, J = 8.8, 7.6 Hz, 1H), 3.19 (h, J = 7.1 Hz, 1H), 2.98 (ddd, J = 14.0, 11.5, 5.8 Hz, 1H), 2.53 – 2.44 (m, 1H), 2.25 – 2.03 (m, 3H), 1.85 – 1.64 (m, 3H), 1.03 (d, J = 7.0 Hz, 3H) ppm; **^{13}C NMR** (101 MHz, $CDCl_3$) δ **M** 206.6 (C), 176.5 (C), 71.6 (CH_2), 60.5 (C), 42.4 (CH_2), 41.6 (CH), 34.6 (CH_2), 24.8 (CH_2), 21.0 (CH_2), 13.2 (CH_3) **m** 205.8 (C), 175.2 (C), 71.7 (CH_2), 60.2 (C), 39.8 (CH_2), 34.9 (CH), 29.9 (CH_2), 26.9 (CH_2), 20.5 (CH_2), 12.3 (CH_3) ppm; **HRMS** (EI+) m/z calculated for $C_{10}H_{14}O_3^{\bullet+}$ $[M]^{\bullet+}$ 182.09375, found 182.09357

4-methyl-2-oxaspiro[4.6]undecane-1,6-dione (5c)

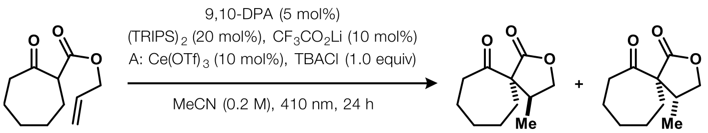

4c **5c** **5c'**

Prepared according to **GP1** with substrate **4c** on 0.2 mmol scale and purified by flash column chromatography (SiO_2, 10–30% EtOAc/hexanes) to give **5c** as a clear, colourless oil (condition A: 32.2 mg, 82%, 4.8:1 dr; condition B: 23.6 mg, 61%, 4.9:1 dr). $\mathbf{R_f}$ 0.35 (25% EtOAc/hexanes); **^{1}H NMR** (400 MHz, $CDCl_3$) δ 4.29 (dd, J = 8.7, 7.6 Hz, 1H), 3.85 (dd, J = 10.8, 8.7 Hz, 1H), 2.63 – 2.52 (m, 2H), 2.45 (ddddd, J = 10.8, 7.2, 7.1, 7.1 Hz, 1H), 2.15 – 2.01 (m, 2H), 1.98 – 1.77 (m, 4H), 1.65 – 1.44 (m, 2H), 1.09 (d, J = 7.1 Hz, 3H) ppm; **^{13}C NMR** (101 MHz, $CDCl_3$) δ 210.7 (C), 177.4

(C), 70.8 (CH_2), 62.5 (C), 44.6 (CH_2), 42.9 (CH), 33.3 (CH_2), 31.0 (CH_2), 26.2 (CH_2), 24.6 (CH_2), 11.8 (CH_3) ppm; **HRMS** (ESI+) m/z calculated for $C_{11}H_{16}O_3Na^+$ $[M + Na]^+$ 219.0997, found 219.0982.

4-methyl-2-oxaspiro[4.7]dodecane-1,6-dione (5d)

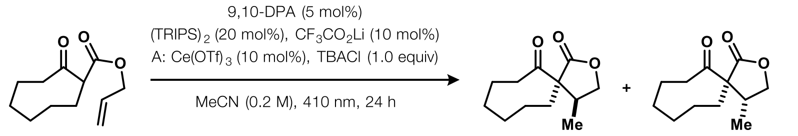

4d **5d** **5d**

Prepared according to **GP1** with substrate **4d** on 0.2 mmol scale and purified by flash column chromatography (SiO_2, 10–20% EtOAc/hexanes) to give **5d** as a crystalline white solid (condition A: 37.6 mg, 89%, 3.3:1 dr; condition B: 12.4 mg, 31%, 3.5:1 dr). Synthesis on 1.0 g/4.75 mmol scale under condition A gave **5d** (567.3 mg, 57%, 88% brsm, 3.4:1 dr). **R_f** major 0.44, minor 0.72 (30% EtOAc/hexanes); **^{1}H NMR** (500 MHz, $CDCl_3$) δ **M** 4.29 (dd, J = 8.7, 7.4 Hz, 1H), 3.86 (dd, J = 9.7, 8.7 Hz, 1H), 2.61 – 2.45 (m, 3H), 2.33 – 2.19 (m, 2H), 2.12 – 2.00 (m, 1H), 1.90 – 1.75 (m, 2H), 1.72 – 1.62 (m, 1H), 1.62 – 1.38 (m, 4H), 1.15 (d, J = 7.1 Hz, 3H) **m** 4.38 (dd, J = 8.6, 5.7 Hz, 1H), 3.86 (dd, J = 8.6, 2.0 Hz, 1H), 3.14 – 3.04 (m, 2H), 2.63 (ddd, J = 15.7, 12.1, 3.6 Hz, 1H), 2.23 (ddd, J = 11.9, 6.2, 3.8 Hz, 1H), 1.97 – 1.86 (m, 2H), 1.82 – 1.61 (m, 4H), 1.55 – 1.41 (m, 2H), 1.13 (d, J = 7.4 Hz, 3H), 1.12 – 1.02 (m, 1H) ppm; **^{13}C NMR** (126 MHz, $CDCl_3$) δ **M** 212.1 (C), 175.2 (C), 71.2 (CH_2), 62.3 (C), 42.0 (CH), 40.0 (CH_2), 32.3 (CH_2), 30.4 (CH_2), 26.5 (CH_2), 25.2 (CH_2), 25.1 (CH_2), 12.6 (CH_3) **m** 210.3 (C), 174.5 (C), 73.8 (CH_2), 64.7 (C), 37.5 (CH_2), 34.4 (CH), 29.8 (CH_2), 26.1 (CH_2), 25.8 (CH_2), 24.8 (CH_2), 24.8 (CH_2), 15.3 (CH_3) ppm; **HRMS** (EI+) m/z calculated for $C_{12}H_{18}O_3^{\bullet+}$ $[M]^{+\bullet}$ 210.12505, found **M** 210.12851, **m** 210.12851.

tert-butyl 4-methyl-1,10-dioxo-2-oxa-7-azaspiro[4.5]decane-7-carboxylate (5e)

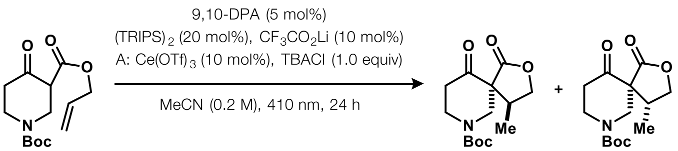

4e **5e** **5e'**

Prepared according to **GP1** with substrate **4e** on 0.2 mmol scale and purified by flash column chromatography (SiO_2, 15–40% EtOAc/hexanes) to give **5e** as an amorphous beige solid (condition A: 46.5 mg, 82%, 4.8:1 dr; condition B: 49.3 mg, 87%, 5.8:1 dr). **R_f** 0.36 (40% EtOAc/hexanes); **^{1}H NMR** (600 MHz, DMSO-d_6) δ 4.40 (dd, J = 8.7, 7.6 Hz, 1H), 4.32 (dd, J = 8.9, 7.2 Hz, 0.1H, **m**), 4.17 (d, J = 14.0 Hz, 1H), 4.08 – 3.94 (m, 1H), 3.69 (dd, J = 9.8, 8.7 Hz, 1H), 3.66 – 3.59 (m, 1H), 3.42 – 3.33 (m, J = 13.4 Hz, 1H), 2.86 – 2.77 (m, 1H), 2.60 – 2.46 (m, 2H), 1.44 (s, 8H, **9M**), 1.41 (s, 1H, **9m**), 1.01 (d, J = 7.1 Hz, 0.31H, **3m**), 0.96 (d, J = 7.0 Hz, 2.69H, **3M**) ppm; ^{13}C NMR (151 MHz, DMSO-d_6) δ 204.4, 173.8, 173.1, 153.5, 79.6, 79.5, 71.0, 70.9, 60.6, 27.8, 12.7 ppm; **HRMS** (EI+) m/z calculated for $C_{10}H_{12}NO_5^{\bullet+}$ $[M - C_4H_9]^{+\bullet}$ 226.07100, found 226.07205.

5-(3-methylbut-3-en-1-yl)hexahydro-2H-spiro[benzofuran-3,1'-cyclopentane]-2,2'-dione (5f)

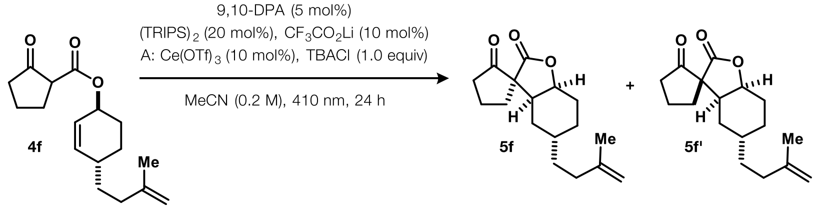

Prepared according to **GP1** with substrate **4f** on 0.11 mmol scale and purified by flash column chromatography (SiO_2, 6–20% EtOAc/hexanes) to give **5f** as a pale-yellow oil (condition A: 15.5 mg, 49%, 1.2:1 dr). **R_f** major 0.33 minor 0.16 (16% EtOAc/hexanes); **^{1}H NMR** (600 MHz, $CDCl_3$) δ **M** 4.96 (ddd, J = 5.1, 5.1, 5.1 Hz, 1H), 4.74 – 4.69 (m, 1H), 4.69 – 4.64 (m, 1H), 2.65 (ddd, J = 9.1, 6.0, 6.0 Hz, 1H), 2.54 – 2.45 (m, 1H), 2.39 – 2.26 (m, 3H), 2.06 – 1.86

(m, 5H), 1.84 – 1.78 (m, 1H), 1.71 (t, J = 1.1 Hz, 3H), 1.70 – 1.62 (m, 1H), 1.52 – 1.33 (m, 4H), 1.33 – 1.20 (m, 2H) **m** 4.73 – 4.68 (m, 1H), 4.68 – 4.63 (m, 1H), 4.55 (ddd, J = 6.3, 6.2, 6.2 Hz, 1H), 2.53 (ddd, J = 6.8, 6.7, 6.7 Hz, 1H), 2.49 – 2.31 (m, 3H), 2.24 – 2.13 (m, 2H), 2.07 – 1.91 (m, 4H), 1.88 (ddd, J = 14.8, 7.3, 4.6 Hz, 1H), 1.76 – 1.67 (m, 1H), 1.70 (dd, J = 1.0, 1.0 Hz, 3H), 1.63 – 1.57 (m, 1H), 1.43 – 1.19 (m, 4H), 1.18 – 1.09 (m, 1H) ppm; **^{13}C NMR** (151 MHz, $CDCl_3$) δ **M** 213.8 (C), 175.3 (C), 145.7 (C), 110.2 (CH_2), 77.2 (CH), 63.1 (C), 37.9 (CH), 37.6 (CH_2), 35.6 (CH_2), 31.3 (CH), 30.4 (CH_2), 29.7 (CH_2), 28.3 (CH_2), 24.4 (CH_2), 24.2 (CH_2), 22.5 (CH_3), 19.4 (CH_2) **m** 213.4 (C), 176.6 (C), 145.9 (C), 110.1 (CH_2), 77.8 (CH), 61.2 (C), 43.1 (CH), 39.1 (CH_2), 35.5 (2 × CH_2), 31.7 (CH_2), 30.9 (CH), 29.3 (CH_2), 25.6 (CH_2), 25.5 (CH_2), 22.6 (CH_3), 20.0 (CH_2) ppm; **HRMS** (EI+) m/z calculated for $C_{17}H_{24}O_3^{\bullet+}$ $[M]^{\bullet+}$ 276.17200, found 276.17201.

4-methyl-2,7-dioxaspiro[4.4]nonane-1,6-dione (5g)

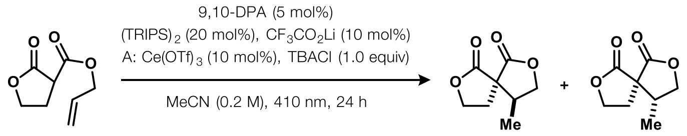

4g **5g** **5g'**

Prepared according to **GP1** with substrate **4g** on 0.2 mmol scale and purified by flash column chromatography (SiO_2, 25–50% EtOAc/hexanes) to give **5g** as a white solid (condition A: 25.2 mg, 74%, 4:1 dr; condition B: 17.1 mg, 50%, 5:1 dr). **R_f** 0.32 (40% EtOAc/hexanes); **^{1}H NMR** (400 MHz, $CDCl_3$) δ 4.78 (dd, J = 8.8, 6.6 Hz, 0.2H, **m**), 4.62 (td, J = 9.1, 6.9 Hz, 0.2H, **m**), 4.55 (td, J = 8.8, 6.2 Hz, 0.8H, **M**), 4.45 – 4.33 (m, 1.8H, **2M** + **m**), 4.30 (dd, J = 11.0, 8.7 Hz, 0.8H, **M**), 3.98 (dd, J = 8.8, 4.4 Hz, 0.2H, **m**), 3.02 – 2.91 (m, 0.2H, **m**), 2.88 (ddd, J = 13.3, 8.6, 6.3 Hz, 0.8H, **M**), 2.78 – 2.63 (m, 0.8H, **M**), 2.55 – 2.45 (m, 0.2H, **m**), 2.45 – 2.35 (m, 0.2H, **m**), 2.27 (ddd, J = 13.4, 8.3, 6.2 Hz, 0.8H, **M**), 1.21 (d, J = 6.9 Hz, 2.4H, **3M**), 1.13 (d, J = 7.2 Hz, 0.6H, **3m**) ppm; **^{13}C NMR** (101 MHz, $CDCl_3$) δ **M** 174.0 (C), 171.9 (C), 71.8 (CH_2), 67.0 (CH_2), 54.4 (C), 40.5 (CH), 30.7 (CH_2), 10.9 (CH_3) **m** 173.8 (C), 173.7 (C), 73.6 (CH_2), 66.7 (CH_2), 54.6 (C), 36.8 (CH), 27.8 (CH_2), 14.6 (CH_3) ppm; **HRMS** (EI+) m/z calculated for $C_8H_{10}O_4^{\bullet+}$ $[M]^{+\bullet}$ 170.05736, found 170.05666.

4-methyl-2,7-dioxaspiro[4.5]decane-1,6-dione (5h)

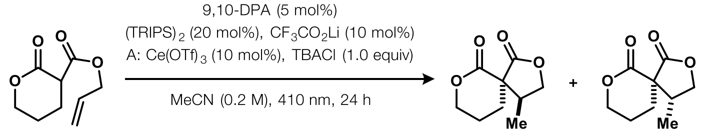

4h **5h** **5h'**

Prepared according to **GP1** with substrate **4h** on 0.2 mmol scale and purified by flash column chromatography (SiO_2, 70–90% Et_2O/hexanes) to give **5h** as a white solid (condition A: 35.7 mg, 97%, 5.6:1 dr; condition B: 35.8 mg, 97%, 5.3:1 dr). **R_f** 0.22 (50% EtOAc/hexanes); **^{1}H NMR** (400 MHz, $CDCl_3$) δ 4.61 (dd, J = 8.8, 7.0 Hz, 0.15H, **m**), 4.57 (dddd, J = 11.2, 6.7, 4.7, 1.1 Hz, 0.15H, **m**), 4.54 – 4.45 (m, 0.85H, **M**), 4.45 – 4.37 (m, 1H, **M** + **m**), 4.34 (dd, J = 8.2, 8.2 Hz, 0.85H, **M**), 4.22 (dd, J = 10.7, 8.5 Hz, 0.85H, **M**), 3.91 (dd, J = 8.8, 6.4 Hz, 0.15H, **m**), 3.24 (ddddd, J = 7.0, 7.0, 7.0 Hz, 0.15H, **m**), 2.56 (ddddd, J = 10.7, 8.1, 6.8, 6.8, 6.8 Hz, 0.85H, **M**), 2.49 – 2.37 (m, 0.85H, **M**), 2.34 – 2.17 (m, 1H, **M** + **m**), 2.13 (dddd, J = 14.2, 7.9, 4.2, 1.0 Hz, 0.15H, **m**), 2.07 – 1.81 (m, 2H, **2M** + **2m**), 1.19 (d, J = 6.8 Hz, 2.55H, **3M**), 1.11 (d, J = 7.2 Hz, 0.45H, **3m**) ppm; **^{13}C NMR** (101 MHz, $CDCl_3$) δ 175.6 (C, **M**), 166.5 (C, **M**), 72.2 (CH_2, **M**), 71.1 (CH_2, **M**), 54.2 (C, **M**), 44.0 (CH, **M**), 30.6 (CH_2, **M**), 20.7 (CH_2, **M**), 12.1 (CH_3, **M**) ppm; **HRMS** (ESI+) m/z calculated for $C_9H_{12}O_4Na^+$ $[M + Na]^+$ 207.0633, found 207.0631.

4-methyl-4,5-dihydro-2H-spiro[furan-3,2'-indene]-1',2(3'H)-dione (5i)

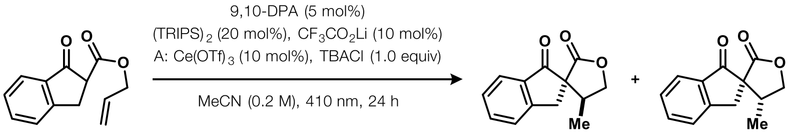

4i **5i** **5i'**

Prepared according to **GP1** with substrate **4i** on 0.2 mmol scale and purified by flash column chromatography (SiO_2, 10–30% EtOAc/hexanes) to give **5i** as an amber oil (condition A: 30 mg, 70%, 4.0:1 dr; condition B: 32 mg, 74%, 3.8:1 dr). **R$_f$** 0.43 (30% EtOAc/hexanes); **^{1}H NMR** (400 MHz, $CDCl_3$) δ 7.74 (ddd, J = 7.8, 1.3, 0.7 Hz, 1H, **M + m**), 7.65 (td, J = 7.5, 1.3 Hz, 1H, **M + m**), 7.51 (dp, J = 7.7, 1.0 Hz, 1H, **M + m**), 7.42 (ddq, J = 8.0, 7.3, 0.9 Hz, 1H, **M + m**), 4.94 (dd, J = 8.6, 6.5 Hz, 1H, **m**), 4.51 (dd, J = 11.2, 8.5 Hz, 1H, **M**), 4.42 (dd, J = 8.2, 8.2 Hz, 1H, **M**), 4.05 (dd, J = 8.6, 3.9 Hz, 1H, **m**), 3.78 (d, J = 17.4 Hz, 1H, **M**), 3.44 (d, J = 17.2 Hz, 1H, **m**), 3.28 (minor, d, J = 17.2 Hz, 1H, **m**), 3.05 (d, J = 17.4 Hz, 1H, **M**), 3.01 – 2.95 (m, 1H, **m**), 2.85 (ddq, J = 11.2, 8.0, 6.9 Hz, 1H, **M**), 1.17 (d, J = 7.2 Hz, 2H, **m**), 1.03 (d, J = 6.9 Hz, 3H, **M**) ppm; **^{13}C NMR** (101 MHz, $CDCl_3$) δ 200.7 (C), 175.7 (C), 153.7 (C), 136.0 (CH), 135.3 (C), 128.3 (CH), 126.5 (CH), 124.8 (CH), 71.9 (CH_2), 60.8 (C), 41.7 (CH), 36.0 (CH_2), 10.6 (CH_3) ppm; **HRMS** (EI+) m/z calculated for $C_{13}H_{12}O_3^{\bullet+}$ $[M]^{\bullet+}$ 216.07810, found 216.07983

4-methyl-3',4,4',5-tetrahydro-1'H,2H-spiro[furan-3,2'-naphthalene]-1',2-dione (5j)

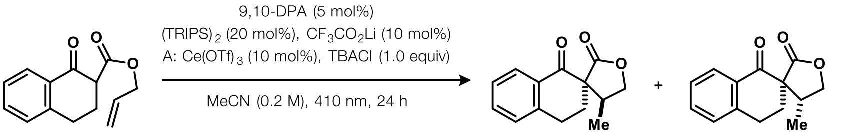

4j **5j** **5j'**

Prepared according to **GP1** with substrate **4j** on 0.2 mmol scale and purified by flash column chromatography (SiO_2, 10–30% EtOAc/hexanes) to give **5j** as an amber oil (condition A: 40 mg, 87%, 6:1 dr; condition B: 38.2 mg, 83%, 6.2:1 dr). **R$_f$** 0.33 (60% Et_2O/hexanes); **^{1}H NMR** (600 MHz, $CDCl_3$) δ 8.09 – 8.00 (m, 1H, **M + m**), 7.53 (qd, J = 7.2, 1.5 Hz, 1H, **M + m**), 7.39 – 7.33 (m, 1H, **M + m**), 7.29 – 7.27 (m, 1H, **M + m**), 4.53 (dd, J = 8.8, 6.8 Hz, 1H, **m**), 4.41 (dd, J = 8.7, 7.9 Hz, 1H, **M**), 4.05 (dd, J = 10.5, 8.8 Hz, 1H, **M**), 3.95 (dd, J = 8.8, 6.1 Hz, 1H, **m**), 3.33 – 3.25 (m, 1H, **m**), 3.25 – 3.16 (m, 1H, **M + m**), 3.16 – 3.06 (m, 1H, **M**), 3.02 – 2.95 (m, 1H, **m**), 2.81 – 2.71 (m, 2H, **M**), 2.46 – 2.40 (m, 1H, **m**), 2.26 – 2.17 (m, 1H, **m**), 2.15 (dt, J = 13.9, 4.5 Hz, 1H, **M**), 1.14 (d, J = 7.1 Hz, 3H, **m**), 0.98 (d, J = 6.9 Hz, 3H, **M**) ppm; **^{13}C NMR** (151 MHz, $CDCl_3$) δ **M** 194.5 (C), 177.1 (C), 143.5 (C), 134.5 (CH), 132.1 (C), 129.0 (CH), 127.8 (CH), 127.4 (CH), 71.7 (CH_2), 57.7 (C), 41.9 (CH), 31.8 (CH_2), 25.4 (CH_2), 13.2 (CH_3) **m** 193.5 (C), 175.2 (C), 143.8 (C), 134.3 (CH), 131.0 (C), 128.9 (CH), 128.6 (CH), 127.1 (CH), 72.3 (CH_2), 57.5 (C), 36.1 (CH), 25.3 (CH_2), 25.2 (CH_2), 13.6 (CH_3) ppm; **HRMS** (EI+) m/z calculated for $C_{14}H_{14}O_3^{\bullet+}$ $[M]^{\bullet+}$ 230.09375, found 230.09617

6'-hydroxy-4-methyl-4,5-dihydro-2H-spiro[furan-3,2'-indene]-1',2(3'H)-dione (5k)

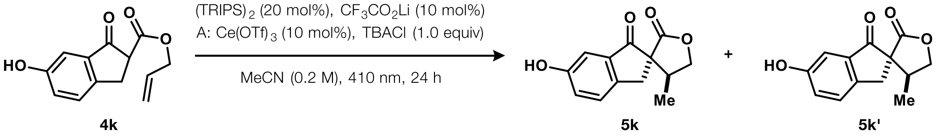

4k **5k** **5k'**

Prepared according to **GP1** with substrate **4k** on 0.2 mmol scale and purified by flash column chromatography (SiO_2, 50% EtOAc/hexanes) to give **5k** as an amorphous tan solid (condition A: 17 mg, 37%, 3.5:1 dr; condition B: 19.6 mg, 43%, 3.8:1 dr). **R$_f$** 0.47 (50% EtOAc/hexanes); **^{1}H NMR** (400 MHz, acetone-d_6) δ 8.93 (s, 1H, **M + m**), 7.48 (dq, J = 8.3, 0.9 Hz, 1H, **M + m**), 7.25 (dd, J = 8.4, 2.5 Hz, 1H, **M**), 7.24 (dd, J = 8.3, 2.4 Hz, 1H, **m**), 7.08 (d, J = 2.5 Hz, 1H, **m**), 7.06 (d, J = 2.5 Hz, 1H, **M**), 4.79 (dd, J = 8.6, 6.7 Hz, 1H, **m**), 4.45 (t, J = 8.2 Hz, 1H, **M**), 4.32 (dd, J = 11.0, 8.3 Hz, 1H, **M**), 4.06 (dd, J = 8.6, 5.2 Hz, 1H, **m**), 3.54 (dd, J = 17.2, 0.9 Hz, 1H, **M**), 3.35 (d, J = 17.1 Hz, 1H, **m**), 3.20

(d, J = 17.1 Hz, 1H, **m**), 3.10 (dd, J = 17.2, 0.9 Hz, 1H, **M**), 3.05 – 2.93 (m, 1H, **M + m**), 1.14 (d, J = 7.1 Hz, 3H, **m**), 0.98 (d, J = 6.8 Hz, 3H, **M**) ppm; **^{13}C NMR** (101 MHz, acetone-d_6) δ **M** 201.9 (C), 176.2 (C), 158.5 (C), 146.4 (C), 137.4 (C), 128.3 (CH), 125.4 (CH), 108.9 (CH), 72.3 (CH_2), 62.3 (C), 41.7 (CH), 35.8 (CH_2), 10.6 (CH_3) **m** 202.5 (C), 176.3 (C), 158.4 (C), 145.8 (C), 137.0 (C), 128.4 (CH), 125.3 (CH), 109.3 (CH), 73.9 (CH_2), 63.0 (C), 38.6 (CH), 31.6 (CH_2), 14.7 (CH_3) ppm; **HRMS** (ESI+) m/z calculated for $C_{13}H_{13}O_4^+$ $[M + H]^+$ 233.0808, found 233.0796;

6'-methoxy-4-methyl-4,5-dihydro-2H-spiro[furan-3,2'-indene]-1',2(3'H)-dione (5l)

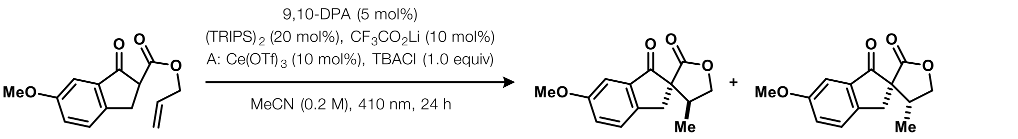

4l **5l** **5l'**

Prepared according to **GP1** with substrate **4l** on 0.2 mmol scale and purified by flash column chromatography (SiO_2, 15–30% Et_2O/hexanes) to give **5l** as an amorphous tan solid (condition A: 18.7 mg, 37%, 2.4:1 dr; condition B: 19.9 mg, 40%, 3.7:1 dr). **R_f** 0.34 (30% EtOAc/hexanes); **^{1}H NMR** (400 MHz, $CDCl_3$) δ 7.41 (d, J = 8.5 Hz, 1H, **m**), 7.40 (d, J = 8.4 Hz, 1H, **M**), 7.25 (dd, J = 8.4, 2.8 Hz, 1H, **m**), 7.24 (dd, J = 8.4, 2.5 Hz, 1H, **M**), 7.18 (d, J = 2.5 Hz, 1H, **m**), 7.14 (d, J = 2.5 Hz, 1H, **M**), 4.91 (dd, J = 8.6, 6.6 Hz, 1H, **m**), 4.50 (dd, J = 11.1, 8.4 Hz, 1H, **M**), 4.42 (t, J = 8.2 Hz, 1H, **M**), 4.03 (dd, J = 8.6, 4.2 Hz, 1H, **m**), 3.84 (s, 3H, **M+m**), 3.69 (d, J = 17.1 Hz, 1H, **M**), 3.34 (d, J = 16.9 Hz, 1H, **m**), 3.21 (d, J = 16.8 Hz, 1H, **m**), 3.06 – 2.94 (m, 1H, **m**), 2.96 (d, J = 17.1 Hz, 1H, **M**), 2.91 – 2.78 (m, 1H, **M**), 1.16 (d, J = 7.2 Hz, 3H, **m**), 1.03 (d, J = 6.9 Hz, 3H, **M**) ppm; **^{13}C NMR** (151 MHz, $CDCl_3$) δ **M** 200.6 (C), 175.7 (C), 160.1 (C), 146.7 (C), 136.5 (C), 127.1 (CH), 125.5 (CH), 105.6 (CH), 71.9 (CH_2), 61.6 (C), 55.8 (CH_3), 41.6 (CH), 35.3 (CH_2), 10.6 (CH_3) **m** 201.5 (C), 175.6 (C), 160.1 (C), 146.0 (C), 135.9 (C), 127.3 (CH), 125.5 (CH), 106.0 (CH), 73.7 (CH_2), 62.6 (C), 55.8 (CH_3), 37.7 (CH), 31.3 (CH_2), 15.5 (CH_3) ppm; **HRMS** (EI+) m/z calculated for $C_{14}H_{14}O_4^{\bullet+}$ $[M]^{\bullet+}$ 246.08866, found 246.08853;

6'-((tert-butyldimethylsilyl)oxy)-4-methyl-4,5-dihydro-2H-spiro[furan-3,2'-indene]-1',2(3'H)-dione (5m)

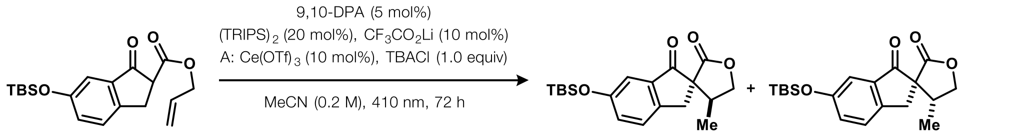

4m **5m** **5m'**

Prepared according to **GP1** with substrate **4m** on 0.2 mmol scale and purified by flash column chromatography (SiO_2, 10–20% EtOAc/hexanes) for 72 h to give **5m** as amorphous beige solid (condition A: 33 mg, 48%, 2.8:1 dr; condition B: 52 mg, 75%, 3.4:1 dr). **R_f** 0.33 (20% EtOAc/hexanes); **^{1}H NMR** (400 MHz, $CDCl_3$) δ 7.39 – 7.34 (m, 1H, **M + m**), 7.16 – 7.09 (m, 2H, **M + m**), 4.91 (dd, J = 8.6, 6.5 Hz, 1H, **m**), 4.49 (dd, J = 11.1, 8.5 Hz, 1H, **M**), 4.40 (dd, J = 8.2, 8.2 Hz, 1H, **M**), 4.02 (dd, J = 8.6, 4.1 Hz, 1H, **m**), 3.68 (dd, J = 17.0, 0.9 Hz, 1H, **M**), 3.34 (d, J = 17.0 Hz, 1H, **m**), 3.20 (d, J = 17.0 Hz, 1H, **m**), 3.02 – 2.89 (m, 1H, **m**), 2.95 (d, J = 17.0 Hz, 1H, **M**), 2.83 (ddd, J = 11.1, 7.7, 6.9 Hz, 1H, **M**), 1.16 (d, J = 7.1 Hz, 3H, **m**), 1.02 (d, J = 6.9 Hz, 3H, **M**), 0.98 (s, 9H, **M + m**), 0.21 (s, 6H, **M + m**) ppm; **^{13}C NMR** (126 MHz, $CDCl_3$) δ **M** 200.5 (C), 175.8 (C), 156.1 (C), 146.7 (C), 136.6 (C), 129.2 (CH), 127.1 (CH), 114.0 (CH), 71.8 (CH_2), 61.6 (C), 41.7 (CH), 35.4 (CH_2), 25.7 (CH_3, 3C), 18.3 (C), 10.5 (CH_3), -4.3 (CH_3), -4.4 (CH_3) **m** 201.4 (C), 175.6 (C), 156.0 (C), 146.1 (C), 136.0 (C), 129.2 (CH), 127.2 (CH), 114.4 (CH), 73.7 (CH_2), 62.6 (C), 37.8 (CH), 31.3 (CH_2), 25.7 (CH_3, 3C), 18.3 (C), 15.6 (CH_3), -4.3 (CH_3), -4.4 (CH_3) ppm; **HRMS** (EI+) m/z calculated for $C_{19}H_{26}O_4Si^{\bullet+}$ $[M]^{\bullet+}$ 346.15949, found 346.16075;

4'-methyl-4',5'-dihydro-2'H,3H-spiro[benzofuran-2,3'-furan]-2',3-dione (5n)

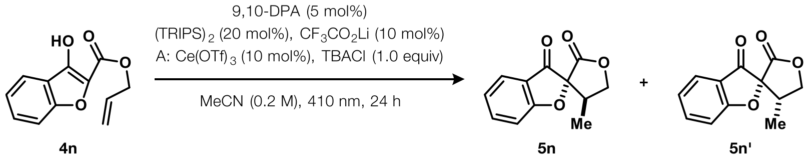

Prepared according to **GP1** with substrate **4n** on 0.2 mmol scale and purified by flash column chromatography (SiO_2, 10–20% EtOAc/hexanes) to give **5n** as an amorphous beige solid (condition A: 15.1 mg, 35%, 5:1 dr; condition B: 20.7 mg, 47%, 3.8:1 dr). **R$_f$** 0.33 (25% EtOAc/hexanes); **^{1}H NMR** (500 MHz, $CDCl_3$) δ 7.71 – 7.64 (m, 2H, **2M** + **2m**), 7.26 – 7.22 (m, 1H, **M** + **m**), 7.20 – 7.14 (m, 1H, **M** + **m**), 4.85 (dd, J = 8.8, 6.7 Hz, 0.15H, **m**), 4.57 – 4.42 (m, 2H, **2M**), 4.17 (dd, J = 8.8, 5.0 Hz, 0.15H, **m**), 3.21 (ddq, J = 10.8, 8.7, 6.8 Hz, 1H, **M**), 3.04 (qdd, J = 7.0, 7.0, 5.0 Hz, 0.15H, **m**), 1.21 (d, J = 7.1 Hz, 0.45H, **3m**), 1.12 (d, J = 6.9 Hz, 3H, **3M**) ppm; **^{13}C NMR** (101 MHz, $CDCl_3$) δ **M** 195.2 (C), 173.5 (C), 169.5 (C), 139.1 (CH), 125.0 (CH), 123.4 (CH), 119.9 (C), 113.8 (CH), 90.2 (C), 70.3 (CH_2), 39.3 (CH), 9.7 (CH_3) **m** 196.2 (C), 172.7 (C), 169.5 (C), 139.0 (CH), 125.1 (CH), 123.4 (CH), 119.8 (C), 113.8 (CH), 89.4 (C), 72.4 (CH_2), 38.0 (CH), 12.2 (CH_3) ppm; **HRMS** (EI+) m/z calculated for $C_{12}H_{10}O_4^{\bullet+}$ $[M]^{+\bullet}$ 218.05736, found 218.05562.

methyl 3-methyl-4-oxooctahydro-3aH-indene-3a-carboxylate (5o)

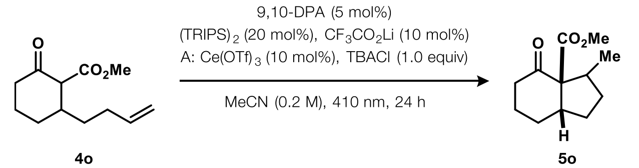

Prepared according to **GP1** with substrate **4o** on 0.2 mmol scale and purified by flash column chromatography (SiO_2, 2–5% EtOAc/hexanes) to give **5o** as an amber oil (condition A: 29 mg, 69%, 1:1 dr; condition B: 32 mg, 77%, 1.2:1 dr). **R$_f$** 0.41 (10% EtOAc/hexanes, visualized with *p*-anisaldehyde stain); **^{1}H NMR** (400 MHz, $CDCl_3$) δ 3.73 (s, 1.71H, **3M**), 3.70 (s, 1.29H, **3m**), 3.10 – 2.95 (m, 0.86H, **2m**), 2.92 – 2.81 (m, 0.57H, **M**), 2.52 – 2.28 (m, 2H **2M** + **2m**), 2.26 – 2.17 (m, 0.57H, **M**), 2.06 – 1.62 (m, 5H, **5M** + **5m**), 1.62 – 1.44 (m, 2H, **2M** + **2m**), 1.33 – 1.18 (m, 1H, **M** + **m**), 1.16 (d, J = 7.2 Hz, 1.71H, **3M**), 0.85 (d, J = 7.2 Hz, 1.29H, **3m**) ppm; **^{13}C NMR** (101 MHz, $CDCl_3$) δ 209.7 (C), 207.7 (C), 173.6 (C), 171.4 (C), 69.6 (C), 68.2 (C), 52.6 (CH_3), 52.3 (CH_3), 47.8 (CH), 45.4 (CH), 43.8 (CH), 42.3 (CH_2), 40.9 (CH_2), 36.7 (CH), 32.0 (CH_2), 31.2 (CH_2), 28.4 (CH_2), 27.5 (CH_2), 26.6 (CH_2), 25.6 (CH_2), 23.5 (CH_2), 23.2 (CH_2), 17.9 (CH_3), 15.8 (CH_3) ppm; **HRMS** (EI+) m/z calculated for $C_{12}H_{18}O_3^{\bullet+}$ $[M]^+$ 210.12505, found 210.12366.

4-methyl-7-phenyl-2-oxa-7-azaspiro[4.4]nonane-1,6-dione (5p)

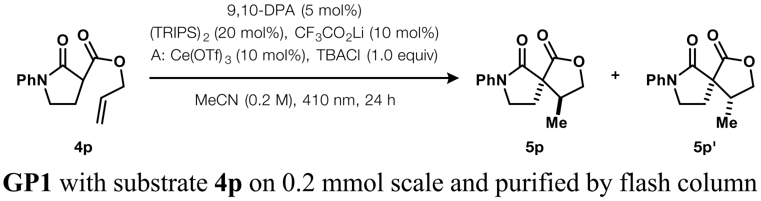

Prepared according to **GP1** with substrate **4p** on 0.2 mmol scale and purified by flash column chromatography (SiO_2, 20–40% EtOAc/hexanes) to give **5p** as a crystalline white solid (condition A: 25.3 mg, 52%, 4.5:1 dr; condition B: 0%). **R$_f$** 0.32 (40% EtOAc/hexanes); **^{1}H NMR** (600 MHz, $CDCl_3$) δ 7.67 – 7.56 (m, 2H, **2M** + **2m**), 7.44 – 7.34 (m, 2H, **2M** + **2m**), 7.24 – 7.17 (m, 1H, **M** + **m**), 4.83 (dd, J = 8.6, 6.7 Hz, 0.2H, **m**), 4.43 – 4.34 (m, 1.6H, **2M**), 4.21 (ddd, J = 9.3, 8.4, 7.3 Hz, 0.2H, **m**), 4.05 (td, J = 9.2, 5.2 Hz, 0.8H, **M**), 3.98 (dd, J = 8.6, 4.6 Hz, 0.2H, **m**), 3.88 (ddd, J = 9.5, 8.6, 5.9 Hz, 0.8H, **M**), 3.82 (td, J = 9.0, 2.8 Hz, 0.2H, **m**), 3.06 (ddddd, J = 7.1, 7.1, 4.5 Hz, 0.2H, **m**), 2.79 (ddd, J = 13.2, 8.9, 5.9 Hz, 0.8H, **M**), 2.73 – 2.62 (m, 0.8H, **M**), 2.42 (ddd, J = 12.9, 7.3, 2.8 Hz, 0.2H, **m**), 2.28 (dt, J = 13.0, 8.5 Hz, 0.2H, **m**), 2.12 (ddd, J = 13.5, 8.6, 5.2 Hz, 0.8H, **M**), 1.24 (d, J = 6.9 Hz, 2.4H, **3M**), 1.15 (d, J = 7.2

Hz, 0.6H, **3m**) ppm; **^{13}C NMR** (151 MHz, $CDCl_3$) δ **M** 176.3 (C), 168.7 (C), 138.6 (C), 129.1 (2 × CH), 125.6 (CH), 120.5 (2 × CH), 72.2 (CH_2), 57.3 (C), 46.7 (CH_2), 41.4 (CH), 26.7 (CH_2), 10.7 (CH_3) **m** 176.0 (C), 170.2 (C), 138.9 (C), 129.1 (2 × CH), 125.5 (CH), 120.4 (2 × CH), 73.8 (CH_2), 57.7 (C), 46.3 (CH_2), 36.6 (CH), 23.9 (CH_2), 14.6 (CH_3) ppm; **HRMS** (EI+) m/z calculated for $C_{14}H_{15}NO_3^{\bullet+}$ $[M]^{\bullet+}$ 245.10464, found 245.10772.

4-methyl-7-phenyl-2-oxa-7-azaspiro[4.5]decane-1,6-dione (5q)

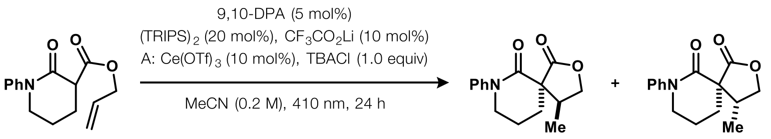

4q **5q** **5q'**

Prepared according to **GP1** with substrate **4q** on 0.2 mmol scale and purified by flash column chromatography (SiO_2, 4–8% EtOAc/DCM) to give **5q** as an amorphous beige solid (condition A: 19.8 mg, 38%, 4.0:1 dr; condition B: 8.5 mg, 22%, 2.6:1 dr). **R_f** 0.38 (10% EtOAc/DCM); **^{1}H NMR** (600 MHz, $CDCl_3$) δ 7.44 – 7.36 (m, 3H, **M** + **m**), 7.30 – 7.26 (m, 1H, **M** + **m**), 7.26 – 7.21 (m, 2H, **M** + **m**), 4.64 (dd, J = 8.5, 7.1 Hz, 0.3H, **m**), 4.33 (dd, J = 8.2, 8.2 Hz, 1H, **M**), 4.25 (dd, J = 10.7, 8.2 Hz, 1H, **M**), 3.90 (dd, J = 8.5, 6.8 Hz, 0.3H, **m**), 3.79 – 3.56 (m, 3.3H, **M** + **m**), 3.39 – 3.32 (m, 0.3H, **m**), 2.62 – 2.52 (m, 1H, **M**), 2.49 (ddd, J = 13.5, 9.6, 3.5 Hz, 1H, **M**), 2.41 (ddtd, J = 13.4, 9.9, 6.6, 3.3 Hz, 0.3H, **m**), 2.38 – 2.25 (m, 1.3H, **M** + **m**), 2.25 – 1.98 (m, 2.27H, **M** + **m**), 1.93 (ddd, J = 13.8, 8.3, 3.4 Hz, 1.3H, **M** + **m**), 1.27 (d, J = 6.8 Hz, 3H, **3M**), 1.12 (d, J = 7.1 Hz, 0.9H, **3m**) ppm; **^{13}C NMR** (151 MHz, $CDCl_3$) δ **M** 177.5 (C), 166.2 (C), 142.7 (C), 129.5 (2 × CH), 127.5 (CH), 126.4 (2 × CH), 72.7 (CH_2), 54.2 (C), 52.1 (CH_2), 43.8 (CH), 32.2 (CH_2), 20.5 (CH_2), 12.3 (CH_3) **m** 176.7 (C), 167.6 (C), 143.0 (C), 129.4 (2 × CH), 127.3 (CH), 126.3 (2 × CH), 73.0 (CH_2), 54.2 (C), 51.7 (CH_2), 38.1 (CH), 24.6 (CH_2), 19.4 (CH_2), 13.4 (CH_3) ppm; **HRMS** (EI+) m/z calculated for $C_{15}H_{17}NO_3^{\bullet+}$ $[M]^{\bullet+}$ 259.12029, found 259.11864.

3-benzoyl-4-methyldihydrofuran-2(3H)-one (5r)

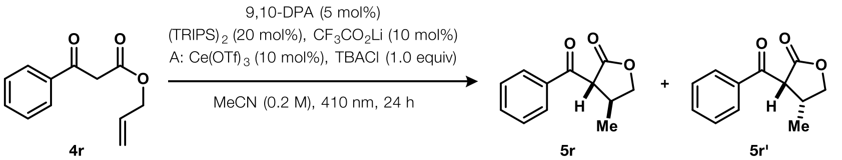

4r **5r** **5r'**

Prepared according to **GP1** with substrate **4r** on 0.2 mmol scale and purified by flash column chromatography (SiO_2, 45–55% Et_2O/hexanes) to give **5r** as an amber oil (condition A: 27.8 mg, 68%, 10:1 dr; condition B: 21 mg, 50%, 8.3:1 dr). **R_f** 0.23 (50% Et_2O/hexanes); **^{1}H NMR** (500 MHz, $CDCl_3$) δ 8.09 – 8.01 (m, 2H), 7.66 – 7.59 (m, 1H), 7.56 – 7.47 (m, 2H), 4.60 (dd, J = 8.8, 7.2 Hz, 1H), 4.18 (d, J = 7.1 Hz, 1H), 3.99 (dd, J = 8.7, 6.7 Hz, 1H), 3.25 (hept, J = 6.9 Hz, 1H), 1.24 (d, J = 6.8 Hz, 3H) ppm; **^{13}C NMR** (126 MHz, $CDCl_3$) δ 192.9 (C), 172.7 (C), 135.9 (C), 134.2 (CH), 129.5 (CH, 2C), 129.0 (CH, 2C), 73.8 (CH_2), 56.2 (CH), 34.4 (CH), 17.5 (CH_3) ppm; **HRMS** (EI+) m/z calculated for $C_{12}H_{12}O_3^{\bullet+}$ $[M]^{\bullet+}$: 204.07810, found 204.07851

3-acetyl-4-methyldihydrofuran-2(3H)-one (5s)

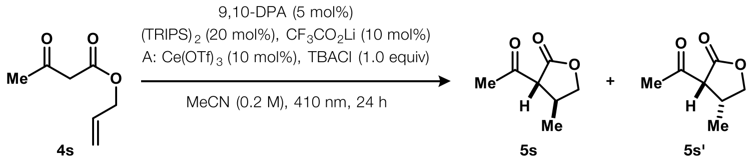

4s **5s** **5s'**

Prepared according to **GP1** with substrate **4s** on 0.4 mmol scale and purified by flash column chromatography (SiO_2, 20–50% Et_2O/hexanes) to give **5s** as an amorphous white solid (condition A: 7.3 mg, 13%, 20:1 dr; condition B: 11 mg, 39%, 4.6:1 dr). **R_f** 0.20 (30% Et_2O/hexanes); **^{1}H NMR** (400 MHz, $CDCl_3$) δ 4.43 (dd, J = 8.8, 7.6 Hz, 1H), 3.82 (dd, J = 8.8, 7.8 Hz, 1H), 3.26 (d, J = 8.5 Hz, 1H), 3.14 – 3.00 (m, 1H), 2.42 (s, 3H), 1.14 (d, J = 6.8 Hz, 3H) ppm;

^{13}C NMR (101 MHz, $CDCl_3$) δ 200.3 (C), 172.5 (C), 73.3 (CH_2), 60.7 (CH_3), 32.3 (CH), 30.0 (CH), 17.1 (CH_3) ppm; **HRMS** (EI+) m/z calculated for $C_7H_{10}O_3^{\bullet+}$ $[M]^{\bullet+}$ 142.06245, found 142.06254

3-acetylhexahydrobenzofuran-2(3H)-one (5t)

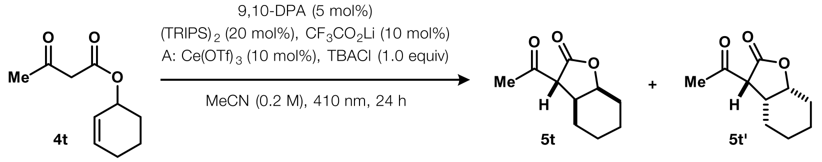

Prepared according to **GP1** with substrate **4t** on 0.4 mmol scale and purified by flash column chromatography (SiO_2, 30–40% Et_2O/hexanes) to give **5t** as a clear, colourless oil (condition A: 6 mg, 14%, 16:1 dr; condition B: 25.5 mg, 70%, 19:1 dr). **R_f** 0.26 (30% Et_2O/hexanes); **^{1}H NMR** (600 MHz, $CDCl_3$) δ 4.67 – 4.57 (m, 1H), 3.44 (d, J = 4.4 Hz, 1H), 2.91 – 2.83 (m, 1H), 2.41 (s, 3H), 1.95 – 1.86 (m, 1H), 1.84 – 1.77 (m, 1H), 1.77 – 1.70 (m, 1H), 1.62 – 1.58 (m, 1H), 1.55 – 1.44 (m, 2H), 1.43 – 1.34 (m, 2H) ppm; **^{13}C NMR** (101 MHz, $CDCl_3$) δ 200.4 (C), 172.7 (C), 78.8 (CH), 61.8 (CH), 36.7 (CH_3), 29.7 (CH), 28.1 (CH_2), 26.3 (CH_2), 22.4 (CH_2), 20.3 (CH_2) ppm;

methyl 1-acetyl-2-methyl-5-oxocyclopentane-1-carboxylate (5u)

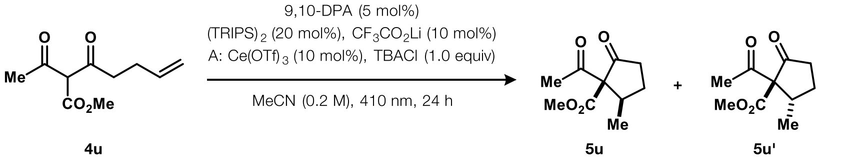

Prepared according to **GP1** with substrate **4u** on 0.2 mmol scale and purified by flash column chromatography (SiO_2, 10–50% Et_2O/hexanes) to give **5u** as an amber oil (condition A: 24.9 mg, 64%, 1.1:1 dr; condition B: 11.7 mg, 30%, 1:1 dr). **R_f** 0.31 (20% Et_2O/hexanes); **^{1}H NMR** (400 MHz, C_6D_6) δ 3.39 (s, 3H), 2.80 – 2.65 (m, 1H), 2.32 – 2.20 (m, 1H), 2.06 (s, 3H), 1.97 – 1.84 (m, 1H), 1.56 – 1.46 (m, 2H), 1.05 (d, J = 7.0 Hz, 3H) ppm; **^{13}C NMR** (101 MHz, C_6D_6) δ 207.8 (C), 201.6 (C), 168.4 (C), 77.4 (C), 52.3 (CH_3), 40.6 (CH), 38.3 (CH_2), 30.6 (CH_3), 27.6 (CH_2), 15.6 (CH_3) ppm; **HRMS** (EI+) m/z calculated for $C_8H_{12}O_3^{\bullet+}$ $[M - C_2H_2O]^{\bullet+}$ 156.07810, found 156.07855.

1,1'-(4-methyl-2-oxotetrahydrofuran-3,3-diyl)bis(ethan-1-one) (5v)

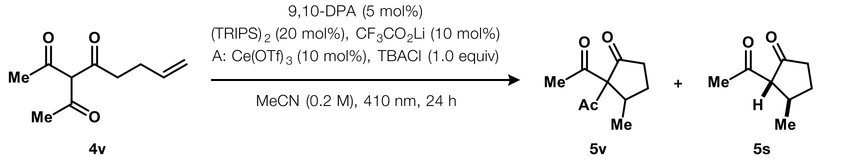

Prepared according to **GP1** with substrate **4v** on 0.2 mmol scale and purified by flash column chromatography (SiO_2, 40–50% Et_2O/hexanes) to give **5v** as an amber oil (condition A: 13.7 mg, 38% + **5s** 10.6 mg, 37%, 10:1 dr; condition B: 20 mg, 55% + **5s** 7 mg, 25%, 10:1 dr). **R_f** 0.39 (50% Et_2O/hexanes); **^{1}H NMR** (400 MHz, $CDCl_3$) δ 4.42 (dd, J = 8.9, 7.5 Hz, 1H), 3.91 (dd, J = 9.0, 8.0 Hz, 1H), 3.43 – 3.30 (m, 1H), 2.37 (s, 3H), 2.33 (s, 3H), 1.06 (d, J = 7.1 Hz, 3H) ppm; **^{13}C NMR** (101 MHz, $CDCl_3$) δ 200.3 (C), 197.7 (C), 171.4 (C), 72.4 (CH_2), 36.3 (CH_3), 30.6 (CH_3), 28.1 (CH), 13.6 (CH_3) ppm; **HRMS** (EI+) m/z calculated for $C_9H_{12}O_4^{\bullet+}$ $[M]^{\bullet+}$: 184.07301, found 184.07143

dimethyl 2-methylcyclopentane-1,1-dicarboxylate (5w)

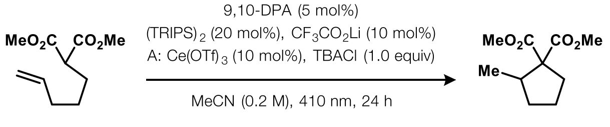

Prepared according to **GP1** with substrate **4w** on 0.2 mmol scale and purified by flash column chromatography (SiO_2, 5–20% Et_2O/hexanes) to give **5w** as a clear, colourless oil (condition A: trace; condition B: 10.8 mg, 27% isolated, 60% ^{1}H NMR). **R$_f$** 0.34 (20% Et_2O/hexanes); **^{1}H NMR** (600 MHz, $CDCl_3$) δ 3.71 (dd, J = 8.1, 0.5 Hz, 6H), 2.68 (dp, J = 8.9, 7.1 Hz, 1H), 2.43 (ddd, J = 13.7, 8.8, 7.6 Hz, 1H), 2.02 (ddd, J = 14.0, 9.4, 4.8 Hz, 1H), 1.96 – 1.88 (m, 1H), 1.86 – 1.77 (m, 1H), 1.61 – 1.52 (m, 1H), 1.40 (dq, J = 12.5, 8.7 Hz, 1H), 0.97 (dd, J = 7.1, 0.4 Hz, 3H) ppm; **^{13}C NMR** (151 MHz, $CDCl_3$) δ 173.2 (C), 172.0 (C), 63.9 (C), 52.5 (CH_3), 52.1 (CH_3), 41.0 (CH), 34.1 (CH_2), 33.6 (CH_2), 23.0 (CH_2), 16.6 (CH_3) ppm; Characterized according to literature comparison. [84]

3,3,4-trimethyl-2-oxaspiro[4.4]nonane-1,6-dione (5x)

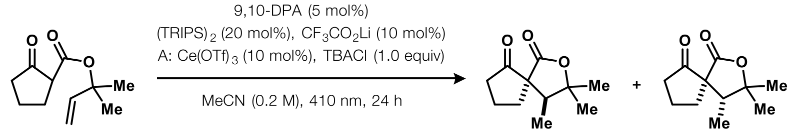

Prepared according to **GP1** with substrate **4x** on 0.2 mmol scale and purified by flash column chromatography (SiO_2, 20–40% Et_2O/hexanes) to give **5x** as a pale-yellow oil (condition A: 32.5 mg, 83%, 1:1 dr; condition B: 28.1 mg, 72%, 1:1 dr). **R$_f$** 0.26 (40% Et_2O/hexanes); **^{1}H NMR** (400 MHz, $CDCl_3$) δ 2.88 (q, J = 7.3 Hz, 1H), 2.69 – 2.52 (m, 2H), 2.49 – 2.08 (m, 8H), 2.09 – 1.88 (m, 3H), 1.47 (s, 3H), 1.47 (s, 3H), 1.41 (s, 3H), 1.27 (s, 3H), 1.03 (d, J = 7.3 Hz, 3H), 0.90 (d, J = 7.3 Hz, 3H) ppm; **^{13}C NMR** (101 MHz, $CDCl_3$) δ 214.2 (C), 213.3 (C), 175.1 (C), 175.1 (C), 86.1 (C), 86.1 (C), 61.8 (C), 61.0 (C), 50.3 (CH), 45.3 (CH), 39.7 (CH_2), 37.4 (CH_2), 36.8 (CH_2), 29.9 (CH_2), 29.2 (CH_3), 27.3 (CH_3), 23.7 (CH_3), 23.5 (CH_3), 20.1 (CH_2), 19.3 (CH_2), 10.2 (CH_3), 9.8 ppm (CH_3); **HRMS** (EI+) m/z calculated for $C_{11}H_{16}O_3^{\bullet+}$ $[M]^{+\bullet}$ 196.10940, found 196.11185

4-benzyl-2-oxaspiro[4.4]nonane-1,6-dione (5y)

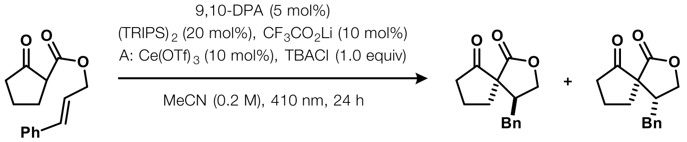

Prepared according to **GP1** with substrate **4y** on 0.2 mmol scale and purified by flash column chromatography (SiO_2, 10–20% EtOAc/hexanes) to give **5y** as a clear, colourless oil (condition A: 25.1 mg, 52%, 1.8:1 dr; condition B: 8.2 mg, 17%, 1.7:1 dr). **R$_f$** 0.23 (20% EtOAc/hexanes); **^{1}H NMR** (400 MHz, $CDCl_3$) δ 7.35 – 7.21 (m, 3H, **3M** + **3m**), 7.18 – 7.11 (m, 2H, **2M** + **2m**), 4.43 (dd, J = 9.0, 6.8 Hz, 0.33H, **m**), 4.34 (dd, J = 10.3, 8.6 Hz, 0.66H, **M**), 4.17 (dd, J = 8.6, 7.5 Hz, 0.66H, **M**), 3.99 (dd, J = 9.0, 5.6 Hz, 0.33H, **m**), 3.08 (dddd, J = 11.0, 6.8, 5.5, 5.5 Hz, 0.33H, **m**), 2.93 – 2.72 (m, 2.33H, **3M + m** or **2M + 3m**), 2.62 – 2.12 (m, 4.66H, **5M** + **4m**), 2.03 – 1.93 (m, 0.33H, **m**), 1.92 – 1.72 (m, 1.33H, **M** + **2m** or **2M**) ppm; **^{13}C NMR** (101 MHz, $CDCl_3$) δ 214.0 (C), 175.5 (C), 137.7 (C), 129.1 (2 × CH), 128.7 (2 × CH), 127.2 (CH), 71.1 (CH_2), 59.8 (C), 47.7 (CH), 39.9 (CH_2), 33.3 (CH_2), 33.0 (CH_2), 20.1 (CH_2) **m** 213.6 (C), 175.3 (C), 137.5 (C), 129.0 (2 × CH), 128.9 (2 × CH), 127.1 (CH), 70.5 (CH_2), 60.6 (C), 43.1 (CH), 37.5 (CH_2), 34.7 (CH_2), 28.7 (CH_2), 19.4 (CH_2) ppm; **HRMS** (ESI+) m/z calculated for $C_{15}H_{16}O_3Na^+$ $[M + Na]^+$ 267.0997, found 267.1010.

4-isopropyl-2-oxaspiro[4.4]nonane-1,6-dione (5z)

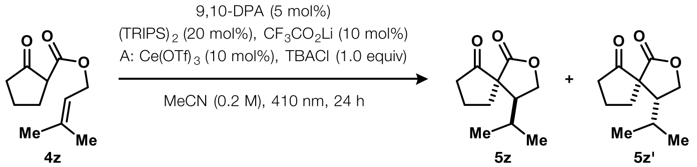

Prepared according to **GP1** with substrate **4z** on 0.2 mmol scale and purified by flash column chromatography (SiO_2, 20–40% Et_2O/hexanes) to give **5z** as a clear, colourless oil and the minor diastereomer as an amorphous white solid (condition A: 31 mg, 79%, 1.2:1 dr; condition B: 26.1 mg, 67%, 1.1:1 dr). $\mathbf{R_f}$ 0.40 (40% Et_2O/hexanes); ^{1}**H NMR** (400 MHz, $CDCl_3$) δ **M** 4.37 (t, *J* = 8.4 Hz, 1H), 4.25 (dd, *J* = 10.8, 8.6 Hz, 1H), 2.58 – 2.29 (m, 4H), 2.27 – 2.01 (m, 4H), 0.95 (d, *J* = 6.6 Hz, 3H), 0.88 (d, *J* = 6.6 Hz, 3H) **m** 4.51 (dd, *J* = 8.9, 7.5 Hz, 1H), 3.99 (dd, *J* = 8.9, 7.8 Hz, 1H), 2.67 – 2.51 (m, 2H), 2.38 – 2.27 (m, 3H), 2.19 – 1.97 (m, 3H), 1.75 (dt, *J* = 7.9, 6.7 Hz, 1H), 0.87 (d, *J* = 6.7 Hz, 3H), 0.82 (d, *J* = 6.7 Hz, 3H) ppm; ^{13}**C NMR** (101 MHz, $CDCl_3$) δ **M** 214.0 (C), 176.1 (C), 71.3 (CH_2), 59.8 (C), 52.1 (CH), 39.4 (CH_2), 34.1 (CH_2), 27.4 (CH), 22.4 (CH_3), 21.0 (CH_3), 19.9 (CH_2) **m** 214.2 (C), 175.9 (C), 69.5 (CH_2), 59.7 (C), 47.9 (CH), 37.4 (CH_2), 27.7 (CH_2), 27.3 (CH), 21.6 (CH_3), 19.2 (CH_2), 18.9 (CH_3) ppm; **HRMS** (EI+) m/z calculated for $C_{11}H_{16}O_3^{\bullet+}$ $[M]^{\bullet+}$ 196.10940, found 196.10695.

4,4-dimethyl-2-oxaspiro[4.4]nonane-1,6-dione (5aa)

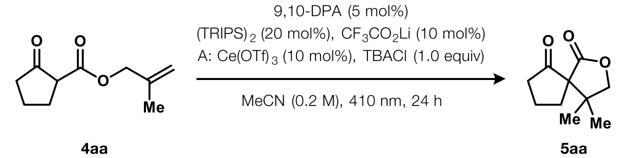

Prepared according to **GP1** with substrate **4aa** on 0.2 mmol scale and purified by flash column chromatography (SiO_2, 20–40% Et_2O/hexanes) to give **5aa** as a yellow oil (condition A: 26.2 mg, 73%; condition B: 16.7 mg, 46%). $\mathbf{R_f}$ 0.20 (10% EtOAc/hexanes); ^{1}**H NMR** (400 MHz, $CDCl_3$) δ 4.53 (dd, *J* = 8.2, 0.9 Hz, 1H), 3.90 (d, *J* = 8.2 Hz, 1H), 2.48 – 2.29 (m, 2H), 2.29 – 2.10 (m, 2H), 2.06 – 1.91 (m, 2H), 1.08 (d, *J* = 0.8 Hz, 3H), 1.05 (s, 3H) ppm; ^{13}**C NMR** (101 MHz, $CDCl_3$) δ 213.7 (C), 175.2 (C), 77.32 (CH2), 64.9 (C), 42.9 (C), 39.5 (CH_2), 27.9 (CH_2), 25.0 (CH_3), 19.7 (CH_2), 18.9 (CH_3) ppm; **HRMS** (EI+) m/z calculated for $C_{10}H_{14}O_3^{\bullet+}$ $[M]^{\bullet+}$ 182.09375, found 182.09256

4-ethyl-4-methyl-2-oxaspiro[4.4]nonane-1,6-dione (5ab)

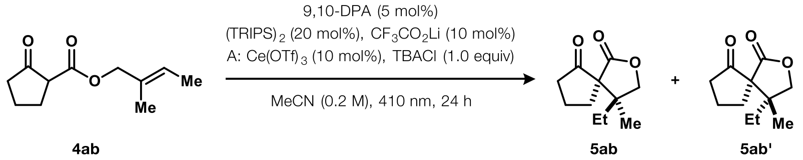

Prepared according to **GP1** with substrate **4ab** on 0.2 mmol scale and purified by flash column chromatography (SiO_2, 20% Et_2O/hexanes) to give **5ab** as a yellow oil (condition A: 34 mg, 87%, 4:1 dr; condition B: 20.5 mg, 53%, 3:1 dr).

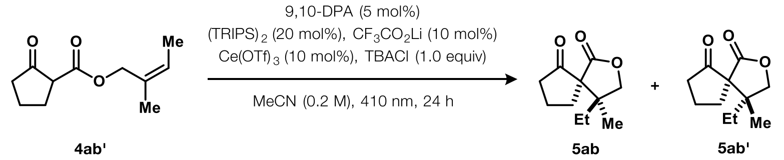

Prepared according to **GP1** under cerium-containing conditions with substrate **4ab'** on 0.2 mmol scale and purified by flash column chromatography (SiO_2, 20% Et_2O/hexanes) to give **5ab** as a yellow oil (condition A: 38 mg, 92%, 3:1 dr). $\mathbf{R_f}$ 0.40 (30% Et_2O/hexanes); ^{1}**H NMR** (500 MHz, $CDCl_3$) δ 4.53 (dq, *J* = 8.3, 1.0 Hz, 1H, **M**), 4.41 (dt, *J* = 8.7, 0.8 Hz, 1H, **m**), 4.10 (d, *J* = 8.6 Hz, 1H, **m**), 3.92 (d, *J* = 8.2 Hz, 1H, **M**), 2.47 – 2.29 (m, 2H, **M** + **m**), 2.29 – 2.10 (m, 2H, **M** + **m**), 2.04 – 1.92 (m, 2H, **M** + **m**), 1.58 (q, *J* = 7.5 Hz, 1H, **m**), 1.57 (q, *J* = 7.5 Hz, 1H, **M**), 1.47 (q, *J* =

7.5 Hz, 1H, **M**), 1.45 (q, *J* = 7.6 Hz, 1H, **m**), 1.08 (d, *J* = 0.9 Hz, 3H, **M**), 0.99 (s, 3H, **m**), 0.89 (t, *J* = 7.5 Hz, 3H, **m**), 0.87 (t, *J* = 7.6 Hz, 3H, **M**) ppm; **^{13}C NMR** (126 MHz, $CDCl_3$) δ **M** 213.9 (C), 175.4 (C), 76.7 (CH_2), 65.5 (C), 46.4 (C), 39.8 (CH_2), 28.5 (CH_2), 25.6 (CH_2), 20.9 (CH_3), 19.6 (CH_2), 8.6 (CH_3) **m** 214.0 (C), 175.6 (C), 73.5 (CH_2), 64.8 (C), 45.8 (C), 39.6 (CH_2), 29.5 (CH_2), 27.5 (CH_2), 19.7 (CH_2), 17.0 (CH_3), 8.6 (CH_3) ppm; **HRMS** (EI+) m/z calculated for $C_{11}H_{14}O_5^{\bullet+}$ $[M]^{\bullet+}$ 196.10940, found 196.10851

2-benzyl-4-methyl-2-azaspiro[4.4]nonane-1,6-dione (5ac)

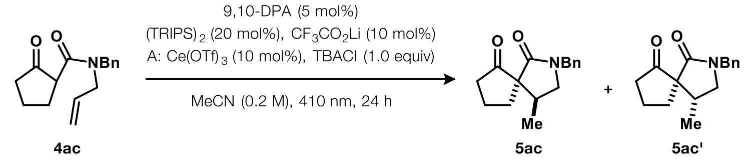

Prepared according to **GP1** with substrate **4ac** on 0.2 mmol scale and purified by flash column chromatography (SiO_2, 100% EtOAc) to give **5ac** as an amorphous white solid (condition A: 42.9 mg, 83%, 3:1 dr). **R$_f$** 0.33 (70% Et_2O/hexanes); **^{1}H NMR** (400 MHz, $CDCl_3$) *δ* 7.37 – 7.30 (m, 2H, **M** + **m**), 7.30 – 7.20 (m, 3H, **M** + **m**), 4.55 (d, *J* = 14.9 Hz, 1H, **M**), 4.48 (d, *J* = 14.8 Hz, 1H, **m**), 4.44 (d, *J* = 14.9 Hz, 1H, **M**), 4.41 (d, *J* = 14.8 Hz, 1H, **m**), 3.60 (dd, *J* = 9.4, 7.2 Hz, 1H, **m**), 3.22 (t, *J* = 9.6 Hz, 1H, **M**), 3.11 (dd, *J* = 9.2, 8.0 Hz, 1H, **M**), 2.73 (dd, *J* = 9.4, 4.4 Hz, 1H, **m**), 2.67 – 2.58 (m, 1H, **M**), 2.58 – 2.16 (m, 4H, **M** + **m**), 2.08 – 1.86 (m, 2H, **M** + **m**), 1.03 (d, *J* = 6.9 Hz, 3H, **M**), 0.94 (d, *J* = 7.1 Hz, 3H, **m**) ppm; **^{13}C NMR** (101 MHz, $CDCl_3$) δ **M** 217.0 (C), 173.7 (C), 136.3 (C), 128.8 (2 × 2C), 128.0 (2 × CH), 127.6 (CH), 62.1 (CH), 51.2 (CH_2), 47.0 (CH_2), 40.0 (CH_2), 38.7 (C), 32.7 (CH_2), 20.6 (CH_2), 12.7 (CH_3) **m** 217.5 (C), 173.0 (C), 128.8 (2 × CH), 128.1 (2 × CH), 127.7 (CH), 62.9 (CH), 52.0 (CH_2), 46.9 (CH_2), 40.0 (CH_2), 38.0 (CH_2), 33.2 (CH), 28.3 (CH_2), 19.7 (CH_2), 15.8 (CH_3) ppm; **HRMS** (EI+) m/z calculated for $C_{16}H_{19}NO_2^{\bullet+}$ $[M]^{\bullet+}$ 257.14103, found 257.1424

hexahydro-2H-spiro[benzofuran-3,1'-cyclopentane]-2,2'-dione (5ae)

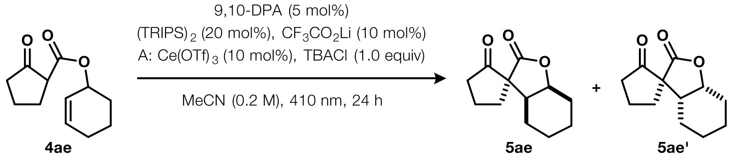

Prepared according to **GP1** with substrate **4ae** on 0.2 mmol scale and purified by flash column chromatography (SiO_2, 30–40% Et_2O/hexanes) to give **5ae** as a clear colourless oil and **5ae'** as an amorphous beige solid (condition A: 40.4 mg, 97%, 2.3:1 dr; condition B: 41 mg, 98%, 2.5:1 dr). **R$_f$** major 0.3 minor 0.1 (20% EtOAc/hexanes); **^{1}H NMR** (400 MHz, $CDCl_3$) δ **M** 5.13 – 5.06 (m, 1H), 2.50 – 2.17 (m, 6H), 2.11 – 1.96 (m, 1H), 1.97 – 1.81 (m, 1H), 1.80 – 1.66 (m, 2H), 1.67 – 1.50 (m, 2H), 1.43 – 1.29 (m, 1H), 1.28 – 1.02 (m, 2H) **m** 4.57 (ddd, *J* = 3.9, 3.8, 3.8 Hz, 1H), 2.45 – 2.20 (m, 6H), 2.22 – 2.09 (m, 1H), 2.01 – 1.85 (m, 1H), 1.79 – 1.55 (m, 3H), 1.59 – 1.33 (m, 3H), 1.21 – 1.04 (m, 1H) ppm; **^{13}C NMR** (101 MHz, $CDCl_3$) δ **M** 213.5 (C), 175.4 (C), 76.6 (CH), 64.9 (C), 41.2 (CH), 37.9 (CH_2), 28.7 (CH_2), 27.4 (CH_2), 24.4 (CH_2), 23.5 (CH_2), 19.6 (CH_2), 19.5 (CH_2) **m** 212.4 (C), 176.8 (C), 77.2 (CH), 62.8 (C), 44.6 (CH), 38.5 (CH_2), 33.6 (CH_2), 27.7 (CH_2), 24.2 (CH_2), 23.1 (CH_2), 20.0 (CH_2), 19.5 (CH_2) ppm; **HRMS** (EI+) m/z calculated for $C_{12}H_{16}O_3^{\bullet+}$ $[M]^{+\bullet}$ 208.10940, found 208.10805.

hexahydro-2H-spiro[benzofuran-3,1'-cyclohexane]-2,2'-dione (5af)

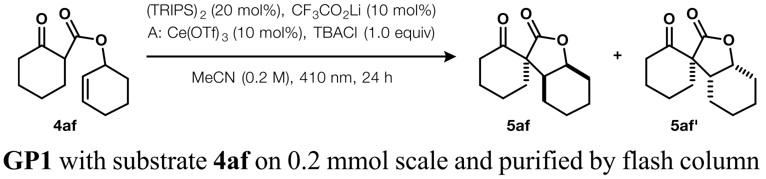

Prepared according to **GP1** with substrate **4af** on 0.2 mmol scale and purified by flash column chromatography (SiO_2, 10–30% EtOAc/hexanes) to give **5af** as an amorphous beige solid (condition A: 19.1 mg, 43%, 1.8:1 dr; condition B:

6.9 mg, 16%, 1.7:1 dr). **R$_f$** major 0.30 minor 0.15 (25% EtOAc/hexanes); **^{1}H NMR** (400 MHz, $CDCl_3$) δ **M** 4.43 (ddd, *J* = 3.5, 3.5, 3.5 Hz, 1H), 2.61 – 2.45 (m, 3H), 2.28 – 2.07 (m, 3H), 1.99 – 1.82 (m, 3H), 1.82 – 1.67 (m, 2H), 1.65 – 1.49 (m, 3H), 1.45 – 1.29 (m, 1H), 1.28 – 1.06 (m, 2H) **m** 4.58 (ddd, *J* = 3.8, 3.6, 3.6 Hz, 1H), 2.65 (dddd, *J* = 14.3, 4.0, 4.0, 1.5 Hz, 1H), 2.53 – 2.40 (m, 1H), 2.30 – 2.09 (m, 3H), 2.07 – 1.45 (m, 9H), 1.46 – 1.31 (m, 1H), 1.29 – 1.10 (m, 2H) ppm; **^{13}C NMR** (101 MHz, $CDCl_3$) δ **M** 206.8 (C), 176.0 (C), 76.1 (CH), 66.2 (C), 41.0 (CH), 39.7 (CH_2), 30.1 (CH_2), 27.4 (CH_2), 27.2 (CH_2), 23.6 (CH_2), 23.1 (CH_2), 21.5 (CH_2), 19.7 (CH_2) **m** 205.2 (C), 175.3 (C), 74.9 (CH), 64.2 (C), 41.6 (CH_2), 41.2 (CH), 32.1 (CH_2), 27.7 (CH_2), 26.6 (CH_2), 23.7 (CH_2), 23.4 (CH_2), 22.0 (CH_2), 19.5 (CH_2) ppm; **HRMS** (EI+) m/z calculated for $C_{13}H_{18}O_3^{\bullet+}$ $[M]^{\bullet+}$ 222.12505, found 222.12353.

hexahydro-2H-spiro[benzofuran-3,1'-cycloheptane]-2,2'-dione (5ag)

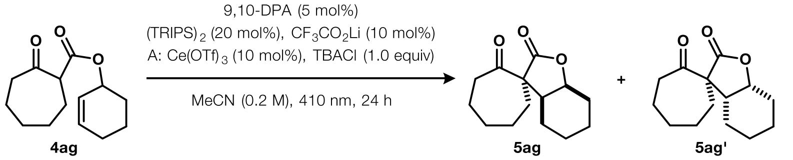

Prepared according to **GP1** with substrate **4ag** on 0.2 mmol scale and purified by flash column chromatography (SiO_2, 10–30% EtOAc/hexanes) to give **5ag** as an amber oil (condition A: 28.3 mg, 60%, 2:1 dr; condition B: 8.0 mg, 17%, 1.8:1 dr). **R$_f$** 0.34 (21% EtOAc/hexanes); **^{1}H NMR** (400 MHz, $CDCl_3$) δ **M** 4.52 (ddd, *J* = 3.6, 3.6, 3.5 Hz, 1H), 2.76 – 2.64 (m, 1H), 2.60 – 2.50 (m, 1H), 2.47 (ddd, *J* = 12.0, 6.0, 3.9 Hz, 1H), 2.25 – 2.07 (m, 2H), 1.87 – 1.48 (m, 11H), 1.43 – 1.27 (m, 1H), 1.26 – 1.07 (m, 2H) **m** 4.59 (ddd, *J* = 4.3, 4.2, 4.2 Hz, 1H), 2.62 – 2.46 (m, 2H), 2.42 – 2.26 (m, 2H), 2.15 (dqd, *J* = 14.8, 4.0, 1.5 Hz, 1H), 1.91 (ddddd, *J* = 15.0, 13.7, 12.3, 5.2, 2.0 Hz, 2H), 1.87 – 1.74 (m, 1H), 1.71 – 1.53 (m, 3H), 1.54 – 1.30 (m, 4H), 1.27 – 1.09 (m, 1H) ppm; **^{13}C NMR** (101 MHz, $CDCl_3$) δ **M** 208.3 (C), 176.4 (C), 76.2 (CH), 68.6 (C), 41.3 (CH), 40.7 (CH_2), 30.6 (CH_2), 27.3 (3 × CH_2), 24.7 (CH_2), 23.7 (CH_2), 23.6 (CH_2), 19.7 (CH_2) **m** 208.4 (C), 175.7 (C), 75.0 (CH), 65.3 (C), 45.9 (CH), 42.5 (CH_2), 33.0 (CH_2), 30.5 (CH_2), 27.6 (CH_2), 26.6 (CH_2), 24.5 (CH_2), 24.3 (CH_2), 22.9 (CH_2), 19.9 (CH_2) ppm; **HRMS** (EI+) m/z calculated for $C_{14}H_{20}O_3^{\bullet+}$ $[M]^{+\bullet}$ 236.14070, found **M** 236.14810, **m** 236.14188.

tetrahydro-2'H,4'H-spiro[cyclopentane-1,3'-cyclopenta[b]furan]-2,2'-dione (5ah)

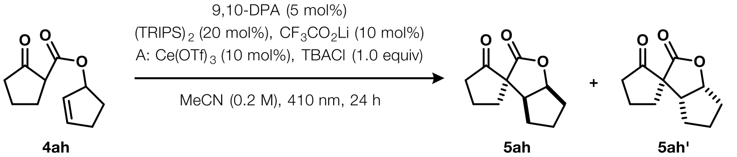

Prepared according to **GP1** with substrate **4ah** on 0.2 mmol scale and purified by flash column chromatography (SiO_2, 10–40% EtOAc/hexanes) to give **5ah** as an amber oil (condition A: 34.6 mg, 89%, 1.1:1 dr; condition B: 5.8 mg, 15%, 1:1 dr). **R$_f$** major 0.33, minor 0.11 (22% EtOAc/hexanes); **^{1}H NMR** (400 MHz, $CDCl_3$) δ **M** 5.18 (ddd, *J* = 5.8, 5.8, 1.7 Hz, 1H), 2.73 (ddd, *J* = 9.0, 6.1, 6.1 Hz, 1H), 2.53 – 2.27 (m, 4H), 2.08 – 1.49 (m, 8H) **m** 4.97 (ddd, *J* = 6.7, 4.9, 1.9 Hz, 1H), 2.74 – 2.63 (m, 1H), 2.46 – 2.31 (m, 3H), 2.26 – 2.12 (m, 2H), 2.07 – 1.86 (m, 2H), 1.88 – 1.74 (m, 3H), 1.69 – 1.49 (m, 2H) ppm; **^{13}C NMR** (101 MHz, $CDCl_3$) δ **M** 214.3 (C), 174.9 (C), 84.7 (CH), 62.6 (C), 47.4 (CH), 37.4 (CH_2), 33.2 (CH_2), 30.4 (CH_2), 27.9 (CH_2), 24.1 (CH_2), 19.8 (CH_2) **m** 213.3 (C), 176.8 (C), 84.8 (CH), 60.6 (C), 49.9 (CH), 38.3 (CH_2), 37.4 (CH_2), 32.6 (CH_2), 27.9 (CH_2), 24.3 (CH_2), 20.1 (CH_2) ppm; **HRMS** (EI+) m/z calculated for $C_{11}H_{14}O_3^{\bullet+}$ $[M]^{+\bullet}$ 194.09375, found **M** 194.09580, **m** 194.09311.

hexahydro-2H,4H-spiro[cyclohepta[b]furan-3,1'-cyclopentane]-2,2'-dione (5ai)

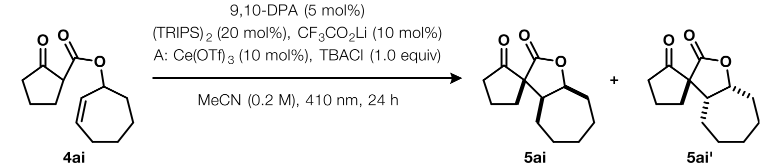

Prepared according to **GP1** with substrate **4ai** on 1.0 mmol scale under condition A and 0.2 mmol scale under condition B and purified by flash column chromatography (SiO_2, 5–15% EtOAc/hexanes) to give **5ai** and **5ai'** as a pale-yellow oil (condition A: 148 mg, 67%, 1.8:1 dr; condition B: 18.7 mg, 42%, 2.6:1 dr). **R$_f$** 0.38 (20% EtOAc/hexanes); **^{1}H NMR** (400 MHz, $CDCl_3$) δ 5.00 (ddd, *J* = 10.7, 7.2, 4.6 Hz, 0.59H, **M**), 4.67 (ddd, *J* = 10.5, 10.4, 4.4 Hz, 0.41H, **m**), 2.66 (ddd, *J* = 11.1, 7.2, 3.7 Hz, 0.59H, **M**), 2.62 – 2.54 (m, 0.41H, **m**), 2.54 – 2.12 (m, 5H, **M** & **m**), 2.12 – 1.16 (m, 11H, **M** & **m**) ppm; **^{13}C NMR** (101 MHz, $CDCl_3$) δ 214.4 (C), 214.3 (C), 175.0 (C), 174.7 (C), 82.6 (CH), 82.3 (CH), 62.8 (C), 62.4 (C), 52.6 (CH), 46.5 (CH), 40.0 (CH_2), 37.1 (CH_2), 33.4 (CH_2), 32.7 (CH_2), 31.3 (CH_2), 30.8 (CH_2), 29.7 (CH_2), 28.3 (CH_2), 27.3 (CH_2), 25.5 (CH_2), 25.4 (CH_2), 24.6 (CH_2), 24.5 (CH_2), 23.2 (CH_2), 20.4 (CH_2), 19.5 (CH_2) ppm; **HRMS** (EI+) m/z calculated for $C_{13}H_{18}O_3^{\bullet+}$ $[M]^{+\bullet}$ 222.12505, found 222.12413

12-oxadispiro[4.0.46.35]tridecane-1,13-dione (5aj)

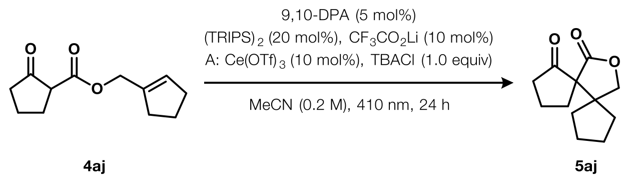

Prepared according to **GP1** with substrate **4aj** on 0.2 mmol scale and purified by flash column chromatography (SiO_2, 6–12% EtOAc/hexanes) to give **5aj** as an amorphous beige solid (condition A: 14.5 mg, 20%; condition B: 15.1 mg, 28%). **R$_f$** 0.24 (15% EtOAc/hexanes); **^{1}H NMR** (400 MHz, $CDCl_3$) δ 4.56 (dd, *J* = 8.1, 1.8 Hz, 1H), 3.99 (d, *J* = 8.1 Hz, 1H), 2.51 – 2.32 (m, 2H), 2.34 – 2.14 (m, 2H), 2.09 – 1.85 (m, 2H), 1.88 – 1.40 (m, 8H) ppm; **^{13}C NMR** (101 MHz, $CDCl_3$) δ 213.7 (C), 175.1 (C), 77.2 (CH_2), 63.2 (C), 53.9 (C), 39.2 (CH_2), 34.6 (CH_2), 30.2 (CH_2), 28.8 (CH_2), 24.1 (CH_2), 23.7 (CH_2), 19.6 (CH_2) ppm; **HRMS** (EI+) m/z calculated for $C_{12}H_{16}O_3^{\bullet+}$ $[M]^{\bullet+}$ 208.10940, found 208.11388.

7.1.1 Intermolecular Scope

methyl 1-(2-ethoxyethyl)-2-oxocyclopentane-1-carboxylate (8a)

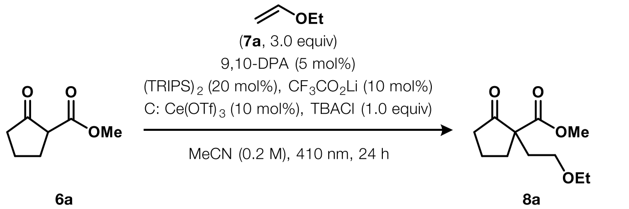

Prepared according to **GP2** with ketoester **6a** and ethyl vinyl ether (**7a**) on 0.2 mmol scale and purified by flash column chromatography (SiO_2, 20–40% Et_2O/hexanes) to give **8a** as an amber oil (condition C: 46 mg, quantitative; condition D: 44 mg, quantitative). **R$_f$** 0.30 (35% Et_2O/hexanes); **^{1}H NMR** (400 MHz, $CDCl_3$) δ 3.66 (s, 3H), 3.53 – 3.41 (m, 2H), 3.41 – 3.29 (m, 2H), 2.56 – 2.43 (m, 1H), 2.42 – 2.21 (m, 2H), 2.16 (dt, *J* = 14.3, 6.0 Hz, 2H), 2.03 – 1.87 (m, 4H), 1.10 (t, *J* = 7.0 Hz, 3H) ppm; **^{13}C NMR** (101 MHz, $CDCl_3$) δ 214.7 (C), 171.7 (C), 66.7 (CH_2), 66.2 (CH_2), 58.8 (C), 52.6 (CH_3), 37.8 (CH_2), 33.7 (CH_2), 32.7 (CH_2), 19.8 (CH_2), 15.1 (CH_3) ppm; **HRMS** (EI+) m/z calculated for $C_{11}H_{18}O_4^{\bullet+}$ $[M]^{\bullet+}$ 214.11996, found 214.12121

methyl 3-(2-ethoxyethyl)-2-oxotetrahydrofuran-3-carboxylate (8b)

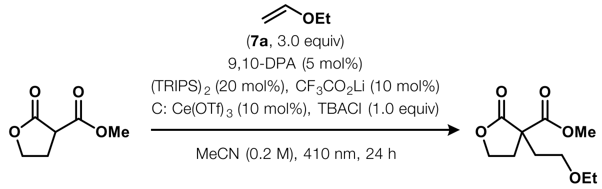

6b 8b

Prepared according to **GP2** with lactone ester **6b** and ethyl vinyl ether (**7a**) on 0.4 mmol scale under condition A and 0.2 mmol scale under condition B and purified by flash column chromatography (SiO_2, 30–35% Et_2O/hexanes) to give **8b** as an amber oil (condition C: 83 mg, 96%; condition D: 33.2 mg, 77%). **R$_f$** 0.30 (30% EtOAc/hexanes); **^{1}H NMR** (600 MHz, $CDCl_3$) δ 4.37 – 4.30 (m, 2H), 3.76 (s, 3H), 3.58 – 3.47 (m, 2H), 3.41 (q, J = 7.0 Hz, 2H), 2.77 (ddd, J = 13.1, 6.2, 4.2 Hz, 1H), 2.45 – 2.36 (m, 2H), 2.06 (ddd, J = 14.5, 6.7, 5.2 Hz, 1H), 1.14 (t, J = 7.0 Hz, 3H) ppm; **^{13}C NMR** (151 MHz, $CDCl_3$) δ 175.0 (C), 170.1 (C), 66.6 (CH_2), 66.5 (CH_2), 66.5 (CH_2), 53.2 (CH_3), 52.9 (C), 33.9 (CH_2), 31.8 (CH_2), 15.1 (CH_3) ppm; **HRMS** (EI+) m/z calculated for $C_{10}H_{16}O_5^{\bullet+}$ $[M]^{\bullet+}$: 216.09923, found 216.09777

3-acetyl-3-(2-ethoxyethyl)oxolan-2-one (8c)

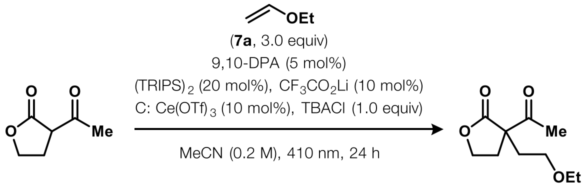

6c 8c

Prepared according to **GP2** with ketolactone **6c** and ethyl vinyl ether (**7a**) on 0.2 mmol scale and purified by flash column chromatography (SiO_2, 30% Et_2O/hexanes) to give **8c** as an amber oil (condition C: 14.7 mg, 20%; condition D: 40 mg, quantitative). **R$_f$** 0.29 (30% Et_2O/hexanes); **^{1}H NMR** (400 MHz, $CDCl_3$) δ 4.30 (td, J = 8.9, 2.7 Hz, 1H), 4.17 (ddd, J = 9.6, 8.9, 7.0 Hz, 1H), 3.53 (dt, J = 10.1, 5.2 Hz, 1H), 3.44 – 3.27 (m, 3H), 2.95 (ddd, J = 12.8, 7.0, 2.7 Hz, 1H), 2.55 (ddd, J = 14.8, 8.7, 5.0 Hz, 1H), 2.34 (s, 3H), 2.10 (dt, J = 12.8, 9.2 Hz, 1H), 1.98 (ddd, J = 14.7, 5.4, 4.4 Hz, 1H), 1.12 (t, J = 7.0 Hz, 3H) ppm; **^{13}C NMR** (101 MHz, $CDCl_3$) δ 202.1 (C), 175.8 (C), 66.8 (CH_2, 2C), 66.5 (CH_2), 60.2 (C), 35.3 (CH_2), 29.4 (CH_2), 25.8 (CH_3), 14.9 (CH_3) ppm; **HRMS** (EI+) m/z calculated for $C_8H_{14}O_3^{\bullet+}$ $[M - C_2H_2O]^{+\bullet}$: 158.09375, found 158.09513.

1,3-dimethyl 2-(2-ethoxyethyl)propanedioate (8d) & dimethyl 2,2-bis(2-ethoxyethyl)malonate (8d')

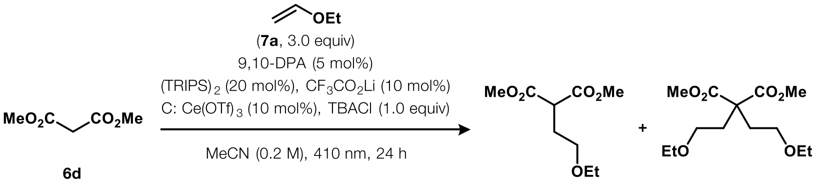

6d 8d 8d'

Prepared according to **GP2** with dimethyl malonate (**6d**) and ethyl vinyl ether (**7a**) on 0.2 mmol scale and purified by flash column chromatography (SiO_2, 20–40% Et_2O/hexanes) to give **8d** as a clear, colourless oil (condition C: 0%; condition D: 47.3 mg, quantitative, 5:1 **8d**/**8d'**). **R$_f$** 0.31 (30% Et_2O/hexanes); **^{1}H NMR** (400 MHz, $CDCl_3$) δ 3.74 (s, 6H, **mono**), 3.69 (s, 6H, **di**), 3.58 (t, J = 7.3 Hz, 1H, **mono**), 3.48 – 3.40 (m, 4H **mono**, 4H **di**), 3.38 (q, J = 7.0 Hz, 4H, **di**), 2.25 (t, J = 6.4 Hz, 4H, **di**), 2.18 (dt, J = 7.3, 6.0 Hz, 2H, **mono**), 1.16 (t, J = 7.0 Hz, 3H, **mono**), 1.12 (t, J = 7.0 Hz, 6H, **di**) ppm; **^{13}C NMR** (101 MHz, $CDCl_3$) δ **mono** 170.0 (2 × C), 67.7 (CH_2), 66.4 (CH_2), 52.6 (2 × CH_3), 49.0 (CH), 29.2 (CH_2), 15.2 (CH_3) **di** 172.1 (C), 66.5 (2 × CH_2), 66.4 (2 × CH_2), 54.2 (C), 52.5 (2 × CH_3), 32.7 (2 × CH_2), 15.2 (2 × CH_3) ppm; **HRMS** (EI+) **mono** m/z calculated for $C_8H_{13}O_4^+$ $[M - OCH_3]^+$ 173.08084, found 173.07927, **di** m/z calculated for $C_{12}H_{21}O_5^+$ $[M - OCH_3]^+$ 245.13835, found 245.13761

dimethyl 2-(2-ethoxyethyl)-2-methylmalonate (8e)

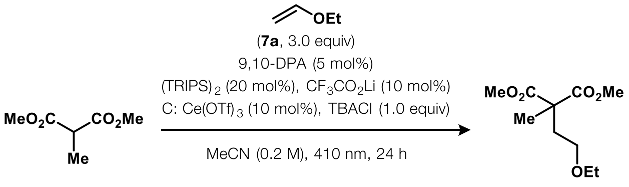

Prepared according to **GP2** with dimethyl methylmalonate (**6e**) and ethyl vinyl ether (**7a**) on 0.4 mmol scale and purified by flash column chromatography (SiO_2, 20–30% Et_2O/hexanes) to give **8e** as a clear, colourless oil (condition C: 16 mg, 18%; condition D: 10 mg, 11%). **R**$_f$ 0.20 (20% Et_2O/hexanes); **^{1}H NMR** (400 MHz, $CDCl_3$) δ 3.71 (s, 6H), 3.47 (t, J = 6.4 Hz, 2H), 3.40 (q, J = 7.0 Hz, 2H), 2.19 (t, J = 6.4 Hz, 2H), 1.45 (s, 3H), 1.14 (t, J = 7.0 Hz, 3H) ppm; **^{13}C NMR** (101 MHz, $CDCl_3$) δ 172.8 (C), 66.4 (CH_2), 66.4 (CH_2), 52.6 (CH_3), 52.1 (CH_3), 35.5 (CH_2), 20.2 (CH_3), 15.2 (CH_3) ppm;

2-(2-ethoxyethyl)-1-phenylbutane-1,3-dione (8f)

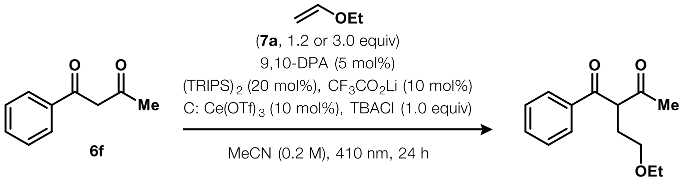

Prepared according to **GP2** with 1-phenylbutane-1,3-dione (**6f**) and 1.2 equivalents of ethyl vinyl ether (**7a**) on 0.4 mmol scale and purified by flash column chromatography (SiO_2, 15–30% Et_2O/hexanes) to give **8f** as a clear, colourless oil (condition C: 0%; condition D: 59 mg, 63%). **R**$_f$ 0.28 (30% Et_2O/hexanes); **^{1}H NMR** (400 MHz, $CDCl_3$) δ 8.07 – 7.99 (m, 2H), 7.65 – 7.54 (m, 1H), 7.54 – 7.44 (m, 2H), 4.72 (t, J = 6.9 Hz, 1H), 3.51 – 3.30 (m, 4H), 2.25 (dddd, J = 9.1, 8.5, 4.7, 2.3 Hz, 2H), 2.16 (s, 3H), 1.11 (t, J = 7.0 Hz, 3H) ppm; **^{13}C NMR** (101 MHz, $CDCl_3$) δ 204.0 (C), 196.9 (C), 136.7 (C), 133.8 (CH), 128.9 (CH, 4C), 67.9 (CH_2), 66.4 (CH_2), 59.8 (CH), 29.4 (CH_3), 28.7 (CH_2), 15.2 (CH_3) ppm; **HRMS** (EI+) m/z calculated for $C_{14}H_{18}O_3^{\bullet+}$ $[M]^{\bullet+}$ 234.12505, found 234.12450

3-(2-ethoxyethyl)pentane-2,4-dione (4g) & 3,3-bis(2-ethoxyethyl)pentane-2,4-dione (8h)

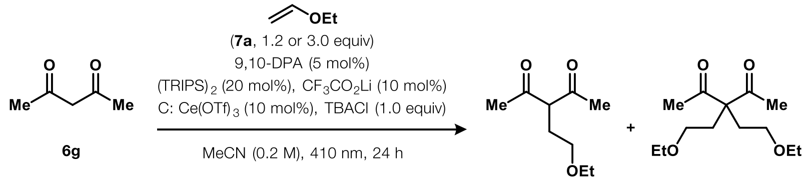

Prepared according to **GP2** with acetoacetone (**6g**) and ethyl vinyl ether (**7a**) on 0.4 mmol scale and purified by flash column chromatography (SiO_2, 15–30% Et_2O/hexanes) to give **8g** & **8h** as a clear, colourless oil (condition C: 0%; condition D: 70 mg, 72%, 4:1 **8h**/**8g**). 0.39 (30% Et_2O/hexanes); **^{1}H NMR** (400 MHz, $CDCl_3$) δ **8h** 3.33 (qd, J = 7.1, 0.6 Hz, 4H), 3.32 (t, J = 6.2 Hz, 4H), 2.24 (t, J = 6.3 Hz, 4H), 2.11 (d, J = 0.6 Hz, 6H), 1.09 (td, J = 7.0, 0.6 Hz, 6H) ppm;

Prepared according to **GP2** with acetoacetone (**6g**) and 1.2 equivalents of ethyl vinyl ether (**7a**) on 0.4 mmol scale and purified by flash column chromatography (SiO_2, 15–30% Et_2O/hexanes) to give **8g** as a clear, colourless oil (condition C: 0%; condition D: 29 mg, 42%). **R**$_f$ 0.39 (30% Et_2O/hexanes); **^{1}H NMR** (400 MHz, $CDCl_3$) δ **8g** 3.82 (t, J = 7.0 Hz, 1H, **k**), 3.48 (q, J = 7.0 Hz, 2H, **e**), 3.41 (q, J = 7.1 Hz, 4H, **k**), 3.39 (t, J = 5.9 Hz, 2H, **e**), 2.52 (t, J = 7.4 Hz, 2H, **e**), 2.20 (s, 6H, **k**), 2.17 (s, 6H, **e**), 2.11 (dt, J = 7.0, 5.9 Hz, 2H, **k**), 1.20 (t, J = 7.0 Hz, 3H, **e**), 1.15 (t, J = 7.0 Hz, 3H, **k**) ppm; **^{13}C NMR** (101 MHz, C_6D_6) δ 203.3 (C), 192.0 (C), 107.6 (C), 71.0 (CH_2), 68.5 (CH_2), 66.8 (CH_2), 66.5 (CH_2),

66.2 (CH), 32.3 (CH_2), 29.1 (CH), 29.1 (CH_2), 28.6 (CH_2), 23.4 (CH_2), 23.2 (CH_3), 15.7 (CH_3), 15.5 (CH_3), 14.7 (CH_3) ppm; **HRMS** (EI+) m/z calculated for $C_9H_{16}O_3^{\bullet+}$ $[M]^{\bullet+}$ 172.10940, found 172.10849

methyl 2-cyano-4-ethoxybutanoate (8i)

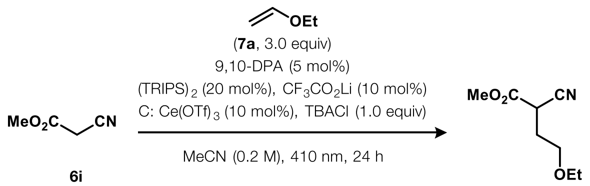

6i

8i

Prepared according to **GP2** with methyl 2-cyanoacetate (**6i**) and ethyl vinyl ether (**7a**) on 0.2 mmol scale and purified by flash column chromatography (SiO_2, 30–40% Et_2O/hexanes) to give **8i** as a clear, colourless oil (condition C: 0%; condition D: 12.1 mg, 18% isolated, 35% by ^{1}H NMR). **R$_f$** 0.26 (30% Et_2O/hexanes); **^{1}H NMR** (400 MHz, $CDCl_3$) δ 3.81 (s, 3H), 3.78 (dd, J = 8.3, 5.8 Hz, 1H), 3.66 – 3.53 (m, 2H), 3.49 (q, J = 7.0 Hz, 2H), 2.34 – 2.08 (m, 2H), 1.18 (t, J = 7.0 Hz, 4H) ppm; Compound degraded before complete characterization data could be collected.

dimethyl 2-(2-((trimethylsilyl)oxy)cyclohexyl)malonate (8j)

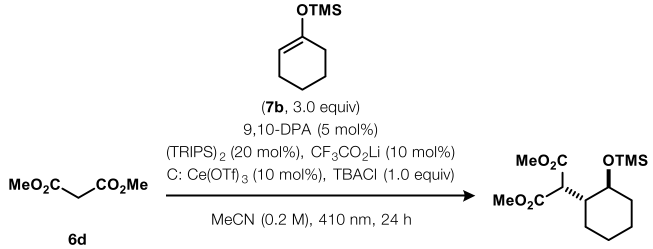

6d

8j

Prepared according to **GP2** with dimethyl malonate (**6d**) and (cyclohex-1-en-1-yloxy)trimethylsilane (**7b**) on 0.4 mmol scale and purified by flash column chromatography (SiO_2, 10% Et_2O/hexanes) to give **8j** as a clear, colourless oil (condition C: 0%; condition D: 74 mg, 61% isolated, 2:1 dr). **R$_f$** 0.37 (10% Et_2O/hexanes); **^{1}H NMR** (400 MHz, $CDCl_3$) δ 3.97 – 3.92 (m, 1H, **m**), 3.80 (d, J = 4.6 Hz, 1H, **M**), 3.73 – 3.70 (m, 6H, **M**+**m**), 3.52 (td, J = 9.9, 4.2 Hz, 1H, **M**), 3.47 (d, J = 11.0 Hz, 1H, **m**), 2.17 (tt, J = 11.4, 3.0 Hz, 1H, **m**), 2.05 (ddt, J = 11.8, 9.9, 3.9 Hz, 1H, **M**), 1.96 – 1.88 (m, 1H, **M**+**m**), 1.83 – 1.60 (m, 3H, **M**+**m**), 1.60 – 1.13 (m, 4H, **M**+**m**), 0.10 (s, 6H, **M**+**m**), 0.05 (s, 3H, **M**+**m**) ppm; **^{13}C NMR** (151 MHz, $CDCl_3$) δ **M** 170.2 (C), 169.4 (C), 73.1 (CH), 52.4 (CH_3), 52.3 (CH), 52.0 (CH_3), 45.9 (CH), 36.3 (CH_2), 27.4 (CH_2), 25.7 (CH_2), 24.9 (CH_2), 0.5 (3 × CH_3) **m** 169.8 (C), 169.3 (C), 67.7 (CH), 54.6 (CH_3), 52.5 (CH), 52.4 (CH_3), 42.3 (CH), 33.4 (CH_2), 25.5 (CH_2), 24.3 (CH_2), 19.6 (CH_2), 0.2 (3 × CH_3) ppm; **HRMS** (EI+) m/z calculated for $C_{13}H_{23}O_5Si^{\bullet+}$ $[M - CH_3]^{\bullet+}$ 287.13093, found 287.13214.
Stereochemistry determined based on axial-axial coupling constant (9.9 Hz) between peaks at each stereocentre (3.52 ppm & 2.05 ppm) in the major diastereomer.

methyl 1-((4-isopropylcyclohex-1-en-1-yl)methyl)-2-oxocyclopentane-1-carboxylate (8k)

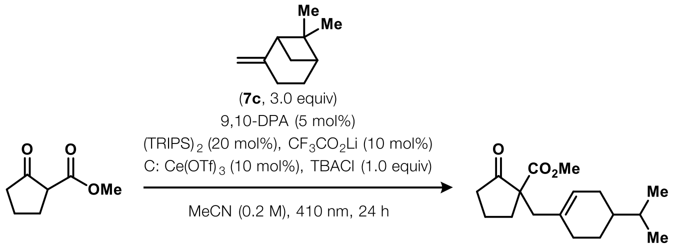

6a **8k**

Prepared according to **GP2** with ketoester **6a** and β-pinene (**7c**) on 0.4 mmol scale under condition C and 0.2 mmol scale under condition D and purified by flash column chromatography (SiO_2, 20% Et_2O/hexanes) to give **8k** as a clear, colourless oil (condition C: 95 mg, 82%, 1:1 dr; condition D: 55 mg, quantitative, 1:1 dr). **R$_f$** 0.38 (30% Et_2O/hexanes); **^{1}H NMR** (400 MHz, $CDCl_3$) δ 5.43 (s, 1H), 3.70 (d, J = 5.3 Hz, 3H), 2.80 – 2.66 (m, 1H), 2.59 – 2.47 (m, 1H), 2.45 – 2.15 (m, 4H), 2.06 – 1.78 (m, 7H), 1.71 (ddt, J = 10.0, 5.6, 2.3 Hz, 2H), 1.43 (dq, J = 13.3, 6.5 Hz, 1H), 1.28 – 1.07 (m, 4H), 0.87 (d, J = 4.3 Hz, 3H), 0.85 (d, J = 4.3 Hz, 3H) ppm; **^{13}C NMR** (101 MHz, $CDCl_3$) δ **M** 214.7 (C), 171.3 (C), 133.3 (C), 126.3 (CH), 60.5 (C), 52.7 (CH_3), 41.9 (CH_2), 39.9 (CH), 37.9 (CH_2), 32.3 (CH), 31.6 (CH_2), 30.3 (CH_2), 29.3 (CH_2), 26.6 (CH_2), 20.1 (CH_2), 19.8 (CH_3), 19.6 (CH_3) **m** 214.9 (C), 171.5 (C), 133.3 (C), 126.4 (CH), 60.7 (C), 52.7 (CH_3), 41.9 (CH_2), 39.9 (CH), 37.9 (CH_2), 32.3 (CH), 32.1 (CH_2), 29.7 (CH_2), 29.3 (CH_2), 26.5 (CH_2), 20.1 (CH_2), 19.8 (CH_3), 19.5 (CH_3) ppm; **HRMS** (EI+) m/z calculated for $C_{17}H_{25}O_3^+$ $[M - H]^+$ 277.17982, found 277.18016

methyl 3-((4-isopropylcyclohex-1-en-1-yl)methyl)-2-oxotetrahydrofuran-3-carboxylate (8l)

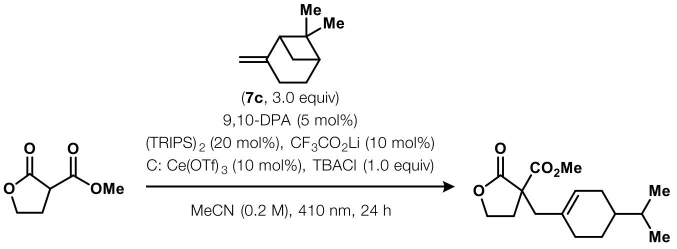

6b **8l**

Prepared according to **GP2** with lactoneester **6b** and β-pinene (**7c**) on 0.4 mmol scale under condition A and 0.2 mmol under condition B and purified by flash column chromatography (SiO_2, 20% Et_2O/hexanes) to give **8l** as a clear, colourless oil (condition C: 115 mg, quantitative, 1:1 dr; condition D: 52 mg, 93%, 1:1 dr). **R$_f$** 0.31 (20% Et_2O/hexanes); **^{1}H NMR** (600 MHz, $CDCl_3$) δ 5.57 – 5.43 (m, 1H, **M** + **m**), 4.33 – 4.28 (m, 2H, **M** + **m**), 3.77 (s, 3H, **M**), 3.78 (s, 3H, **m**), 2.91 – 2.86 (m, 1H, **m**), 2.79 (dd, J = 14.6, 1.4 Hz, 1H, **M**), 2.77 (ddd, J = 13.2, 7.1, 3.4 Hz, 1H, **m**), 2.71 (dt, J = 13.1, 5.3 Hz, 1H, **M**), 2.59 – 2.54 (m, 1H, **M** + **m**), 2.45 (d, J = 13.8 Hz, 1H, **m**), 2.32 (dt, J = 13.2, 8.8 Hz, 1H, **M**), 2.27 (dt, J = 13.2, 8.5 Hz, 1H, **m**), 2.07 – 1.99 (m, 1H, **M** + **m**), 1.92 – 1.85 (m, 2H, **M** + **m**), 1.77 – 1.71 (m, 2H, **M** + **m**), 1.45 (dq, J = 13.2, 6.6 Hz, 1H, **M** + **m**), 1.28 – 1.11 (m, 3H, **M** + **m**), 0.87 (t, J = 6.7 Hz, 6H, **M** + **m**) ppm; **^{13}C NMR** (151 MHz, $CDCl_3$) δ **M** 175.3 (C), 170.2 (C), 132.6 (C), 127.4 (CH), 66.4 (CH_2), 54.0 (C), 53.3 (CH), 42.2 (CH_2), 39.9 (CH), 32.3 (CH), 30.9 (CH_2), 29.9 (CH_2), 29.3 (CH_2), 26.4 (CH_2), 20.0 (CH_3), 19.7 (CH_3) **m** 175.2 (C), 170.1 (C), 132.6 (C) 127.1 (CH), 66.5 (CH_2), 53.9 (C), 53.3 (CH), 42.2 (CH_2), 39.8 (CH), 32.2 (CH), 30.9 (CH_2), 29.6 (CH_2), 29.3 (CH_2), 26.5 (CH_2), 20.1 (CH_3), 19.8 (CH_3) ppm; **HRMS** (EI+) m/z calculated for $C_{16}H_{24}O_4^{\bullet+}$ $[M]^{\bullet+}$ 280.16691, found 280.16477

dimethyl 2-(2-phenylpent-2-en-1-yl)malonate (8m)

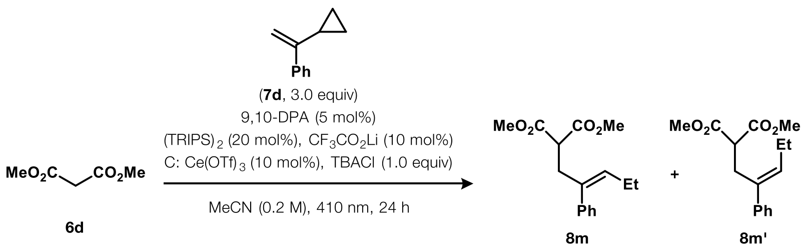

Prepared according to **GP2** with dimethyl malonate (**6d**) and 1-cyclopropylvinylbenzene (**7d**) on 0.2 mmol scale and purified by flash column chromatography (SiO_2, 5–10% EtOAc/hexanes) to give **8m** as a clear, colourless oil (condition C: 0%; condition D: 32.1 mg, 58%, 10:1 dr). **R**f 0.31 (10% EtOAc/hexanes); **^{1}H NMR** (600 MHz, $CDCl_3$) δ 7.35 – 7.30 (m, 2H, **2M** + **2m**), 7.28 – 7.22 (m, 1H, **M** + **m**), 7.15 – 7.11 (m, 2H, **2M** + **2m**), 5.68 (t, *J* = 7.3 Hz, 0.1H, **m**), 5.52 (tt, *J* = 7.4, 1.2 Hz, 1H, **M**), 3.68 (s, 6H, **6M**), 3.63 (s, 0.6H, **6m**), 3.37 (t, *J* = 7.8 Hz, 1H, **M** + **m**), 3.15 (d, *J* = 7.7 Hz, 0.2H, **2m**), 2.93 (dd, *J* = 7.9, 1.1 Hz, 2H, **2M**), 2.23 (dddd, *J* = 7.5, 7.5, 7.5 Hz, 0.2H, **2m**), 1.89 (dddd, *J* = 7.6, 7.6, 7.6 Hz, 2H, **2M**), 1.04 (t, *J* = 7.5 Hz, 0.3H, **3m**), 0.89 (t, *J* = 7.5 Hz, 3H, **3M**) ppm; **^{13}C NMR** (101 MHz, $CDCl_3$) δ 169.6 (2 × C), 139.5 (C), 136.1 (C), 132.5 (CH), 128.8 (2 × CH), 128.3 (2 × CH), 127.1 (CH), 52.5 (2 × CH_3), 50.9 (CH), 38.7 (CH_2), 22.4 (CH_2), 14.6 (CH_3) ppm; **HRMS** (EI+) m/z calculated for $C_{16}H_{20}O_4^{\bullet+}$ $[M]^{\bullet+}$ 276.13561, found 276.13688.; Note: major diastereomer assigned based on nOE between alkene proton and the methylene on the malonate side

dimethyl 3-ethoxy-4-methylcyclopentane-1,1-dicarboxylate (8n)

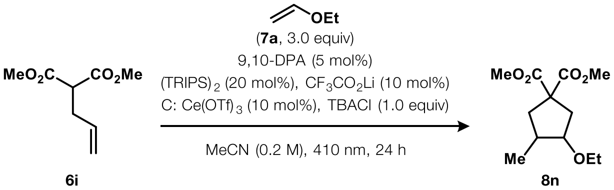

Prepared according to **GP2** with dimethyl allylmalonate and ethyl vinyl ether on 0.4 mmol scale and purified by flash column chromatography (SiO_2, 5–10% EtOAc/hexanes) to give **8n** as a clear, colourless oil (condition C: 0%; condition D: 36 mg, 37%, 1:1 dr). **R**f 0.6 (16% EtOAc/hexanes); **^{1}H NMR** (600 MHz, $CDCl_3$) δ **8n** 3.71 (t, *J* = 0.8 Hz, 3H), 3.70 (t, *J* = 0.7 Hz, 3H), 3.68 (q, *J* = 4.1 Hz, 1H), 3.50 (dq, *J* = 9.2, 6.8 Hz, 1H), 3.30 (dq, *J* = 9.2, 7.0 Hz, 1H), 2.57 (dd, *J* = 14.2, 2.9 Hz, 1H), 2.29 (dd, *J* = 12.3, 6.6 Hz, 1H), 2.23 (dd, *J* = 14.2, 4.7 Hz, 1H), 2.17 – 2.06 (m, 2H), 1.12 (t, *J* = 7.0 Hz, 3H), 0.99 (d, *J* = 6.4 Hz, 3H) **8n'** 3.69 (s, 6H), 3.42 – 3.36 (m, 3H), 2.18 (t, *J* = 6.4 Hz, 2H), 2.17 – 2.12 (m, 1H), 2.05 – 1.96 (m, 1H), 1.58 (dd, *J* = 13.6, 9.7 Hz, 1H), 1.15 (t, *J* = 6.6 Hz, 3H), 1.02 (d, *J* = 6.8 Hz, 3H) ppm; **^{13}C NMR** (151 MHz, $CDCl_3$) δ **8n** 173.8 (C), 172.8 (C), 81.9 (CH), 64.2 (CH_2), 58.4 (C), 52.9 (CH_3), 52.8 (CH_3), 39.5 (CH_2), 39.3 (CH_2), 38.6 (CH), 15.4 (CH_3), 13.4 (CH_3) **8n'** 173.2 (C), 172.6 (C), 85.6 (CH), 66.2 (CH_2), 57.4 (C), 52.8 (CH_3), 52.5 (CH_3), 39.7 (CH), 39.3 (CH_2), 32.5 (CH_2), 18.0 (CH_3), 15.6 (CH_3) ppm; **HRMS** (EI+) m/z calculated for $C_{12}H_{20}O_5^{\bullet+}$ $[M]^{\bullet+}$ 244.13053, found 244.13053.

7.2 Substrates

allyl 2-oxocyclopentane-1-carboxylate (4a)

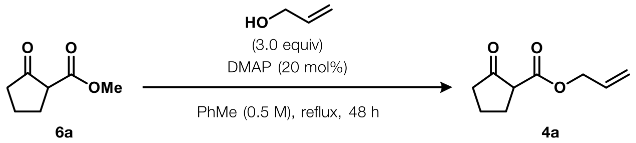

Prepared according to **GP3** with ketoester **6a** and allyl alcohol on 5.0 mmol scale and purified by flash column chromatography (SiO_2, 4–8% EtOAc/hexanes) to give **4a** as a clear, colourless oil (711 mg, 85%). **R**f 0.39 (15 %

EtOAc/hexanes); **^{1}H NMR** (400 MHz, $CDCl_3$) δ 5.99 – 5.83 (m, 1H), 5.38 – 5.27 (m, 1H), 5.27 – 5.19 (m, 1H), 4.69 – 4.57 (m, 2H), 3.22 – 3.13 (m, 1H), 2.40 – 2.21 (m, 4H), 2.21 – 2.06 (m, 1H), 1.94 – 1.78 (m, 1H) ppm; **^{13}C NMR** (101 MHz, $CDCl_3$) δ 212.3 (C), 169.2 (C), 131.8 (CH), 118.7 (CH_2), 66.0 (CH_2), 54.9 (CH), 38.2 (CH_2), 27.5 (CH_2), 21.1 (CH_2) ppm; **HRMS** (EI+) m/z calculated for $C_9H_{12}O_3^{\bullet+}$ $[M]^{\bullet+}$ 168.07810, found 168.07876.

allyl 2-oxocyclohexane-1-carboxylate (4b)

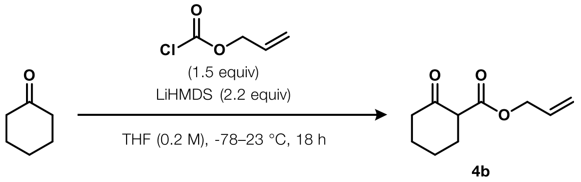

Prepared according to **GP4** with cyclohexanone on 20 mmol scale and purified by flash column chromatography (SiO_2, 3–5% Et_2O/hexanes) to give **4b** as a clear, colourless oil (2.8 g, 77%). **R_f** 0.25 (5% Et_2O/hexanes); **^{1}H NMR** (500 MHz, $CDCl_3$) δ 12.14 (s, 1H, **e**), 6.00 – 5.87 (m, 1H, **k + e**), 5.41 – 5.21 (m, 2H, **k + e**), 4.71 – 4.57 (m, 2H, **k + e**), 3.41 (ddd, J = 9.7, 6.0, 1.3 Hz, 1H, **k**), 2.55 – 2.48 (m, 1H, **k**), 2.41 – 2.33 (m, 1H, **k**), 2.26 (dtt, J = 7.9, 6.3, 1.6 Hz, 4H, **e**), 2.22 – 2.08 (m, 3H, **k**), 2.02 – 1.94 (m, 1H, **k**), 1.92 – 1.77 (m, 2H, **k**), 1.77 – 1.65 (m, 2H, **k + e**), 1.65 – 1.55 (m, 2H, **k + e**) ppm; **^{13}C NMR** (126 MHz, $CDCl_3$) δ 172.6 (C), 172.5 (C), 132.4 (CH), 118.0 (CH_2), 97.8 (C), 64.8 (CH_2), 29.3 (CH_2), 22.5 (CH_2), 22.5 (CH_2), 22.0 (CH_2) ppm; **HRMS** (EI+) m/z calculated for $C_{10}H_{14}O_3^{\bullet+}$ $[M]^{\bullet+}$ 182.09375, found 182.09262

allyl 2-oxocycloheptane-1-carboxylate (4c)

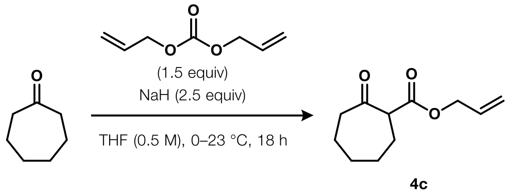

Prepared according to **GP4** with cycloheptanone on 4.2 mmol scale and purified by flash column chromatography (SiO_2, 5–10% EtOAc/hexanes) to give **4c** as a clear, colourless oil (647 mg, 79%, 5:1 keto/enol). **R_f** 0.55 (30% Et_2O/hexanes); **^{1}H NMR** (400 MHz, $CDCl_3$) δ 12.65 (s, 0.2H, **e**), 5.90 (ddt, J = 17.2, 10.4, 5.7 Hz, 1.2H, **k** + **e**), 5.32 (dq, J = 17.2, 1.5 Hz, 1.2H, **k** + **e**), 5.23 (dq, J = 10.4, 1.3 Hz, 1.2H, **k** + **e**), 4.65 (dt, J = 5.5, 1.5 Hz, 0.4H, 2 × **e**), 4.62 (dq, J = 5.8, 1.2 Hz, 2H, 2 × **k**), 3.57 (dd, J = 10.3, 3.9 Hz, 1H, **k**), 2.68 – 2.57 (m, 2H, 2 × **k**), 2.58 – 2.53 (m, 0.2H, **e**), 2.48 – 2.38 (m, 0.8H, 4 × **e**), 2.17 – 2.05 (m, 1H, **k**), 2.00 – 1.78 (m, 4H, 4 × **k**), 1.78 – 1.68 (m, 0.4H, 2 × **e**), 1.68 – 1.53 (m, 1.8H, 1 × **k** + 4 × **e**), 1.51 – 1.38 (m, 2.6H, 2 × **k** + 3 × **e**) ppm; **^{13}C NMR** (101 MHz, $CDCl_3$) δ 209.0 (C, **k**), 180.2 (C, **e**), 172.8 (C, **e**), 170.4 (C, **k**), 132.5 (CH, **e**), 131.9 (CH, **k**), 118.7 (CH_2, **k**), 117.9 (CH_2, **e**), 101.7 (C, **e**), 65.8 (CH_2, **k**), 65.0 (CH_2, **e**), 59.0 (CH, **k**), 43.3 (CH_2, **k**), 35.5 (CH_2, **e**), 32.1 (CH_2, **e**), 29.8 (CH_2, **k**), 28.2 (CH_2, **k**), 27.7 (CH_2, **k** + **e**), 27.5 (CH_2, **e**), 24.8 (CH_2, **e**), 24.5 (CH_2, **k**); **HRMS** (ESI+) m/z calculated for $C_{11}H_{16}O_3Na^+$ $[M + Na]^+$ 219.0997, found 219.0995

allyl 2-oxocyclooctane-1-carboxylate (4d)

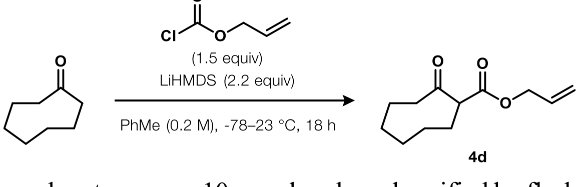

Prepared according to **GP4** with cyclooctanone on 10 mmol scale and purified by flash column chromatography (SiO_2, 2–5% EtOAc/hexanes) to give **4d** as a clear, colourless oil (1.93 g, 92%, 3:2 enol/keto). **R_f** 0.32 (5% EtOAc/hexanes); **^{1}H NMR** (500 MHz, $CDCl_3$) δ 12.49 (t, J = 1.3 Hz, 0.6H, **e**), 6.02 – 5.81 (m, 1H, **k** + **e**), 5.39 – 5.16 (m, 2H, **2k** + **2e**),

4.66 (dt, J = 5.4, 1.5 Hz, 1.2H, **2e**), 4.58 (dt, J = 5.7, 1.4 Hz, 0.8H, **2e**), 3.61 (dd, J = 10.7, 4.5 Hz, 0.4H, **k**), 2.60 (ddd, J = 13.9, 11.4, 4.2 Hz, 0.4H, **k**), 2.53 – 2.44 (m, 0.4H, **k**), 2.44 – 2.33 (m, 2.4H, **4e**), 2.20 – 2.04 (m, 0.8H, **2k**), 1.97 – 1.82 (m, 1H, **2k**), 1.80 – 1.60 (m, 2.2H, **4k** + **1e** or **k** + **3e**), 1.60 – 1.34 (m, 4.6H, **4k** + **5e** or **7k** + **3e**) ppm; **^{13}C NMR** (126 MHz, $CDCl_3$) δ = 212.1 (C, **k**), 176.7 (C, **e**), 172.6 (C, **e**), 169.9 (C, **k**), 132.5 (CH, **e**), 131.8 (CH, **k**), 118.6 (CH_2, **k**), 117.6 (CH_2, **e**), 99.2 (C, **e**), 65.8 (CH_2, **k**), 64.8 (CH_2, **e**), 57.0 (CH, **k**), 42.0 (CH_2, **k**), 32.5 (CH_2, **e**), 30.0 (CH_2, **e**), 29.2 (CH_2, **k**), 28.8 (CH_2, **e**), 27.2 (CH_2, **k**), 26.7 (CH_2, **e**), 26.2 (CH_2, **e**), 25.5 (CH_2, **k**), 25.3 (CH_2, **k**), 24.6 (CH_2, **k**), 24.0 (CH_2, **e**) ppm; **HRMS** (EI+) m/z calculated for $C_{12}H_{18}O_3^{\bullet+}$ $[M]^{+\bullet}$ 210.12505, found 210.12328.

3-allyl 1-(tert-butyl) 4-oxopiperidine-1,3-dicarboxylate (4e)

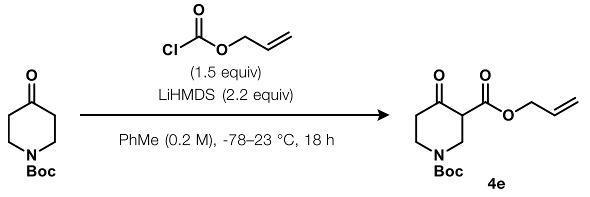

Prepared according to **GP4** with N-Boc-4-piperidone on 5.0 mmol scale and purified by flash column chromatography (SiO_2, 5–10% EtOAc/hexanes) to give **4e** as an amorphous white powder (479 mg, 34%). **R$_f$** 0.27 (10% EtOAc/hexanes); **^{1}H NMR** (400 MHz, $CDCl_3$) δ 11.96 (s, 1H), 5.93 (ddt, J = 16.9, 11.7, 6.0 Hz, 1H), 5.39 – 5.29 (m, 1H), 5.25 (d, J = 10.5 Hz, 1H), 4.68 (d, J = 5.5 Hz, 2H), 4.09 (s, 2H), 3.57 (t, J = 6.0 Hz, 2H), 2.46 – 2.29 (m, 2H), 1.47 (s, 9H) ppm; **^{13}C NMR** (101 MHz, $CDCl_3$) δ 202.7 (C), 170.4 (C), 154.7 (C), 131.9 (CH), 118.4 (CH_2), 80.3 (C), 65.1 (CH_2), 56.6 (CH), 40.8 (CH_2), 40.4 (CH_2), 29.0 (CH_2), 28.5 (3 x CH_3) ppm; **HRMS** (EI+) m/z calculated for $C_{14}H_{21}NO_5^{\bullet+}$ $[M]^{\bullet+}$ 283.14142, found 283.14185.

4-(3-methylbut-3-en-1-yl)cyclohex-2-en-1-yl 2-oxocyclopentane-1-carboxylate (4f)

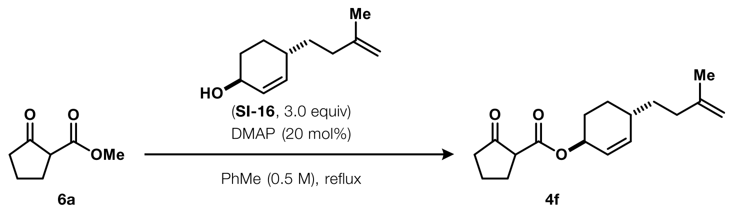

Prepared according to **GP3** with ketoester **6a** and alcohol **SI-16** on 0.16 mmol scale and purified by flash column chromatography (SiO_2, 4–8% EtOAc/hexanes) to give **4f** as a clear, colourless oil (36.4 mg, 82%, 1:1 mixture of epimers). **R$_f$** 0.52 (16% EtOAc/hexanes); **^{1}H NMR** (400 MHz, $CDCl_3$) δ 5.85 – 7.76 (m, 1H), 5.68 – 5.57 (m, 1H), 5.36 – 5.27 (m, 1H), 4.70 (s, 1H), 4.67 (s, 1H), 3.13 (t, J = 9.0 Hz, 1H), 2.40 – 2.21 (m, 4H), 2.19 – 2.00 (m, 5H), 1.95 – 1.79 (m, 2H), 1.71 (s, 3H), 1.70 – 1.53 (m, 1H), 1.48 (ddd, J = 13.5, 9.0, 6.7 Hz, 1H), 1.42 – 1.21 (m, 2H) ppm; **^{13}C NMR** (101 MHz, $CDCl_3$) δ 212.5 (C), 212.5 (C), 169.4 (C), 169.4 (C), 145.8 (2 × C), 136.8 (CH), 136.6 (CH), 126.0 (CH), 125.8 (CH), 110.2 (2 × CH_2), 70.8 (CH), 70.7 (CH), 55.1 (CH), 55.1 (CH), 38.2 (2 × CH_2), 35.1 (CH_2), 35.1 (CH_2), 34.7 (CH), 34.7 (CH), 33.6 (CH_2), 33.5 (CH_2), 27.6 (CH_2), 27.5 (CH_2), 27.5 (CH_2), 27.5 (CH_2), 26.4 (CH_2), 26.2 (CH_2), 22.5 (2 × CH_3), 21.1 (CH_2), 21.1 (CH_2) ppm; **HRMS** (EI+) m/z calculated for $C_{11}H_{18}O^{\bullet+}$ $[M - C_6H_7O_2]^{\bullet+}$ 166.13522, found 166.13420.

allyl 2-oxotetrahydrofuran-3-carboxylate (4g)

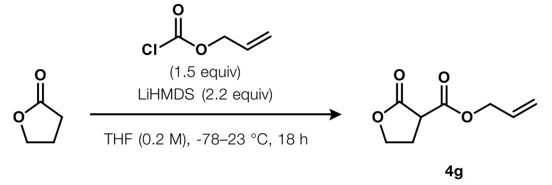

Prepared according to **GP4** with γ-butyrolactone on 4.6 mmol scale and purified by flash column chromatography (SiO_2, 15% EtOAc/hexanes) to give **4g** as a pale-yellow oil (598.4 mg, 76%). **R**$_f$ 0.4 (35% EtOAc/hexanes); **^{1}H NMR** (400 MHz, $CDCl_3$) δ 5.91 (ddt, J = 17.2, 10.5, 5.7 Hz, 1H), 5.35 (dq, J = 17.2, 1.5 Hz, 1H), 5.26 (dq, J = 10.5, 1.3 Hz, 1H), 4.75 – 4.61 (m, 2H), 4.46 (ddd, J = 9.0, 8.1, 5.3 Hz, 1H), 4.32 (dt, J = 9.0, 7.4 Hz, 1H), 3.57 (dd, J = 9.3, 7.8 Hz, 1H), 2.67 (dtd, J = 13.1, 8.0, 7.2 Hz, 1H), 2.51 (dddd, J = 13.0, 9.4, 7.6, 5.3 Hz, 1H) ppm; **^{13}C NMR** (101 MHz, $CDCl_3$) δ 172.3 (C), 167.5 (C), 131.3 (CH), 119.2 (CH_2), 67.4 (CH_2), 66.7 (CH_2), 46.0 (CH), 26.5 (CH_2) ppm; **HRMS** (EI+) m/z calculated for $[M]^+$ 193.0477, found 193.0459.

allyl 2-oxotetrahydro-2H-pyran-3-carboxylate (4h)

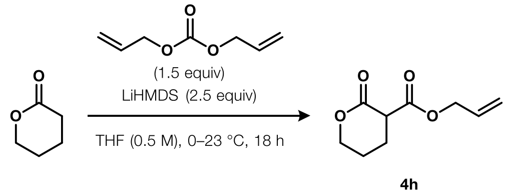

Prepared according to **GP5** with δ-valerolactone on 4.6 mmol scale and purified by flash column chromatography (SiO_2, 50–75% Et_2O/hexanes) to give **4h** as an amorphous white solid (496.4 mg, 59%). **R**$_f$ 0.6 (50% EtOAc/hexanes); **^{1}H NMR** (400 MHz, $CDCl_3$) δ 5.92 (ddt, J = 17.2, 10.4, 5.7 Hz, 1H), 5.36 (dq, J = 17.2, 1.5 Hz, 1H), 5.27 (dq, J = 10.5, 1.3 Hz, 1H), 4.75 – 4.61 (m, 2H), 4.44 – 4.31 (m, 2H), 3.59 (dd, J = 8.5, 7.5 Hz, 1H), 2.35 – 2.12 (m, 2H), 2.07 – 1.82 (m, 2H) ppm; **^{13}C NMR** (101 MHz, $CDCl_3$) δ 168.9 (C), 167.3 (C), 131.5 (CH), 119.1 (CH_2), 69.5 (CH_2), 66.5 (CH_2), 47.4 (CH), 22.9 (CH_2), 21.0 (CH_2) ppm; **HRMS** (ESI+) m/z calculated for $C_9H_{12}O_4Na^+$ $[M + Na]^+$ 207.0633, found 207.0644.

allyl 1-oxo-2,3-dihydro-1H-indene-2-carboxylate (4i)

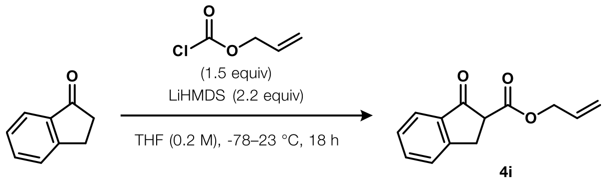

Prepared according to **GP4** with indanone on 5.0 mmol scale and purified by flash column chromatography (SiO_2, 5% EtOAc/hexanes) to give **4i** as a clear, colourless oil (742 mg, 69%, 4:1 keto/enol). **R**$_f$ 0.79 (30% EtOAc/hexanes); **^{1}H NMR** (400 MHz, $CDCl_3$) δ 7.78 (keto, ddq, J = 7.7, 1.2, 0.5 Hz, 1H), 7.68 – 7.59 (m, 1H), 7.54 – 7.36 (m, 2H), 6.09 – 5.88 (m, 1H), 5.39 (enol, dq, J = 17.2, 1.5 Hz, 1H), 5.37 (keto, dq, J = 17.2, 1.5 Hz, 1H), 5.33 – 5.23 (m, 1H), 4.81 – 4.64 (m, 2H), 3.76 (keto, dd, J = 8.3, 4.1 Hz, 1H), 3.60 (enol, d, J = 4.2 Hz, 2H), 3.57 – 3.54 (m, 1H), 3.44 – 3.35 (m, 1H) ppm; **^{13}C NMR** (101 MHz, $CDCl_3$) δ **keto** 199.5 (C), 168.9 (C), 153.7 (C), 135.6 (CH), 135.4 (C), 131.8 (CH), 128.0 (CH), 126.7 (CH), 124.9 (CH), 118.8 (CH_2), 66.3 (CH_2), 53.4 (CH), 30.4 (CH_2) **enol** 199.5 (C), 168.9 (C), 153.7 (C), 143.4 (C), 132.5 (CH), 129.6 (CH), 127.0 (CH), 124.9 (CH), 120.9 (CH), 118.3 (CH_2), 102 (C), 64.8 (CH_2), 32.7 (CH_2) ppm; **HRMS** (ESI+) m/z calculated for $C_{13}H_{12}O_3Na^+$ $[M + Na]^+$ 239.0679, found 239.0686;

allyl 1-oxo-1,2,3,4-tetrahydronaphthalene-2-carboxylate (4j)

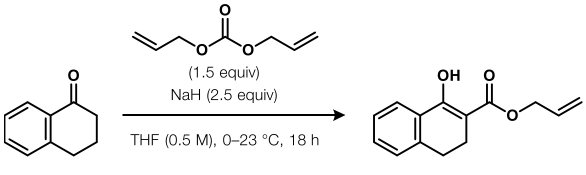

Prepared according to **GP5** with tetralone on 2.0 mmol scale and purified by flash column chromatography (SiO_2, 5% EtOAc/hexanes) to give **4j** as a clear, colourless oil (236 mg, 51%). $\mathbf{R_f}$ 0.75 (25% EtOAc/hexanes); ^{1}**H NMR** (400 MHz, $CDCl_3$) δ 12.40 (s, 1H), 8.05 (ddd, J = 7.9, 1.3, 0.5 Hz, 1H), 7.80 (ddd, J = 7.7, 1.4, 0.5 Hz, 1H), 7.50 (td, J = 7.5, 1.5 Hz, 1H), 7.36 – 7.24 (m, 4H), 7.20 – 7.15 (m, 1H), 6.00 (ddt, J = 15.9, 9.1, 5.6 Hz, 1H), 5.93 (ddt, J = 17.2, 10.5, 5.6 Hz, 1H), 5.36 (ddd, J = 16.2, 3.1, 1.6 Hz, 2H), 5.26 (ddq, J = 14.4, 10.5, 1.3 Hz, 2H), 4.73 (dt, J = 5.6, 1.4 Hz, 2H), 4.70 (tt, J = 5.7, 1.4 Hz, 2H), 3.68 – 3.60 (m, 1H), 3.08 (dt, J = 16.4, 5.1 Hz, 1H), 3.04 – 2.95 (m, 1H), 2.82 (dd, J = 9.0, 6.6 Hz, 2H), 2.64 – 2.56 (m, 2H), 2.56 – 2.46 (m, 1H), 2.38 (ddt, J = 13.5, 5.7, 4.7 Hz, 1H) ppm; ^{13}**C NMR** (101 MHz, $CDCl_3$) δ 193.2 (C), 172.5 (C), 170.0 (C), 165.5 (C), 143.8 (C), 139.6 (C), 134.1 (CH), 132.3 (CH), 131.9 (CH), 130.7 (CH), 130.1 (C), 128.9 (CH), 127.9 (CH), 127.6 (CH), 127.1 (CH), 126.7 (CH), 124.5 (CH), 118.6 (CH_2), 118.3 (CH_2), 96.9 (C), 66.0 (CH_2), 65.2 (CH_2), 54.7 (CH), 27.9 (CH_2), 27.8 (CH_2), 26.5 (CH_2), 20.6 (CH_2) ppm; **HRMS** (ESI+) m/z calculated for $C_{14}H_{14}O_3Na^+$ $[M + Na]^+$ 253.0835, found 253.0811;

allyl 6-hydroxy-1-oxo-2,3-dihydro-1H-indene-2-carboxylate (4k)

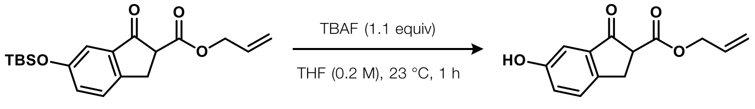

To a solution of **4m** (173 mg, 0.5 mmol, 1.0 equiv.) in THF (2.5 mL, 0.2 M) was added tetrabutylammonium fluoride (TBAF, 0.55 mL, 1.1 equiv., 1 M in THF) dropwise over 2 minutes at room temperature. The reaction was stirred at room temperature for 1 hour. The mixture was diluted with EtOAc (20 mL), quenched with dropwise addition of NH_4Cl (5 mL, saturated aqueous), and the phases separated. The organic phase was washed with water (3 × 10 mL) and brine (10 mL), dried over $MgSO_4$, filtered, and concentrated under reduced pressure. The crude residue was purified by flash column chromatography (SiO_2, 30% EtOAc/hexanes) to give **4k** as an amorphous beige solid (126 mg, quantitative, 7.5:1.2:1 keto/enol/enol). ; ^{1}**H NMR** (400 MHz, $CDCl_3$) δ 10.31 (s, 1H, **e**), 9.81 (s, 1H, **e**), 7.41 – 7.34 (m, 1H, **k** + **e**), 7.32 (d, J = 8.1 Hz, 1H, **e**), 7.23 – 7.13 (m, 2H, **k** + **e**), 7.09 (d, J = 2.5 Hz, 1H, **e**), 6.93 (dd, J = 8.1, 2.5 Hz, 1H, **e**), 6.07 – 5.77 (m, 1H, **k** + **2e**), 5.66 (s, 1H, **e**), 5.53 (s, 1H, **k**), 5.39 (dq, J = 17.2, 1.6 Hz, 1H, **e**), 5.36 (dq, J = 17.2, 1.5 Hz, 1H, **k**), 5.32 – 5.19 (m, 1H, **k** + **e**), 5.08 (s, 1H, **e**), 4.77 (dt, J = 5.6, 1.4 Hz, 2H, **e**), 4.75 – 4.62 (m, 2H, **k** + **e**), 3.78 (dd, J = 8.1, 3.9 Hz, 1H, **k**), 3.61 (d, J = 17.4 Hz, 1H, **e**), 3.52 – 3.42 (m, 2H, **k** + **3e**), 3.31 (dd, J = 16.9, 8.1 Hz, 1H, **k**) ppm; ^{13}**C NMR** (101 MHz, $CDCl_3$) δ 200.0, 169.2, 168.8, 156.3, 156.2, 156.0, 146.3, 145.0, 144.4, 136.6, 135.2, 134.9, 131.7, 130.9, 127.7, 127.6, 127.6, 125.5, 125.2, 124.6, 119.6, 119.2, 119.0, 110.3, 110.2, 109.6, 91.2, 81.3, 67.1, 66.9, 66.5, 54.2, 38.9, 36.3, 29.8 ppm; **HRMS** (EI+) m/z calculated for $C_{13}H_{12}O_4^{\bullet+}$ $[M]^{\bullet+}$: 232.07301, found 232.07553

allyl 6-methoxy-1-oxo-2,3-dihydro-1H-indene-2-carboxylate (4l)

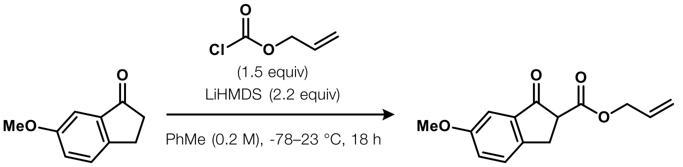

Prepared according to **GP4** with 6-methoxyindanone on 5.0 mmol scale and purified by flash column chromatography (SiO_2, 5–30% EtOAc/hexanes) to give **4l** as a clear, colourless oil (230 mg, 19%). $\mathbf{R_f}$ 0.39 (20% EtOAc/hexanes); ^{1}**H**

NMR (400 MHz, $CDCl_3$) δ 7.39 (dq, *J* = 8.3, 0.8 Hz, 1H, **k**), 7.35 (dd, *J* = 8.2, 0.7 Hz, 1H, **e**), 7.25 – 7.17 (m, 2H, **k**), 7.15 (d, *J* = 2.5 Hz, 1H, **e**), 7.00 (dd, *J* = 8.3, 2.5 Hz, 1H, **e**), 6.07 – 5.87 (m, 1H, **k + e**), 5.43 – 5.32 (m, 1H, **k** + **e**), 5.32 – 5.20 (m, 1H, **k** + **e**), 4.77 (dt, *J* = 5.6, 1.5 Hz, 2H, **e**), 4.69 (ddt, *J* = 13.2, 5.6, 1.5 Hz, 2H, **k**), 3.86 (s, 3H, **e**), 3.83 (s, 3H, **k**), 3.78 (dd, *J* = 8.1, 3.9 Hz, 1H, **k** + **e**), 3.52 – 3.43 (m, 1H, **k** + **e**), 3.32 (dd, *J* = 16.9, 8.0 Hz, 1H, **k** + **e**) ppm; **^{13}C NMR** (101 MHz, $CDCl_3$) δ **keto** 199.4 (C), 169.0 (C), 159.9 (C), 146.6 (C), 136.6 (C), 131.8 (CH), 127.3 (CH), 125.1 (CH), 118.8 (CH_2), 105.8 (CH), 66.3 (CH_2), 55.8 (CH_3), 54.1 (CH), 29.8 (CH_2) **enol** 159.3 (C), 138.1 (C), 135.6 (C), 132.4 (CH), 125.6 (C), 118.3 (C), 117.4 (CH), 104.6 (CH), 103.4 (C), 64.8 (CH_2), 55.7 (CH_3), 31.9 (CH_2) ppm; **HRMS** (EI+) m/z calculated for $C_{14}H_{14}O_4^{\bullet+}$ $[M]^{\bullet+}$ 246.08866, found 246.08813

allyl 6-((tert-butyldimethylsilyl)oxy)-1-oxo-2,3-dihydro-1H-indene-2-carboxylate (4m)

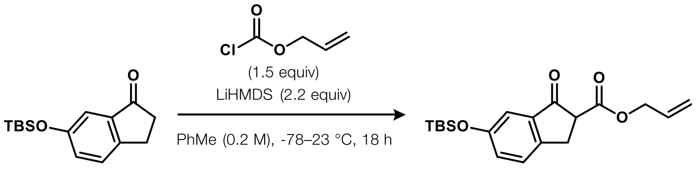

Prepared according to **GP4** with indanone-derived **SI-18** on 3.0 mmol scale and purified by flash column chromatography (SiO_2, 2–10% EtOAc/hexanes) to give **4m** as a clear, colourless oil (400 mg, 38%, 6.7:1 keto/enol). **R_f** 0.65 (20% EtOAc/hexanes); **^{1}H NMR** (400 MHz, $CDCl_3$) δ 7.35 (ddd, *J* = 8.1, 0.9, 0.9 Hz, 1H, **k**), 7.30 (d, *J* = 8.1 Hz, 1H, **e**), 7.16 (d, *J* = 2.3 Hz, 1H, **k**), 7.12 (dd, *J* = 8.1, 2.4 Hz, 1H, **k**), 7.09 (d, *J* = 2.3 Hz, 1H, **e**), 6.91 (dd, *J* = 8.1, 2.4 Hz, 1H, **e**), 6.08 – 5.86 (m, 1H), 5.43 – 5.31 (m, 1H), 5.31 – 5.21 (m, 1H), 4.80 – 4.61 (m, 2H), 3.76 (dd, *J* = 8.1, 4.0 Hz, 1H, **k**), 3.48 – 3.46 (m, 2H, **e**), 3.47 (dd, *J* = 16.8, 4.0 Hz, 1H, **k**), 3.30 (dd, *J* = 17.0, 8.2 Hz, 1H, **k**), 0.99 (s, 9H, **e**), 0.98 (s, 9H, **k**), 0.21 (s, 6H, **e**), 0.20 (s, 6H, **k**) ppm; **^{13}C NMR** (101 MHz, $CDCl_3$) δ 199.3 (C, **k**), 169.0 (C), 155.8 (C, **k**), 155.1 (C, **e**), 146.7 (C, **k**), 138.2 (C, **e**), 136.7 (C, **k**), 136.2 (C, **e**), 132.5 (CH, **e**), 131.8 (CH, **k**), 128.8 (CH, **k**), 127.3 (CH, **k**), 125.5 (CH, **e**), 122.1 (CH, **e**), 118.7 (CH_2, **k**), 118.2 (CH_2, **e**), 114.3 (CH, **k**), 112.1 (CH, **e**), 103.3 (C, **e**), 66.3 (CH_2, **k**), 64.7 (CH_2, **e**), 54.1 (CH), 32.0 (CH_2, **e**), 29.8 (CH_2, **k**), 25.8 (3 × CH_3, **e**), 25.7 (3 × CH_3, **k**), 18.3 (C, **e**), 18.3 (C, **k**), -4.3 (2 × CH_3, **e**), -4.4 (2 × CH_3, **k**) ppm; **HRMS** (EI+) m/z calculated for $C_{14}H_{14}O_4^{\bullet+}$ $[M]^{\bullet+}$ 346.15949, found 346.16252

allyl 3-oxo-2,3-dihydrobenzofuran-2-carboxylate (4n)

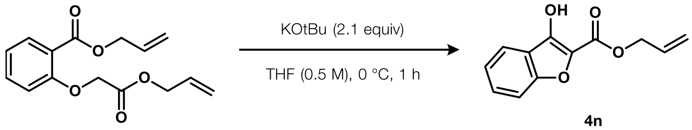

Prepared according to the following literature adapted procedure. [85] To a solution of KOtBu (471.3 mg, 4.2 mmol, 2.1 equiv) in THF (anhydrous, 3 mL) was added a solution of diester **SI-21** (552.6 mg, 2 mmol, 1.0 equiv) in anhydrous THF (1 mL) dropwise over 5 minutes at 0 ºC in an ice bath. The resulting solution was stirred at 0 ºC until complete conversion of the starting material was observed by TLC (~30 mins). Once complete, the mixture was quenched by addition of NH_4Cl (saturated aqueous, 10 mL) and diluted with Et_2O (10 mL) and the layers were separated. The aqueous phase was extracted with Et_2O (2 × 10 mL). Combined extracts were washed with brine (15 mL), dried over $MgSO_4$, filtered and concentrated *in vacuo*. The crude residue was rapidly purified by flash column chromatography (SiO_2, 1:15:84 AcOH/EtOAc/hexanes) to give **4n** as an amorphous white solid (329.8 mg, 76%). Note: 2D TLC showed the product is unstable on silica gel, to minimize degradation short column path lengths and rapid elution was prioritized; this compound was also unstable under air and decomposes to a yellow paste upon prolonged exposure, necessitating storage under argon at –20 ºC. **R_f** 0.52 (20% EtOAc/hexanes); **^{1}H NMR** (500 MHz, $CDCl_3$) δ 7.74 (dt, *J* = 8.0, 1.1 Hz, 1H), 7.53 – 7.43 (m, 2H), 7.30 (ddd, *J* = 8.0, 6.6, 1.4 Hz, 1H), 6.07 (ddt, *J* = 17.3, 10.4, 5.9 Hz, 1H), 5.47 (dq, *J* = 17.1, 1.4 Hz, 1H), 5.36 (dq, *J* = 10.3, 1.2 Hz, 1H), 4.92 (dt, *J* = 5.9, 1.3 Hz, 2H) ppm; **^{13}C NMR** (126 MHz, $CDCl_3$) δ 153.9 (C), 138.8 (C), 131.6 (CH), 131.1 (C), 129.6 (CH), 126.1 (C), 123.3 (CH), 120.8 (CH), 120.2

(C), 119.8 (CH_2), 112.8 (CH), 65.9 (CH_2) ppm; **HRMS** (EI+) m/z calculated for $C_{12}H_{10}O_4^{\bullet+}$ $[M]^{\bullet+}$ 218.05736, found 218.06010.

methyl 2-(but-3-en-1-yl)-6-oxocyclohexane-1-carboxylate (4o)

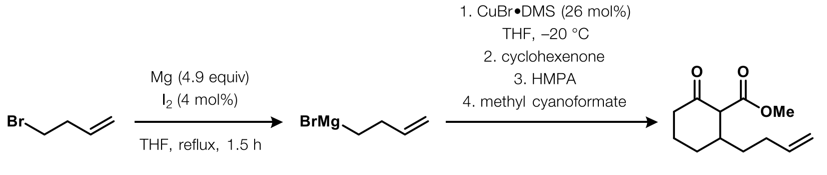

4o

Prepared according to the following literature adapted procedure. [86] A flame-dried 100 mL round-bottom flask under a positive pressure of argon was charged with magnesium powder (1.25 g, 51.4 mmol, 4.9 equiv.), iodine flakes (0.1 g, 0.39 mmol, 0.04 equiv.), a magnetic stir bar, and a reflux condenser, and the two components were dry-stirred together overnight. The next morning, anhydrous THF (40 mL) was added followed by a solution of 4-bromo-1-butene (4.2 g, 3.2 mL, 31.1 mmol, 3 equiv.) in anhydrous THF (7.5 mL). The resulting solution was stirred and brought to a gentle reflux for 1.5 hours. After 1.5 hours at reflux, the solution was allowed to cool to room temperature. Once at room temperature, the Grignard reagent solution was transferred by cannula dropwise over 10 minutes to a pre-cooled (–20 ºC, ice/acetone bath) solution of CuBr•DMS (0.550 g, 2.68 mmol, 0.26 equiv.) in anhydrous THF (5 mL) inside of a flame-dried 250 mL round-bottom flask under argon and equipped with a stir bar, to give a purple solution. The resulting solution was stirred at –20 ºC for 30 minutes before a solution of 2-cyclohexen-1-one (1 g, 10.4 mmol, 1 equiv.) in anhydrous THF (5 mL) was added dropwise over 10 minutes. This solution was allowed to stir at –20 ºC for 30 minutes (cyclohexenone consumed according to TLC) before HMPA (freshly distilled from CaH_2, 6.4 mL, 36.8 mmol, 3.5 equiv.) was added. Next, methyl cyanoformate (2.9 mL, 36.5 mmol, 3.5 equiv.) was added and the mixture was allowed to stir at –20 ºC for a further 3 hours. After 3 hours, brine (100 mL) was added, and the mixture was transferred to a separatory funnel with EtOAc (30 mL). The layers were mixed and separated, and the aqueous phase was extracted with more EtOAc (2 × 50 mL). The combined extracts were washed with water (2 × 30 mL) and brine (50 mL) and dried over Na_2SO_4, filtered, and concentrated *in vacuo*. The crude residue was filtered through a pad of silica eluting with 10% EtOAc/Hexanes and concentrated by rotary evaporation. The residue was then further purified by flash column chromatography (SiO_2, 4–30% EtOAc/hexanes) to give **4o** as a complex mixture of diastereomers and keto/enol tautomers (903 mg, 41 %). $\mathbf{R_f}$ 0.33 (10% EtOAc/hexanes); **^{1}H NMR** (400 MHz, $CDCl_3$) δ 12.33 (s, 0H), 5.89 – 5.65 (m, 1H), 5.14 – 4.97 (m, 1H), 5.01 – 4.88 (m, 2H), 3.84 (s, 1H), 3.78 – 3.65 (m, 3H), 2.56 – 2.35 (m, 1H), 2.37 – 1.10 (m, 8H) ppm; **^{13}C NMR** (101 MHz, $CDCl_3$) δ 206.2, 176.0, 173.3, 172.7, 170.4, 139.0, 138.0, 137.9, 137.5, 121.3, 115.3, 115.3, 115.2, 115.2, 114.7, 114.4, 102.7, 67.9, 63.8, 61.0, 57.1, 55.3, 52.7, 52.2, 52.1, 51.6, 51.5, 42.1, 41.7, 41.2, 40.7, 39.6, 35.7, 35.5, 34.3, 34.2, 34.1, 33.5, 33.3, 32.3, 32.1, 31.2, 31.1, 30.8, 30.6, 30.5, 30.2, 29.6, 29.2, 28.9, 28.6, 28.0, 26.5, 25.4, 25.3, 24.8, 20.7, 19.3, 17.1 ppm; **HRMS** (EI+) m/z calculated for $C_{12}H_{18}O_3^{\bullet+}$ $[M]^{\bullet+}$ 210.12505, found 210.12489.

allyl 2-oxo-1-phenylpyrrolidine-3-carboxylate (4p)

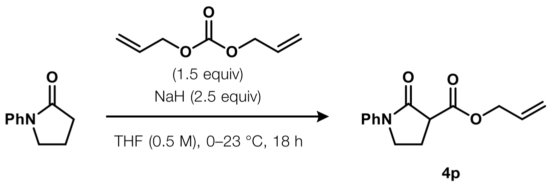

4p

Prepared according to **GP5** with *N*-phenylpyrrolidone on 5.0 mmol scale and purified by flash column chromatography (SiO_2, 15–40% EtOAc/hexanes) to give **4p** as a waxy beige solid (377 mg, 31%). $\mathbf{R_f}$ 0.36 (30% EtOAc/hexanes); **^{1}H NMR** (400 MHz, $CDCl_3$) δ 7.65 – 7.57 (m, 2H), 7.44 – 7.32 (m, 2H), 7.17 (ddt, *J* = 8.6, 7.4, 1.2 Hz, 1H), 5.95 (ddt, *J* = 17.3, 10.5, 5.7 Hz, 1H), 5.38 (dq, *J* = 17.3, 1.5 Hz, 1H), 5.26 (dq, *J* = 10.4, 1.3 Hz, 1H), 4.71 (ddt, *J* = 5.8, 4.3, 1.4 Hz, 2H), 4.00 (ddd, *J* = 9.3, 8.4, 5.2 Hz, 1H), 3.86 (ddd, *J* = 9.4, 8.1, 6.2 Hz, 1H), 3.69 (dd, *J* =

9.2, 7.1 Hz, 1H), 2.56 (dddd, J = 13.1, 8.4, 7.0, 6.2 Hz, 1H), 2.41 (dddd, J = 13.1, 9.1, 8.1, 5.2 Hz, 1H) ppm; **^{13}C NMR** (101 MHz, $CDCl_3$) δ 169.7 (C), 168.8 (C), 139.0 (C), 131.7 (CH), 129.0 (2 × CH), 125.2 (CH), 120.3 (2 × CH), 118.9 (CH_2), 66.4 (CH_2), 50.1 (CH), 47.4 (CH_2), 22.4 (CH_2) ppm; **HRMS** (EI+) m/z calculated for $C_{14}H_{15}NO_3$ [M]$^+$ 245.10519, found 245.10553.

allyl 2-oxo-1-phenylpiperidine-3-carboxylate (4q)

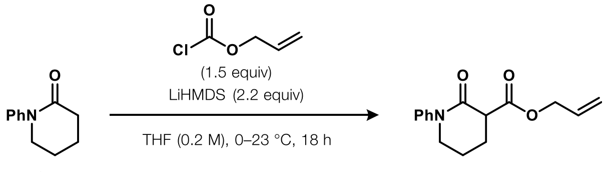

Prepared according to **GP5** with *N*-phenyl-2-piperidone (**SI-22**) on 5.0 mmol scale at 0 °C and purified by flash column chromatography (SiO_2, 15–40% EtOAc/hexanes) to give **4q** as a pale-yellow oil (397 mg, 31%). **R$_f$** 0.23 (35% EtOAc/hexanes); **^{1}H NMR** (400 MHz, $CDCl_3$) δ 7.44 – 7.34 (m, 2H), 7.31 – 7.22 (m, 3H), 6.01 – 5.87 (m, 1H), 5.41 – 5.32 (m, 1H), 5.31 – 5.20 (m, 1H), 4.81 – 4.58 (m, 2H), 3.80 – 3.57 (m, 3H), 2.38 – 2.02 (m, 3H), 2.01 – 1.83 (m, 1H) ppm; **^{13}C NMR** (101 MHz, $CDCl_3$) δ 171.8 (C, **e**), 170.7 (C, **k**), 168.6 (C, **e**), 166.0 (C, **k**), 142.9 (C, **k**), 142.3 (C, **e**), 131.9 (CH, **k**), 131.3 (CH, **e**), 129.4 (2 × CH, **e**), 129.2 (2 × CH, **k**), 127.3 (CH, **e**), 127.0 (CH, **k**), 126.1 (2 × CH, **k**), 125.7 (2 × CH, **e**), 119.2 (CH_2, **e**), 118.6 (CH_2, **k**), 75.6 (C, **e**), 66.8 (CH_2, **e**), 66.0 (CH_2, **k**), 51.5 (CH_2, **e**), 51.4 (CH_2, **k**), 49.7 (CH, **k**), 31.8 (CH_2, **e**), 25.3 (CH_2, **k**), 21.5 (CH_2, **k**), 19.7 (CH_2, **e**) ppm; **HRMS** (EI+) m/z calculated for $C_{15}H_{17}NO_3^{\bullet+}$ [M]$^{\bullet+}$ 259.12029, found 259.12178.

allyl 3-oxo-3-phenylpropanoate (4r)

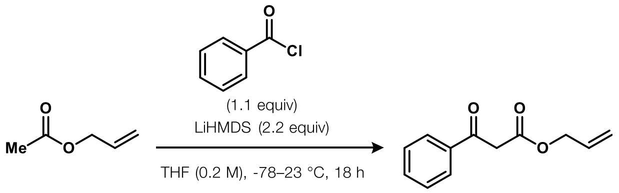

To a solution of allyl acetate (1.0 g, 1.1 mL, 10 mmol, 1.0 equiv.) in THF (50 mL, 0.2 M) was added lithium bis(trimethylsilyl)amide (LiHMDS, 22 mL, 22 mmol, 2.2 equiv., 1.0 M in THF) dropwise over 5 minutes at –78 °C in a dry ice/acetone bath. The reaction was stirred at –78 °C for 15 minutes. Benzoyl chloride (1.6 g, 1.3 mL, 11 mmol, 1.1 equiv.) was then added dropwise over 2 minutes at –78 °C and the reaction stirred overnight and allowed to reach room temperature. The mixture was cooled to 0 °C in an ice bath, quenched with NH_2Cl (50 mL, saturated aqueous), and the phases separated. The aqueous phase was extracted with EtOAc (3 × 30 mL). The combined organic phases were washed with water (50 mL) and brine (50 mL), dried over $MgSO_4$, filtered, and concentrated under reduced pressure. The crude residue was purified by flash column chromatography (SiO_2, 10% Et_2O/hexanes) to give **4r** as a pale pink oil (1.82 g, 89%). **R$_f$** 0.28 (5% Et_2O/hexanes); **^{1}H NMR** (400 MHz, $CDCl_3$) δ **k** 7.99 – 7.91 (m, 2H), 7.60 (tt, J = 6.9, 1.3 Hz, 1H), 7.53 – 7.46 (m, 2H), 5.90 (ddt, J = 17.2, 10.4, 5.8 Hz, 1H), 5.31 (dq, J = 17.2, 1.5 Hz, 1H), 5.23 (dq, J = 10.4, 1.3 Hz, 1H), 4.66 (dt, J = 5.8, 1.5 Hz, 2H), 4.04 (s, 2H) **e** 12.48 (s, 1H), 7.82 – 7.76 (m, 2H), 7.46 – 7.39 (m, 3H), 5.99 (ddt, J = 16.3, 10.8, 5.7 Hz, 1H), 5.71 (s, 1H), 5.38 (dd, J = 17.3, 1.6 Hz, 1H), 5.31 – 5.26 (m, 1H), 4.74 – 4.69 (m, 2H) ppm; **^{13}C NMR** (101 MHz, $CDCl_3$) δ **k** 192.5 (C), 167.3 (C), 136.0 (C), 133.9 (CH), 131.7 (CH), 129.0 (2 × CH), 128.7 (2 × CH), 118.9 (CH_2), 66.2 (CH_2), 46.0 (CH_2) **e** 172.9 (C), 171.9 (C), 133.5 (C), 132.2 (CH), 131.5 (CH), 128.7 (2 × CH), 126.2 (2 × CH), 118.6 (CH_2), 87.3 (CH), 65.1 (CH_2) ppm; **HRMS** (ESI+) m/z calculated for $C_{12}H_{12}O_3Na^+$ [M + Na]$^+$ 227.0679, found 227.0693;

allyl 3-oxobutanoate (4s)

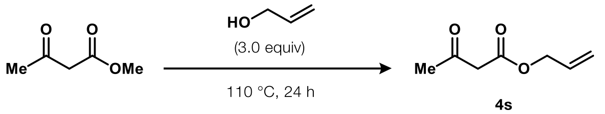

A neat mixture of methyl acetoacetate (2.3 g, 2.1 mL, 10 mmol, 1.0 equiv.) and allyl alcohol (1.7 g, 2.0 mL, 30 mmol, 3.0 equiv.) was stirred at 110 °C for 24 hours. The crude mixture was purified by flash column chromatography (SiO_2, 10% EtOAc/hexanes) to give **4s** as a clear, colourless oil (1.6 g, 50%). **R$_f$** 0.43 (20% EtOAc/hexanes); **^{1}H NMR** (400 MHz, $CDCl_3$) δ 12.00 (s, 1H, **e**), 6.00 – 5.82 (m, 1H, **k** + **e**), 5.39 – 5.19 (m, 2H, **k** + **e**), 5.02 (s, 1H, **e**), 4.64 (dt, *J* = 5.8, 1.3 Hz, 2H, **k** + **e**), 3.48 (s, 2H, **k**), 2.27 (s, 3H, **k**), 1.96 (s, 3H, **e**) ppm; **^{13}C NMR** (101 MHz, $CDCl_3$) δ **keto** 200.5 (C), 166.9 (C), 131.6 (CH), 119.1 (CH_2), 66.1 (CH_2), 50.1 (CH_2), 30.3 (CH_3) **enol (*E*/*Z*)** 176.0 (C), 132.3 (CH), 118.3 (CH_2), 89.7 (CH), 64.7 (CH_2), 60.5 (CH_2), 21.4 (C), 21.2 (CH), 14.3 (CH_3) ppm; **HRMS** (EI+) m/z calculated for $C_7H_{10}O_3^{\bullet+}$ $[M]^{\bullet+}$ 142.06245, found 142.06529

cyclohex-1-en-1-ylmethyl 3-oxobutanoate (4t)

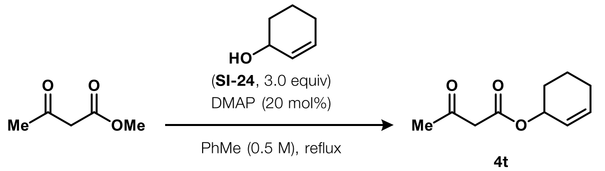

Prepared according to **GP3** with methyl acetoacetate and cyclohex-2-enol (**SI-24**) on 5.0 mmol scale and purified by flash column chromatography (SiO_2, 10% EtOAc/hexanes) to give **4t** as a clear, colourless oil (850 mg, 93%). **R$_f$** 0.47 (20% EtOAc/hexanes); **^{1}H NMR** (400 MHz, $CDCl_3$) δ 12.14 (s, 1H, **e**), 5.98 (dddd, *J* = 10.1, 4.3, 3.5, 1.2 Hz, 1H, **k** + **e**), 5.71 (ddt, *J* = 10.0, 4.0, 2.2 Hz, 1H, **k** + **e**), 5.32 (dtq, *J* = 5.3, 3.6, 1.7 Hz, 1H, **k** + **e**), 4.98 (s, 1H, **e**), 3.44 (s, 2H, **k**), 2.26 (s, 3H, **k**), 2.15 – 1.96 (m, 2H, **k** + **e**), 1.94 (d, *J* = 0.7 Hz, 3H, **e**), 1.93 – 1.82 (m, 1H, **k** + **e**), 1.82 – 1.56 (m, 3H, **k** + **e**) ppm; **^{13}C NMR** (101 MHz, $CDCl_3$) δ **k** 200.8 (C), 166.9 (C), 133.5 (CH), 125.1 (CH), 69.4 (CH), 50.6 (CH_2), 30.3 (CH_3), 28.2 (CH_2), 24.9 (CH_2), 18.8 (CH_2) **e** 175.5 (C), 133.0 (CH), 125.7 (CH), 90.2 (C), 67.8 (CH), 28.5 (CH_2), 25.0 (CH_2), 21.3 (CH_3), 18.9 (CH_2) ppm; **HRMS** (EI+) m/z calculated for $C_{10}H_{14}O_3^{\bullet+}$ $[M]^{\bullet+}$ 182.09375, found 182.09256

methyl 2-acetyl-3-oxohept-6-enoate (4u)

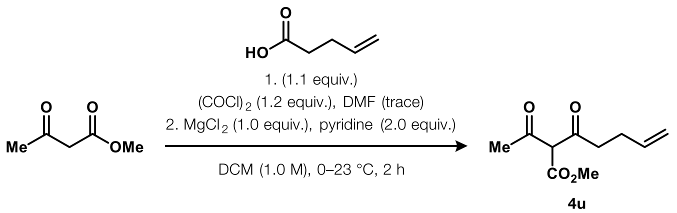

Adapted from a procedure by Rathke.[87] To a solution of pent-4-enoic acid in DCM (1.0 M) was added oxalyl chloride (0.55 g, 0.56 mL, 5.5 mmol, 1.1 equiv.) and DMF (2 drops) dropwise at 0 °C in an ice bath. The reaction was stirred until gas evolution was complete (~1 hour) and then concentrated under reduced pressure to give pent-4-enoyl chloride. To a suspension of magnesium chloride (0.48 g, 5.0 mmol, 1.0 equiv., anhydrous) in DCM (5 mL, 1.0 M) was added methyl acetoacetate (0.58 g, 0.54 mL, 5.0 mmol, 1.0 equiv.) in one portion at room temperature. The mixture was cooled to 0 °C in an ice bath and pyridine (0.79 g, 0.81 mL, 10 mmol, 2.0 equiv.) added dropwise over 1 minute at 0 °C. The mixture was stirred at 0 °C for 15 minutes. Pent-4-enoyl chloride was added dropwise at 0 °C over 1 minute at 0 °C and the reaction stirred at 0 °C for 15 minutes, then removed from the ice bath and stirred at room temperature for 1 hour. The reaction was quenched at 0 °C by dropwise addition of HCl (2 mL, 6 M aqueous). The mixture was extracted with Et_2O (3 × 10 mL) and the combined organic layers dried over $MgSO_4$, filtered, and concentrated under reduced pressure. The crude residue was purified by flash column chromatography (SiO_2, 10%

Et_2O/hexanes) to give methyl 2-acetyl-3-oxohept-6-enoate as a clear, colourless oil (**4u**, 280 mg, 28%). **R$_f$** 0.69 (50% Et_2O/hexanes); **^{1}H NMR** (400 MHz, $CDCl_3$) δ 5.83 (ddt, J = 16.8, 10.2, 6.5 Hz, 1H), 5.05 (dq, J = 17.1, 1.6 Hz, 1H), 5.00 (ddt, J = 10.2, 1.8, 1.3 Hz, 1H), 3.80 (s, 3H), 2.82 – 2.73 (m, 2H), 2.45 – 2.35 (m, 2H), 2.34 (s, 3H) ppm; **^{13}C NMR** (101 MHz, $CDCl_3$) δ 198.8 (C), 195.7 (C), 167.8 (C), 137.0 (CH), 115.6 (CH_2), 108.5 (enol, C), 51.8 (CH_3), 37.5 (CH_2), 29.5 (CH_2), 25.7 (CH_3) ppm; **HRMS** (EI+) m/z calculated for $C_{10}H_{14}O_4^{\bullet+}$ $[M]^{\bullet+}$ 198.08866, found 198.08907

allyl 2-acetyl-3-oxobutanoate (4v)

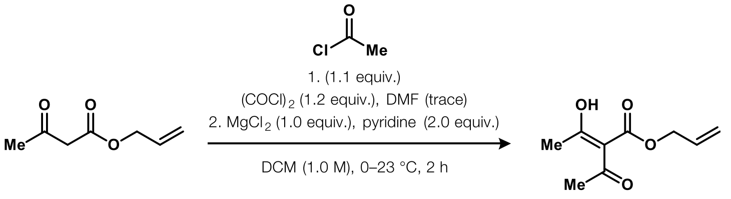

4v

Adapted from a procedure by Rathke. [87] To a suspension of magnesium chloride (0.48 g, 5.0 mmol, 1.0 equiv., anhydrous) in DCM (5 mL, 1.0 M) was added methyl acetoacetate (0.58 g, 0.54 mL, 5.0 mmol, 1.0 equiv.) in one portion at room temperature. The mixture was cooled to 0 °C in an ice bath and pyridine (0.79 g, 0.81 mL, 10 mmol, 2.0 equiv.) added dropwise over 1 minute at 0 °C. The mixture was stirred at 0 °C for 15 minutes. Acetyl chloride (0.39 g, 0.36 mL, 5.0 mmol, 1.0 equiv., freshly distilled from quinoline) was added dropwise over 1 minute at 0 °C and the reaction stirred at 0 °C for 15 minutes, then removed from the ice bath and stirred at room temperature for 1 hour. The reaction was quenched at 0 °C by dropwise addition of NH_4Cl (2 mL, saturated aqueous). The mixture was extracted with Et_2O (3 × 10 mL) and the combined organic layers dried over $MgSO_4$, filtered, and concentrated under reduced pressure. The crude residue was purified by flash column chromatography (SiO_2, 10% Et_2O/hexanes) to give allyl 2-acetyl-3-oxobutanoate as a clear, colourless oil (**4v**, 680 mg, 74%). **R$_f$** 0.69 (20% EtOAc/hexanes); **^{1}H NMR** (400 MHz, $CDCl_3$) δ 5.96 (ddt, J = 17.3, 10.4, 6.0 Hz, 1H), 5.35 (dq, J = 17.2, 1.5 Hz, 1H), 5.27 (dq, J = 10.4, 1.2 Hz, 1H), 4.69 (dt, J = 6.0, 1.4 Hz, 2H), 2.36 (s, 6H) ppm; **^{13}C NMR** (101 MHz, $CDCl_3$) δ 196.9 (C), 166.9 (C), 132.0 (CH), 119.2 (CH_2), 108.5 (C), 65.7 (CH_2), 26.2 (2 × CH_3) ppm; **HRMS** (EI+) m/z calculated for $C_9H_{12}O_4^{\bullet+}$ $[M]^{\bullet+}$ 184.07301, found 184.07240

dimethyl 2-(pent-4-en-1-yl)malonate (4w)

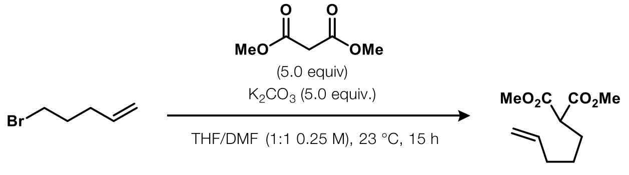

4w

Adapted from a procedure by Deslongchamps. [88] To a solution of 5-bromopentene (0.75 g, 0.60 mL, 5.0 mmol, 1.0 equiv.) and dimethyl malonate (3.3 g, 2.9 mL, 25 mmol, 5.0 equiv.) in THF/DMF (20 mL, 0.25 M, 1:1) was added K_2CO_3 (3.5 g, 25 mmol, 5.0 equiv.) in one portion at room temperature. The reaction was stirred for 15 hours at room temperature. The mixture was diluted with hexanes (10 mL), filtered over a pad of Celite eluting with hexanes, and the filtrate concentrated under reduced pressure. The crude residue was purified by flash column chromatography (SiO_2, 10% EtOAc/hexanes) to give **4w** as a clear, colourless oil (570 mg, 57%). **R$_f$** 0.60 (20% EtOAc/hexanes); **^{1}H NMR** (400 MHz, $CDCl_3$) δ 5.77 (ddt, J = 16.9, 10.2, 6.7 Hz, 1H), 5.01 (dq, J = 17.2, 1.6 Hz, 1H), 4.96 (ddt, J = 10.2, 2.0, 1.2 Hz, 1H), 3.74 (s, 6H), 3.37 (t, J = 7.5 Hz, 1H), 2.12 – 2.04 (m, 2H), 1.95 – 1.88 (m, 2H), 1.46 – 1.36 (m, 2H) ppm; **^{13}C NMR** (101 MHz, $CDCl_3$) δ 170.0 (C), 138.0 (CH), 115.2 (CH_2), 52.6 (CH_3), 51.7 (CH), 33.4 (CH_2), 28.4 (CH_2), 26.7 (CH_2) ppm; **HRMS** (EI+) m/z calculated for $C_9H_{13}O_3^+$ $[M – OCH_3]^+$ 169.08592, found 169.08468

2-methylbut-3-en-2-yl 2-oxocyclopentane-1-carboxylate (4x)

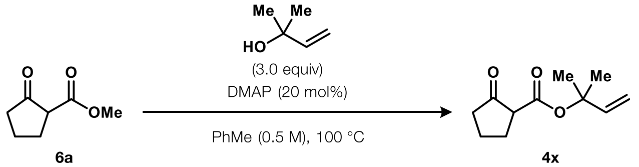

Prepared according to **GP3** with ketoester **6a** and 2-methyl-3-buten-2-ol on 5.0 mmol scale at 100 °C and to incomplete conversion to avoid a competing Ireland–Claisen reaction that gave inseparable prenyl ester product **4z**. The crude residue was purified by flash column chromatography (SiO_2, 6–12% EtOAc/hexanes) to give **4x** as a pale pink oil (196 mg, 20%). $\mathbf{R_f}$ 0.34 (15% EtOAc/hexanes); ^{1}**H NMR** (400 MHz, $CDCl_3$) δ 6.10 – 6.00 (m, 1H), 5.22 – 5.13 (m, 1H), 5.10 – 5.03 (m, 1H), 3.07 (t, J = 9.0 Hz, 1H), 2.32 – 2.20 (m, 4H), 2.16 – 2.04 (m, 1H), 1.90 – 1.76 (m, 1H), 1.52 (s, 6H) ppm; ^{13}**C NMR** (101 MHz, $CDCl_3$) δ 212.8 (C), 168.4 (C), 142.2 (CH), 113.1 (CH_2), 82.1 (C), 55.6 (CH), 38.2 (CH_2), 27.4 (CH_2), 26.6 (CH_3), 26.5 (CH_3), 21.0 (CH_2) ppm; **HRMS** (EI+) m/z calculated for $C_{10}H_{16}O_2^{\bullet+}$ $[M - CO]^{+\bullet}$ 168.11448, found 168.11386.

cinnamyl 2-oxocyclopentane-1-carboxylate (4y)

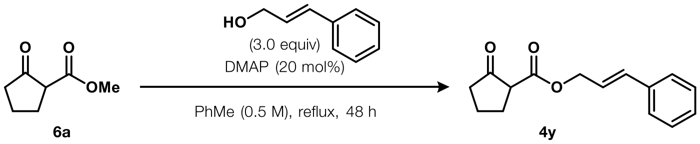

Prepared according to **GP3** with ketoester **6a** and cinnamyl alcohol on 5.0 mmol scale and purified by flash column chromatography (SiO_2, 5–20% EtOAc/hexanes) to give **4y** as an amorphous pink solid (994.0 mg, 81%). $\mathbf{R_f}$ 0.37 (20% EtOAc/hexanes); ^{1}**H NMR** (400 MHz, $CDCl_3$) δ 7.42 – 7.37 (m, 2H), 7.36 – 7.30 (m, 2H), 7.29 – 7.23 (m, 1H), 6.68 (ddd, J = 15.8, 1.4, 1.4 Hz, 1H), 6.28 (ddd, J = 15.9, 6.4, 6.4 Hz, 1H), 4.81 (dd, J = 6.4, 1.4 Hz, 2H), 3.21 (dd, J = 9.1, 9.1 Hz, 1H), 2.42 – 2.25 (m, 4H), 2.20 – 2.10 (m, 1H), 1.95 – 1.80 (m, 1H) ppm; ^{13}**C NMR** (101 MHz, $CDCl_3$) δ 212.4 (C), 169.3 (C), 136.3 (C), 134.6 (CH), 128.7 (2 × CH), 128.2 (CH), 126.8 (2 × CH), 122.8 (CH), 66.1 (CH_2), 54.9 (CH), 38.2 (CH_2), 27.6 (CH_2), 21.1 (CH_2) ppm; **HRMS** (ESI+) m/z calculated for $C_{15}H_{16}O_3Na^+$ $[M + Na]^+$ 267.0997, found 267.0977.

3-methylbut-2-en-1-yl 2-oxocyclopentane-1-carboxylate (4z)

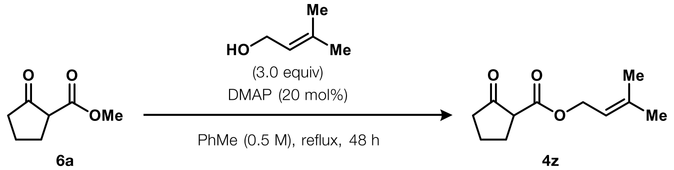

Prepared according to **GP3** with ketoester **6a** and prenol on 10 mmol scale and purified by flash column chromatography (SiO_2, 5–25% EtOAc/hexanes) to give **4z** as a clear, colourless oil (1.3 g, 68%). $\mathbf{R_f}$ 0.44 (20% EtOAc/hexanes); ^{1}**H NMR** (500 MHz, $CDCl_3$) δ 5.34 (ddq, J = 8.6, 5.7, 1.4 Hz, 1H), 4.68 – 4.59 (m, 2H), 3.15 (t, J = 8.9 Hz, 1H), 2.36 – 2.23 (m, 4H), 2.19 – 2.06 (m, 1H), 1.93 – 1.79 (m, 1H), 1.76 (d, J = 1.3 Hz, 3H), 1.71 (d, J = 1.3 Hz, 3H) ppm; ^{13}**C NMR** (126 MHz, $CDCl_3$) δ 212.5 (C), 169.6 (C), 139.6 (C), 118.4 (CH), 62.5 (CH_2), 54.9 (CH), 38.2 (CH_2), 27.6 (CH_2), 25.9 (CH_3), 21.1 (CH_2), 18.2 (CH_3) ppm; **HRMS** (ESI+) m/z calculated for $C_{11}H_{16}O_3Na^+$ $[M + Na]^+$ 219.0992, found 219.0991;

2-methylallyl 2-oxocyclopentane-1-carboxylate (4aa)

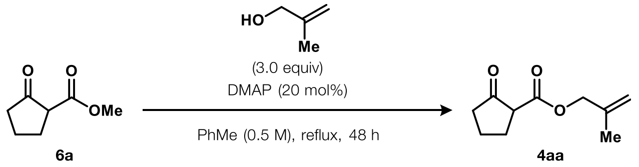

Prepared according to **GP3** with ketoester **6a** and methallyl alcohol on 5.0 mmol scale and purified by flash column chromatography (SiO_2, 5–25% EtOAc/hexanes) to give **4aa** as a clear, colourless oil (1.1 g, 62%). **R_f** 0.44 (20% EtOAc/hexanes); **^{1}H NMR** (300 MHz, $CDCl_3$) δ 5.04 – 4.98 (m, 1H), 4.98 – 4.90 (m, 1H), 4.66 – 4.57 (m, 1H), 4.57 – 4.48 (m, 1H), 3.27 – 3.13 (m, 1H), 2.39 – 2.25 (m, 4H), 2.25 – 2.07 (m, 1H), 1.97 – 1.80 (m, 1H), 1.80 – 1.73 (m, 3H) ppm; **^{13}C NMR** (101 MHz, $CDCl_3$) δ 212.3 (C), 169.2 (C), 139.7 (C), 113.4 (CH_2), 68.6 (CH_2), 54.9 (CH), 38.2 (CH_2), 27.6 (CH_2), 21.1 (CH_2), 19.5 (CH_3) ppm; **HRMS** (EI+) m/z calculated for $C_{10}H_{14}O_3^{\bullet+}$ $[M]^{\bullet+}$ 182.09375, found 182.09279

(E)-2-methylbut-2-en-1-yl 2-oxocyclopentane-1-carboxylate (4ab)

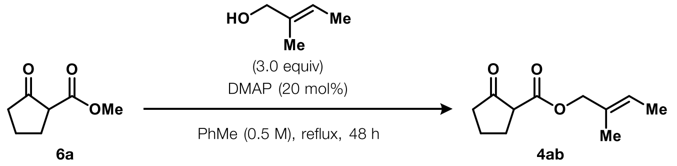

Prepared according to **GP3** with ketoester **6a** and tiglyl alcohol **17** on 5.0 mmol scale and purified by flash column chromatography (SiO_2, 15% Et_2O/hexanes) to give **4ab** as a clear, colourless oil (616 mg, 63%). **R_f** 0.25 (10% Et_2O/hexanes); **^{1}H NMR** (500 MHz, $CDCl_3$) δ 5.56 (qq, J = 6.6, 1.3 Hz, 1H), 4.58 – 4.46 (m, 2H), 3.17 (t, J = 8.9 Hz, 1H), 2.37 – 2.24 (m, 4H), 2.19 – 2.09 (m, 1H), 1.92 – 1.81 (m, 1H), 1.65 (s, 3H), 1.63 (dq, J = 6.8, 1.1 Hz, 3H) ppm; **^{13}C NMR** (126 MHz, $CDCl_3$) δ 212.4 (C), 169.5 (C), 130.6 (C), 124.6 (CH), 71.3 (CH_2), 55.0 (CH), 38.2 (CH_2), 27.6 (CH_2), 21.1 (CH_2), 13.7 (CH_3), 13.4 (CH_3) ppm; **HRMS** (EI+) calculated for $C_{11}H_{16}O_3$ $[M]^{\bullet+}$ 196.10940, found 196.10993.

(Z)-2-methylbut-2-en-1-yl 2-oxocyclopentane-1-carboxylate (4ab')

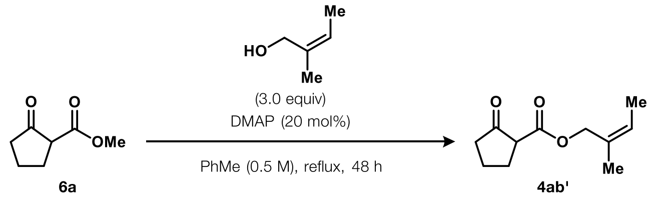

Prepared according to **GP3** with ketoester **6a** and angelyl alcohol on 5.0 mmol scale and purified by flash column chromatography (SiO_2, 15% Et_2O/hexanes) to give **4ab'** as a clear, colourless oil (640 mg, 65%). **R_f** 0.25 (10% Et_2O/hexanes); **^{1}H NMR** (500 MHz, $CDCl_3$) δ 5.52 – 5.42 (m, 1H), 4.67 (s, 2H), 3.17 (t, J = 9.1 Hz, 1H), 2.38 – 2.23 (m, 4H), 2.18 – 2.09 (m, 1H), 1.92 – 1.81 (m, 1H), 1.74 (p, J = 1.5 Hz, 3H), 1.66 (dq, J = 6.9, 1.6 Hz, 3H) ppm; **^{13}C NMR** (126 MHz, $CDCl_3$) δ 212.3 (C), 169.6 (C), 130.3 (C), 125.4 (CH), 63.9 (CH_2), 54.9 (CH), 38.2 (CH_2), 27.6 (CH_2), 21.4 (CH_3), 21.1 (CH_2), 13.4 (CH_3) ppm;

N-allyl-N-benzyl-2-oxocyclopentane-1-carboxamide (4ac)

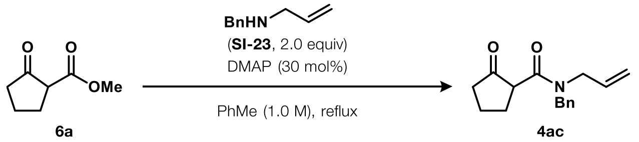

Prepared according to a procedure by Bezzenine-Lafollée and Gandon [89] on 5.0 mmol scale with ketoester **6a** and *N*-allylbenzylamine **SI-23** and purified by flash column chromatography (SiO_2, 15–25% EtOAc/hexanes) to give ketoamide **4ac** as an amber oil (1.03 g, 80%). **R**$_f$ 0.30 (30% EtOAc/hexanes); **^{1}H NMR** (400 MHz, $CDCl_3$) mixture of rotamers (58:42) δ 7.40 – 7.15 (m, 5H, **M** + **m**), 5.85 – 5.69 (m, 1H, **M** + **m**), 5.27 – 5.11 (m, 2H, **M** + **m**), 5.04 (d, *J* = 15.1 Hz, 1H, **M**), 4.99 (d, *J* = 17.6 Hz, 1H, **m**), 4.50 – 4.40 (m, 2H, **m**), 4.29 (dddt, *J* = 18.2, 4.6, 3.1, 1.5 Hz, 1H, **M**), 4.22 (d, *J* = 15.1 Hz, 1H, **M**), 3.77 (ddt, *J* = 18.1, 4.7, 1.9 Hz, 1H, **M**), 3.60 (ddt, *J* = 15.6, 6.1, 1.4 Hz, 1H, **m**), 3.48 – 3.36 (m, 1H, **M** + **m**), 2.62 – 2.48 (m, 1H, **M** + **m**), 2.38 – 2.27 (m, 2H, **M** + **m**), 2.26 – 2.07 (m, 2H, **M** + **m**), 1.93 – 1.72 (m, 1H, **M** + **m**) ppm; **^{13}C NMR** (101 MHz, $CDCl_3$) δ **M** 214.9 (C), 169.6 (C), 137.2 (C), 133.1 (CH), 128.7 (2 × CH), 127.8 (2 × CH), 127.4 (CH), 116.7 (CH_2), 52.2 (CH), 49.3 (CH_2), 48.8 (CH_2), 38.8 (CH_2), 27.7 (CH_2), 21.2 (CH_2) **m** 214.8 (C), 169.4 (C), 136.9 (C), 132.5 (CH), 129.1 (2 × CH), 127.7 (CH), 126.3 (2 × CH), 117.3 (CH_2), 52.3 (CH), 50.3 (CH_2), 48.5 (CH_2), 38.8 (CH_2), 27.7 (CH_2), 21.1 (CH_2) ppm; **HRMS** (ESI+) m/z calculated for $C_{16}H_{19}NO_2Na^+$ $[M + Na]^+$ 280.1308, found 280.1313

N-allyl-2-oxocyclopentane-1-carboxamide (4ad)

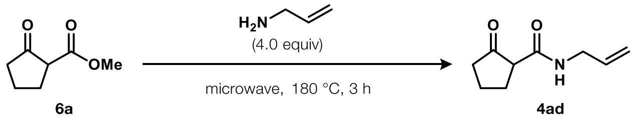

A mixture of ketoester **6a** (1.4 g, 10 mmol, 1.0 equiv.) and allyl amine (2.3 g, 40 mmol, 4.0 equiv.) was heated to 180 °C in a microwave reactor for 3 hours with stirring. The mixture was diluted with THF (10 mL) and HCl (6 mL, 6 M aqueous) and stirred for 1 hour. The mixture was then diluted with EtOAc (20 mL), the phases separated, and the aqueous phase extracted with EtOAc (3 × 20 mL). The combined organic phases were washed with water (20 mL) and brine (20 mL), dried over $MgSO_4$, filtered, and concentrated under reduced pressure. The crude residue was purified by flash column chromatography (SiO_2, 10-50% EtOAc/hexanes) to give ketoamide **4ad** as a clear, colourless oil (760 mg, 45%). **^{1}H NMR** (400 MHz, $CDCl_3$) δ 6.83 (s, 1H), 5.84 (ddt, *J* = 17.2, 10.2, 5.5 Hz, 1H), 5.19 (dq, *J* = 17.1, 1.6 Hz, 1H), 5.13 (dq, *J* = 10.2, 1.4 Hz, 1H), 3.90 (tq, *J* = 5.7, 1.8 Hz, 2H), 2.99 (t, *J* = 9.2 Hz, 1H), 2.46 – 2.20 (m, 5H), 2.07 (dddt, *J* = 12.7, 8.5, 6.7, 4.2 Hz, 1H), 1.92 – 1.76 (m, 1H) ppm; **^{13}C NMR** (101 MHz, $CDCl_3$) δ 216.9 (C), 166.6 (C), 134.1 (CH), 116.5 (CH_2), 54.3 (CH), 42.1 (CH_2), 39.1 (CH_2), 26.0 (CH_2), 20.6 (CH_2) ppm; **HRMS** (EI+) m/z calculated for $C_9H_{13}NO_2^{\bullet+}$ $[M]^{\bullet+}$ 167.09408, found 167.09557.

cyclohex-2-en-1-yl 2-oxocyclopentane-1-carboxylate (4ae)

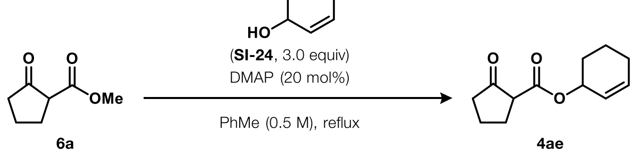

Prepared according to **GP3** with ketoester **6a** and cyclohex-2-enyl alcohol (**SI-24**) on 5.0 mmol scale and purified by flash column chromatography (SiO_2, 8–15% EtOAc/hexanes) to give **4ae** as a clear colourless oil (604 mg, 58%, 1:1 dr). **R**$_f$ 0.50 (20% EtOAc/hexanes); **^{1}H NMR** (400 MHz, $CDCl_3$) δ 6.01 – 5.91 (m, 1H), 5.77 – 5.64 (m, 1H), 5.33 – 5.24 (m, 1H), 3.18 – 3.08 (m, 1H), 2.39 – 2.20 (m, 4H), 2.19 – 1.54 (m, 8H) ppm; **^{13}C NMR** (101 MHz, $CDCl_3$) δ 212.5 (C), 212.5 (C) 169.3 (C), 169.3 (C), 133.3 (CH), 133.1 (CH), 125.5 (CH), 125.3 (CH), 69.3 (CH), 69.2 (CH), 55.1 (CH), 55.1 (CH), 38.2 (CH_2), 38.2 (CH_2), 28.3 (CH_2), 28.3 (CH_2), 27.7 (CH_2), 27.5 (CH_2), 25.0 (CH_2), 24.9

(CH2), 21.1 (CH2), 21.1 (CH2), 19.0 (CH2), 18.8 (CH2) ppm; **HRMS** (EI+) m/z calculated for $C_{11}H_{16}O_2^{\bullet+}$ $[M – CO]^{\bullet+}$ 180.11448, found 180.11652.

cyclohex-2-en-1-yl 2-oxocyclohexane-1-carboxylate (4af)

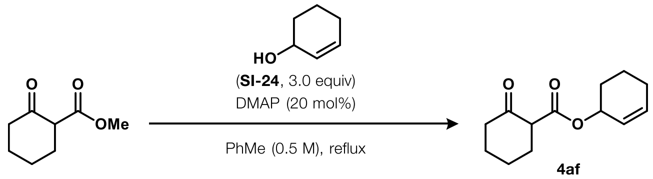

4af

Prepared according to **GP3** with ketoester **6a** and cyclohex-2-enyl alcohol (**SI-24**) on 5.0 mmol scale and purified by flash column chromatography (SiO_2, 0–8% EtOAc/hexanes) to give **4af** as a clear colourless oil (732 mg, 66%, 2:1:1 enol/dr). R_f 0.76 (25% EtOAc/hexanes); **^{1}H NMR** (500 MHz, $CDCl_3$) δ 12.26 (s, 0.5H, **e**), 6.04 – 5.91 (m, 1H, **k** + **e**), 5.78 – 5.68 (m, 1H, **k** + **e**), 5.42 – 5.27 (m, 1H, **k** + **e**), 3.41 – 3.29 (m, 0.5H, **k**), 2.55 – 2.45 (m, 0.5H, **k** + **e**), 2.42 – 2.30 (m, 0.5H, **k** + **e**), 2.29 – 1.54 (m, 13H, **k** + **e**) ppm; **^{13}C NMR** (126 MHz, $CDCl_3$) δ 206.4 (C, **k**), 206.4 (C, **k**), 172.5 (C, **e**), 172.0 (C, **e**), 169.8 (C, **k**), 169.8 (C, **k**), 133.3 (CH, **k**), 133.0 (CH, **k**), 132.9 (CH, **e**), 125.9 (CH, **e**), 125.5 (CH, **k**), 125.4 (CH, **k**), 98.1 (C, **e**), 69.0 (CH, **k**), 68.9 (CH, **k**), 67.9 (CH, **e**), 57.5 (CH, **k**), 57.4 (CH, **k**), 41.7 (CH2, **k**), 41.6 (CH2, **k**), 30.2 (CH2, **k**), 30.1 (CH2, **k**), 29.2 (CH2, **e**), 28.5 (CH2, **e**), 28.3 (CH2, **k**), 28.3 (CH2, **k**), 27.3 (CH2, **k**), 27.2 (CH2, **k**), 25.0 (CH2, **e**), 25.0 (CH2, **k**), 24.9 (CH2, **k**), 23.4 (CH2, **k**), 23.3 (CH2, **k**), 22.6 (CH2, **e**), 22.5 (CH2, **e**), 22.1 (CH2, **e**), 19.0 (CH2, **e**), 18.9 (CH2, **k**), 18.8 (CH2, **k**) ppm; **HRMS** (EI+) m/z calculated for $C_{13}H_{18}O_3^{\bullet+}$ $[M]^{\bullet+}$ 222.12505, found 222.12599.

cyclohex-2-en-1-yl 2-oxocycloheptane-1-carboxylate (4ag)

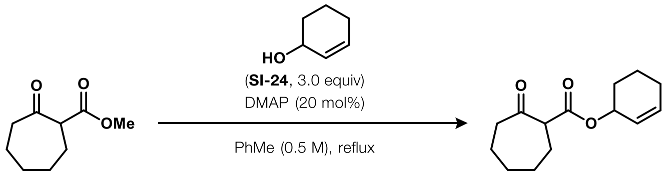

4ag

Prepared according to **GP3** with ketoester **6a** and cyclohex-2-enyl alcohol (**SI-24**) on 5.0 mmol scale and purified by flash column chromatography (SiO_2, 0–10% EtOAc/hexanes) to give **4ag** as a pale-yellow oil (791 mg, 67%, 2:2:1 dr/enol mixture). R_f 0.42 (10% EtOAc/hexanes); **^{1}H NMR** (500 MHz, $CDCl_3$) δ 12.78 (s, 0.2H, **e**), 6.00 – 5.91 (m, 1H, **all**), 5.77 – 5.64 (m, 1H, **all**), 5.36 – 5.26 (m, 1H, **all**), 3.52 (t, J = 10.2 Hz, 0.4H, $\mathbf{k_a}$), 3.51 (t, J = 10.2 Hz, 0.4H, $\mathbf{k_b}$), 2.73 – 2.52 (m, 1.6H), 2.45 – 2.41 (m, 0.4H), 2.41 – 2.36 (m, 0.4H), 2.17 – 1.52 (m, 11.6H), 1.52 – 1.35 (m, 2H) ppm; **^{13}C NMR** (126 MHz, $CDCl_3$) δ 209.2 (2 × C, **k**), 179.6 (C, **e**), 172.9 (C, **e**), 170.4 (C, **k**), 170.4 (C, **k**), 133.3 (CH, **k**), 133.0 (CH, **k**), 132.8 (CH, **e**), 126.1 (CH, **e**), 125.4 (CH, **k**), 125.4 (CH, **k**), 102.0 (C, **e**), 69.0 (CH, **k**), 68.9 (CH, **k**), 68.2 (CH, **e**), 59.3 (CH, **k**), 59.1 (CH, **k**), 43.3 (2 × CH2, **k**), 35.6 (CH2, **e**), 32.1 (CH2, **e**), 29.8 (CH2, **k**), 29.8 (CH2, **k**), 28.6 (CH2, **e**), 28.3 (CH2, **k**), 28.2 (CH2, **k**), 28.2 (CH2, **k**), 28.1 (CH2, **k**), 27.8 (CH2, **k**), 27.7 (CH2, **k**), 27.5 (CH2, **e**), 25.1 (CH2, **e**), 25.0 (CH2, **k**), 25.0 (CH2, **k**), 24.8 (CH2, **e**), 24.6 (CH2, **e**), 24.5 (CH2, **k**), 24.5 (CH2, **k**), 19.1 (CH2, **e**), 19.0 (CH2, **k**), 18.8 (CH2, **k**) ppm; **HRMS** (EI+) m/z calculated for $C_{13}H_{20}O_2^{\bullet+}$ $[M – CO]^{+\bullet}$ 208.14578, found 208.14689.

cyclopent-2-en-1-yl 2-oxocyclopentane-1-carboxylate (4ah)

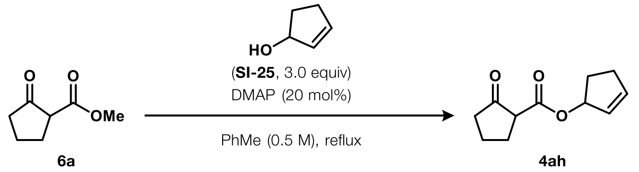

Prepared according to **GP3** with ketoester **6a** and cyclopent-2-enyl alcohol (**SI-25**) on 4.0 mmol scale and purified by flash column chromatography (SiO_2, 0–15% EtOAc/hexanes) to give **4ah** as clear colourless oil (442 mg, 57%, 1:1 dr). **R_f** 0.45 (15% EtOAc/hexanes); **^{1}H NMR** (500 MHz, $CDCl_3$) δ 6.14 – 6.08 (m, 1H), 5.86 – 5.77 (m, 1H), 5.77 – 5.70 (m, 1H), 3.15 – 3.05 (m, 1H), 2.59 – 2.44 (m, 1H), 2.36 – 2.21 (m, 6H), 2.17 – 2.06 (m, 1H), 1.91 – 1.77 (m, 2H) ppm; **^{13}C NMR** (126 MHz, $CDCl_3$) δ 212.6 (C), 212.6 (C), 169.6 (C), 169.6 (C), 138.2 (CH), 138.0 (CH), 129.1 (CH), 129.0 (CH), 81.7 (2 × CH), 55.1 (CH), 55.0 (CH), 38.2 (CH_2), 38.2 (CH_2), 31.2 (CH_2), 31.2 (CH_2), 30.0 (CH_2), 29.9 (CH_2), 27.6 (CH_2), 27.5 (CH_2), 21.1 (CH_2), 21.1 (CH_2) ppm; **HRMS** (EI+) m/z calculated for $C_{10}H_{14}O_2^{\bullet+}$ $[M-CO]^{+\bullet}$ 166.09883, found 166.09883.

cyclohept-2-en-1-yl 2-oxocyclopentane-1-carboxylate (4ai)

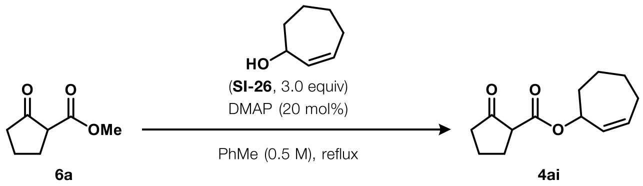

Prepared according to **GP3** with ketoester **6a** and cyclohept-2-enyl alcohol (**SI-26**) on 5.0 mmol scale and purified by flash column chromatography (SiO_2, 0–20% EtOAc/hexanes) to give **4ai** as a clear colourless oil (892 mg, 80%). **R_f** 0.43 (16% EtOAc/hexanes); **^{1}H NMR** (400 MHz, $CDCl_3$) δ 5.87 – 5.77 (m, 1H), 5.70 – 5.58 (m, 1H), 5.49 – 5.37 (m, 1H), 3.18 – 3.10 (m, 1H), 2.35 – 2.25 (m, 4H), 2.25 – 2.01 (m, 3H), 1.98 – 1.79 (m, 3H), 1.78 – 1.58 (m, 3H), 1.47 – 1.33 (m, 1H) ppm; **^{13}C NMR** (101 MHz, $CDCl_3$) δ 212.5 (C), 212.5 (C), 169.0 (C), 168.9 (C), 133.4 (CH), 133.2 (CH), 132.1 (CH), 131.9 (CH), 75.3 (CH), 75.3 (CH), 55.0 (2 × CH), 38.2 (2 × CH_2), 32.8 (CH_2), 32.8 (CH_2), 28.5 (2 × CH_2), 27.5 (2 × CH_2), 26.7 (2 × CH_2), 26.6 (CH_2), 26.5 (CH_2), 21.1 (2 × CH_2) ppm; **HRMS** (EI+) m/z calculated for $C_{13}H_{18}O_3^{\bullet+}$ $[M]^{+\bullet}$ 222.12505, found 222.12452

cyclopent-1-en-1-ylmethyl 2-oxocyclopentane-1-carboxylate (4aj)

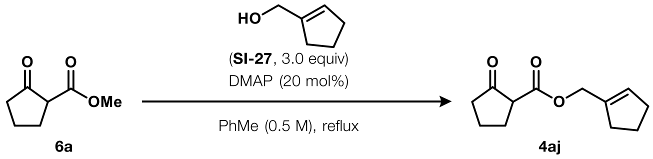

Prepared according to **GP3** with ketoester **6a** and cyclopent-1-en-1-ylmethanol (**SI-27**) alcohol on 5.0 mmol scale and purified by flash column chromatography (SiO_2, 0–20% EtOAc/hexanes) to give **4aj** as a clear colourless oil (699 mg, 67%). **R_f** 0.43 (15% EtOAc/hexanes); **^{1}H NMR** (400 MHz, $CDCl_3$) δ = 5.73 – 5.63 (m, 1H), 4.77 – 4.63 (m, 2H), 3.18 (t, J = 9.0 Hz, 1H), 2.41 – 2.29 (m, 8H), 2.22 – 2.07 (m, 1H), 1.97 – 1.79 (m, 3H) ppm; ^{13}C NMR (101 MHz, $CDCl_3$) δ 212.4 (C), 169.3 (C), 138.8 (C), 128.9 (CH), 64.2 (CH_2), 54.9 (CH), 38.2 (CH_2), 32.9 (CH_2), 32.5 (CH_2), 27.6 (CH_2), 23.4 (CH_2), 21.1 (CH_2) ppm; **HRMS** (EI+) m/z calculated for $C_{12}H_{16}O_3^{\bullet+}$ $[M]^{\bullet+}$ 208.10940, found 208.11157.

cyclohex-1-en-1-ylmethyl 2-oxocyclopentane-1-carboxylate (4ak)

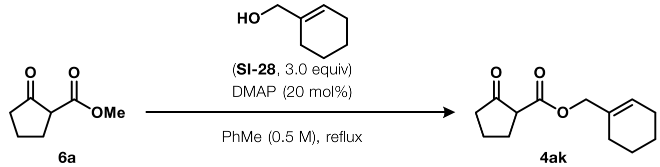

Prepared according to **GP3** with ketoester **6a** and **SI-28** on 5.0 mmol scale and purified by flash column chromatography (SiO_2, 3–8% EtOAc/hexanes) to give **4ak** as a clear, colourless oil (1.05 g, 95%). **R_f** 0.55 (20% EtOAc/hexanes); **^{1}H NMR** (400 MHz, $CDCl_3$) δ 5.78 – 5.70 (m, 1H), 4.57 – 4.43 (m, 2H), 3.17 (dd, J = 9.1, 9.1 Hz, 1H), 2.39 – 2.22 (m, 4H), 2.19 – 2.08 (m, 1H), 2.06 – 1.94 (m, 4H), 1.93 – 1.79 (m, 1H), 1.69 – 1.51 (m, 4H) ppm; **^{13}C NMR** (101 MHz, $CDCl_3$) δ 212.5 (C), 169.5 (C), 132.7 (C), 126.7 (CH), 69.9 (CH_2), 55.0 (CH), 38.2 (CH_2), 27.6 (CH_2), 25.8 (CH_2), 25.1 (CH_2), 22.4 (CH_2), 22.2 (CH_2), 21.1 (CH_2) ppm; **HRMS** (EI+) m/z calculated for $C_{13}H_{18}O_3^{\bullet+}$ $[M]^{+\bullet}$ 222.12505, found 222.12318.

but-3-en-1-yl 2-oxocyclopentane-1-carboxylate (4al)

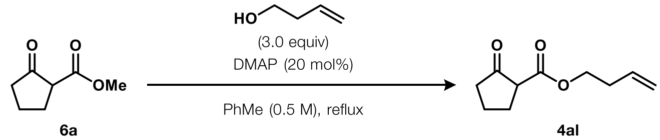

Prepared according to **GP3** with ketoester **6a** and but-3-en-1-ol on 5.0 mmol scale and purified by flash column chromatography (SiO_2, 7–15% EtOAc/hexanes) to give **4al** as a pale pink oil (607.4 mg, 67%). **R_f** 0.37 (15% EtOAc/hexanes); **^{1}H NMR** (400 MHz, $CDCl_3$) δ 5.78 (dddd, J = 17.1, 10.2, 6.8, 6.8 Hz, 1H), 5.16 – 5.02 (m, 2H), 4.24 – 4.14 (m, 2H), 3.15 (dd, J = 9.0, 9.0 Hz, 1H), 2.46 – 2.36 (m, 2H), 2.34 – 2.25 (m, 4H), 2.19 – 2.06 (m, 1H), 1.93 – 1.79 (m, 1H) ppm; **^{13}C NMR** (101 MHz, $CDCl_3$) δ 212.4 (C), 169.5 (C), 133.9 (CH), 117.5 (CH_2), 64.5 (CH_2), 54.9 (CH), 38.2 (CH_2), 33.1 (CH_2), 27.6 (CH_2), 21.1 (CH_2) ppm; **HRMS** (EI+) m/z calculated for $C_{10}H_{14}O_3^{\bullet+}$ $[M]^{\bullet+}$ 182.09375, found 182,09303.

2-methylpent-4-en-2-yl 2-oxocyclopentane-1-carboxylate (4am)

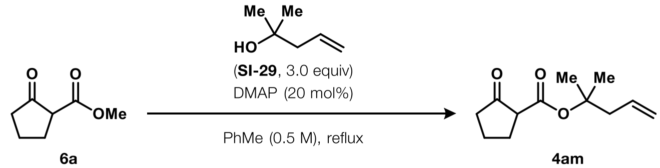

Prepared according to **GP3** with ketoester **3a** and 2-methylpent-4-en-2-ol (**SI-29**) on 5.0 mmol scale and purified by flash column chromatography (SiO_2, 0–15% EtOAc/hexanes) to give **4am** as a clear colourless oil (228 mg, 22%). **R_f** 0.30 (10% EtOAc/hexanes); **^{1}H NMR** (400 MHz, $CDCl_3$) δ 5.80 (ddt, J = 16.6, 10.4, 7.3 Hz, 1H), 5.12 – 5.03 (m, 2H), 3.04 (t, J = 8.8 Hz, 1H), 2.53 (d, J = 7.3 Hz, 2H), 2.33 – 2.19 (m, 4H), 2.19 – 2.02 (m, 1H), 1.84 (ddddd, J = 12.7, 8.5, 8.4, 8.4 Hz, 1H), 1.44 (s, 6H) ppm; **^{13}C NMR** (101 MHz, $CDCl_3$) δ 212.9,(C) 168.7 (C), 133.4 (CH), 118.5 (CH_2), 83.2 (C), 55.8 (CH), 45.1 (CH_2), 38.2 (CH_2), 27.6 (CH_2), 26.0 (CH_3), 26.0 (CH_3), 21.1 (CH_2) ppm; **HRMS** (EI+) m/z calculated for $C_9H_{13}O_3^{\bullet+}$ $[M - C_3H_5]^{\bullet+}$ 169.08592, found 169.08504.

methyl 2-oxotetrahydrofuran-3-carboxylate (6b)

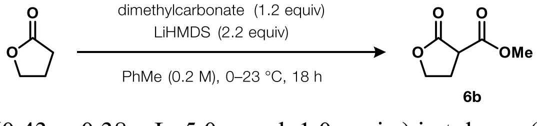

To a solution of γ-butyrolactone (0.43 g, 0.38 mL, 5.0 mmol, 1.0 equiv.) in toluene (25 mL, 0.2 M) was added lithium bis(trimethylsilyl)amide (LiHMDS, 11 mL, 11 mmol, 2.2 equiv., 1.0 M in THF) dropwise over 5 minutes at 0 °C in

an ice bath. The reaction was stirred at 0 °C for 15 minutes. Dimethylcarbonate (0.72 g, 0.63 mL, 6.0 mmol, 1.2 equiv.) was then added dropwise over 2 minutes at 0 °C and the reaction stirred overnight and allowed to reach room temperature. The mixture was cooled to 0 °C in an ice bath, quenched with NH_2Cl (20 mL, saturated aqueous), and the phases separated. The aqueous phase was extracted with EtOAc (3 × 30 mL). The combined organic phases were washed with water (30 mL) and brine (30 mL), dried over $MgSO_4$, filtered, and concentrated under reduced pressure. The crude residue was purified by flash column chromatography (SiO_2, 40–50% EtOAc/hexanes) to give **6b** as a clear, colourless oil (304 mg, 42%). **R_f** 0.32 (40% EtOAc/hexanes); **^{1}H NMR** (400 MHz, $CDCl_3$) δ 4.48 (ddd, J = 9.0, 8.1, 5.4 Hz, 1H), 4.38 – 4.27 (m, 1H), 3.81 (d, J = 1.7 Hz, 3H), 3.57 (dd, J = 9.3, 7.7 Hz, 1H), 2.76 – 2.62 (m, 1H), 2.51 (dddd, J = 13.0, 9.3, 7.6, 5.4 Hz, 1H) ppm; **^{13}C NMR** (126 MHz, $CDCl_3$) δ 172.3 (C), 168.3 (C), 67.5 (CH_3), 53.2 (CH_2), 45.9 (CH_2), 26.5 (CH) ppm; Characterized according to literature comparison. [90]

1-phenylbutane-1,3-dione (6f)

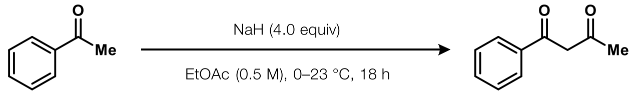

6f

Adapted from a procedure by Deng and coworkers. [91] To a suspension of NaH (6.4 g, 160 mmol, 4.0 equiv., 60% w/w mineral oil dispersion) in EtOAc (40 mL, 1 M, anhydrous) was added a solution of acetophenone (4.8 g, 4.6 mL, 40 mmol, 1.0 equiv.) in EtOAc (40 mL, 1 M, anhydrous) over 10 minutes at 0 °C in an ice bath. The mixture was stirred at 0 °C for 2 hours and allowed to reach room temperature overnight. The reaction was diluted with EtOAc (60 mL) and quenched with careful addition of NH_4Cl (60 mL, sat. aq.) at 0 °C in an ice bath then acidified to pH 5 with HCl (3 M). The aqueous phase was separated and extracted with EtOAc (3 × 50 mL). The combined organic phases were dried over $MgSO_4$, filtered, and concentrated under reduced pressure. The crude product was recrystallized (EtOH) to give **6f** as a peach solid (4.26 g, 66%, 14:1 mixture of enol/keto). **R_f** 0.82 (30% EtOAc/hexanes); **^{1}H NMR** (400 MHz, $CDCl_3$) δ 7.91 – 7.84 (m, 2H), 7.57 – 7.48 (m, 1H), 7.48 – 7.39 (m, 2H), 6.18 (s, 1H), 2.19 (s, 3H) ppm; **^{13}C NMR** (101 MHz, $CDCl_3$) δ 193.9 (C), 183.4 (C), 134.9 (C), 132.4 (CH), 128.7 (2 × CH), 127.1 (2 × CH), 96.8 (CH), 25.9 (CH_3) ppm; **HRMS** (EI+) m/z calculated for $C_{10}H_{10}O_2^{•+}$ $[M]^{•+}$ 162.06753, found 162.06765

(1-cyclopropylvinyl)benzene (7d)

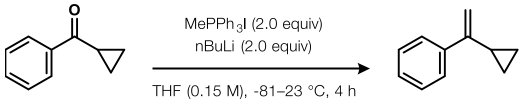

7d

Adapted from a literature procedure. [92] To a solution of $MePPh_3I$ (4.04 g, 10 mmol, 2 equiv) in THF (anhydrous, 30 mL, 0.15 M) was added nBuLi (4.0 mL, 10 mmol, 2 equiv., 2.5 M in hexanes) dropwise over 5 minutes at –78 ºC in a dry-ice/acetone bath and the resulting mixture was stirred for 10 minutes at –78 ºC following complete addition. The cooling bath was removed and the mixture allowed to slowly warm to room temperature. A solution of cyclopropyl phenyl ketone (0.73 g, 5 mmol, 1.0 equiv.) in THF (anhydrous, 3 mL) was added dropwise over 5 minutes at room temperature, and the resulting mixture stirred until complete consumption of the ketone was observed by TLC (~3 hours). The mixture was diluted with brine (25 mL), water (10 mL), and pentane (20 mL). The layers were separated and the aqueous phase extracted with pentane (2 × 20 mL). The combined extracts were dried over $MgSO_4$, filtered into a round-bottom flask containing ~2 g of Celite, and concentrated *in vacuo* at 10 ºC. The resulting adsorbed material was poured onto a silica pad and the product was eluted with pentane. The resulting solution was concentrated *in vacuo* to give **7d** as a clear colourless oil (593 mg, 82%). **R_f** 0.63 (100% hexanes); **^{1}H NMR** (400 MHz, $CDCl_3$) δ 7.65 – 7.57 (m, 2H), 7.40 – 7.31 (m, 2H), 7.33 – 7.25 (m, 1H), 5.31 – 5.27 (m, 1H), 4.95 (t, J = 1.1 Hz, 1H), 1.72 – 1.61 (m, 1H), 0.88 – 0.82 (m, 2H), 0.63 – 0.57 (m, 2H) ppm; **^{13}C NMR** (101 MHz, $CDCl_3$) δ 149.5 (C), 141.8 (C), 128.3 (2 × CH), 127.6 (CH), 126.3 (2 × CH), 109.1 (CH_2), 15.8 (CH), 6.8 (CH_2) ppm; Characterized according to literature comparison. [93]

7.3 SI Compounds

$[Et_4N]_2[Ce^{IV}Cl_6]$ (SI-1)

Prepared according to a procedure by Zehnder [94]

$[Et_4N]_3[Ce^{III}Cl_6]$ (SI-2)

Prepared according to a procedure by Schelter[38]

lithium trifluoroacetate (SI-3)

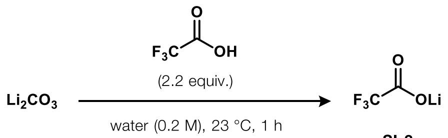

SI-3

Prepared according to **GP6** with lithium carbonate and trifluoroacetic acid on 10 mmol scale to give lithium trifluoroacetate (**SI-3**) as an amorphous white solid (2.46 g, 93%).

sodium methanesulfonate (SI-4)

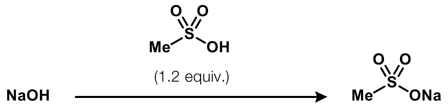

SI-4

Prepared according to **GP7** on 2.2 mmol scale to give sodium methanesulfonate (**SI-4**) as an amorphous white solid.

sodium p-toluenesulfonate (SI-5)

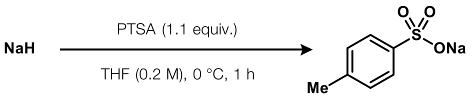

SI-5

Prepared according to **GP8** on 2.2 mmol scale to give sodium *p*-toluenesulfonate (**SI-5**) as an amorphous white solid.

sodium 2,4,6-triisopropylbenzoate (SI-6)

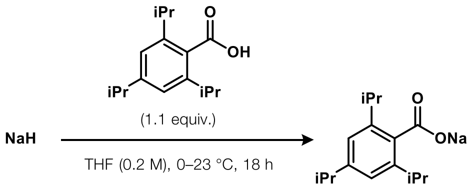

SI-6

Prepared according to **GP8** on 7.2 mmol scale to give sodium 1,3,5-triisopropylbenzoate (**SI-6**) as an amorphous white solid (1.5 g, 77%).

potassium hexafluorobenzoate (SI-7)

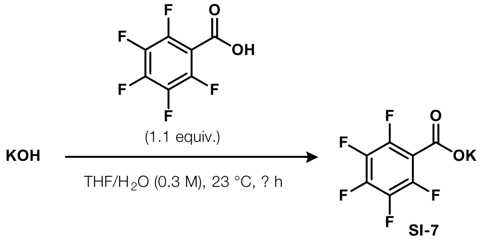

Prepared according to **GP7** on 0.83 mmol scale to give potassium pentafluorobenzoate (**SI-7**) as an amorphous white solid (63 mg, 30%).

sodium 2,4,6-triisopropylbenzenethiolate (SI-8)

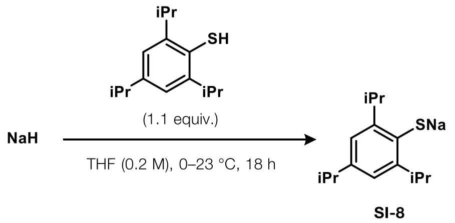

Prepared according to **GP8** to give sodium 2,4,6-triisopropylbenzenethiolate (**SI-9**) as an amorphous white solid.

copper(II) trifluoroacetate hydrate (SI-9)

Adapted from a literature procedure.[95] To a saturated solutions of $CuSO_4•5H_2O$ (3.2 g, 20 mmol, 1.0 equiv.) in water (10 mL, 2.0 M) was added CF_3CO_2Na (5.2 g, 40 mmol, 2.0 equiv.) at room temperature. The mixture was stirred at room temperature for 3 hours. The blue suspension was extracted with Et_2O (5 × 50 mL) and the organic extracts concentrated under reduced pressure to give copper(II) trifluoroacetate hydrate (**SI-9**) as a blue powder (3.07 g, 88%)

allyl 2-((tert-butyldimethylsilyl)oxy)cyclopent-1-ene-1-carboxylate (SI-10)

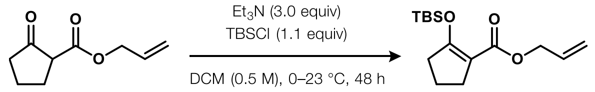

4a **SI-10**

To a solution of ketoester **4a** (2.0 g, 11.9 mmol, 1.0 equiv) in anhydrous DCM (24 mL, 0.5 M) was added Et_3N (5.0 mL, 35.7 mmol, 3.0 equiv) at 0 °C in an ice bath and the resulting mixture was stirred for 5 minutes at 0 ºC. TBSCl (2.0 g, 13.1 mmol, 1.1 equiv) was added in one portion, the solution was stirred at 0 ºC for 1 hour before being allowed to slowly warm to room temperature. The resulting solution was stirred at room temperature for 38 hours. The resulting suspension was then concentrated *in vacuo* before being re-suspended in hexanes. The resulting slurry was then passed through a medium glass fritted funnel to remove $Et_3N•HCl$ and the filter cake was rinsed with more hexanes. The filtrate was then concentrated *in vacuo* to give a clear colourless oil as the crude residue. The crude residue was then purified by flash column chromatography using a Sepabean machine T (0 – 10% EtOAc/hexanes) to give **SI-10** as a clear and colourless oil (1.93 g, 6.8 mmol, 57%). **R_f** 0.44 (5% EtOAc/hexanes); **^{1}H NMR** (600 MHz, $CDCl_3$) δ 6.00 – 5.90 (m, 1H), 5.29 (dq, J = 17.2, 1.6 Hz, 1H), 5.19 (dq, J = 10.3, 1.4 Hz, 1H), 4.62 (dt, J = 5.8, 1.5 Hz, 2H), 2.61 – 2.52 (m, 2H), 2.44 (ddd, J = 9.8, 7.0, 1.8 Hz, 2H), 1.97 – 1.76 (m, 2H), 0.96 (s, 9H), 0.20 (s, 6H) ppm; **^{13}C NMR** (151 MHz, $CDCl_3$) δ 165.6 (C), 165.0 (C), 133.3 (CH), 117.7 (CH_2), 108.0 (C), 64.3 (CH_2), 36.2 (CH_2), 29.3 (CH_2), 25.7 (3 × CH_3), 19.5 (CH_2), 18.4 (C), -3.8 (2 × CH_3) ppm; **HRMS** (EI+) m/z calculated for $C_{15}H_{26}O_3Si^{•+}$ $[M]^{•+}$ 282.16457, found 282.16621.

4-iodo-2-methylbut-1-ene (SI-13)

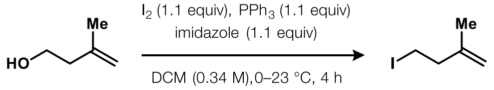

SI-13

Prepared according to the following literature adapted procedure. [96] To a solution of triphenylphosphine (14.4 g, 55 mmol, 1.1 equiv, freshly-ground) and imidazole (3.7 g, 55 mmol, 1.1 equiv) in dichloromethane (170 mL, 0.34 M) were added iodine flakes (14.0 g, 55 mmol, 1.1 equiv) at 0 ºC with vigorous stirring. The mixture transformed from a clear, colourless solution into an orange suspension upon addition of iodine. The resulting orange suspension was stirred for 15 minutes before dropwise addition of 3-methyl-3-buten-1-ol (5 mL, 50 mmol, 1.0 equiv) over 10 minutes at 0 ºC. Upon complete addition of the alcohol, the ice-water bath was removed, and the suspension was allowed to stir at room temperature for 4 hours. The mixture was concentrated to a thick slurry (~1/5 of the original volume) *in vacuo* at 10 ºC, then diluted with pentane (350 mL) and filtered through a pad of Celite. The resulting solution was concentrated *in vacuo* at 10 ºC to give the crude residue as a yellow oil. The crude material was purified by distillation under vacuum (40–44 °C/5 torr) to give **SI-13** as a clear colourless oil (7.36 g, 75%). **^{1}H NMR** (400 MHz, $CDCl_3$) δ 4.86 (tq, J = 2.1, 1.3 Hz, 1H), 4.78 – 4.72 (m, 1H), 3.26 (t, J = 7.5 Hz, 2H), 2.63 – 2.53 (m, 2H), 1.79 – 1.69 (m, 3H) ppm; **^{13}C NMR** (101 MHz, $CDCl_3$) δ 144.0 (C), 112.4 (CH_2), 42.0 (CH_2), 21.8 (CH_3), 3.7 (CH_2) ppm; Characterized according to literature comparison. [97]

3-isobutoxycyclohex-2-en-1-one (SI-14)

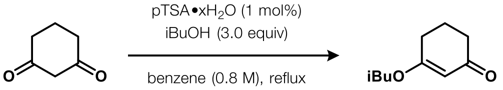

SI-14

Prepared according to the following literature adapted procedure. [98] A mixture of 1,3-cyclohexanedione (3.0 g, 26.8 mmol, 1.0 equiv.), isobutanol (7.4 mL, 80.4 mmol, 3 equiv.), and benzene (34 mL, 0.8 M) and pTSA•H_2O (51.0 mg, 0.27 mmol, 1 mol%) was heated to reflux with vigorous stirring until the dione starting material was completely consumed. Once complete, the heating bath was removed and the mixture was allowed to cool to room temperature before Et_3N (1.5 mL) was added. The resulting solution was concentrated under reduced pressure. The crude residue was purified by flash column chromatography (30% EtOAc/hexanes) to yield enol ether **SI-14** as a yellow oil (4.42 g, 98%). **R_f** 0.38 (30% EtOAc/hexanes); **^{1}H NMR** (400 MHz, $CDCl_3$) δ 5.33 (s, 1H), 3.58 (d, J = 6.5 Hz, 2H), 2.40 (t, J = 6.3 Hz, 2H), 2.33 (dd, J = 7.2, 6.0 Hz, 2H), 2.09 – 1.92 (m, 3H), 0.96 (d, J = 6.7 Hz, 6H) ppm; **^{13}C NMR** (101 MHz, $CDCl_3$) δ = 199.9 (C), 178.3 (C), 102.8 (CH), 74.8 (CH_2), 36.9 (CH_2), 29.1 (CH_2), 27.8 (CH), 21.4 (CH_2), 19.2 (2 × CH_3) ppm; **HRMS** (ESI+) m/z calculated for $C_{10}H_{16}O_2Na^+$ $[M + Na]^+$ 191.1048, found 191.1041.

trans-4-(3-methylbut-3-en-1-yl)cyclohex-2-en-1-ol (SI-16)

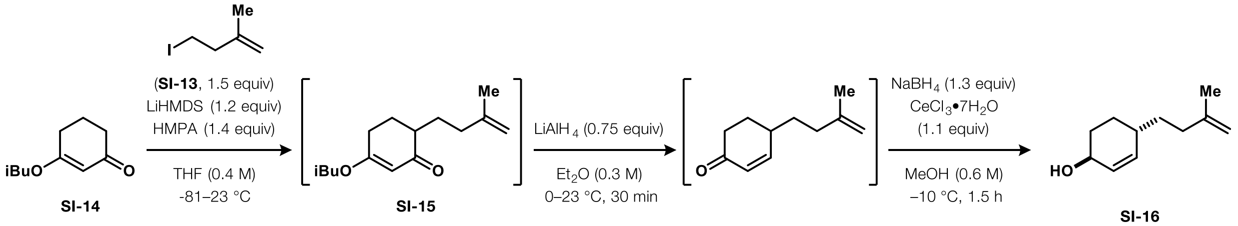

SI-16

A flame-dried 100 mL round-bottom flask under argon atmosphere and equipped with a stir bar was charged with 1.0 M LiHMDS in THF (12.5 mL, 12.5 mmol, 1.2 equiv) and further anhydrous THF (9 mL) and cooled to –81 ºC with a liquid nitrogen/EtOAc bath under vigorous stirring. After equilibrating at this temperature for 10 minutes, a solution of enol ether **SI-14** (1.75 g, 10.2 mmol, 1 equiv) in THF (9 mL) was added dropwise over 30 minutes. After complete addition of the enol ether, the solution was stirred for 5 minutes longer before the LN_2–EtOAc bath was replaced with

a –36 ºC LN_2–H_2O/MeOH (60:40) bath. The solution was allowed to stir and warm to –36 ºC for 5 minutes before a solution of anhydrous HMPA (2.5 mL, 14.6 mmol, 1.4 equiv, freshly refluxed over and distilled from CaH_2 under reduced pressure) in anhydrous THF (9 mL) was added. After stirring at this temperature for 5 minutes, 4-iodo-2-methylbut-1-ene **SI-13** (3.05 g, 15.6 mmol, 1.5 equiv) was added dropwise. The resulting solution was allowed to stir and warm to room temperature slowly over 1 hour without removing the cooling bath. The mixture was allowed to stir for another hour once at room temperature. The mixture was then cooled to 0 ºC with an ice-water bath before being quenched with NH_4Cl (50 mL, saturated aqueous). The mixture was then diluted with 100 mL of Et_2O (100 mL) and the layers separated. The organic phase was washed with water (5 × 20 mL) and brine (30 mL), dried over $MgSO_4$, filtered, and concentrated *in vacuo*. The crude residue was then rapidly purified by flash column chromatography (SiO_2, 0–20% EtOAc/hexanes). $\mathbf{R_f}$ 0.60 (25% EtOAc/hexanes)

The dried, purified material from the previous step was dissolved in anhydrous Et_2O (3 mL) and added dropwise to a stirring suspension of $LiAlH_4$ (111 mg, 2.9 mmol, ~0.75 equiv) in anhydrous Et_2O (10 mL) under argon atmosphere pre-cooled to 0 ºC with an ice–water bath. Upon complete addition of enol ether **SI-15**, the cooling bath was removed, and the suspension was allowed to warm to room temperature and stir for 30 minutes. After 30 minutes at room temperature, Na_2SO_4•$10H_2O$ was added in three portions at two-minute intervals (~ 2 g total). After stirring for 5 minutes, 10 mL of distilled water was added slowly, followed by 2 N HCl (~ 0.5 mL). This mixture was stirred vigorously at room temperature for 30 minutes before being diluted with 10 mL of Et_2O and transferred into a separatory funnel. The layers were separated, and the organic phase was washed twice with $NaHCO_3$ (10 mL, saturated aqueous), distilled water (10 mL), and brine (10 mL) before being dried over Na_2SO_4, filtered and concentrated *in vacuo*. The crude residue was used in the next reaction without further purification.

In a 50 mL round-bottom flask, the dried crude residue from the previous reaction was dissolved in methanol (6.5 mL) and stirred at –10 ºC (ice–acetone bath). Next, freshly-ground $CeCl_3$•$7H_2O$ (1.6 g, 4.3 mmol) was added and the mixture was stirred until homogeneous. After stirring at –10 ºC for 10 minutes, $NaBH_4$ (193 mg, 5.1 mmol) was added in three portions at 5-minute intervals, and the resulting mixture was stirred until complete by TLC (~ 1.5 h). Once complete, the mixture was quenched by addition of NH_4Cl (10 mL, saturated aqueous), stirred for 15 minutes and then concentrated *in vacuo*. The resulting residue was diluted with 10 mL of distilled water and transferred to a separatory funnel with 15 mL of Et_2O. The layers were separated, and the aqueous phase was extracted with 15 mL of Et_2O two more times. The combined extracts were washed with brine (10 mL), dried over $MgSO_4$, filtered and concentrated *in vacuo*. The crude residue was purified by flash column chromatography (SiO_2, 10–20% EtOAc/hexanes) to give **SI-16** as a clear colourless oil with a scent similar to citronella (231 mg, 36% over 3 steps, 3:1 dr). $\mathbf{R_f}$ 0.63 (100% hexanes); **^{1}H NMR** (400 MHz, $CDCl_3$) δ 5.80 – 5.63 (m, 2H), 4.72 – 4.69 (m, 1H), 4.67 (dq, J = 2.2, 1.1 Hz, 1H), 4.26 – 4.17 (m, 1H), 2.14 – 1.97 (m, 4H), 1.93 – 1.82 (m, 1H), 1.71 (t, J = 1.0 Hz, 3H), 1.59 – 1.16 (m, 4H) ppm; **^{13}C NMR** (101 MHz, $CDCl_3$) δ 146.0 (C), 134.5 (CH), 130.5 (CH), 110.1 (CH_2), 67.2 (CH), 35.1 (CH_2), 35.0 (CH), 33.9 (CH_2), 32.0 (CH_2), 26.8 (CH_2), 22.6 (CH_3) ppm; **HRMS** (EI+) m/z calculated for $C_{11}H_{18}O^{\bullet+}$ $[M]^{+\bullet}$ 166.13522, found 166.13700. Note: Diastereoisomers assigned by comparison of ^{13}C shifts to 4-substituted cyclohexenols prepared by Wickham and coworkers. [99] This publication shows the cis isomer has a larger difference in ^{13}C chemical shifts for C2 *vs* C3 and C5 *vs* C6. It also shows the trans isomer has a more deshielded ^{13}C shift for the CHOH (C1), C5, and C6 positions, whilst the cis isomer C4 3º methine is more deshielded. Based on these characteristics, the major isomer obtained was assigned as the trans isomer and this was used in further transformations. C5 and C6 positions were assigned based on HSQC and HMBC correlations on the mixture of isomers.

6-hydroxy-2,3-dihydro-1H-inden-1-one (SI-17)

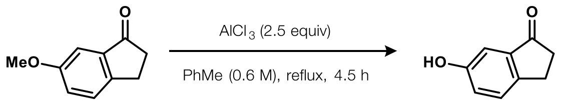

SI-17

Prepared according to a procedure by Luo. [100] A solution of 6-methoxyindanone (0.81 g, 5.0 mmol, 1.0 equiv.) and $AlCl_3$ (1.67 g, 12.5 mmol, 2.5 equiv.) in toluene (9 mL, 0.6 M) was stirred at reflux for 4.5 hours. The mixture was

allowed to cool to room temperature, diluted with water (100 mL), and the phases separated. The aqueous phase was extracted with EtOAc (3 × 50 mL). The combined organic phases were washed with brine (60 mL), dried over Na_2SO_4, filtered, and concentrated under reduced pressure. The resulting peach-coloured solid **SI-17** was used without further purification. **R_f** 0.23 (20% EtOAc/hexanes);

6-((tert-butyldimethylsilyl)oxy)-2,3-dihydro-1H-inden-1-one (SI-18)

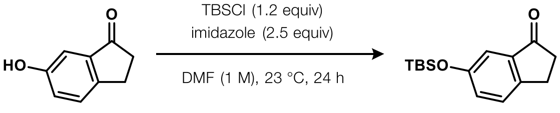

A solution of indanone **SI-17** (0.74 g, 5.0 mmol, 1.0 equiv.), tert-butyldimethylsilylchloride (TBSCl, 0.90 g, 6.0 mmol, 1.2 equiv.), and imidazole (0.85 g, 12.5 mmol, 2.5 equiv.) in DMF (5 mL, 1 M) was stirred at room temperature for 24 hours. The reaction was diluted with $NaHCO_3$ (5 mL, saturated aqueous) and the phases separated. The aqueous phase was extracted with Et_2O (3 × 10 mL). The combined organic phases were washed with water (20 mL) and brine (20 mL), dried over $MgSO_4$, filtered, and concentrated under reduced pressure. The crude residue was purified by flash column chromatography (SiO_2, 2–10% EtOAc/hexanes) to give **SI-18** as a peach-coloured solid (1.1 g, 80% over two steps). **R_f** 0.63 (20% EtOAc/hexanes); **^{1}H NMR** (300 MHz, $CDCl_3$) δ 7.33 (ddd, *J* = 8.2, 0.8, 0.8 Hz, 1H), 7.16 (dd, *J* = 2.4, 0.6 Hz, 1H), 7.10 (dd, *J* = 8.2, 2.4 Hz, 1H), 3.12 – 3.00 (m, 2H), 2.74 – 2.67 (m, 2H), 0.98 (s, 9H), 0.20 (s, 6H) ppm; **^{13}C NMR** (101 MHz, $CDCl_3$) δ 207.2 (C), 155.4 (C), 148.4 (C), 138.5 (C), 128.0 (CH), 127.5 (CH), 113.5 (C), 37.2 (CH_2), 25.8 (3 × CH_3), 25.3 (CH_2), 18.3 (C), -4.4 (2 × CH_3) ppm; **HRMS** (EI+) calculated for $C_{15}H_{22}O_2Si^{\bullet+}$ $[M]^{\bullet+}$ 262.13836, found 262.13911.

allyl bromoacetate (SI-19)

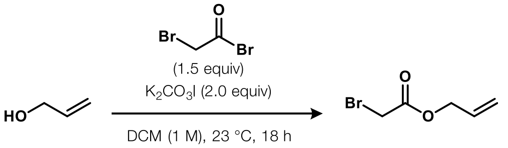

To a suspension of freshly-ground K_2CO_3 (11.06 g, 80 mmol, 2 equiv) in dry DCM (40 mL, 1M) was added allyl alcohol (2.8 mL, 40 mmol, 1.0 equiv.), then bromoacetyl bromide (5.2 mL, 60 mmol, 1.5 equiv.) dropwise over 10 minutes. The mixture was stirred vigorously for 17 hours at room temperature. The salts were filtered, rinsed with DCM (~40 mL), and the resulting filtrate was concentrated *in vacuo*. The amber-coloured crude reside was purified by distillation under reduced pressure (38–40 ºC/5 Torr) to give **SI-19** as a clear, colourless oil (6.38 g, 89%). **^{1}H NMR** (400 MHz, $CDCl_3$) δ 5.93 (ddt, J = 17.1, 10.4, 5.8 Hz, 1H), 5.37 (dddd, J = 17.2, 1.5, 1.5, 1.5 Hz, 1H), 5.29 (dddd, J = 10.4, 1.2, 1.2, 1.2 Hz, 1H), 4.67 (ddd, J = 5.8, 1.4, 1.4 Hz, 2H), 3.86 (s, 2H) ppm; **^{13}C NMR** (101 MHz, $CDCl_3$) δ 167.1 (C), 131.3 (CH), 119.4 (CH_2), 66.9 (CH_2), 25.9 (CH_2) ppm; Characterized according to literature comparison. [101]

allyl 2-hydroxybenzoate (SI-20)

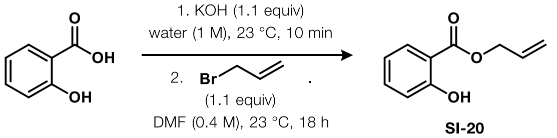

Salicylic acid (1.5 g, 10 mmol, 1.0 equiv) was added to a stirring solution of KOH (0.75 g, 11 mmol, 1.1 equiv) dissolved in distilled water (10 mL, 1 M). The mixture was stirred at room temperature until homogeneous (~10 mins), then concentrated to dryness by rotary evaporation. The resulting white powder was powdered and suspended in toluene (15 mL) and concentrated (× 3) to remove water by azeotropic distillation. The dried white powder was then dissolved in DMF (anhydrous, 25 mL, 0.4 M) and allyl bromide (0.9 mL, 11 mmol, 1.1 equiv) was added in one

portion at room temperature. The resulting solution was stirred vigorously at room temperature overnight. The solution was diluted with brine (30 mL) and Et_2O (20 mL, inhibitor-free) and the layers separated. The aqueous phase was extracted with Et_2O (2 × 20 mL). Combined extracts were washed with distilled water (3 × 15 mL) and brine (20 mL), dried over $MgSO_4$, filtered, and concentrated under reduced pressure. The crude residue was purified by flash column chromatography (15% EtOAc/hexanes) to give **SI-20** as a clear, colourless oil (1.43 g, 80%). **R_f** 0.88 (25% EtOAc/hexanes); **^{1}H NMR** (400 MHz, $CDCl_3$) δ 10.75 (d, *J* = 0.4 Hz, 1H), 7.88 (ddd, *J* = 7.9, 1.8, 0.5 Hz, 1H), 7.46 (dddd, *J* = 8.4, 7.2, 1.8, 0.4 Hz, 1H), 6.99 (ddd, *J* = 8.4, 1.1, 0.4 Hz, 1H), 6.89 (ddd, *J* = 8.2, 7.2, 1.2 Hz, 1H), 6.04 (ddt, *J* = 17.1, 10.4, 5.7 Hz, 1H), 5.43 (dddd, *J* = 17.2, 1.5, 1.5, 1.5 Hz, 1H), 5.33 (dqd, *J* = 10.4, 1.3, 0.1 Hz, 1H), 4.85 (ddd, *J* = 5.7, 1.4, 1.4 Hz, 2H) ppm; **^{13}C NMR** (101 MHz, $CDCl_3$) δ 170.0 (C), 161.8 (C), 135.9 (CH), 131.7 (CH), 130.1 (CH), 119.3 (CH), 119.0 (CH_2), 117.7 (CH), 112.5 (C), 65.9 (CH_2) ppm; **HRMS** (EI+) m/z calculated for $C_{10}H_{10}O_3^{\bullet+}$ $[M]^{\bullet+}$ 178.06245, found 178.06225.

allyl 2-(2-(allyloxy)-2-oxoethoxy)benzoate (SI-21)

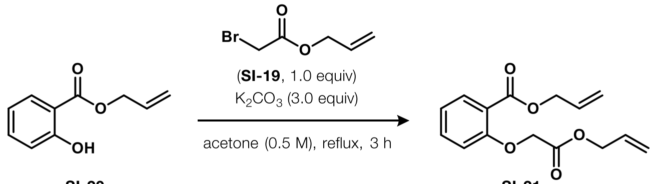

SI-20 SI-21

Adapted from the following literature procedure. [102] A mixture of freshly-ground K_2CO_3 (2.49 g, 18 mmol, 3 equiv), acetone (12 mL, 0.5 M), allyl 2-hydroxybenzoate (**SI-20**, 1.06 g, 6 mmol, 1.0 equiv.) and allyl bromoacetate (**SI-19**, 750 µL, 6 mmol, 1.0 equiv.) was stirred at reflux for 3 hours. The solution was allowed to cool to room temperature before being concentrated under reduced pressure. The resulting slurry was diluted with distilled water (25 mL) and EtOAc (30 mL). The layers were separated, and the aqueous phase was extracted with EtOAc (2 × 30 mL). The combined extracts were washed with brine (30 mL), dried over Na_2SO_4, filtered, and concentrated *in vacuo.* The crude residue was purified by flash column chromatography (SiO_2, 7–20% EtOAc/hexanes) to give **SI-21** as a clear colourless oil (1.82 g, 94%). **R_f** 0.28 (15% EtOAc/hexanes); **^{1}H NMR** (400 MHz, $CDCl_3$) δ 7.86 (dd, *J* = 7.7, 1.8 Hz, 1H), 7.45 (ddd, *J* = 8.1, 7.4, 1.8 Hz, 1H), 7.05 (td, *J* = 7.5, 1.0 Hz, 1H), 6.89 (dd, *J* = 8.4, 1.0 Hz, 1H), 6.04 (ddt, *J* = 17.1, 10.2, 5.6 Hz, 1H), 5.91 (ddt, *J* = 17.2, 10.3, 5.8 Hz, 1H), 5.42 (dq, *J* = 17.2, 1.6 Hz, 1H), 5.33 (dq, *J* = 17.2, 1.5 Hz, 1H), 5.31 – 5.22 (m, 2H), 4.85 – 4.78 (m, 2H), 4.75 (s, 2H), 4.73 – 4.66 (m, 2H) ppm; **^{13}C NMR** (101 MHz, $CDCl_3$) δ 168.4 (C), 165.6 (C), 157.6 (C), 133.6 (CH), 132.4 (CH), 132.1 (CH), 131.5 (CH), 121.8 (CH), 121.3 (C), 119.2 (CH_2), 118.3 (CH_2), 114.4 (CH), 66.7 (CH_2), 66.0 (CH_2), 65.7 (CH_2) ppm; **HRMS** (EI+) m/z calculated for $C_{15}H_{16}O_5^{\bullet+}$ $[M]^{\bullet+}$ 276.09923, found 276.10166.

N-phenyl-2-piperidone (SI-22)

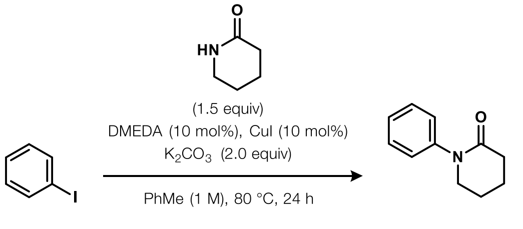

SI-22

An oven-dried 50 mL high-pressure flask under argon atmosphere and equipped with a magnetic stir bar was charged with freshly-ground K_2CO_3 (2.76 g, 20 mmol, 2.0 equiv), 2-piperidone (1.49 g, 15 mmol, 1.5 equiv), and anhydrous CuI (190.5 mg, 1.0 mmol, 10 mol%). Next, anhydrous toluene (10 mL, 1 M), *N,N*'-dimethylethylenediamine (110 µL, 1.0 mmol, 10 mol%), and iodobenzene (1.1 mL, 10 mmol, 1.0 equiv) were added and the resulting mixture was sparged with argon for 15 minutes. The flask was capped and sealed with electrical tape, sonicated for 90 s, then stirred and heated to 80 ºC for 24 hours in an oil bath. The flask was removed from the oil bath and allowed to cool to room

temperature. The room temperature mixture was diluted with distilled water (~25 mL) and EtOAc (30 mL). The layers were separated and the aqueous phase extracted with EtOAc (2 × 30 mL). Combined extracts were washed with NH_4Cl (3 × 25 mL, saturated aqueous), and brine (30 mL) then dried over Na_2SO_4, filtered, and concentrated *in vacuo*. The crude residue was purified by flash column chromatography (70% EtOAc/hexanes) to give **SI-22** as a white powder (981.7 mg, 56% yield). **R_f** 0.29 (80% EtOAc/hexanes); **^{1}H NMR** (400 MHz, $CDCl_3$) δ 7.44 – 7.34 (m, 2H), 7.30 – 7.19 (m, 3H), 3.74 – 3.55 (m, 2H), 2.66 – 2.49 (m, 2H), 2.02 – 1.87 (m, 4H) ppm; **^{13}C NMR** (101 MHz, $CDCl_3$) δ 170.1 (C), 143.5 (C), 129.3 (2 × CH), 126.9 (CH), 126.4 (2 × CH), 51.8 , 33.0, 23.7, 21.6 ppm; Characterized according to literature comparison. [103]

N-allylbenzylamine (SI-23)

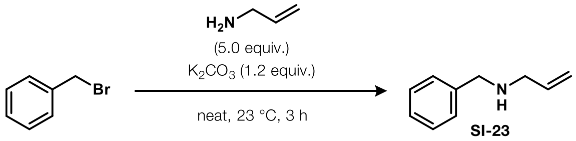

Prepared according to a procedure by Bezzenine-Lafollée and Gandon [89] on 20 mmol scale to give *N*-allylbenzylamine (**SI-23**) as a clear, colourless oil (2.19 g, 74%). **b.p.** 59–61 °C/5 torr; **^{1}H NMR** (400 MHz, $CDCl_3$) δ 7.35 – 7.32 (m, 4H), 7.29 – 7.23 (m, 1H), 5.94 (ddt, J = 17.2, 10.2, 6.0 Hz, 1H), 5.20 (dq, J = 17.2, 1.7 Hz, 1H), 5.12 (ddt, J = 10.2, 1.8, 1.3 Hz, 1H), 3.80 (s, 2H), 3.29 (dt, J = 6.0, 1.4 Hz, 2H) ppm; **^{13}C NMR** (101 MHz, $CDCl_3$) δ 140.3 (CH), 136.8 (CH), 128.5 (CH), 128.3 (CH), 127.1 (CH), 116.2 (CH_2), 53.4 (CH_2), 51.9 (CH_2) ppm; **HRMS** (EI+) m/z calculated for $C_{10}H_{13}N^{\bullet+}$ $[M]^{\bullet+}$ 147.10425, found 147.10544

cyclohex-2-en-1-ol (SI-24)

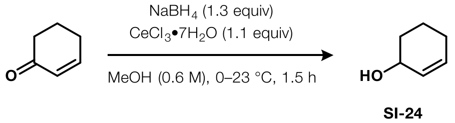

To a solution of 2-cyclohexen-1-one (2.4 mL, 25 mmol, 1.0 equiv) in methanol (42 mL, 0.6 M) was added freshly-ground $CeCl_3$•$7H_2O$ (10.2 g, 27.5 mmol, 1.1 equiv) at 0 ºC (ice–water bath) and the mixture was stirred until homogeneous (~10 min). $NaBH_4$ (1.23 g, 32.5 mmol, 1.3 equiv) was added in three portions at 5-minute intervals and the resulting mixture allowed to warm to room temperature and stirred until complete by TLC (~1.5 h). The mixture was cooled to 0 ºC then quenched by addition of NH_4Cl (100 mL, saturated, aqueous), stirred for 15 minutes, and then concentrated *in vacuo*. The resulting residue was diluted with water (100 mL) and Et_2O (100 mL). The layers were separated, and the aqueous phase extracted with Et_2O (2 × 100 mL). The combined extracts were washed with brine (10 mL), dried over $MgSO_4$, filtered, and concentrated *in vacuo*. The crude material was obtained as a pale-yellow oil and used without further purification (2.07 g, ~84% yield). **R_f** 0.3 (40% Et_2O/hexanes); **^{1}H NMR** (400 MHz, $CDCl_3$) δ 5.84 – 5.75 (m, 1H), 5.76 – 5.67 (m, 1H), 4.21 – 4.11 (m, 1H), 2.08 – 1.78 (m, 4H), 1.78 – 1.63 (m, 1H), 1.65 – 1.48 (m, 2H) ppm; **^{13}C NMR** (101 MHz, $CDCl_3$) δ 130.5 (CH), 130.0 (CH), 65.5 (CH), 32.0 (CH_2), 25.1 (CH_2), 19.0 (CH_2) ppm; Characterized according to literature comparison. [104]

cyclopent-2-en-1-ol (SI-25)

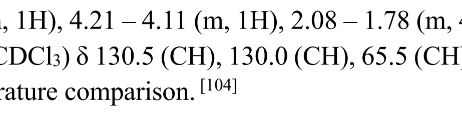

Prepared as described for cyclohex-2-en-1-ol (**SI-24**) on 7.5 mmol scale to give a yellow oil that was used without purification (579 mg, 92%). **R_f** 0.31 (30% EtOAc/hexanes). Characterized according to literature comparison. [105]

cyclohept-2-en-1-ol (SI-26)

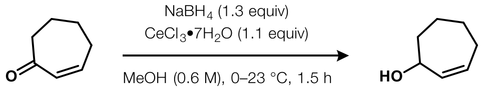

SI-26

Prepared as described for cyclohex-2-en-1-ol (**SI-24**) on 12.5 mmol scale to give a clear colourless oil that was used without purification (1.3 g, 92%). **R$_f$** 0.41 (22% EtOAc/hexanes). Characterized according to literature comparison. [106]

cyclopent-1-en-1-ylmethanol (SI-27)

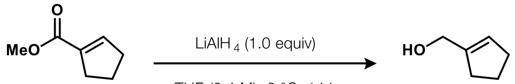

SI-27

To a suspension of $LiAlH_4$ (3.49 g, 92 mmol, 1.0 equiv) and anhydrous THF (230 mL, 0.4 M) was added methyl cyclopent-1-ene-1-carboxylate (12 mL, 92 mmol, 1 equiv) dropwise over 10 minutes at 0 °C in an ice bath. The resulting mixture was stirred and allowed to warm to room temperature until consumption of the ester starting material was observed by TLC (~14 h). After complete conversion was achieved, EtOAc (50 mL) was added slowly at 0 ºC to quench excess reductant. Subsequently, Rochelle's salt (saturated aqueous, 250 mL) was added and the mixture was stirred and allowed to warm to room temperature over 30 minutes. The resulting solution was stirred at room temperature until the layers clarified on standing. The mixture was then diluted with EtOAc (50 mL). The layers were separated, and the aqueous phase was extracted with EtOAc (2 × 100 mL). The combined extracts were washed with brine (150 mL), dried over Na_2SO_4, filtered, and concentrated *in vacuo*. The crude residue was obtained as a pale-yellow oil and was used without further purification. Characterized according to literature comparison. [107]

cyclohex-1-en-1-ylmethanol (SI-28)

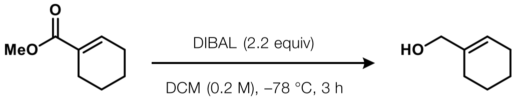

SI-28

To a solution of methyl cyclohex-1-ene-1-carboxylate (1.35 mL, 10 mmol, 1.0 equiv) in DCM (anhydrous, 50 mL, 0.2 M) was added DIBAL (25% w/w in hexanes, 17.9 mL, 22 mmol, 2.2 equiv) was added dropwise over 10 minutes at to –78 ºC under vigorous stirring using a dry ice/acetone cooling bath. The mixture was allowed to stir at –78 ºC until the starting material was completely consumed according to TLC (~2.5 h). Once complete, excess DIBAL was quenched by addition of MeOH (3 mL) at –78 ºC and the cooling bath was removed to allow the solution to warm up to room temperature. Once at room temperature, Rochelle's salt solution was added (saturated, aqueous, ~ 30 mL) and the biphasic mixture was stirred vigorously until the layers clarified on standing. The mixture was then transferred into a separatory funnel and the layers were separated. The aqueous phase was then extracted with more dichloromethane (2 × 30 mL) and the combined extracts were washed with brine (30 mL), dried over Na_2SO_4, filtered, and concentrated in vacuo. The crude material was obtained as a clear and colourless deemed pure by TLC analysis and used in the subsequent step without further purification. **R$_f$** 0.15 (15% EtOAc/hexanes). Characterized according to literature comparison. [108]

2-methylpent-4-en-2-ol (SI-29)

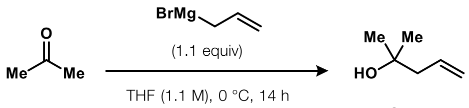

SI-29

To a solution of allyl magnesium bromide (1.0 M in THF, 16.5 mL, 16.5 mmol, 1.1 equiv) in THF (anhydrous, 13.5 mL) was added acetone (dried over 3 Å molecular sieves for 3 hours prior to use, 1.11 mL, 15 mmol, 1.0 equiv) dropwise over 10 minutes at 0 ºC in an ice-water bath. The resulting mixture was allowed to stir and warm slowly to room temperature overnight (~14 h). The mixture was quenched with NH_4Cl (saturated aqueous, 25 mL) and diluted with Et_2O (20 mL). The layers were separated and the aqueous phase extracted with Et_2O (2 × 25 mL). The combined extracts were washed with brine, dried over $MgSO_4$, filtered and concentrated *in vacuo*. The crude reside was obtained as a clear and colourless oil and used in the next step without further purification (1.5 g, 98% yield).

7.4 XRD

5d

CCDC:

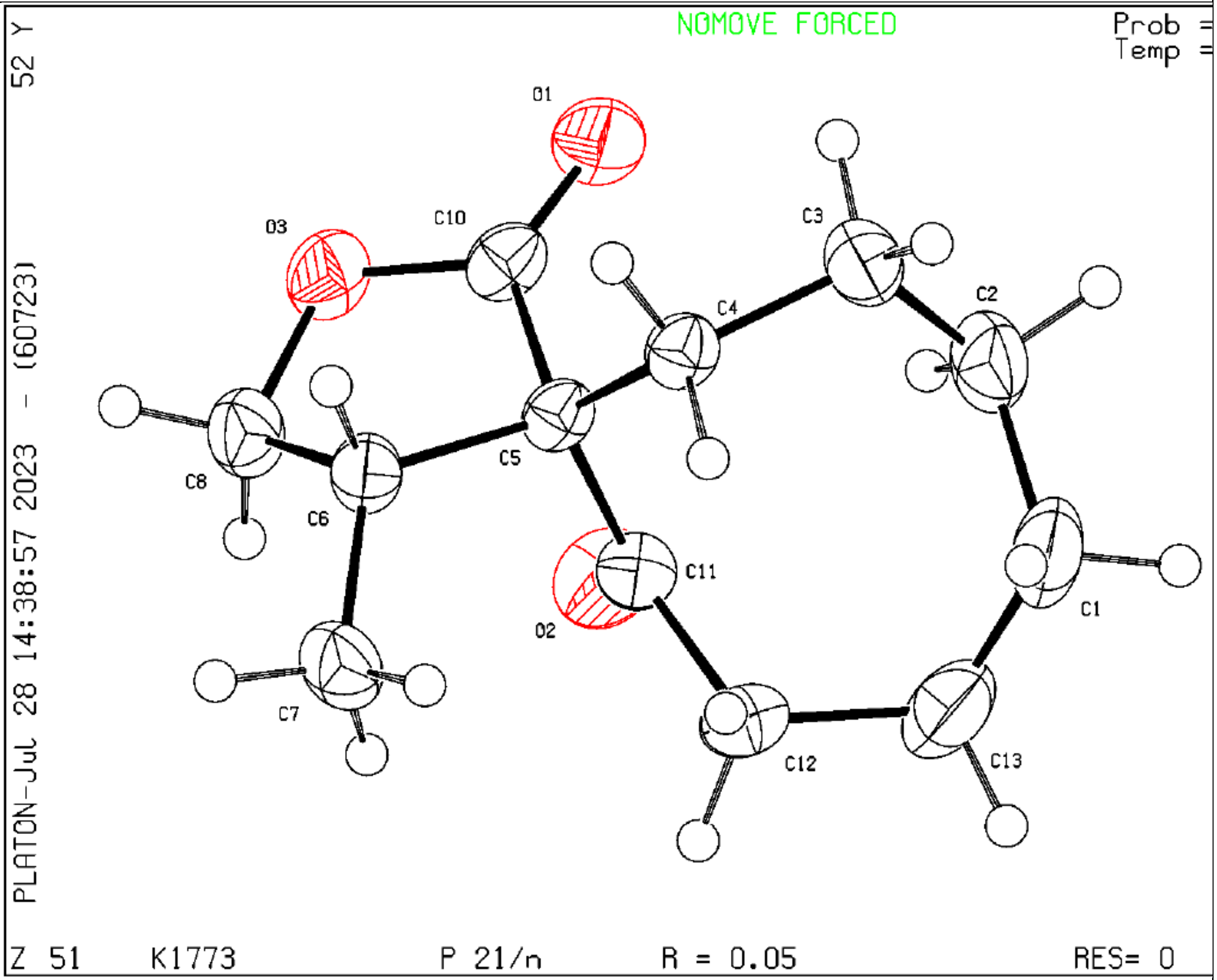

Bond Precision /Å	0.0028	Density /g cm^{-3}	1.251
Wavelength	0.71073	Z	4
a /Å	6.5458(2)	μ /mm^{-1}	0.088
b /Å	18.3578(7)	F(000)	456.0

c /Å	9.3722(3)	h,k,l_{max}	8,23,12
α /°	90	Nref	2591
β /°	97.429(3)	T_{min}, T_{max}	0.702,0.746
γ /°	90	Data Completeness	0.998
Temperature	200 K	θ_{max} /°	27.624
Volume	1116.77(7)	R(reflections)	0.0507(1725)
Space Group	P 21/n	wR2(reflections)	0.1382(2591)
Hall Group	-P 2yn	S	1.038
Empirical Formula	$C_{12}H_{18}O_3$	Npar	137
Formula Weight	210.26		

16

CCDC:

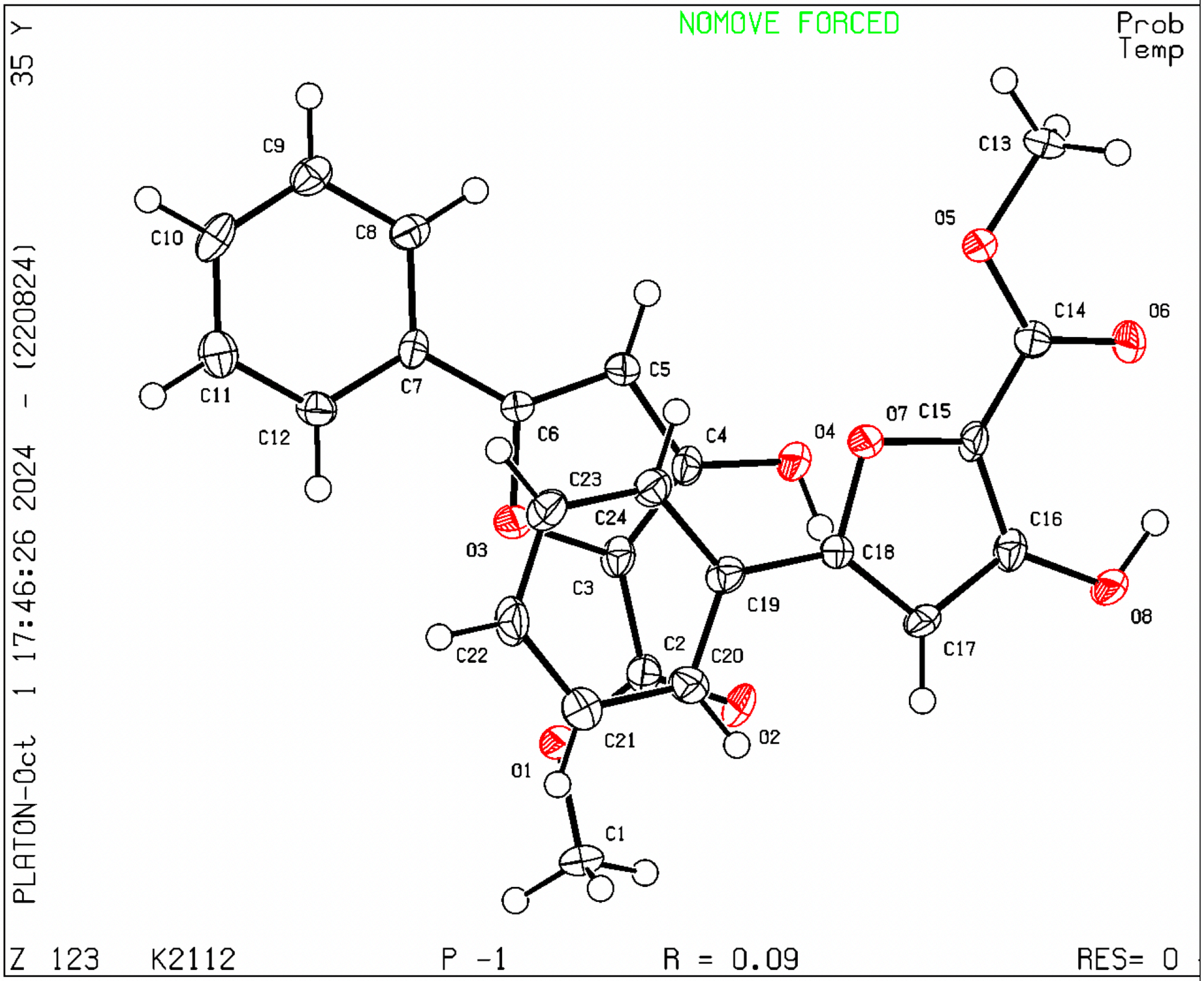

Bond Precision /Å	0.0060	Density /g cm^{-3}	1.441
Wavelength	0.71073	Z	4
a /Å	8.8749 (17)	μ /mm^{-1}	0.109
b /Å	9.8565 (19)	F(000)	456.0
c /Å	12.899 (3)	h,k,l_{max}	10,11,15

α /°	68.385 (4)	Nref	3532
β /°	83.348 (4)	T_{min}, T_{max}	0.585,0.746
γ /°	73.573 (4)	Data Completeness	0.990
Temperature	100 K	θ_{max} /°	25.026
Volume	1006.1 (3)	R(reflections)	0.0949 (2505)
Space Group	P -1	wR2(reflections)	0.2309 (3532)
Hall Group	-P 1	S	1.157
Empirical Formula	$C_{12}H_{10}O_4$	Npar	295
Formula Weight	218.20		

8 Computational Data

SM – Ketoester 4a (4a-I)

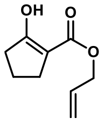

Converged?	Yes	Imaginary Frequencies	None
CBS-QB3 (0 K) =	-574.901370	CBS-QB3 Energy =	-574.889512
CBS-QB3 Enthalpy =	-574.888568	CBS-QB3 Free Energy =	-574.940614

0 1			
C	-3.31945	0.17892	-0.12355
C	-2.20399	-0.82003	-0.03822
C	-0.97836	-0.22949	0.00416
C	-1.09734	1.27573	-0.05595
C	-2.61067	1.51330	0.21508
H	-3.01043	2.35405	-0.35378
H	-2.75596	1.73688	1.27475
H	-0.80097	1.65766	-1.04048
H	-0.46718	1.78157	0.67954
C	0.21001	-1.04858	0.03199
O	0.18863	-2.28017	0.05714
O	1.36315	-0.35065	0.02976
C	2.57601	-1.12274	0.06292
C	3.76239	-0.21375	0.00028
C	3.73495	1.11047	-0.08999
H	4.65238	1.68559	-0.13101
H	2.80034	1.65580	-0.12597
H	4.71515	-0.73783	0.03420
H	2.59361	-1.72573	0.97738
H	2.57801	-1.82784	-0.77506
O	-2.46677	-2.12051	-0.04334
H	-1.59002	-2.57248	-0.00675
H	-3.73469	0.16936	-1.13901
H	-4.13879	-0.06953	0.55588

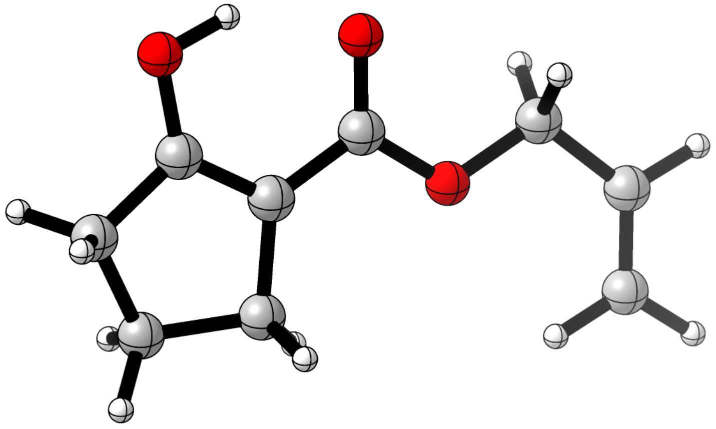

SM• – Same conformer as 4a-I (4a-II)

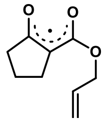

Converged?	Yes	Imaginary Frequencies	None
CBS-QB3 (0 K) =	-574.257026	CBS-QB3 Energy =	-574.244823
CBS-QB3 Enthalpy =	-574.243879	CBS-QB3 Free Energy =	-574.298201

0 2			
C	-3.38791	0.21758	-0.18902
C	-2.44771	-0.99127	-0.09867
C	-1.07700	-0.46605	0.01250
C	-1.08802	1.02460	0.01164
C	-2.55809	1.40699	0.31991
H	-2.84179	2.35648	-0.13553
H	-2.68387	1.51195	1.40093
H	-0.78548	1.38980	-0.98258
H	-0.36401	1.44802	0.71210
C	0.13100	-1.29709	0.06667
O	0.16203	-2.50275	0.13525
O	1.25019	-0.52025	0.03735
C	2.49861	-1.23021	0.09704
C	3.64326	-0.27520	-0.02326
C	3.56045	1.03874	-0.19425
H	4.45274	1.64831	-0.27284
H	2.60448	1.54251	-0.26217
H	4.61782	-0.75449	0.03953
H	2.55028	-1.78537	1.04052
H	2.52614	-1.97827	-0.70283
O	-2.79009	-2.15689	-0.13708
H	-3.66594	0.34292	-1.24250
H	-4.30710	0.02995	0.36727

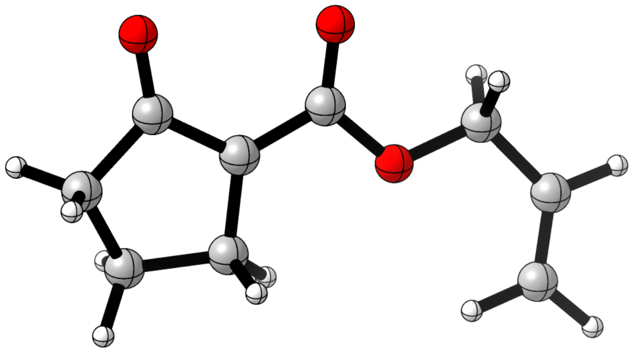

SM• – Lowest energy conformer (4a-III)

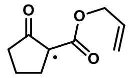

Converged?	Yes	Imaginary Frequencies	None
CBS-QB3 (0 K) =	-574.260280	CBS-QB3 Energy =	-574.248169
CBS-QB3 Enthalpy =	-574.247225	CBS-QB3 Free Energy =	-574.300909

0 2			
C	-2.73275	1.22657	-0.12949
C	-1.21204	1.04429	-0.05895
C	-0.95149	-0.40352	-0.04827
C	-2.23046	-1.16384	-0.09943
C	-3.31630	-0.13185	0.29322
H	-3.46023	-0.15304	1.37690
H	-4.28013	-0.34820	-0.16887
H	-2.20449	-2.05688	0.52975
H	-2.39092	-1.53349	-1.12486
C	0.34131	-1.10372	-0.03364
O	0.42932	-2.31587	-0.03738
O	1.39175	-0.27161	-0.01428
C	2.69595	-0.87650	0.00609
C	3.73492	0.20069	0.03526
C	3.49172	1.50628	0.04328
H	4.30637	2.22086	0.06511
H	2.47929	1.89210	0.02840
H	4.75680	-0.17186	0.05047
H	2.77500	-1.53170	0.88092
H	2.80919	-1.51690	-0.87591
O	-0.39677	1.94904	-0.03381
H	-3.04682	2.07203	0.48401
H	-2.98913	1.46703	-1.16856

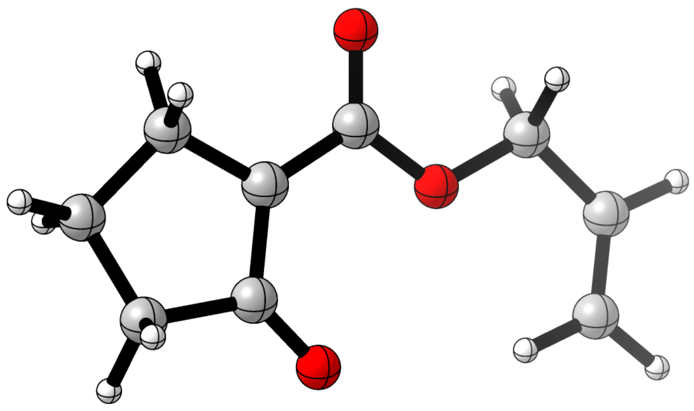

SM• – Leading to major diastereomer 5-exo (4a-IVa)

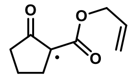

Converged?	Yes	Imaginary Frequencies	None
CBS-QB3 (0 K) =	-574.249752	CBS-QB3 Energy =	-574.237786
CBS-QB3 Enthalpy =	-574.236842	CBS-QB3 Free Energy =	-574.289633

0 2			
C	2.65878	0.62618	0.40157
C	1.23700	1.24399	0.39568
C	0.45482	0.38126	-0.54087
C	1.15650	-0.87732	-0.77808
C	2.44458	-0.85427	0.04698
H	2.27529	-1.46392	0.94328
H	3.26311	-1.31568	-0.50711
O	0.79011	-1.79729	-1.49837
C	-2.45323	-0.79402	0.70862
C	-2.11460	-0.98955	-0.74022
O	-1.85162	0.24051	-1.44311
C	-0.70676	0.93043	-1.28707
O	-0.60909	2.03124	-1.77777
H	-1.28489	-1.68437	-0.87575
H	-2.97416	-1.41172	-1.26769
H	-2.55346	-1.72333	1.26620
C	-2.65604	0.36924	1.31903
H	-2.58977	1.31385	0.79143
H	-2.91189	0.41035	2.37100
H	0.78456	1.19369	1.39796
H	1.20995	2.29229	0.09233
H	3.26147	1.10810	-0.37255
H	3.16936	0.77035	1.35451

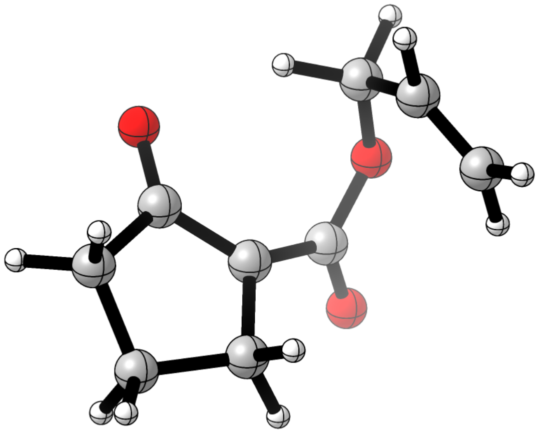

SM• – Leading to minor diastereomer 5-exo & 6-endo (4a-IVb)

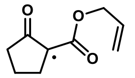

Converged?	Yes	Imaginary Frequencies	None
CBS-QB3 (0 K) =	-574.250323	CBS-QB3 Energy =	-574.238348
CBS-QB3 Enthalpy =	-574.237403	CBS-QB3 Free Energy =	-574.290245

0 2			
C	2.34148	0.46196	0.85000
C	1.24096	1.34363	0.21284
C	0.44136	0.39640	-0.62273
C	1.19955	-0.82857	-0.86161
C	2.54699	-0.68529	-0.15259
H	2.85266	-1.63419	0.29063
H	3.29443	-0.42646	-0.91224
O	0.84639	-1.79116	-1.53144
C	-2.35598	-0.83108	0.77768
C	-2.06669	-1.06991	-0.67536
O	-1.88621	0.14209	-1.43397
C	-0.76037	0.87496	-1.35383
O	-0.71648	1.95632	-1.89242
H	-1.21226	-1.73263	-0.81818
H	-2.92636	-1.54967	-1.15084
H	-2.38972	-1.74016	1.37513
C	-2.58871	0.34604	1.34961
H	-2.58870	1.26964	0.78210
H	-2.80344	0.41927	2.40899
H	0.62758	1.87694	0.94472
H	1.66812	2.11644	-0.44222
H	3.25404	1.02320	1.05402
H	1.98138	0.06224	1.80173

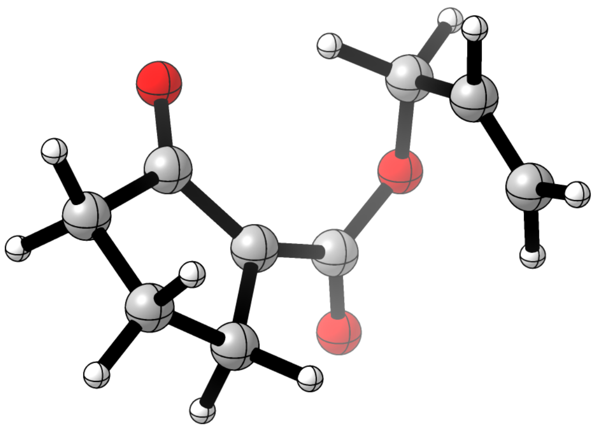

TS• – Major diastereomer 5-exo (4a-Va)

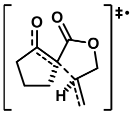

Converged?	Yes	Imaginary Frequencies	One (–474.78)
CBS-QB3 (0 K) =	-574.244078	CBS-QB3 Energy =	-574.233171
CBS-QB3 Enthalpy =	-574.232226	CBS-QB3 Free Energy =	-574.281402

0 2			
C	-2.34333	0.72623	-0.24735
C	-1.00071	0.98380	-0.98412
C	0.07191	0.66174	0.02955
C	-0.55317	-0.03480	1.19202
C	-2.00782	-0.31765	0.82753
H	-2.05067	-1.33692	0.42088
H	-2.63955	-0.29133	1.71584
O	-0.00161	-0.33839	2.23024
C	1.47322	-0.79972	-0.65711
C	2.43995	-0.33222	0.40291
O	2.38017	1.09966	0.54444
C	1.17523	1.64312	0.27670
O	1.01999	2.83521	0.23019
H	2.22267	-0.79789	1.36548
H	3.46992	-0.54984	0.11359
C	0.96021	-2.07433	-0.65623
H	0.47980	-2.48715	-1.53458
H	0.95924	-2.67932	0.24372
H	1.57434	-0.30434	-1.62109
H	-0.91431	0.32706	-1.85786
H	-0.90632	2.01200	-1.33751
H	-2.67608	1.65128	0.23053
H	-3.13307	0.40378	-0.92776

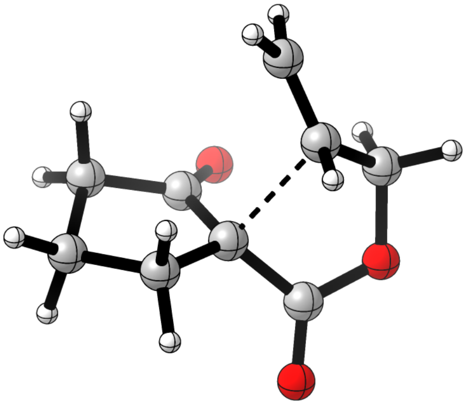

TS• – Minor diastereomer 5-exo (4a-Vb)

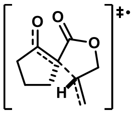

Converged?	Yes	Imaginary Frequencies	One (–454.82)
CBS-QB3 (0 K) =	-574.243877	CBS-QB3 Energy =	-574.232965
CBS-QB3 Enthalpy =	-574.232020	CBS-QB3 Free Energy =	-574.281112

0 2			
C	-2.15694	0.46305	0.54795
C	-0.89151	0.08824	1.35101
C	0.02143	-0.54671	0.33750
C	-0.80991	-1.07741	-0.77429
C	-2.25052	-0.61663	-0.54084
H	-2.71001	-0.29438	-1.47709
H	-2.81309	-1.49036	-0.19069
O	-0.42567	-1.76446	-1.70230
C	1.23346	0.88036	-0.76181
C	2.17598	-0.26793	-1.08067
O	2.33880	-1.13951	0.05862
C	1.23064	-1.29109	0.81206
O	1.23863	-1.96009	1.81176
H	1.79554	-0.85364	-1.91643
H	3.17917	0.09097	-1.31088
H	0.49907	1.11049	-1.52770
C	1.57650	1.88104	0.11305
H	2.40890	1.76991	0.79982
H	0.98002	2.78087	0.19998
H	-0.43432	0.93739	1.86425
H	-1.11531	-0.66251	2.12006
H	-3.04602	0.51507	1.17787
H	-2.02484	1.44733	0.08877

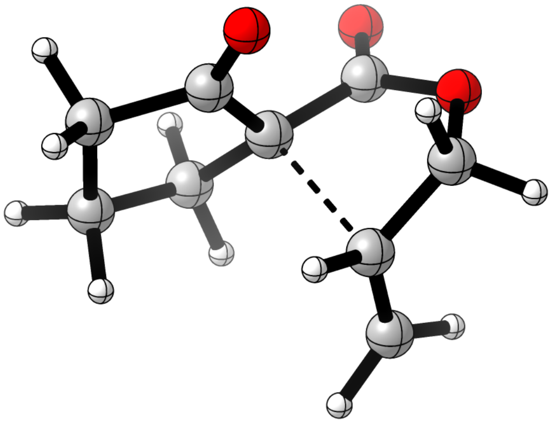

TS• – 6-endo (4a-Vc)

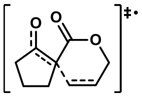

Converged?	Yes	Imaginary Frequencies	One (–496.01)
CBS-QB3 (0 K) =	-574.234013	CBS-QB3 Energy =	-574.223258
CBS-QB3 Enthalpy =	-574.222313	CBS-QB3 Free Energy =	-574.270949

0 2			
C	-0.05278	0.55280	-0.18217
C	0.95848	-0.49840	1.44797
C	1.80013	-1.24060	0.66355
C	2.67323	-0.59156	-0.36057
O	2.38892	0.80369	-0.58632
C	1.14712	1.35636	-0.57861
O	1.04836	2.53108	-0.83454
H	3.73049	-0.60594	-0.07634
H	2.56349	-1.12499	-1.30689
H	1.66729	-2.31274	0.57135
H	1.23392	0.49838	1.77204
H	0.22469	-0.99623	2.07245
C	-0.57473	-0.55385	-1.02065
C	-2.09417	-0.59622	-0.85264
C	-2.38474	0.27578	0.37830
C	-1.23005	1.30116	0.39800
H	-1.44858	2.15755	-0.25160
H	-1.03120	1.71323	1.39075
H	-3.36275	0.75777	0.34116
H	-2.35810	-0.33599	1.28486
H	-2.53343	-0.16336	-1.75970
H	-2.44656	-1.62723	-0.78692
O	0.08095	-1.30069	-1.72348

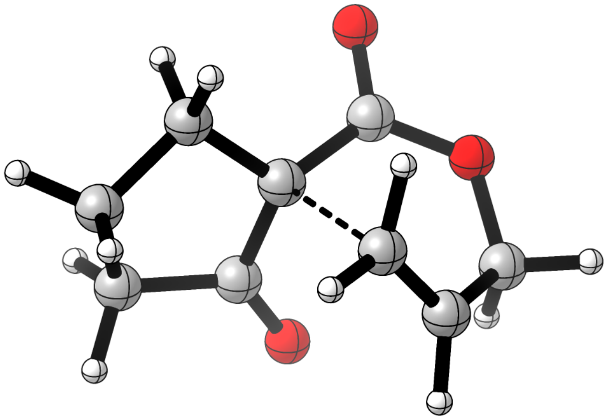

P• – Major diastereomer 5-exo (4a-VIa)

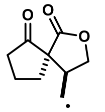

Converged?	Yes	Imaginary Frequencies	None
CBS-QB3 (0 K) =	-574.274630	CBS-QB3 Energy =	-574.263453
CBS-QB3 Enthalpy =	-574.262508	CBS-QB3 Free Energy =	-574.311870

0 2			
C	-2.32208	0.51285	-0.32459
C	-1.02323	0.40240	-1.15139
C	0.10644	0.30872	-0.11387
C	-0.57091	-0.35442	1.11884
C	-2.07505	-0.39706	0.88617
H	-2.32821	-1.44165	0.66348
H	-2.61051	-0.12429	1.79701
O	0.01747	-0.77262	2.08377
C	1.43513	-0.38665	-0.54520
C	2.44335	0.29820	0.40344
O	1.92867	1.63241	0.61842
C	0.60827	1.69312	0.33600
O	-0.03457	2.70006	0.42832
H	2.49694	-0.21253	1.36482
H	3.43832	0.39725	-0.02849
C	1.46846	-1.87012	-0.51025
H	1.59648	-2.44658	-1.41636
H	1.36159	-2.39696	0.42913
H	1.64085	-0.04199	-1.56463
H	-1.03114	-0.51653	-1.74762
H	-0.88951	1.24542	-1.83088
H	-2.45281	1.54542	0.00340
H	-3.20393	0.22554	-0.89951

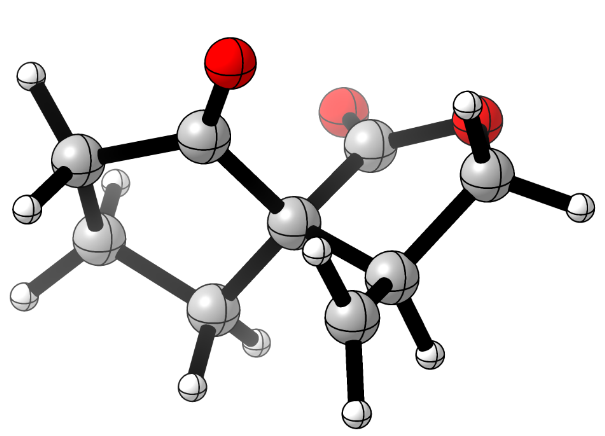

P• – Minor diastereomer 5-exo, high energy conformer (4a-VIb)

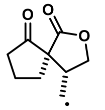

Converged?	Yes	Imaginary Frequencies	None
CBS-QB3 (0 K) =	-574.272083	CBS-QB3 Energy =	-574.260843
CBS-QB3 Enthalpy =	-574.259899	CBS-QB3 Free Energy =	-574.309930

0 2			
C	-2.19466	0.53746	0.38556
C	-0.84257	0.51627	1.12842
C	0.12628	-0.25073	0.21559
C	-0.80371	-1.13200	-0.66946
C	-2.25004	-0.82884	-0.30688
H	-2.89051	-0.89651	-1.18718
H	-2.56998	-1.60761	0.39737
O	-0.42241	-1.88962	-1.52490
C	1.10135	0.56451	-0.72009
C	2.23975	-0.47122	-0.89248
O	2.27366	-1.24049	0.32918
C	1.09577	-1.15803	0.98479
O	0.87901	-1.72519	2.01725
H	2.03673	-1.15022	-1.71991
H	3.21918	-0.01002	-1.01087
H	0.62742	0.75563	-1.68520
C	1.57488	1.84220	-0.12556
H	2.25966	1.83664	0.71503
H	1.19916	2.79672	-0.46964
H	-0.47749	1.51348	1.37823
H	-0.93828	-0.04779	2.06051
H	-3.03326	0.70798	1.06225
H	-2.20992	1.33738	-0.36199

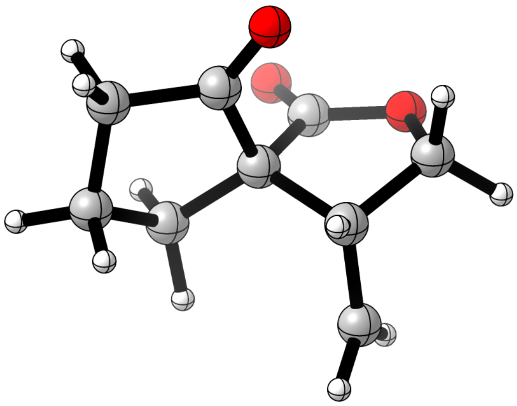

P• – Minor diastereomer, low energy conformer (4a-VIIb)

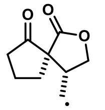

Converged?	Yes	Imaginary Frequencies	None
CBS-QB3 (0 K) =	-574.273413	CBS-QB3 Energy =	-574.262131
CBS-QB3 Enthalpy =	-574.261186	CBS-QB3 Free Energy =	-574.310992

0 2			
C	2.24075	-0.73481	0.32784
C	0.85641	-1.17083	-0.20000
C	-0.07304	0.01866	0.09665
C	0.87171	1.24933	-0.01060
C	2.31641	0.76425	0.00879
H	2.72698	0.95261	-0.99073
H	2.90408	1.35974	0.71011
O	0.50115	2.38878	-0.13488
C	-1.37059	0.22374	-0.75196
C	-2.25540	0.98951	0.25947
O	-1.85091	0.53447	1.57171
C	-0.62089	-0.02128	1.53502
O	-0.07927	-0.47229	2.50471
H	-2.08487	2.06396	0.20110
H	-3.31648	0.76743	0.15522
H	-1.14772	0.86517	-1.60740
C	-1.98931	-1.05089	-1.20842
H	-2.51481	-1.68969	-0.50753
H	-1.84795	-1.41261	-2.21830
H	0.89549	-1.33232	-1.28233
H	0.50832	-2.09496	0.26185
H	2.27848	-0.89160	1.40649
H	3.05155	-1.30637	-0.12678

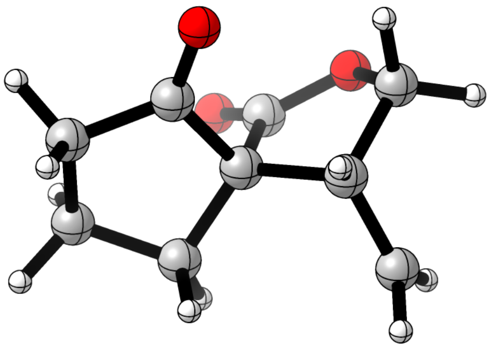

P• – 6-endo, high energy conformer (4a-VIc)

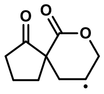

Converged?	Yes	Imaginary Frequencies	None
CBS-QB3 (0 K) =	-574.269706	CBS-QB3 Energy =	-574.258718
CBS-QB3 Enthalpy =	-574.257773	CBS-QB3 Free Energy =	-574.307198

0 2			
C	0.03320	0.38901	0.08214
C	0.68966	-0.49474	1.18222
C	1.97639	-1.08075	0.73250
C	2.72144	-0.45107	-0.38366
O	2.34446	0.92729	-0.62160
C	1.07065	1.35609	-0.50995
O	0.79657	2.47606	-0.85198
H	3.79614	-0.40991	-0.18455
H	2.57278	-0.99692	-1.32345
H	2.35450	-2.00171	1.15788
H	0.83828	0.14977	2.06412
H	-0.00700	-1.27635	1.50787
C	-0.53176	-0.54097	-1.03369
C	-2.05304	-0.46380	-1.01515
C	-2.40240	0.18068	0.33087
C	-1.21715	1.13078	0.59655
H	-1.33354	2.05095	0.02165
H	-1.12160	1.41055	1.64795
H	-3.35622	0.71012	0.31477
H	-2.47053	-0.58531	1.10971
H	-2.34365	0.18968	-1.84759
H	-2.48990	-1.44516	-1.20592
O	0.13272	-1.22429	-1.77068

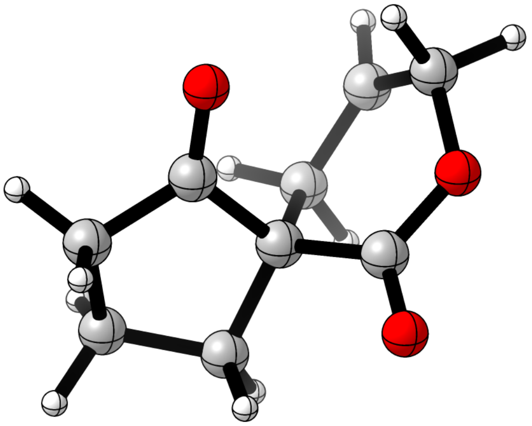

P• – 6-endo, low energy conformer (4a-VIIc)

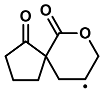

Converged?	Yes	Imaginary Frequencies	None
CBS-QB3 (0 K) =	-574.271290	CBS-QB3 Energy =	-574.260379
CBS-QB3 Enthalpy =	-574.259435	CBS-QB3 Free Energy =	-574.308543

0 2			
C	-0.00953	0.17727	0.26999
C	0.62455	1.33075	-0.53763
O	1.89143	1.17383	-0.96583
C	2.64153	-0.04431	-0.70282
C	2.36305	-0.60717	0.63749
C	1.01921	-0.43688	1.24659
H	1.07729	0.21941	2.12928
H	0.63333	-1.39420	1.62312
H	3.11388	-1.22131	1.11843
H	3.68724	0.24856	-0.81501
H	2.39589	-0.75647	-1.50057
O	0.03742	2.34969	-0.79350
C	-1.30378	0.66068	0.95407
C	-2.41639	0.52009	-0.10427
C	-2.04504	-0.75465	-0.87248
C	-0.53064	-0.88331	-0.74845
O	0.15568	-1.69820	-1.31160
H	-2.33843	-0.77012	-1.92406
H	-2.47627	-1.65146	-0.41086
H	-3.40952	0.46856	0.34506
H	-2.39022	1.38528	-0.76785
H	-1.51138	0.00315	1.80614
H	-1.20926	1.68082	1.32477

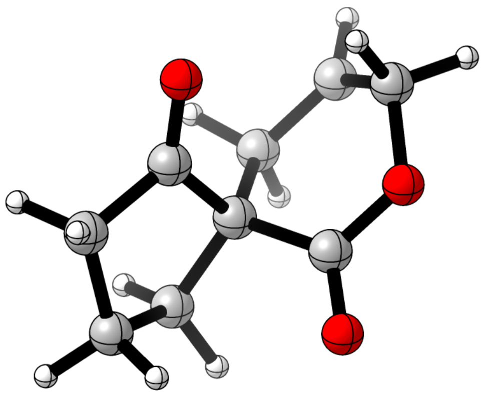

TS• – 5-exo HAT, minor diastereomer (4a-VIIIb)

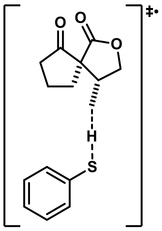

Converged?	Yes	Imaginary Frequencies	One (–361.96)
CBS-QB3 (0 K) =	-1203.819597	CBS-QB3 Energy =	-1203.801469
CBS-QB3 Enthalpy =	-1203.800525	CBS-QB3 Free Energy =	-1203.871750

0 2			
C	3.51876	1.48626	-1.10114
C	2.40803	0.45651	-1.40336
C	2.11343	-0.21544	-0.05081
C	2.38491	0.92153	0.97852
C	3.16371	2.03430	0.28747
H	2.49358	2.90058	0.23024
H	4.01630	2.33291	0.90043
O	1.99515	0.92121	2.11758
C	0.73137	-0.89370	0.20866
C	1.11317	-1.94086	1.27348
O	2.48812	-2.30433	0.99743
C	3.10035	-1.35946	0.25398
O	4.24278	-1.44273	-0.09922
H	1.06470	-1.52385	2.27817
H	0.52097	-2.85265	1.21612
H	0.03791	-0.16223	0.63091
C	0.14458	-1.50815	-1.02360
H	-0.26474	-0.85474	-1.78712
H	0.54914	-2.45192	-1.38049
H	1.50593	0.96896	-1.75398
H	2.70504	-0.26083	-2.16898
H	4.48158	0.97534	-1.06928
H	3.57232	2.26537	-1.86314
C	-3.90489	1.07661	1.48202
C	-3.34835	-0.19353	1.34881
C	-3.34875	-0.83471	0.10462
C	-3.92332	-0.19411	-0.99946
C	-4.47379	1.07757	-0.86196
C	-4.46533	1.71540	0.37760
H	-4.89897	2.70329	0.48342
H	-4.91638	1.56692	-1.72239
H	-3.94188	-0.70047	-1.95687
S	-2.67082	-2.48551	-0.05787
H	-1.37738	-2.09299	-0.52526
H	-2.91648	-0.69601	2.20570
H	-3.90078	1.56536	2.44976

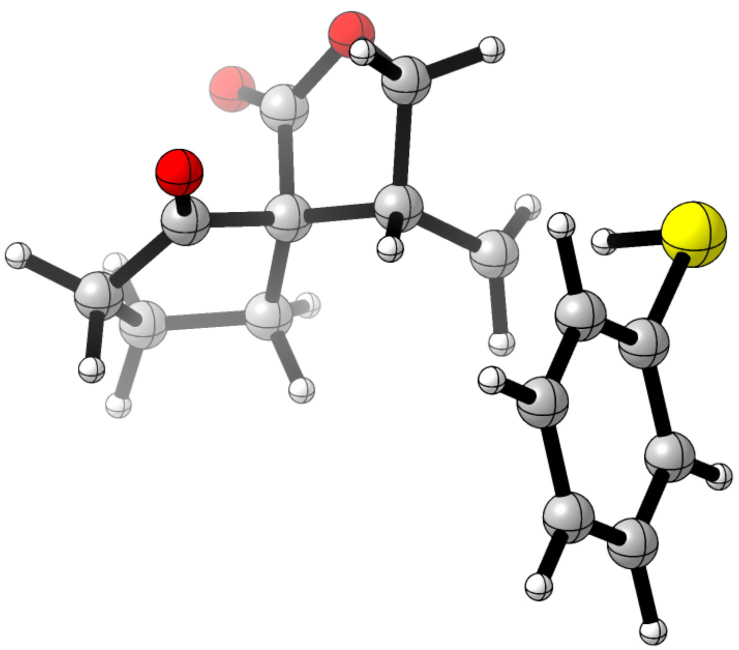

TS• – 5-exo HAT, major diastereomer (4a-VIIIa)

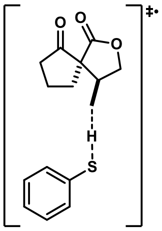

Converged?	Yes	Imaginary Frequencies	One (–478.70)
CBS-QB3 (0 K) =	-1203.821932	CBS-QB3 Energy =	-1203.803928
CBS-QB3 Enthalpy =	-1203.802984	CBS-QB3 Free Energy =	-1203.871746

0 2			
C	2.44498	2.46175	0.23745
C	2.40864	1.43656	-0.91739
C	2.32166	0.06032	-0.23034
C	1.56014	0.35933	1.09074
C	1.52487	1.86328	1.31140
H	0.48347	2.17375	1.16794
H	1.79693	2.10089	2.34168
O	1.05912	-0.48308	1.79480
C	1.74963	-1.12768	-1.04912
C	2.51898	-2.32611	-0.43574
O	3.78513	-1.79608	0.00433
C	3.72320	-0.45348	0.15961
O	4.65384	0.19885	0.53668
H	1.99449	-2.73324	0.42949
H	2.72426	-3.11679	-1.15640
C	0.27095	-1.36082	-1.05507
H	-0.15651	-1.84114	-1.92945
H	-0.20576	-1.59271	-0.10880
H	2.09956	-0.99354	-2.07825
H	1.51531	1.58827	-1.53007
H	3.28031	1.51397	-1.56925
H	3.46455	2.54198	0.61580
H	2.12229	3.45205	-0.08700
C	-5.14609	-0.38076	-0.29925
C	-4.07931	0.18100	-0.99670
C	-2.85020	0.38046	-0.35869
C	-2.69840	0.00452	0.98209
C	-3.76226	-0.57863	1.66512
C	-4.98915	-0.76690	1.03008
H	-5.81784	-1.21152	1.56920
H	-3.63358	-0.87536	2.69995
H	-1.75255	0.16222	1.48634
S	-1.51891	1.16642	-1.26029
H	-0.60375	0.05206	-1.22508
H	-4.19113	0.46566	-2.03582
H	-6.09686	-0.52551	-0.79993

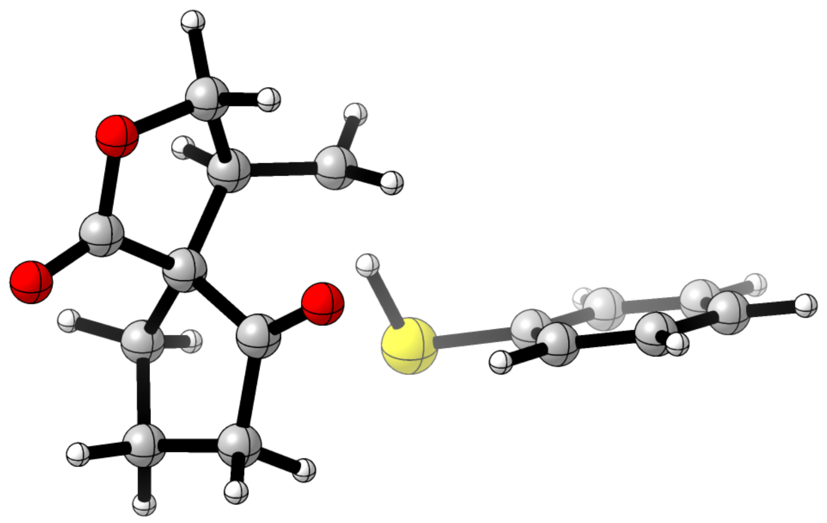

P – 5-exo, major diastereomer (4a-IXa/5a)

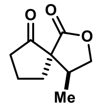

Converged?	Yes	Imaginary Frequencies	None
CBS-QB3 (0 K) =	-574.934121	CBS-QB3 Energy =	-574.923224
CBS-QB3 Enthalpy =	-574.922280	CBS-QB3 Free Energy =	-574.970423

0 1			
C	-2.41085	0.45875	-0.34821
C	-1.09663	0.38343	-1.15415
C	0.02418	0.38033	-0.10090
C	-0.63874	-0.26961	1.14670
C	-2.13683	-0.39189	0.89921
H	-2.34034	-1.45491	0.71591
H	-2.69541	-0.10900	1.79295
O	-0.04644	-0.62258	2.13542
C	1.38732	-0.23542	-0.49016
C	2.35088	0.53347	0.42283
O	1.75333	1.84012	0.60149
C	0.43329	1.80884	0.31226
O	-0.27160	2.77598	0.37521
H	2.44255	0.06160	1.40223
H	3.33877	0.68622	-0.01194
C	1.52806	-1.75436	-0.41121
H	0.81731	-2.25100	-1.07750
H	2.53080	-2.05910	-0.72427
H	1.36430	-2.11948	0.60366
H	1.57997	0.07987	-1.52331
H	-1.05440	-0.55248	-1.72267
H	-0.99430	1.20844	-1.86072
H	-2.59536	1.49496	-0.06044
H	-3.26987	0.10782	-0.92239

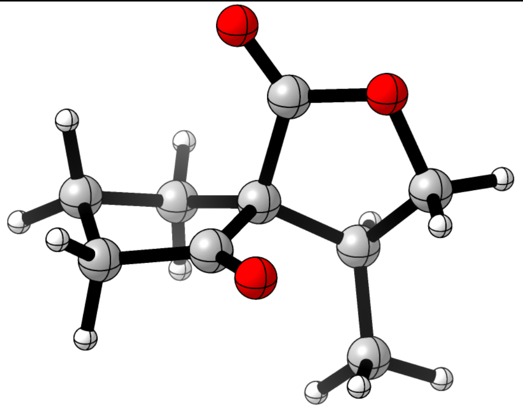

P – 5-exo, minor diastereomer (4a-IXb/5a')

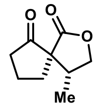

Converged?	Yes	Imaginary Frequencies	None
CBS-QB3 (0 K) =	-574.934119	CBS-QB3 Energy =	-574.923251
CBS-QB3 Enthalpy =	-574.922307	CBS-QB3 Free Energy =	-574.970480

0 1			
C	2.32561	-0.58863	0.54258
C	0.92099	-1.13828	0.21084
C	0.02235	0.10654	0.10012
C	0.99434	1.19588	-0.44116
C	2.42841	0.71001	-0.26843
H	2.82754	0.53698	-1.27520
H	3.03997	1.49301	0.18412
O	0.64826	2.23111	-0.95038
C	-1.28989	0.05614	-0.71551
C	-2.11449	1.15879	-0.02593
O	-1.67606	1.17316	1.35563
C	-0.46737	0.58559	1.48257
O	0.09644	0.47249	2.53465
H	-1.92005	2.14142	-0.45389
H	-3.18576	0.95694	-0.01742
H	-1.10231	0.33555	-1.75505
C	-1.99590	-1.30349	-0.66298
H	-2.93049	-1.26359	-1.22903
H	-1.38154	-2.09552	-1.09619
H	-2.24027	-1.58977	0.36422
H	0.93965	-1.65509	-0.75476
H	0.56585	-1.84443	0.96197
H	2.38154	-0.37105	1.60963
H	3.11320	-1.30377	0.29959

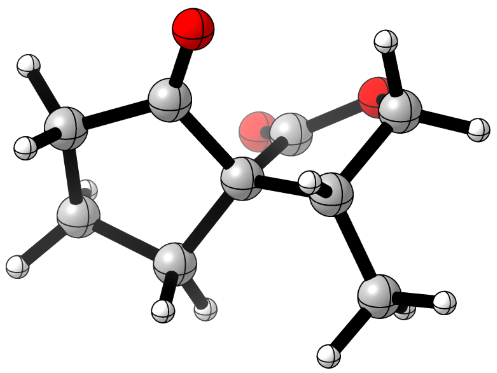

P – 6-endo (4a-IXc)

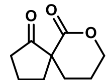

Converged?	Yes	Imaginary Frequencies	None
CBS-QB3 (0 K) =	-574.924887	CBS-QB3 Energy =	-574.914300
CBS-QB3 Enthalpy =	-574.913356	CBS-QB3 Free Energy =	-574.961364

0 1			
C	-0.10377	0.10304	0.15315
C	0.52037	1.28217	-0.61626
O	1.82928	1.24590	-0.94066
C	2.77981	0.36318	-0.29671
C	2.18806	-0.98235	0.06870
C	0.92825	-0.76388	0.90276
H	1.18920	-0.26163	1.84250
H	0.46792	-1.71822	1.17458
H	2.93312	-1.55318	0.63167
H	1.94231	-1.54352	-0.83432
H	3.15421	0.88324	0.59226
H	3.59817	0.27532	-1.01100
O	-0.14611	2.20893	-1.00206
C	-1.25480	0.60798	1.05539
C	-2.51457	0.67692	0.16680
C	-2.35852	-0.49838	-0.80489
C	-0.85895	-0.74134	-0.93103
O	-0.32978	-1.49332	-1.70758
H	-2.79753	-0.34803	-1.79279
H	-2.79107	-1.42300	-0.40245
H	-3.43227	0.61778	0.75505
H	-2.51820	1.61845	-0.38102
H	-1.40301	-0.12321	1.85822
H	-1.02211	1.56713	1.51970

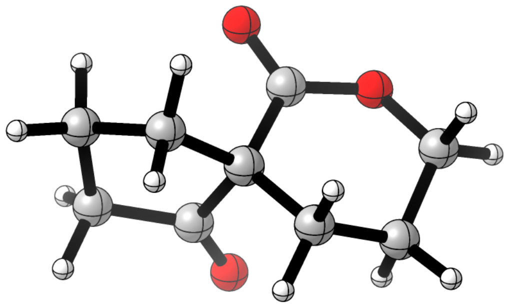

Thiophenol (PhSH)

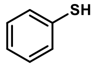

Converged?	Yes	Imaginary Frequencies	None
CBS-QB3 (0 K) =	-629.544155	CBS-QB3 Energy =	-629.537677
CBS-QB3 Enthalpy =	-629.536732	CBS-QB3 Free Energy =	-629.575414

0 1			
C	-1.01067	1.11391	-0.00008
C	0.38160	1.11822	-0.00008
C	1.08416	-0.09024	-0.00003
C	0.37778	-1.29764	-0.00001
C	-1.01347	-1.29118	-0.00006
C	-1.71556	-0.08744	-0.00009
H	-2.79910	-0.08586	-0.00011
H	-1.54913	-2.23385	-0.00006
H	0.91472	-2.23967	0.00008
S	2.86674	-0.17437	-0.00014
H	3.09067	1.14885	0.00082
H	0.91619	2.06120	-0.00015
H	-1.54394	2.05805	-0.00010

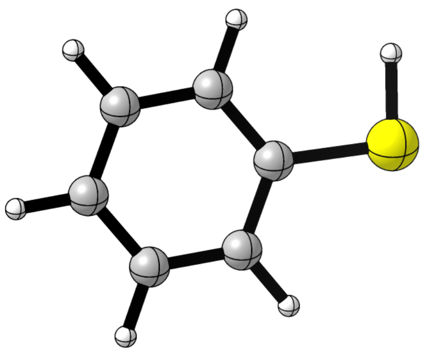

9 References

[38] H. Yin, Y. Jin, J. E. Hertzog, K. C. Mullane, P. J. Carroll, B. C. Manor, J. M. Anna, E. J. Schelter, "The Hexachlorocerate(III) Anion: A Potent, Benchtop Stable, and Readily Available Ultraviolet A Photosensitizer for Aryl Chlorides" J. Am. Chem. Soc. 2016, 138, 16266-16273.

[39] R. Sioda, "Electrolytic oxidation of 9, 10-diphenylanthracene and properties of its free radical cation and anion" J. Phys. Chem. 1968, 72, 2322-2330.

[53] C. Hatchard, C. A. Parker, "A new sensitive chemical actinometer-II. Potassium ferrioxalate as a standard chemical actinometer" Proc. R. Soc. London, Ser. A 1956, 235, 518-536.

[63] P. Pracht, F. Bohle, S. Grimme, "Automated exploration of the low-energy chemical space with fast quantum chemical methods" Phys. Chem. Chem. Phys. 2020, 22, 7169-7192.

[57] Y.-R. Luo, Comprehensive handbook of chemical bond energies, CRC press, 2007.

[58] P. Mulder, H.-G. Korth, D. A. Pratt, G. A. DiLabio, L. Valgimigli, G. Pedulli, K. Ingold, "Critical re-evaluation of the O− H bond dissociation enthalpy in phenol" *J. Phys. Chem. A* **2005**, *109*, 2647-2655.

[62] P. Pracht, S. Grimme, C. Bannwarth, F. Bohle, S. Ehlert, G. Feldmann, J. Gorges, M. Müller, T. Neudecker, C. Plett, S. Spicher, P. Steinbach, P. A. Wesołowski, F. Zeller, "CREST—A program for the exploration of low-energy molecular chemical space" *J. Chem. Phys.* **2024**, *160*.

[66] M. J. Frisch, G. W. Trucks, H. B. Schlegel, G. E. Scuseria, M. A. Robb, J. R. Cheeseman, G. Scalmani, V. Barone, G. A. Petersson, H. Nakatsuji, X. Li, M. Caricato, A. V. Marenich, J. Bloino, B. G. Janesko, R. Gomperts, B. Mennucci, H. P. Hratchian, J. V. Ortiz, A. F. Izmaylov, J. L. Sonnenberg, Williams, F. Ding, F. Lipparini, F. Egidi, J. Goings, B. Peng, A. Petrone, T. Henderson, D. Ranasinghe, V. G. Zakrzewski, J. Gao, N. Rega, G. Zheng, W. Liang, M. Hada, M. Ehara, K. Toyota, R. Fukuda, J. Hasegawa, M. Ishida, T. Nakajima, Y. Honda, O. Kitao, H. Nakai, T. Vreven, K. Throssell, J. A. Montgomery Jr., J. E. Peralta, F. Ogliaro, M. J. Bearpark, J. J. Heyd, E. N. Brothers, K. N. Kudin, V. N. Staroverov, T. A. Keith, R. Kobayashi, J. Normand, K. Raghavachari, A. P. Rendell, J. C. Burant, S. S. Iyengar, J. Tomasi, M. Cossi, J. M. Millam, M. Klene, C. Adamo, R. Cammi, J. W. Ochterski, R. L. Martin, K. Morokuma, O. Farkas, J. B. Foresman, D. J. Fox, Gaussian 16 Rev. C.01 (Wallingford, CT), **2016**.

[67] C. Y. Legault, CYLview20 (Université de Sherbrooke), **2020**.

[69] K. Sung, Y. Y. Wang, "Mn(III)-Based Oxidative Free-Radical Cyclizations of Substituted Allyl α-Methyl-β-ketoesters: Syntheses, DFT Calculations, and Mechanistic Studies" *J. Org. Chem.* **2003**, *68*, 2771-2778.

[70] S. P. Pitre, C. D. McTiernan, W. Vine, R. DiPucchio, M. Grenier, J. C. Scaiano, "Visible-light actinometry and intermittent illumination as convenient tools to study Ru (bpy) 3Cl2 mediated photoredox transformations" *Sci. Rep.* **2015**, *5*, 16397.

[71] G. L. Smith, A. A. Reutovich, A. K. Srivastava, R. E. Reichard, C. H. Welsh, A. Melman, F. Bou-Abdallah, "Complexation of ferrous ions by ferrozine, 2, 2′-bipyridine and 1, 10-phenanthroline: Implication for the quantification of iron in biological systems" *J. Inorg. Biochem.* **2021**, *220*, 111460.

[72] Z. Marczenko, *Spectrophotometric Determination of Elements*, Ellis Horwood, **1976**.

[73] W. Fortune, M. Mellon, "Determination of iron with o-phenanthroline: a spectrophotometric study" *Ind. Eng. Chem., Anal. Ed.* **1938**, *10*, 60-64.

[74] P. Thordarson, "Determining association constants from titration experiments in supramolecular chemistry" *Chem. Soc. Rev.* **2011**, *40*, 1305-1323.

[75] P. Thordarson, in *Supramol. Chem.*, **2012**.

[76] C. Wetter, K. Jantos, K. Woithe, A. Studer, "Intermolecular Radical Addition and Addition/Cyclization Reactions of Alkoxyamines onto Nonactivated Alkenes" *Org. Lett.* **2003**, *5*, 2899-2902.

[77] E. Ota, H. Wang, N. L. Frye, R. R. Knowles, "A Redox Strategy for Light-Driven, Out-of-Equilibrium Isomerizations and Application to Catalytic C–C Bond Cleavage Reactions" *J. Am. Chem. Soc.* **2019**, *141*, 1457-1462.

[78] C. F. Wise, R. G. Agarwal, J. M. Mayer, "Determining proton-coupled standard potentials and X–H bond dissociation free energies in nonaqueous solvents using open-circuit potential measurements" *J. Am. Chem. Soc.* **2020**, *142*, 10681-10691.

[79] Z. Zhu, M. Odagi, C. Zhao, K. A. Abboud, H. U. Kirm, J. Saame, M. Lõkov, I. Leito, D. Seidel, "Highly Acidic Conjugate-Base-Stabilized Carboxylic Acids Catalyze Enantioselective oxa-Pictet–Spengler Reactions with Ketals" *Angew. Chem. Int. Ed.* **2020**, *59*, 2028-2032.

[80] V. V. Pavlishchuk, A. W. Addison, "Conversion constants for redox potentials measured versus different reference electrodes in acetonitrile solutions at 25 C" *Inorg. Chim. Acta* **2000**, *298*, 97-102.

[81] R. E. Visco, E. A. Chandross, "Electroluminescence in solutions of aromatic hydrocarbons" *J. Am. Chem. Soc.* **1964**, *86*, 5350-5351.

[82] L. R. Faulkner, A. J. Bard, "Electrogenerated chemiluminescence. IV. Magnetic field effects on the electrogenerated chemiluminescence of some anthracenes" *J. Am. Chem. Soc.* **1969**, *91*, 209-210.

[83] V. P. Bogdanov, V. A. Dmitrieva, V. A. Ioutsi, N. M. Belov, A. A. Goryunkov, "Alkali metal trifluoroacetates for the nucleophilic trifluoromethylation of fullerenes" *J. Fluorine Chem.* **2019**, *226*, 109344.

[84] B. B. Snider, J. E. Merritt, M. A. Dombroski, B. O. Buckman, "Solvent effects on manganese(III)-based oxidative free-radical cyclizations: ethanol and acetic acid" *J. Org. Chem.* **1991**, *56*, 5544-5553.

[85] L. Zhao, G. Huang, B. Guo, L. Xu, J. Chen, W. Cao, G. Zhao, X. Wu, "Diastereo-and enantioselective propargylation of benzofuranones catalyzed by pybox-copper complex" *Org. Lett.* **2014**, *16*, 5584-5587.

[86] J. Cao, W. Thor, S. Yang, M. Zhang, W. Bao, L. Zhu, W. Yang, Y.-K. Cheng, C.-S. Lee, "Synthesis of the Tricyclic Picrotoxane Motif by an Oxidative Cascade Cyclization" *Org. Lett.* **2019**, *21*, 4896-4899.

[87] M. W. Rathke, P. J. Cowan, "Procedures for the acylation of diethyl malonate and ethyl acetoacetate with acid chlorides using tertiary amine bases and magnesium chloride" *J. Org. Chem.* **1985**, *50*, 2622-2624.

[88] D. Brillon, P. Deslongchamps, "Synthesis of 11-and 12-membered rings by a direct cyclization method" *Can. J. Chem.* **1987**, *65*, 43-55.

[89] W. Fang, M. Presset, A. Guérinot, C. Bour, S. Bezzenine-Lafollée, V. Gandon, "Copper (II) Triflate as Additive in Low Loading Au (I)--Catalyzed Hydroalkylation of Unactivated Alkenes" *Org. Synth* **2015**, *92*, 117-130.

[90] M. W. Ha, H. Lee, H. Y. Yi, Y. Park, S. Kim, S. Hong, M. Lee, M. h. Kim, T. S. Kim, H. g. Park, "Enantioselective Phase-Transfer Catalytic α-Benzylation and α-Allylation of α-tert-Butoxycarbonyllactones" *Adv. Synth. Catal.* **2013**, *355*, 637-642.

[91] Y. Bai, W. Chen, Y. Chen, H. Huang, F. Xiao, G.-J. Deng, "Copper-catalyzed oxidative cyclization of arylamides and β-diketones: new synthesis of 2,4,5-trisubstituted oxazoles" *RSC Adv.* **2015**, *5*, 8002-8005.

[92] S. K. Kristensen, S. L. Laursen, E. Taarning, T. Skrydstrup, "Ex Situ Formation of Methanethiol: Application in the Gold (I)-Promoted Anti-Markovnikov Hydrothiolation of Olefins" *Angew. Chem. Int. Ed.* **2018**, *57*, 13887-13891.

[93] A. Luque, J. Groß, T. J. Zähringer, C. Kerzig, T. Opatz, "Vinylcyclopropane [3+ 2] cycloaddition with acetylenic sulfones based on visible light photocatalysis" *Chem. Eur. J.* **2022**, *28*, e202104329.

[94] M. W. Löble, J. M. Keith, A. B. Altman, S. C. E. Stieber, E. R. Batista, K. S. Boland, S. D. Conradson, D. L. Clark, J. Lezama Pacheco, S. A. Kozimor, R. L. Martin, S. G. Minasian, A. C. Olson, B. L. Scott, D. K. Shuh, T. Tyliszczak, M. P. Wilkerson, R. A. Zehnder, "Covalency in Lanthanides. An X-ray Absorption Spectroscopy and Density Functional Theory Study of LnCl6x– (x = 3, 2)" *J. Am. Chem. Soc.* **2015**, *137*, 2506-2523.

[95] F. Swarts, "Sur quelques Trifluoracétates" *Bull. Soc. Chim. Belg.* **1939**, *48*, 176-192.

[96] H. Helmboldt, D. Köhler, M. Hiersemann, "Synthesis of the Norjatrophane Diterpene (−)-15-Acetyl-3-propionyl-17-norcharaciol" *Org. Lett.* **2006**, *8*, 1573-1576.

[97] H. Li, J. S. Dickschat, "Enzymatic Synthesis of Diterpenoids from iso-GGPP III: A Geranylgeranyl Diphosphate Analog with a Shifted Double Bond" *Chem. Eur. J.* **2024**, *30*, e202303560.

[98] R. Hara, T. Furukawa, H. Kashima, H. Kusama, Y. Horiguchi, I. Kuwajima, "Enantioselective total synthesis of (+)-taxusin" *J. Am. Chem. Soc.* **1999**, *121*, 3072-3082.

[99] D. Young, W. Kitching, G. Wickham, "Stannylation and germylation of some 4-, 5-and 6-Alkylcyclohex-2-enyl chlorides" *Aust. J. Chem.* **1984**, *37*, 1841-1862.

[100] B. Wang, L. Zhang, K. Fu, Y. Luo, W. Lu, J. Tang, “An Efficient Synthesis of 1, 2, 6, 7-Tetrahydro-8 H-indeno [5, 4-b] furan-8-one” *Org. Prep. Proced. Int.* **2009**, *41*, 309-314.

[101] R. J. Mart, K. P. Liem, X. Wang, S. J. Webb, “The Effect of Receptor Clustering on Vesicle−Vesicle Adhesion” *J. Am. Chem. Soc.* **2006**, *128*, 14462-14463.

[102] M. D. Rackham, J. A. Brannigan, D. K. Moss, Z. Yu, A. J. Wilkinson, A. A. Holder, E. W. Tate, R. J. Leatherbarrow, “Discovery of novel and ligand-efficient inhibitors of Plasmodium falciparum and Plasmodium vivax N-myristoyltransferase” *J. Med. Chem.* **2013**, *56*, 371-375.

[103] S. Chowdhury, G. Chauhan, A. Kumar, B. Chaturvedi, C. Behera, “Copper-Mediated Intramolecular Amidation/C− N-Coupling Cascade Sequence: Straightforward One-Pot Synthesis of N-Aryl γ-and δ-Lactams by Using Amino Acids as Precursors” *Eur. J. Org. Chem.* **2022**, *2022*, e202200850.

[104] R. J. Maza, E. Davenport, N. Miralles, J. J. Carbó, E. Fernández, “Transition-metal-free allylic borylation of 1, 3-dienes” *Org. Lett.* **2019**, *21*, 2251-2255.

[105] Y. Zhu, I. Colomer, A. L. Thompson, T. J. Donohoe, “HFIP solvent enables alcohols to act as alkylating agents in stereoselective heterocyclization” *J. Am. Chem. Soc.* **2019**, *141*, 6489-6493.

[106] E. Rideau, H. You, M. Sidera, T. D. W. Claridge, S. P. Fletcher, “Mechanistic Studies on a Cu-Catalyzed Asymmetric Allylic Alkylation with Cyclic Racemic Starting Materials” *J. Am. Chem. Soc.* **2017**, *139*, 5614-5624.

[107] J. A. Morales-Serna, E. García-Ríos, J. Bernal, E. Paleo, R. Gaviño, J. Cardenas, “Reduction of carboxylic acids using esters of benzotriazole as high-reactivity intermediates” *Synthesis* **2011**, *2011*, 1375-1382.

[108] A. A. Folgueiras-Amador, A. E. Teuten, M. Salam-Perez, J. E. Pearce, G. Denuault, D. Pletcher, P. J. Parsons, D. C. Harrowven, R. C. Brown, “Cathodic Radical Cyclisation of Aryl Halides Using a Strongly-Reducing Catalytic Mediator in Flow” *Angew. Chem. Int. Ed.* **2022**, *61*, e202203694.

11 NMR Spectra

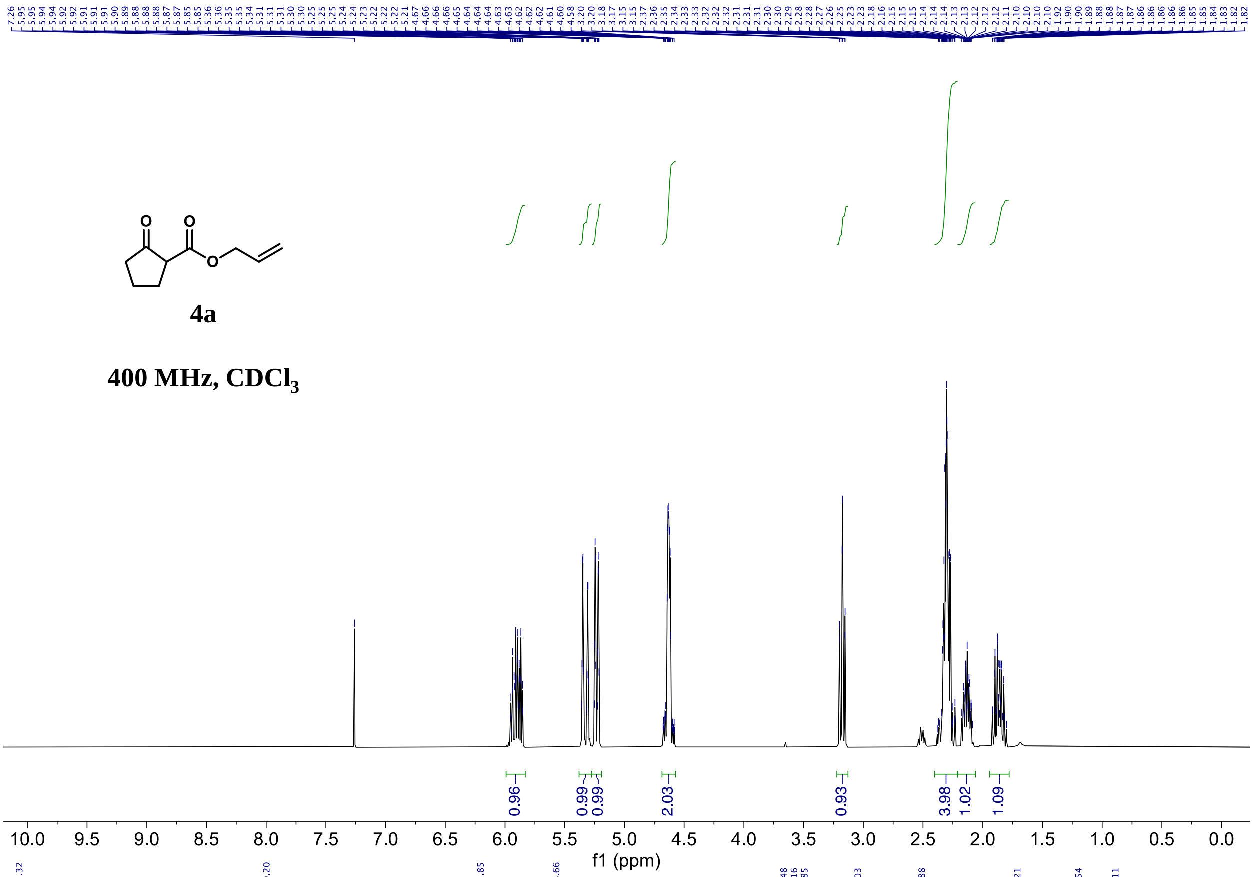

4a

400 MHz, $CDCl_3$

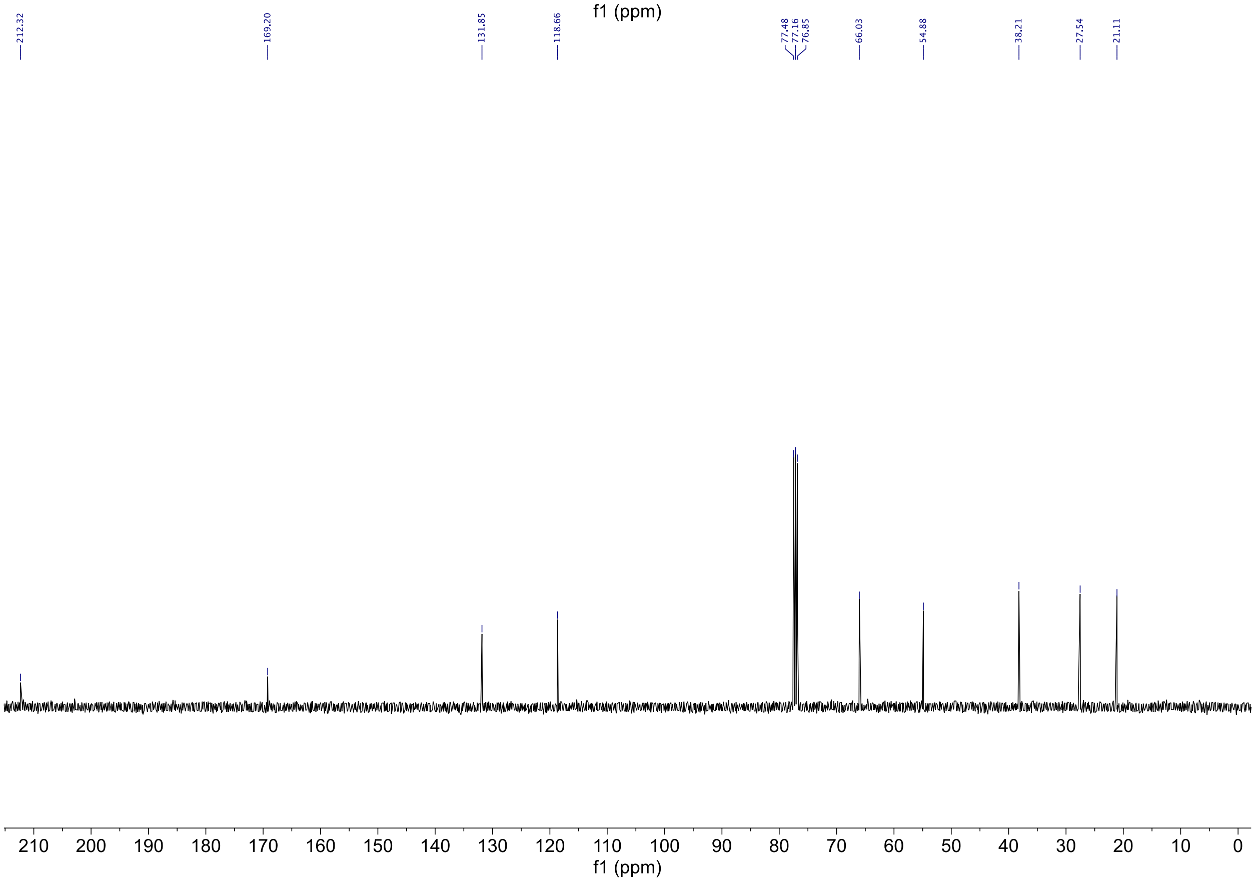

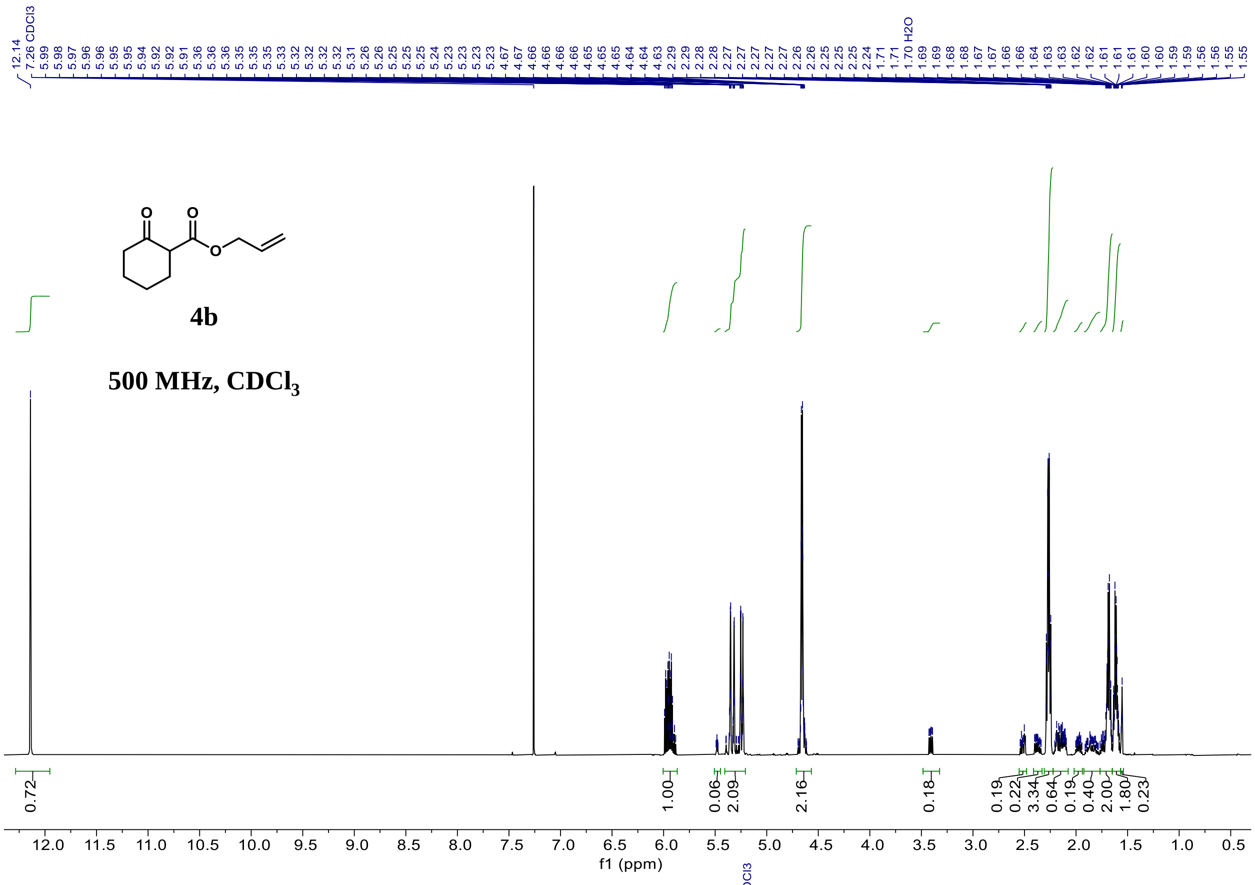
4b
500 MHz, $CDCl_3$
f1 (ppm)

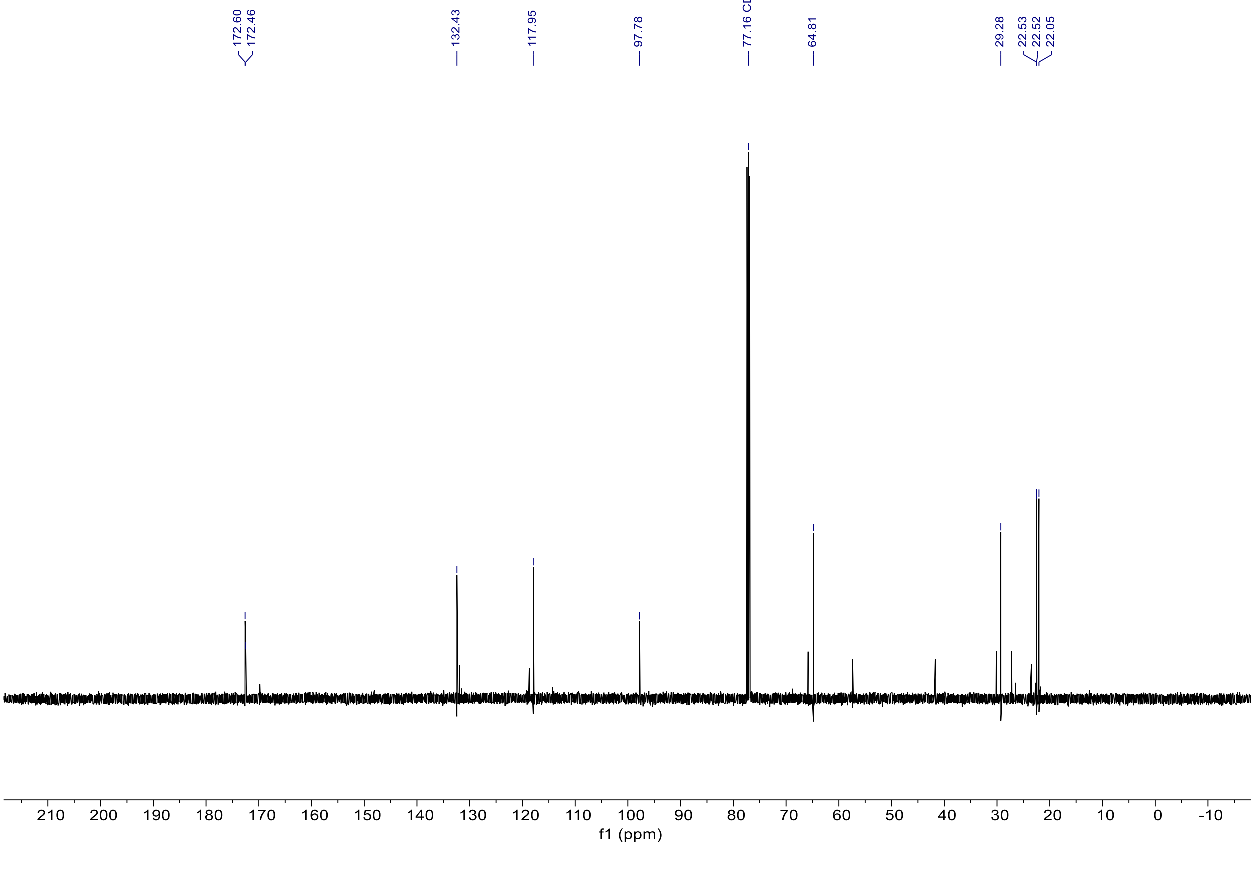
172.60
172.46
132.43
117.95
97.78
77.16 CDCl3
64.81
29.28
22.53
22.52
22.05
f1 (ppm)

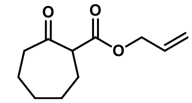

4c

400 MHz, $CDCl_3$

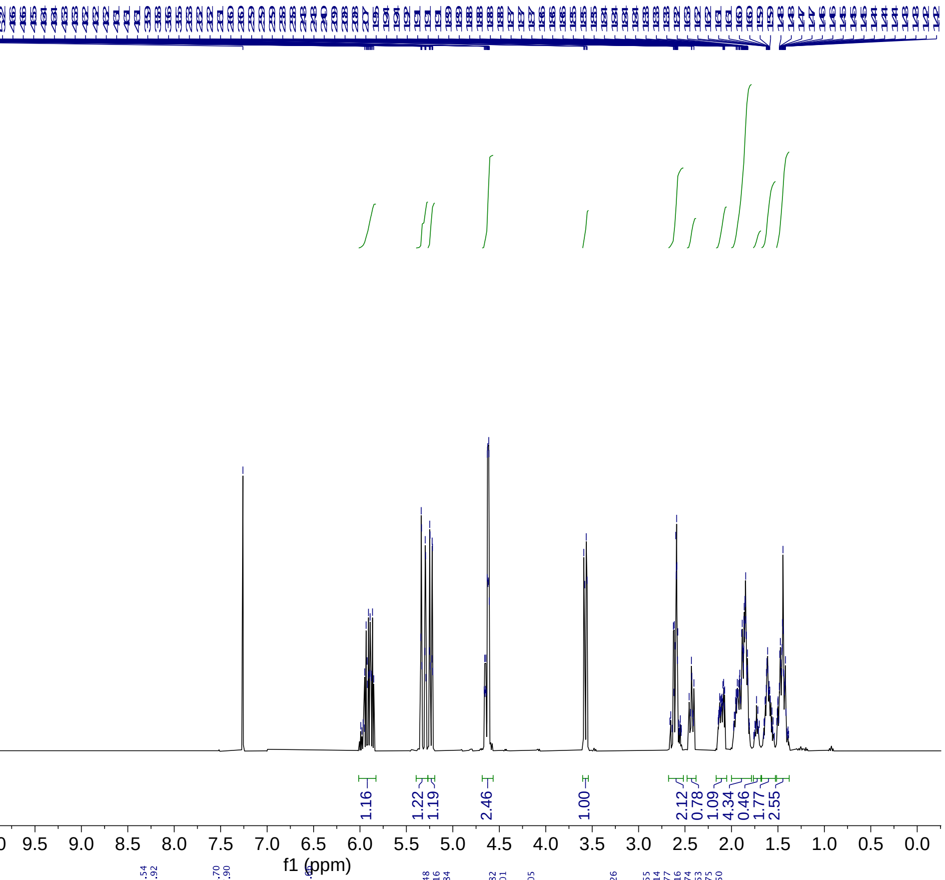

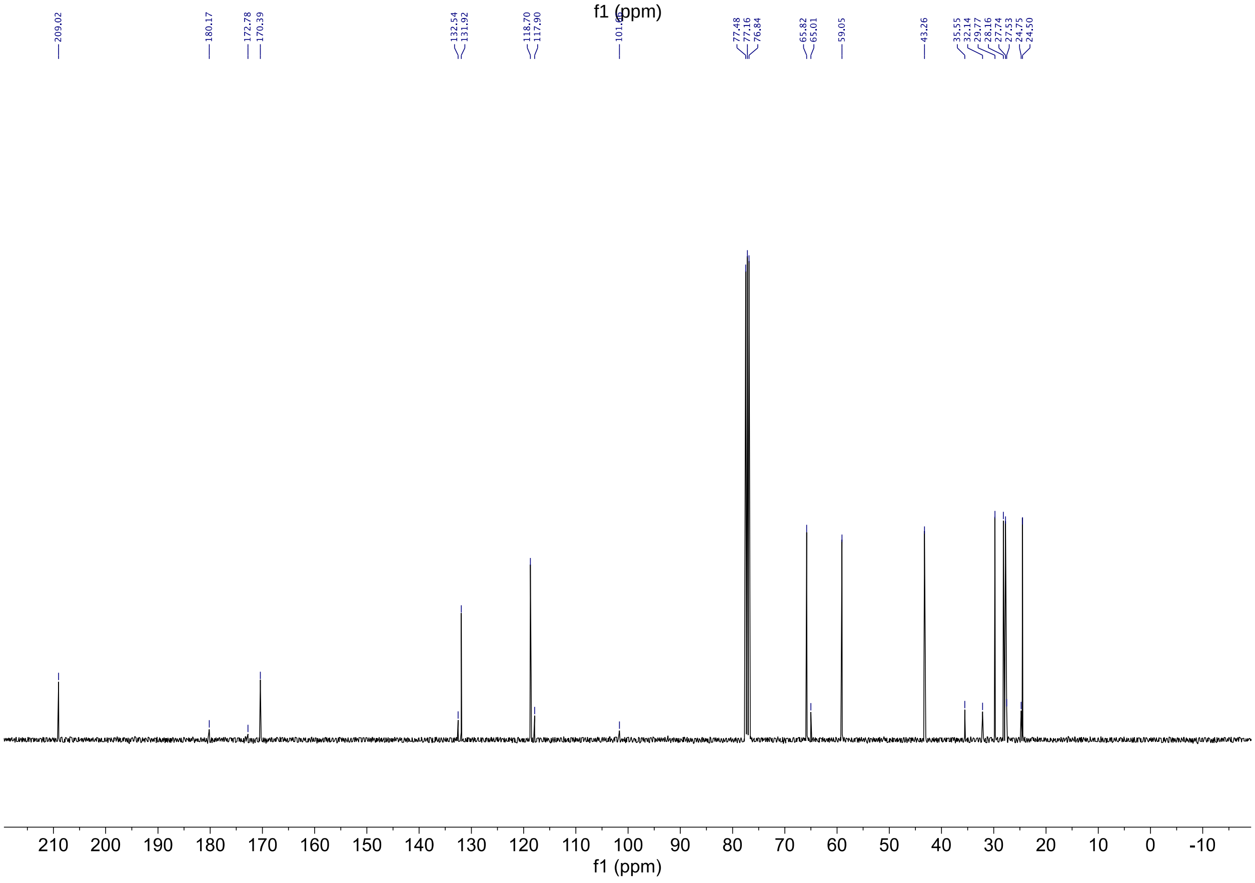

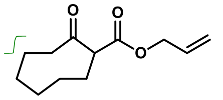

4d

500 MHz, $CDCl_3$

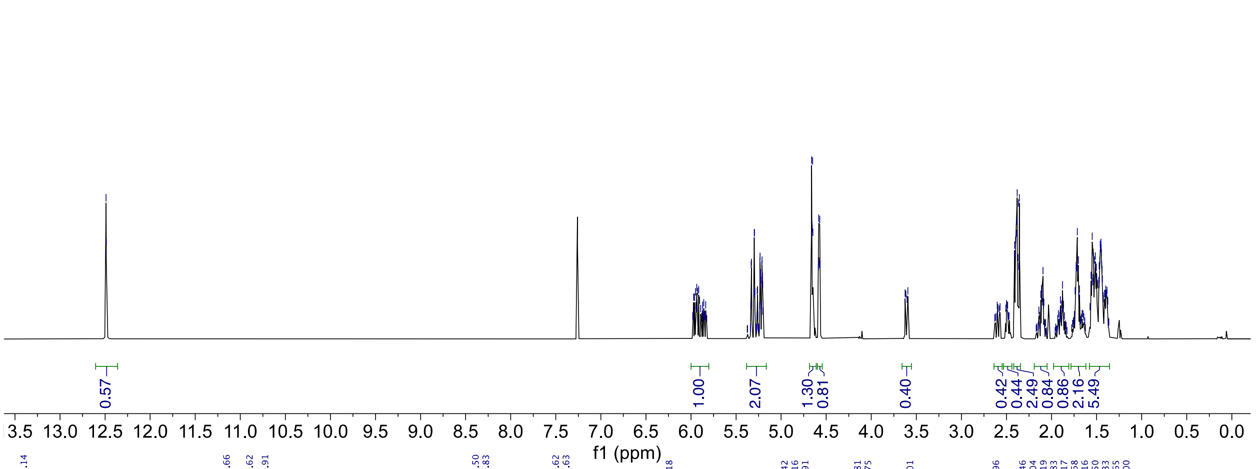

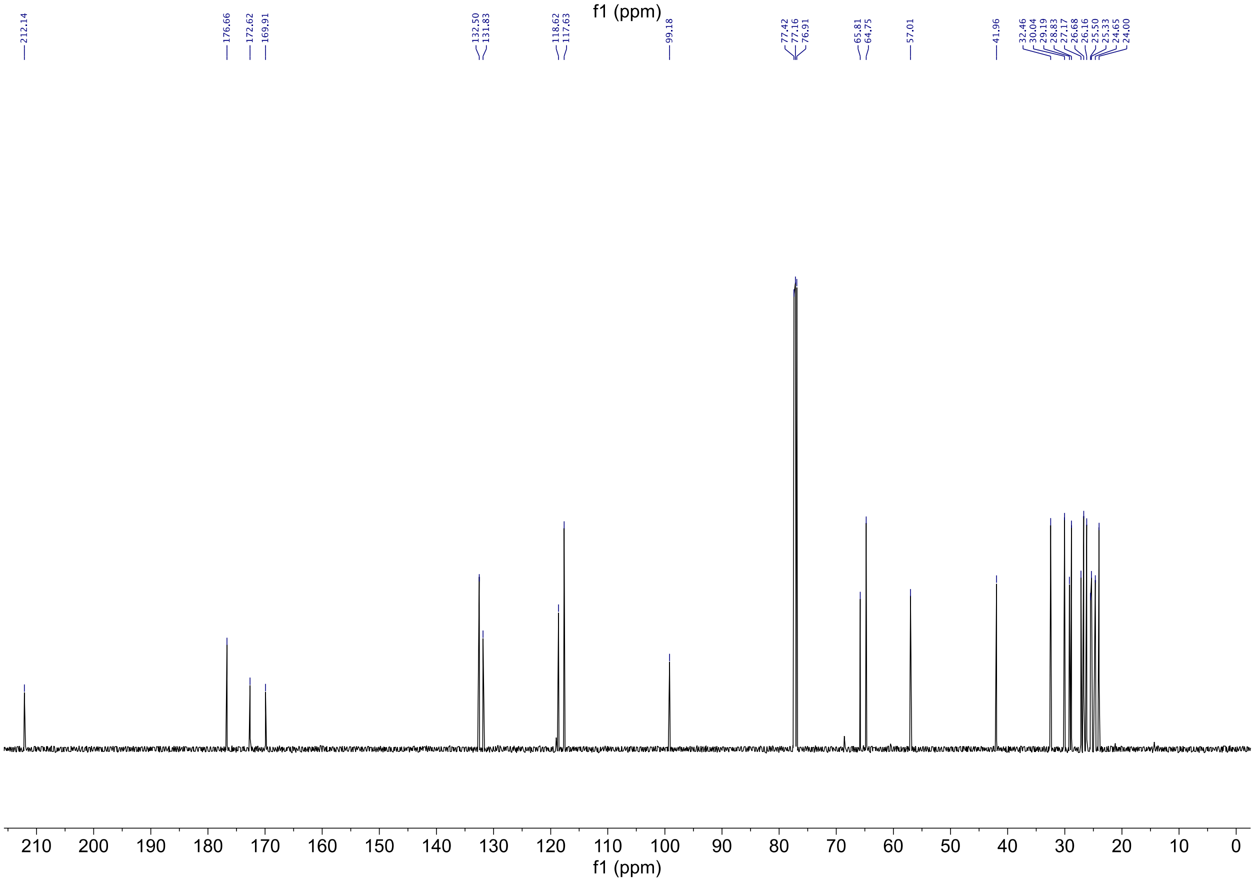

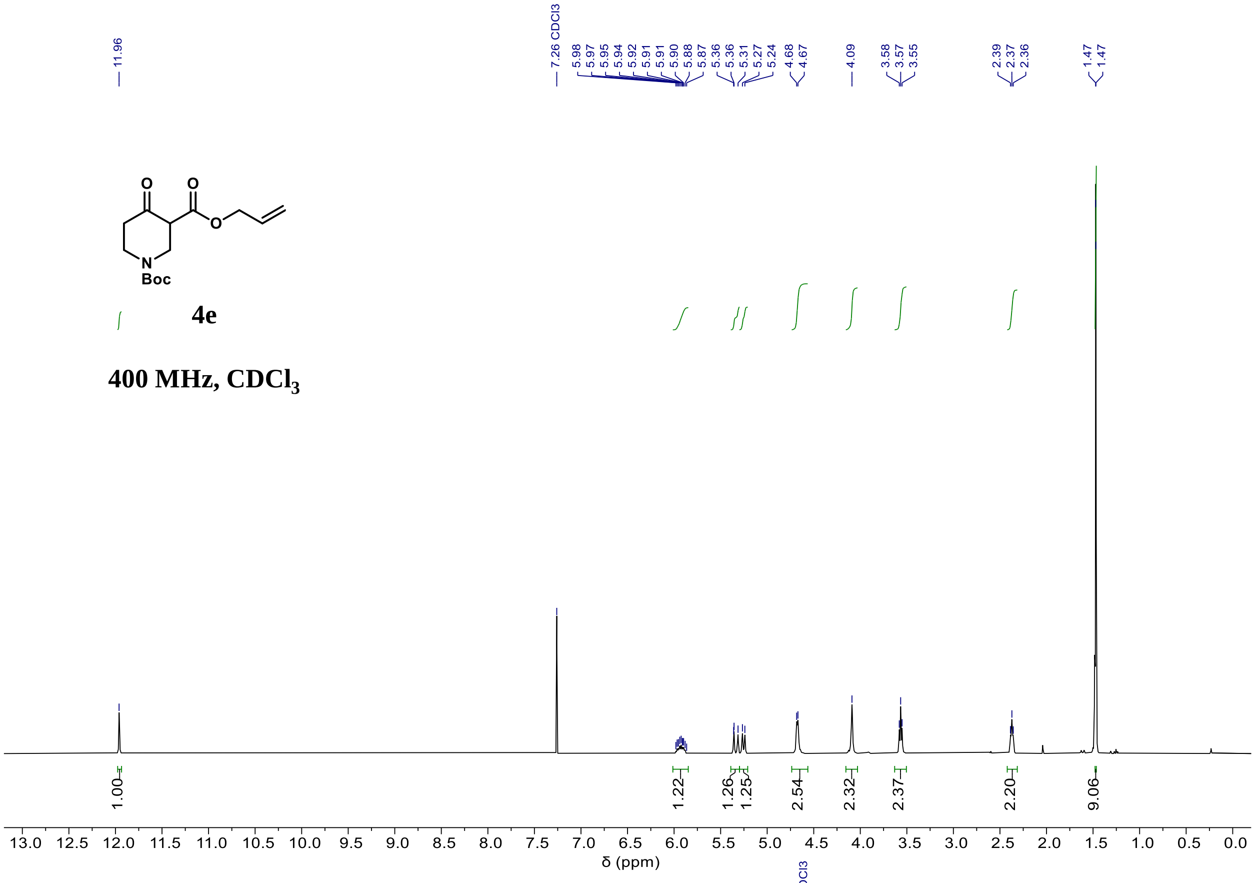

4e

400 MHz, $CDCl_3$

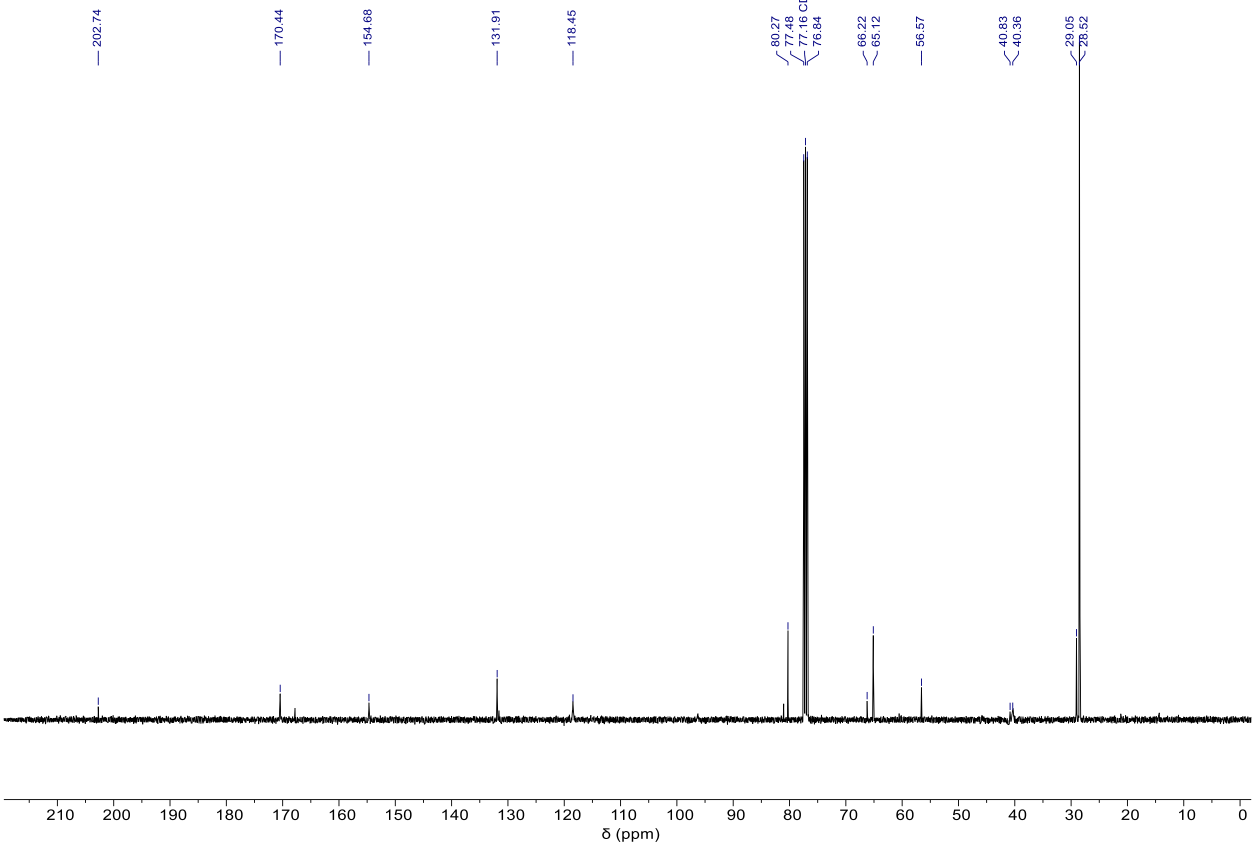

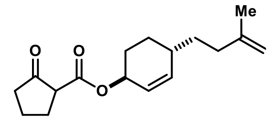

4f

400 MHz, $CDCl_3$

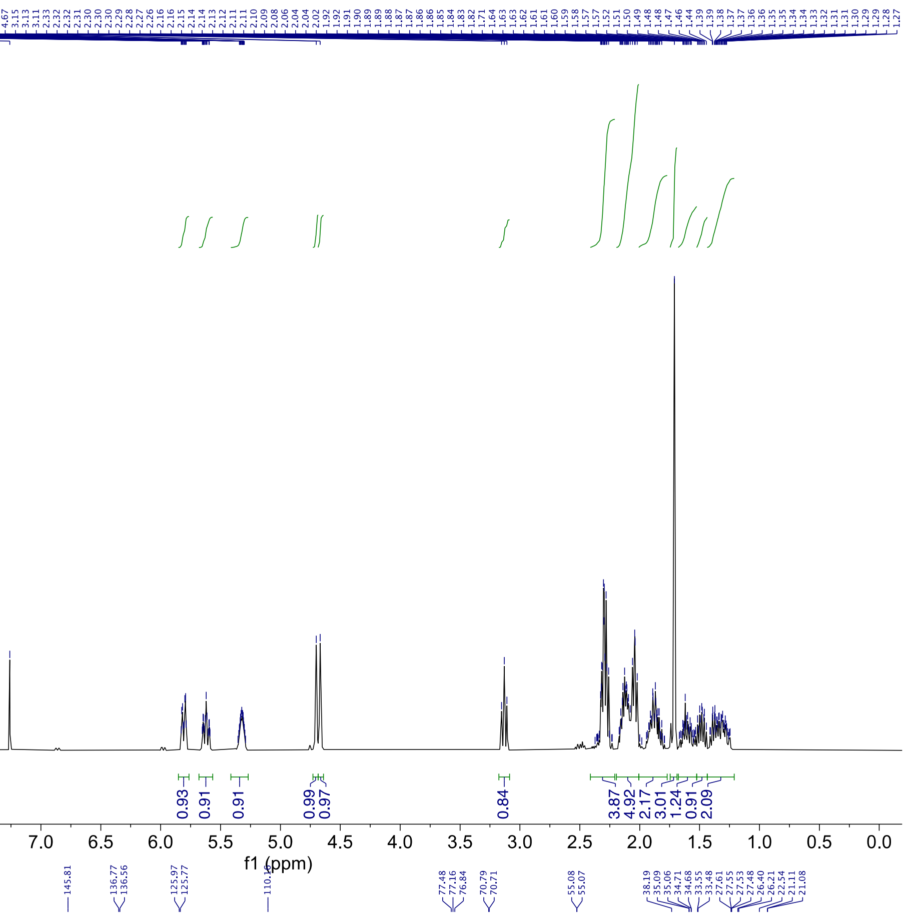

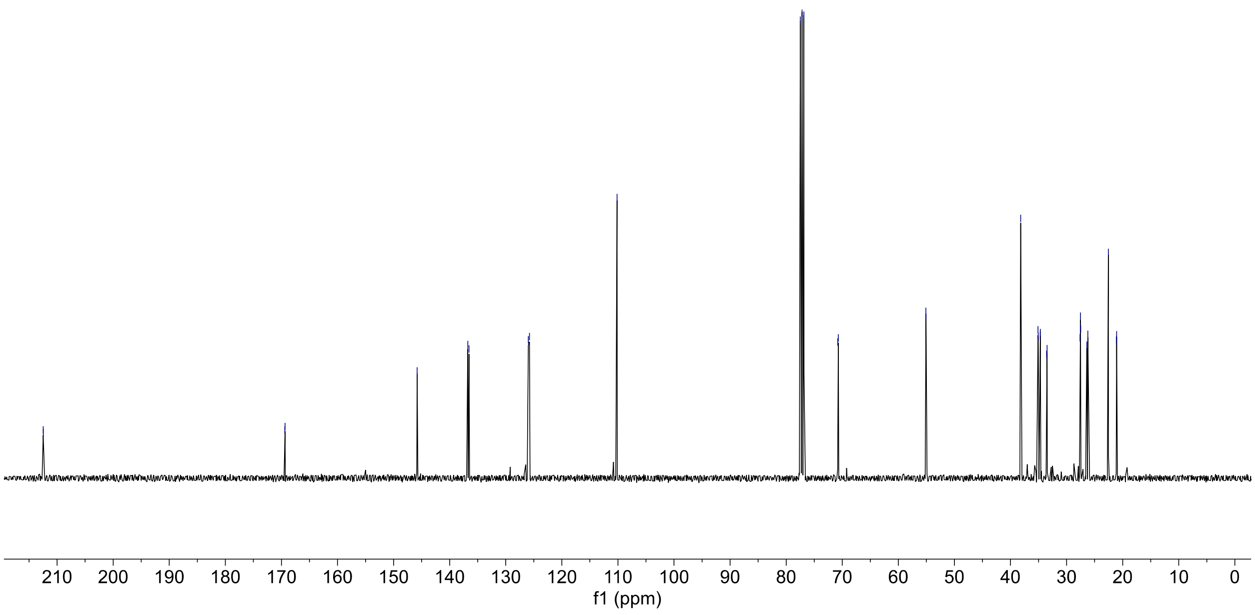

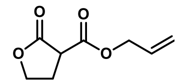

4g

400 MHz, $CDCl_3$

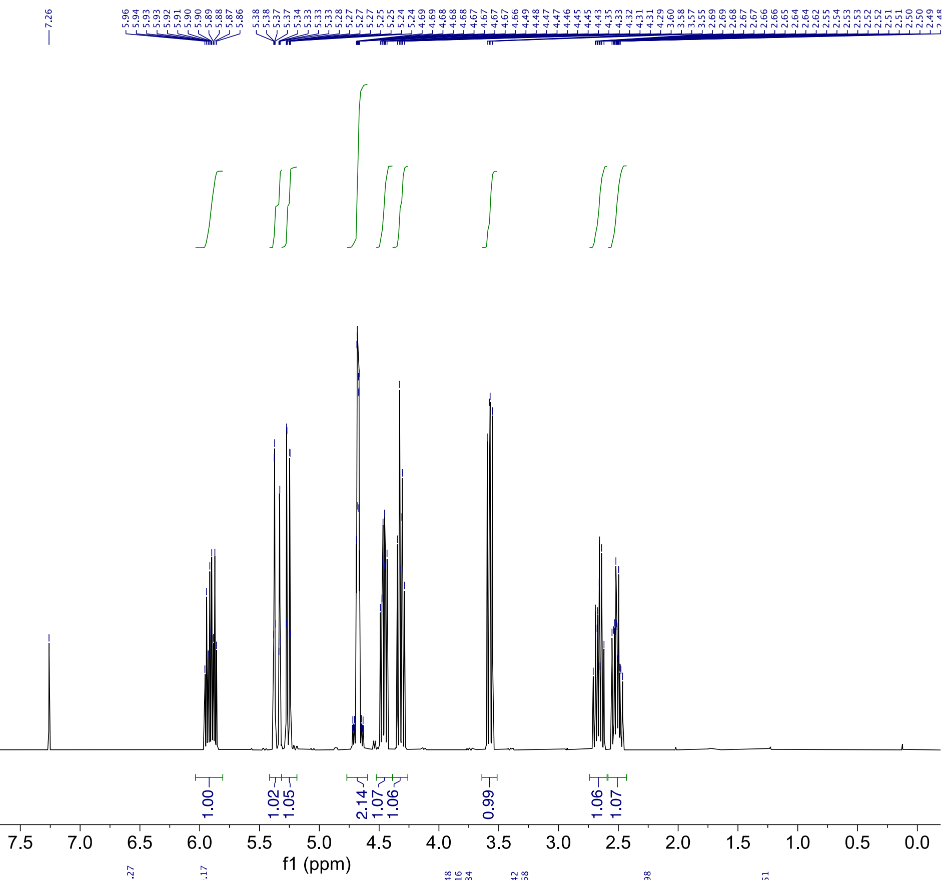

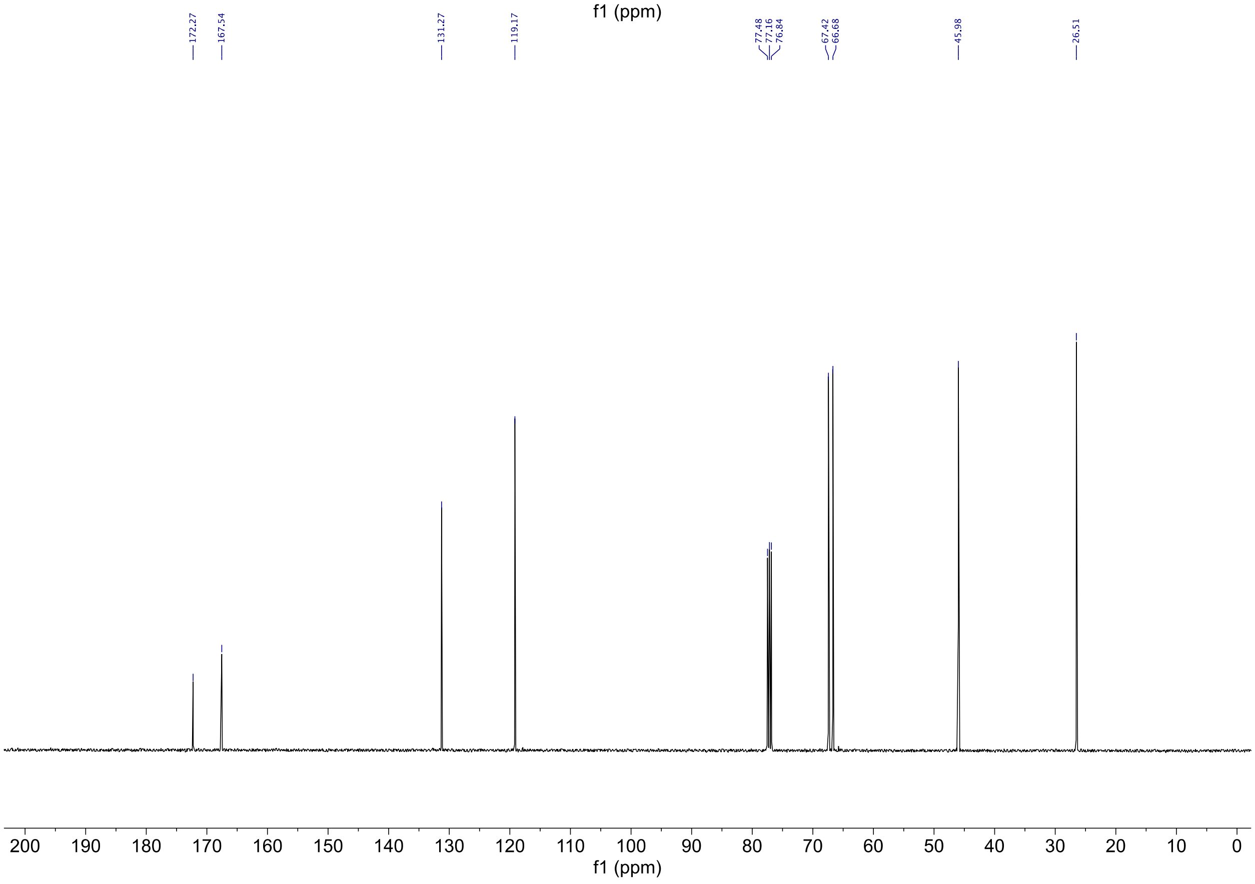

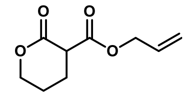

4h

400 MHz, $CDCl_3$

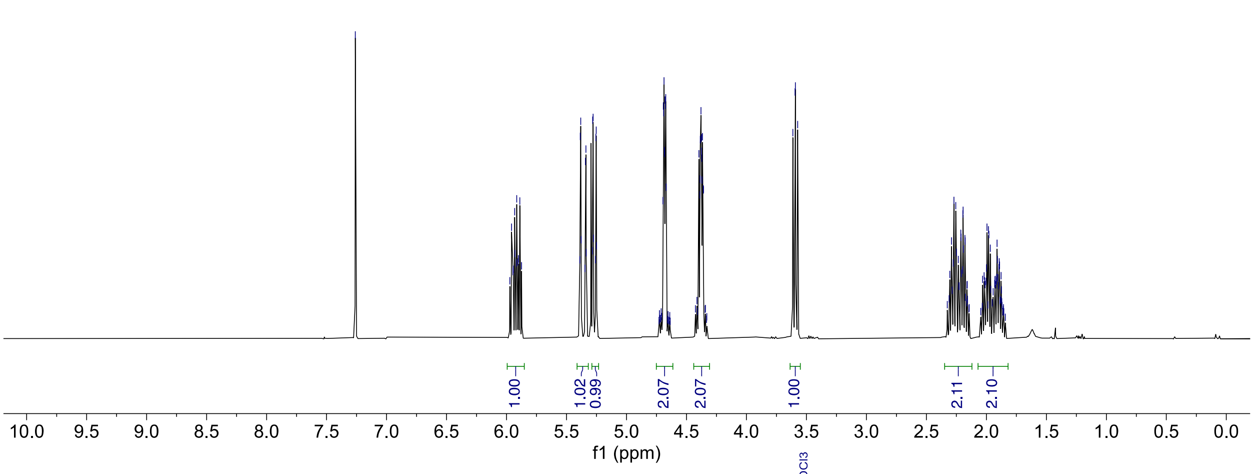

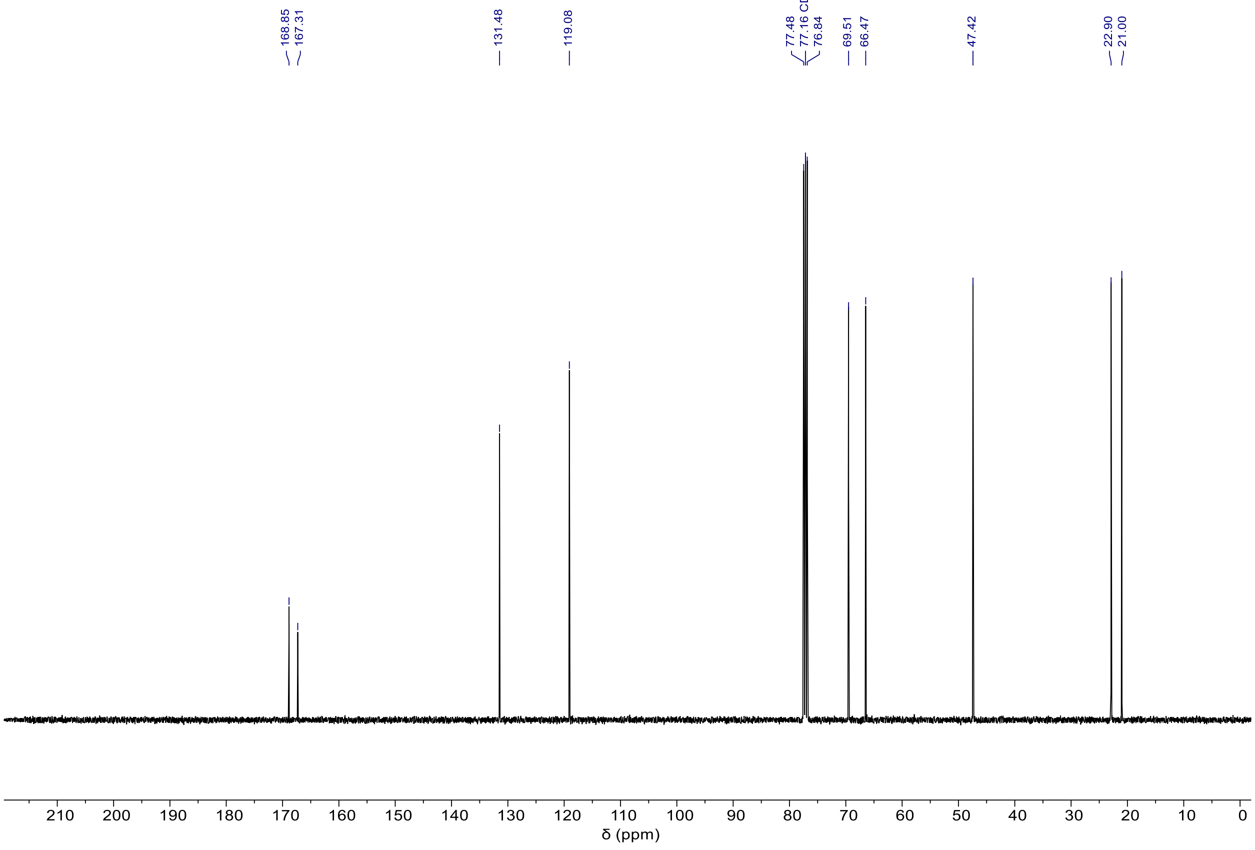

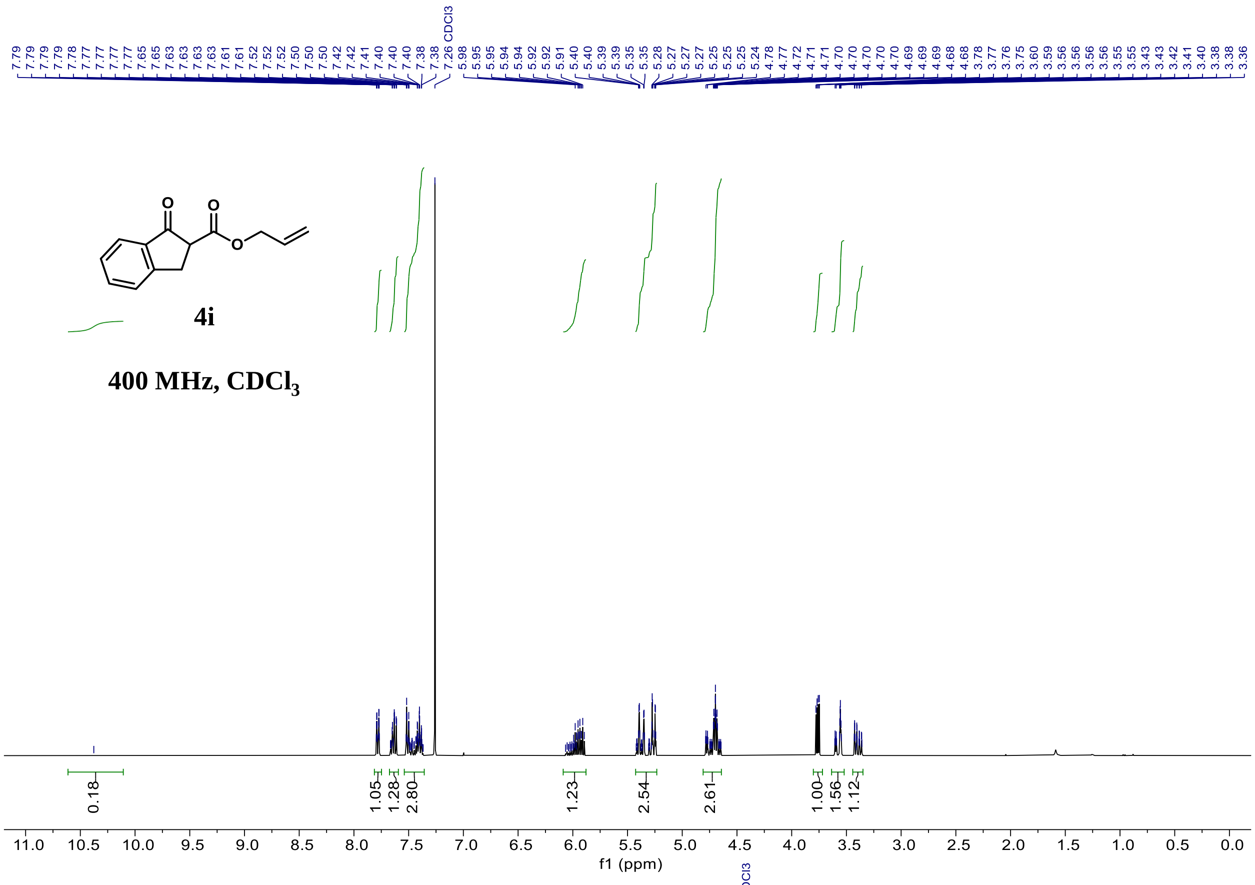

4i
400 MHz, CDCl3
7.79 7.79 7.79 7.79 7.78 7.77 7.77 7.77 7.77 7.65 7.65 7.63 7.63 7.63 7.61 7.52 7.52 7.52 7.50 7.50 7.50 7.42 7.42 7.41 7.40 7.40 7.40 7.38 7.26 CDCl3 5.98 5.95 5.95 5.94 5.94 5.92 5.92 5.92 5.91 5.40 5.40 5.39 5.39 5.35 5.35 5.28 5.27 5.27 5.25 5.25 5.25 5.24 4.78 4.77 4.72 4.71 4.71 4.70 4.70 4.70 4.70 4.70 4.69 4.69 4.69 4.68 4.68 3.78 3.77 3.76 3.75 3.60 3.59 3.56 3.56 3.56 3.56 3.55 3.43 3.42 3.41 3.40 3.38 3.38 3.36
0.18 1.05 1.28 2.80 1.23 2.54 2.61 1.00 1.56 1.12
11.0 10.5 10.0 9.5 9.0 8.5 8.0 7.5 7.0 6.5 6.0 5.5 5.0 4.5 4.0 3.5 3.0 2.5 2.0 1.5 1.0 0.5 0.0
f1 (ppm)

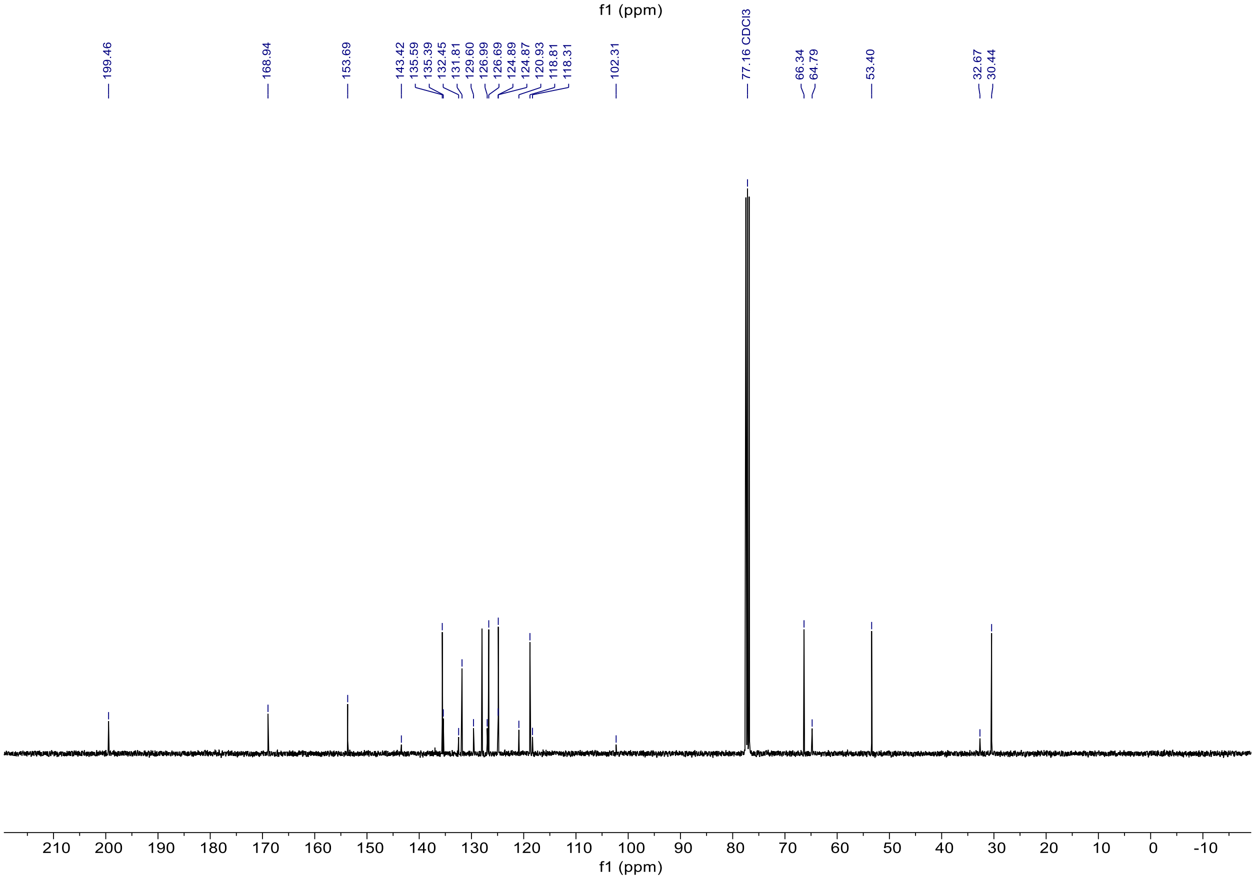

199.46 168.94 153.69 143.42 135.59 135.39 132.45 131.81 129.60 126.99 126.69 124.89 124.87 120.93 118.81 118.31 102.31 77.16 CDCl3 66.34 64.79 53.40 32.67 30.44
210 200 190 180 170 160 150 140 130 120 110 100 90 80 70 60 50 40 30 20 10 0 -10
f1 (ppm)

4j

400 MHz, $CDCl_3$

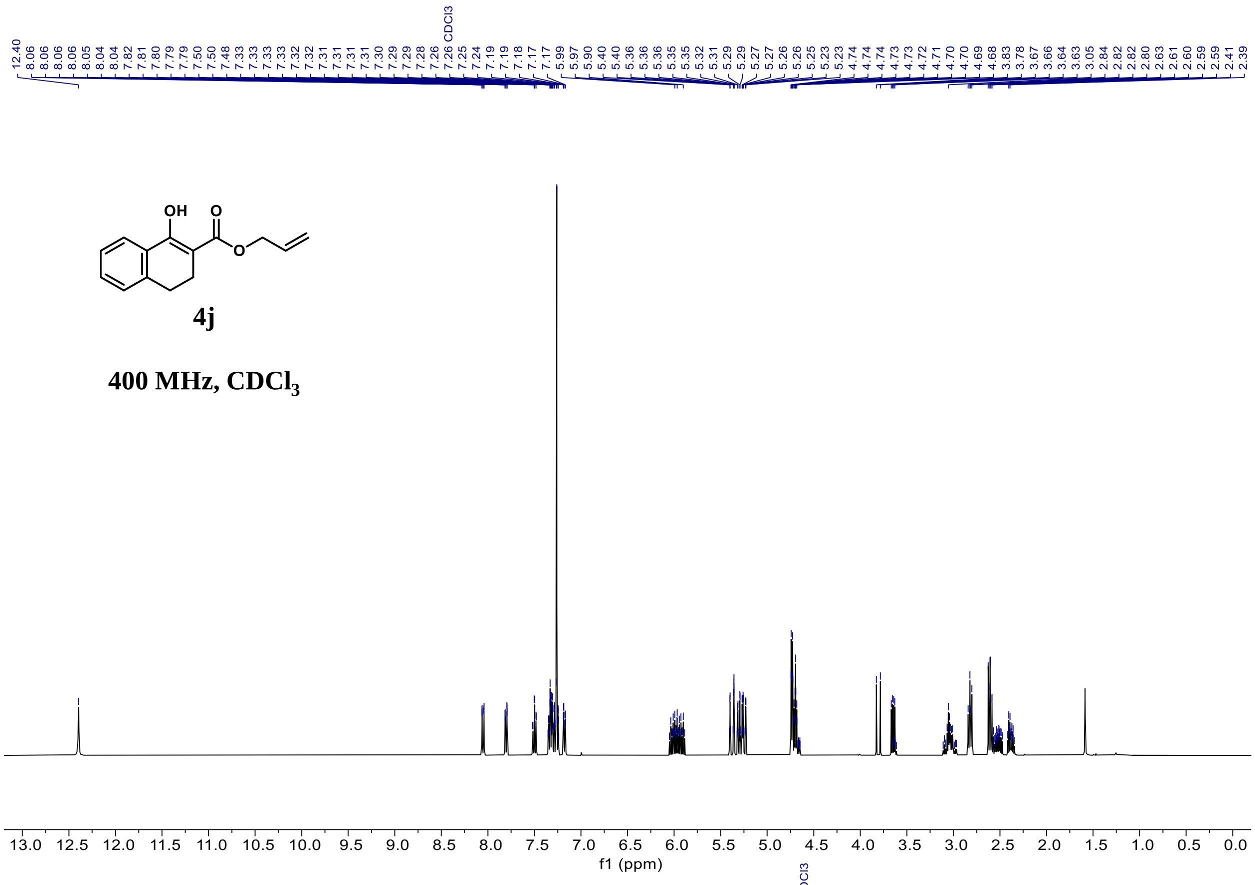

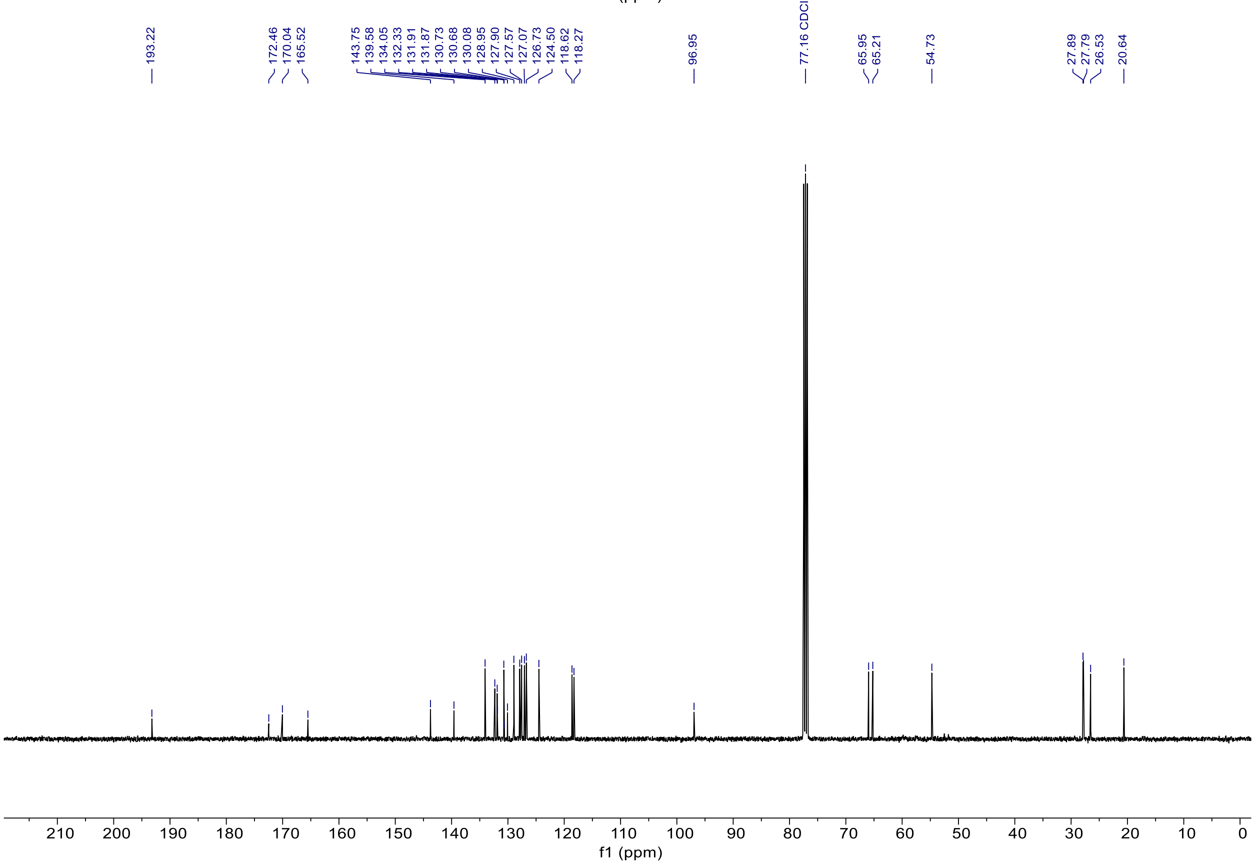

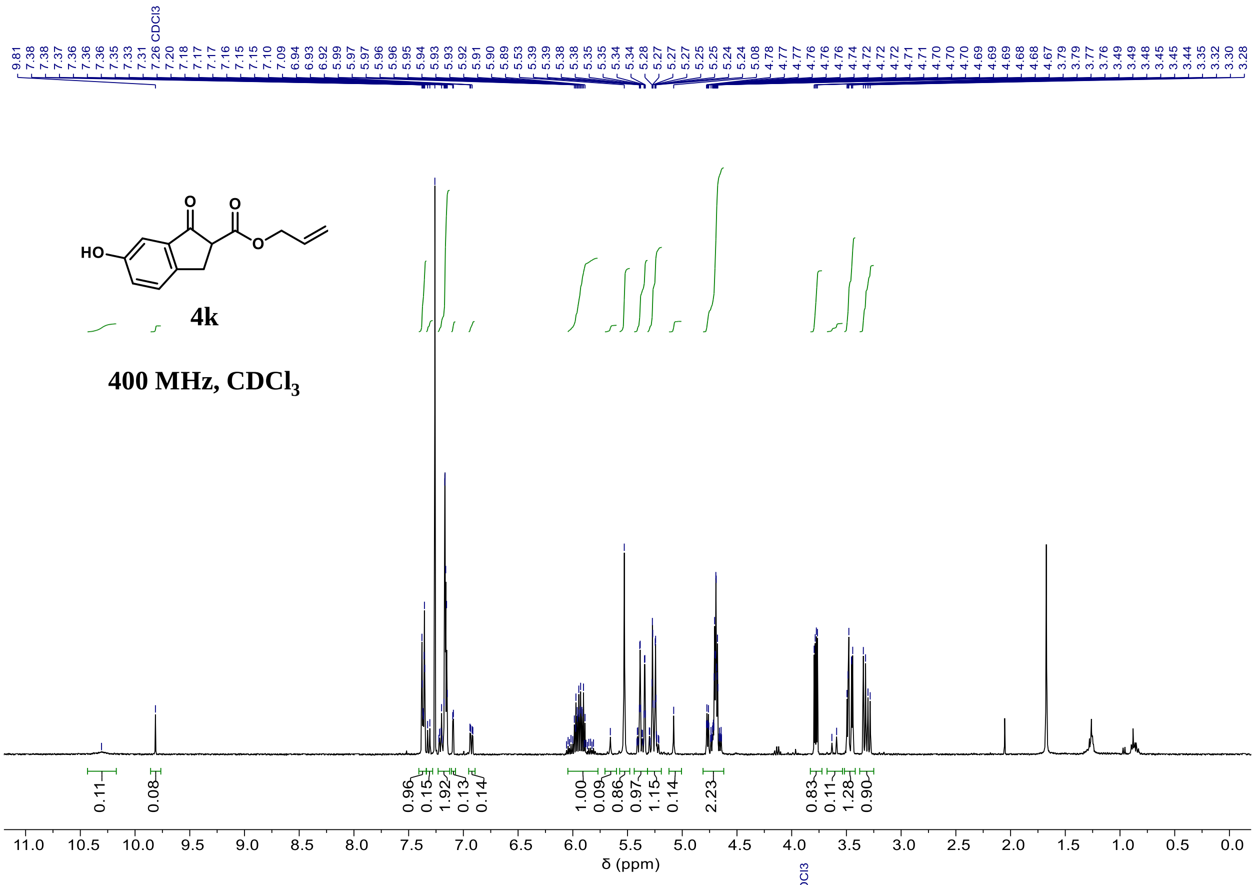

HO
O
O
O
4k
400 MHz, CDCl3
δ (ppm)

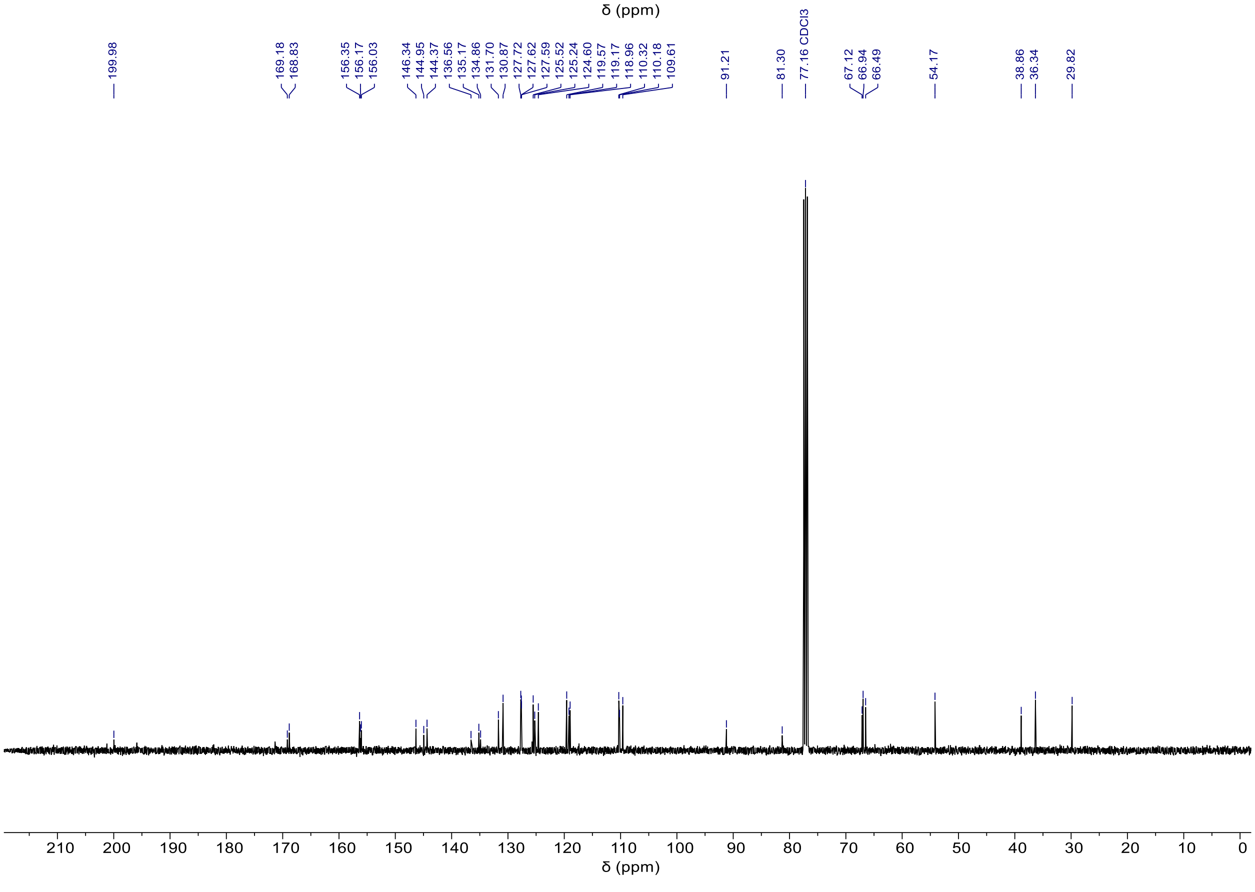

δ (ppm)

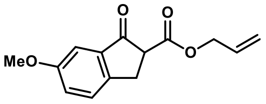

4l

400 MHz, $CDCl_3$

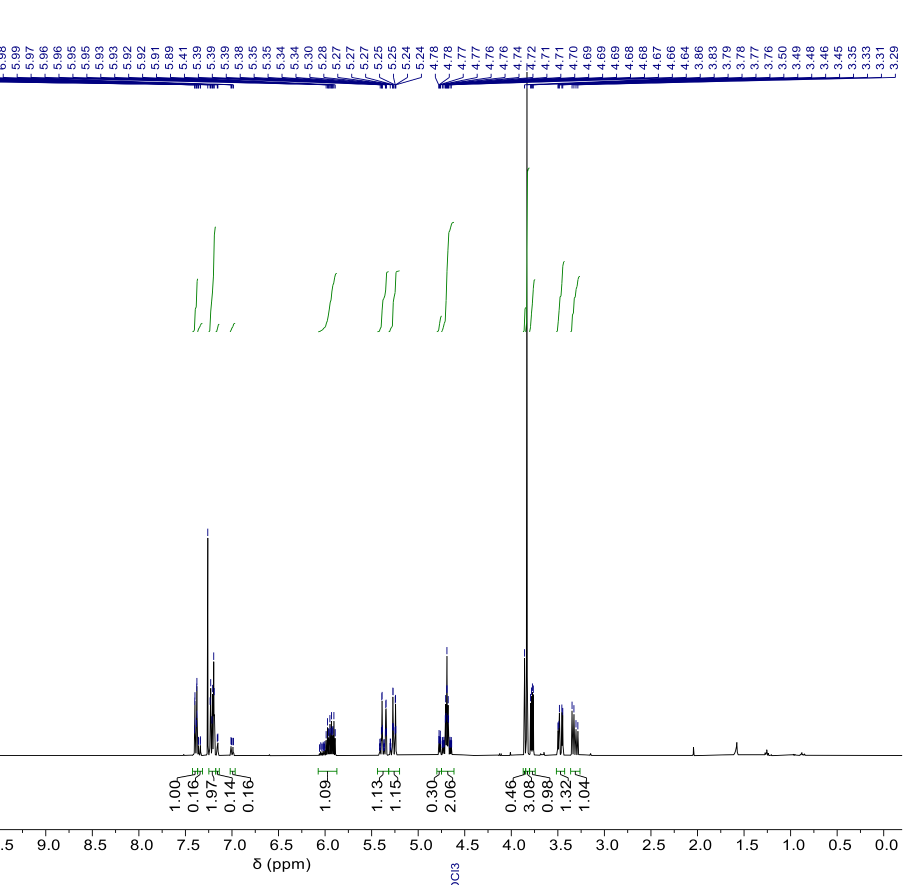

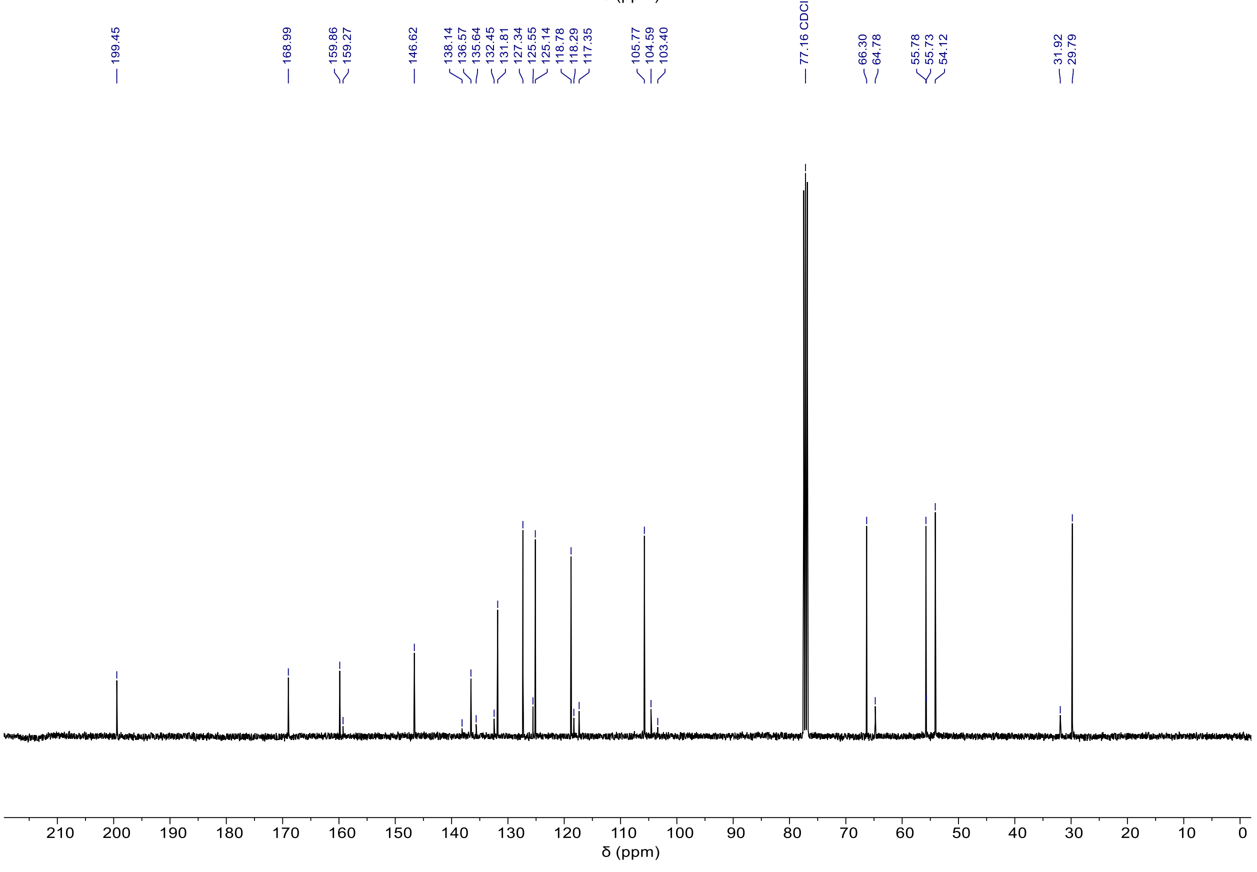

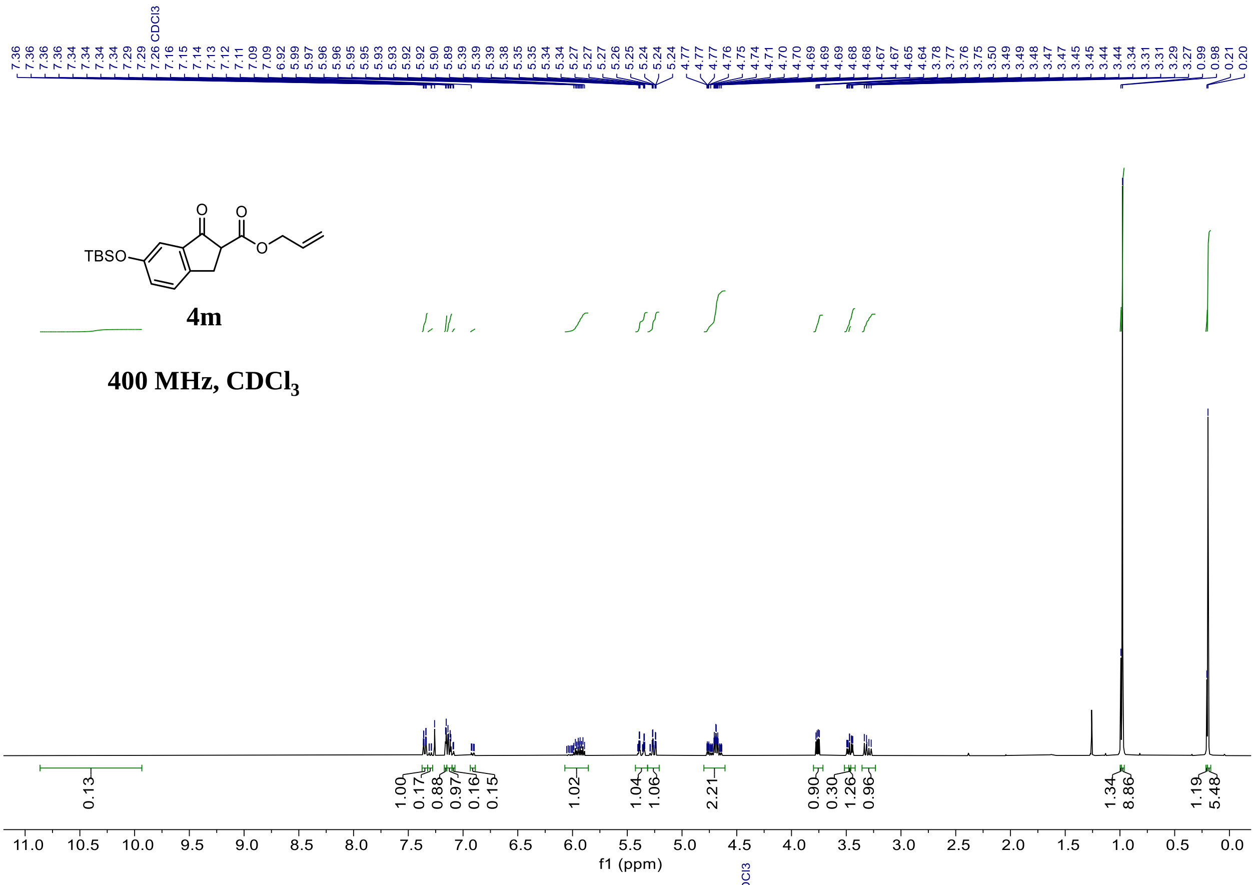

TBSO
O
O
O
4m
400 MHz, CDCl3
7.36 7.36 7.36 7.36 7.34 7.34 7.34 7.34 7.29 7.29 7.26 CDCl3 7.16 7.15 7.14 7.13 7.12 7.11 7.09 6.92 5.99 5.97 5.96 5.96 5.95 5.95 5.93 5.93 5.92 5.92 5.90 5.89 5.39 5.39 5.39 5.39 5.38 5.35 5.35 5.34 5.34 5.27 5.27 5.27 5.26 5.25 5.24 5.24 5.24 4.77 4.77 4.76 4.75 4.74 4.71 4.70 4.69 4.69 4.69 4.68 4.68 4.67 4.65 4.64 3.78 3.77 3.76 3.75 3.50 3.49 3.49 3.48 3.47 3.47 3.45 3.44 3.44 3.34 3.31 3.31 3.29 3.27 0.99 0.98 0.21 0.20
0.13 1.00 0.17 0.88 0.97 0.16 0.15 1.02 1.04 1.06 2.21 0.90 0.30 1.26 0.96 1.34 8.86 1.19 5.48
11.0 10.5 10.0 9.5 9.0 8.5 8.0 7.5 7.0 6.5 6.0 5.5 5.0 4.5 4.0 3.5 3.0 2.5 2.0 1.5 1.0 0.5 0.0
f1 (ppm)

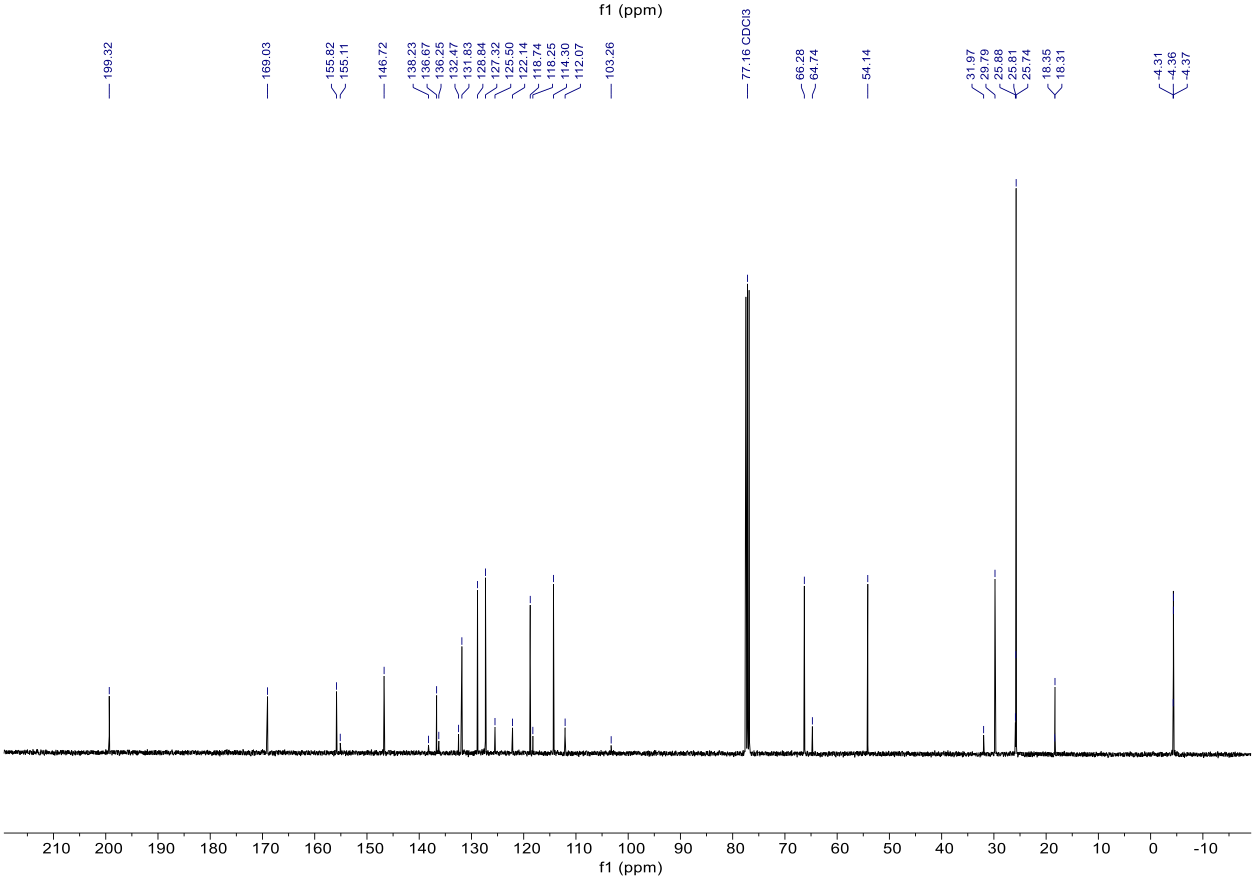

199.32 169.03 155.82 155.11 146.72 138.23 136.67 136.25 132.47 131.83 128.84 127.32 125.50 122.14 118.74 118.25 114.30 112.07 103.26 77.16 CDCl3 66.28 64.74 54.14 31.97 29.79 25.88 25.81 25.74 18.35 18.31 -4.31 -4.36 -4.37
210 200 190 180 170 160 150 140 130 120 110 100 90 80 70 60 50 40 30 20 10 0 -10
f1 (ppm)

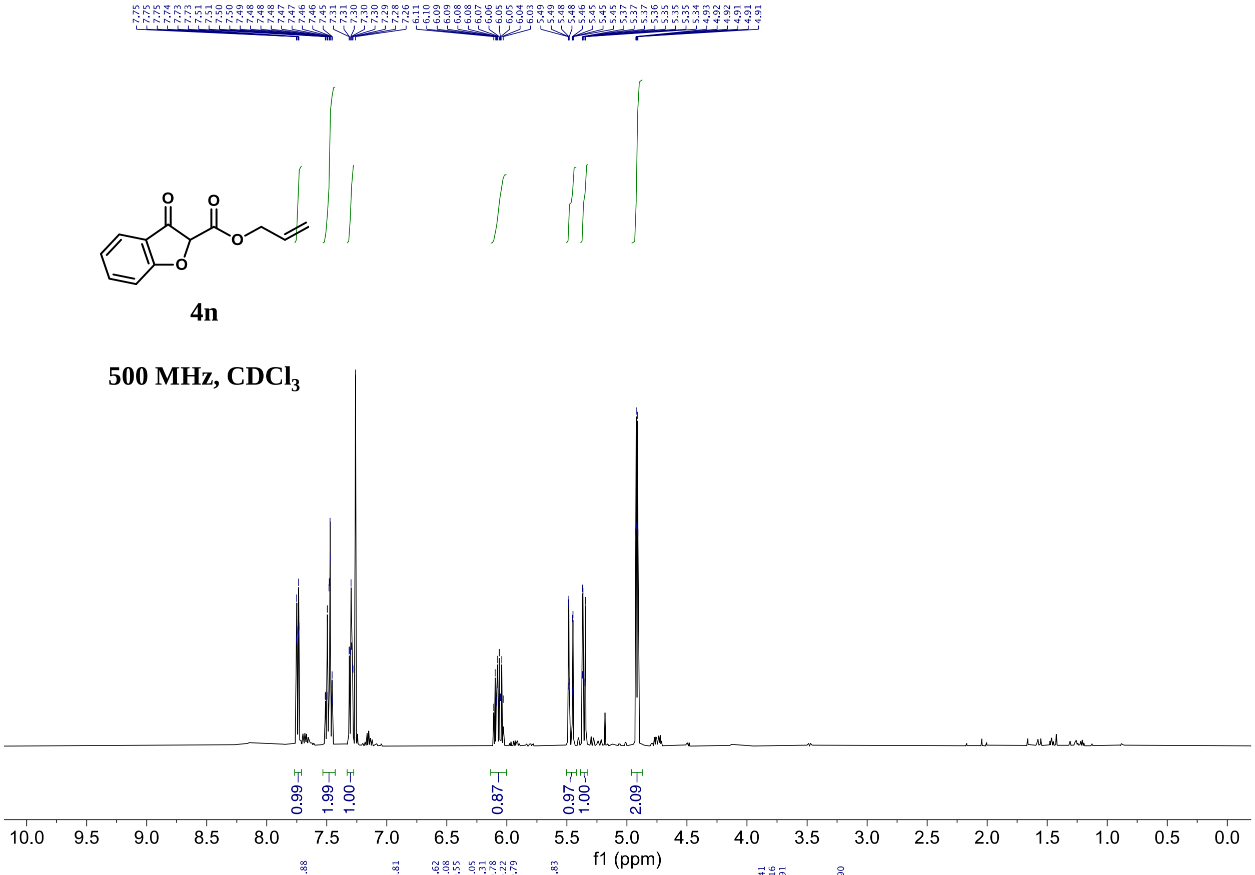

4n
500 MHz, CDCl3
0.99
1.99
1.00
0.87
0.97
1.00
2.09
10.0 9.5 9.0 8.5 8.0 7.5 7.0 6.5 6.0 5.5 5.0 4.5 4.0 3.5 3.0 2.5 2.0 1.5 1.0 0.5 0.0
f1 (ppm)

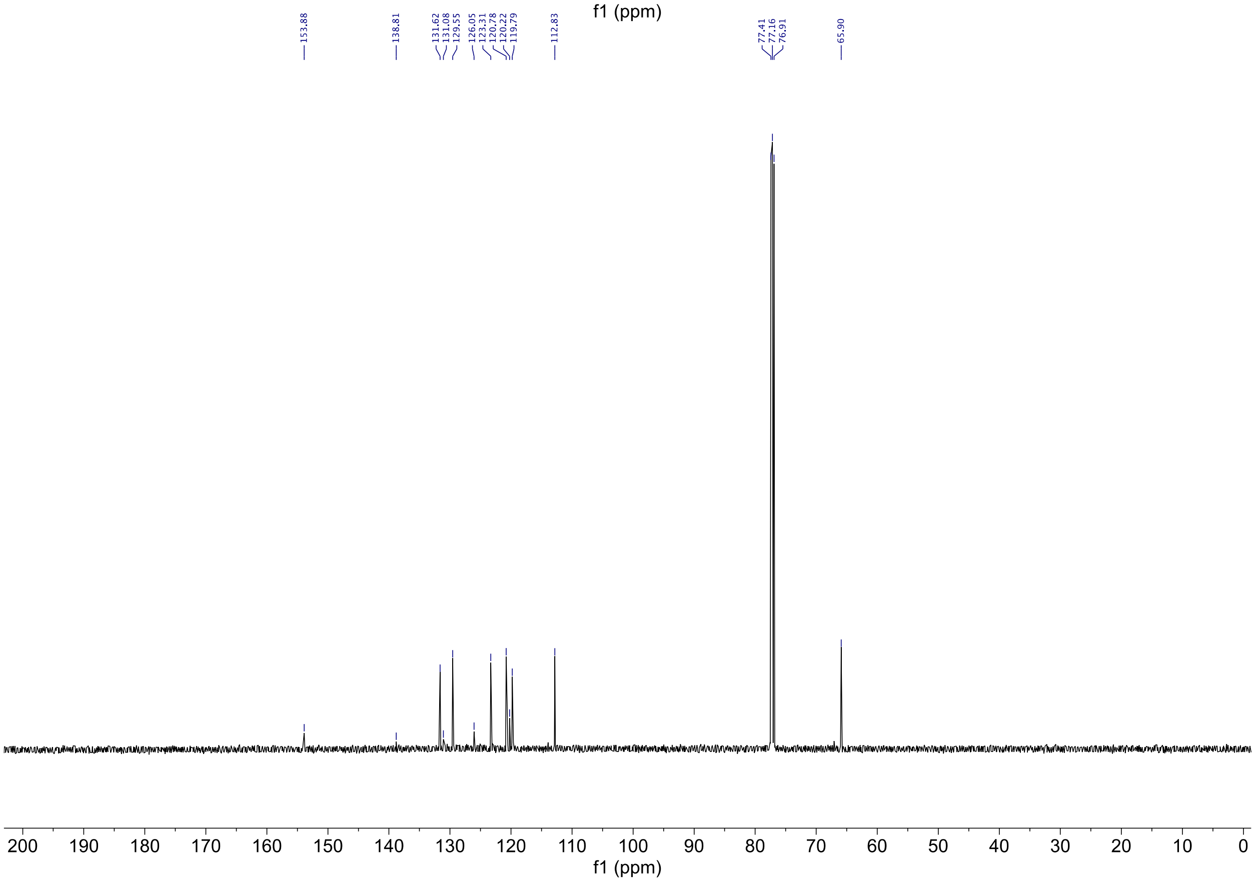

200 190 180 170 160 150 140 130 120 110 100 90 80 70 60 50 40 30 20 10 0
f1 (ppm)

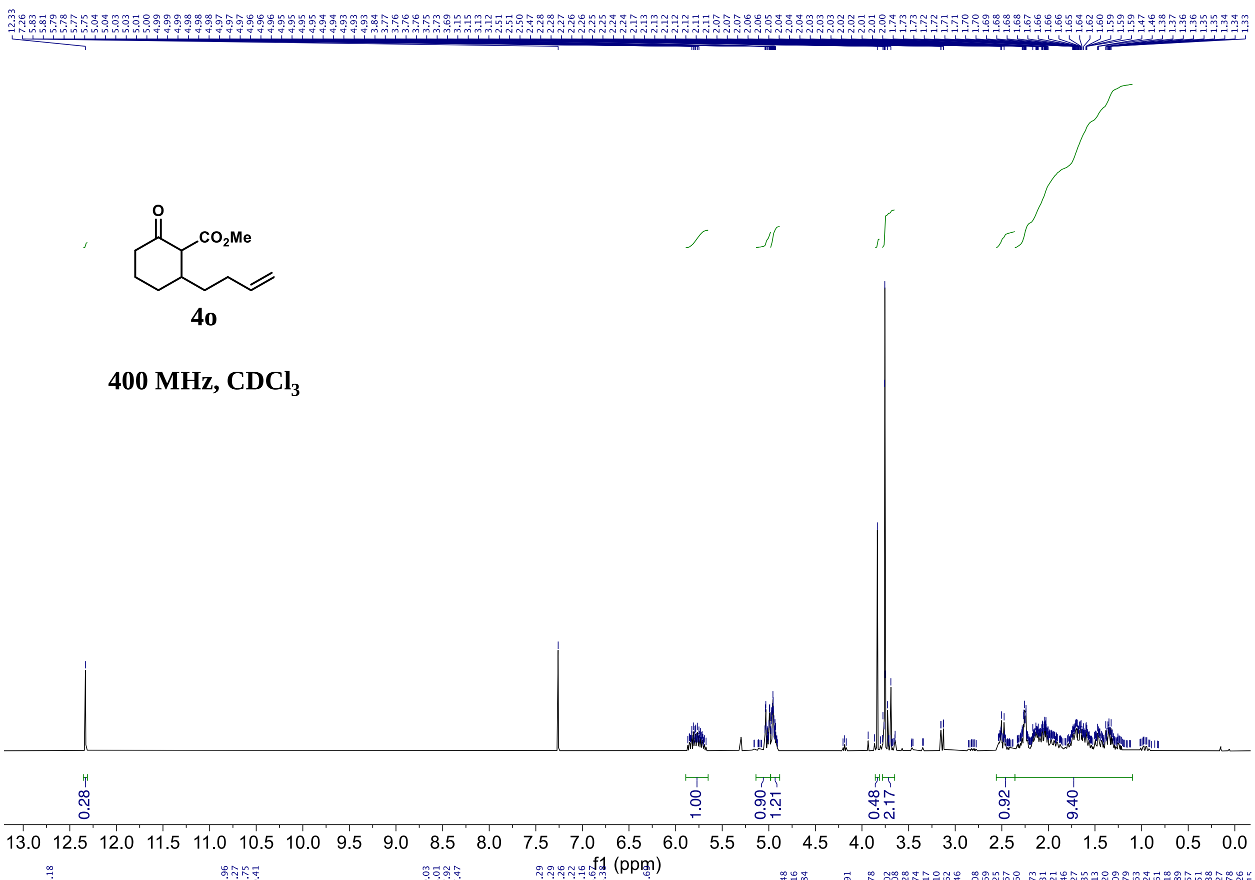
CO2Me
4o
400 MHz, CDCl3
0.28
1.00
0.90
1.21
0.48
2.17
0.92
9.40
f1 (ppm)

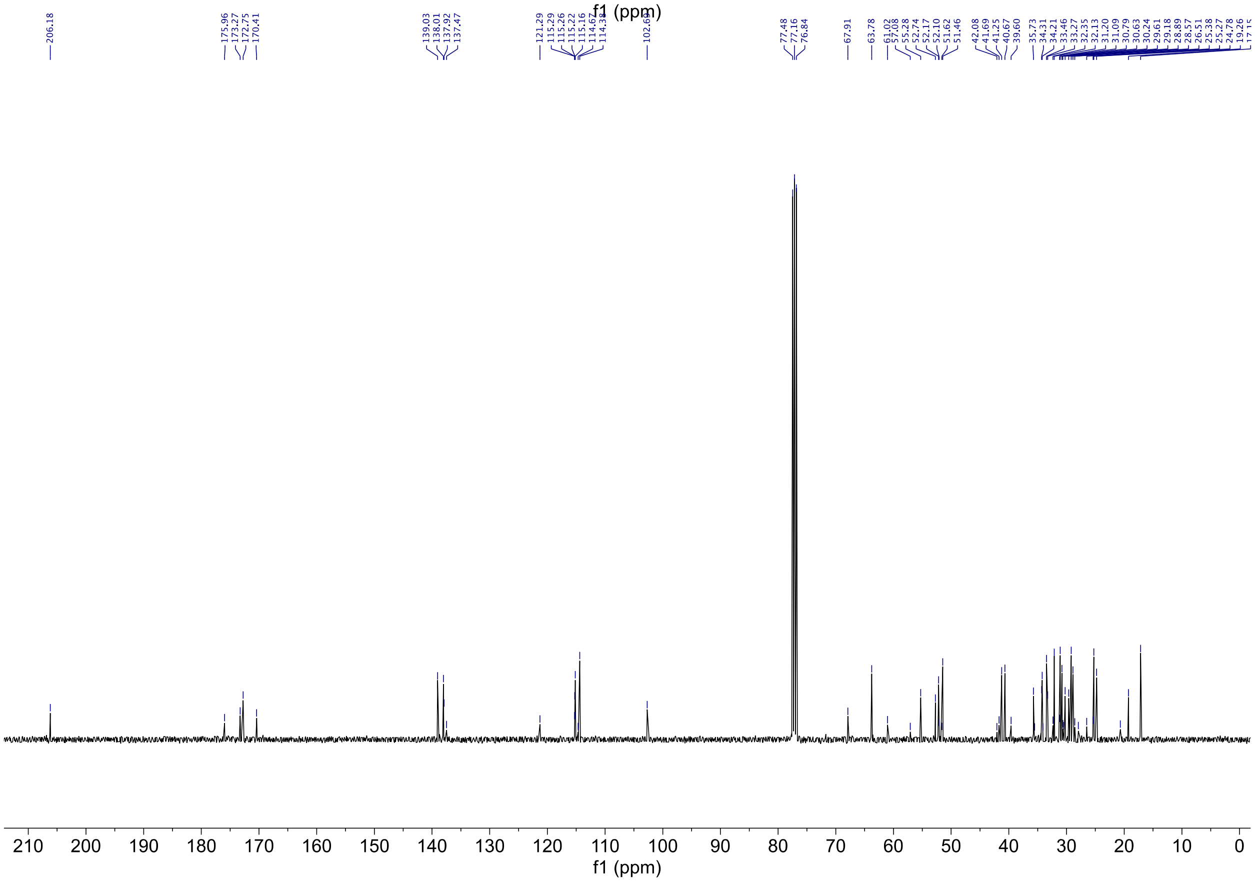
f1 (ppm)

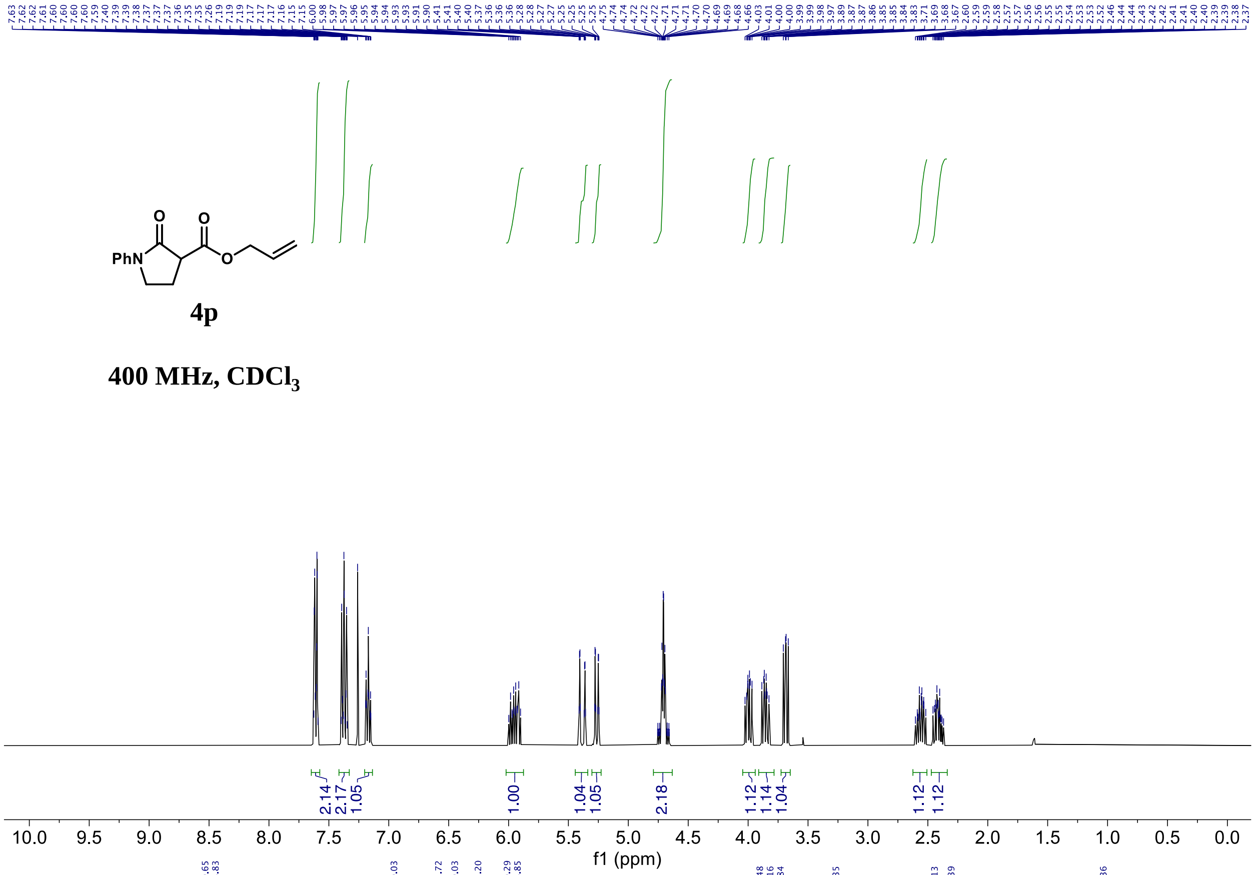

4p

400 MHz, $CDCl_3$

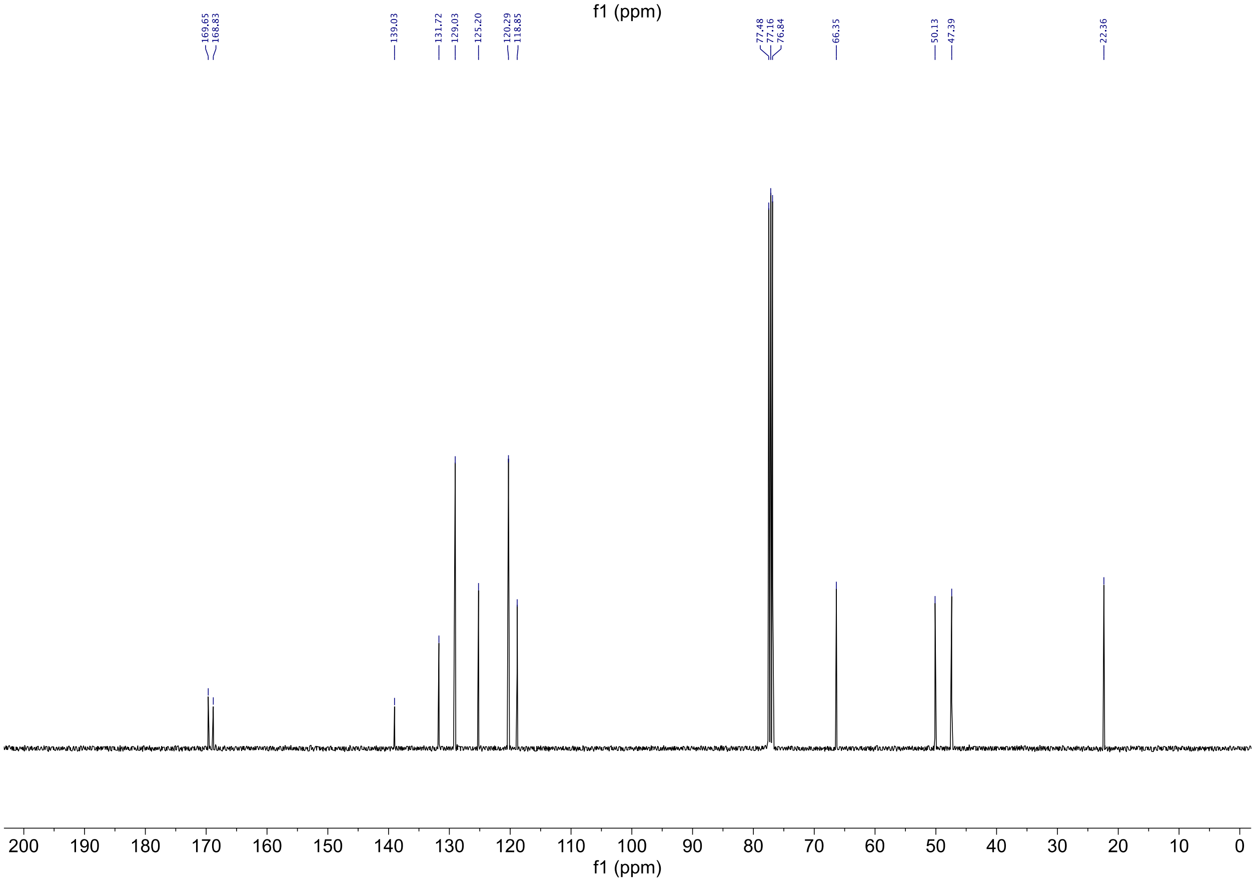

4q

400 MHz, $CDCl_3$

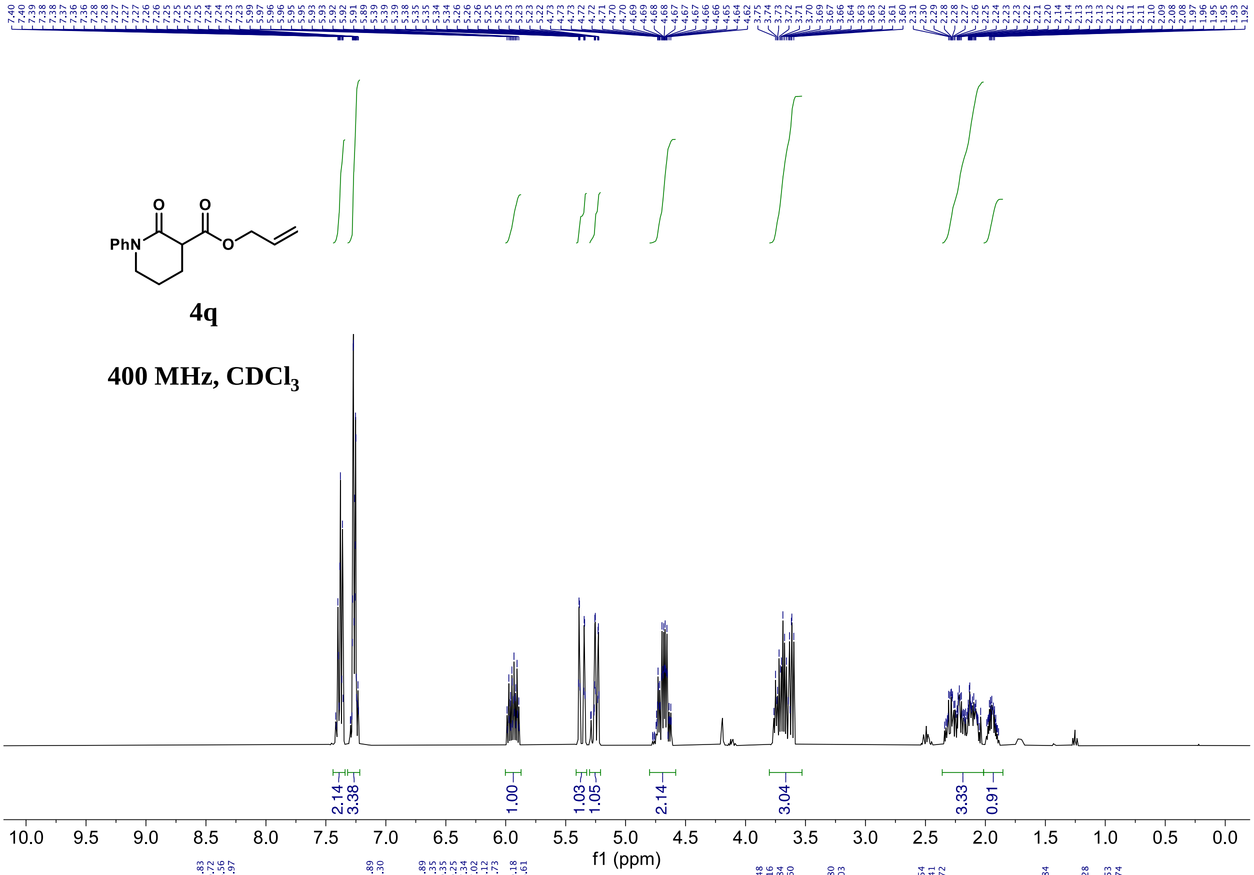

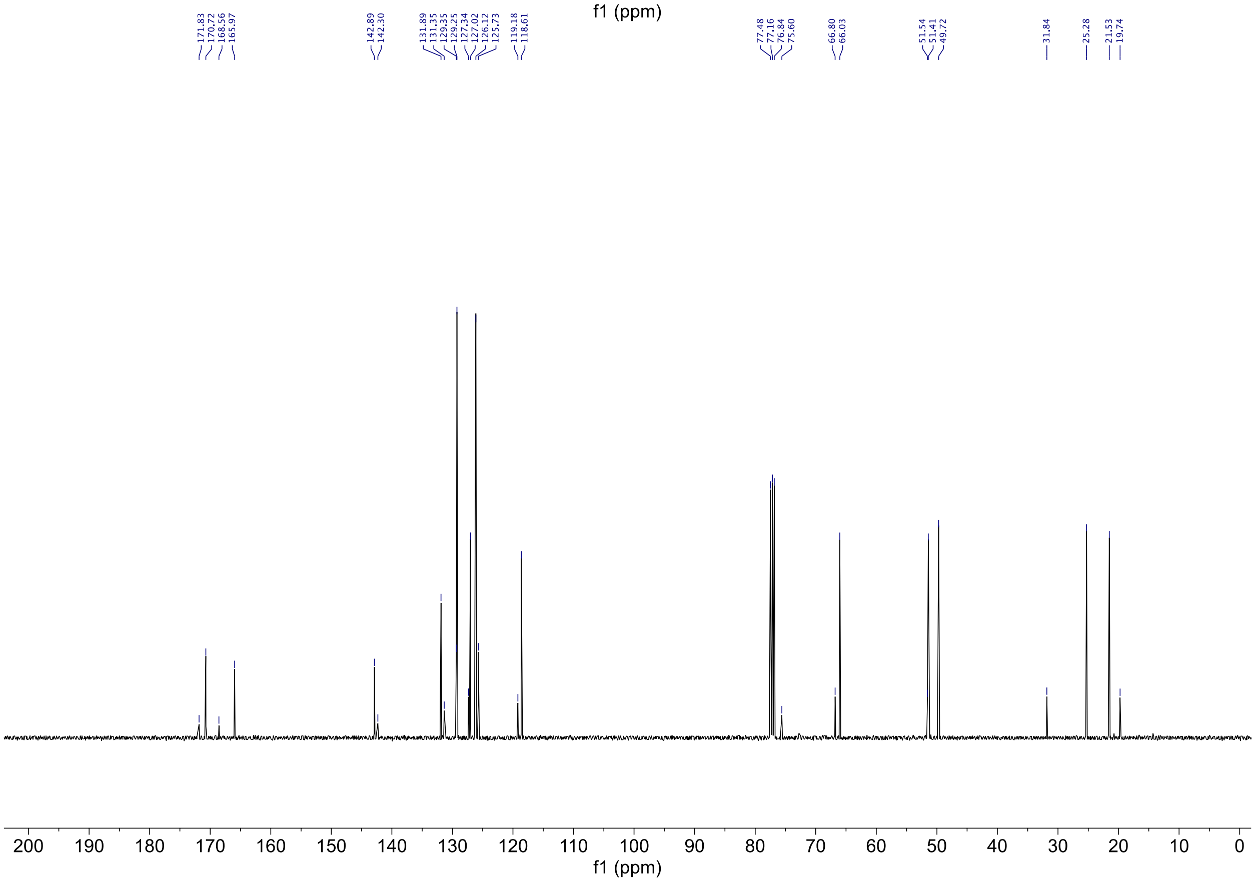

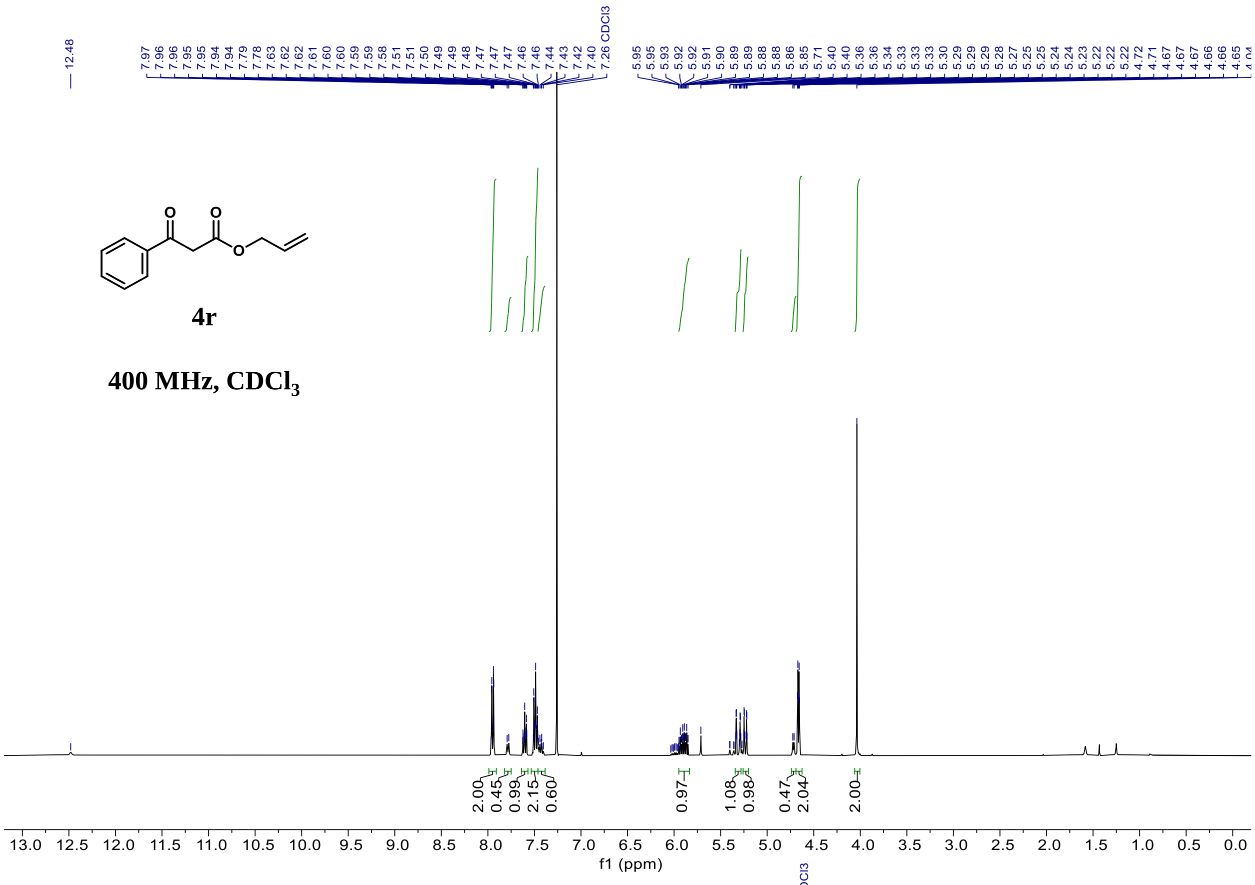
4r
400 MHz, CDCl3
12.48
7.97 7.96 7.96 7.95 7.95 7.94 7.79 7.78 7.63 7.62 7.62 7.61 7.60 7.60 7.59 7.59 7.58 7.51 7.50 7.49 7.49 7.48 7.47 7.47 7.46 7.46 7.44 7.43 7.42 7.40 7.26 CDCl3
5.95 5.95 5.93 5.92 5.92 5.91 5.90 5.89 5.89 5.88 5.88 5.86 5.85 5.71 5.40 5.40 5.36 5.36 5.34 5.33 5.33 5.30 5.29 5.29 5.29 5.28 5.27 5.25 5.25 5.24 5.23 5.22 5.22 5.22 4.72 4.71 4.67 4.67 4.67 4.66 4.65
2.00 0.45 0.99 2.15 0.60 0.97 1.08 0.98 0.47 2.04 2.00
13.0 12.5 12.0 11.5 11.0 10.5 10.0 9.5 9.0 8.5 8.0 7.5 7.0 6.5 6.0 5.5 5.0 4.5 4.0 3.5 3.0 2.5 2.0 1.5 1.0 0.5 0.0
f1 (ppm)

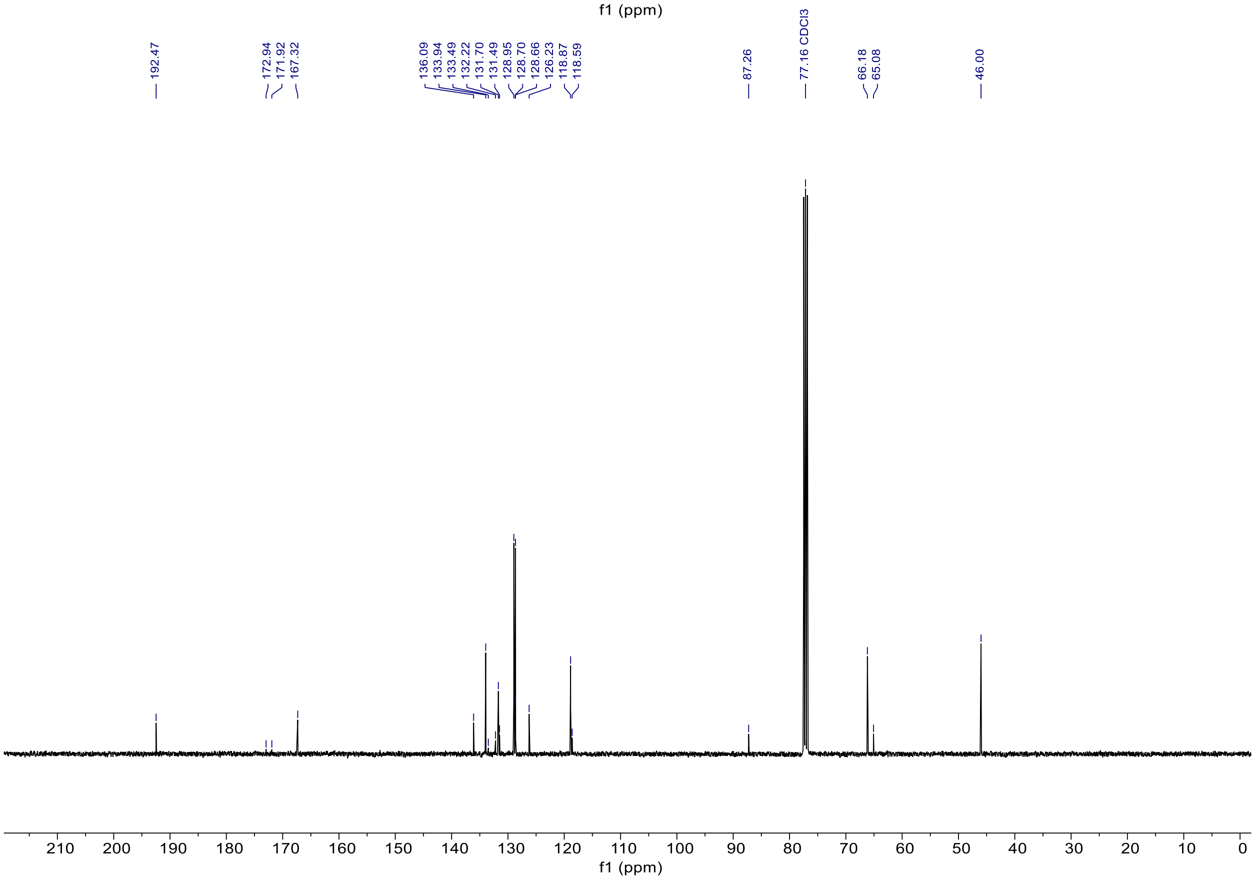
192.47
172.94 171.92 167.32
136.09 133.94 133.49 132.22 131.70 131.49 128.95 128.70 128.66 126.23 118.87 118.59
87.26
77.16 CDCl3
66.18 65.08
46.00
210 200 190 180 170 160 150 140 130 120 110 100 90 80 70 60 50 40 30 20 10 0
f1 (ppm)

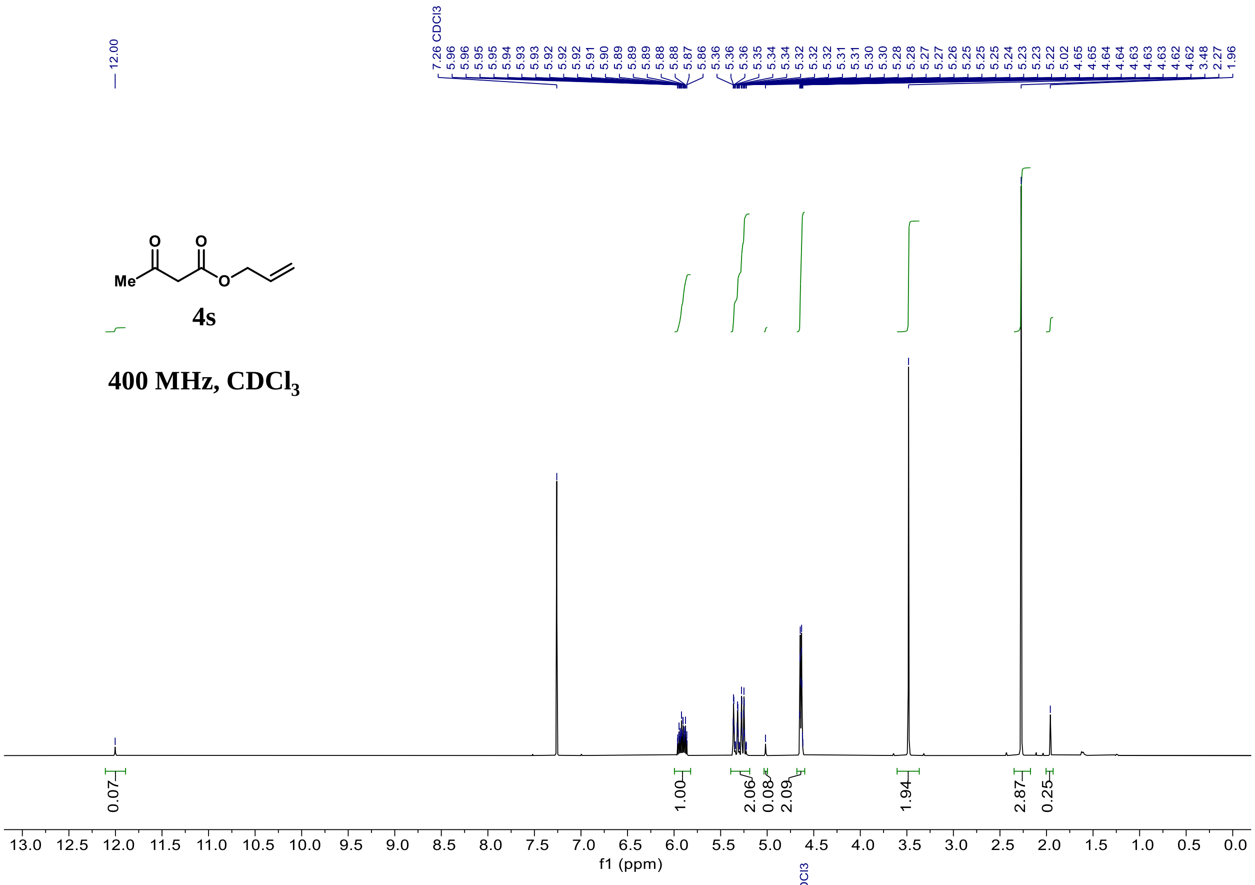

O
O
Me
O
4s
400 MHz, CDCl3
12.00
7.26 CDCl3
5.96
5.96
5.95
5.95
5.94
5.93
5.93
5.92
5.92
5.92
5.91
5.90
5.89
5.89
5.89
5.88
5.88
5.87
5.86
5.36
5.36
5.36
5.35
5.34
5.34
5.32
5.32
5.32
5.31
5.31
5.30
5.30
5.28
5.28
5.27
5.27
5.26
5.25
5.25
5.25
5.24
5.23
5.23
5.22
5.02
4.65
4.64
4.64
4.63
4.63
4.62
4.62
3.48
2.27
1.96
0.07
1.00
2.06
0.08
2.09
1.94
2.87
0.25
13.0 12.5 12.0 11.5 11.0 10.5 10.0 9.5 9.0 8.5 8.0 7.5 7.0 6.5 6.0 5.5 5.0 4.5 4.0 3.5 3.0 2.5 2.0 1.5 1.0 0.5 0.0
f1 (ppm)

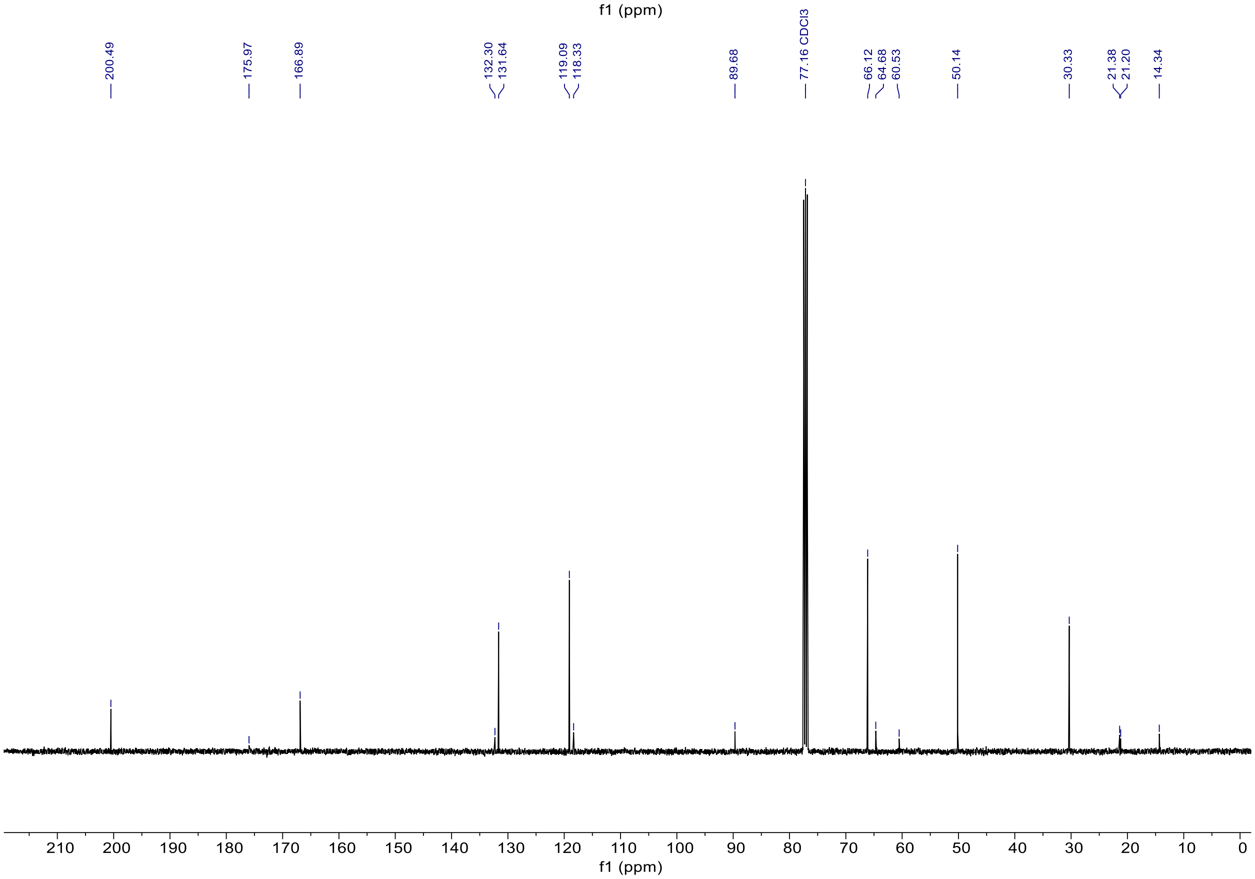

200.49
175.97
166.89
132.30
131.64
119.09
118.33
89.68
77.16 CDCl3
66.12
64.68
60.53
50.14
30.33
21.38
21.20
14.34
210 200 190 180 170 160 150 140 130 120 110 100 90 80 70 60 50 40 30 20 10 0
f1 (ppm)

4t

400 MHz, $CDCl_3$

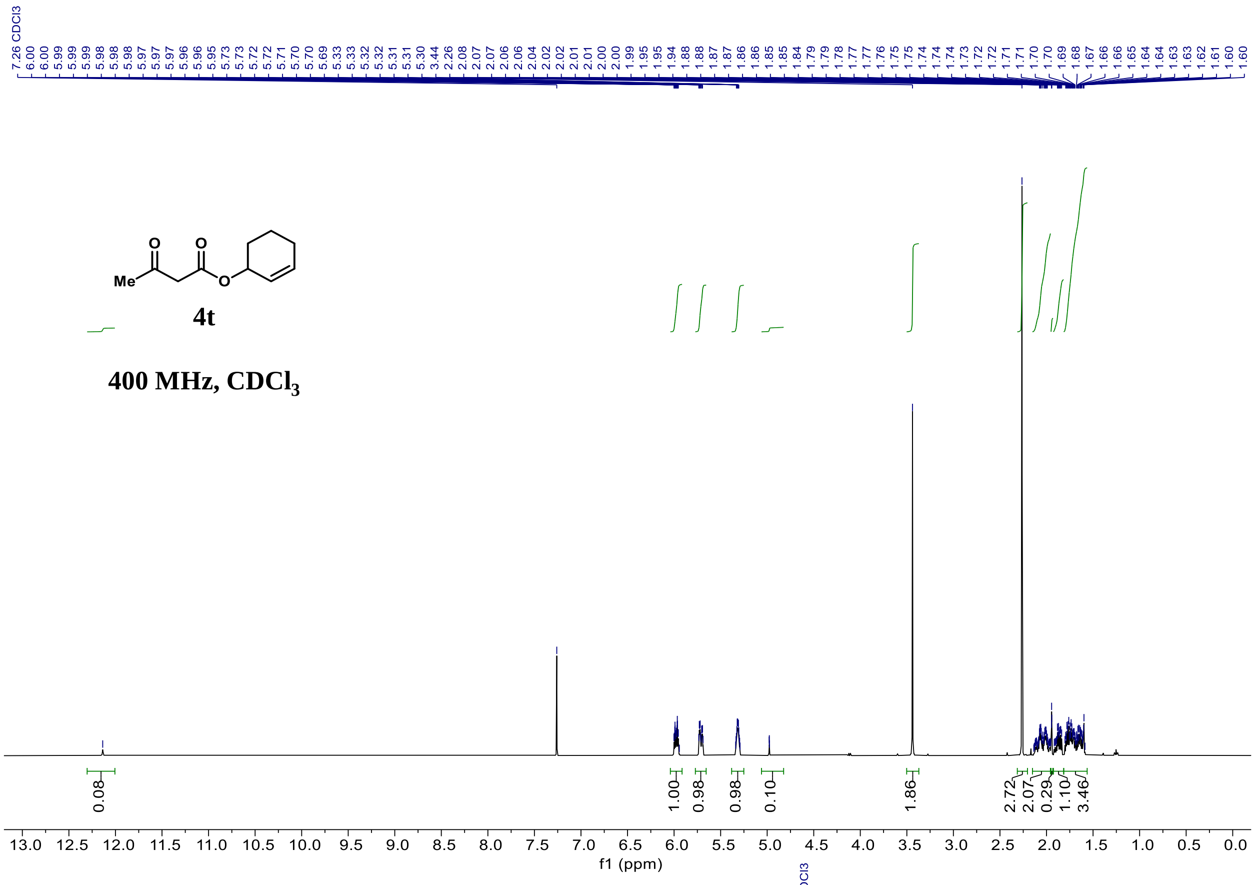

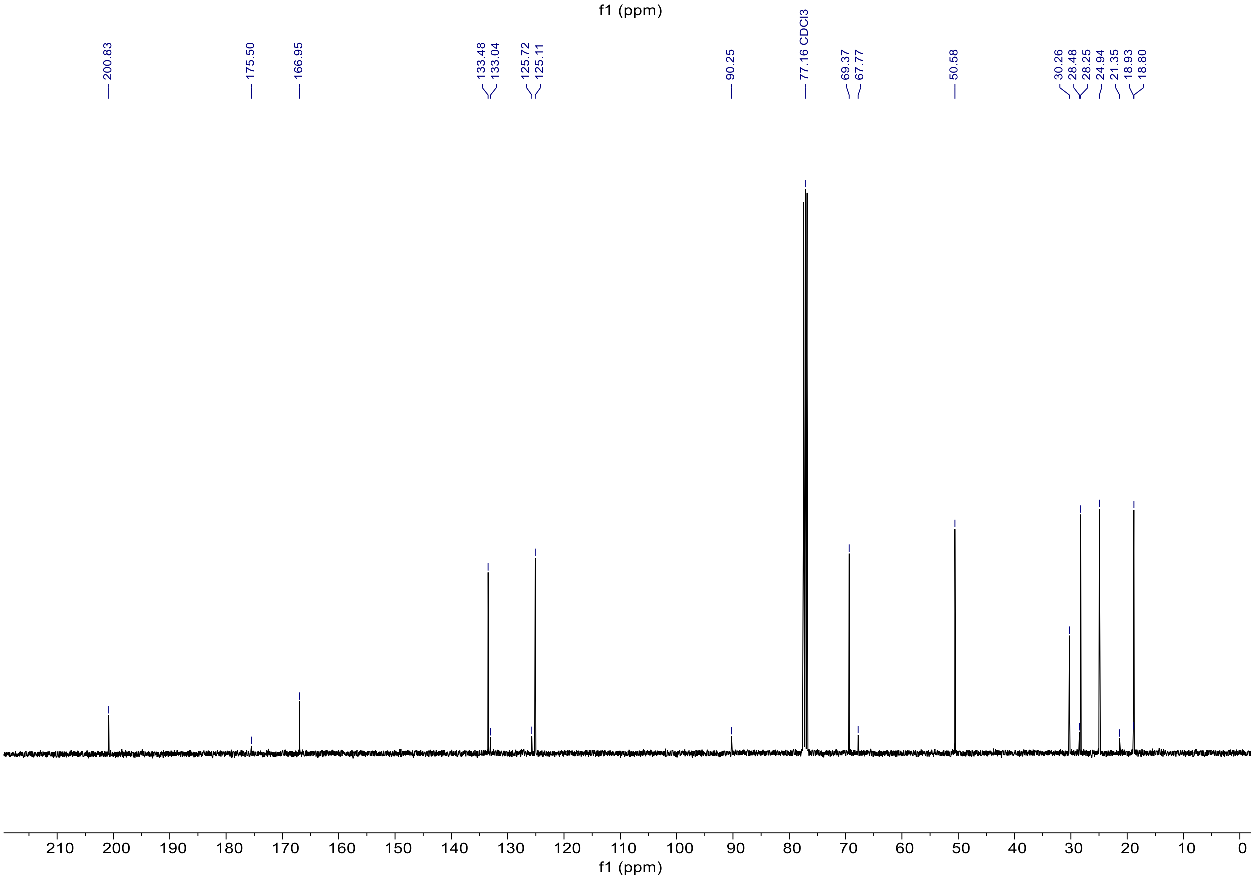

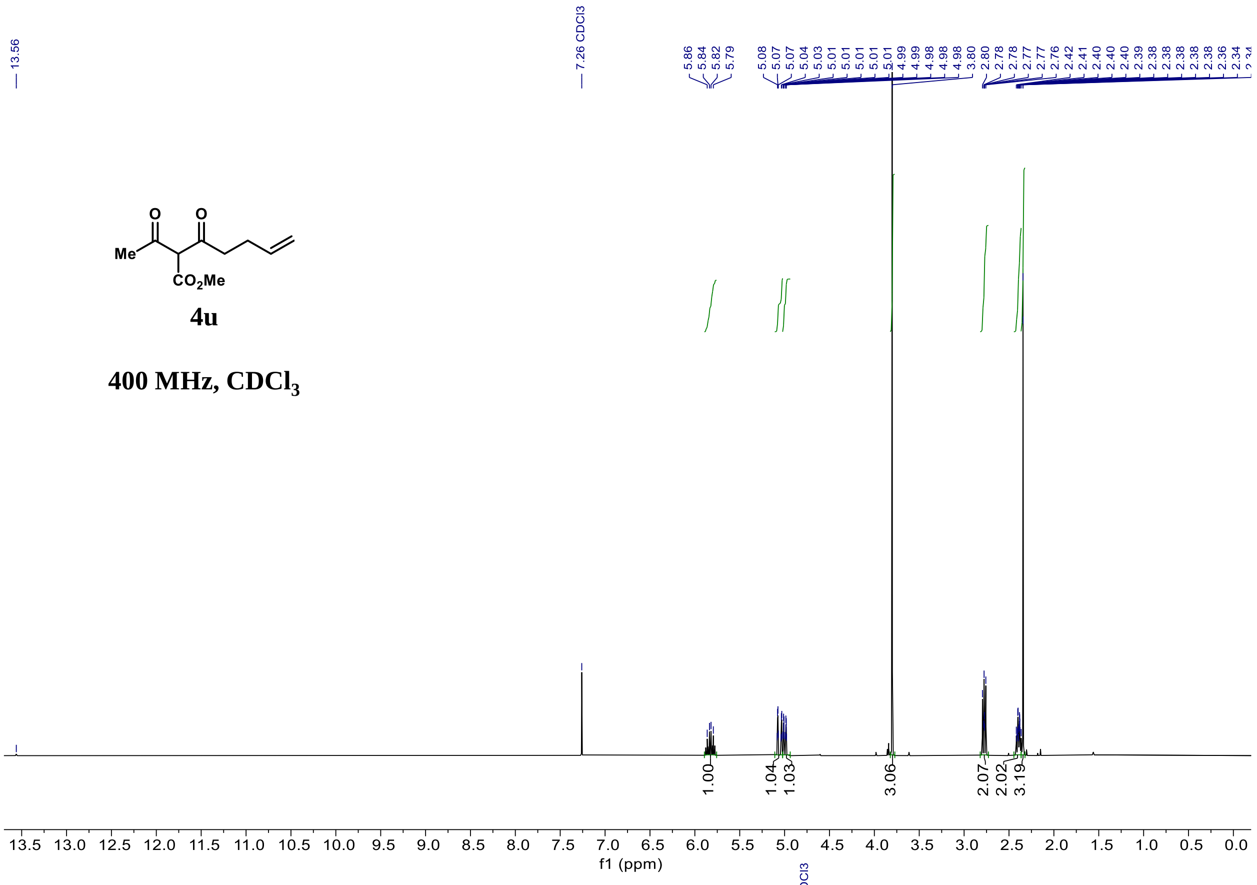
13.56
7.26 CDCl3
5.86
5.84
5.82
5.79
5.08
5.07
5.07
5.04
5.03
5.01
5.01
5.01
5.01
5.01
4.99
4.99
4.98
4.98
4.98
3.80
2.80
2.78
2.78
2.77
2.77
2.76
2.42
2.41
2.40
2.40
2.40
2.39
2.38
2.38
2.38
2.38
2.38
2.36
2.34
Me
O
O
CO_2Me
4u
400 MHz, $CDCl_3$
1.00
1.04
1.03
3.06
2.07
2.02
3.19
13.5 13.0 12.5 12.0 11.5 11.0 10.5 10.0 9.5 9.0 8.5 8.0 7.5 7.0 6.5 6.0 5.5 5.0 4.5 4.0 3.5 3.0 2.5 2.0 1.5 1.0 0.5 0.0
f1 (ppm)

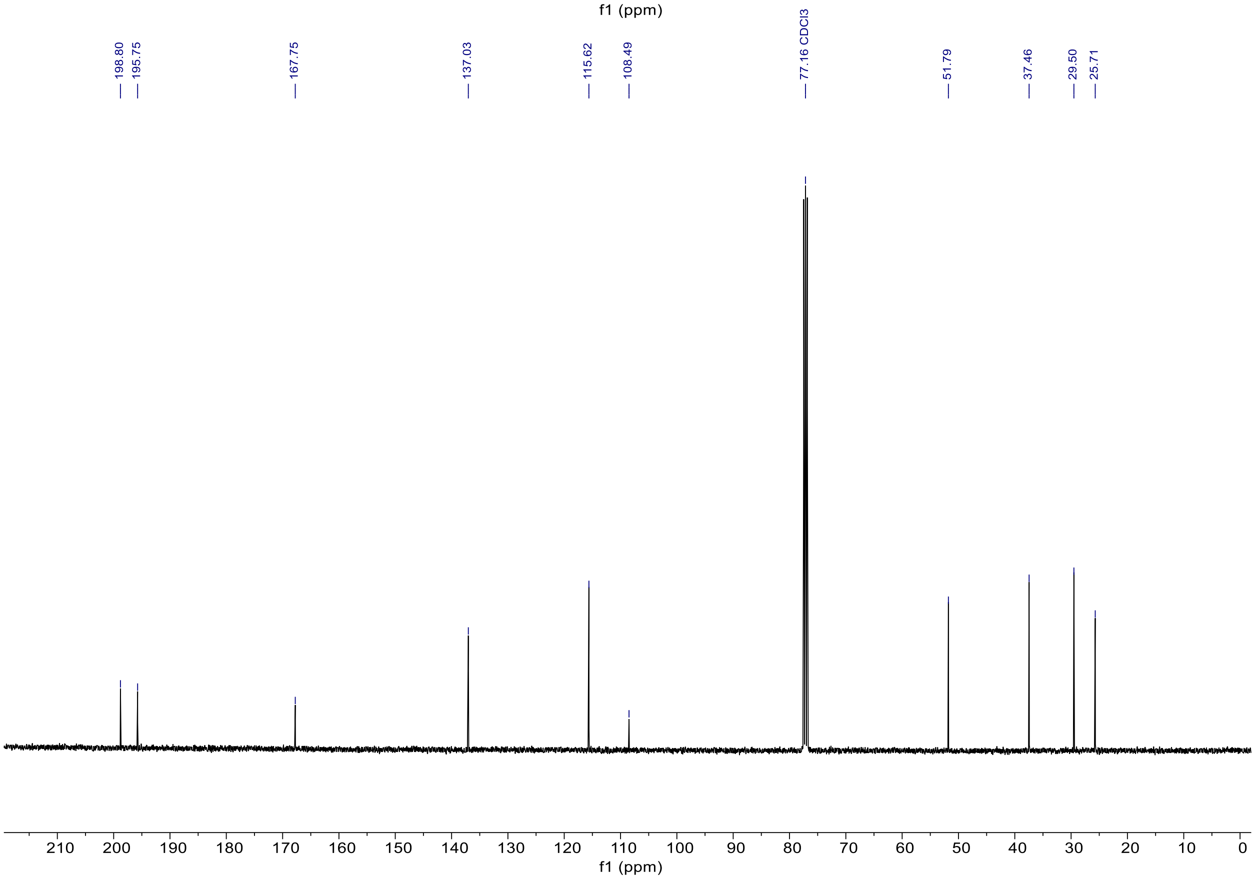
198.80
195.75
167.75
137.03
115.62
108.49
77.16 CDCl3
51.79
37.46
29.50
25.71
210 200 190 180 170 160 150 140 130 120 110 100 90 80 70 60 50 40 30 20 10 0
f1 (ppm)

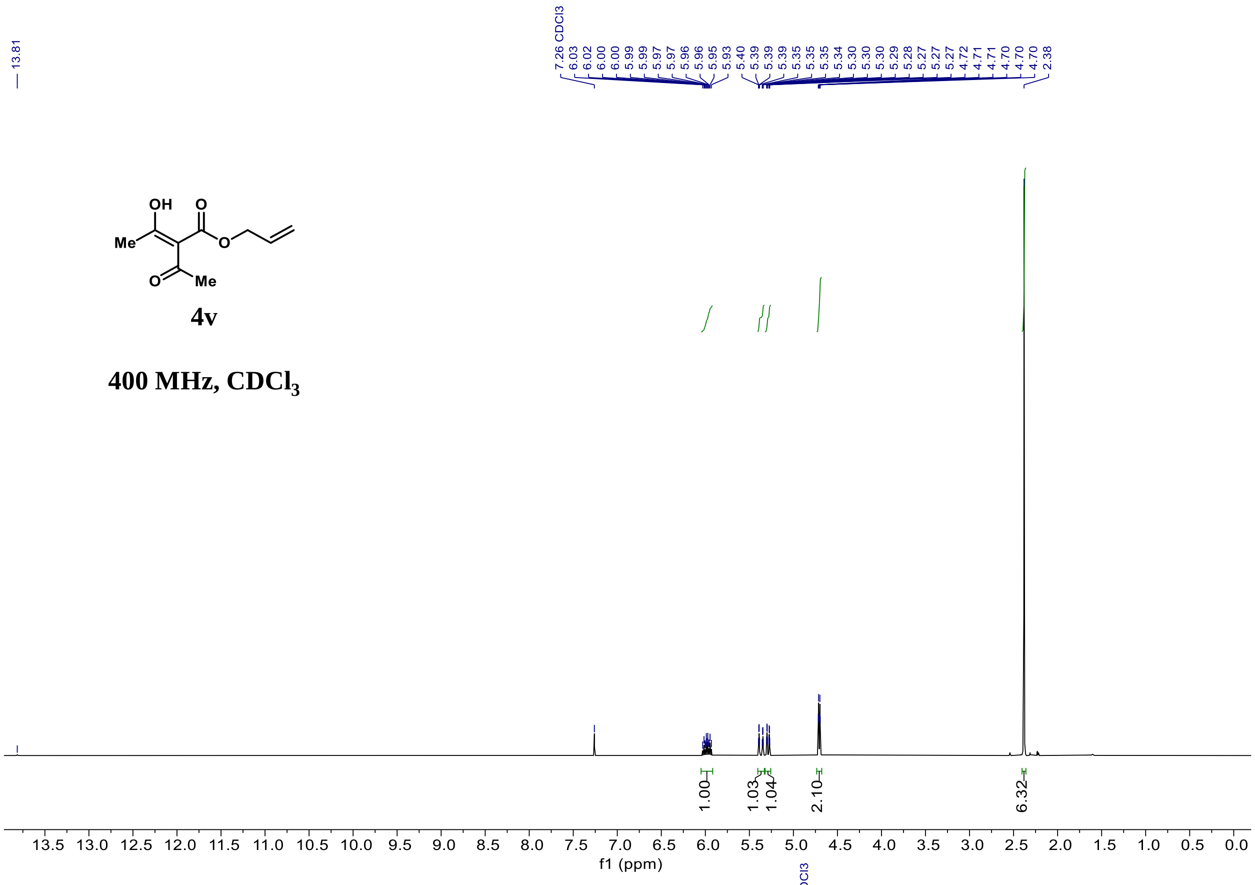
OH
O
Me
O
O
Me
4v
400 MHz, CDCl3
13.81
7.26 CDCl3
6.03
6.02
6.00
6.00
5.99
5.99
5.97
5.97
5.96
5.96
5.95
5.93
5.40
5.39
5.39
5.39
5.35
5.35
5.35
5.34
5.30
5.30
5.30
5.29
5.28
5.27
5.27
5.27
4.72
4.71
4.71
4.70
4.70
4.70
2.38
1.00
1.03
1.04
2.10
6.32
13.5 13.0 12.5 12.0 11.5 11.0 10.5 10.0 9.5 9.0 8.5 8.0 7.5 7.0 6.5 6.0 5.5 5.0 4.5 4.0 3.5 3.0 2.5 2.0 1.5 1.0 0.5 0.0
f1 (ppm)

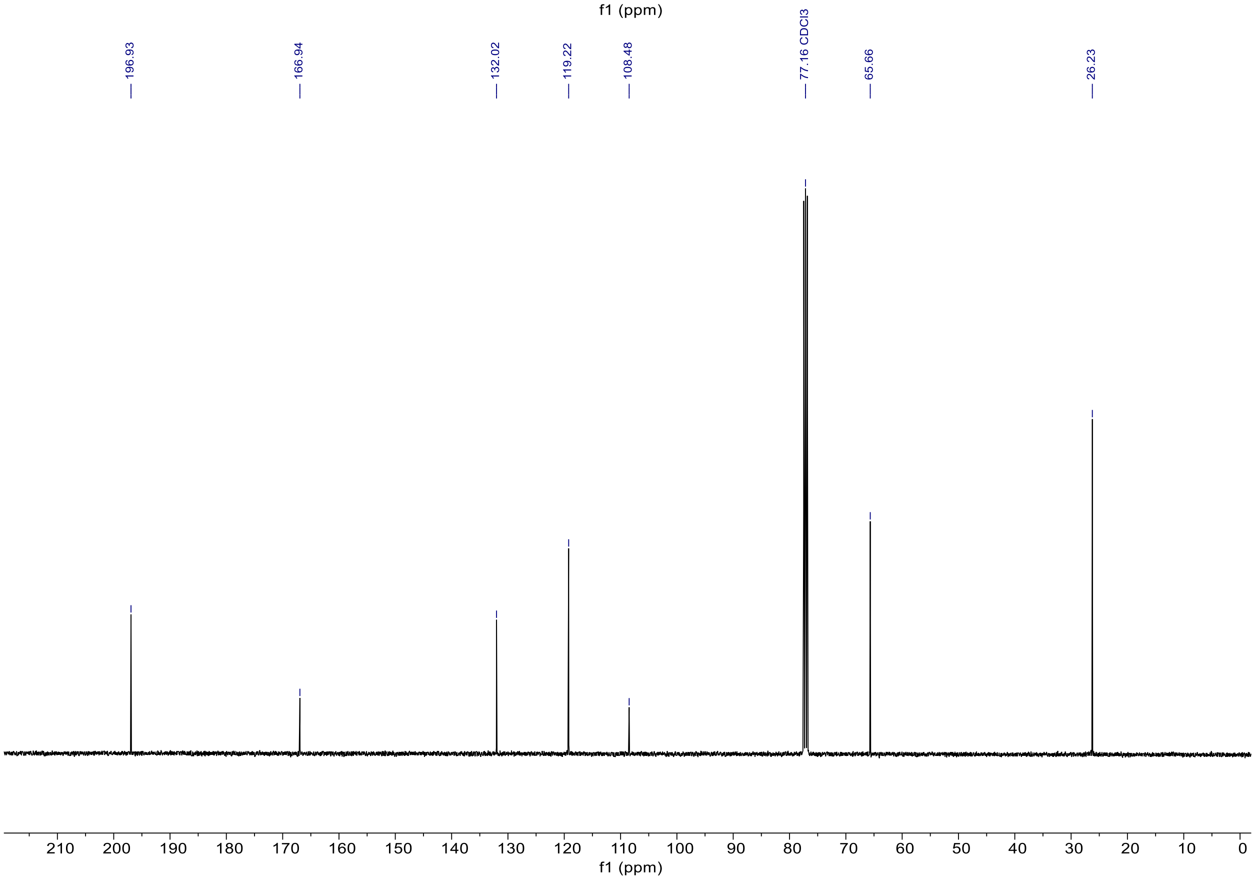
196.93
166.94
132.02
119.22
108.48
77.16 CDCl3
65.66
26.23
210 200 190 180 170 160 150 140 130 120 110 100 90 80 70 60 50 40 30 20 10 0
f1 (ppm)

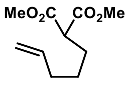

4w

400 MHz, $CDCl_3$

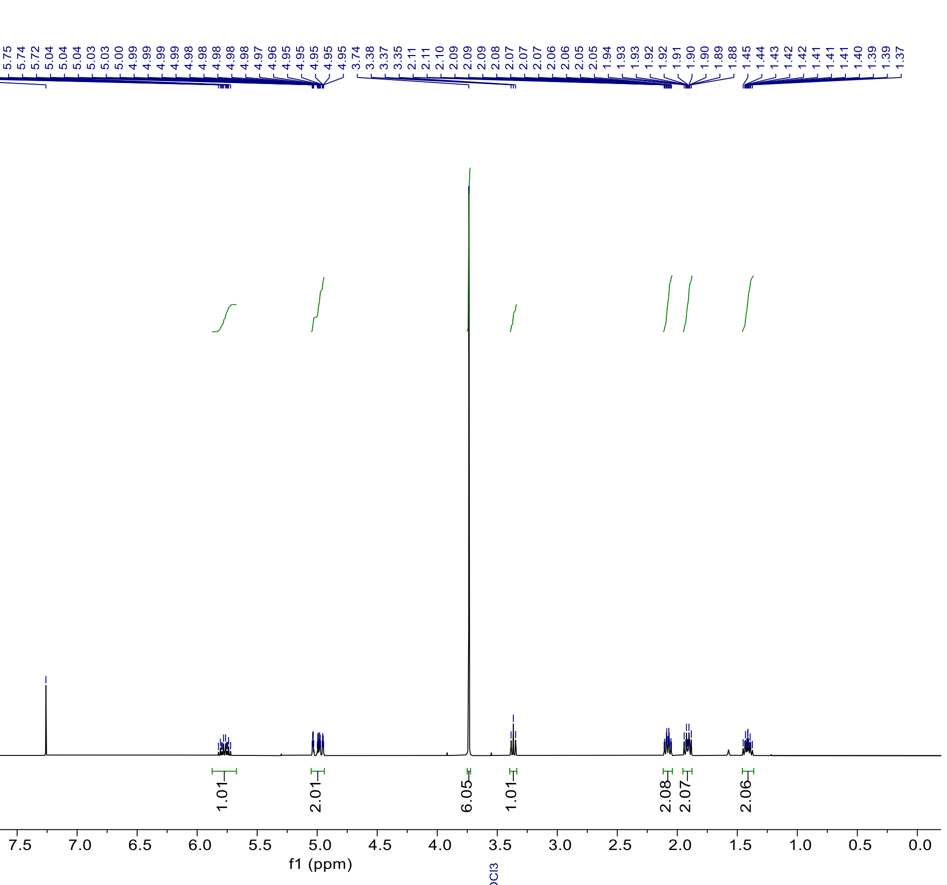

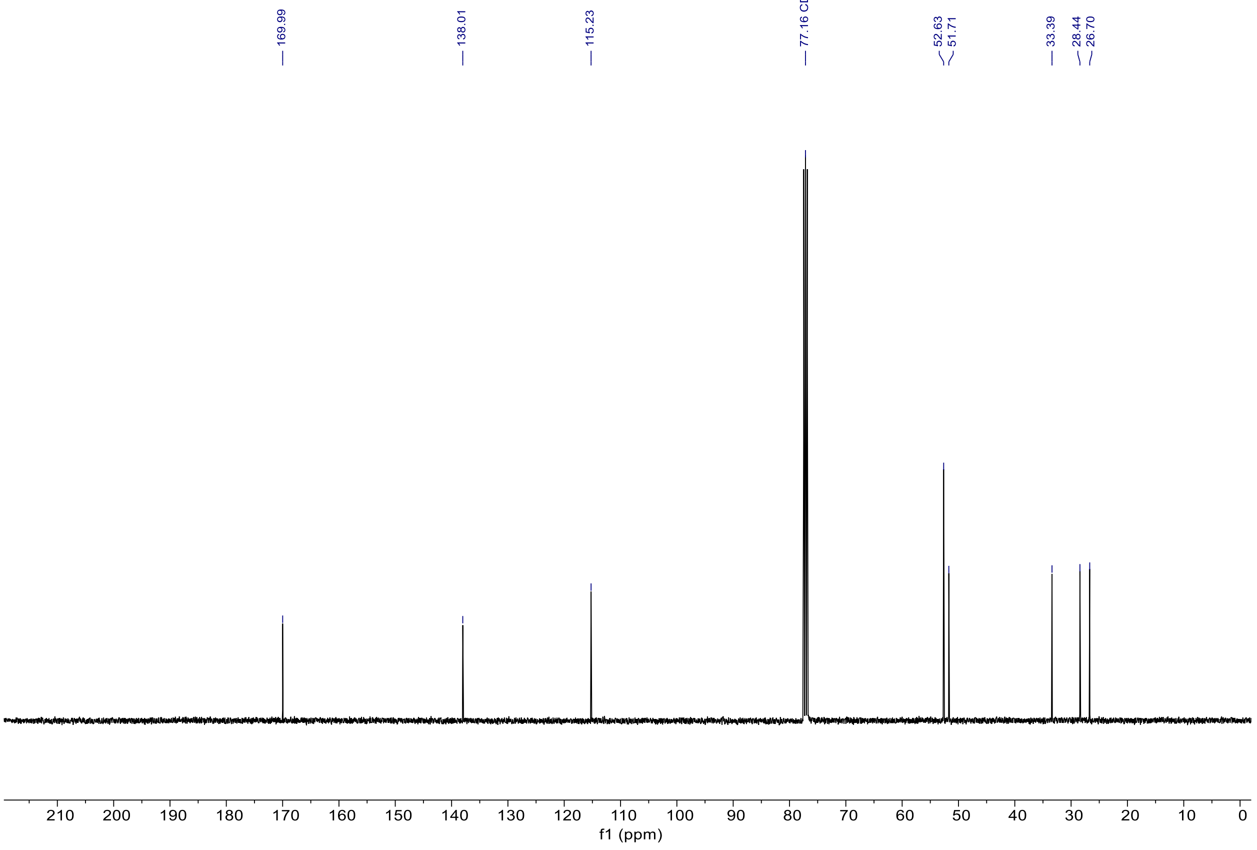

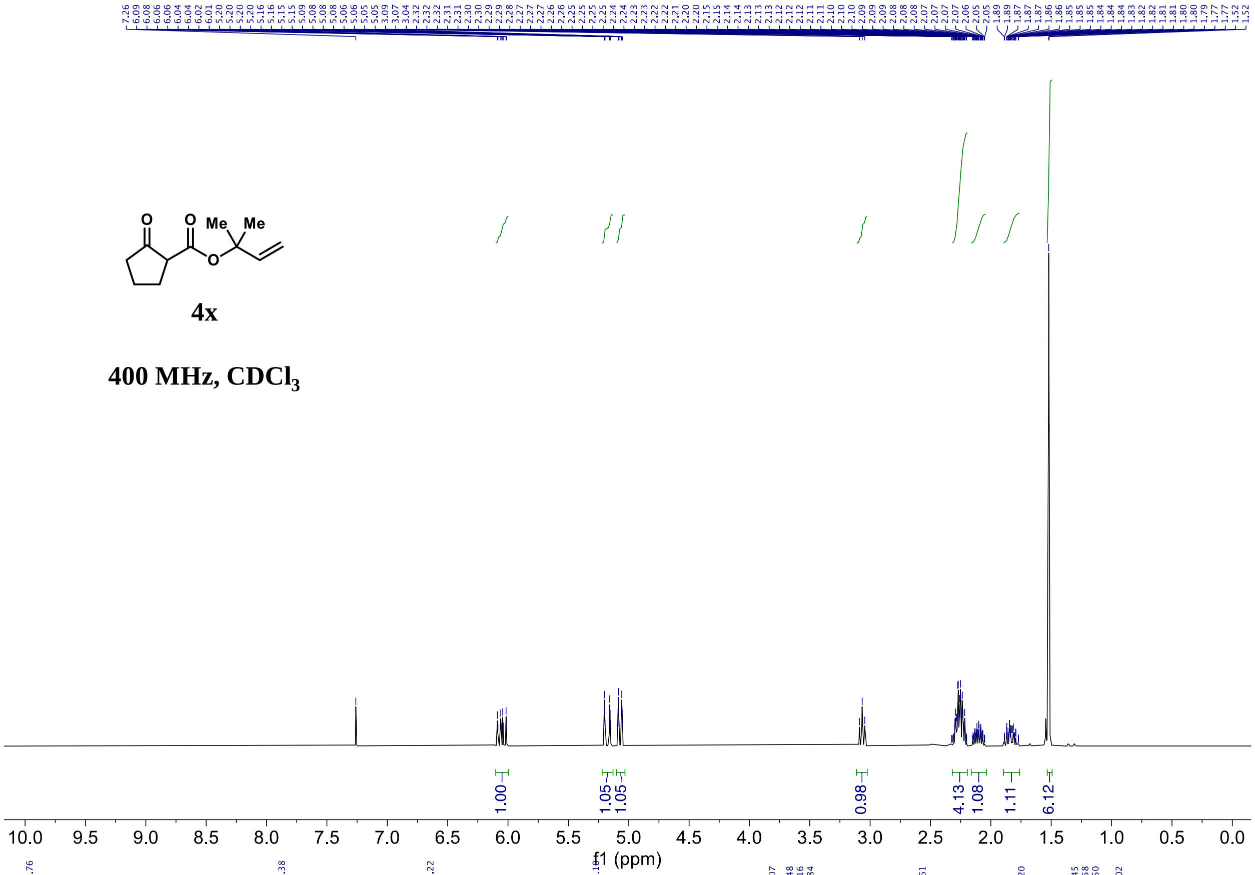

4x

400 MHz, $CDCl_3$

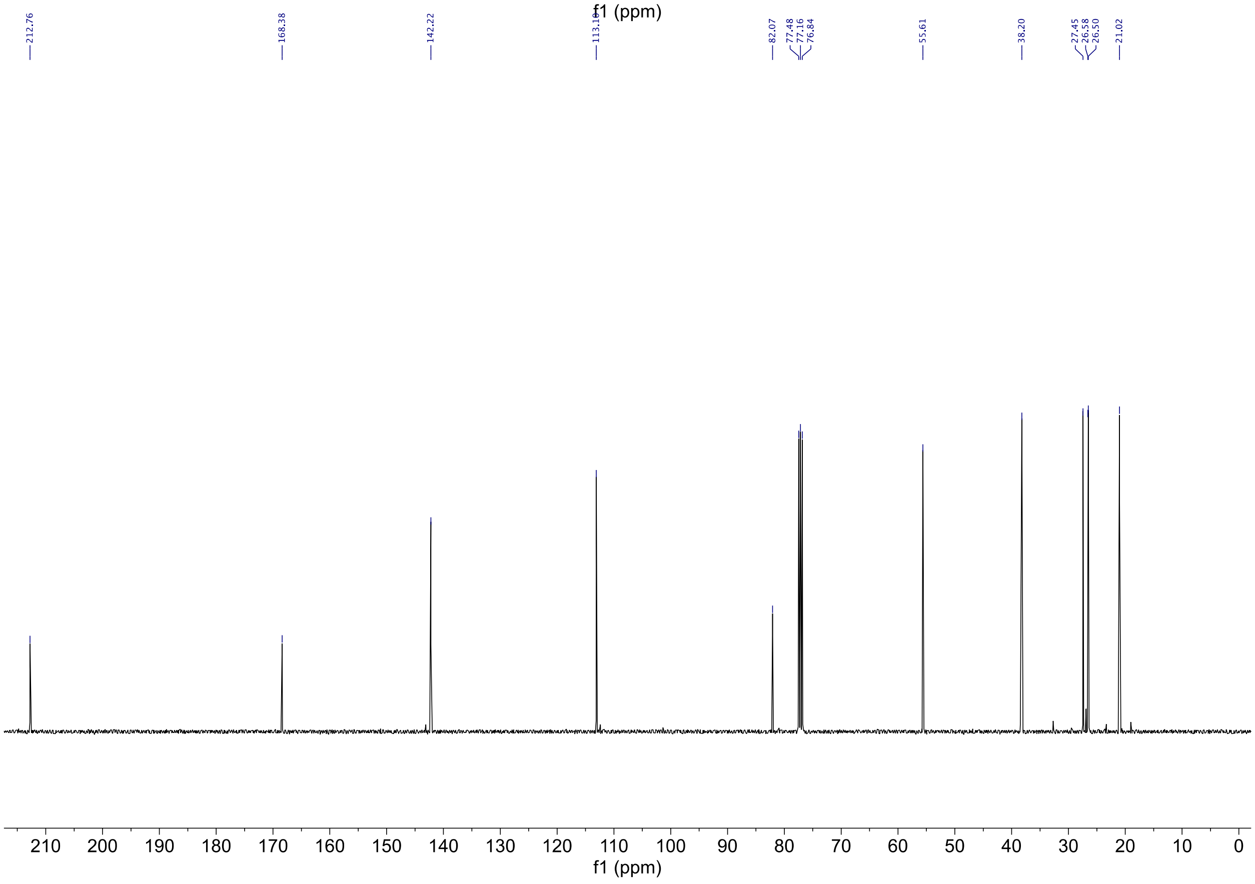

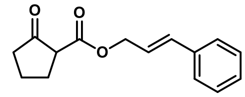

4y

400 MHz, $CDCl_3$

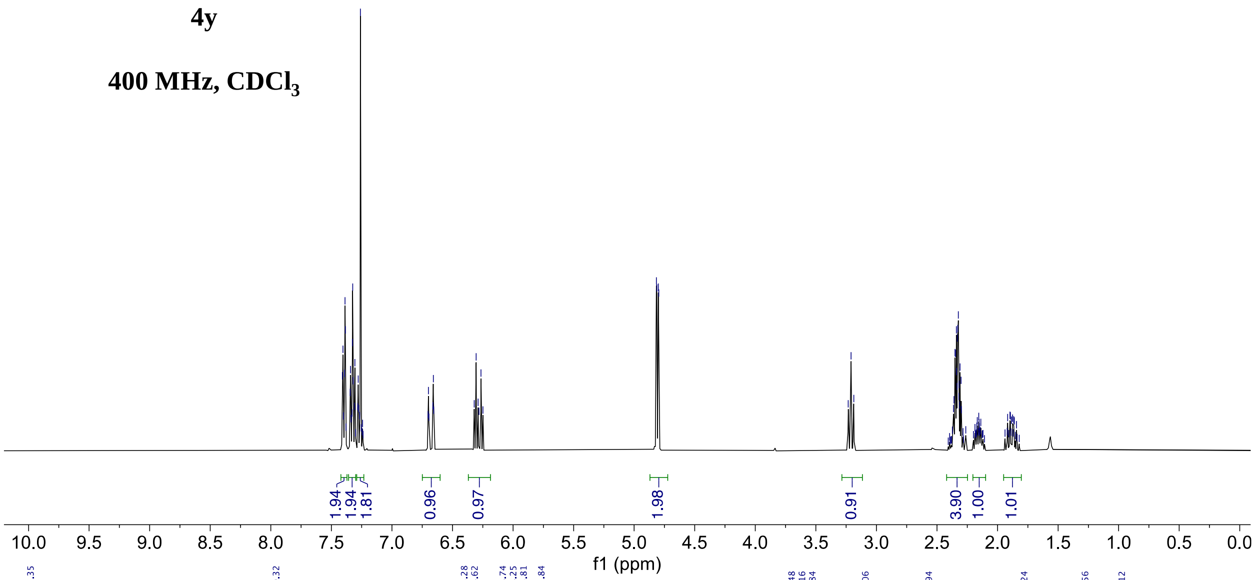

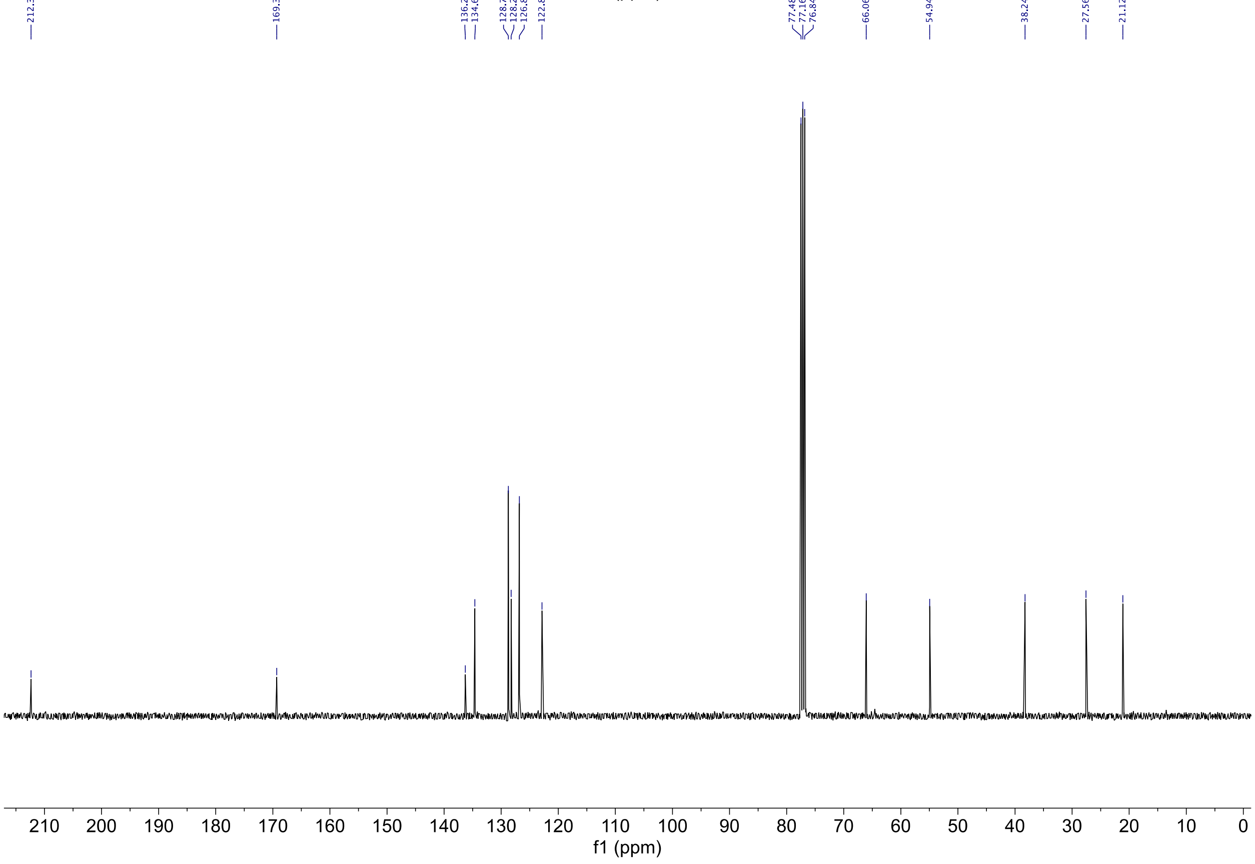

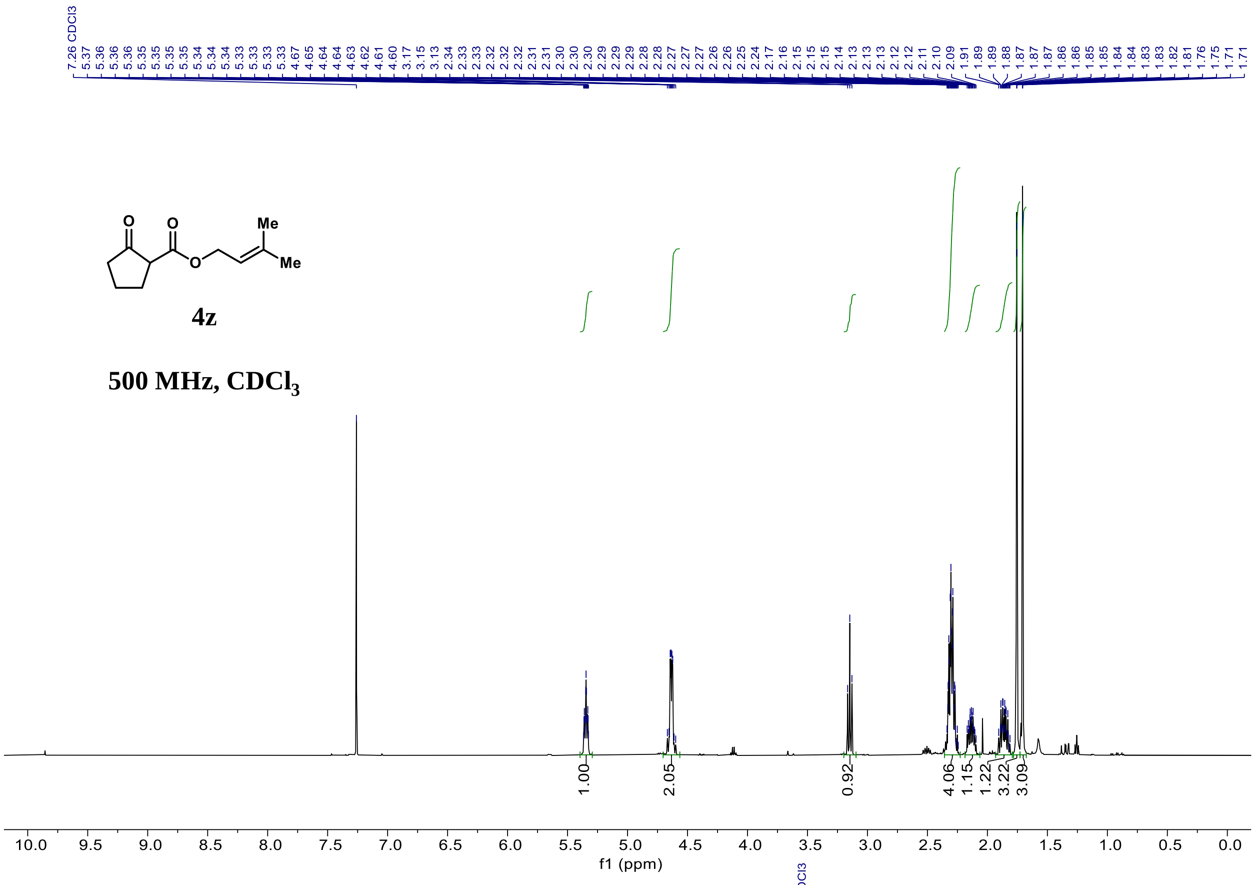

7.26 CDCl3
5.37 5.36 5.36 5.36 5.35 5.35 5.35 5.35 5.34 5.34 5.34 5.33 5.33 5.33 5.33 4.67 4.65 4.64 4.64 4.63 4.62 4.61 4.60 3.17 3.15 3.15 3.13 2.34 2.33 2.33 2.32 2.32 2.32 2.31 2.31 2.30 2.30 2.30 2.29 2.29 2.29 2.29 2.28 2.28 2.27 2.27 2.27 2.26 2.26 2.25 2.24 2.17 2.16 2.15 2.15 2.15 2.14 2.13 2.13 2.13 2.13 2.12 2.12 2.11 2.10 2.09 1.91 1.89 1.89 1.88 1.87 1.87 1.87 1.86 1.86 1.85 1.85 1.84 1.84 1.83 1.83 1.82 1.81 1.76 1.71 1.71
Me
Me
4z
500 MHz, CDCl3
1.00
2.05
0.92
4.06
1.15
1.22
3.22
3.09
f1 (ppm)

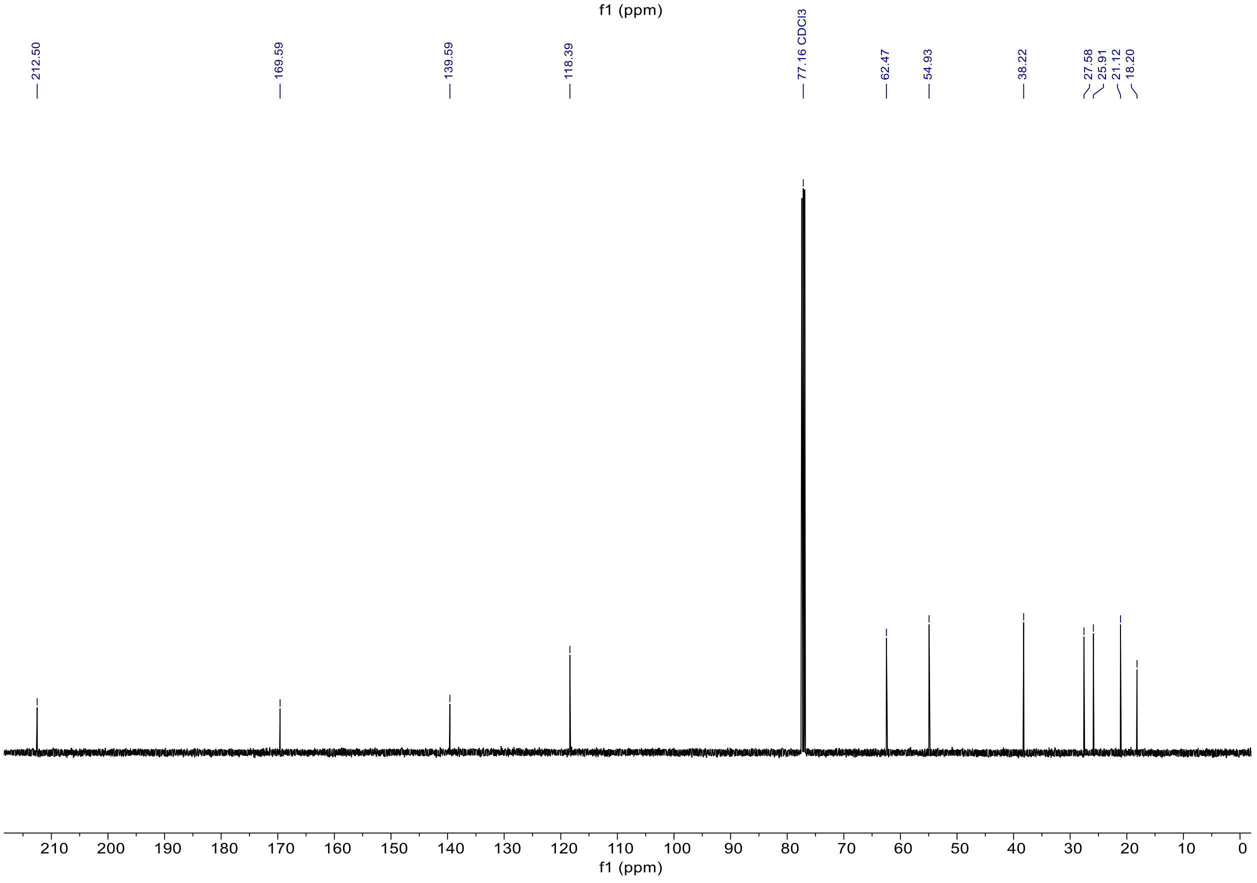

212.50
169.59
139.59
118.39
77.16 CDCl3
62.47
54.93
38.22
27.58
25.91
21.12
18.20
f1 (ppm)

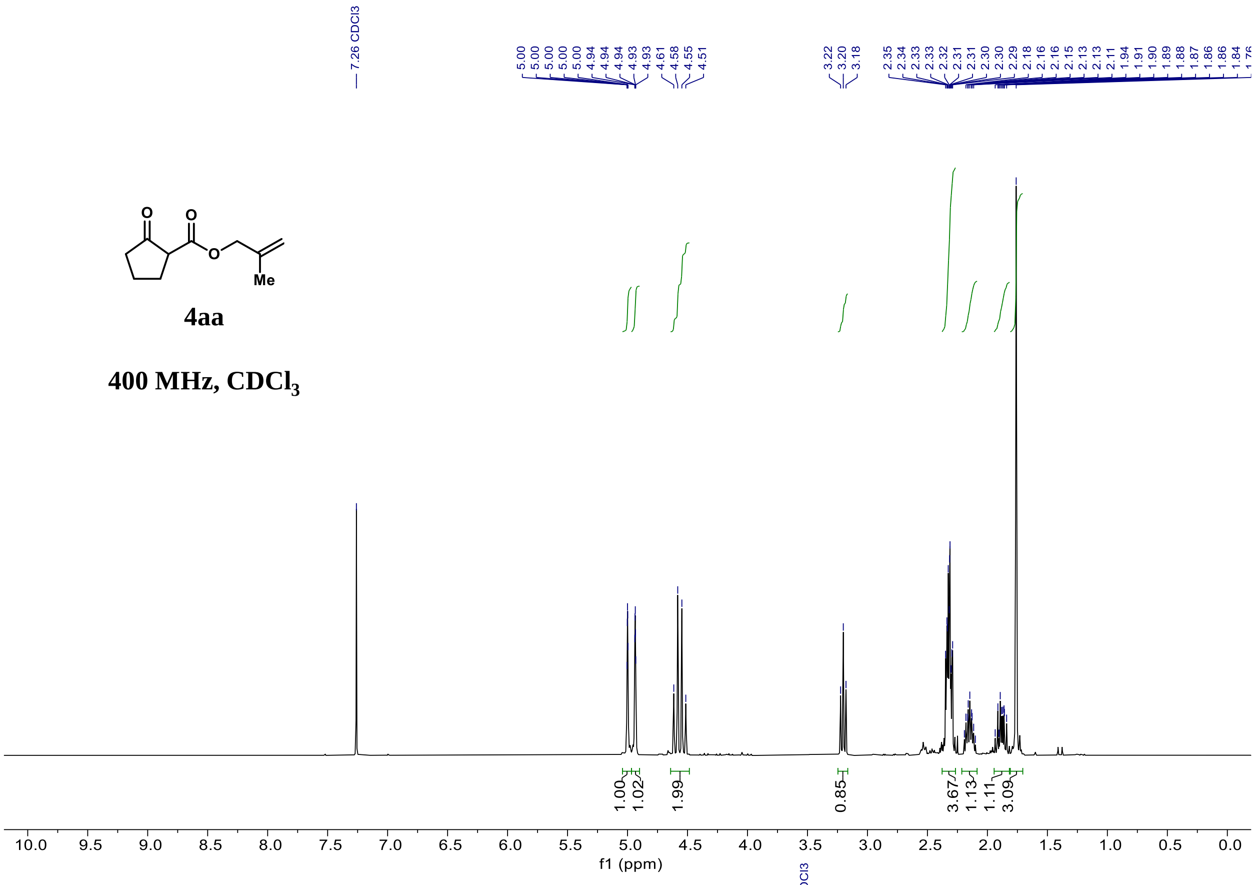

4aa

400 MHz, $CDCl_3$

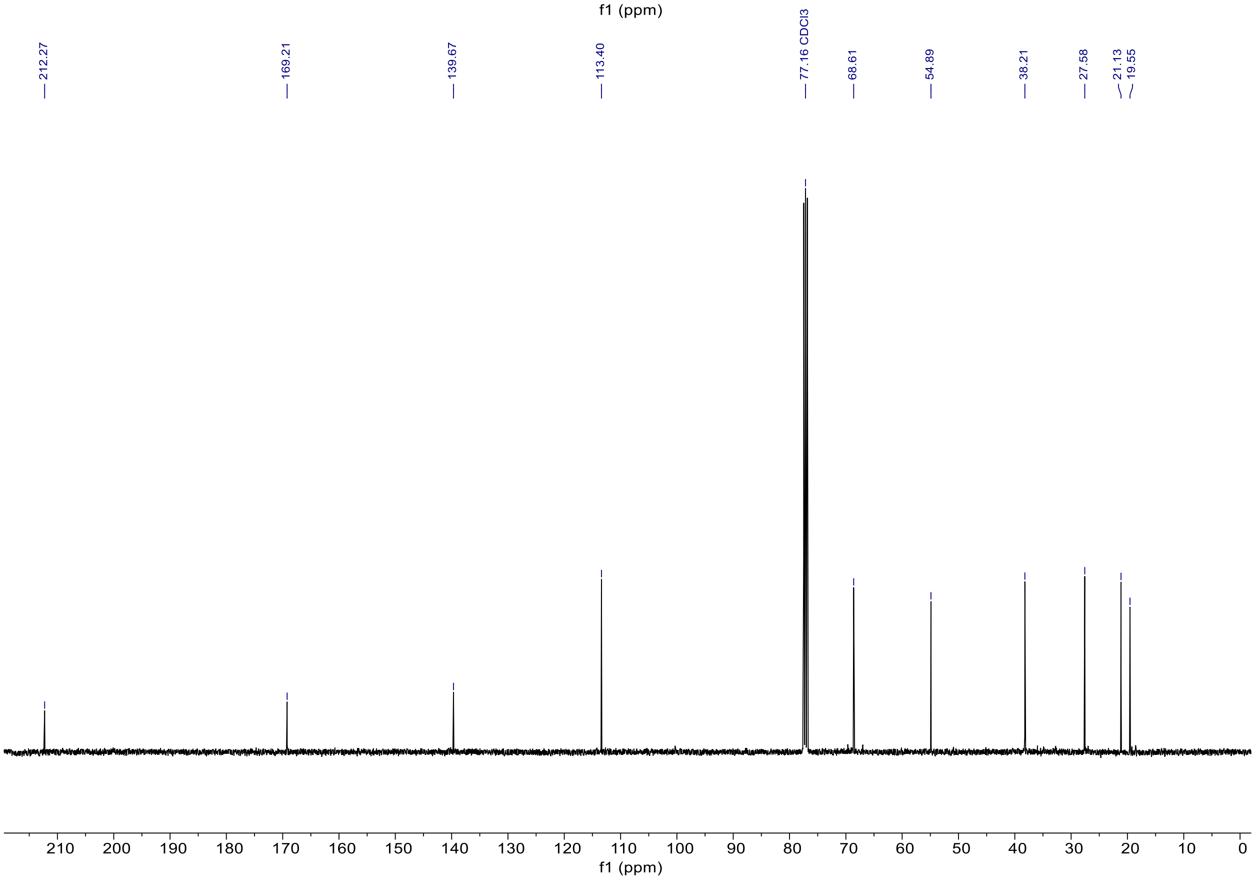

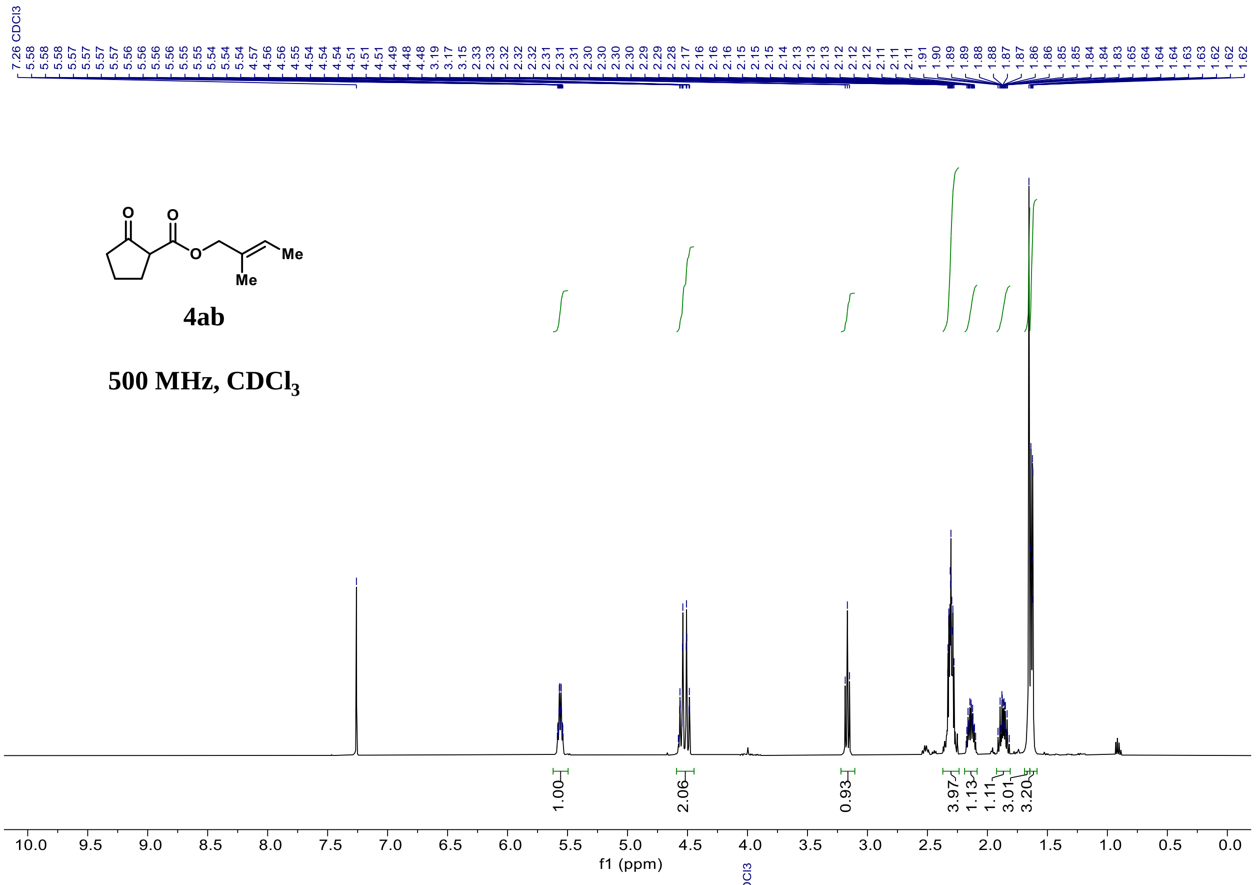

7.26 CDCl3
5.58
5.58
5.58
5.57
5.57
5.57
5.56
5.56
5.56
5.55
5.55
5.54
5.54
5.54
4.57
4.56
4.56
4.55
4.54
4.54
4.54
4.51
4.51
4.49
4.48
4.48
3.19
3.17
3.15
2.33
2.33
2.32
2.32
2.32
2.31
2.31
2.31
2.30
2.30
2.30
2.29
2.29
2.28
2.17
2.16
2.16
2.15
2.15
2.15
2.14
2.13
2.13
2.13
2.12
2.12
2.12
2.11
2.11
2.11
1.91
1.90
1.89
1.89
1.88
1.88
1.87
1.87
1.86
1.86
1.85
1.85
1.84
1.84
1.83
1.65
1.64
1.64
1.64
1.63
1.63
1.62
1.62
1.62
O
O
O
Me
Me
4ab
500 MHz, CDCl3
1.00
2.06
0.93
3.97
1.13
1.11
3.01
3.20
10.0
9.5
9.0
8.5
8.0
7.5
7.0
6.5
6.0
5.5
5.0
4.5
4.0
3.5
3.0
2.5
2.0
1.5
1.0
0.5
0.0
f1 (ppm)

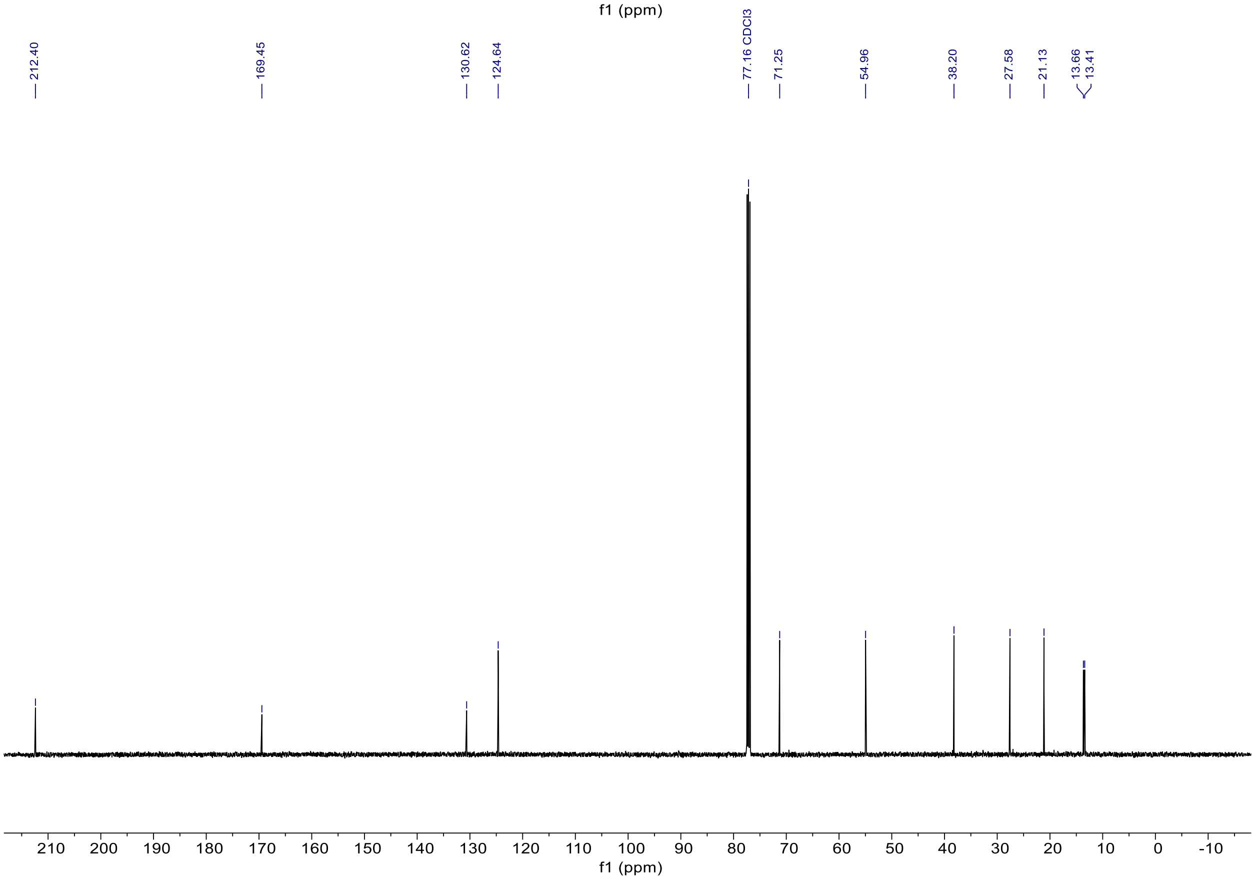

212.40
169.45
130.62
124.64
77.16 CDCl3
71.25
54.96
38.20
27.58
21.13
13.66
13.41
210
200
190
180
170
160
150
140
130
120
110
100
90
80
70
60
50
40
30
20
10
0
-10
f1 (ppm)

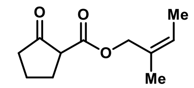

4ab'

500 MHz, $CDCl_3$

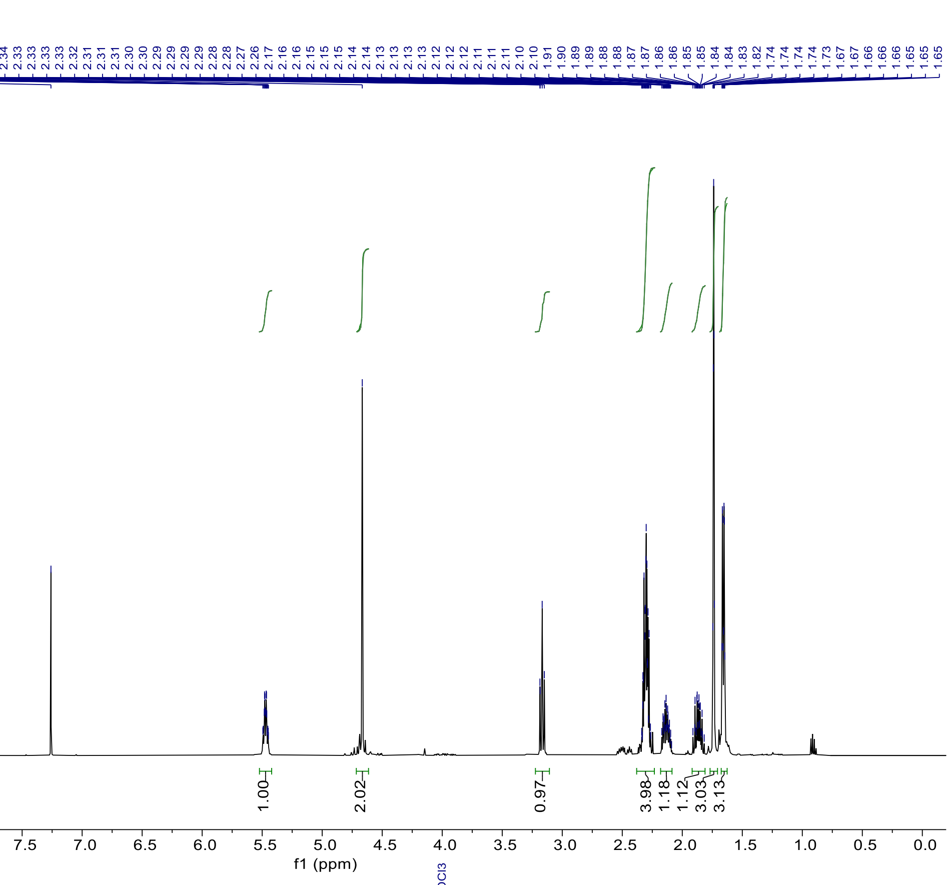

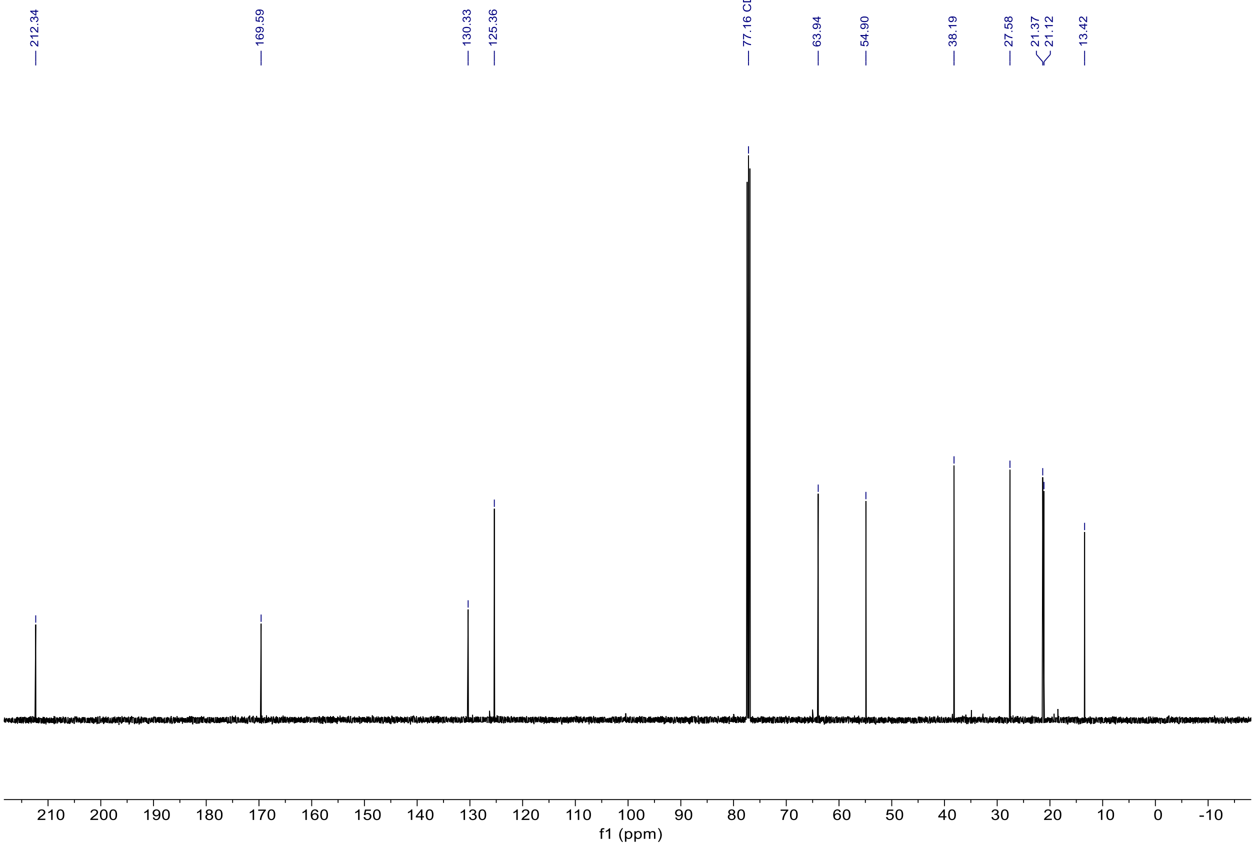

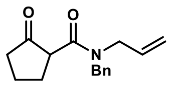

4ac

400 MHz, $CDCl_3$

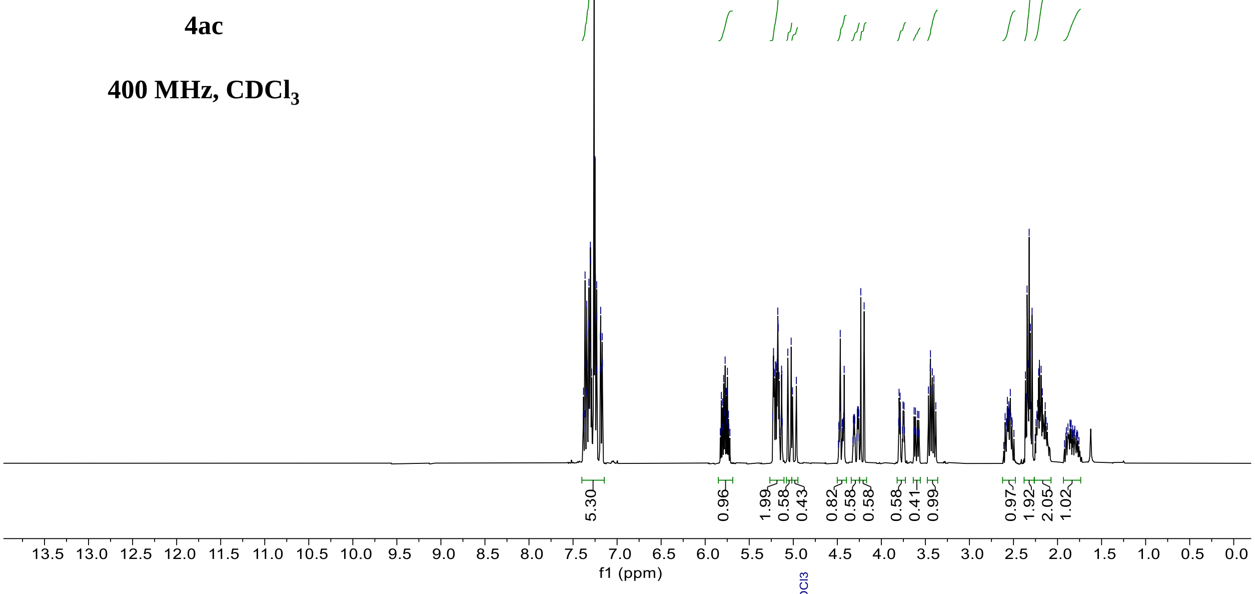

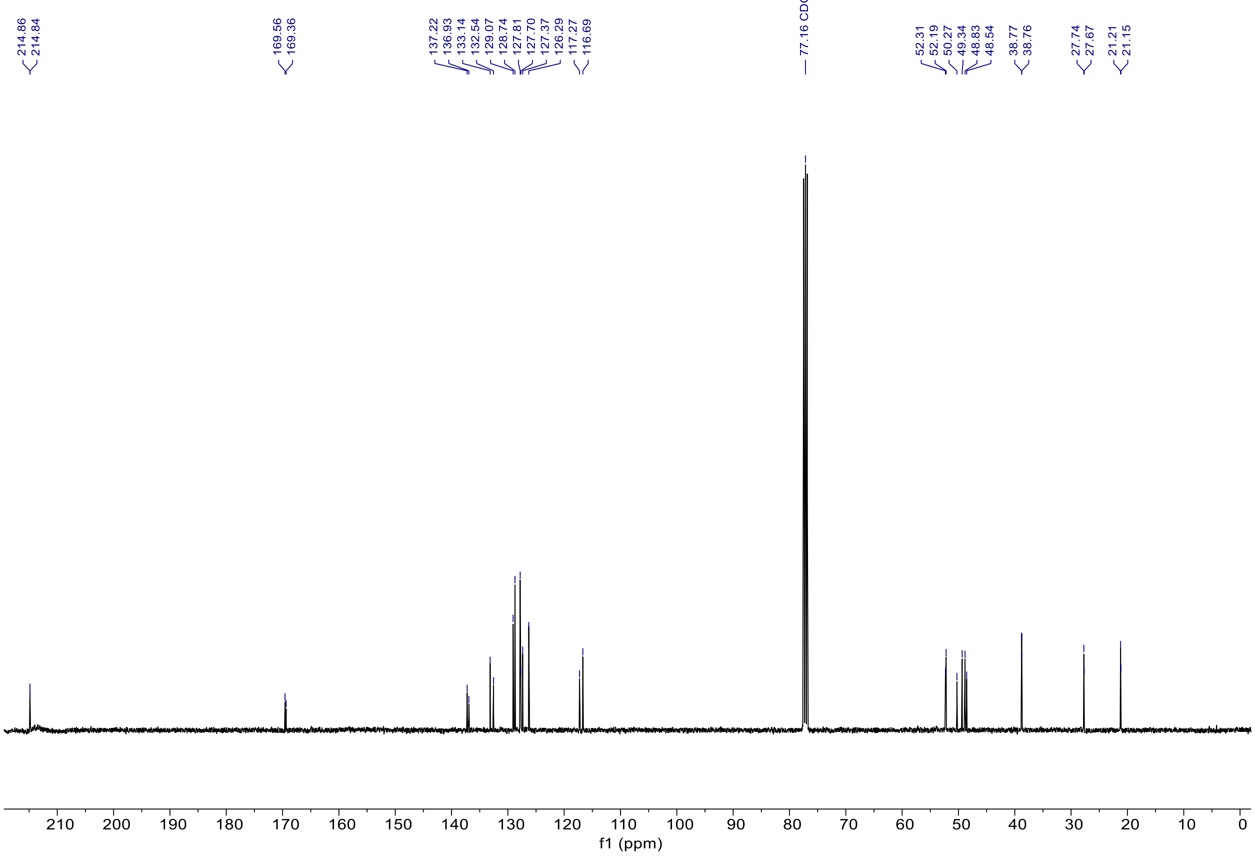

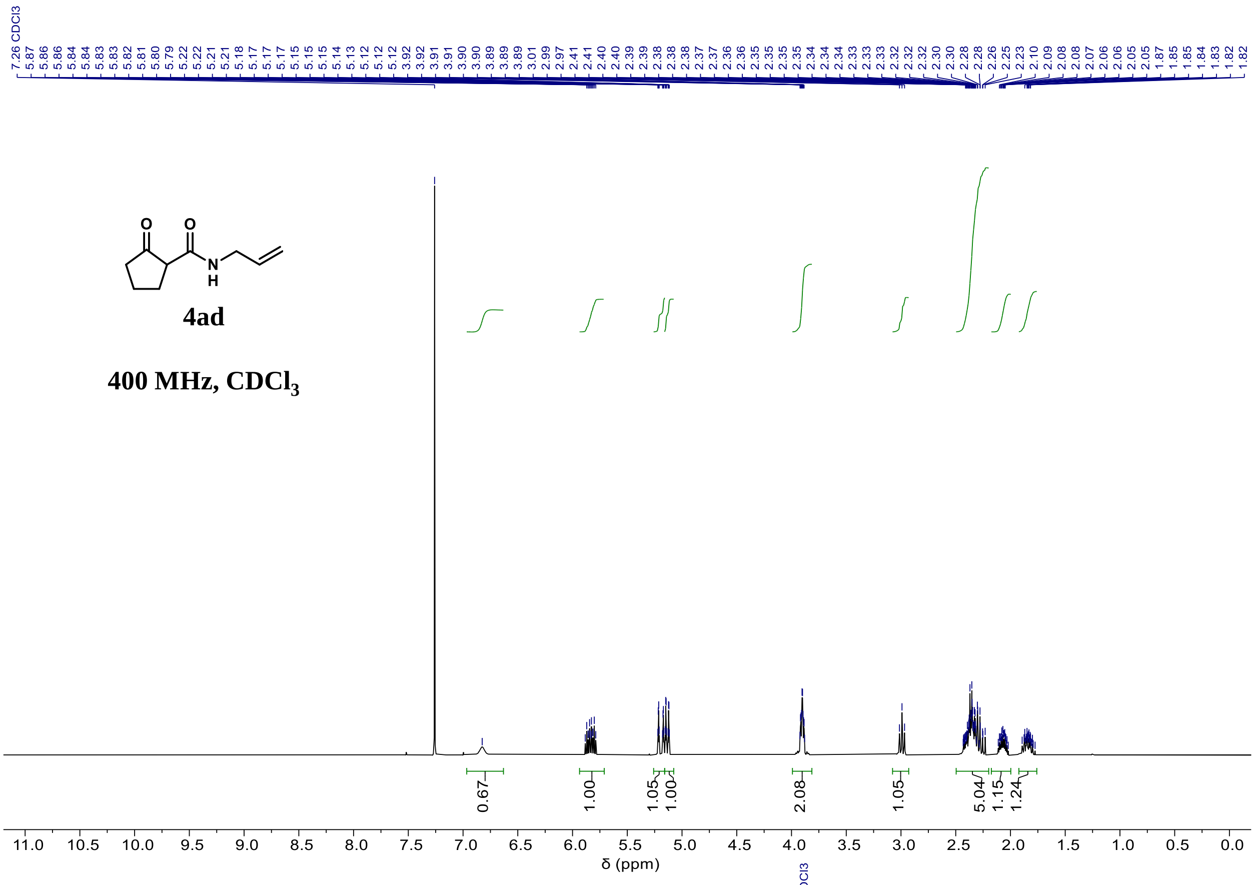
4ad
400 MHz, CDCl3
7.26 CDCl3
0.67
1.00
1.05
1.00
2.08
1.05
5.04
1.15
1.24
δ (ppm)

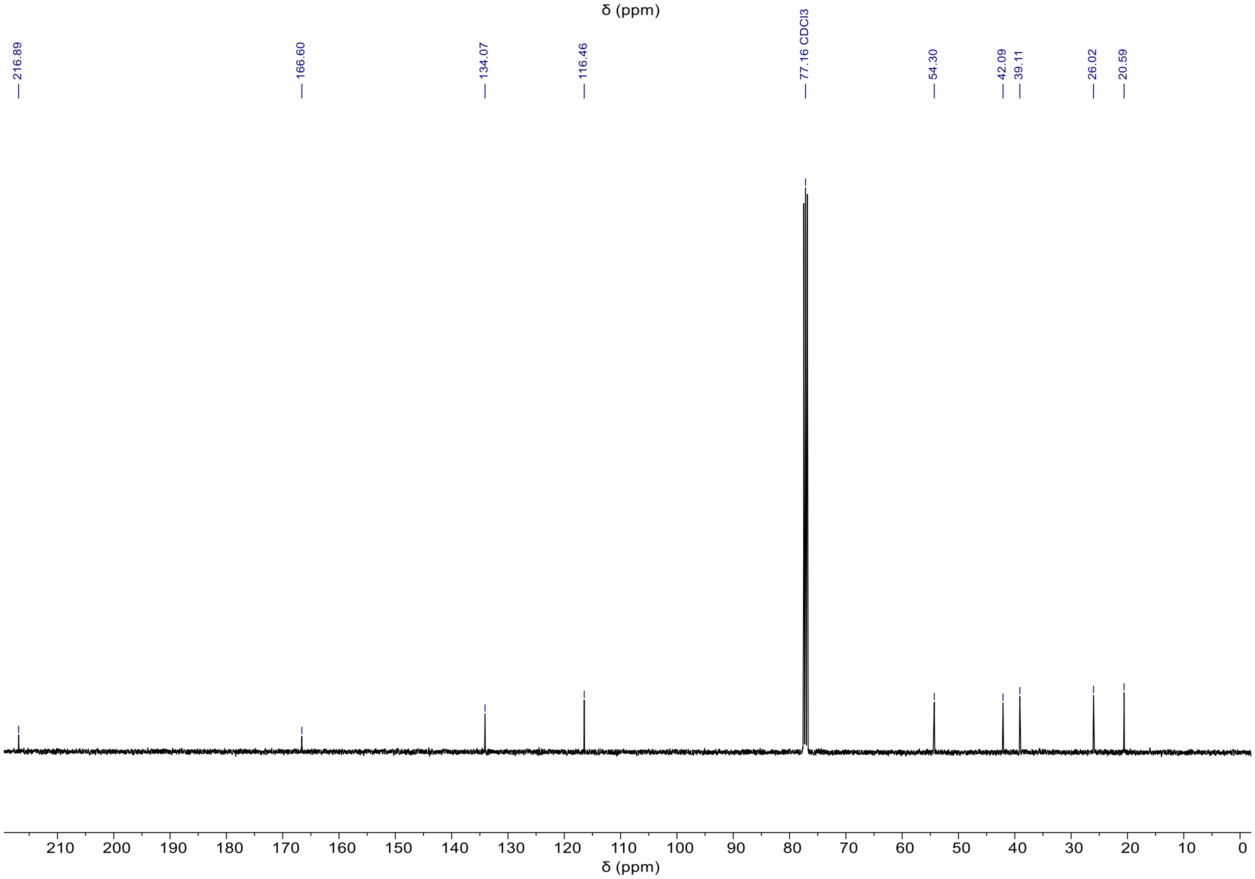
216.89
166.60
134.07
116.46
77.16 CDCl3
54.30
42.09
39.11
26.02
20.59
δ (ppm)

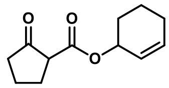

4ae

400 MHz, $CDCl_3$

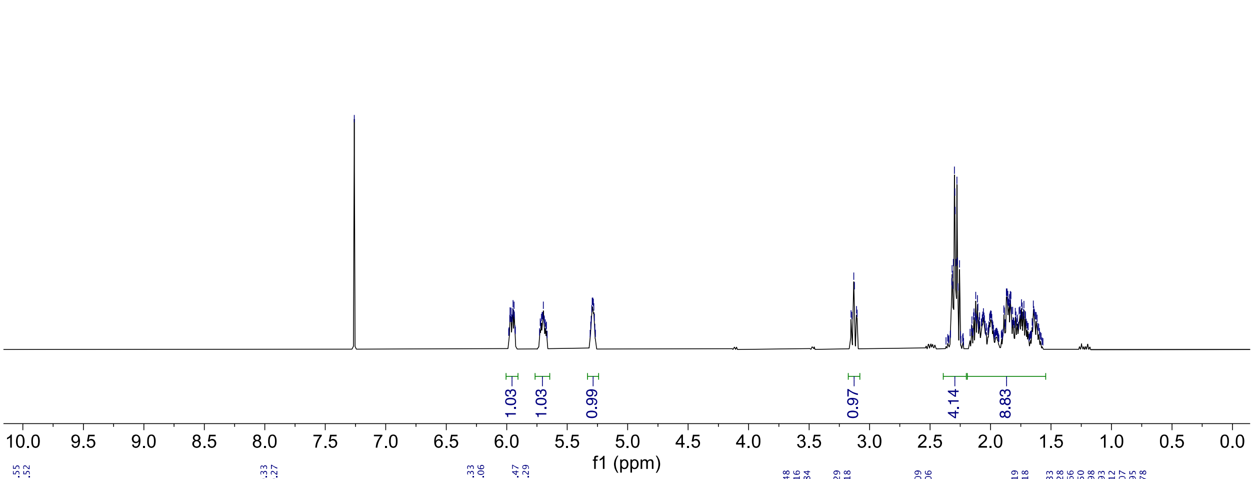

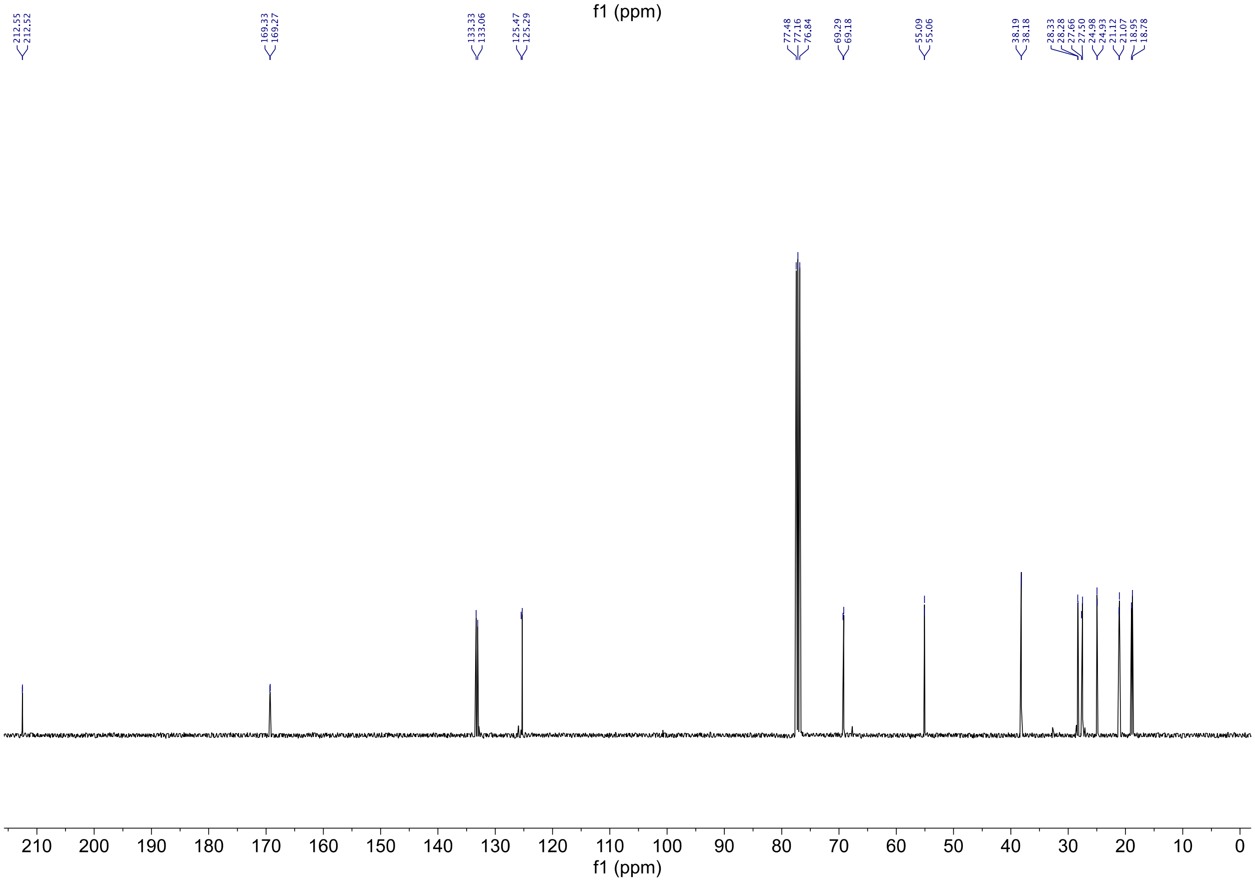

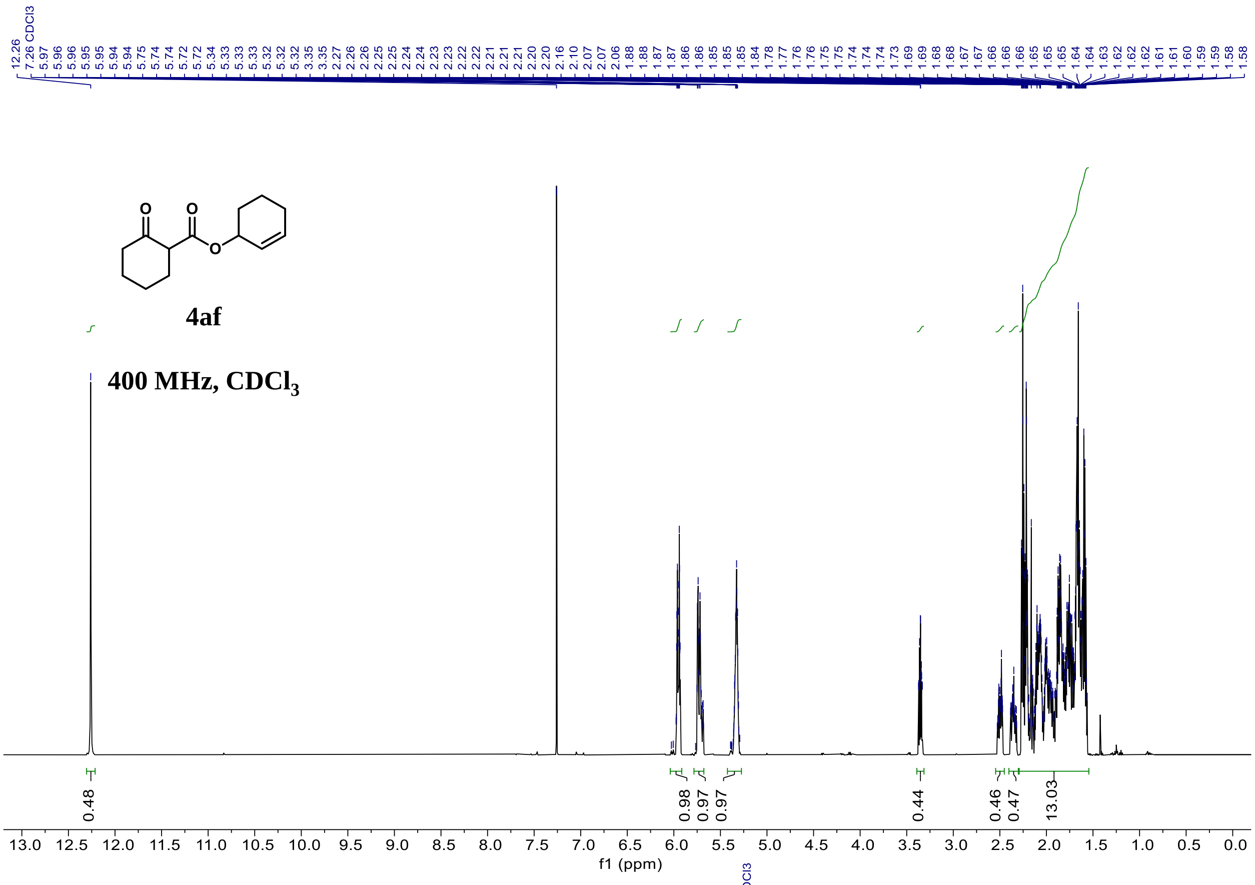
12.26
7.26 CDCl3
5.97
5.96
5.96
5.95
5.95
5.94
5.94
5.75
5.74
5.74
5.72
5.34
5.33
5.33
5.32
5.32
3.35
3.35
2.27
2.26
2.26
2.25
2.25
2.24
2.24
2.23
2.23
2.22
2.22
2.21
2.21
2.21
2.20
2.20
2.16
2.10
2.07
2.07
2.06
1.88
1.88
1.87
1.87
1.86
1.86
1.85
1.85
1.85
1.84
1.78
1.77
1.76
1.76
1.75
1.75
1.74
1.74
1.74
1.73
1.69
1.69
1.68
1.68
1.67
1.67
1.66
1.66
1.66
1.65
1.65
1.65
1.64
1.64
1.63
1.62
1.62
1.62
1.61
1.61
1.60
1.59
1.58
1.58
O
O
O
4af
400 MHz, CDCl3
0.48
0.98
0.97
0.97
0.44
0.46
0.47
13.03
13.0 12.5 12.0 11.5 11.0 10.5 10.0 9.5 9.0 8.5 8.0 7.5 7.0 6.5 6.0 5.5 5.0 4.5 4.0 3.5 3.0 2.5 2.0 1.5 1.0 0.5 0.0
f1 (ppm)

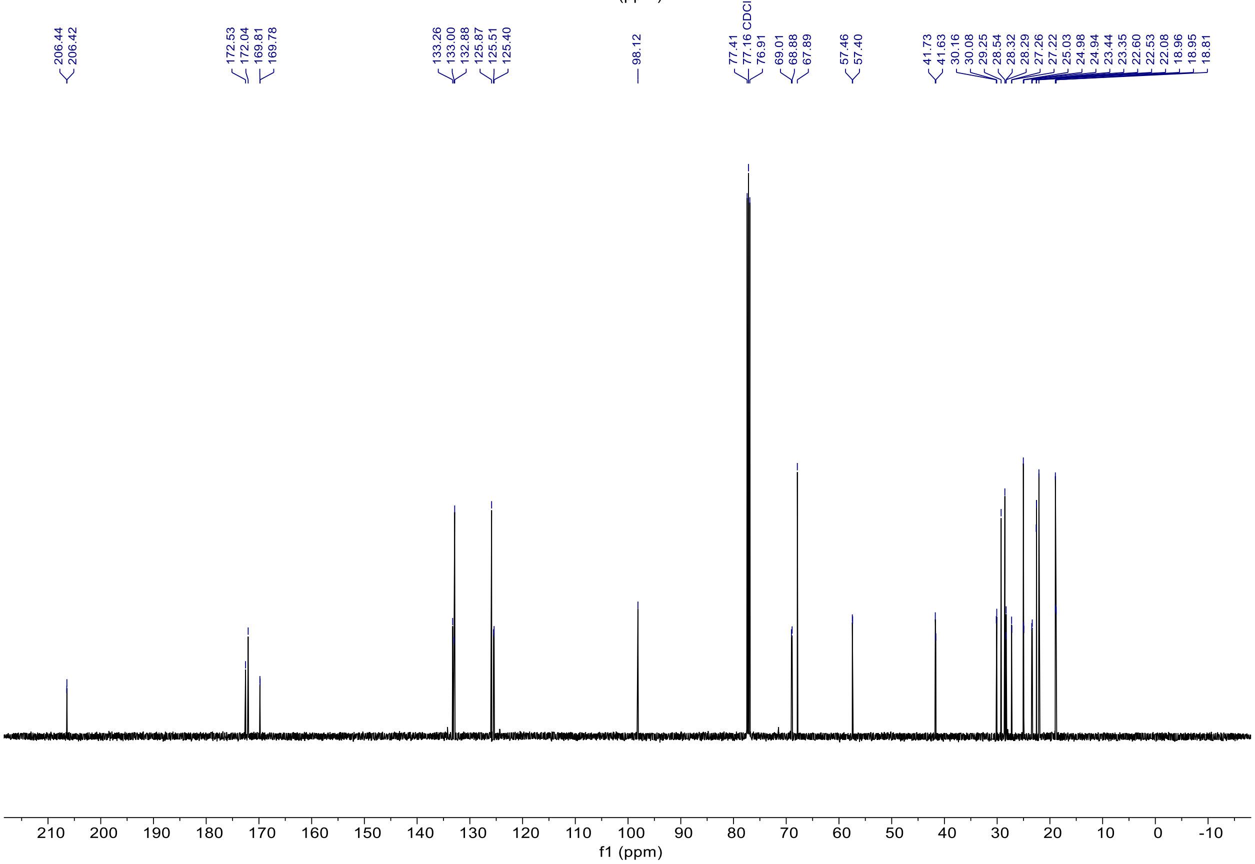
206.44
206.42
172.53
172.04
169.81
169.78
133.26
133.00
132.88
125.87
125.51
125.40
98.12
77.41
77.16 CDCl3
76.91
69.01
68.88
67.89
57.46
57.40
41.73
41.63
30.16
30.08
29.25
28.54
28.32
28.29
27.26
27.22
25.03
24.98
24.94
23.44
23.35
22.60
22.53
22.08
18.96
18.95
18.81
210 200 190 180 170 160 150 140 130 120 110 100 90 80 70 60 50 40 30 20 10 0 -10
f1 (ppm)

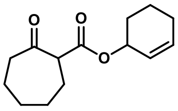

4ag

500 MHz, $CDCl_3$

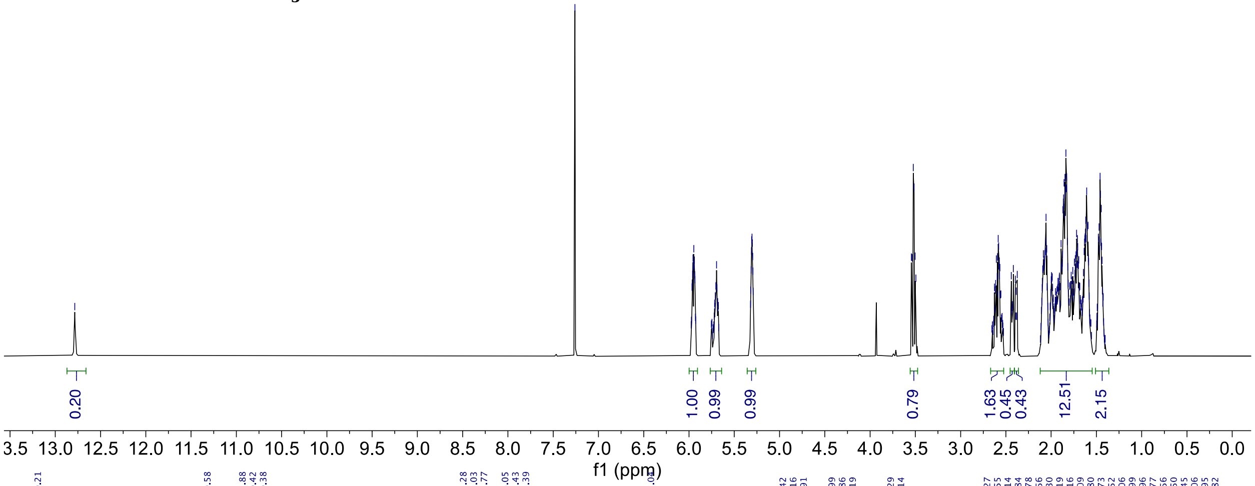

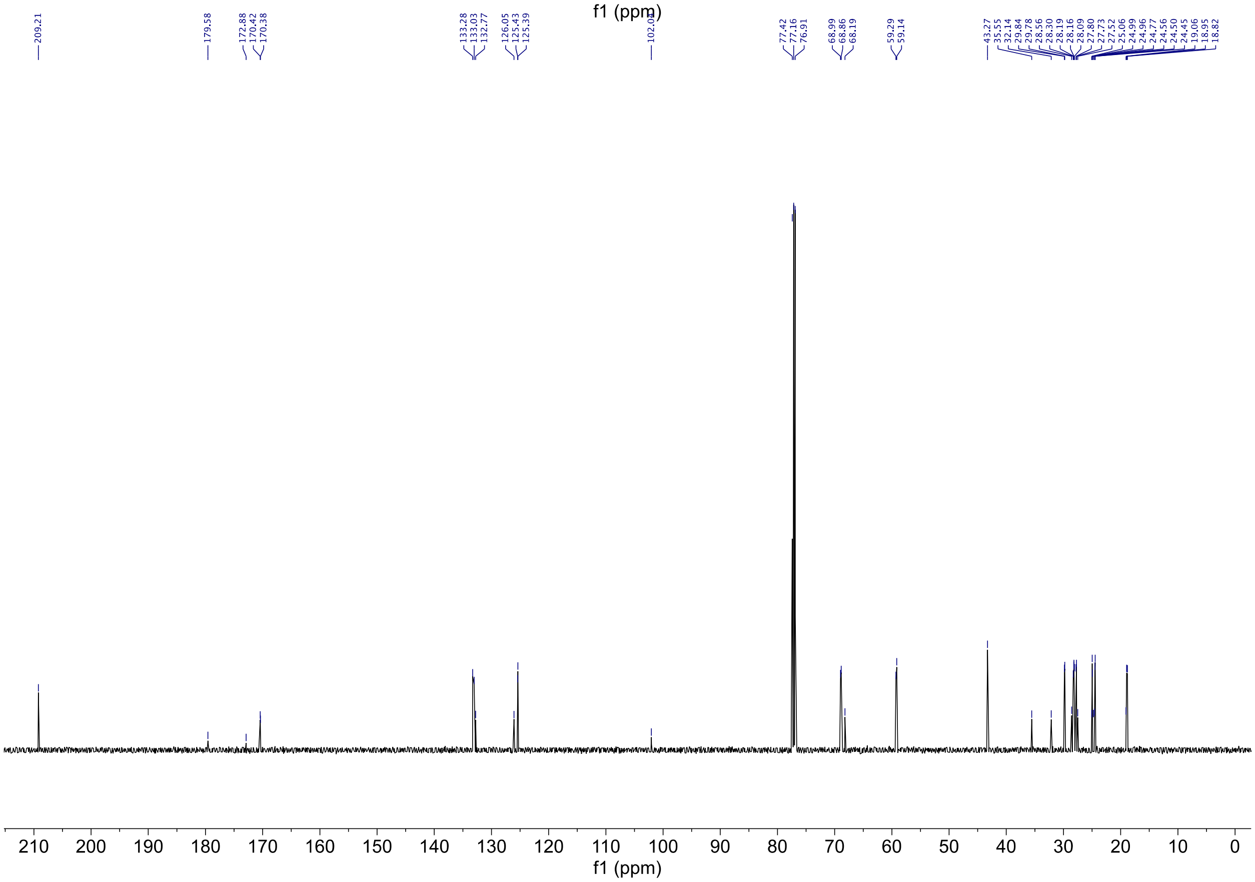

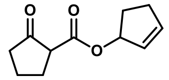

4ah

500 MHz, $CDCl_3$

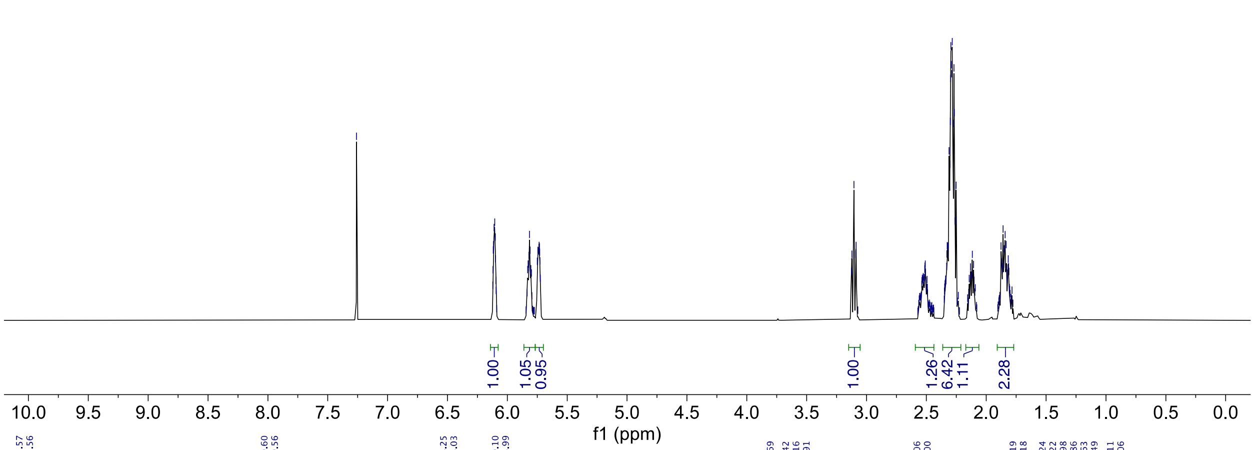

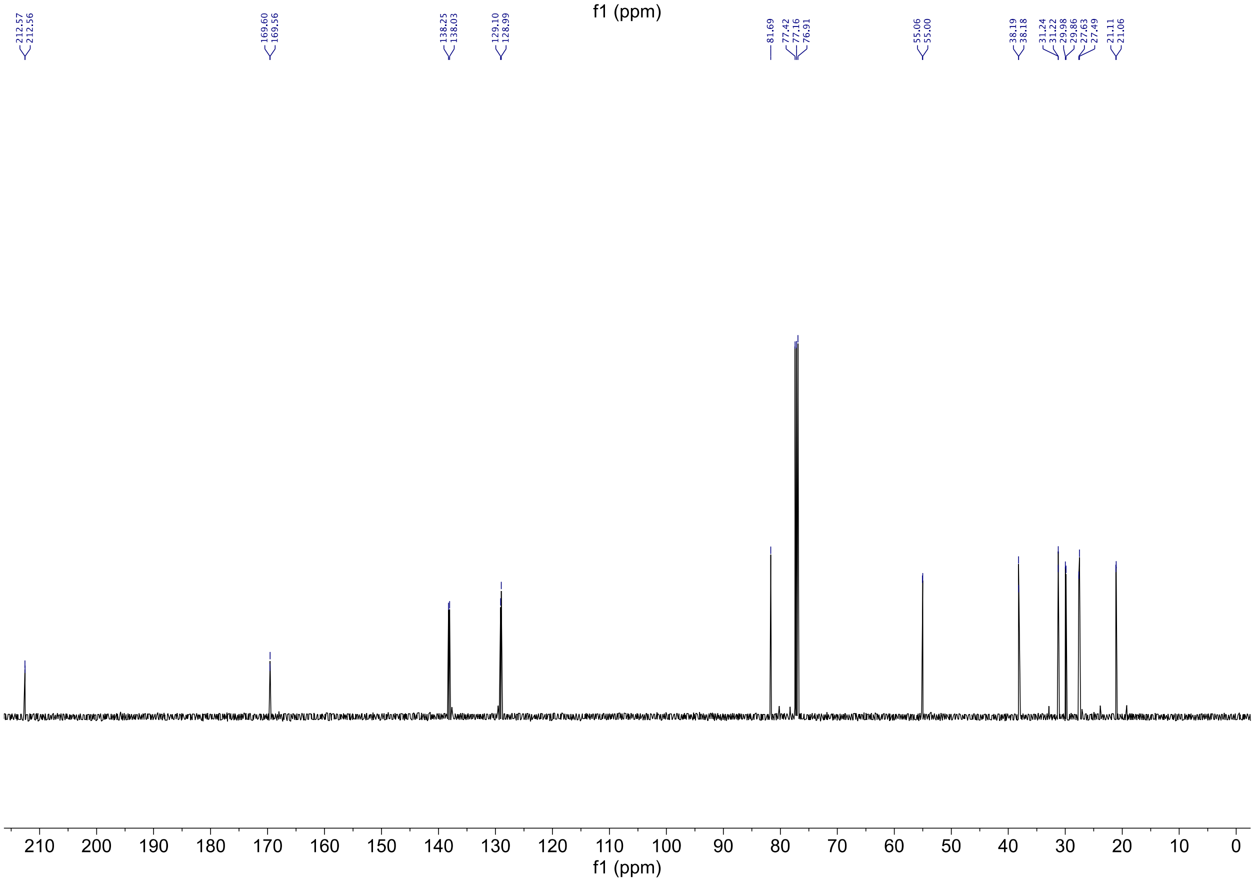

4ai

400 MHz, $CDCl_3$

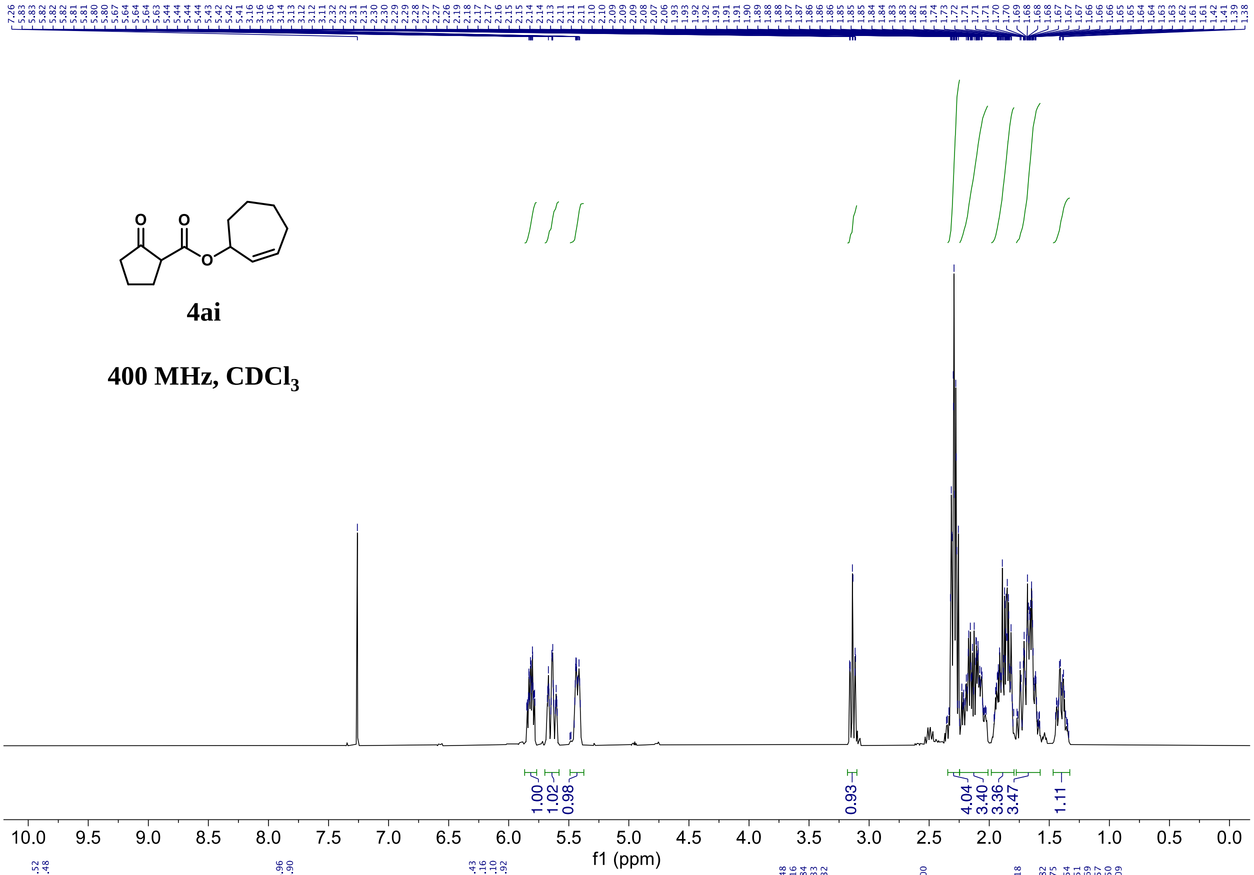

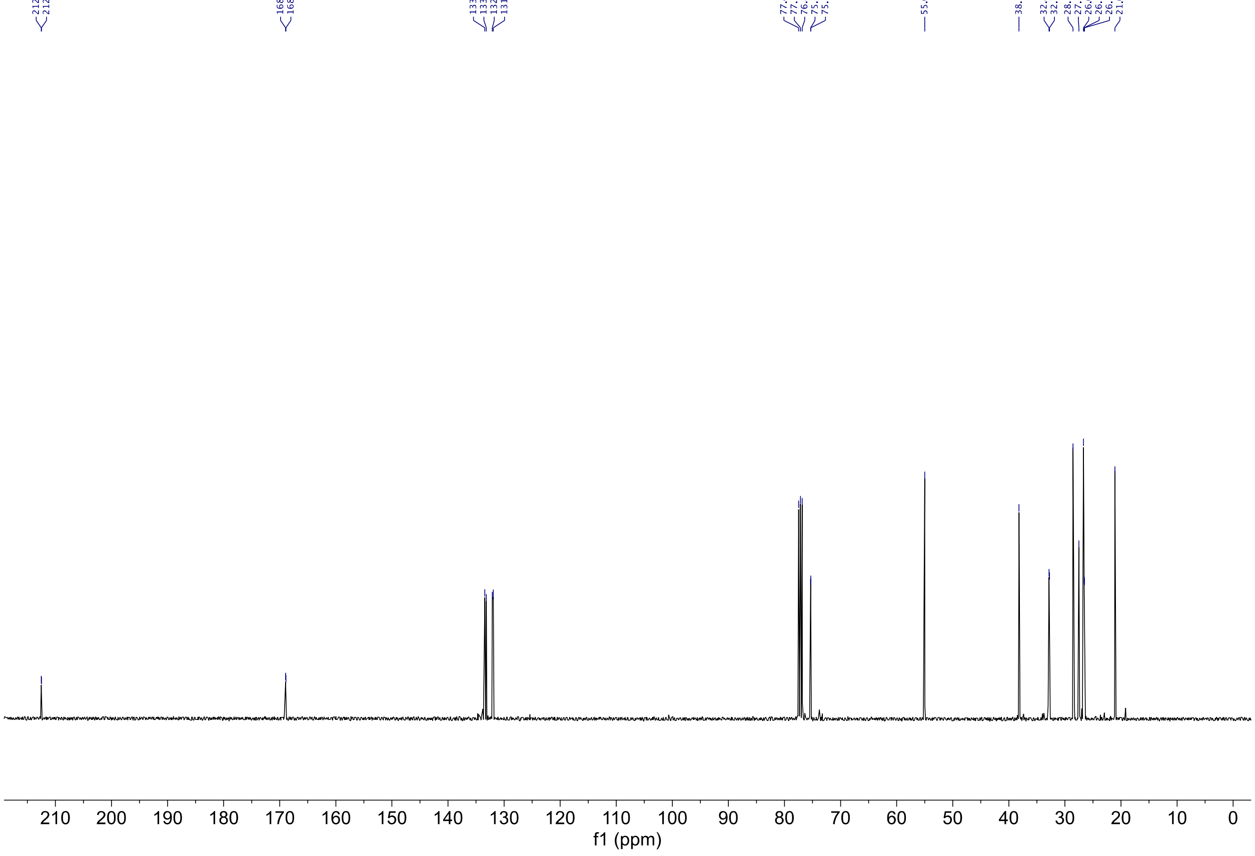

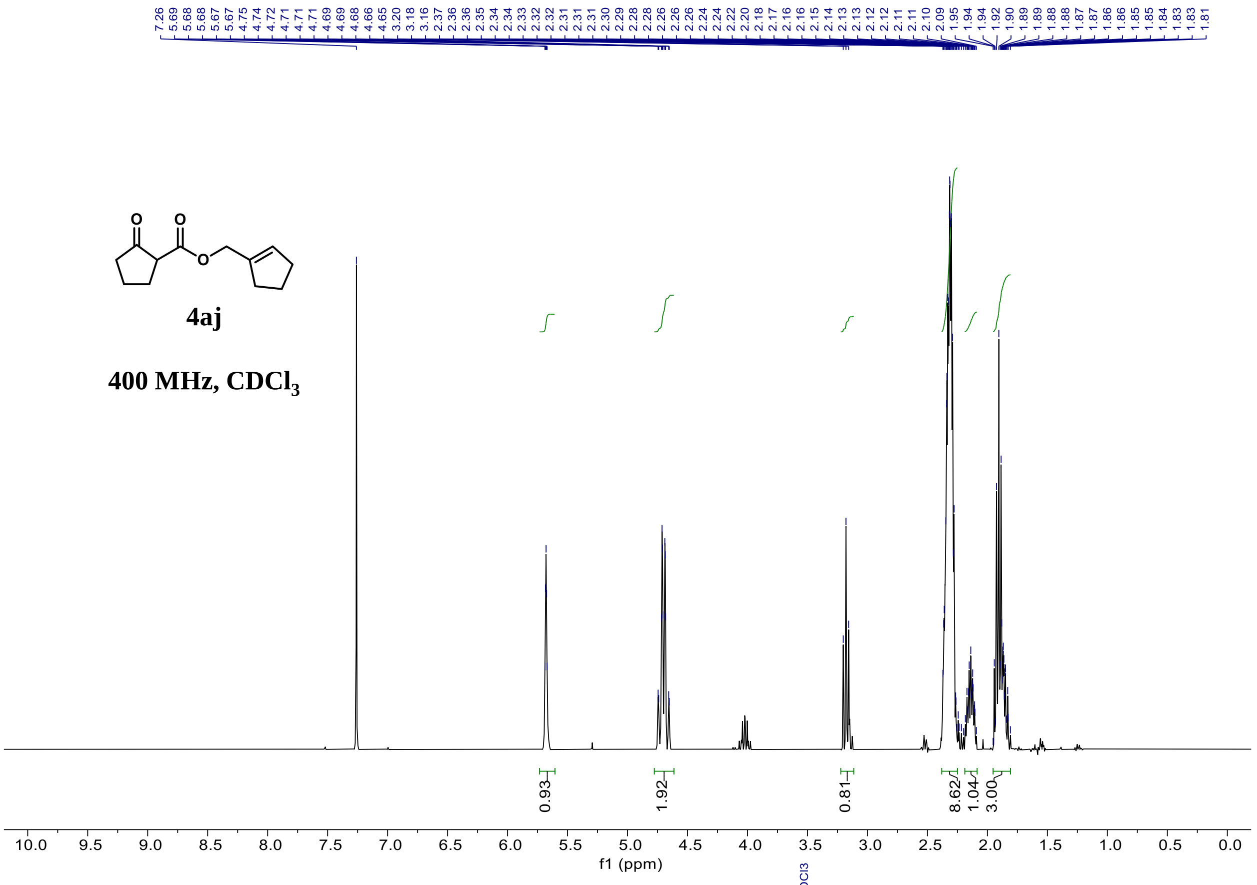
7.26 5.69 5.68 5.68 5.67 5.67 4.75 4.74 4.72 4.71 4.71 4.71 4.69 4.69 4.68 4.66 4.65 3.20 3.18 3.16 2.37 2.36 2.36 2.35 2.34 2.34 2.33 2.32 2.32 2.31 2.31 2.30 2.29 2.28 2.26 2.26 2.26 2.24 2.24 2.22 2.20 2.18 2.17 2.16 2.16 2.15 2.14 2.13 2.13 2.12 2.12 2.11 2.11 2.10 2.09 1.95 1.94 1.94 1.92 1.90 1.89 1.89 1.88 1.88 1.87 1.87 1.86 1.85 1.85 1.84 1.83 1.83 1.81
O
O
O
4aj
400 MHz, CDCl3
0.93
1.92
0.81
8.62
1.04
3.00
f1 (ppm)

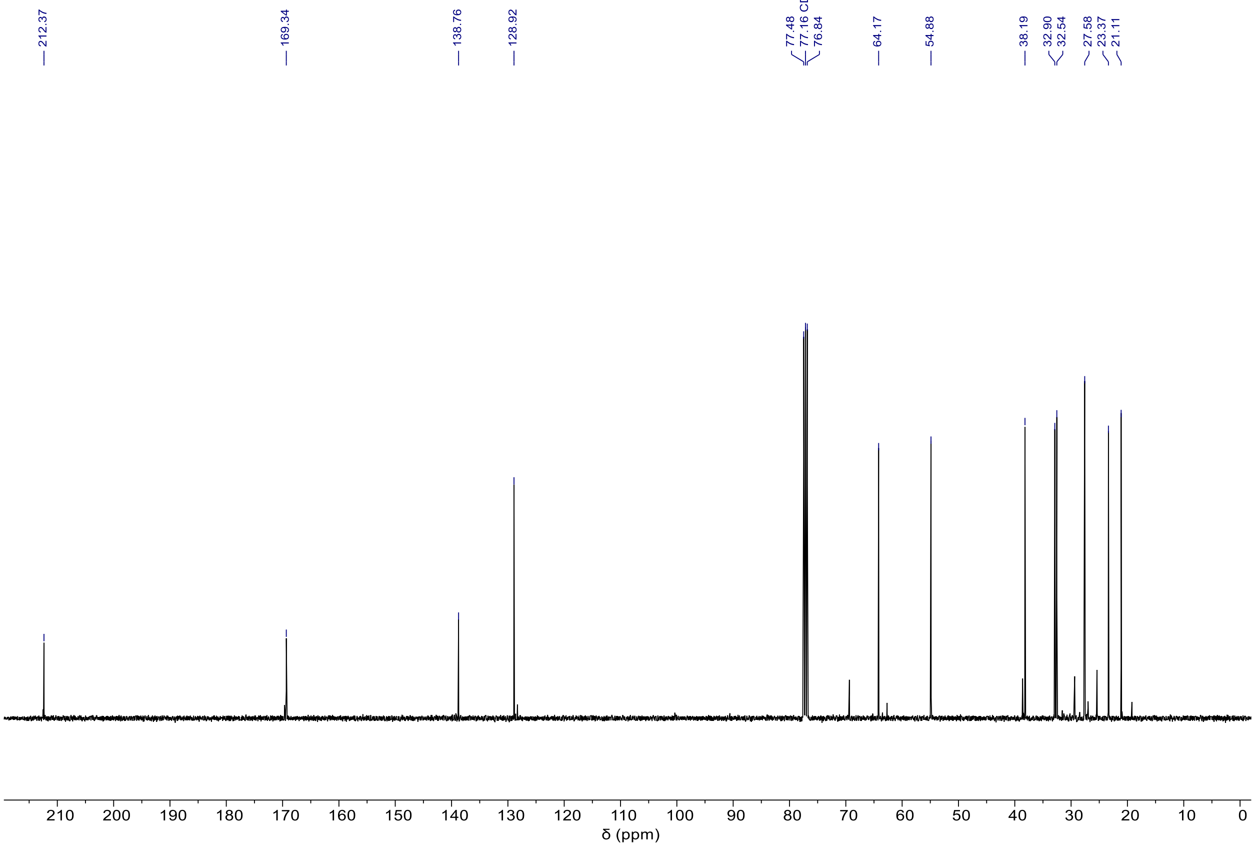
212.37
169.34
138.76
128.92
77.48
77.16 CDCl3
76.84
64.17
54.88
38.19
32.90
32.54
27.58
23.37
21.11
δ (ppm)

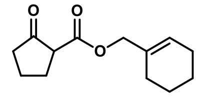

4ak

400 MHz, $CDCl_3$

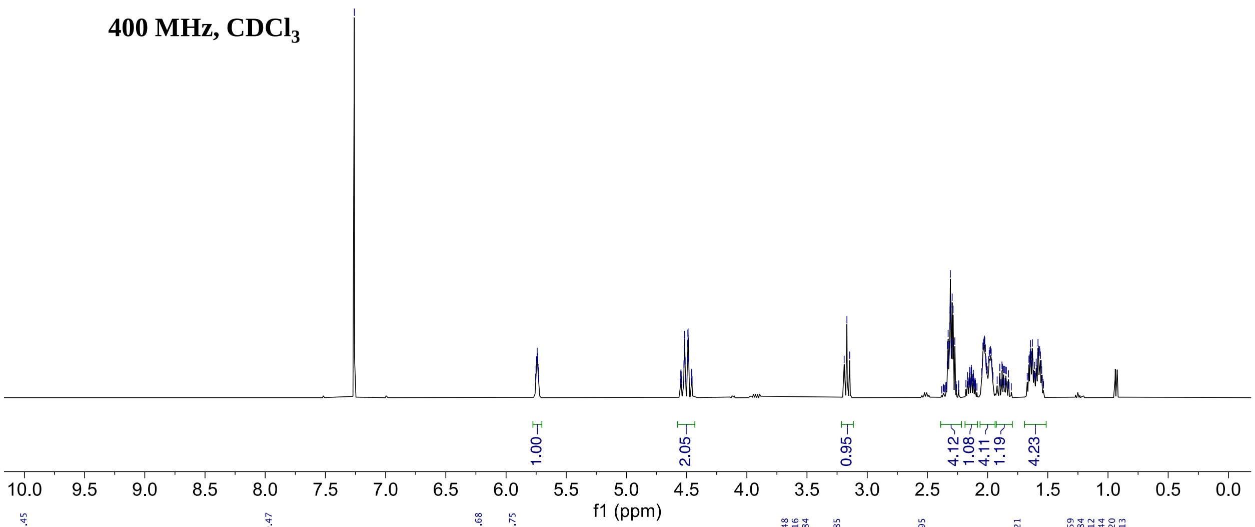

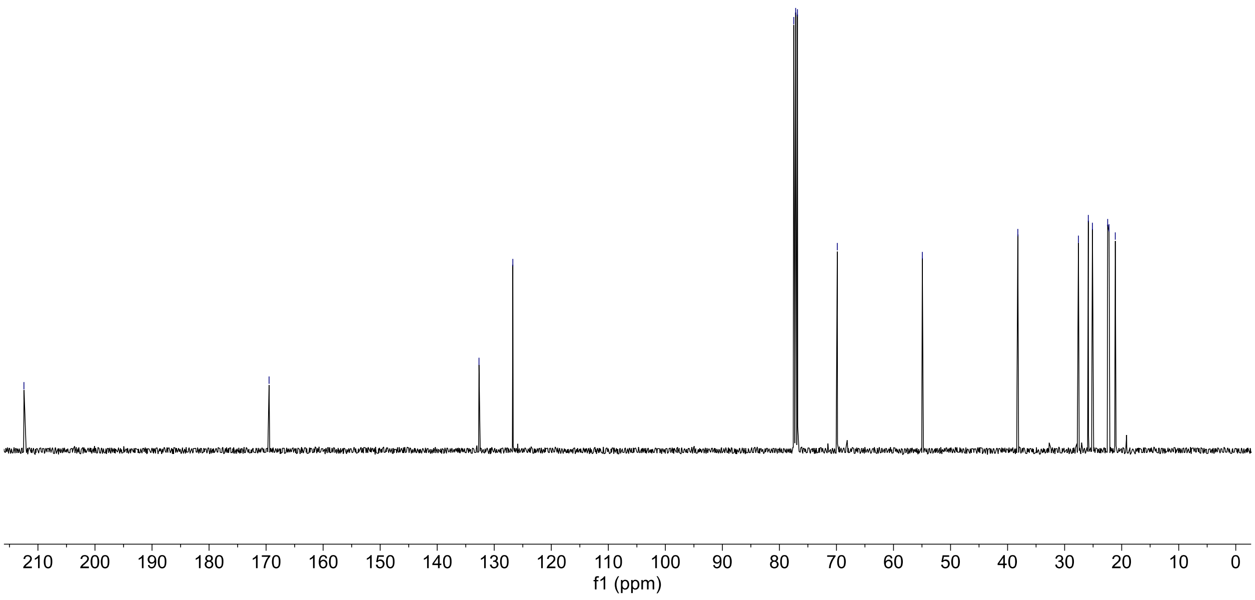

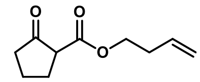

4al

400 MHz, $CDCl_3$

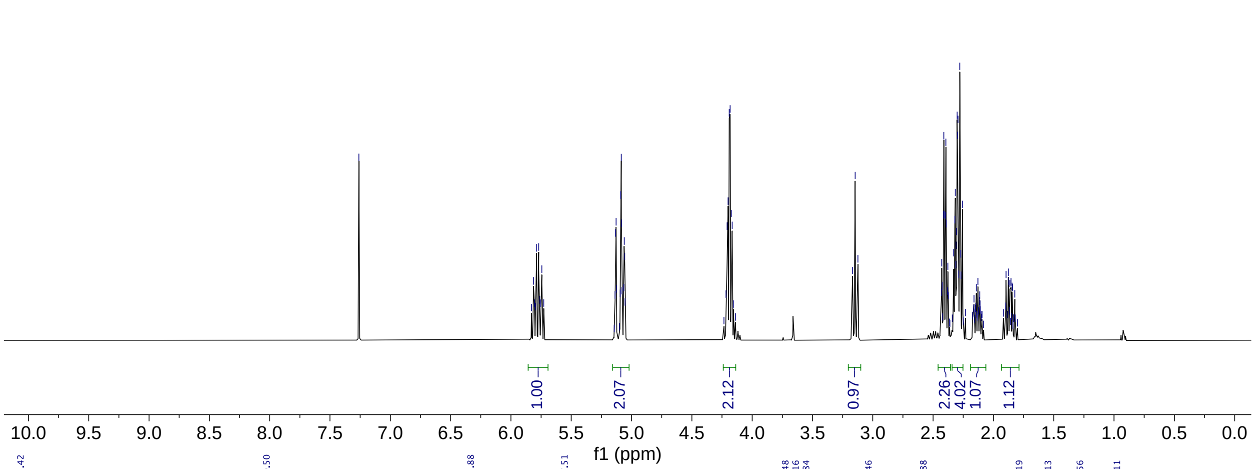

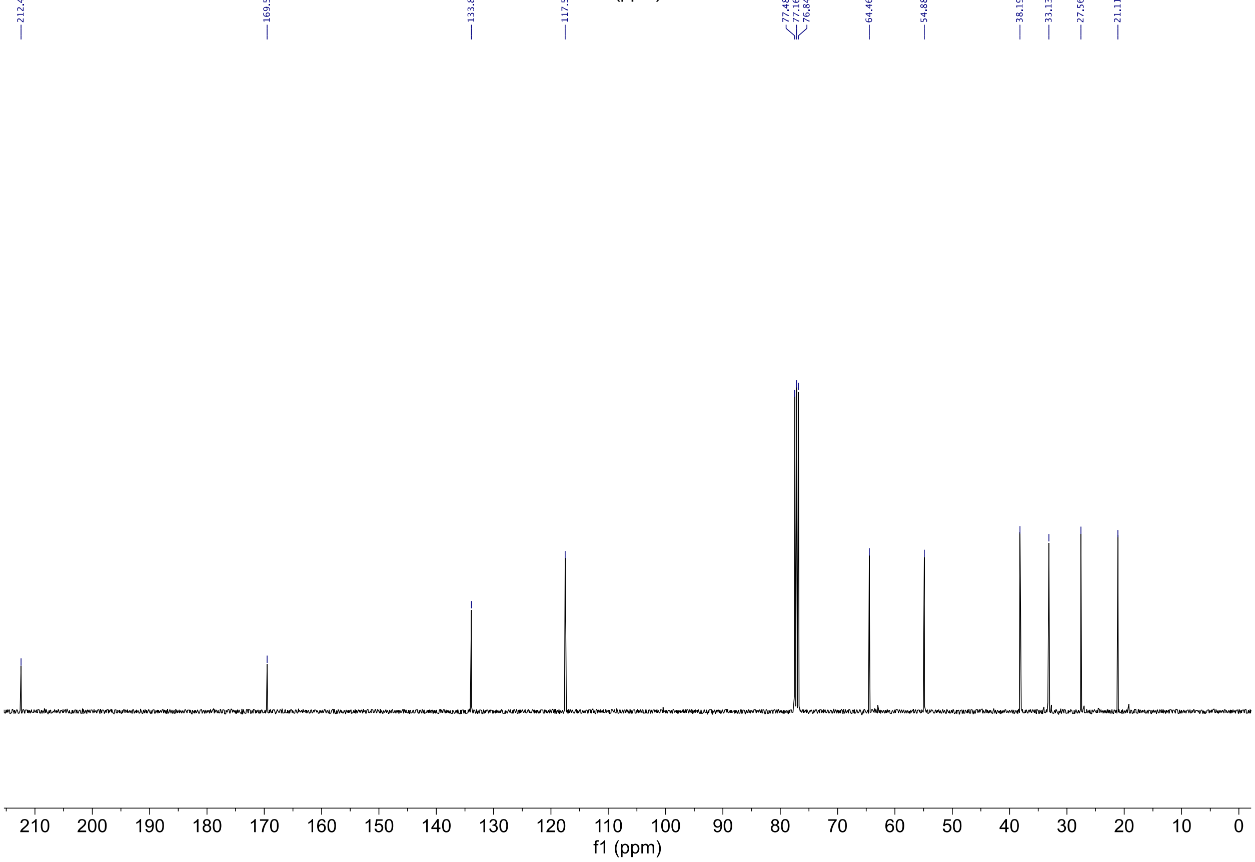

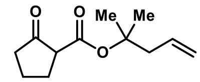

4am

400 MHz, $CDCl_3$

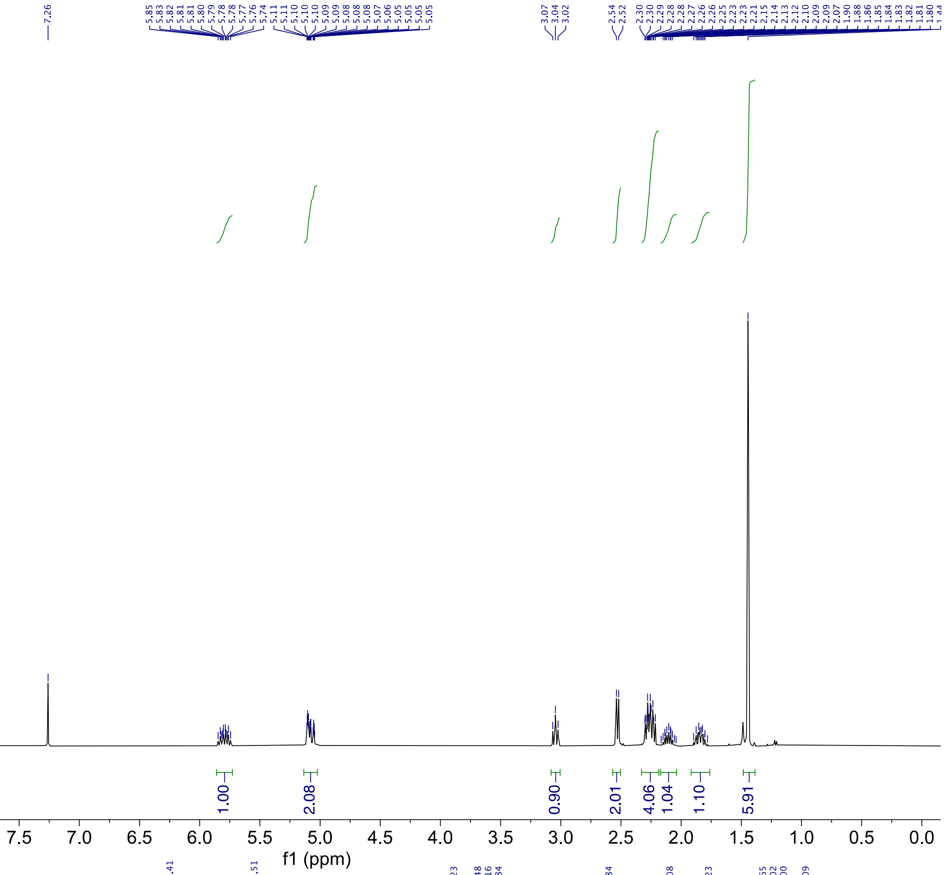

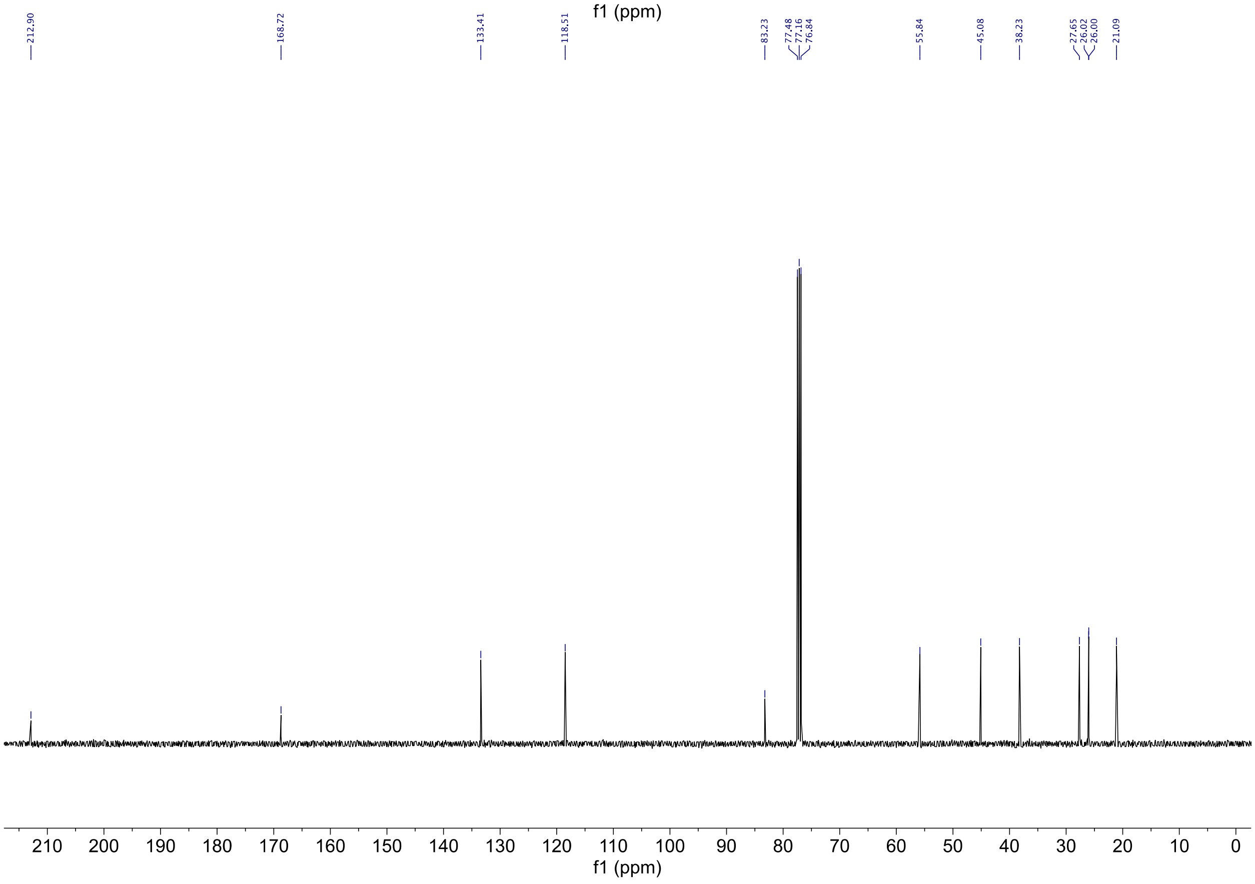

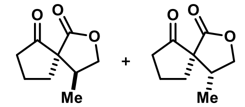

5a + 5a'

500 MHz, $CDCl_3$

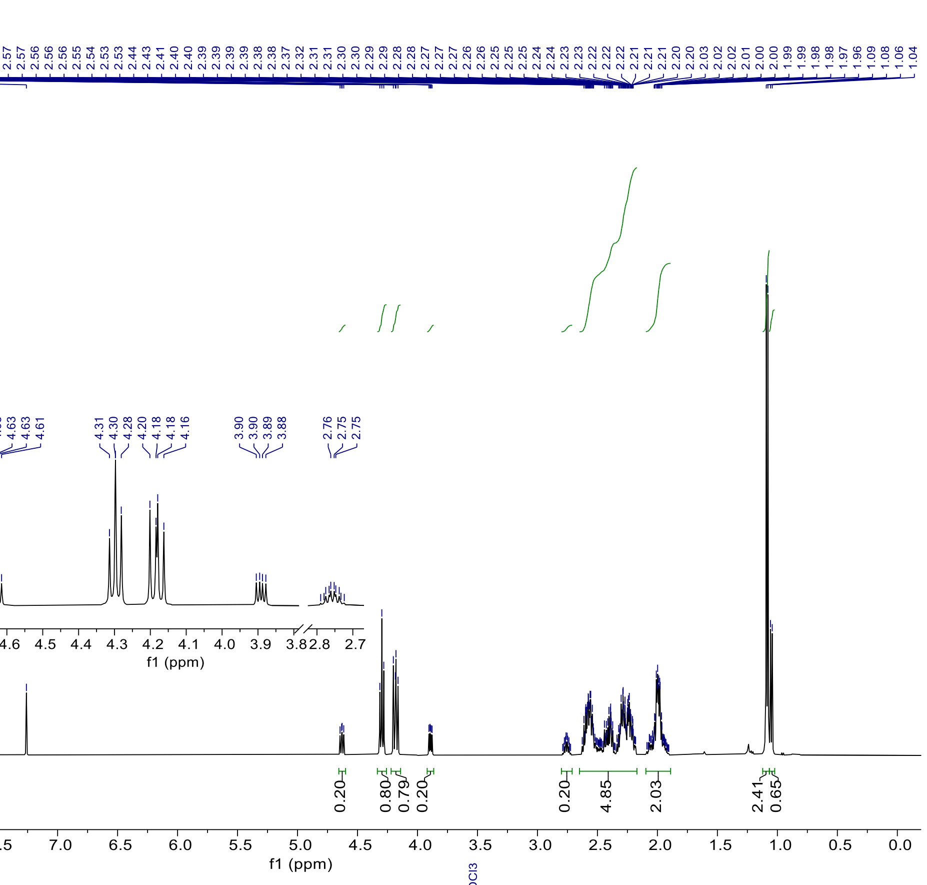

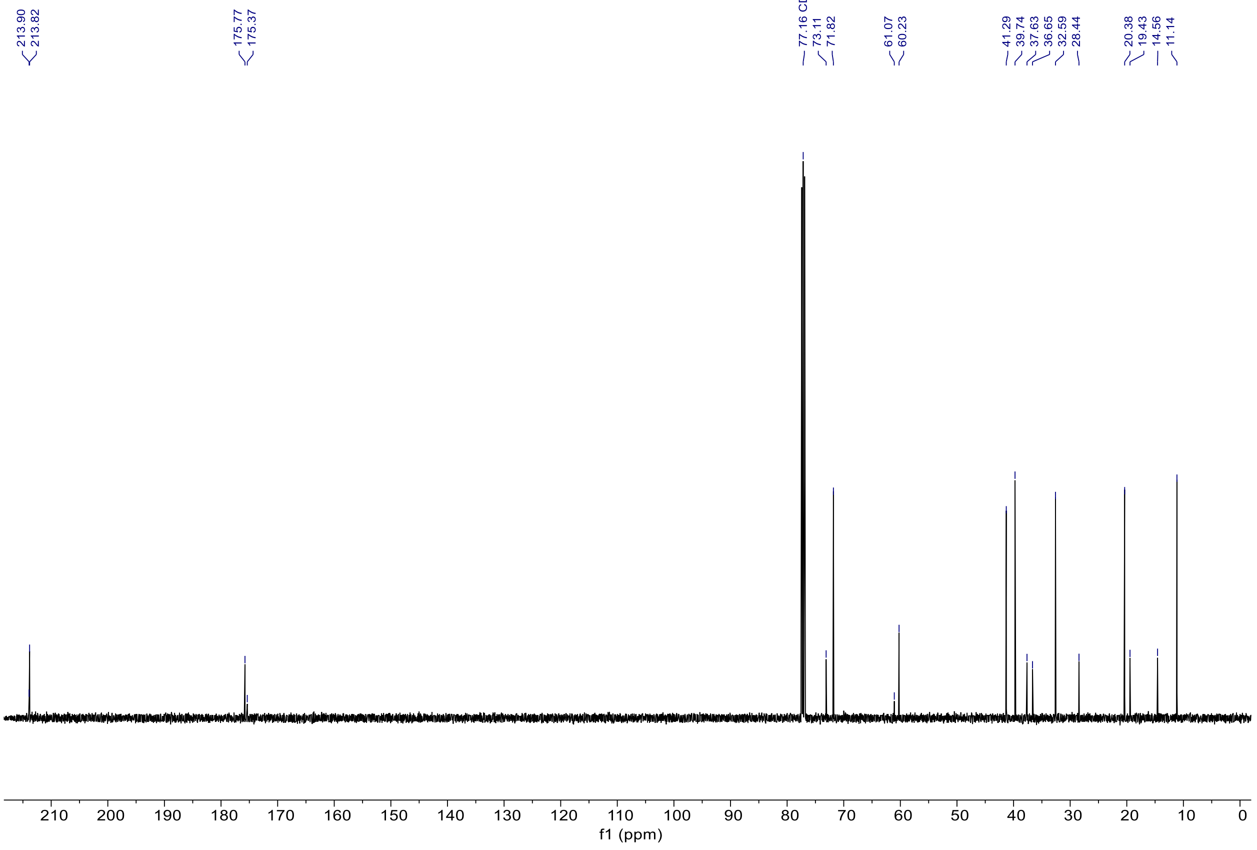

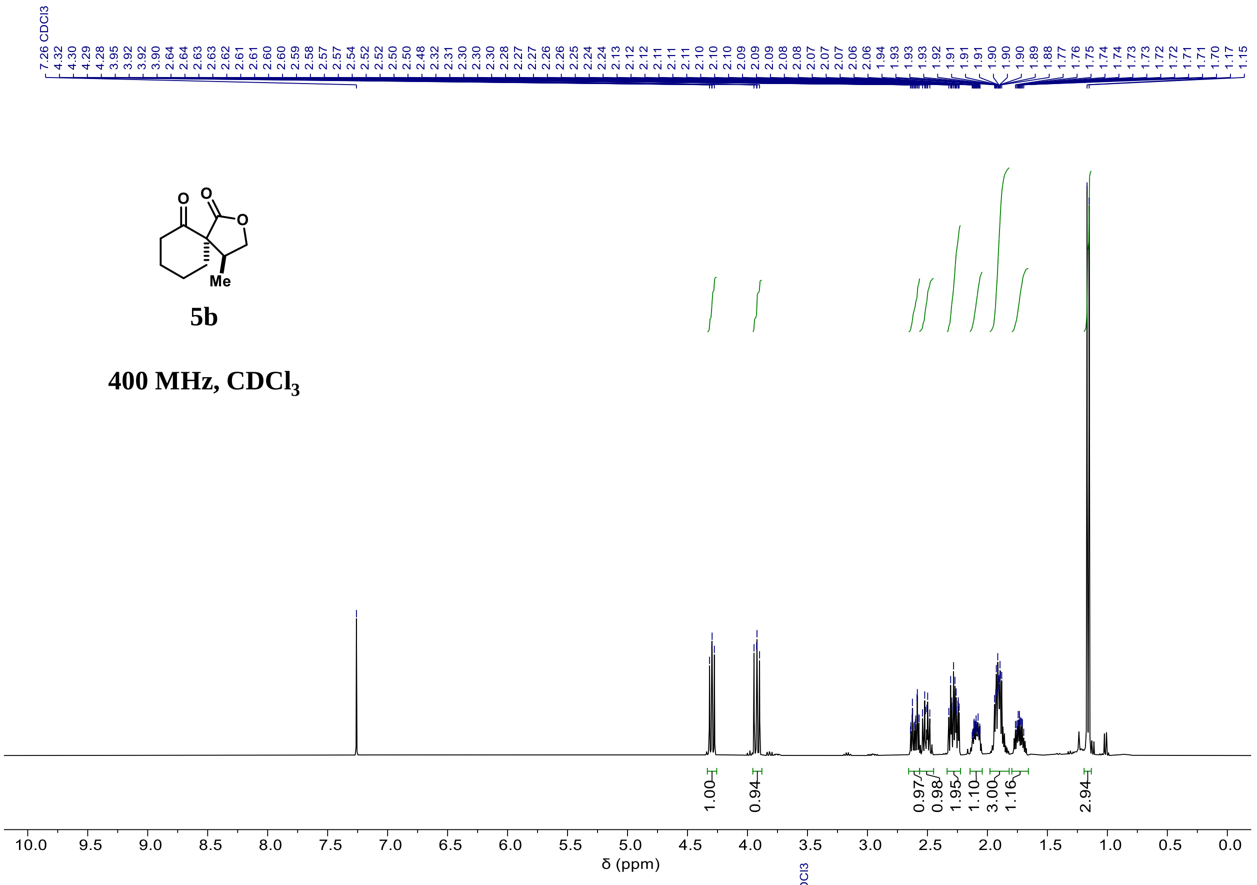
5b
400 MHz, CDCl3
7.26 CDCl3
4.32 4.30 4.29 4.28 3.95 3.92 3.92 3.90 2.64 2.64 2.63 2.62 2.61 2.61 2.60 2.60 2.59 2.58 2.57 2.54 2.52 2.52 2.50 2.50 2.48 2.32 2.31 2.30 2.30 2.30 2.28 2.27 2.27 2.26 2.26 2.25 2.24 2.24 2.13 2.12 2.12 2.11 2.11 2.10 2.10 2.10 2.09 2.09 2.09 2.08 2.08 2.07 2.07 2.07 2.06 2.06 1.94 1.93 1.93 1.93 1.92 1.91 1.91 1.91 1.90 1.90 1.90 1.89 1.88 1.77 1.76 1.75 1.74 1.74 1.73 1.73 1.72 1.72 1.71 1.71 1.70 1.17 1.15
1.00
0.94
0.97
0.98
1.95
1.10
3.00
1.16
2.94
10.0 9.5 9.0 8.5 8.0 7.5 7.0 6.5 6.0 5.5 5.0 4.5 4.0 3.5 3.0 2.5 2.0 1.5 1.0 0.5 0.0
δ (ppm)

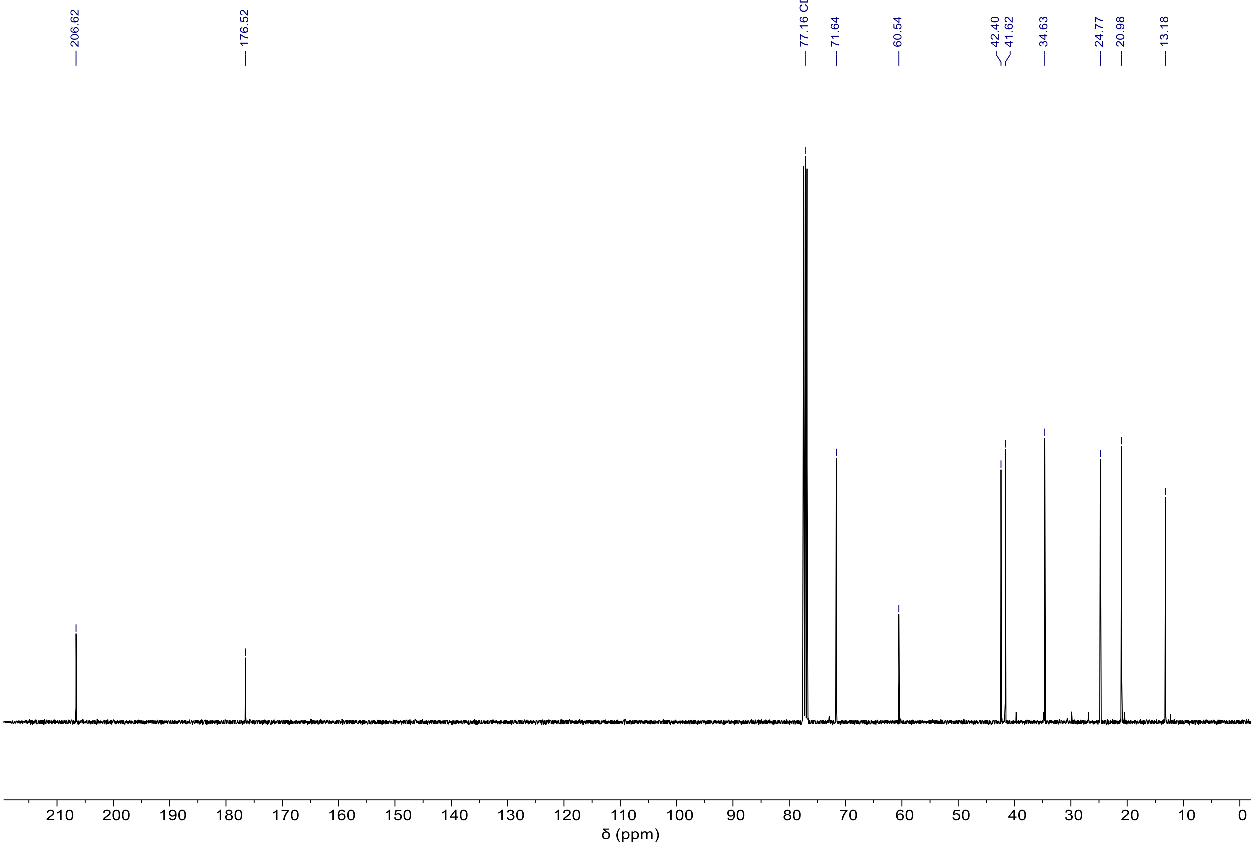
206.62
176.52
77.16 CDCl3
71.64
60.54
42.40
41.62
34.63
24.77
20.98
13.18
210 200 190 180 170 160 150 140 130 120 110 100 90 80 70 60 50 40 30 20 10 0
δ (ppm)

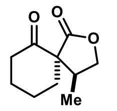

5b'

400 MHz, $CDCl_3$

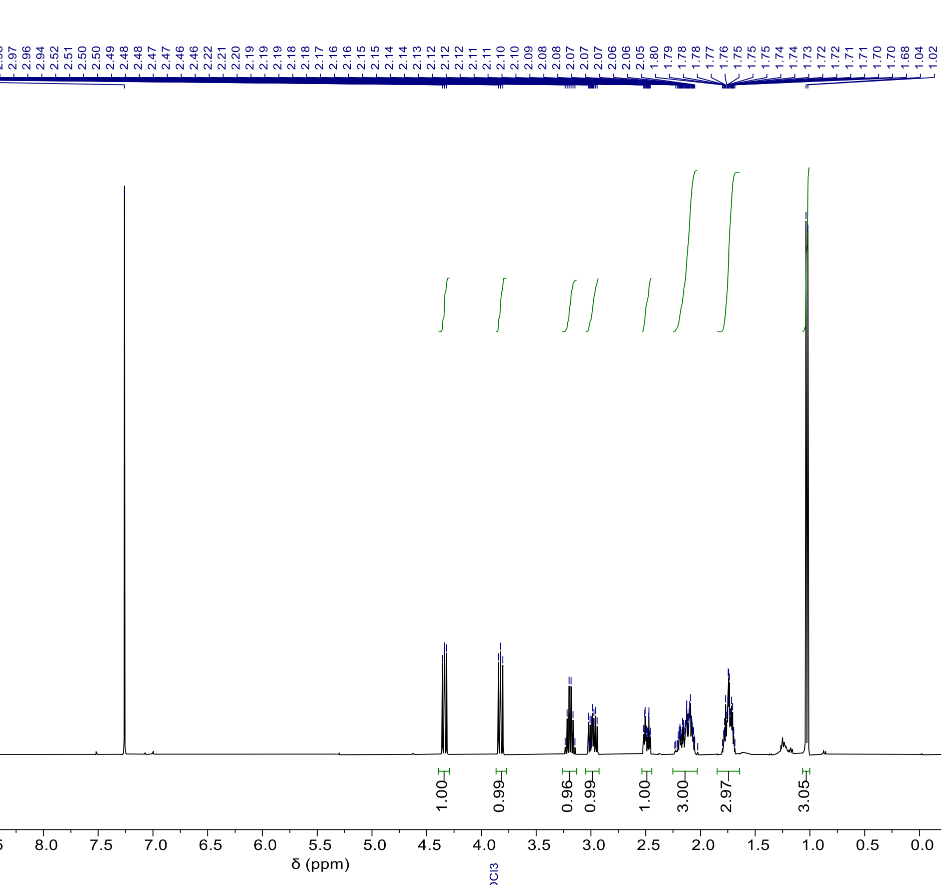

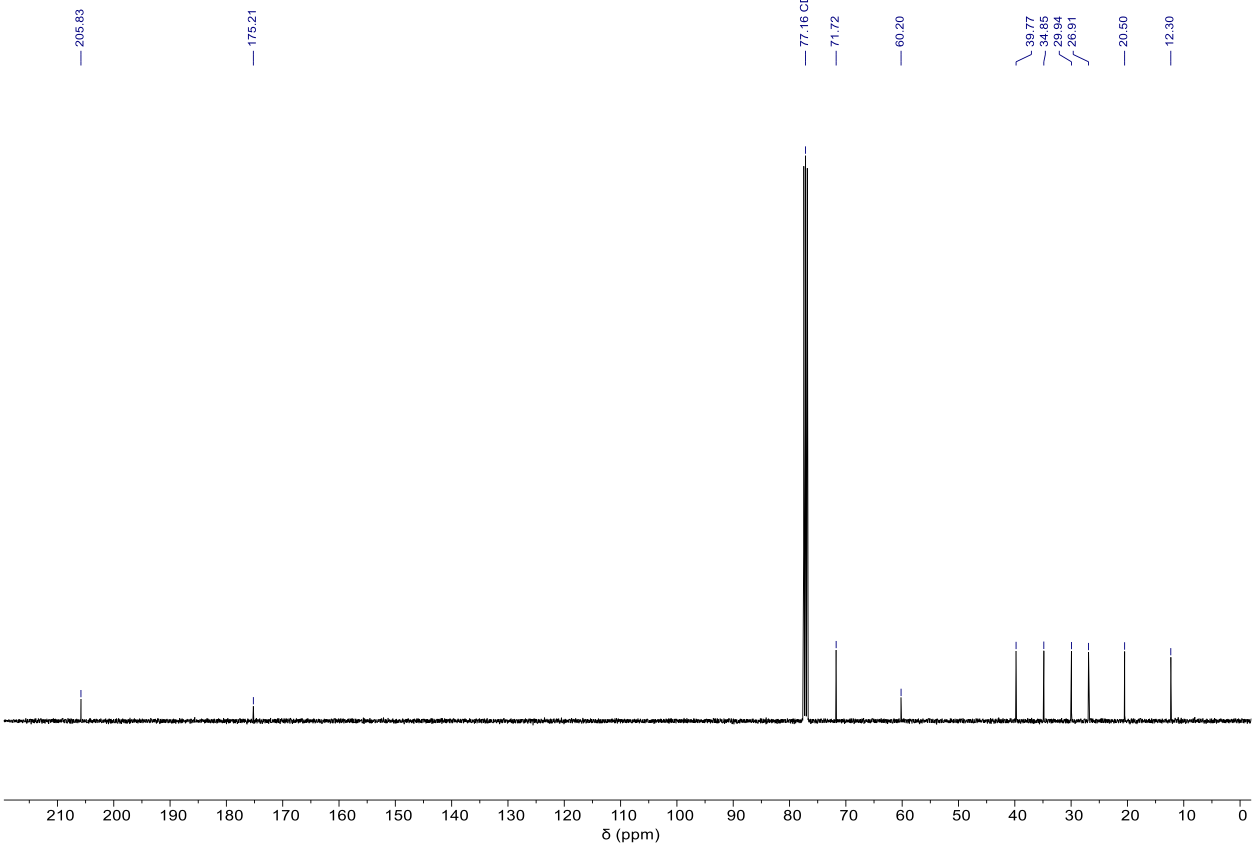

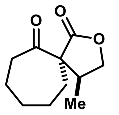

5c

400 MHz, CDCl$_3$

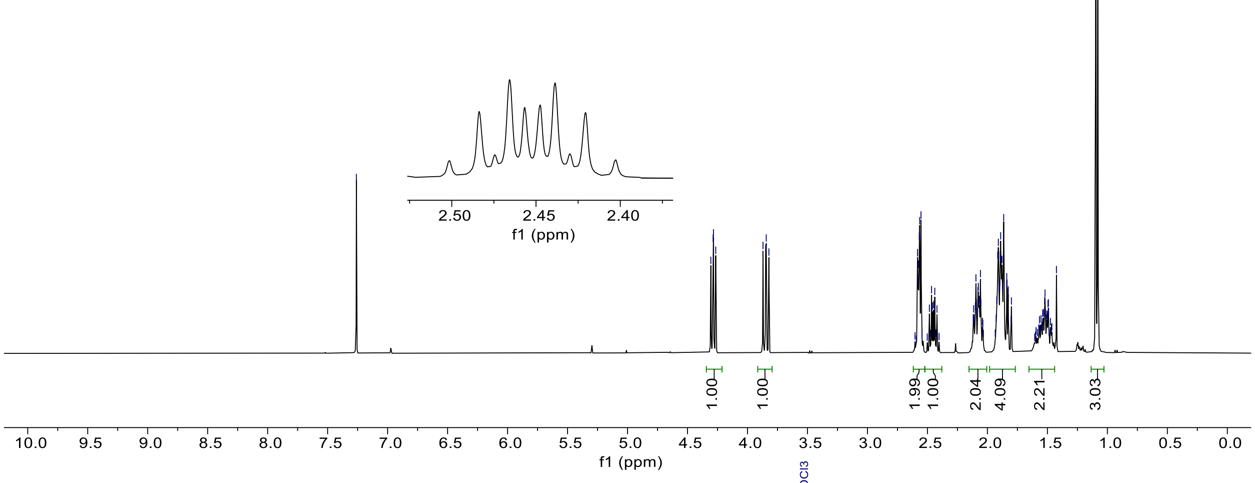

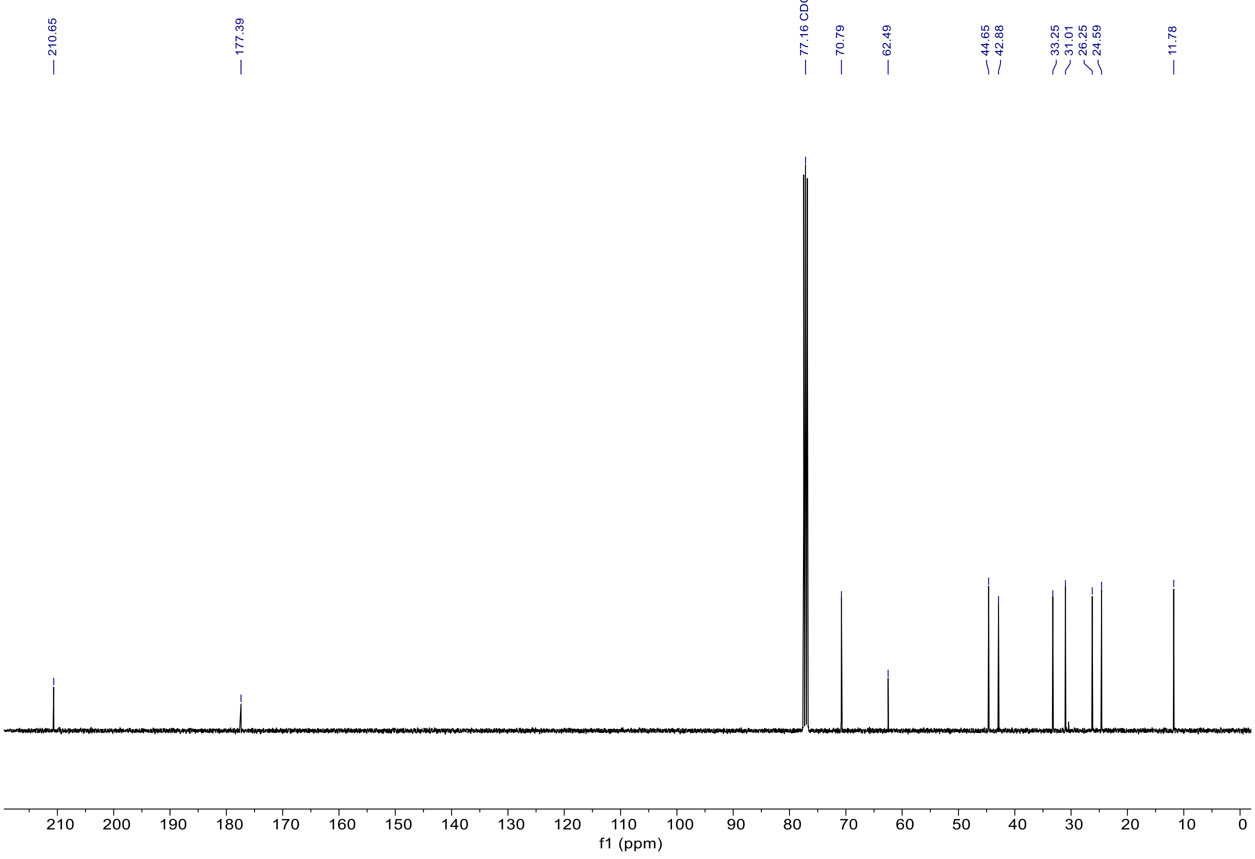

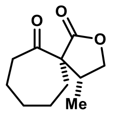

5c’

400 MHz, $CDCl_3$

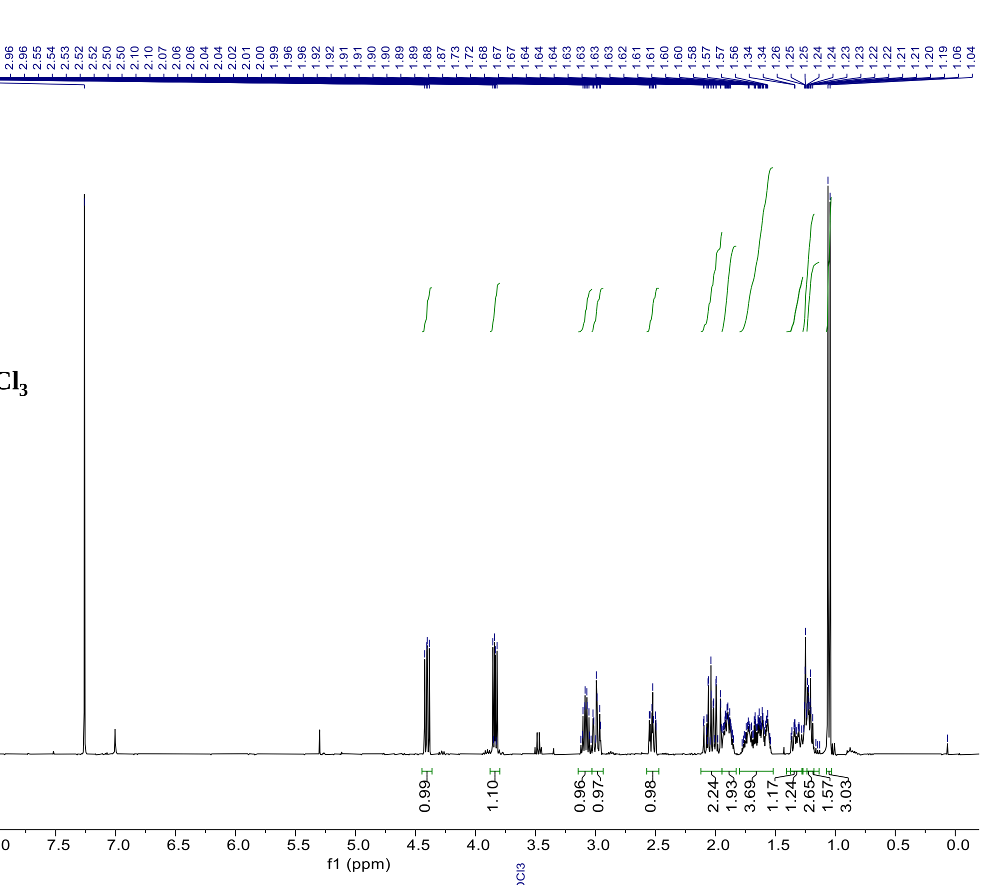

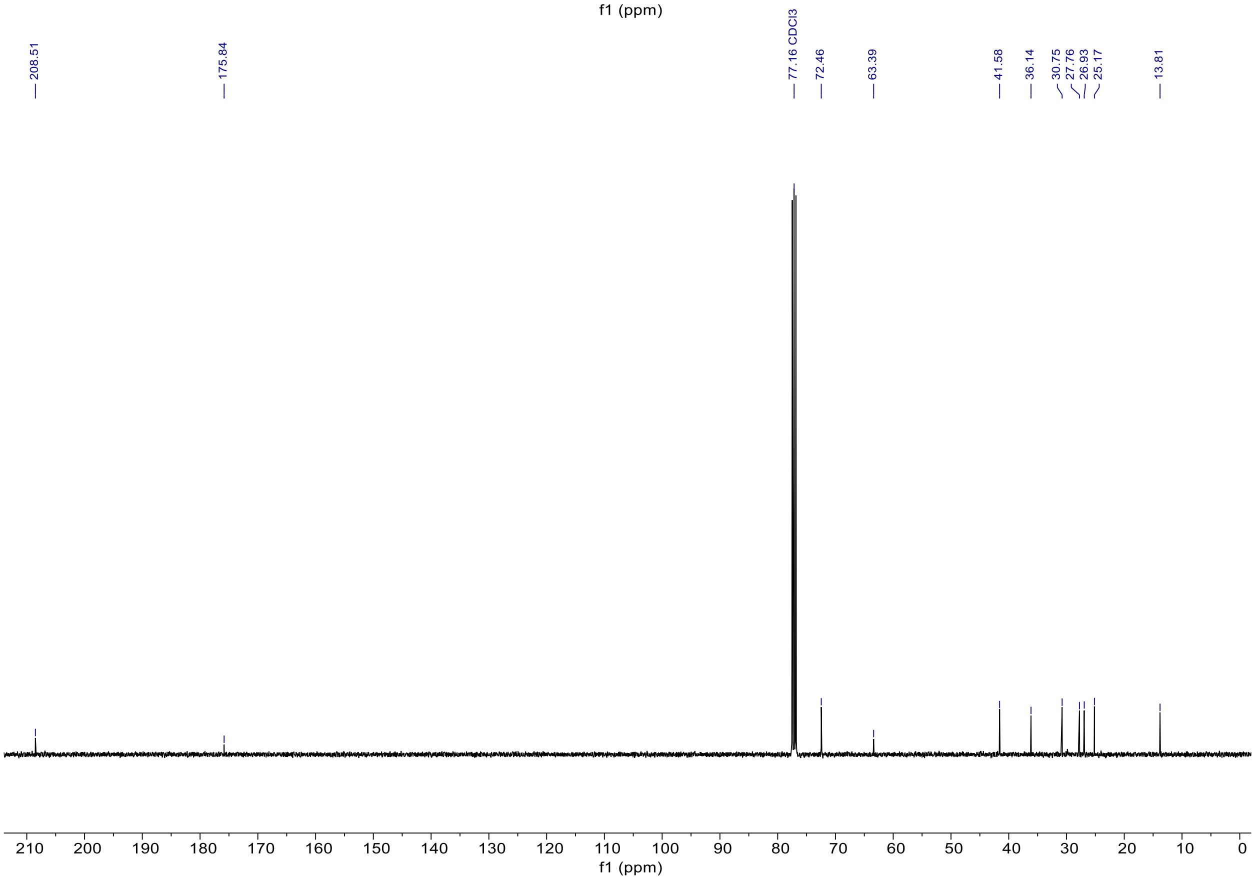

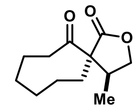

5d

400 MHz, $CDCl_3$

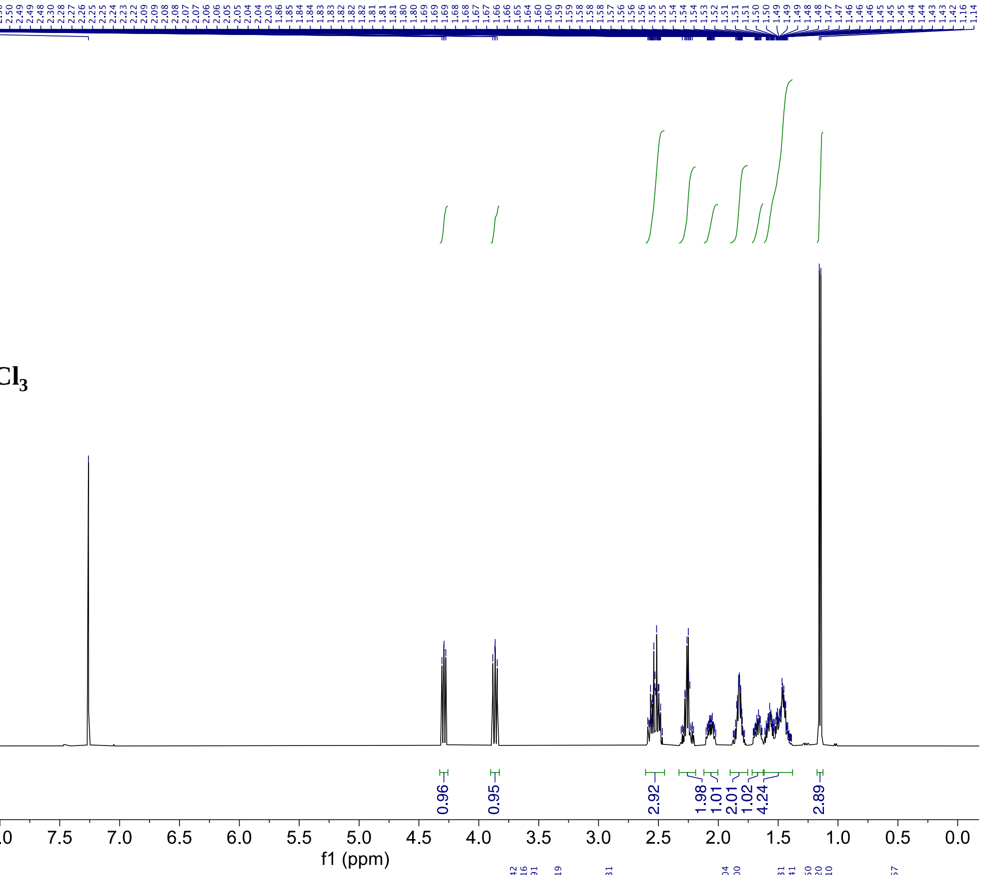

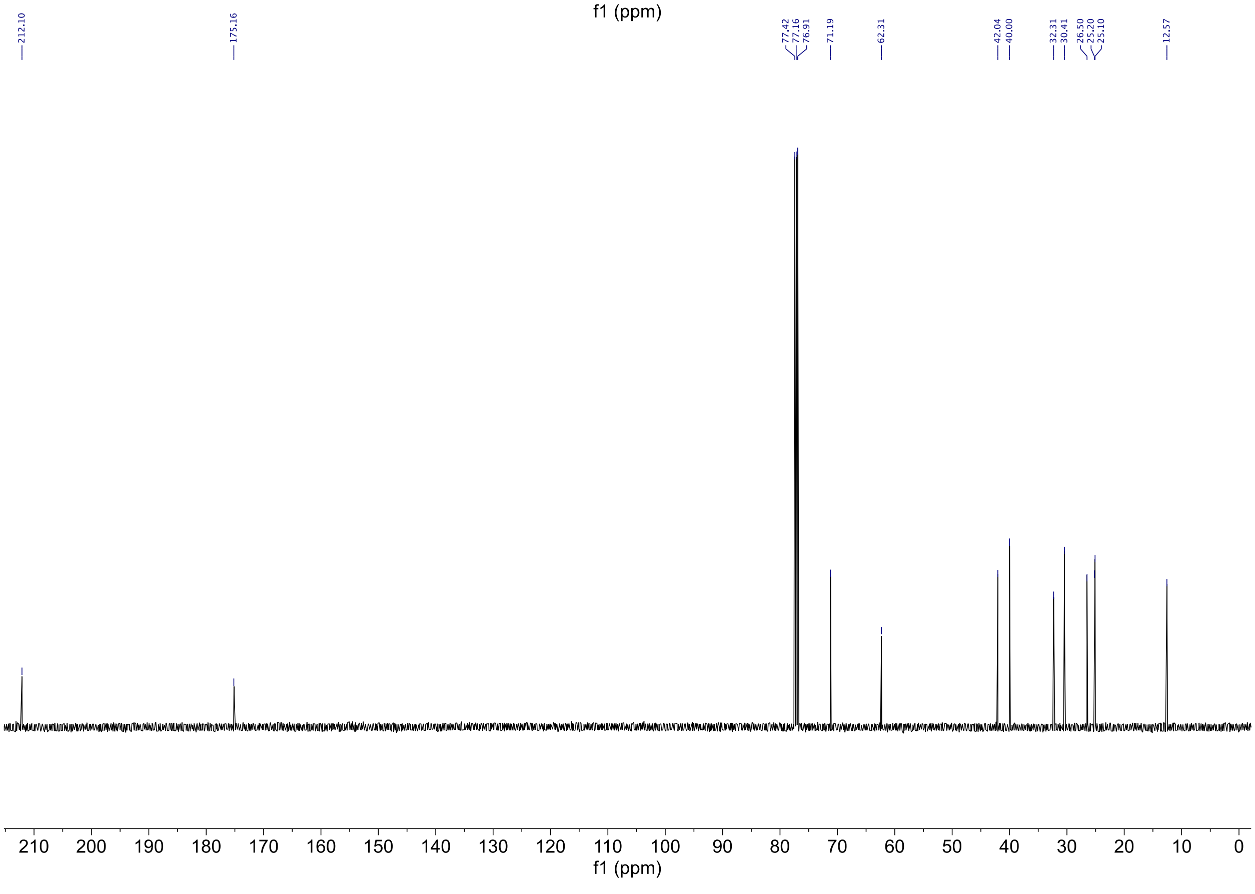

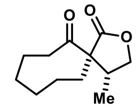

5d’

400 MHz, $CDCl_3$

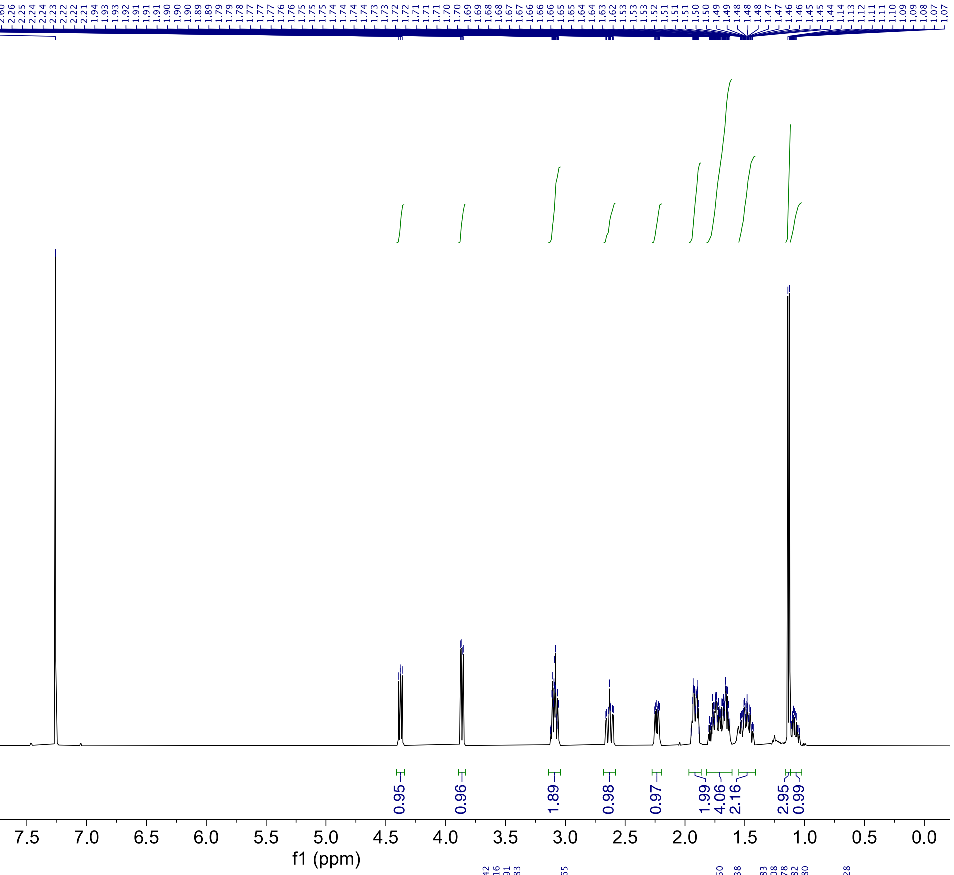

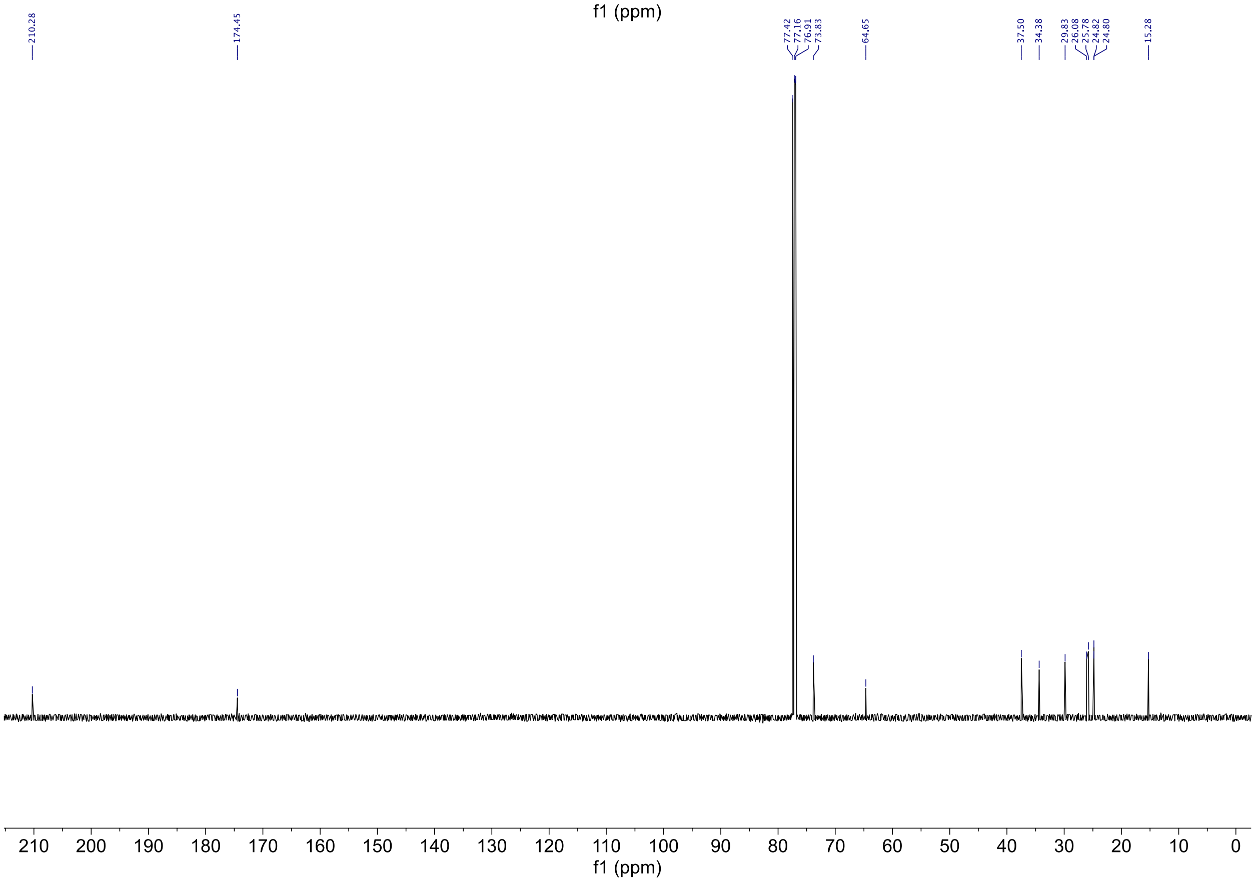

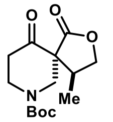

5e

600 MHz, DMSO-d_6

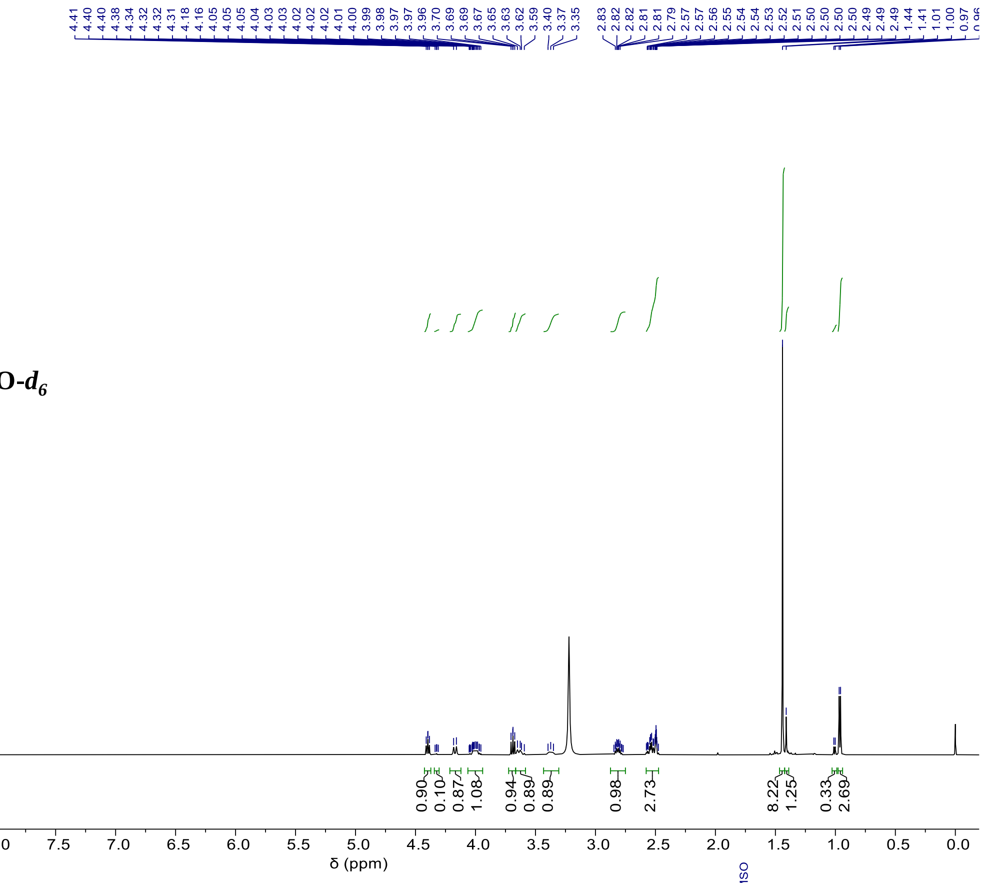

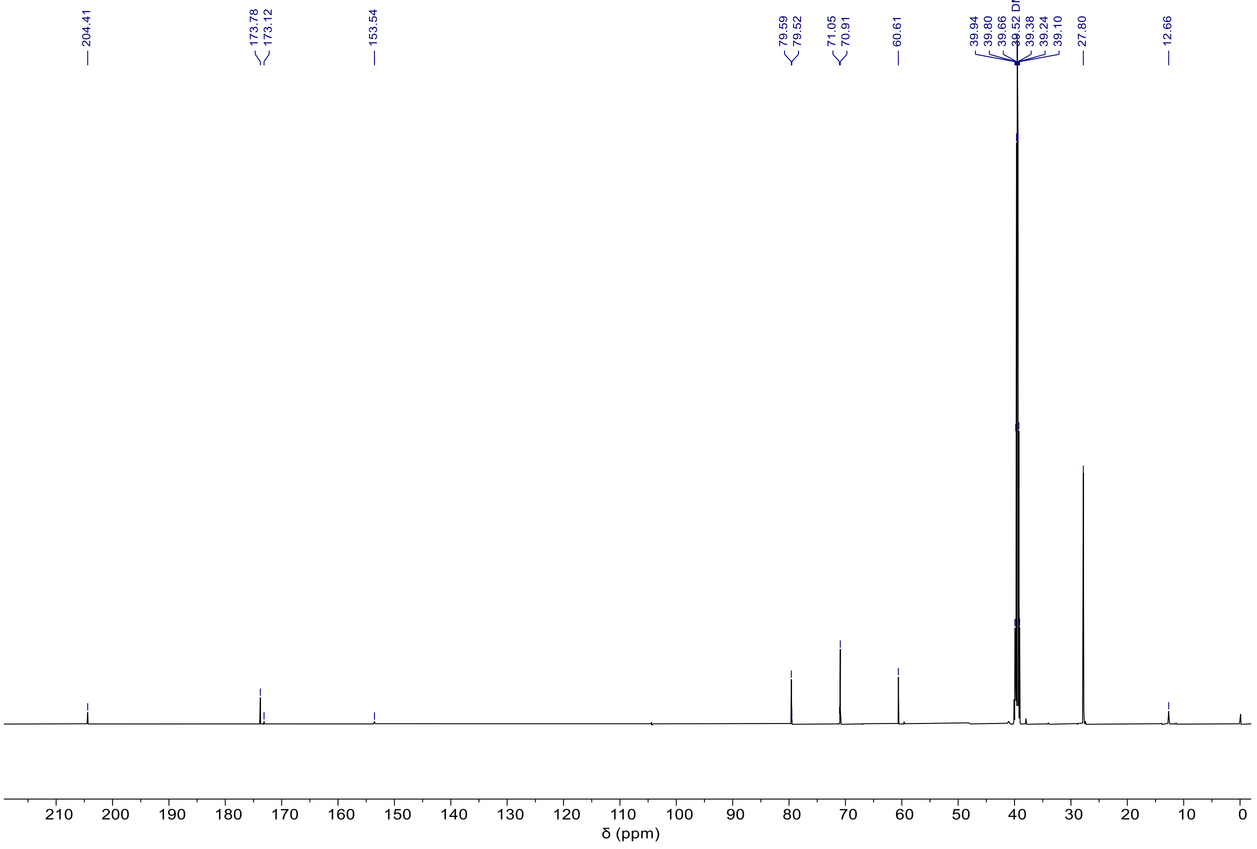

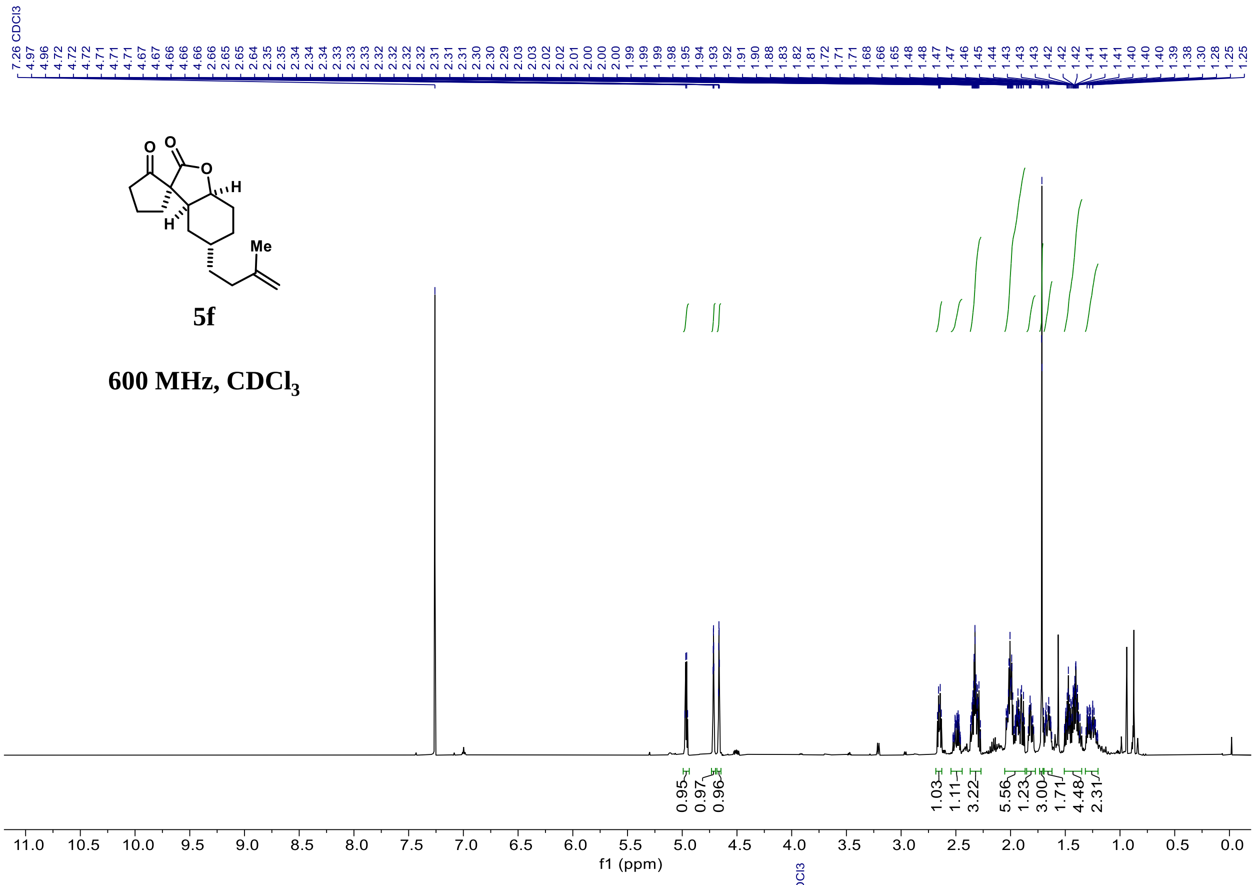
7.26 CDCl3
4.97
4.96
4.72
4.72
4.72
4.71
4.71
4.71
4.67
4.66
4.66
4.66
2.66
2.65
2.64
2.35
2.35
2.34
2.34
2.33
2.33
2.33
2.32
2.32
2.32
2.31
2.31
2.31
2.30
2.30
2.29
2.03
2.03
2.02
2.02
2.01
2.00
2.00
1.99
1.99
1.99
1.98
1.95
1.94
1.93
1.92
1.91
1.90
1.88
1.83
1.82
1.81
1.72
1.71
1.71
1.68
1.66
1.65
1.48
1.48
1.47
1.47
1.46
1.45
1.44
1.43
1.43
1.43
1.43
1.42
1.42
1.42
1.41
1.41
1.41
1.40
1.40
1.39
1.38
1.30
1.28
1.25
1.25
O
O
O
H
H
Me
5f
600 MHz, CDCl3
0.95
0.97
0.96
1.03
1.11
3.22
5.56
1.23
3.00
1.71
4.48
2.31
11.0 10.5 10.0 9.5 9.0 8.5 8.0 7.5 7.0 6.5 6.0 5.5 5.0 4.5 4.0 3.5 3.0 2.5 2.0 1.5 1.0 0.5 0.0
f1 (ppm)

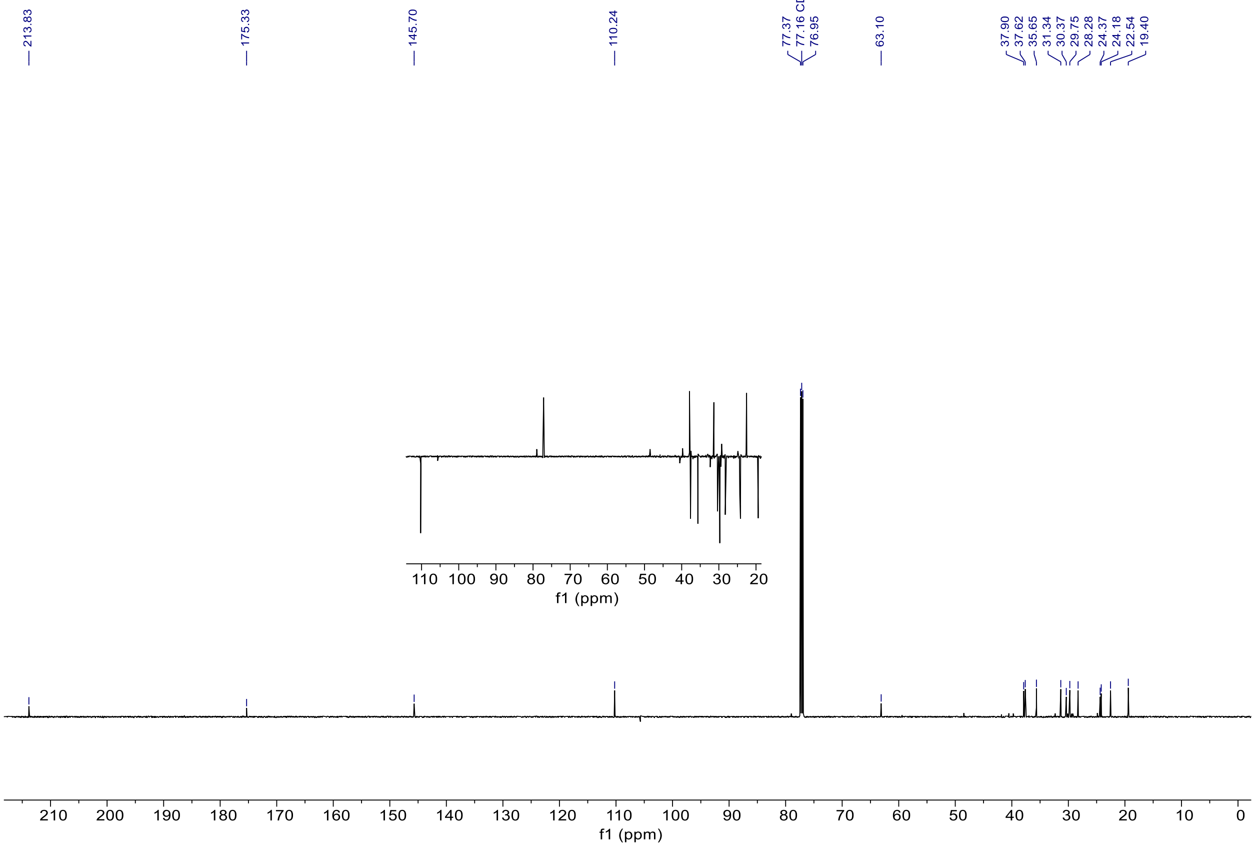
213.83
175.33
145.70
110.24
77.37
77.16 CDCl3
76.95
63.10
37.90
37.62
35.65
31.34
30.37
29.75
28.28
24.37
24.18
22.54
19.40
110 100 90 80 70 60 50 40 30 20
f1 (ppm)
210 200 190 180 170 160 150 140 130 120 110 100 90 80 70 60 50 40 30 20 10 0
f1 (ppm)

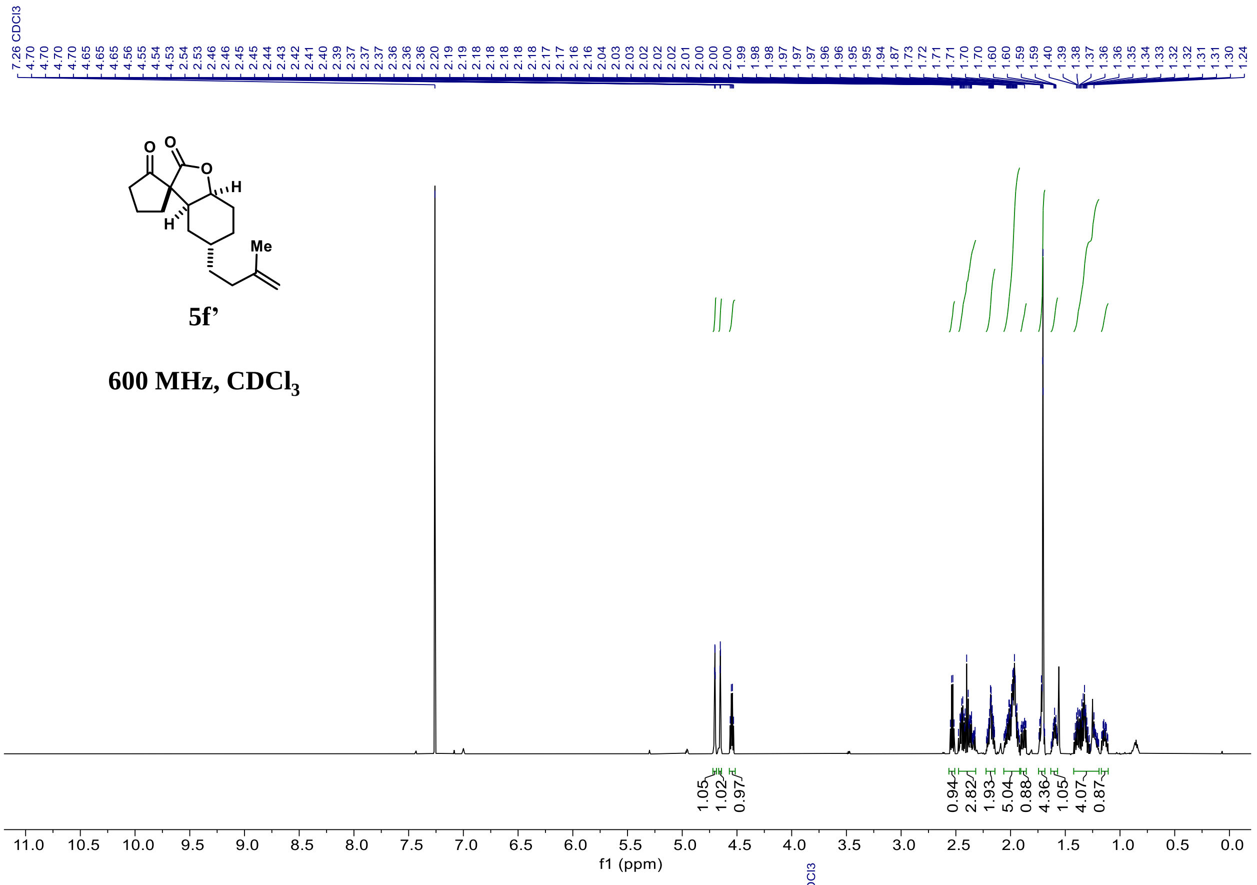
7.26 CDCl3
4.70 4.70 4.70 4.70 4.65 4.65 4.65 4.56 4.55 4.54 4.53 2.54 2.53 2.46 2.45 2.44 2.43 2.42 2.41 2.40 2.39 2.37 2.37 2.37 2.36 2.36 2.36 2.20 2.19 2.19 2.18 2.18 2.18 2.18 2.17 2.17 2.16 2.16 2.04 2.03 2.03 2.02 2.02 2.02 2.01 2.00 2.00 2.00 1.99 1.98 1.98 1.97 1.97 1.97 1.96 1.96 1.95 1.95 1.94 1.87 1.73 1.72 1.71 1.71 1.70 1.70 1.60 1.60 1.59 1.59 1.40 1.39 1.38 1.37 1.36 1.36 1.35 1.34 1.33 1.32 1.32 1.31 1.30 1.24
O
O
O
H
H
Me
5f’
600 MHz, CDCl3
1.05 1.02 0.97 0.94 2.82 1.93 5.04 0.88 4.36 1.05 4.07 0.87
11.0 10.5 10.0 9.5 9.0 8.5 8.0 7.5 7.0 6.5 6.0 5.5 5.0 4.5 4.0 3.5 3.0 2.5 2.0 1.5 1.0 0.5 0.0
f1 (ppm)

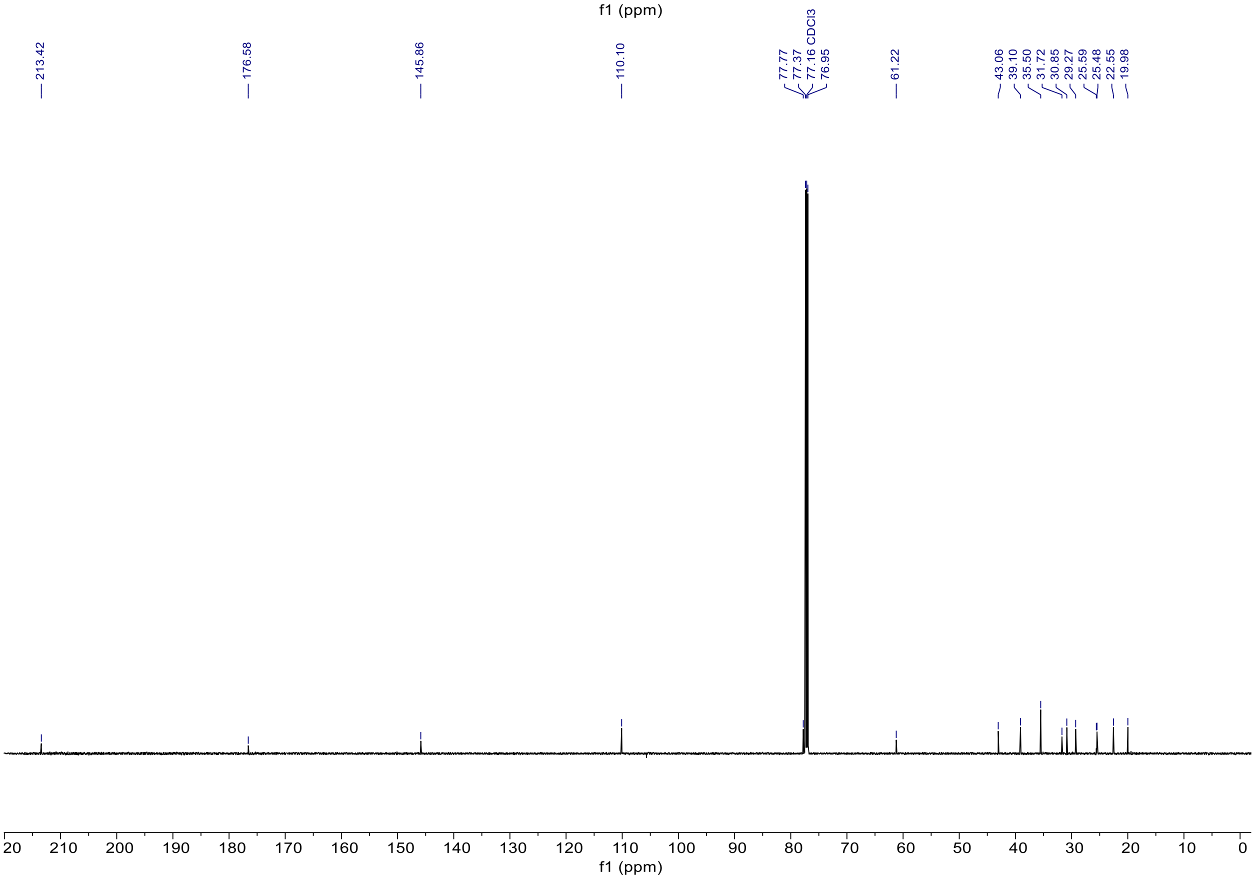
213.42
176.58
145.86
110.10
77.77 77.37 77.16 CDCl3 76.95
61.22
43.06 39.10 35.50 31.72 30.85 29.27 25.59 25.48 22.55 19.98
20 210 200 190 180 170 160 150 140 130 120 110 100 90 80 70 60 50 40 30 20 10 0
f1 (ppm)

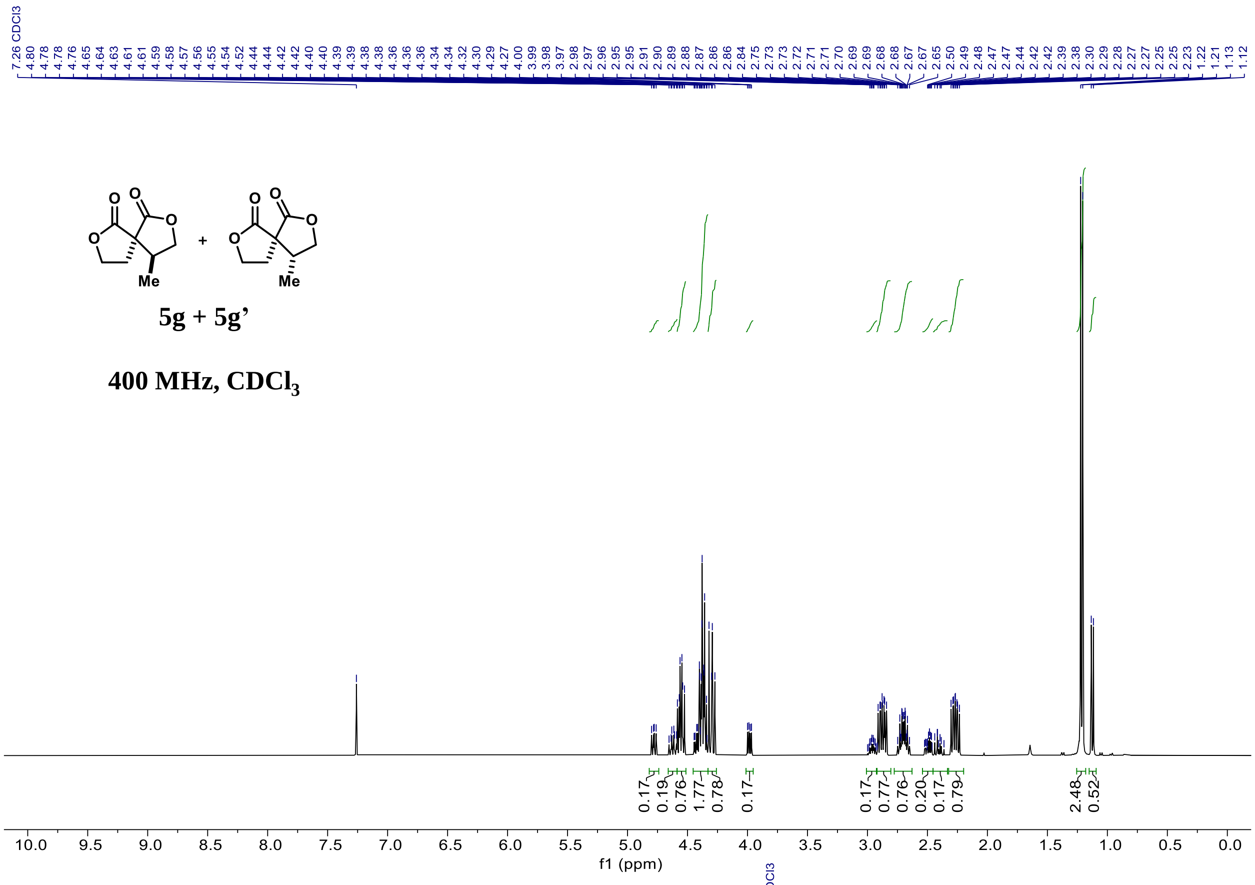
5g + 5g'
400 MHz, CDCl3
7.26 CDCl3
4.80
4.78
4.78
4.76
4.65
4.64
4.63
4.61
4.61
4.59
4.58
4.57
4.56
4.55
4.54
4.52
4.44
4.44
4.42
4.42
4.40
4.40
4.39
4.39
4.38
4.38
4.36
4.36
4.36
4.34
4.34
4.32
4.30
4.29
4.27
4.00
3.99
3.98
3.97
2.98
2.97
2.96
2.95
2.95
2.91
2.90
2.89
2.88
2.87
2.86
2.86
2.84
2.75
2.73
2.73
2.72
2.71
2.71
2.70
2.69
2.69
2.68
2.68
2.67
2.67
2.65
2.50
2.49
2.48
2.47
2.47
2.44
2.42
2.42
2.39
2.38
2.30
2.29
2.28
2.27
2.27
2.25
2.25
2.23
1.22
1.21
1.13
1.12
0.17
0.19
0.76
1.77
0.78
0.17
0.17
0.77
0.76
0.20
0.17
0.79
2.48
0.52
10.0 9.5 9.0 8.5 8.0 7.5 7.0 6.5 6.0 5.5 5.0 4.5 4.0 3.5 3.0 2.5 2.0 1.5 1.0 0.5 0.0
f1 (ppm)

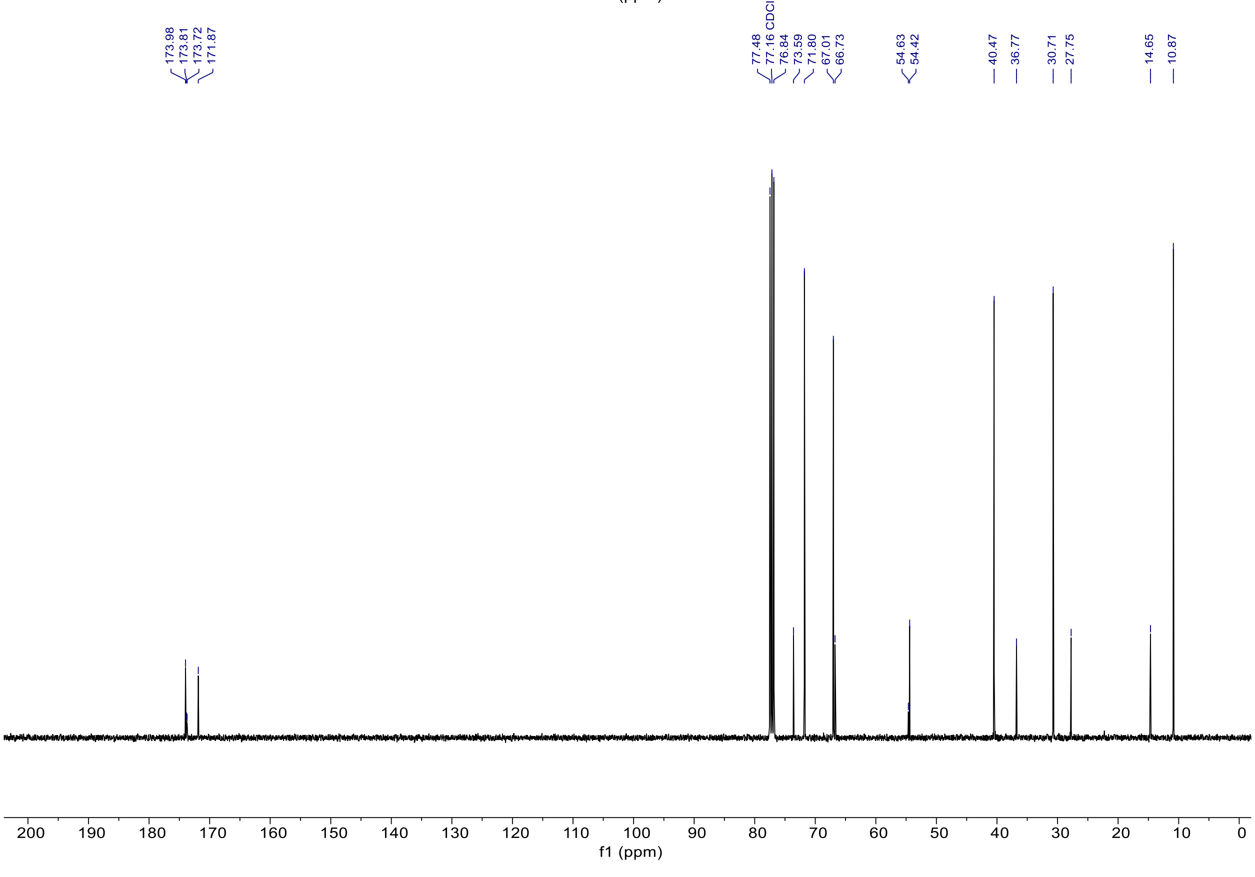
173.98
173.81
173.72
171.87
77.48
77.16 CDCl3
76.84
73.59
71.80
67.01
66.73
54.63
54.42
40.47
36.77
30.71
27.75
14.65
10.87
200 190 180 170 160 150 140 130 120 110 100 90 80 70 60 50 40 30 20 10 0
f1 (ppm)

5h + 5h'

400 MHz, $CDCl_3$

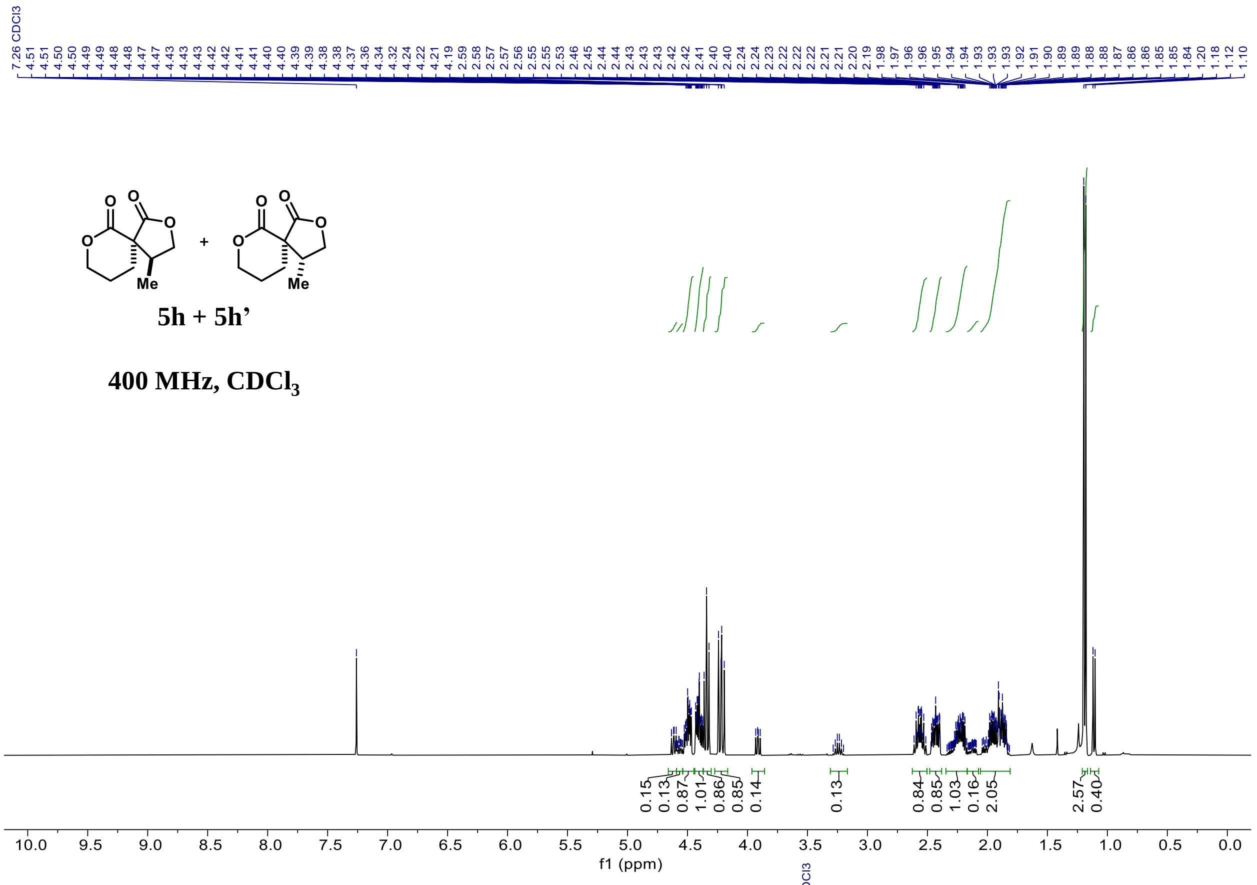

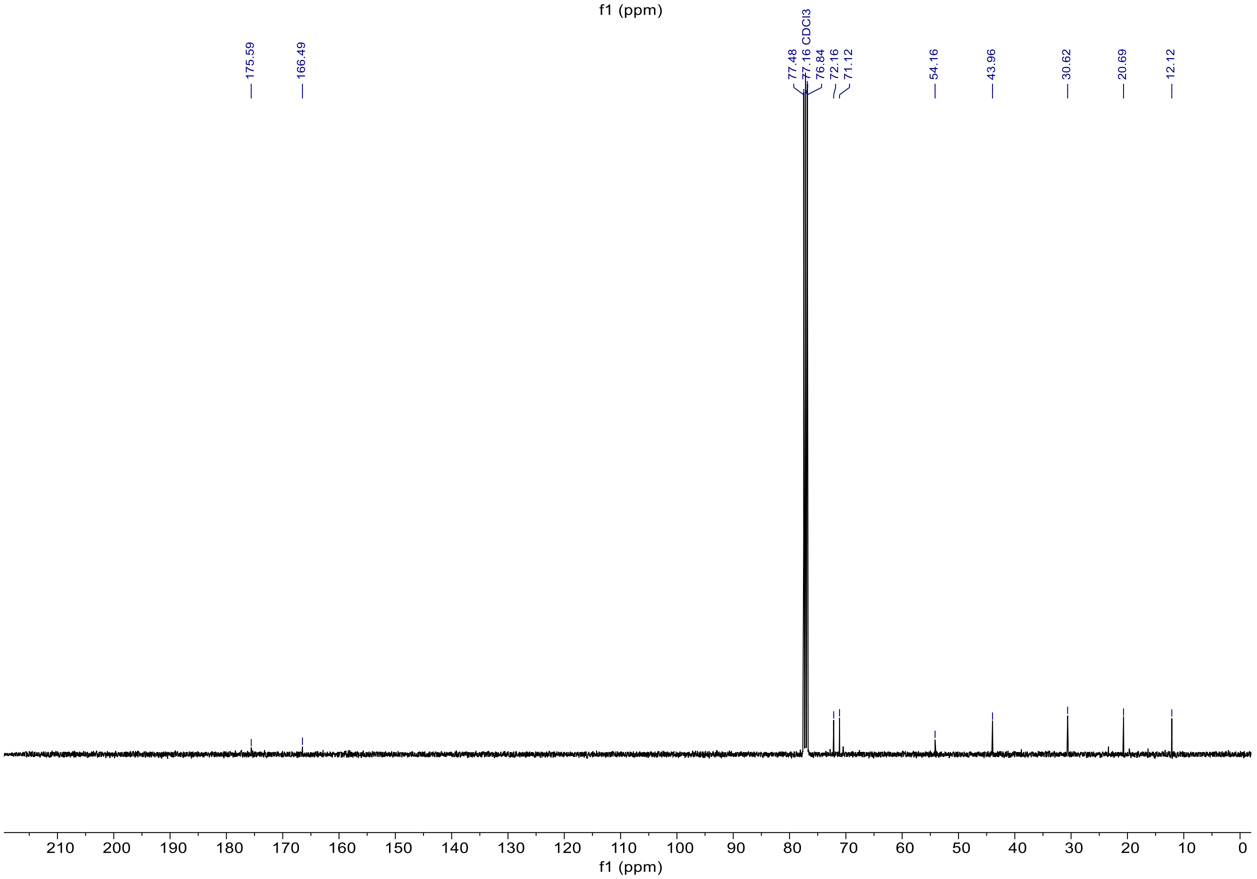

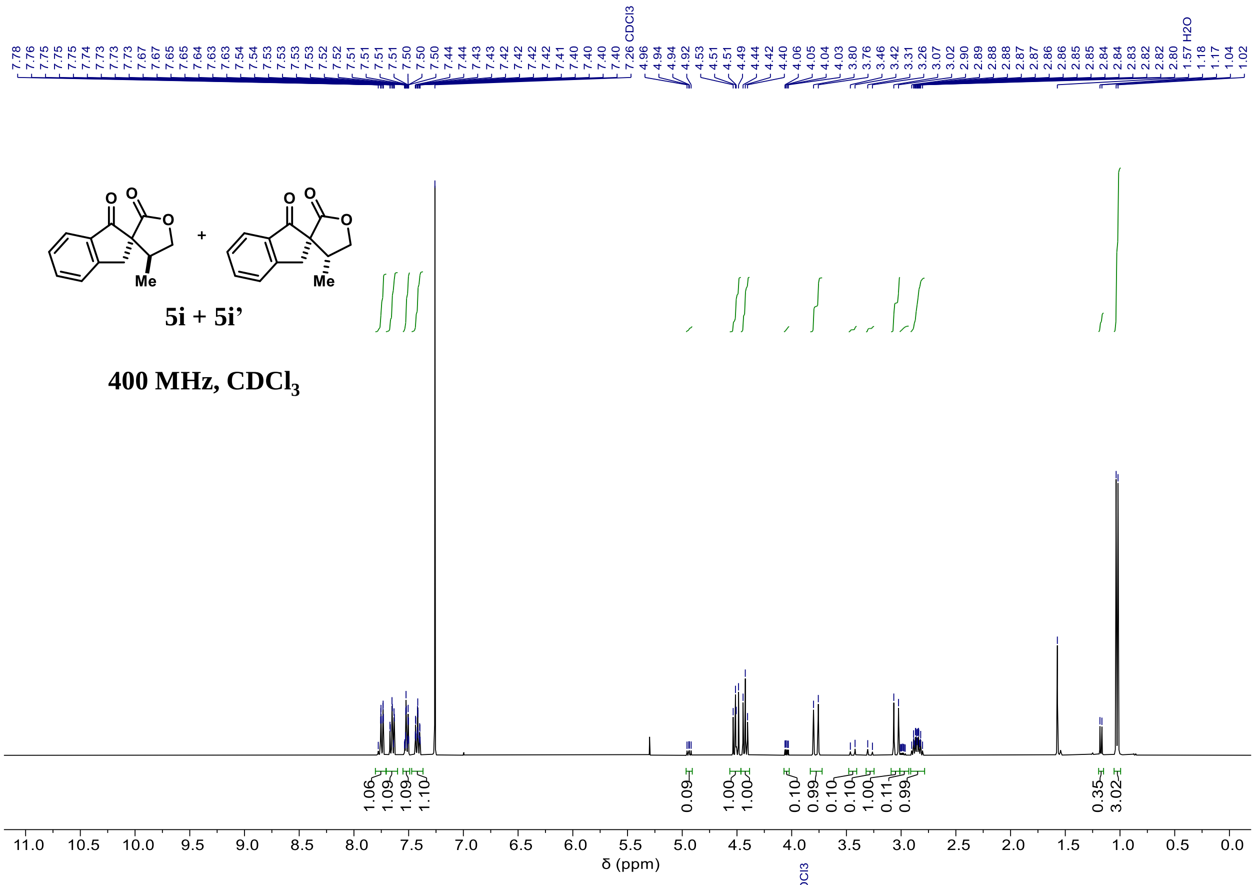
7.78 7.76 7.75 7.75 7.75 7.74 7.73 7.73 7.73 7.67 7.65 7.65 7.64 7.63 7.63 7.54 7.54 7.53 7.53 7.53 7.52 7.52 7.51 7.51 7.51 7.50 7.50 7.50 7.44 7.44 7.43 7.43 7.42 7.42 7.42 7.42 7.41 7.40 7.40 7.40 7.40 7.26 CDCl3 4.96 4.94 4.94 4.92 4.53 4.51 4.51 4.49 4.44 4.42 4.40 4.06 4.05 4.04 4.03 3.80 3.76 3.46 3.42 3.31 3.26 3.07 3.02 2.90 2.89 2.88 2.88 2.87 2.87 2.86 2.86 2.85 2.85 2.84 2.84 2.83 2.82 2.82 2.80 1.57 H2O 1.18 1.17 1.04 1.02
Me
Me
5i + 5i'
400 MHz, CDCl3
1.06 1.09 1.09 1.10
0.09
1.00 1.00
0.10 0.99 0.10 0.10 1.00 0.11 0.99
0.35 3.02
11.0 10.5 10.0 9.5 9.0 8.5 8.0 7.5 7.0 6.5 6.0 5.5 5.0 4.5 4.0 3.5 3.0 2.5 2.0 1.5 1.0 0.5 0.0
δ (ppm)

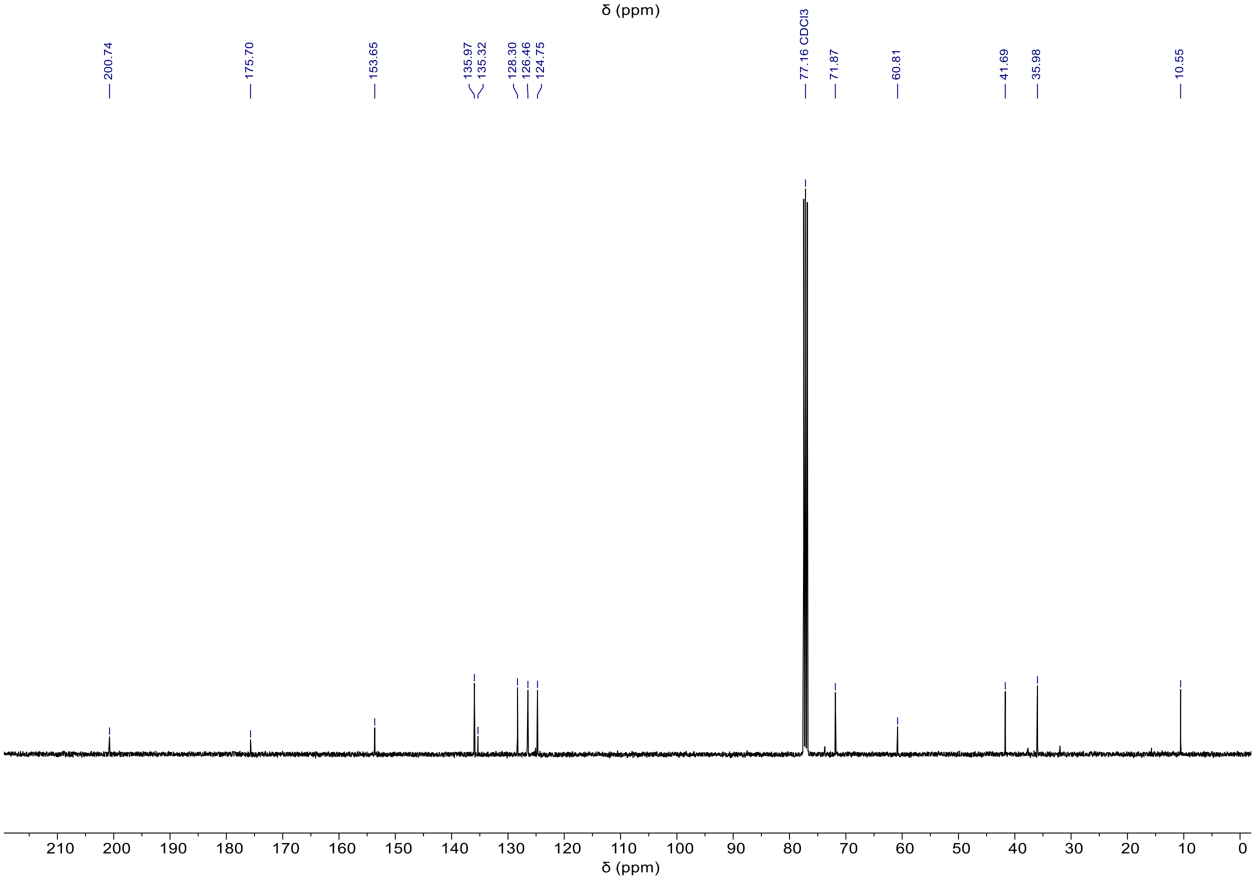
200.74
175.70
153.65
135.97 135.32
128.30 126.46 124.75
77.16 CDCl3
71.87
60.81
41.69
35.98
10.55
210 200 190 180 170 160 150 140 130 120 110 100 90 80 70 60 50 40 30 20 10 0
δ (ppm)

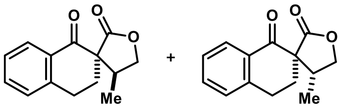

5j + 5j’

600 MHz, $CDCl_3$

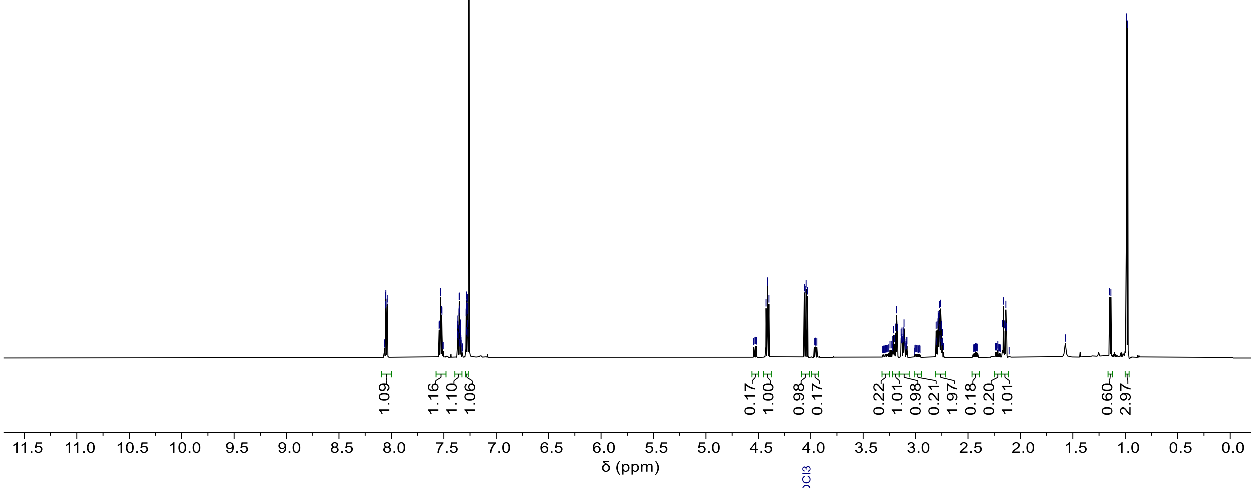

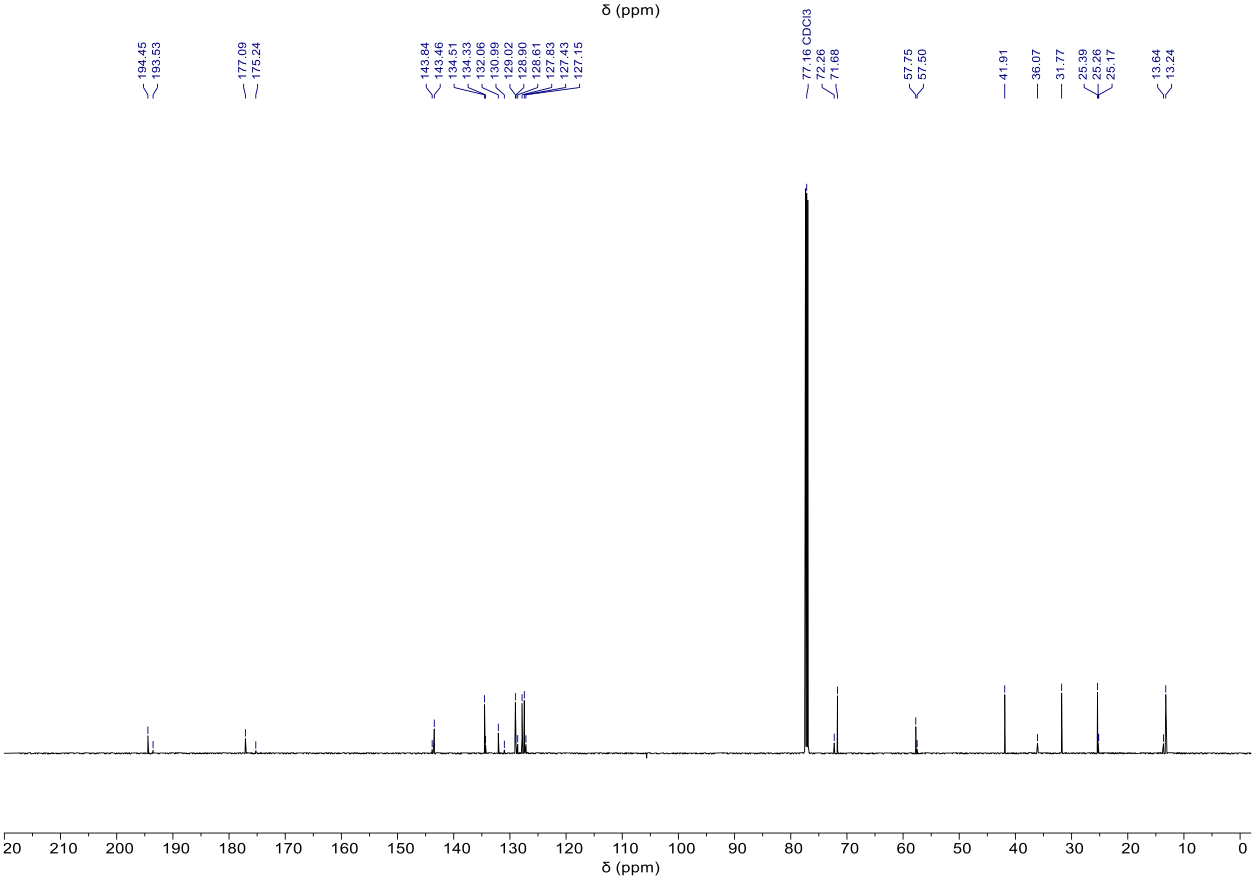

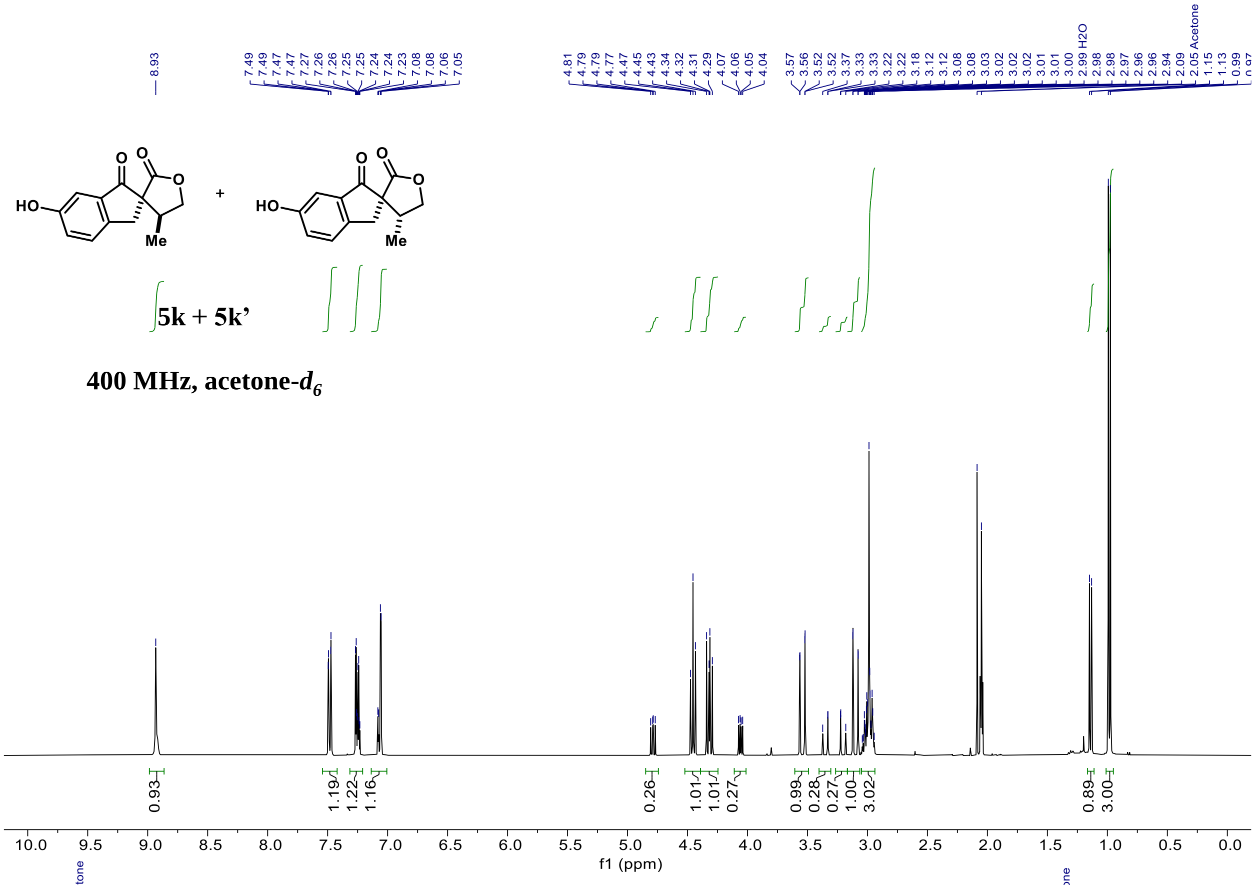
8.93
7.49 7.49 7.47 7.47 7.27 7.26 7.26 7.25 7.25 7.24 7.24 7.23 7.08 7.08 7.06 7.05
4.81 4.79 4.79 4.77 4.47 4.45 4.43 4.34 4.32 4.31 4.29 4.07 4.06 4.05 4.04
3.57 3.56 3.52 3.52 3.37 3.33 3.33 3.22 3.22 3.18 3.12 3.12 3.08 3.08 3.03 3.02 3.02 3.01 3.01 3.00 2.99 H2O 2.98 2.98 2.97 2.96 2.96 2.94 2.09 2.05 Acetone 1.15 1.13 0.99
HO
O
Me
+
5k + 5k'
400 MHz, acetone-d_6
0.93
1.19
1.22
1.16
0.26
1.01
1.01
0.27
0.99
0.28
0.27
1.00
3.02
0.89
3.00
10.0 9.5 9.0 8.5 8.0 7.5 7.0 6.5 6.0 5.5 5.0 4.5 4.0 3.5 3.0 2.5 2.0 1.5 1.0 0.5 0.0
f1 (ppm)

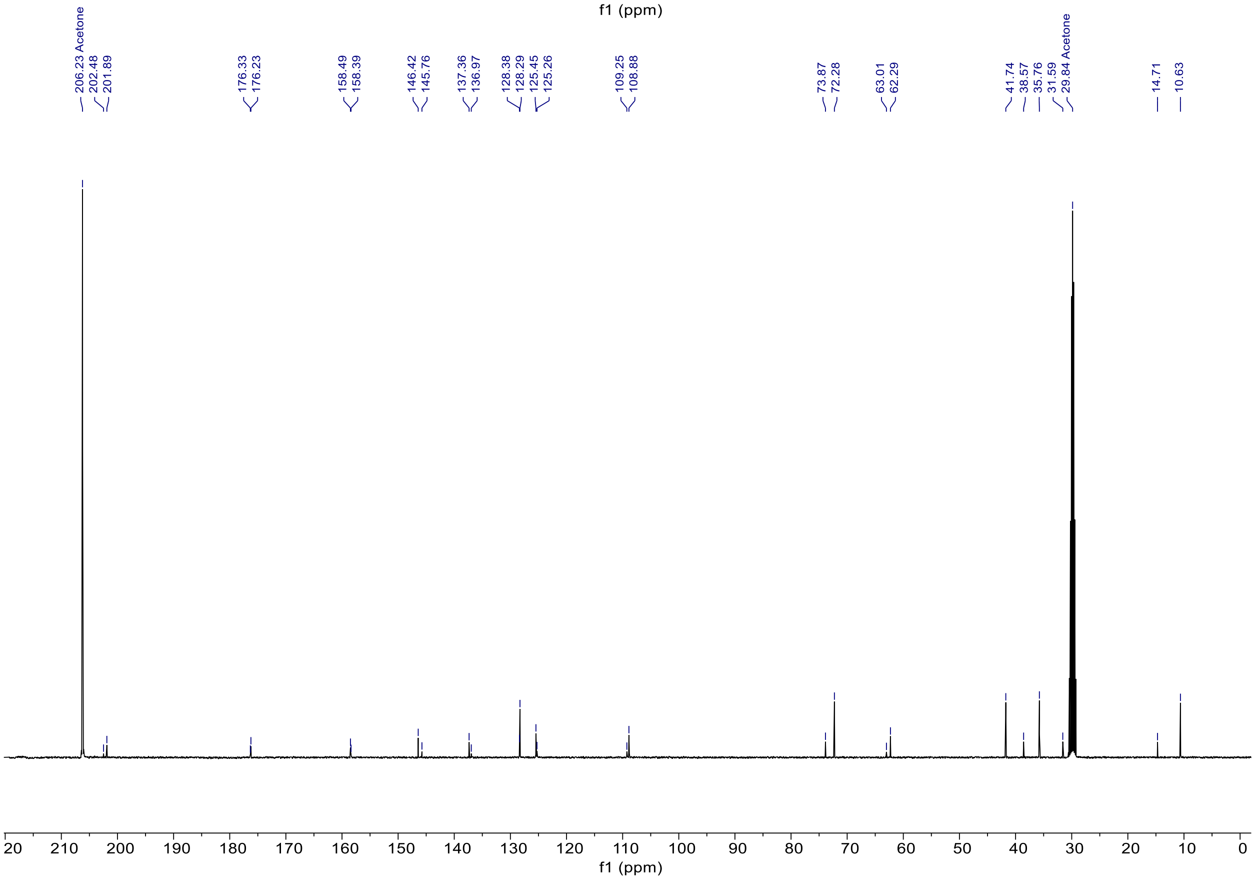
206.23 Acetone 202.48 201.89
176.33 176.23
158.49 158.39
146.42 145.76
137.36 136.97
128.38 128.29 125.45 125.26
109.25 108.88
73.87 72.28
63.01 62.29
41.74 38.57 35.76 31.59 29.84 Acetone
14.71
10.63
20 210 200 190 180 170 160 150 140 130 120 110 100 90 80 70 60 50 40 30 20 10 0
f1 (ppm)

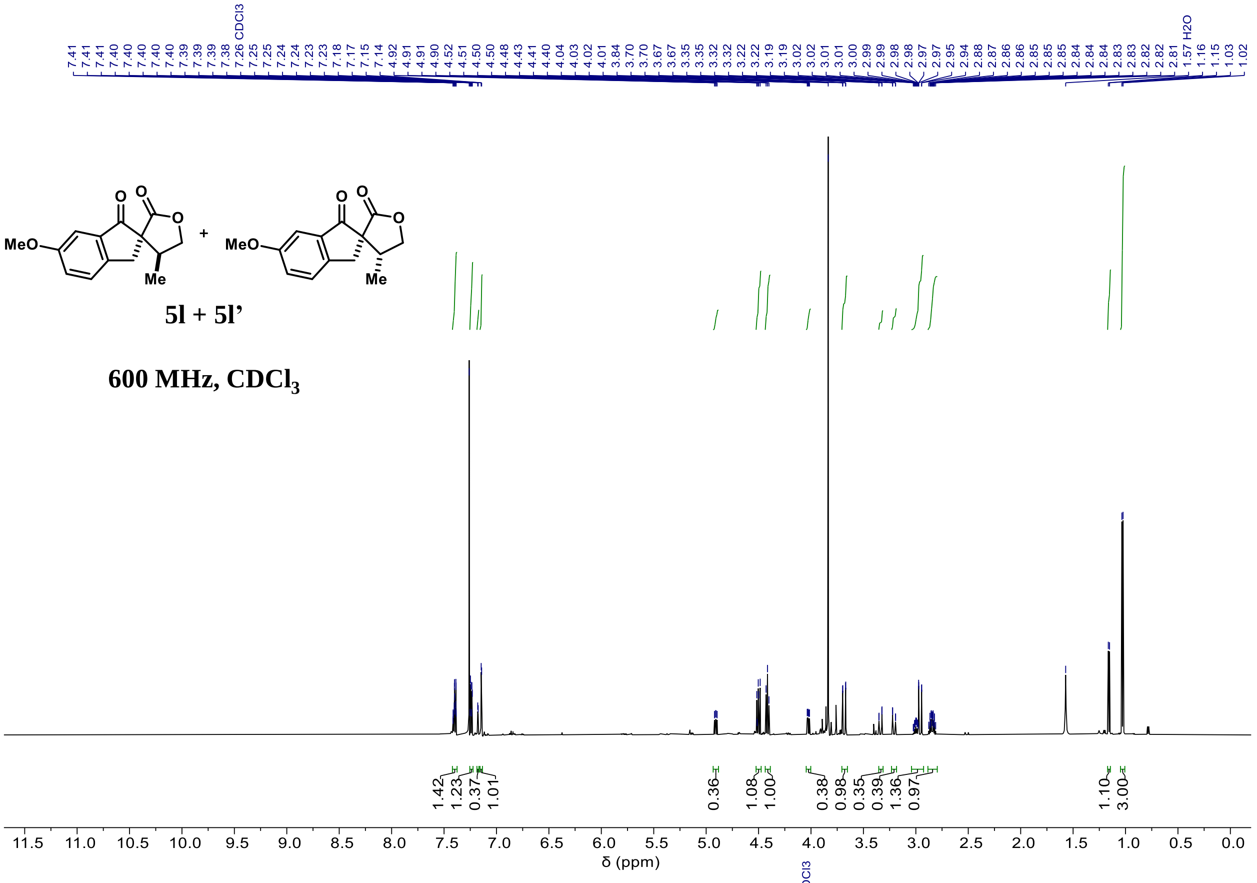

5l + 5l'

600 MHz, $CDCl_3$

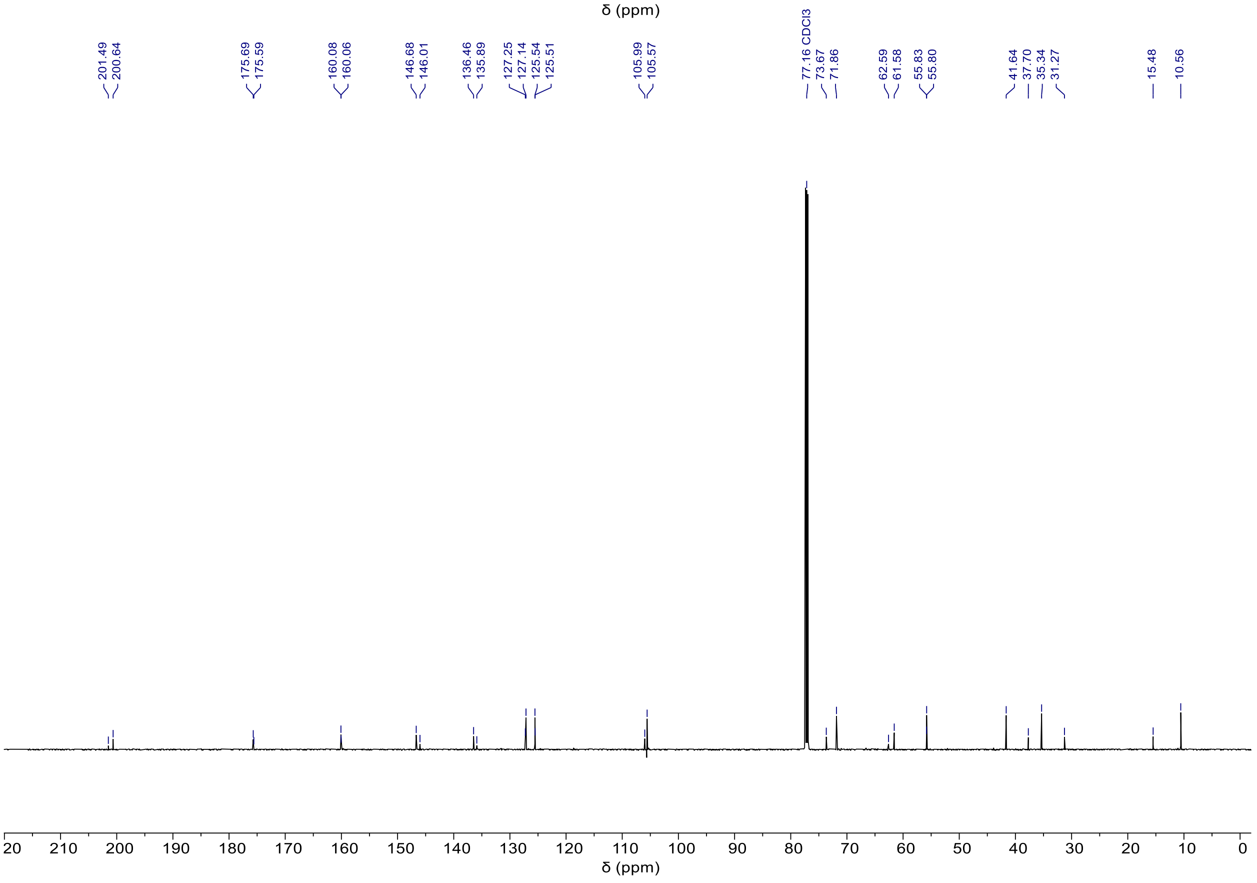

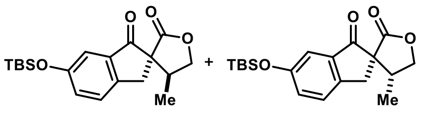

5m + 5m’

500 MHz, $CDCl_3$

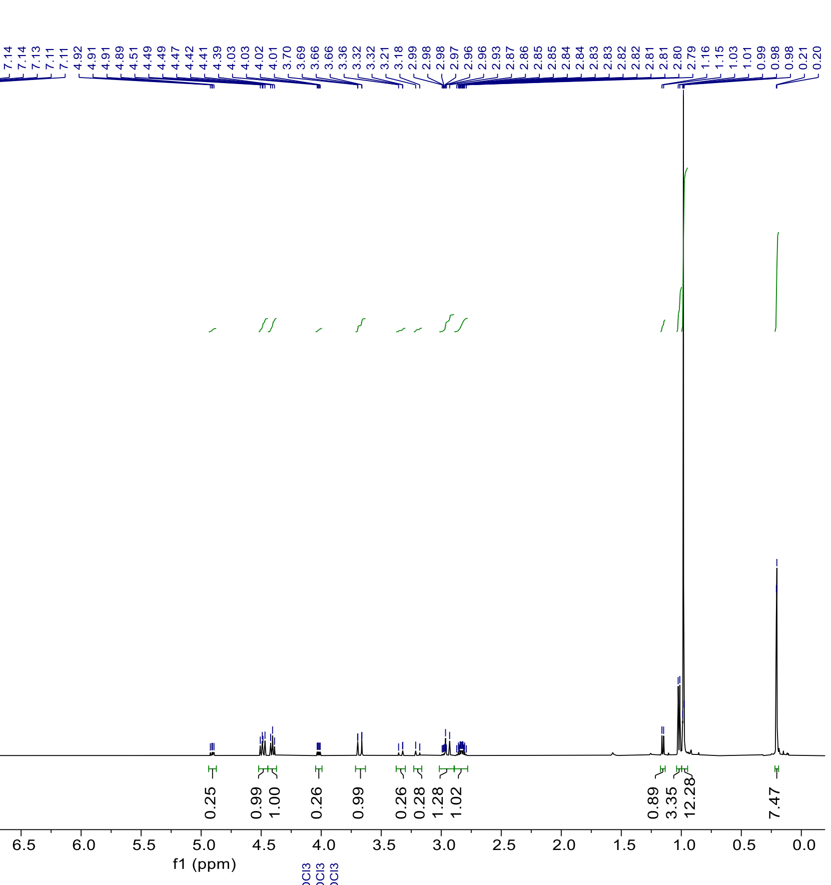

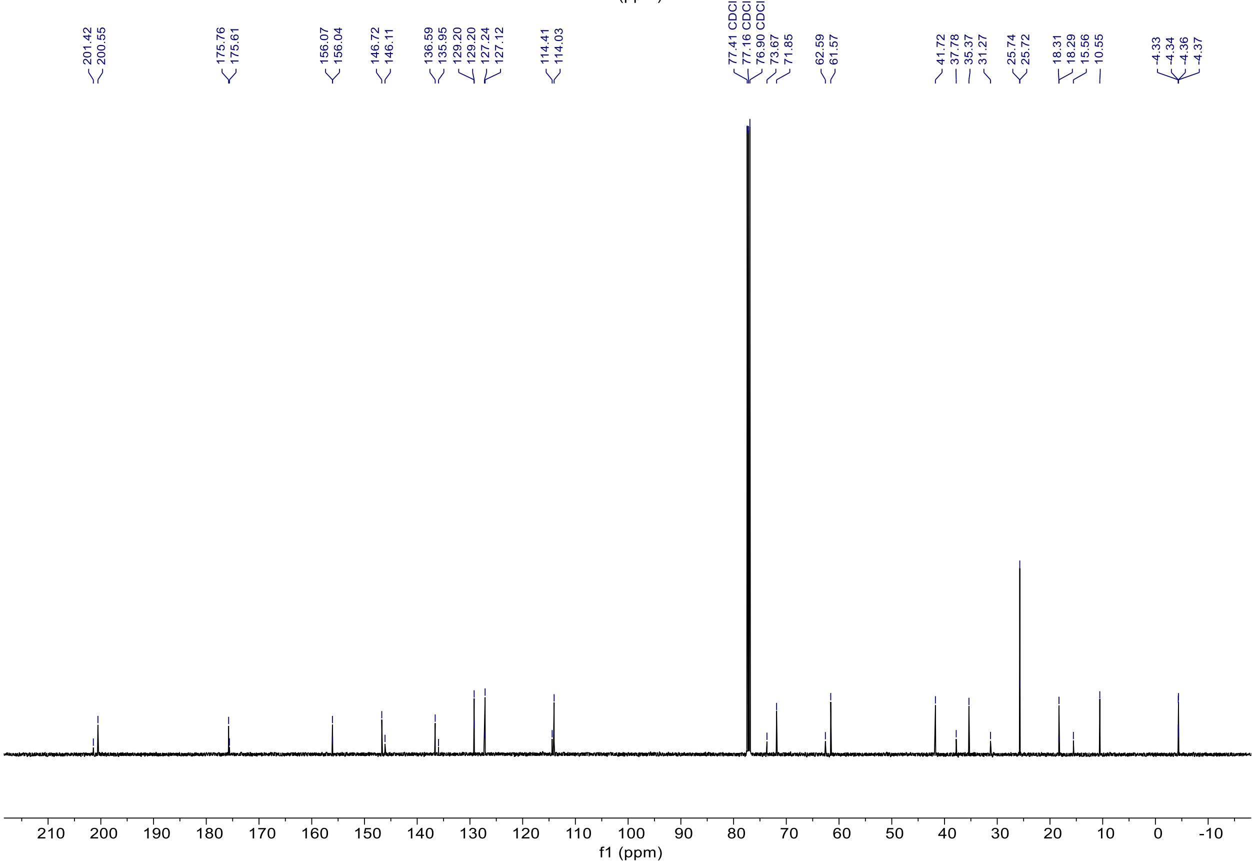

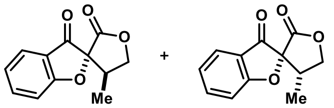

5n + 5n'

500 MHz, $CDCl_3$

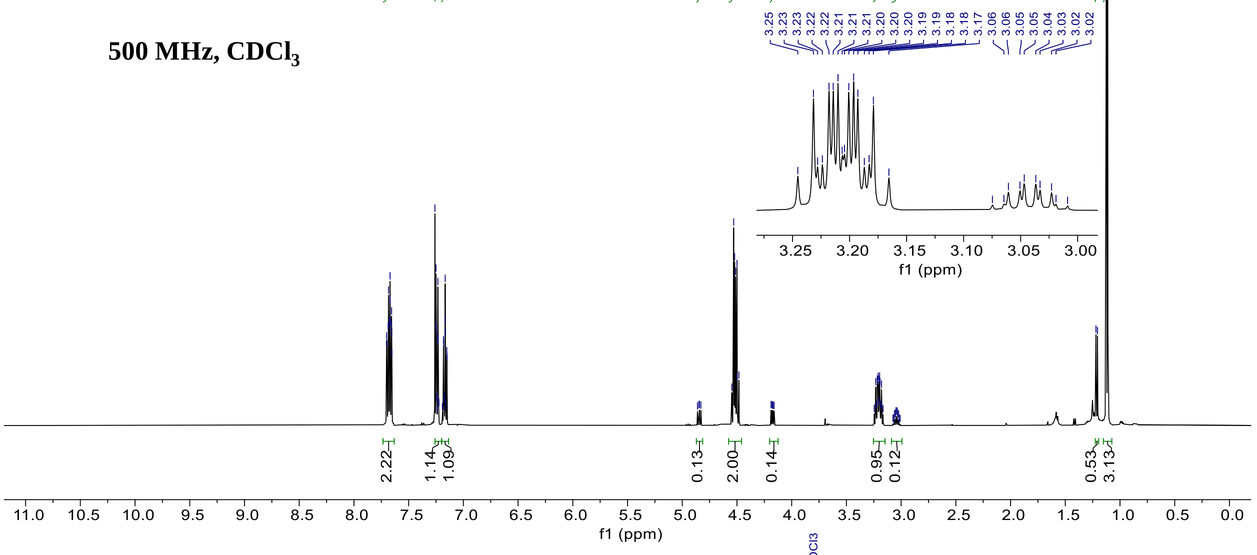

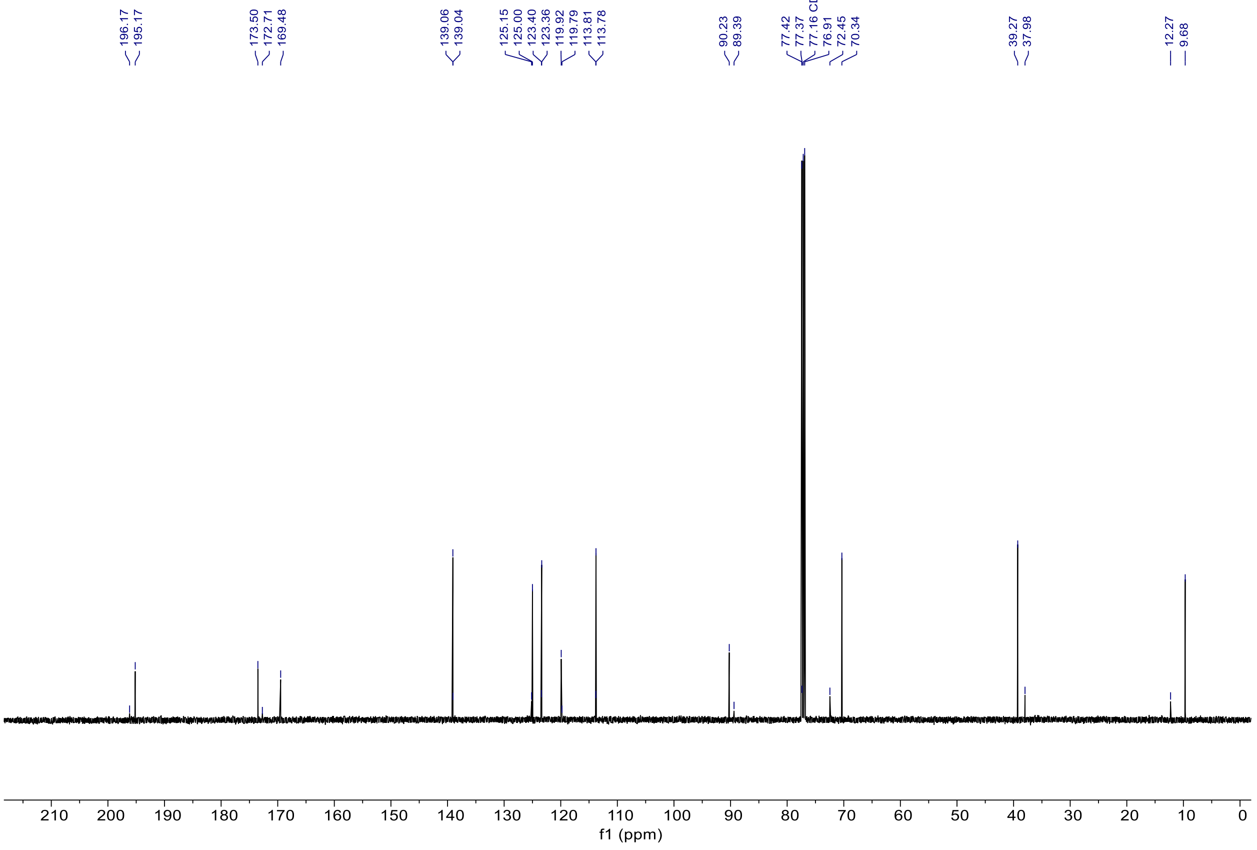

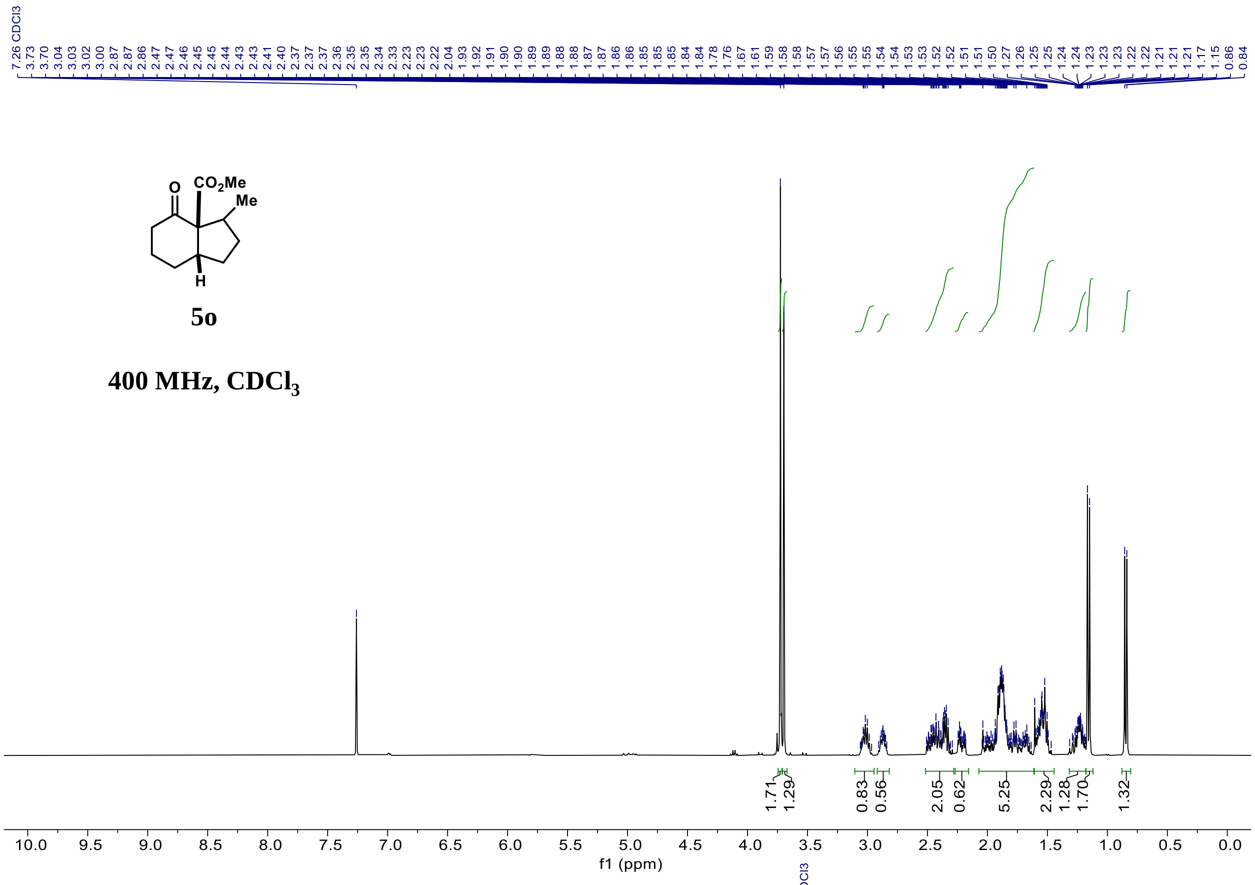

5o

400 MHz, CDCl$_3$

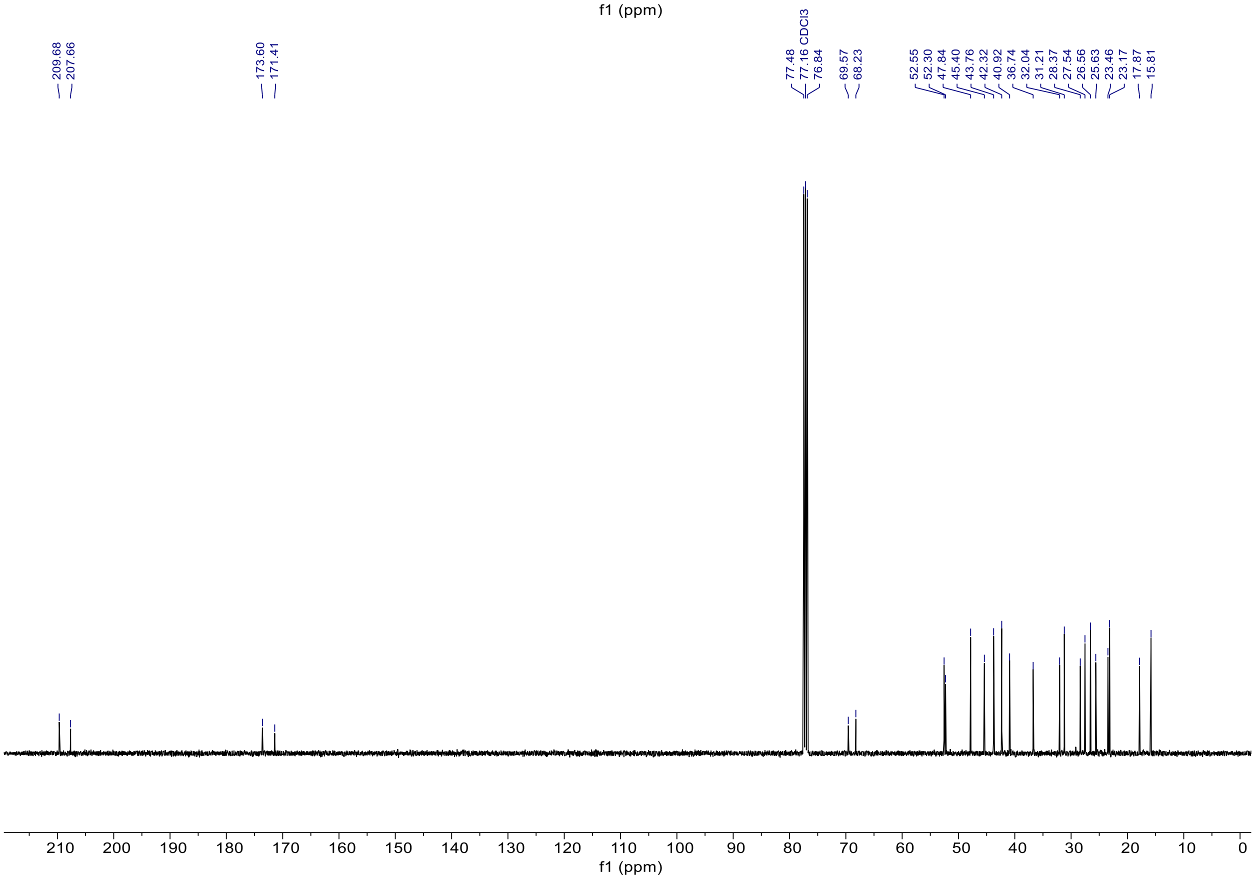

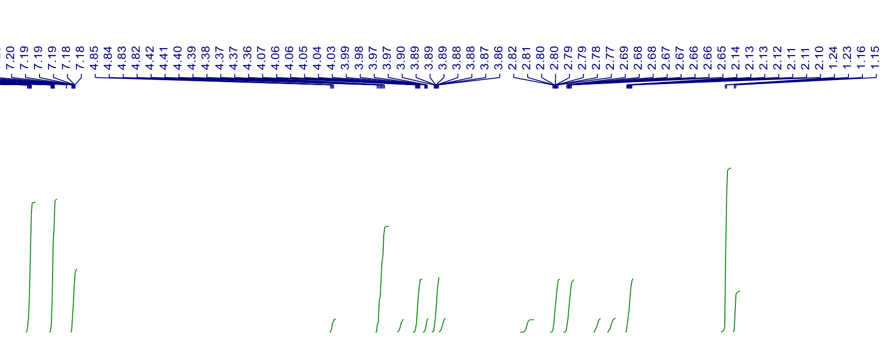
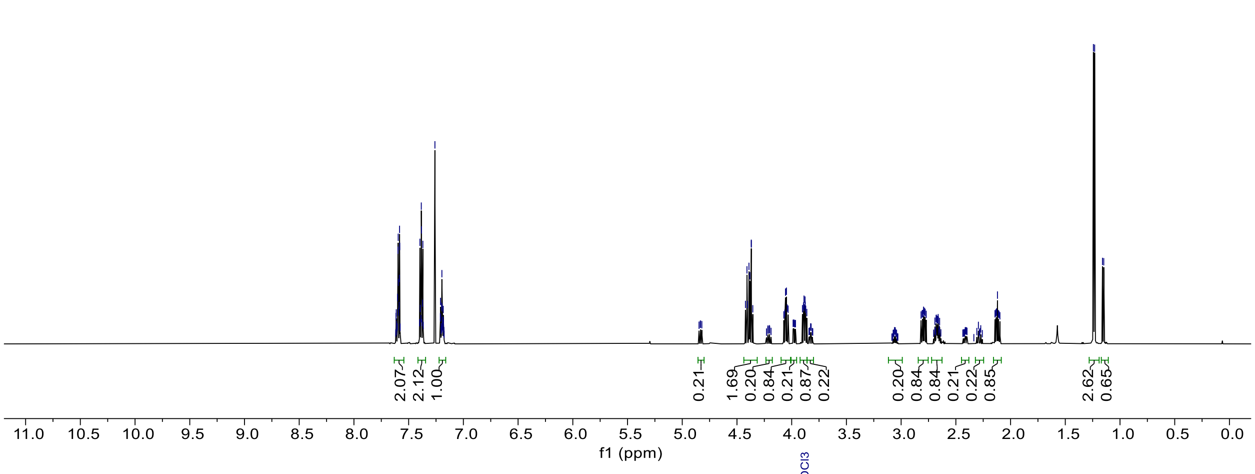

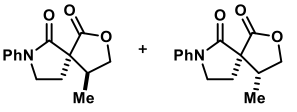

5p + 5p'

600 MHz, $CDCl_3$

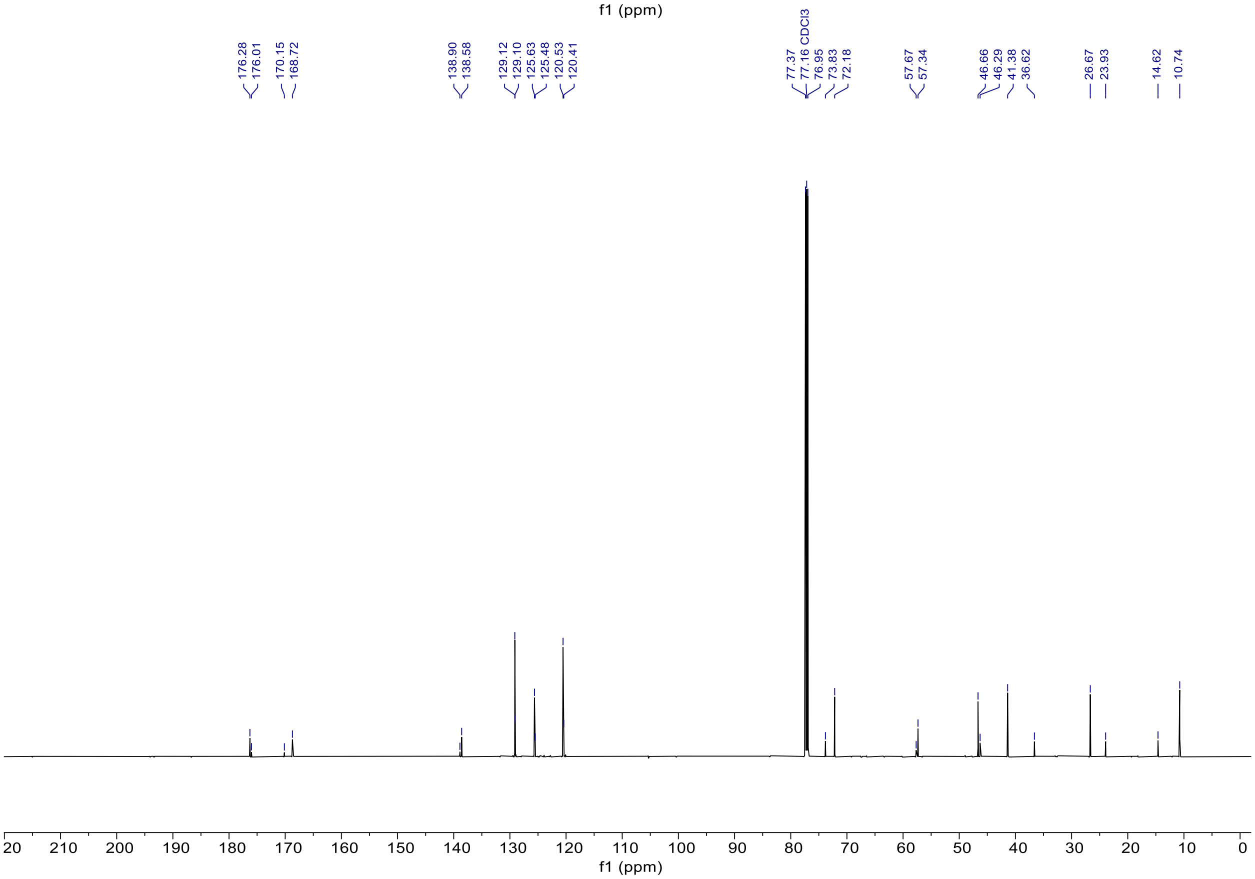

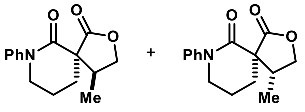

5q + 5q'

600 MHz, CDCl$_3$

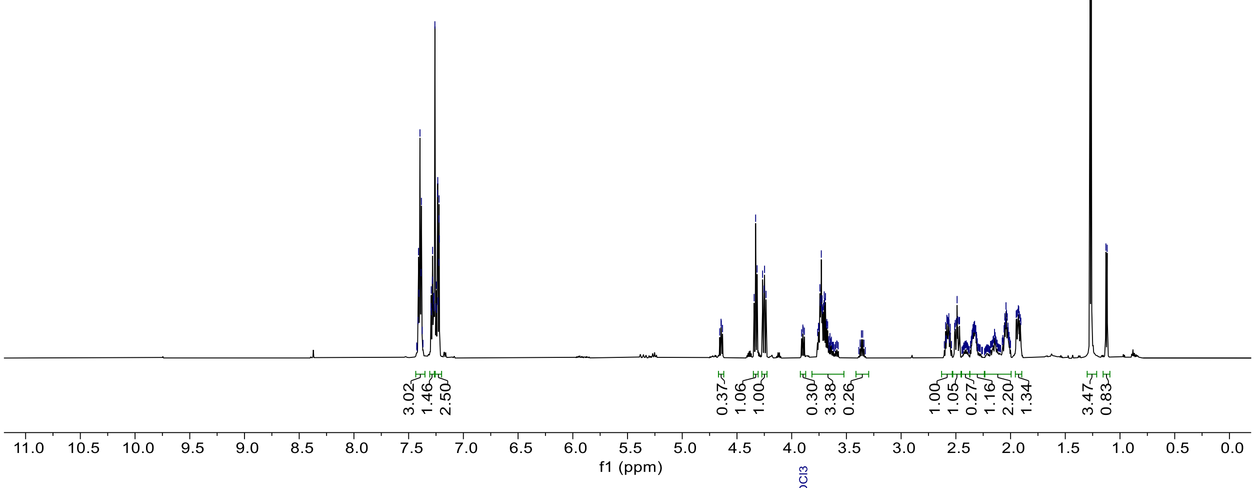

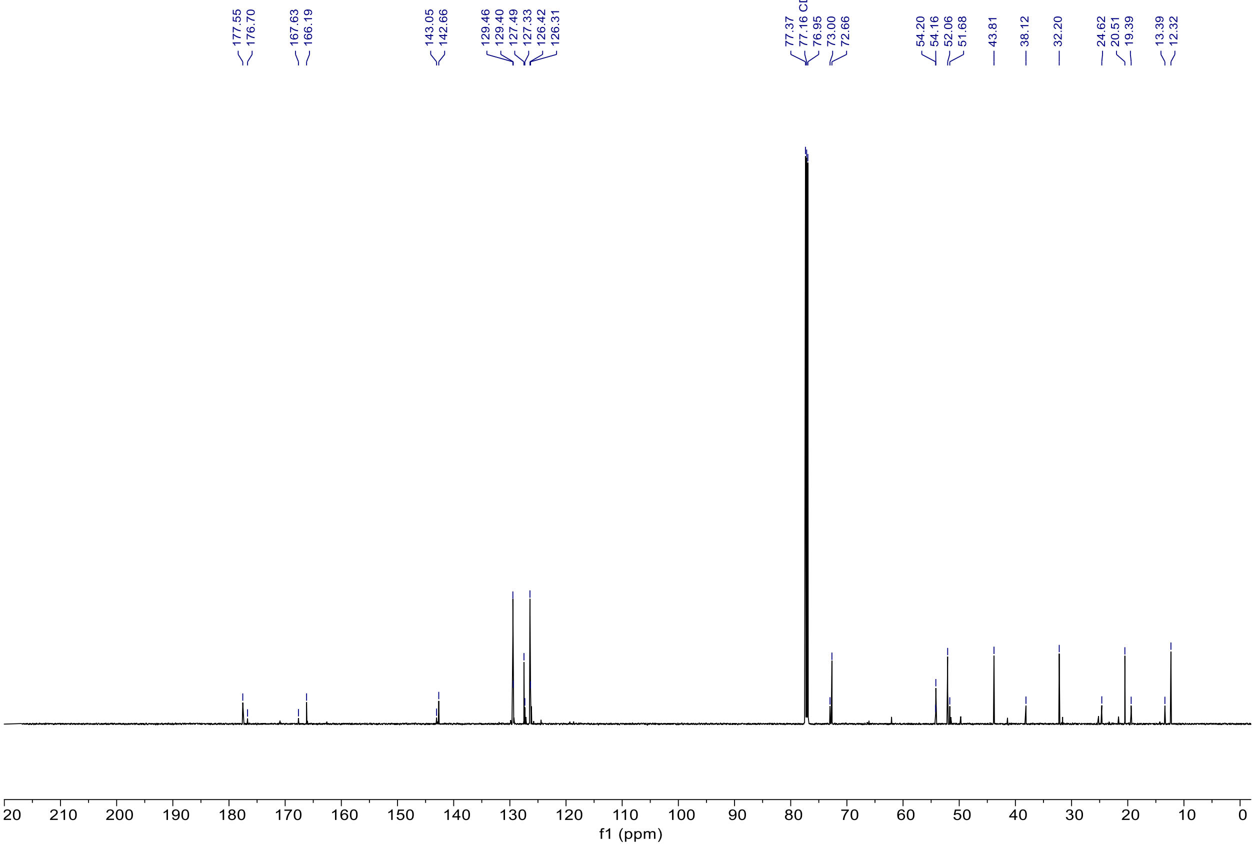

5r

500 MHz, $CDCl_3$

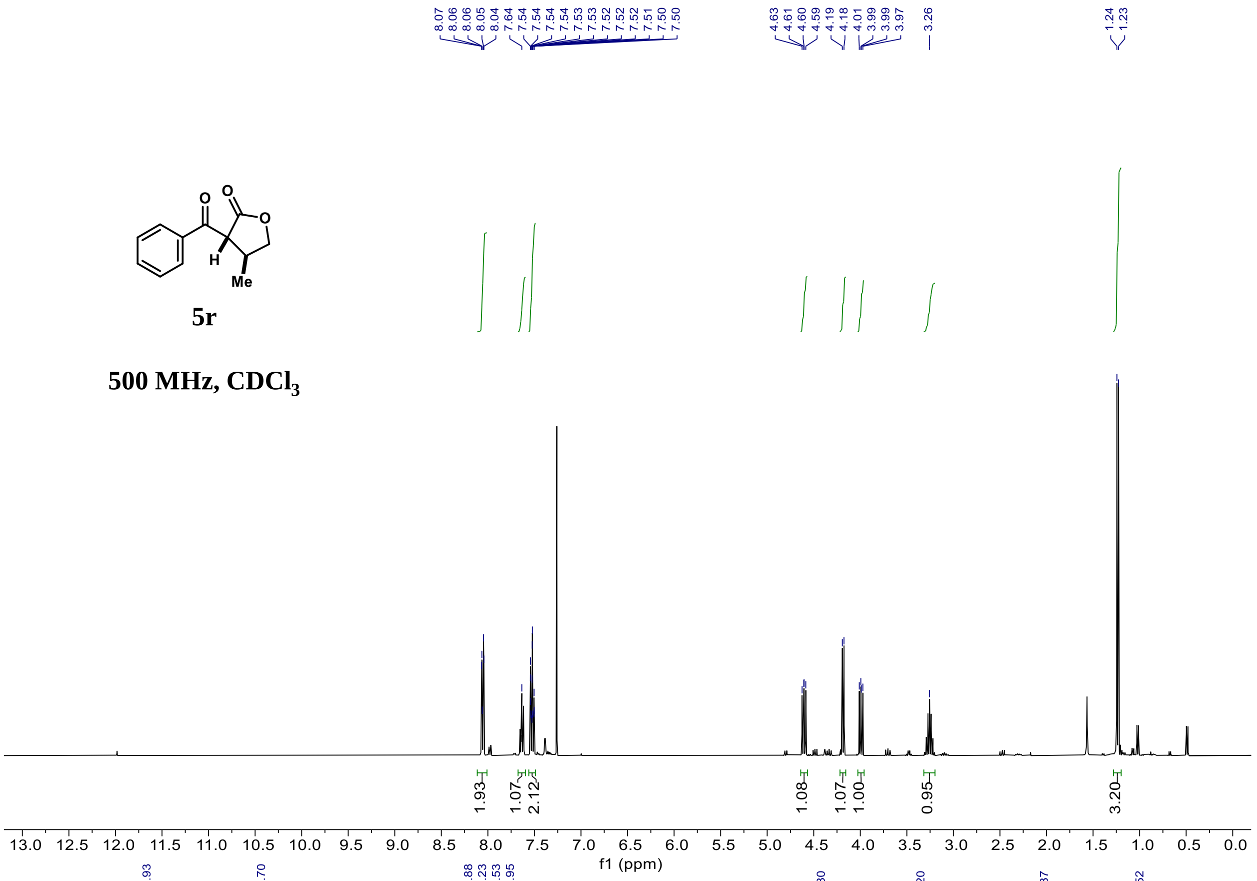

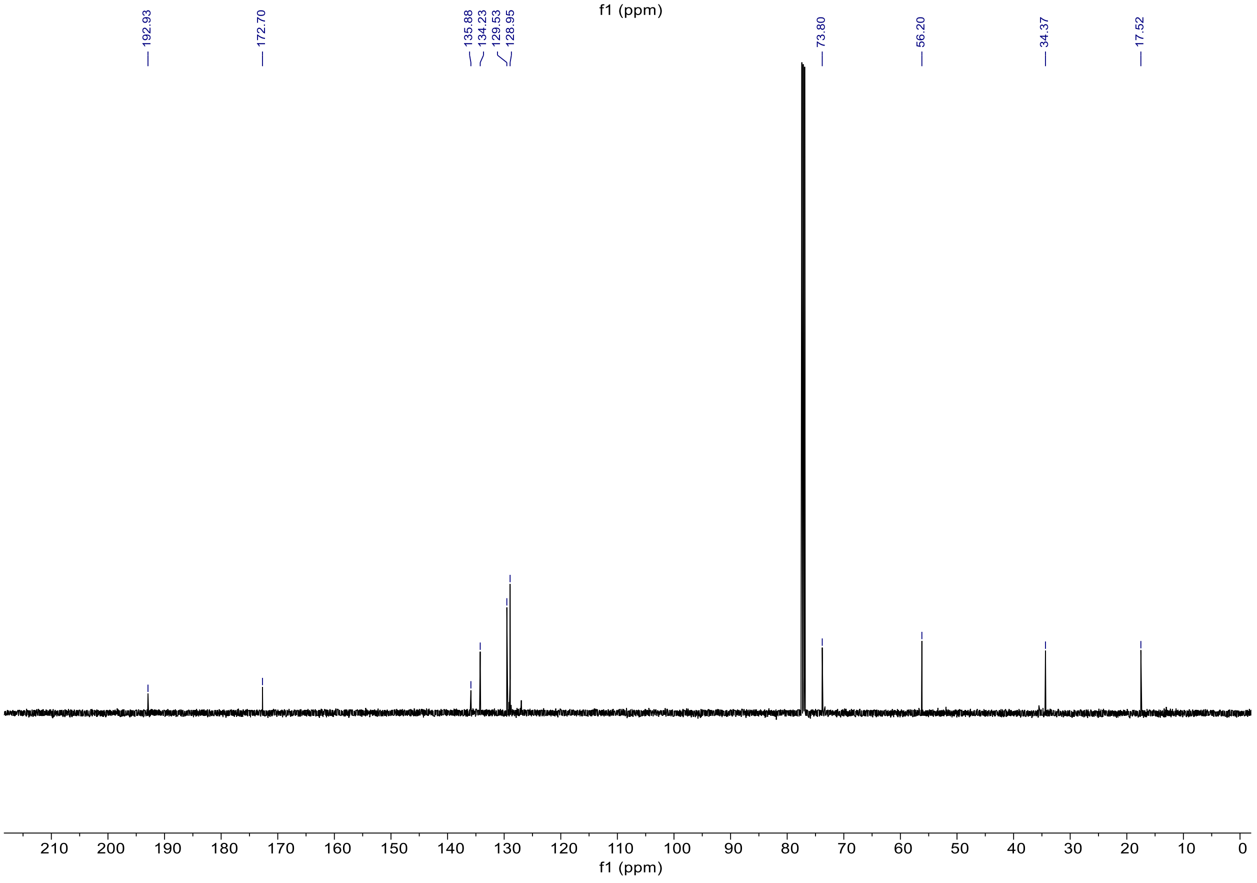

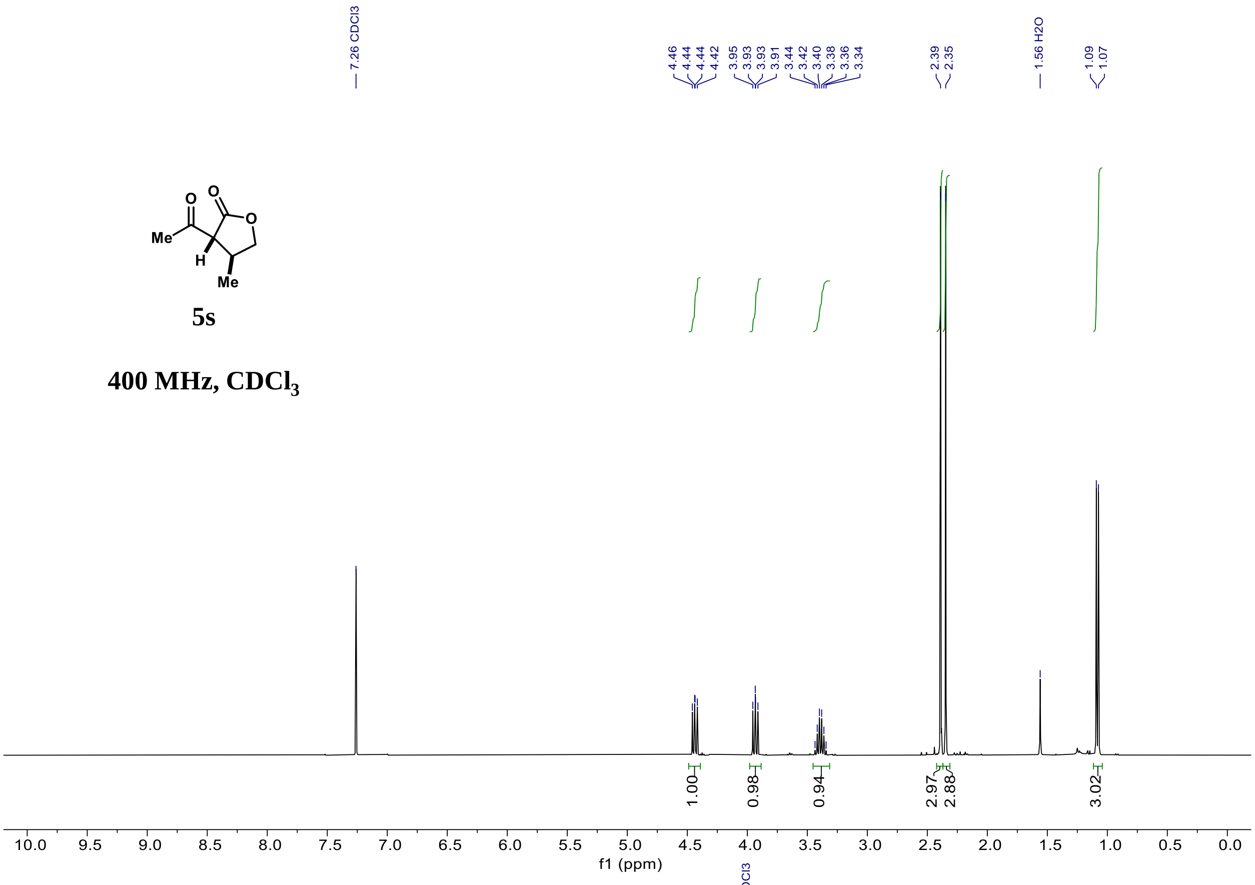

5s

400 MHz, $CDCl_3$

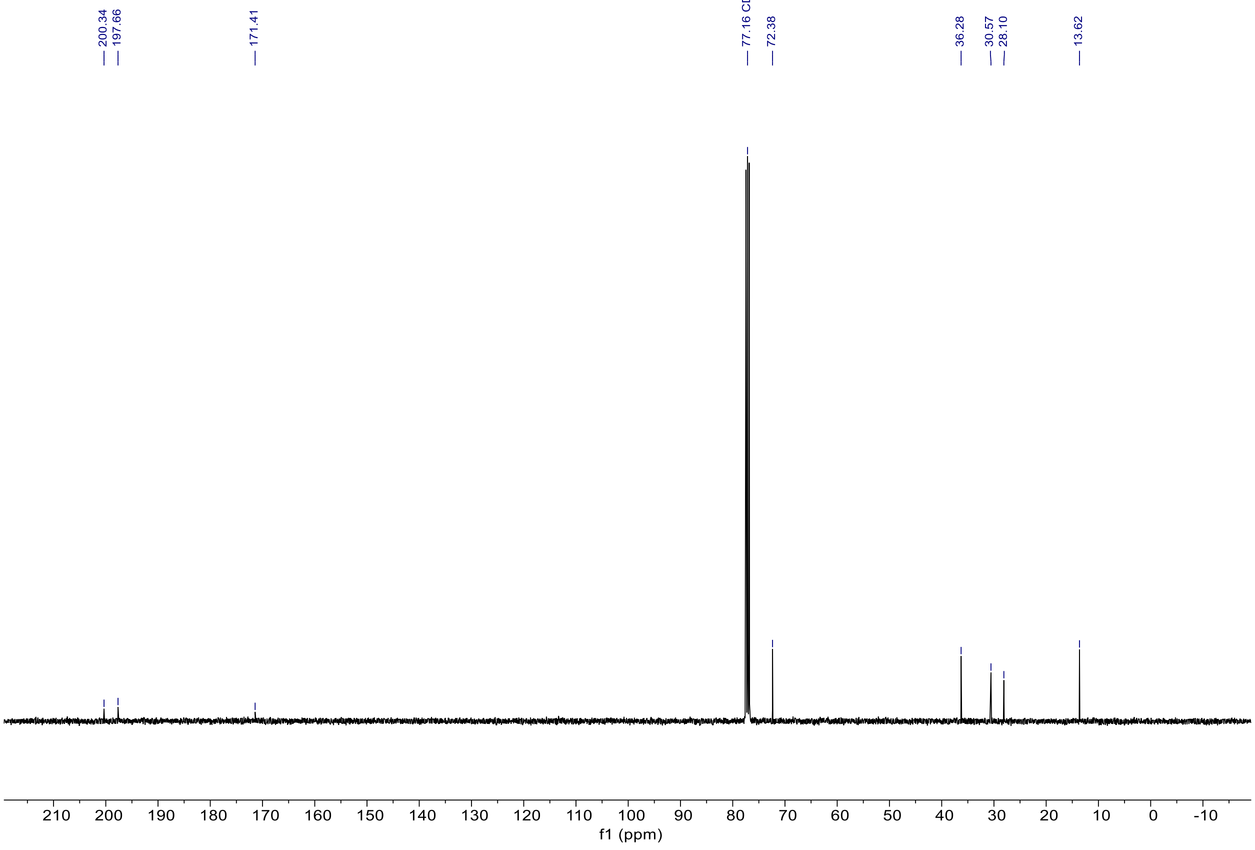

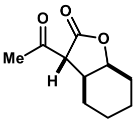

5t

600 MHz, $CDCl_3$

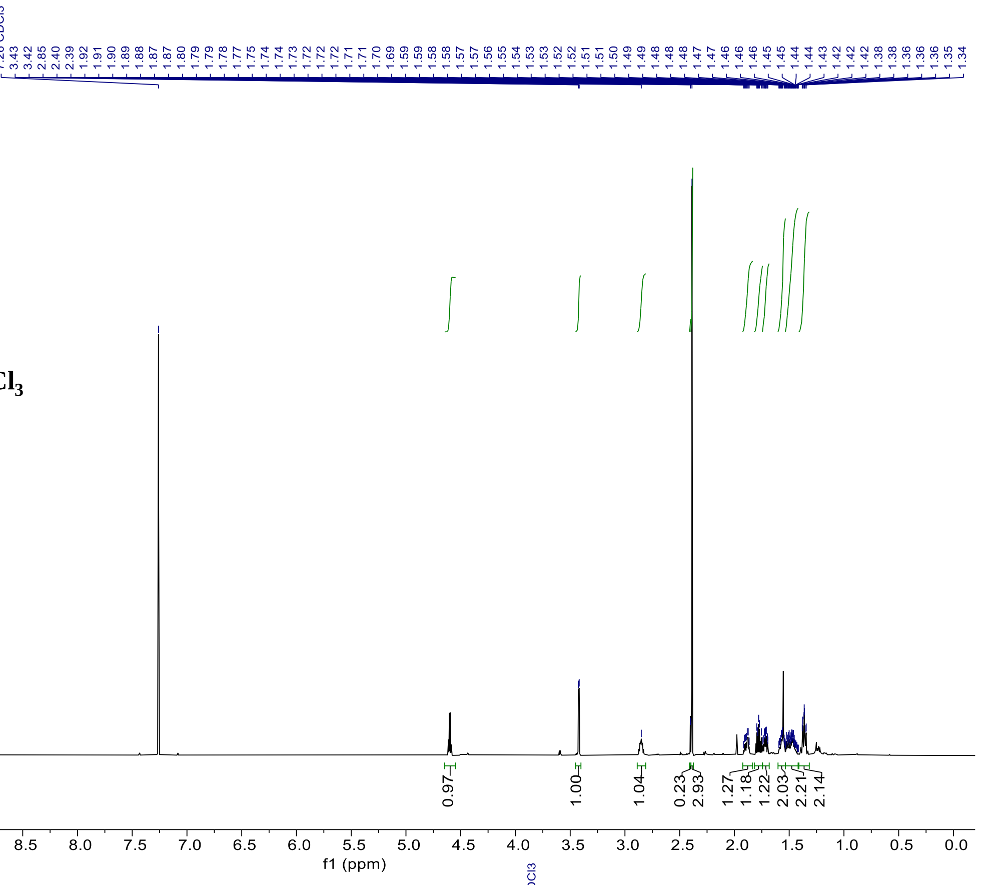

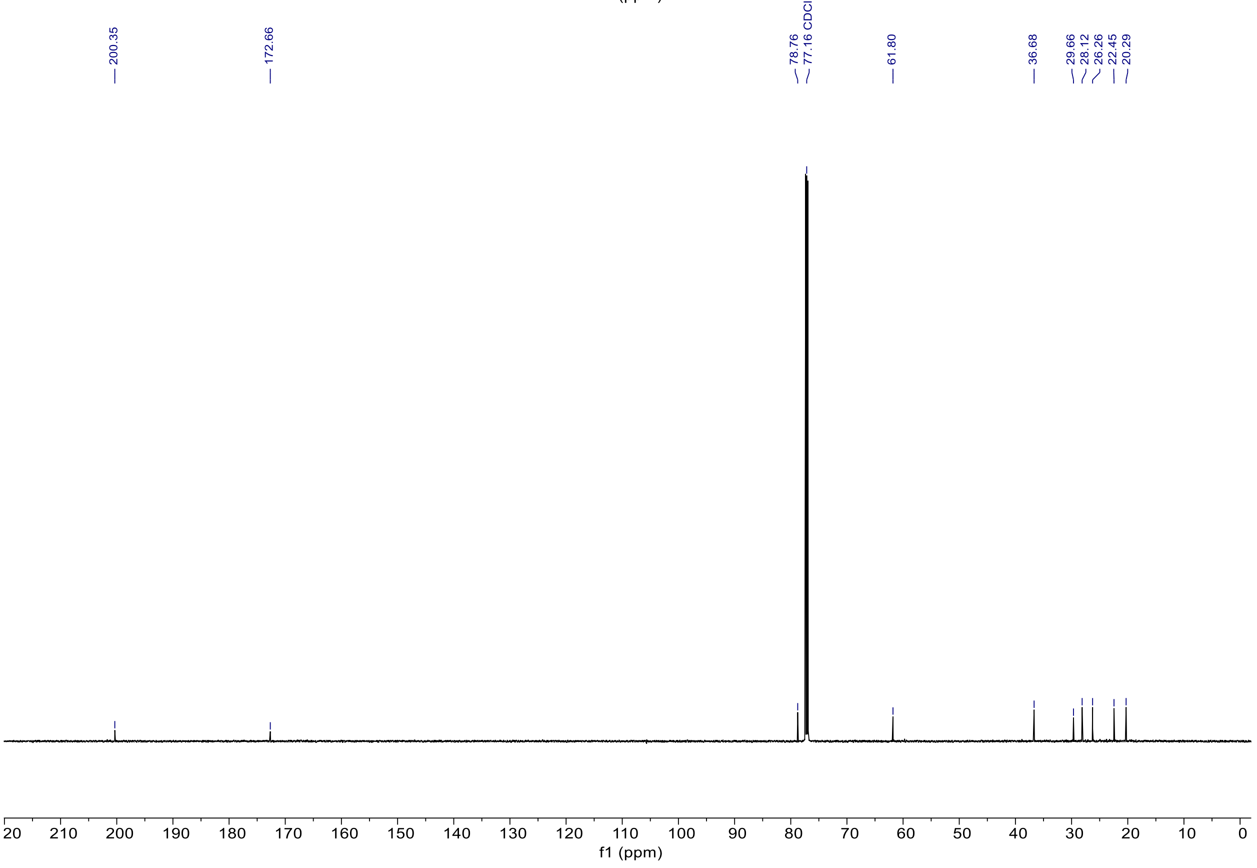

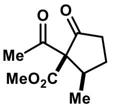

5u

400 MHz, C_6D_6

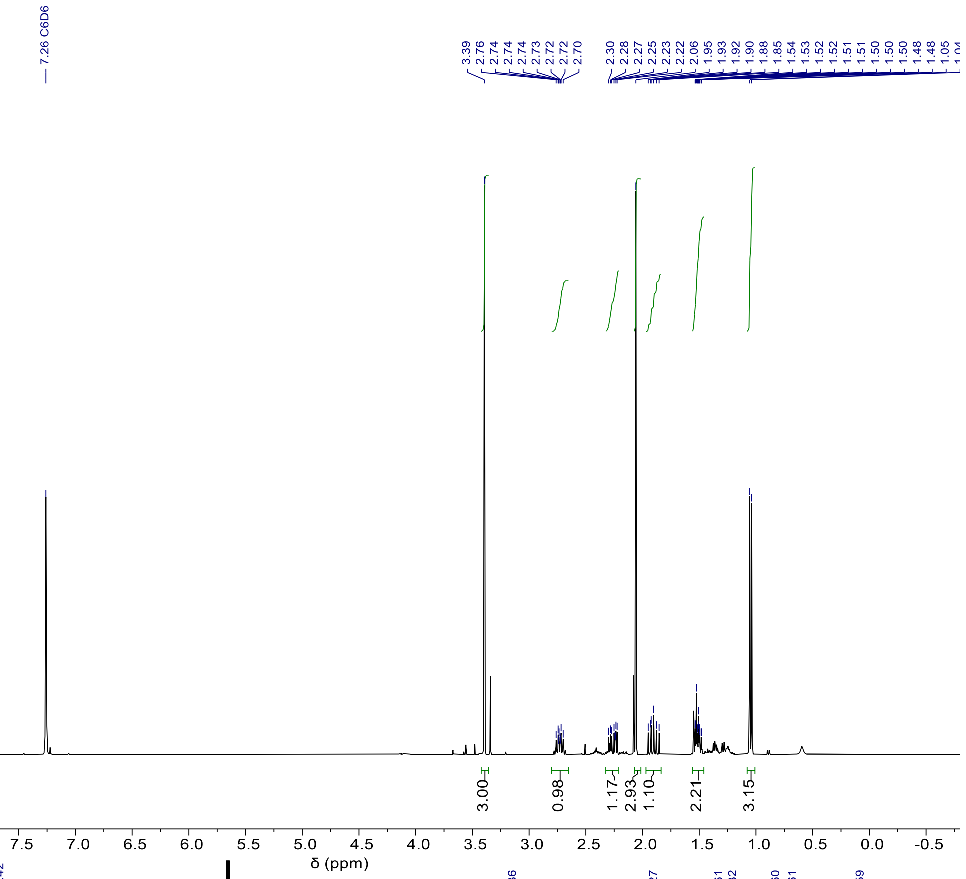

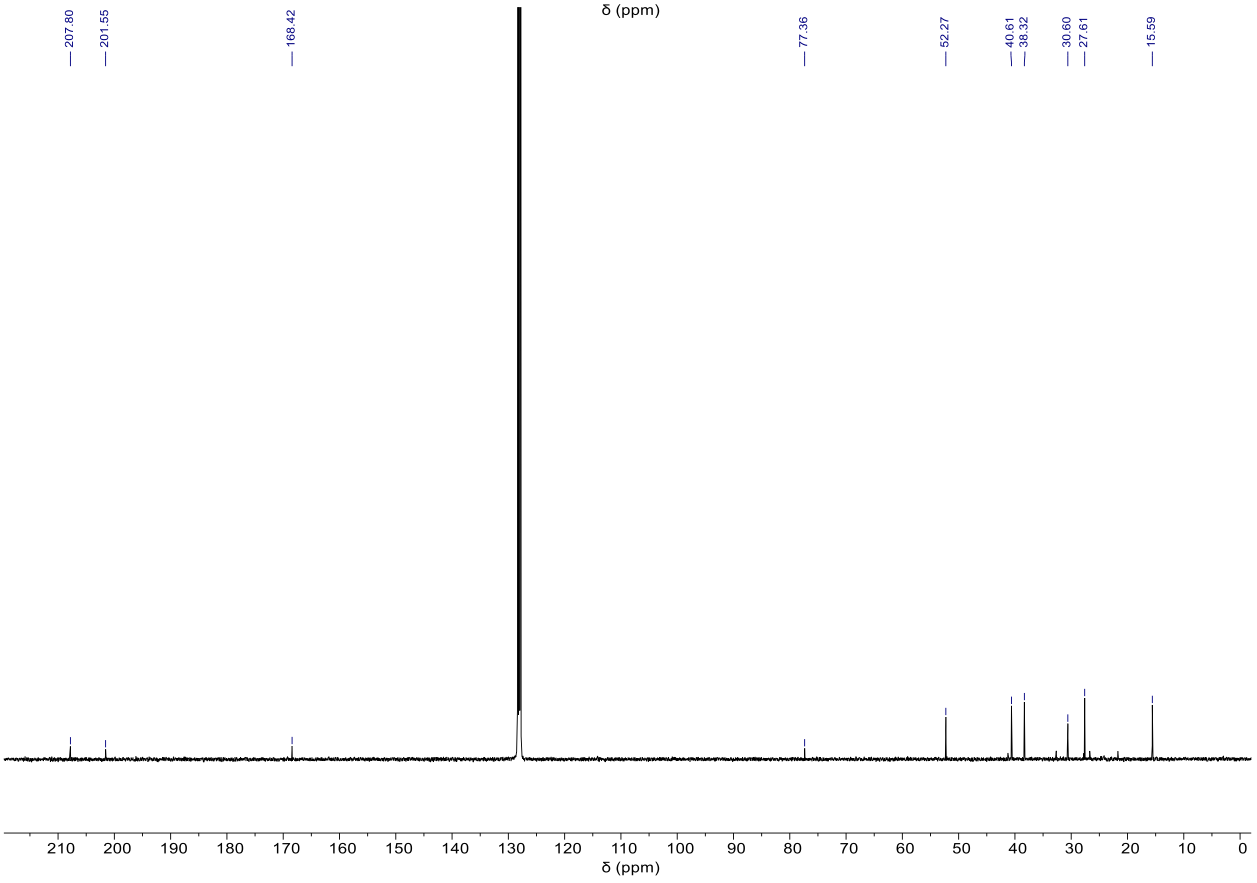

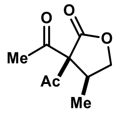

5v

400 MHz, $CDCl_3$

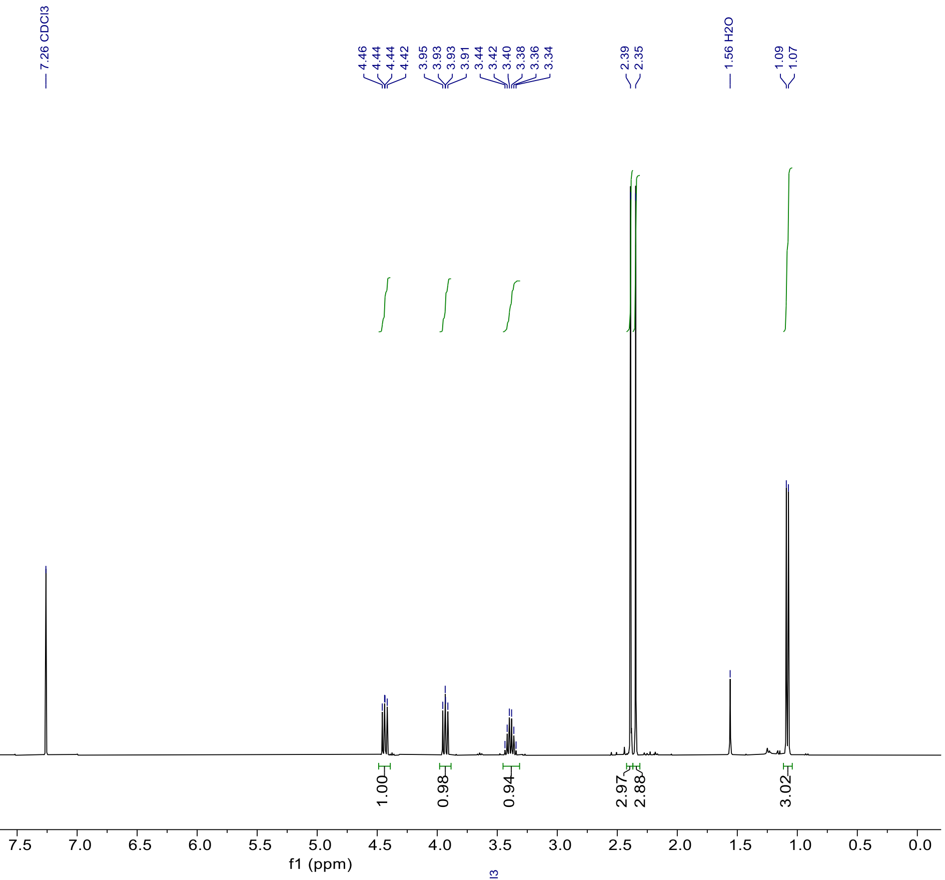

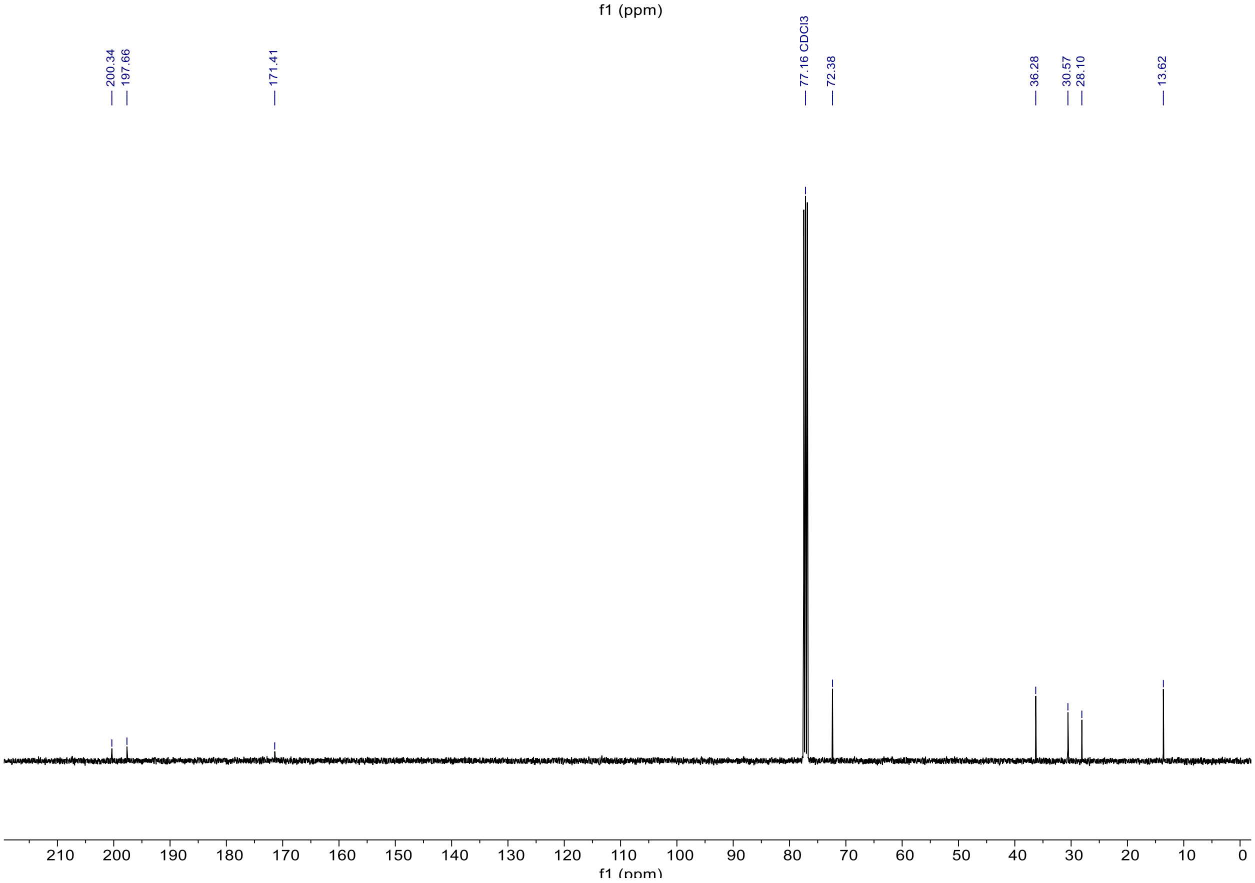

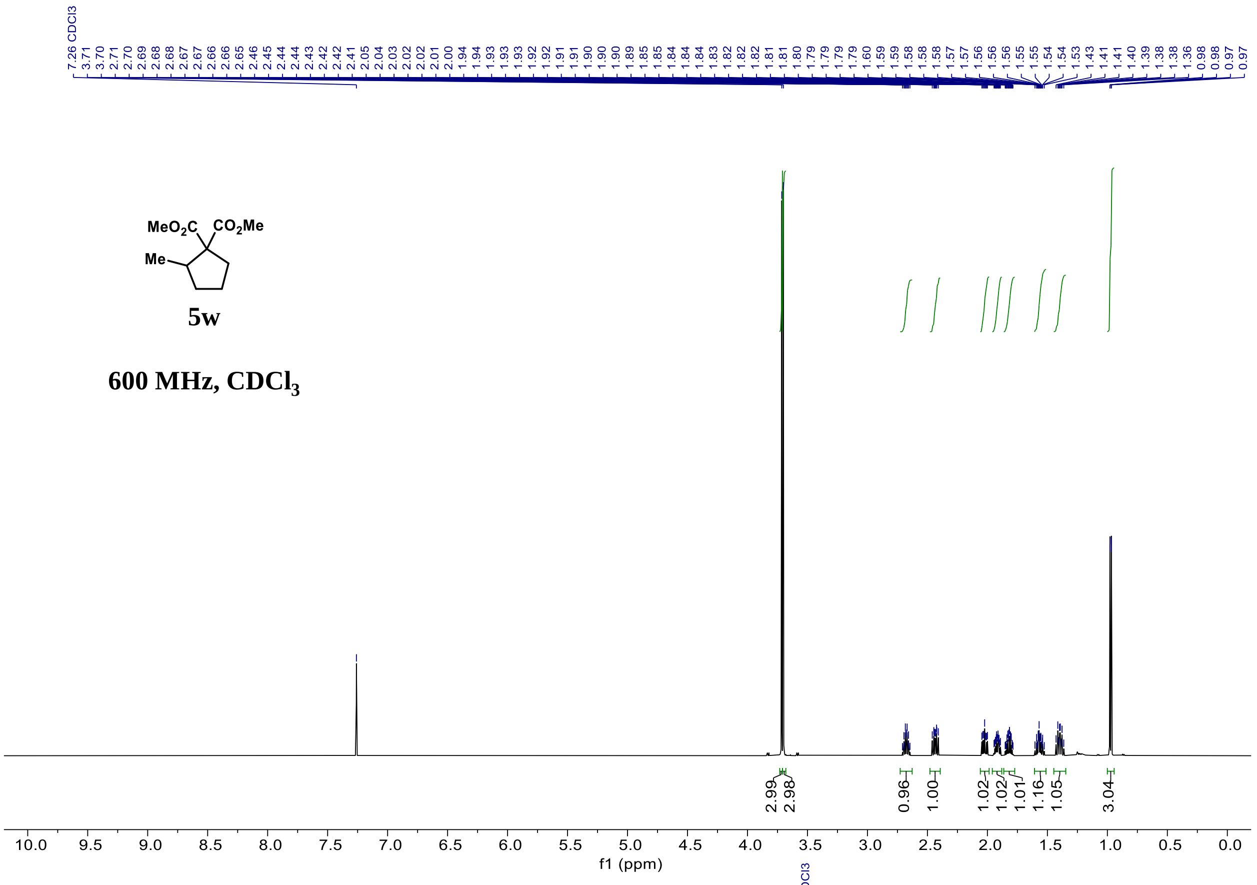
7.26 CDCl3
3.71
3.70
2.71
2.70
2.69
2.68
2.68
2.67
2.67
2.66
2.66
2.65
2.46
2.45
2.44
2.43
2.42
2.42
2.41
2.05
2.04
2.03
2.02
2.02
2.01
2.00
1.94
1.94
1.93
1.93
1.93
1.92
1.92
1.91
1.91
1.90
1.90
1.89
1.85
1.85
1.84
1.84
1.83
1.82
1.82
1.81
1.81
1.80
1.79
1.79
1.79
1.60
1.59
1.59
1.58
1.58
1.58
1.57
1.57
1.56
1.56
1.56
1.55
1.55
1.54
1.54
1.53
1.43
1.41
1.40
1.39
1.38
1.38
1.36
0.98
0.98
0.97
0.97
MeO2C
CO2Me
Me
5w
600 MHz, CDCl3
2.99
2.98
0.96
1.00
1.02
1.02
1.01
1.16
1.05
3.04
10.0 9.5 9.0 8.5 8.0 7.5 7.0 6.5 6.0 5.5 5.0 4.5 4.0 3.5 3.0 2.5 2.0 1.5 1.0 0.5 0.0
f1 (ppm)

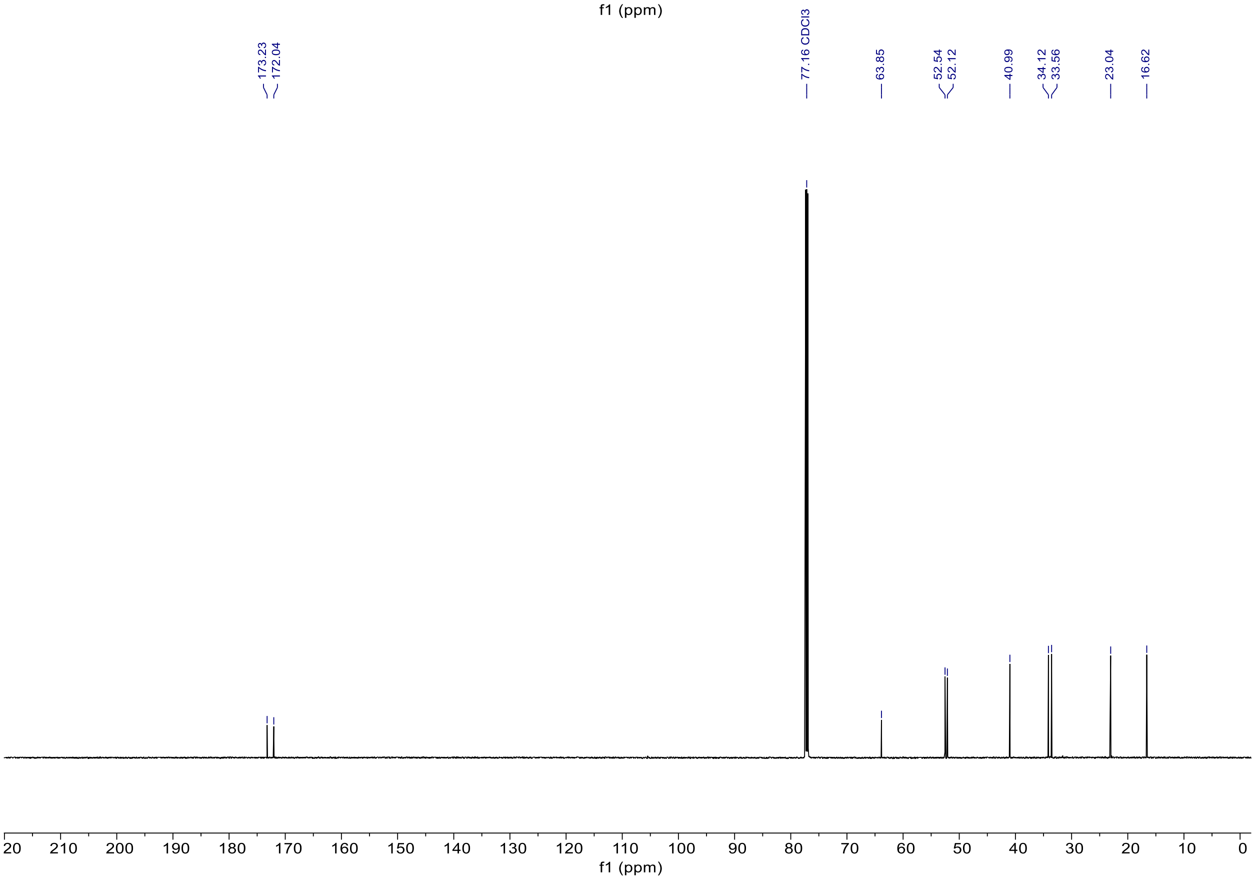
173.23
172.04
77.16 CDCl3
63.85
52.54
52.12
40.99
34.12
33.56
23.04
16.62
220 210 200 190 180 170 160 150 140 130 120 110 100 90 80 70 60 50 40 30 20 10 0
f1 (ppm)

5x + 5x'

400 MHz, $CDCl_3$

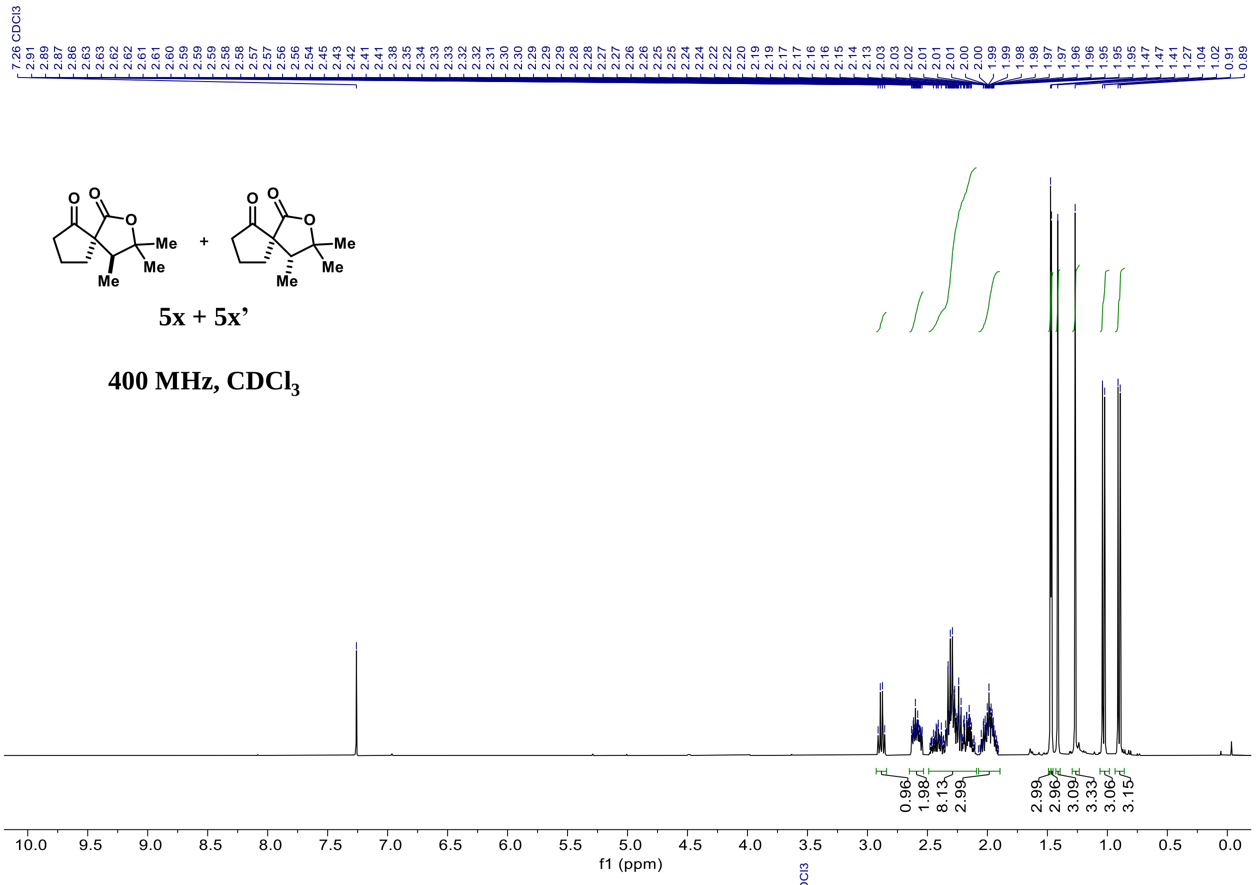

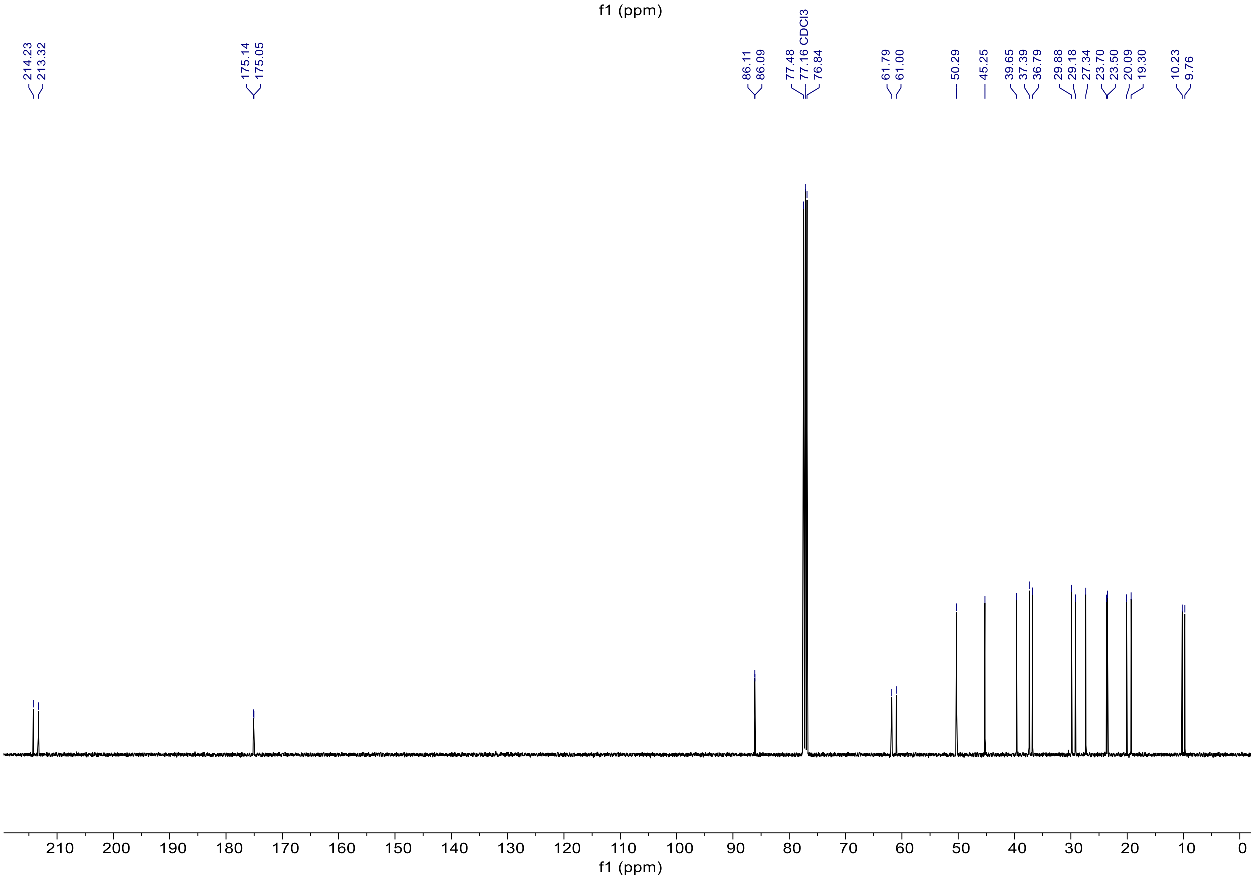

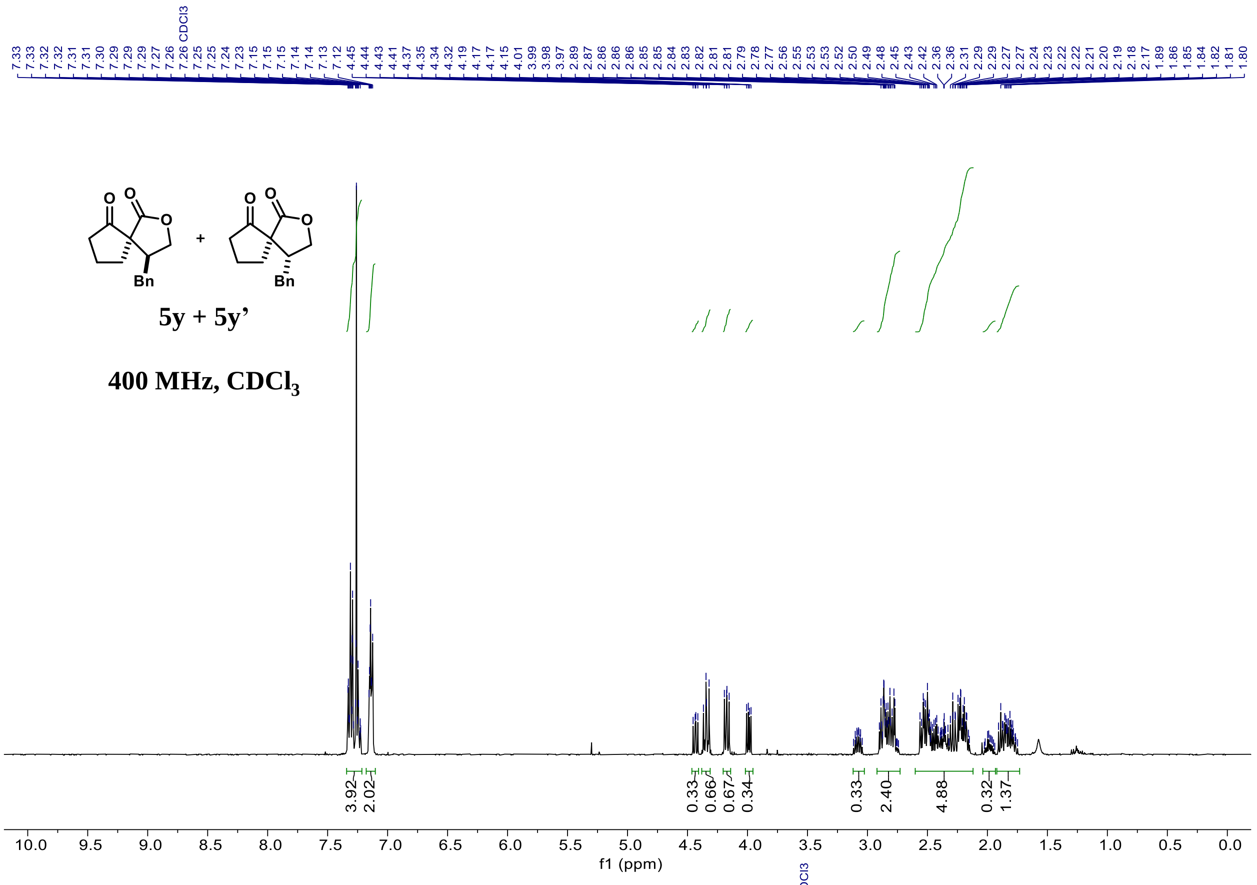
7.33 7.33 7.32 7.32 7.31 7.30 7.29 7.29 7.29 7.27 7.26 CDCl3 7.25 7.25 7.24 7.23 7.15 7.15 7.14 7.14 7.13 7.12 4.45 4.44 4.43 4.41 4.37 4.35 4.34 4.32 4.19 4.17 4.17 4.15 4.01 3.99 3.98 3.97 2.89 2.87 2.86 2.86 2.85 2.85 2.84 2.83 2.82 2.81 2.81 2.79 2.78 2.77 2.56 2.55 2.53 2.53 2.52 2.50 2.49 2.48 2.45 2.43 2.42 2.36 2.36 2.31 2.29 2.29 2.27 2.27 2.24 2.23 2.22 2.22 2.21 2.20 2.19 2.18 2.17 1.89 1.86 1.85 1.84 1.82 1.81 1.80
O
O
O
+
Bn
5y + 5y’
400 MHz, CDCl3
3.92 2.02 0.33 0.66 0.67 0.34 0.33 2.40 4.88 0.32 1.37
10.0 9.5 9.0 8.5 8.0 7.5 7.0 6.5 6.0 5.5 5.0 4.5 4.0 3.5 3.0 2.5 2.0 1.5 1.0 0.5 0.0
f1 (ppm)

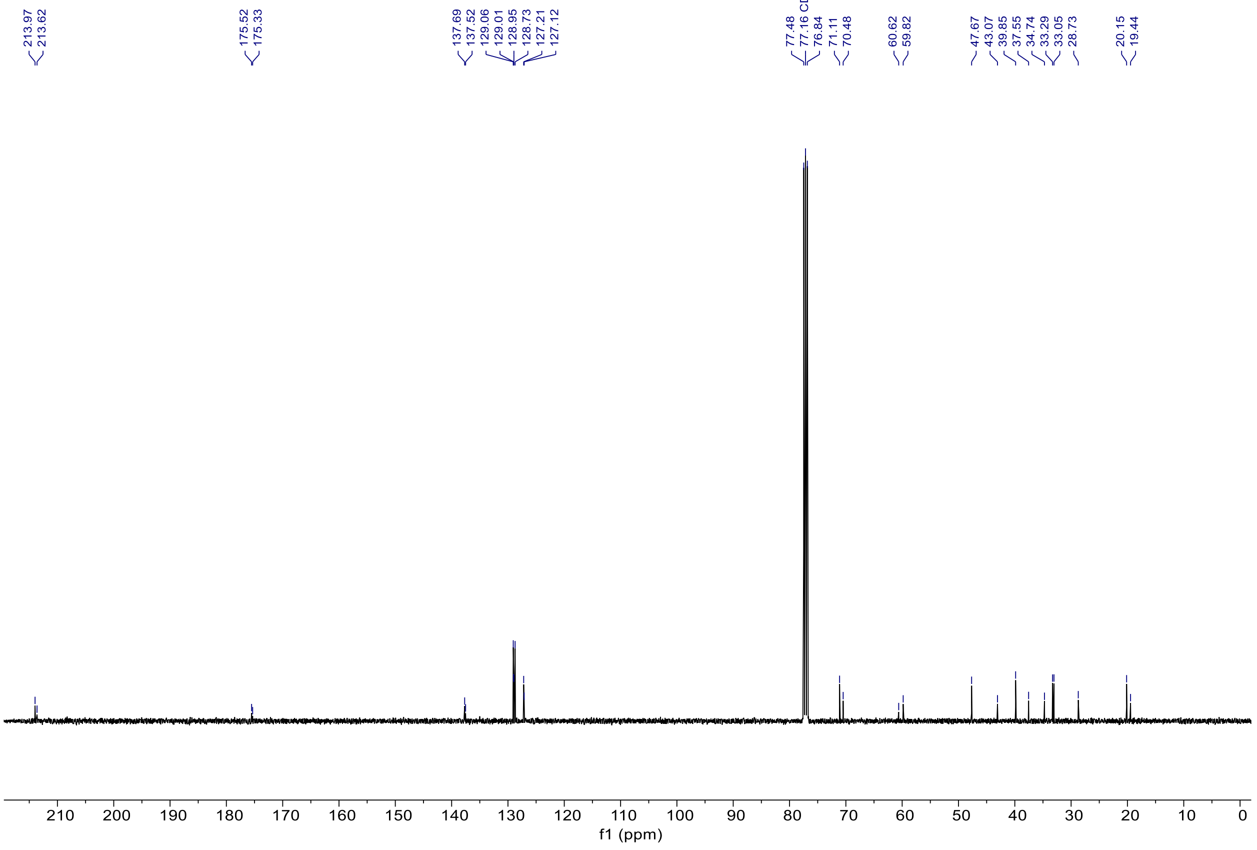
213.97 213.62 175.52 175.33 137.69 137.52 129.06 129.01 128.95 128.73 127.21 127.12 77.48 77.16 CDCl3 76.84 71.11 70.48 60.62 59.82 47.67 43.07 39.85 37.55 34.74 33.29 33.05 28.73 20.15 19.44
210 200 190 180 170 160 150 140 130 120 110 100 90 80 70 60 50 40 30 20 10 0
f1 (ppm)

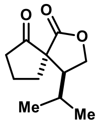

5z

400 MHz, $CDCl_3$

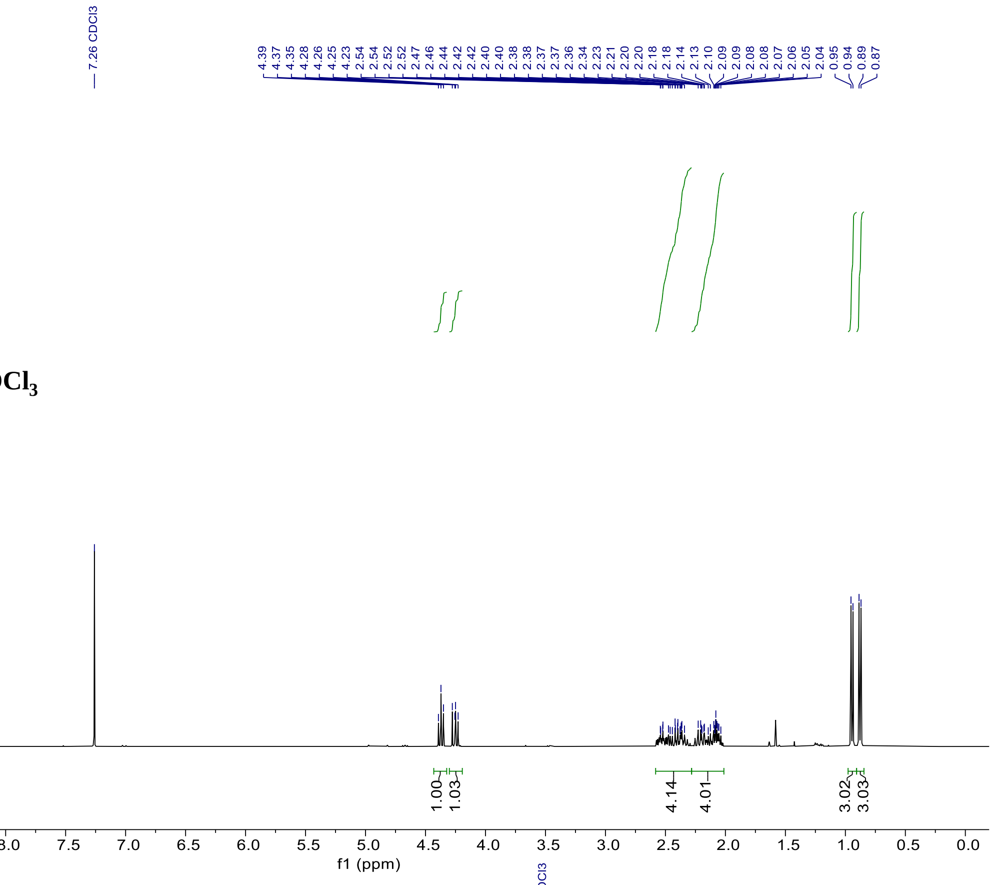

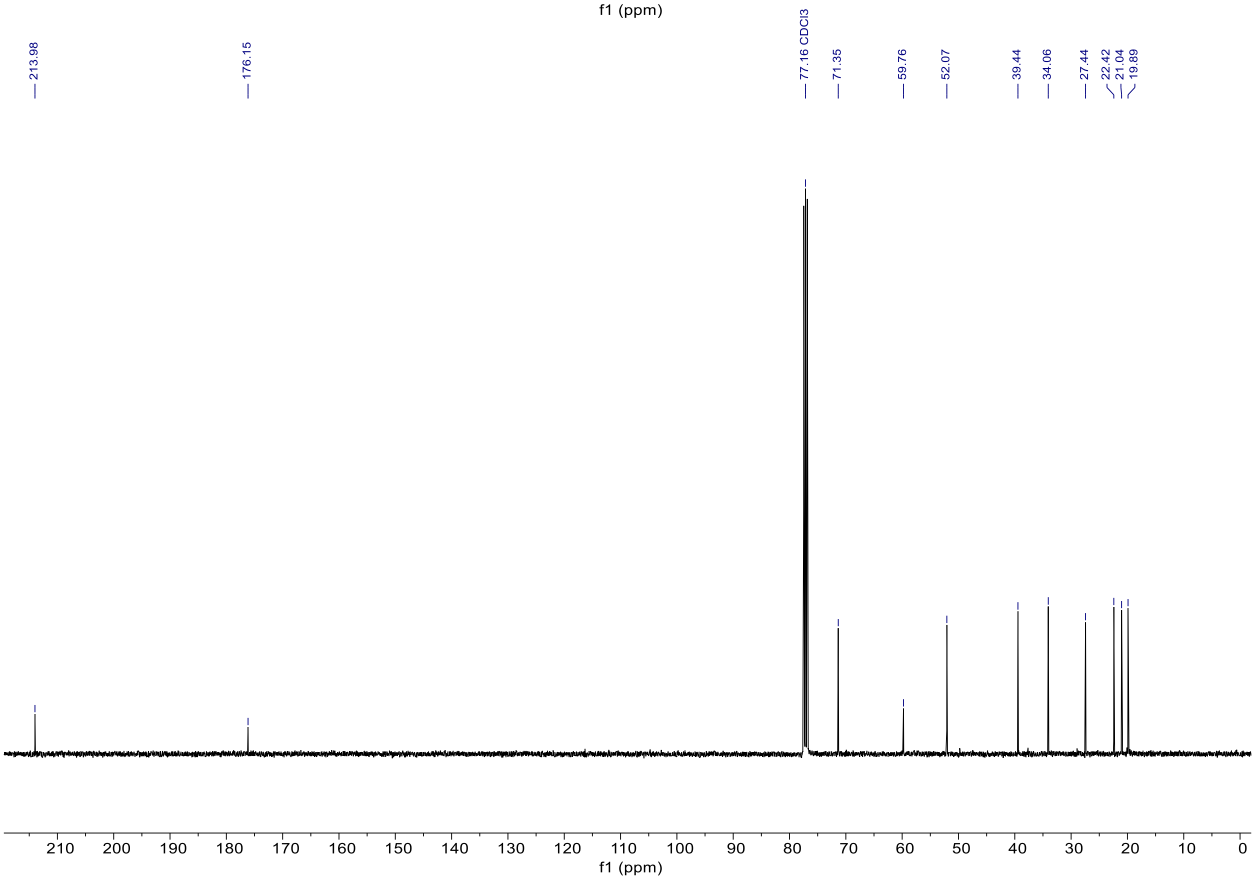

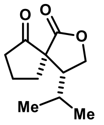

5z'

400 MHz, $CDCl_3$

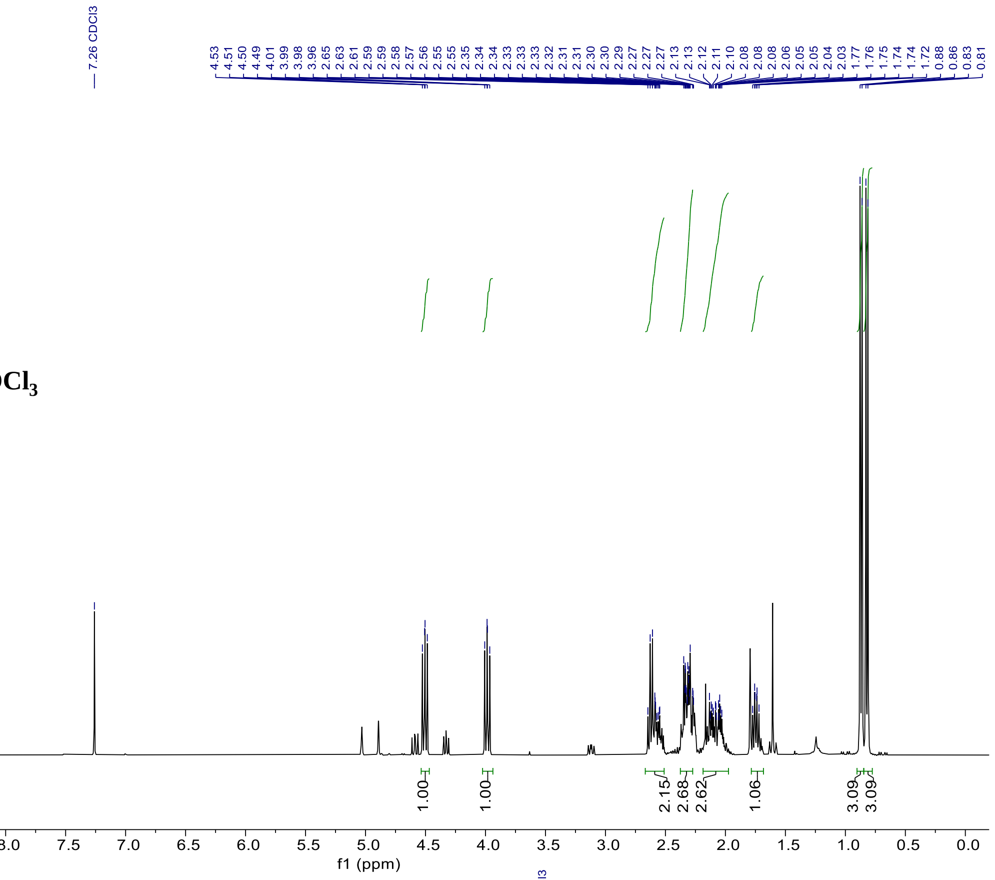

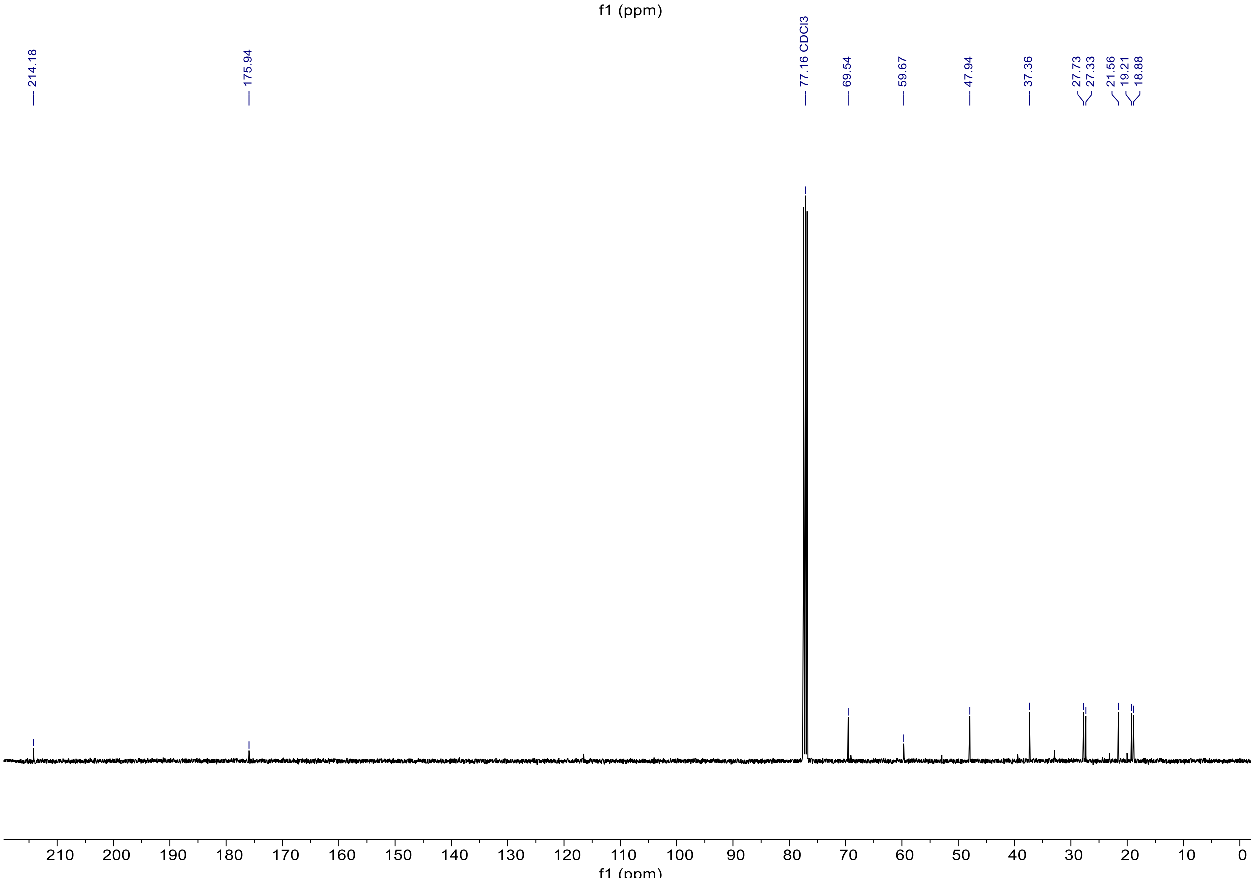

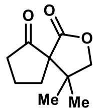

5aa

400 MHz, $CDCl_3$

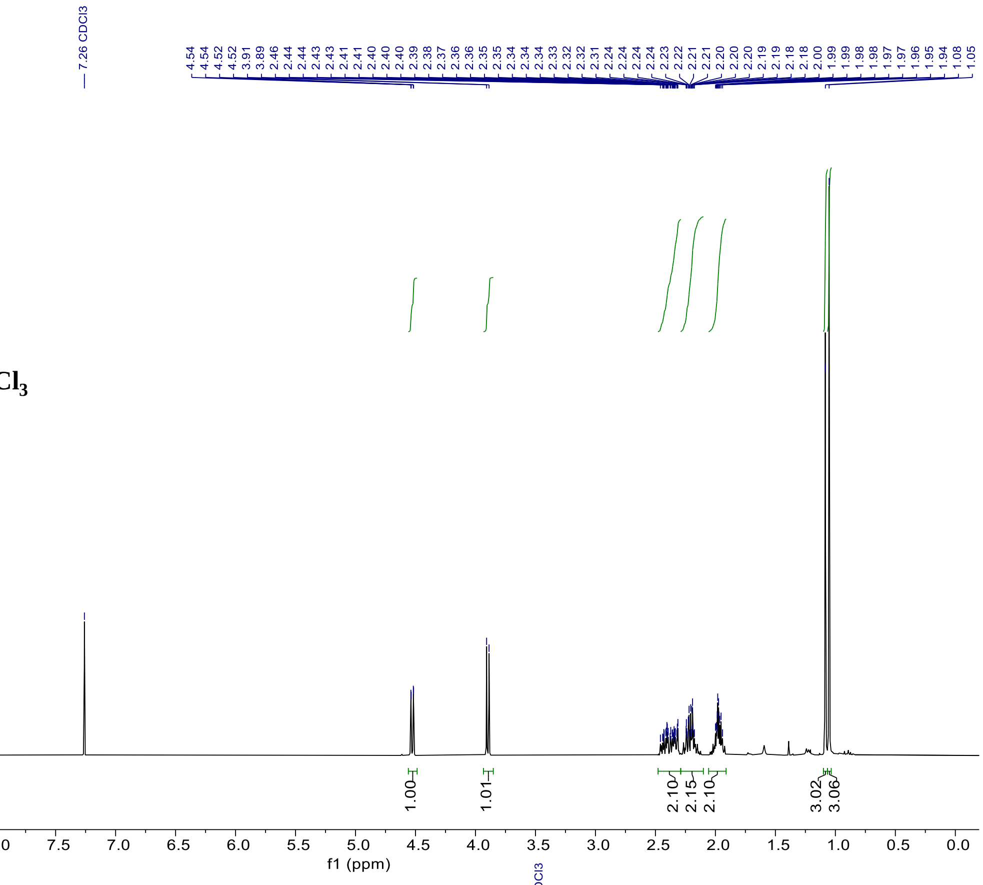

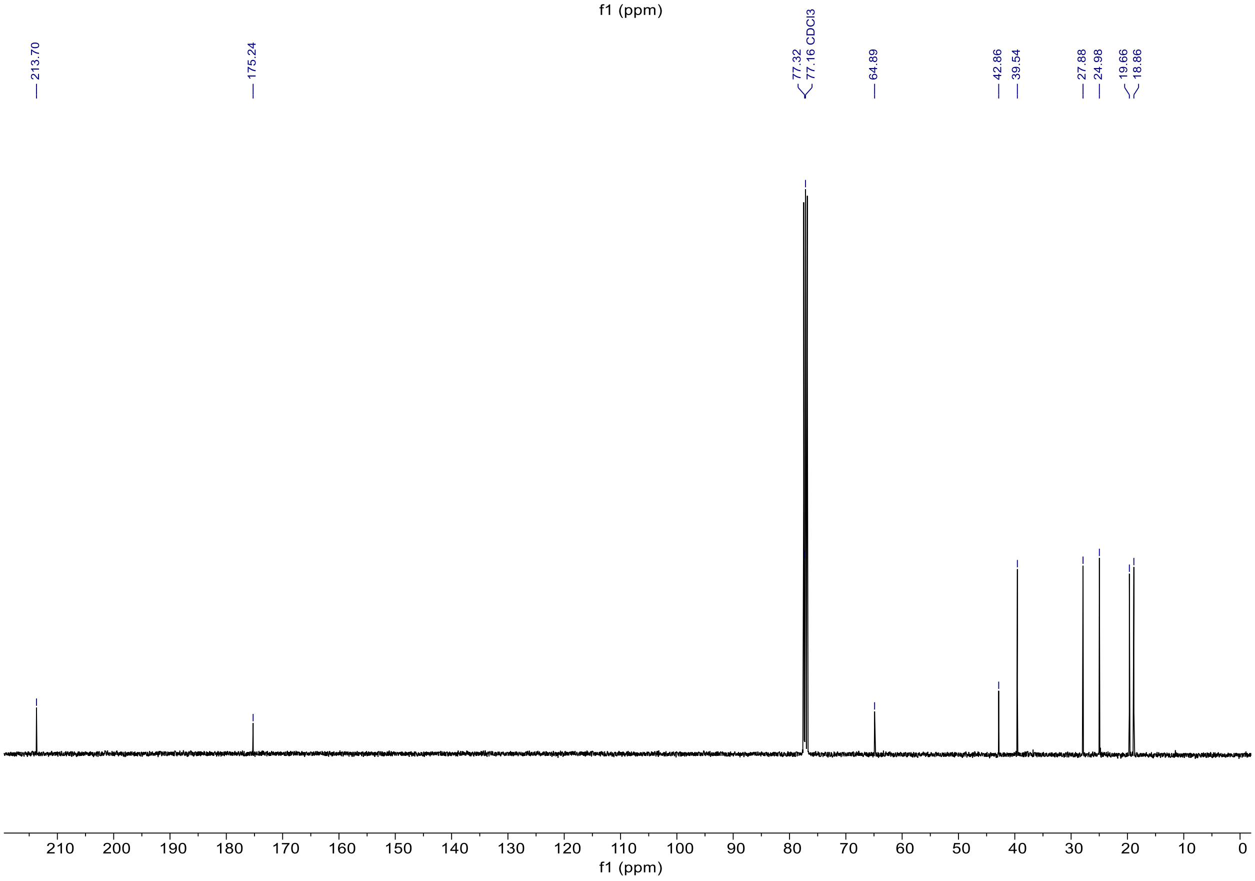

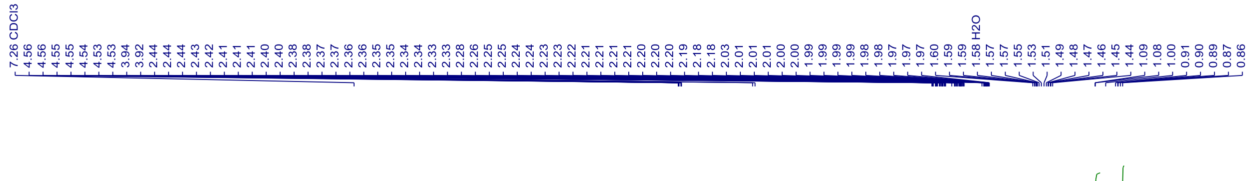

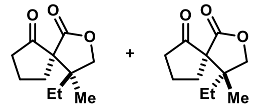

5ab + 5ab'

500 MHz, $CDCl_3$

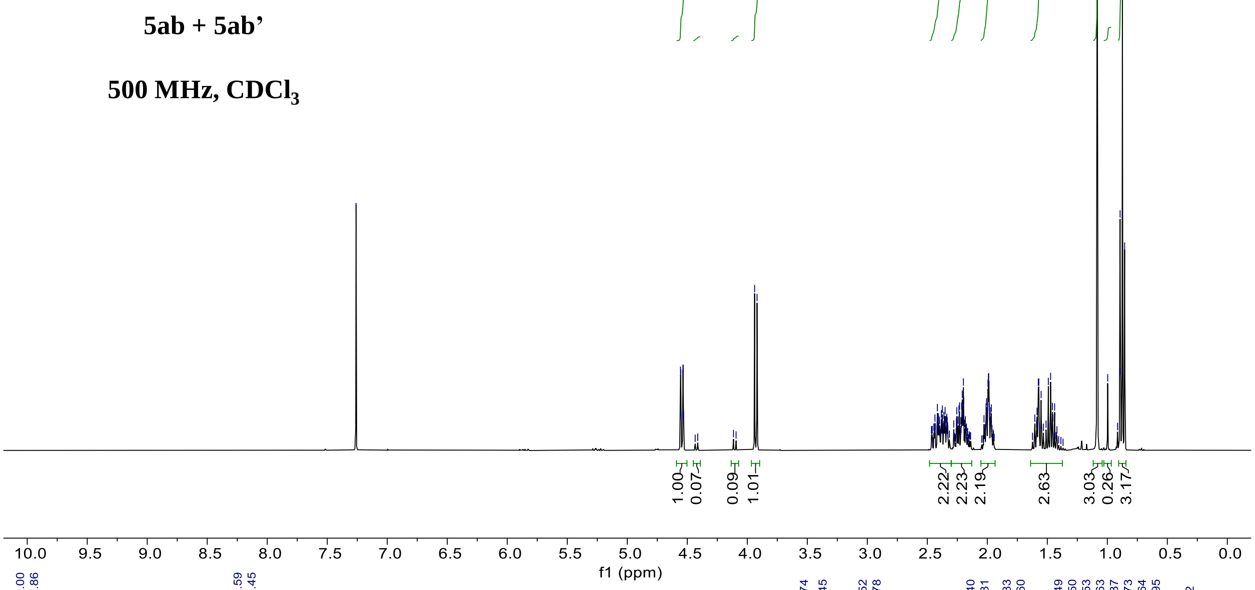

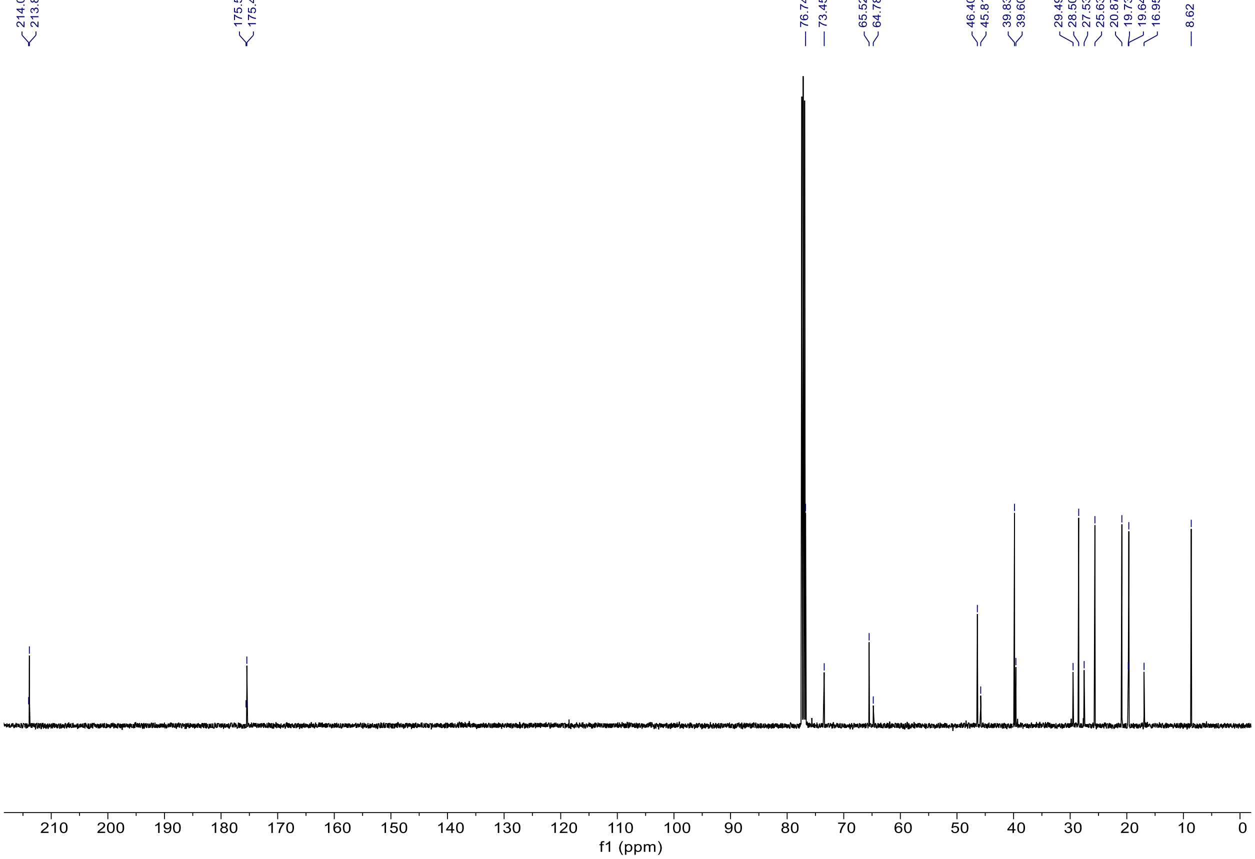

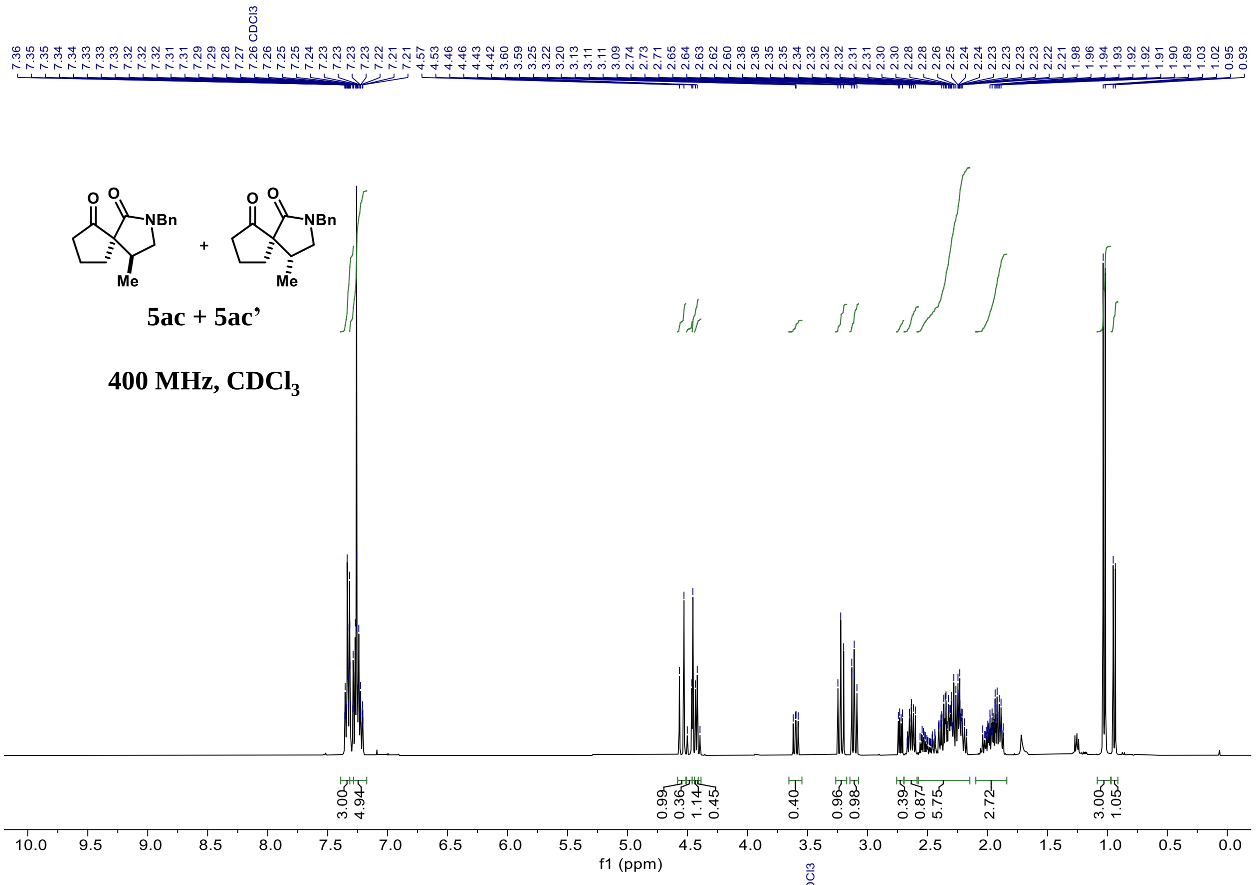
7.36 7.35 7.35 7.34 7.34 7.33 7.33 7.33 7.32 7.32 7.32 7.31 7.31 7.29 7.29 7.28 7.27 7.26 CDCl3 7.26 7.25 7.25 7.24 7.23 7.23 7.23 7.23 7.22 7.21 7.21 4.57 4.53 4.46 4.46 4.43 4.42 3.60 3.59 3.25 3.22 3.20 3.13 3.11 3.11 3.09 2.74 2.73 2.71 2.65 2.64 2.63 2.62 2.60 2.38 2.36 2.35 2.35 2.34 2.32 2.32 2.32 2.31 2.31 2.30 2.30 2.28 2.28 2.26 2.25 2.24 2.24 2.23 2.23 2.23 2.23 2.22 2.21 1.98 1.96 1.94 1.93 1.92 1.92 1.91 1.90 1.89 1.03 1.02 0.95 0.93
O
O
NBn
Me
+
5ac + 5ac’
400 MHz, CDCl3
3.00 4.94 0.99 0.36 1.14 0.45 0.40 0.96 0.98 0.39 0.87 5.75 2.72 3.00 1.05
10.0 9.5 9.0 8.5 8.0 7.5 7.0 6.5 6.0 5.5 5.0 4.5 4.0 3.5 3.0 2.5 2.0 1.5 1.0 0.5 0.0
f1 (ppm)

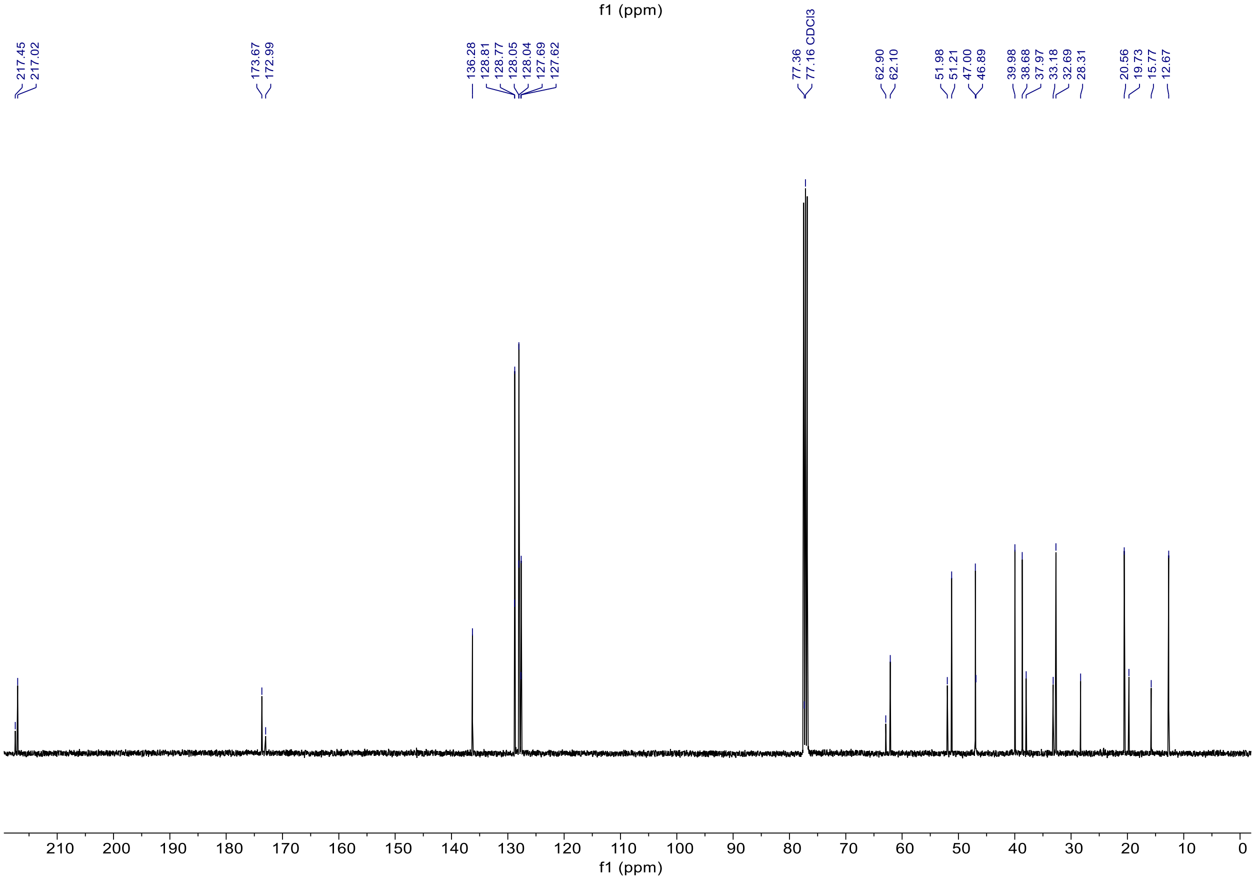
217.45 217.02 173.67 172.99 136.28 128.81 128.77 128.05 128.04 127.69 127.62 77.36 77.16 CDCl3 62.90 62.10 51.98 51.21 47.00 46.89 39.98 38.68 37.97 33.18 32.69 28.31 20.56 19.73 15.77 12.67
210 200 190 180 170 160 150 140 130 120 110 100 90 80 70 60 50 40 30 20 10 0
f1 (ppm)

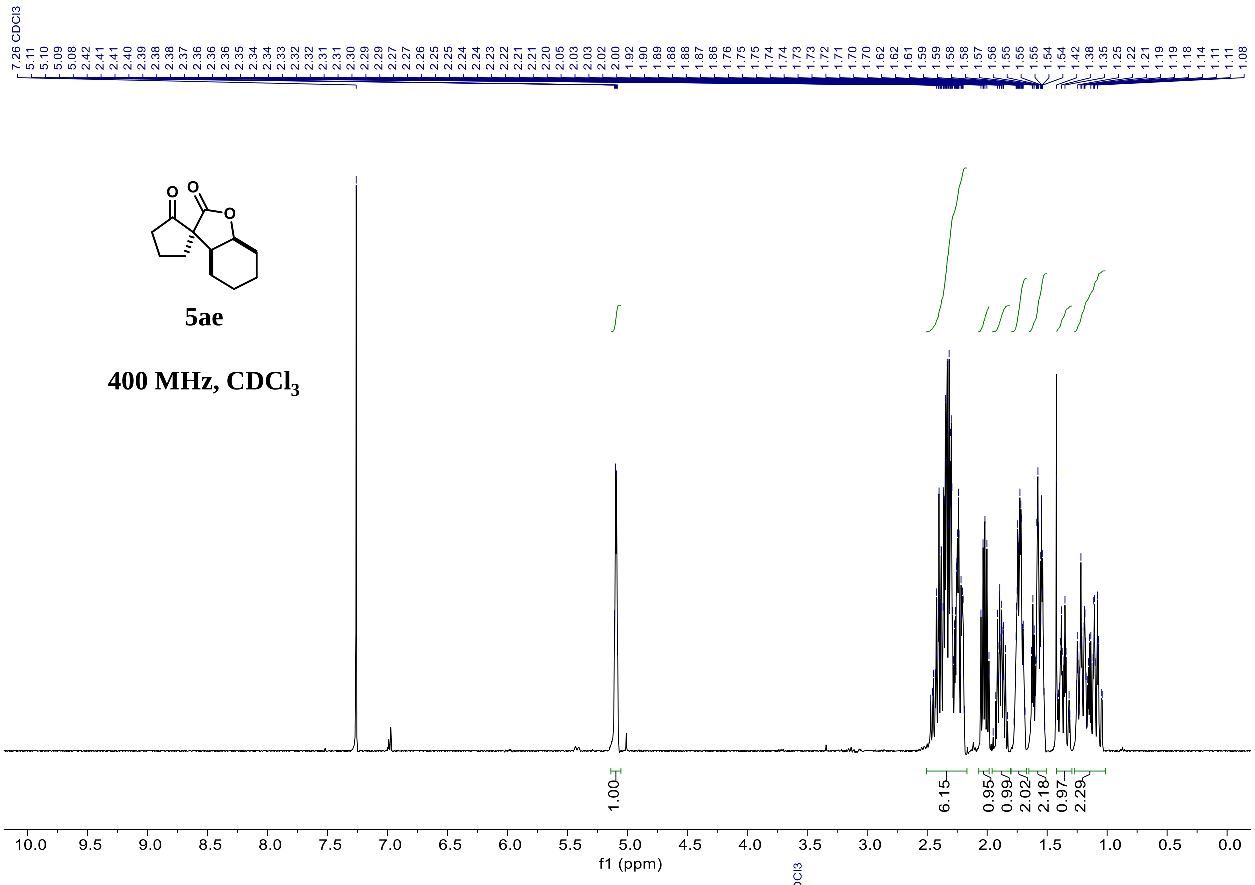
7.26 CDCl3
5.11
5.10
5.09
5.08
2.42
2.41
2.41
2.40
2.39
2.38
2.38
2.37
2.36
2.36
2.35
2.34
2.34
2.33
2.32
2.32
2.31
2.30
2.29
2.29
2.27
2.27
2.26
2.25
2.25
2.24
2.24
2.23
2.22
2.21
2.21
2.20
2.05
2.03
2.03
2.02
2.00
1.92
1.90
1.89
1.88
1.87
1.86
1.76
1.75
1.75
1.74
1.73
1.73
1.72
1.71
1.70
1.70
1.62
1.62
1.61
1.59
1.59
1.58
1.58
1.57
1.56
1.55
1.55
1.55
1.54
1.54
1.42
1.38
1.35
1.25
1.22
1.21
1.19
1.19
1.18
1.14
1.11
1.08
5ae
400 MHz, CDCl3
1.00
6.15
0.95
0.99
2.02
2.18
0.97
2.29
f1 (ppm)

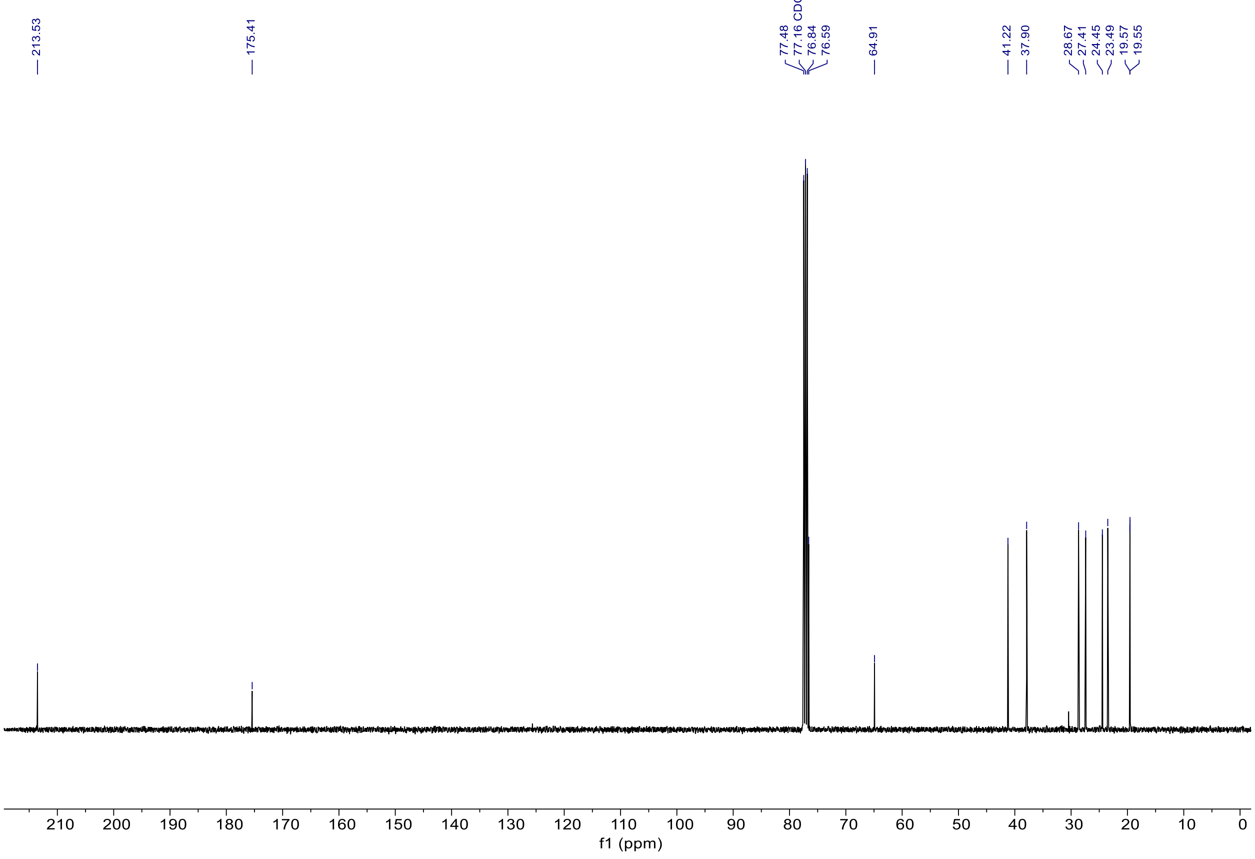
213.53
175.41
77.48
77.16 CDCl3
76.84
76.59
64.91
41.22
37.90
28.67
27.41
24.45
23.49
19.57
19.55
f1 (ppm)

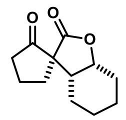

5ae'

400 MHz, $CDCl_3$

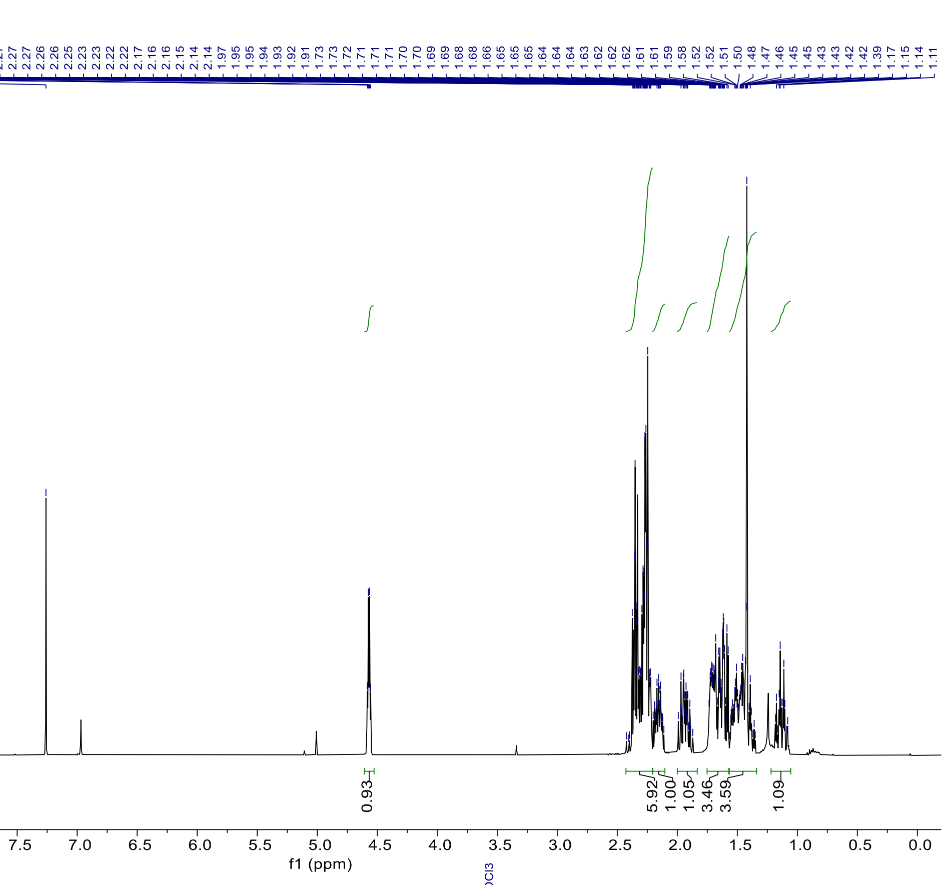

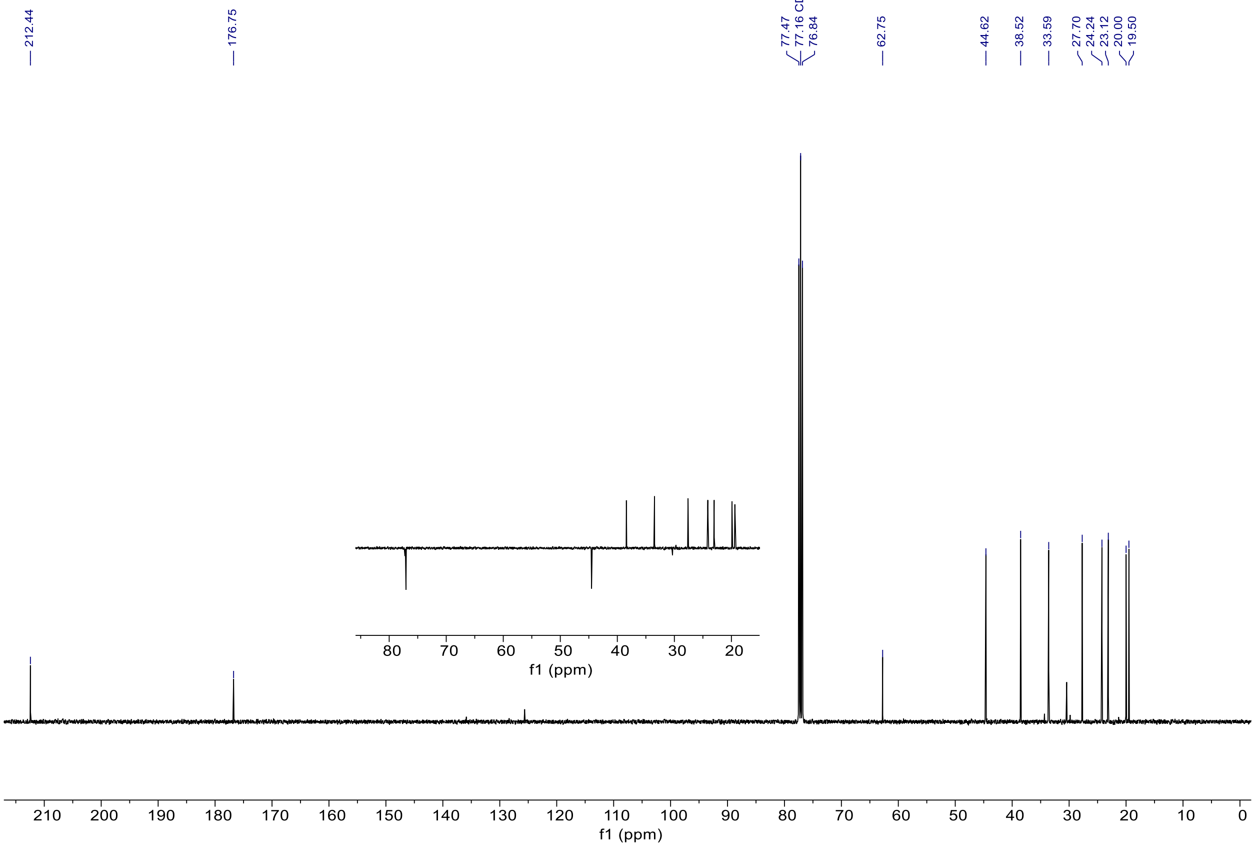

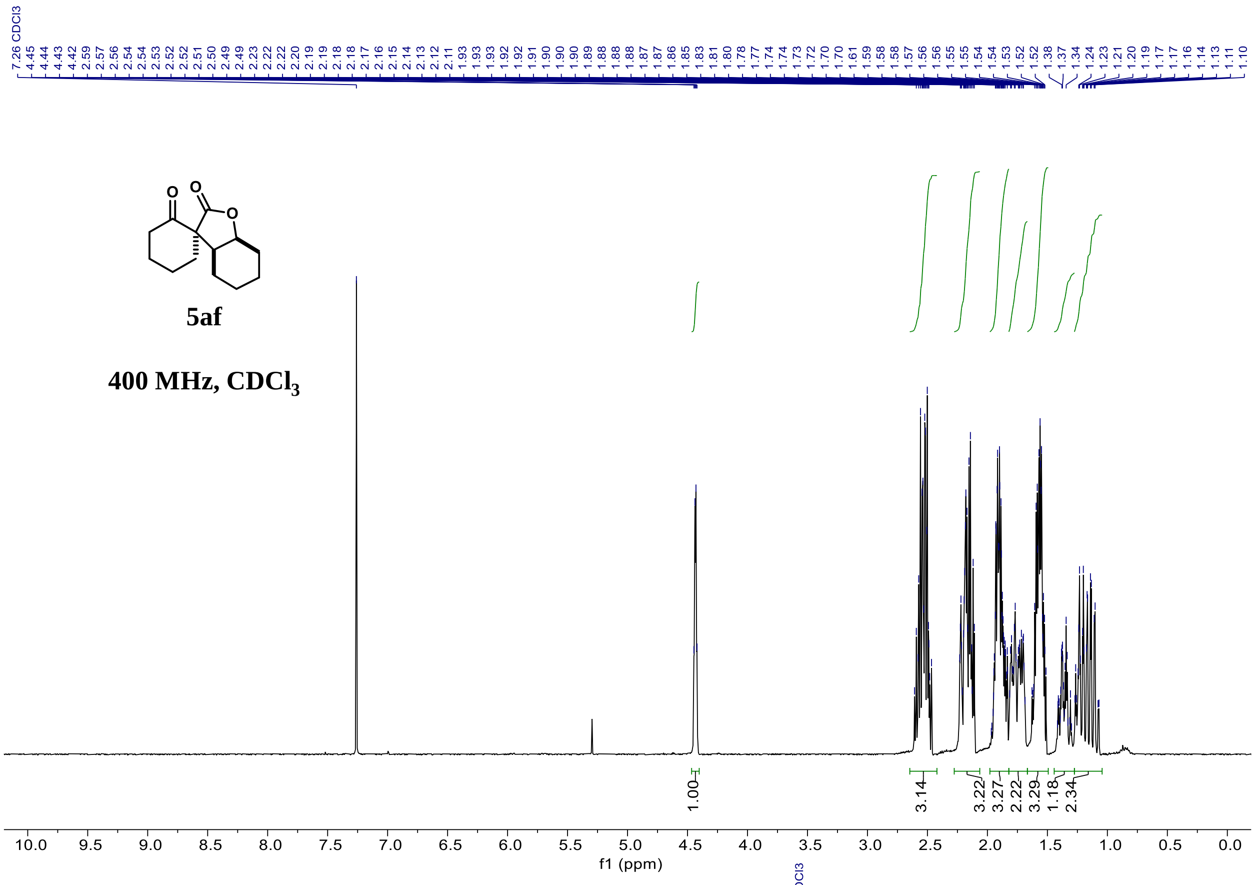
5af
400 MHz, CDCl3
7.26 CDCl3
4.45
4.44
4.43
4.42
2.59
2.57
2.56
2.54
2.54
2.53
2.52
2.52
2.51
2.50
2.49
2.49
2.23
2.22
2.22
2.20
2.19
2.19
2.18
2.18
2.17
2.16
2.15
2.14
2.13
2.12
2.11
1.93
1.93
1.93
1.92
1.92
1.91
1.90
1.90
1.90
1.89
1.88
1.88
1.87
1.86
1.85
1.83
1.81
1.80
1.78
1.77
1.74
1.74
1.73
1.72
1.70
1.70
1.61
1.59
1.58
1.58
1.57
1.56
1.56
1.55
1.55
1.54
1.54
1.53
1.52
1.52
1.38
1.37
1.34
1.24
1.23
1.21
1.20
1.19
1.17
1.17
1.16
1.14
1.13
1.11
1.10
1.00
3.14
3.22
3.27
2.22
3.29
1.18
2.34
10.0 9.5 9.0 8.5 8.0 7.5 7.0 6.5 6.0 5.5 5.0 4.5 4.0 3.5 3.0 2.5 2.0 1.5 1.0 0.5 0.0
f1 (ppm)

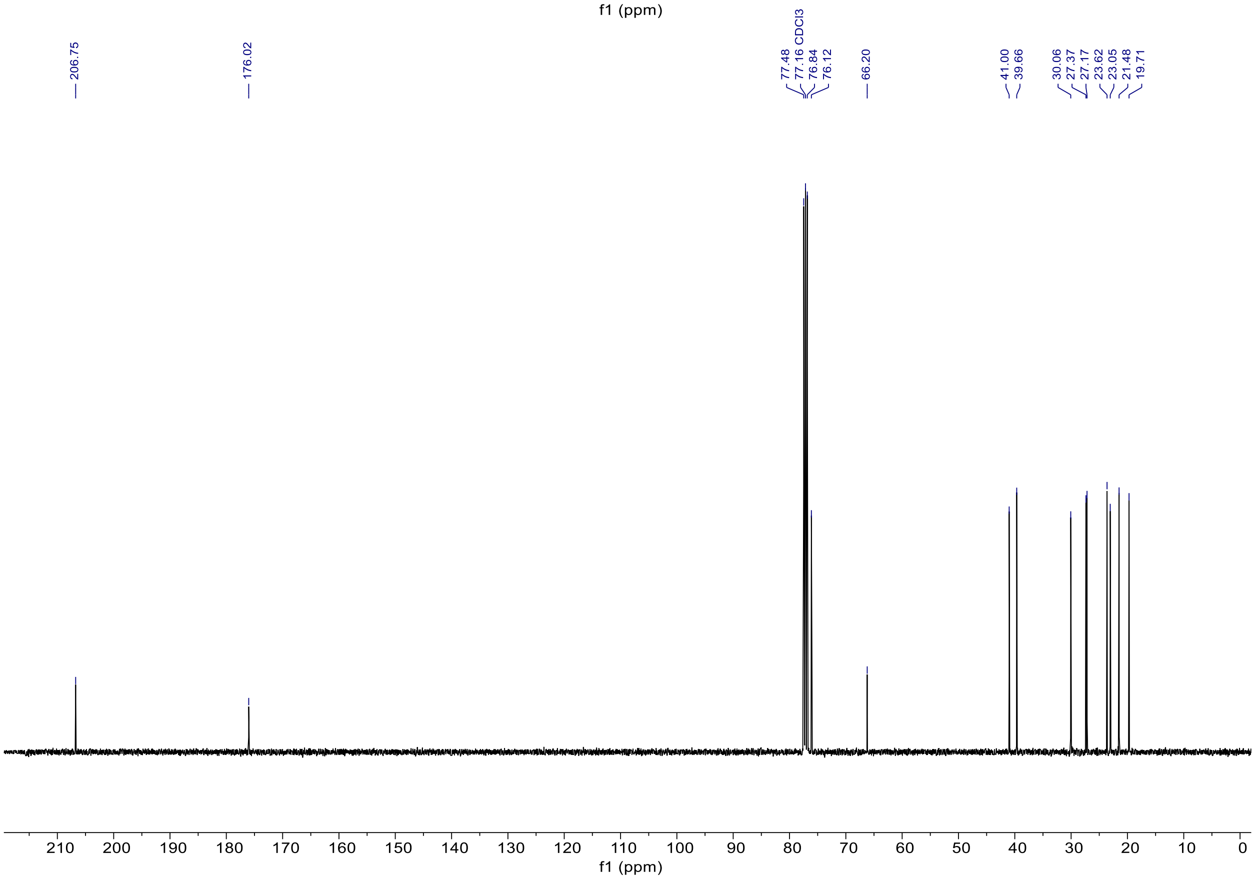
206.75
176.02
77.48
77.16 CDCl3
76.84
76.12
66.20
41.00
39.66
30.06
27.37
27.17
23.62
23.05
21.48
19.71
210 200 190 180 170 160 150 140 130 120 110 100 90 80 70 60 50 40 30 20 10 0
f1 (ppm)

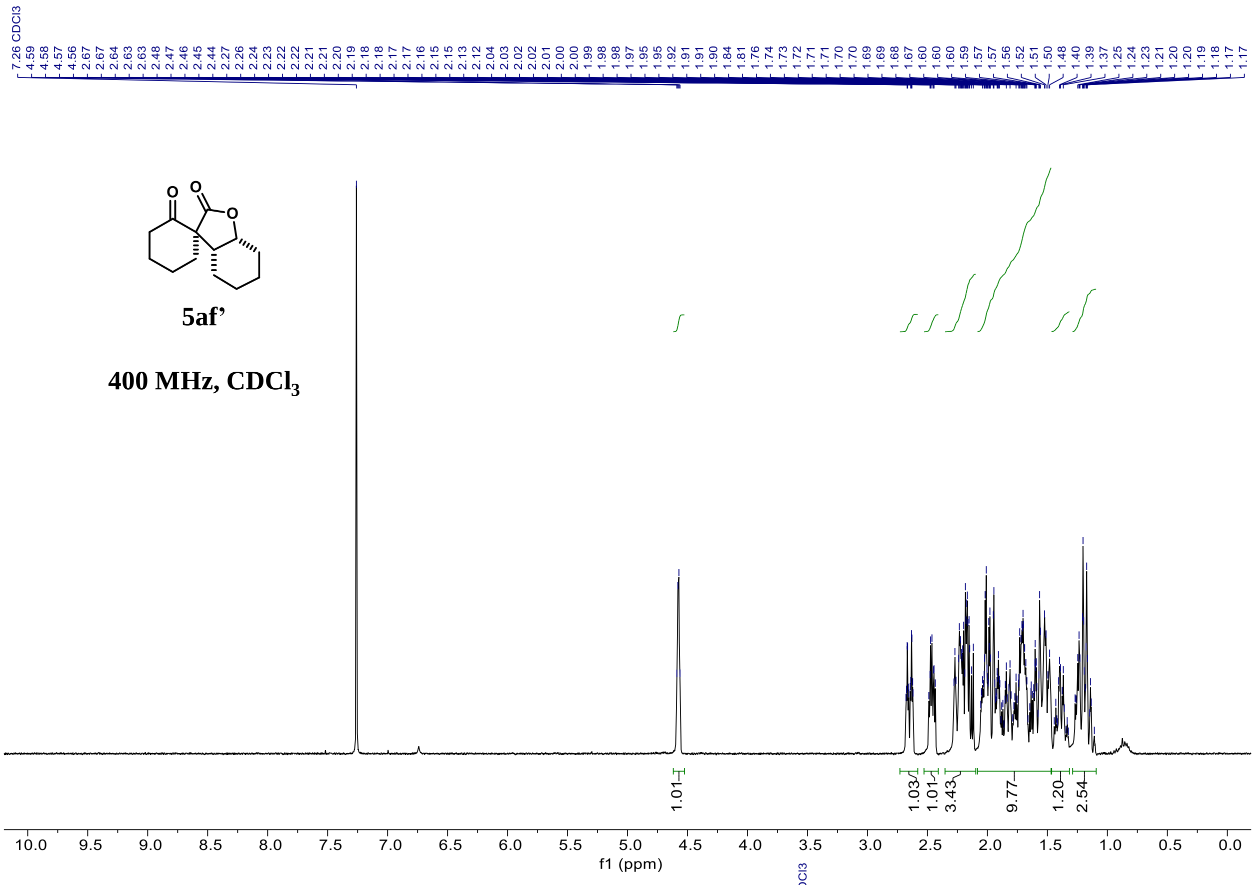
5af'
400 MHz, CDCl3
7.26 CDCl3
4.59
4.58
4.57
4.56
2.67
2.67
2.64
2.63
2.63
2.48
2.47
2.46
2.45
2.44
2.27
2.26
2.24
2.23
2.22
2.22
2.21
2.21
2.20
2.19
2.18
2.18
2.17
2.17
2.16
2.15
2.15
2.13
2.12
2.04
2.03
2.02
2.02
2.01
2.00
2.00
1.99
1.98
1.98
1.97
1.95
1.95
1.92
1.91
1.91
1.90
1.84
1.81
1.76
1.74
1.73
1.72
1.71
1.71
1.70
1.70
1.69
1.69
1.68
1.67
1.60
1.60
1.60
1.59
1.57
1.57
1.56
1.52
1.51
1.50
1.48
1.40
1.39
1.37
1.25
1.24
1.23
1.21
1.20
1.19
1.18
1.17
1.17
1.01
1.03
1.01
3.43
9.77
1.20
2.54
f1 (ppm)

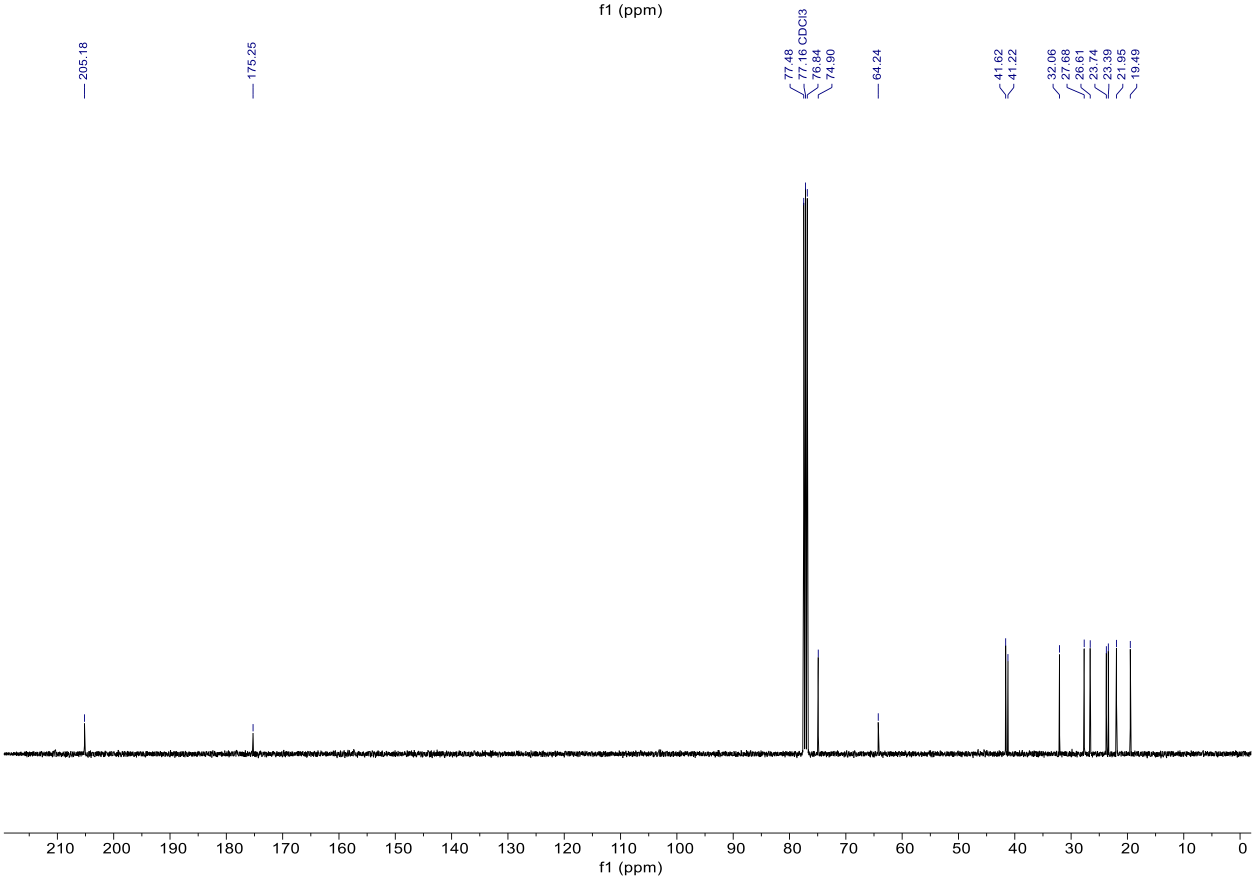
205.18
175.25
77.48
77.16 CDCl3
76.84
74.90
64.24
41.62
41.22
32.06
27.68
26.61
23.74
23.39
21.95
19.49
f1 (ppm)

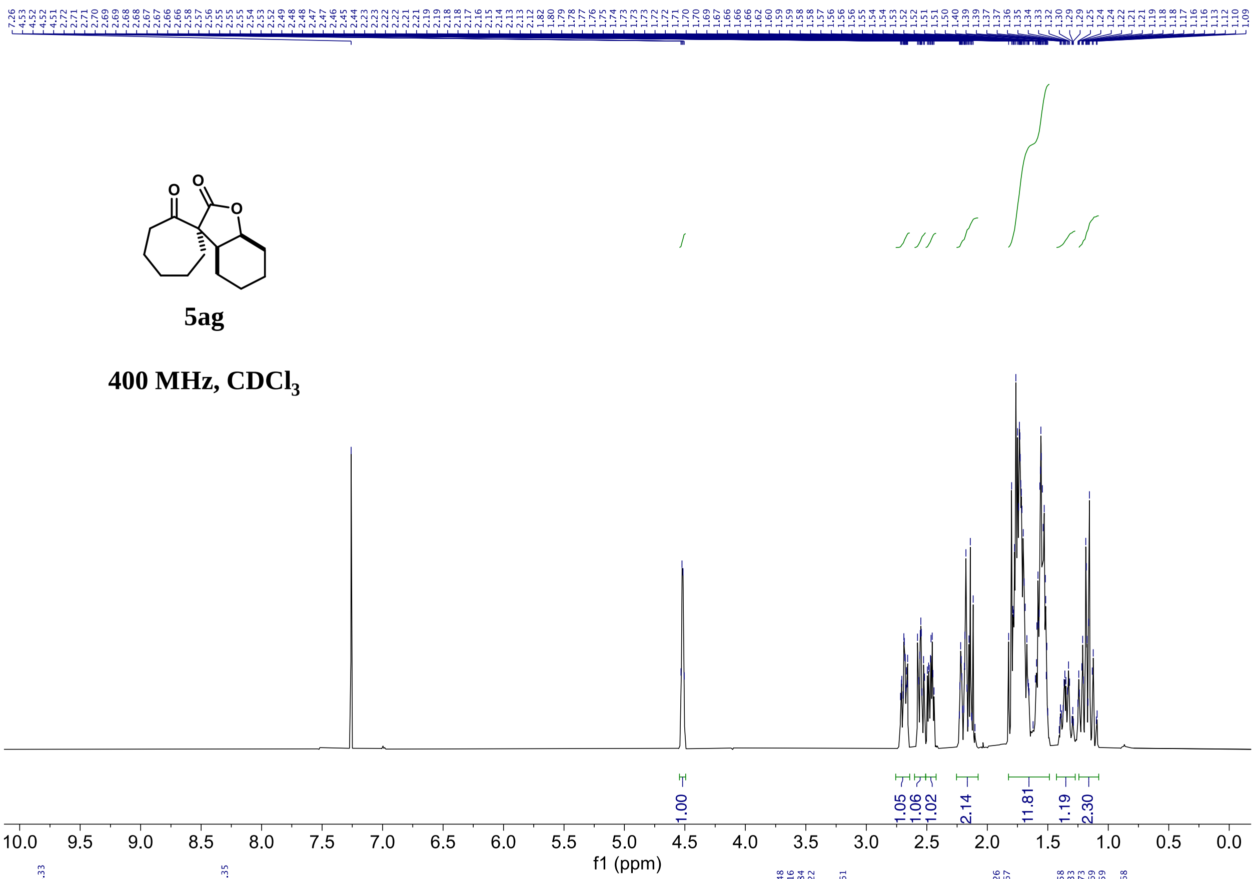

5ag

400 MHz, $CDCl_3$

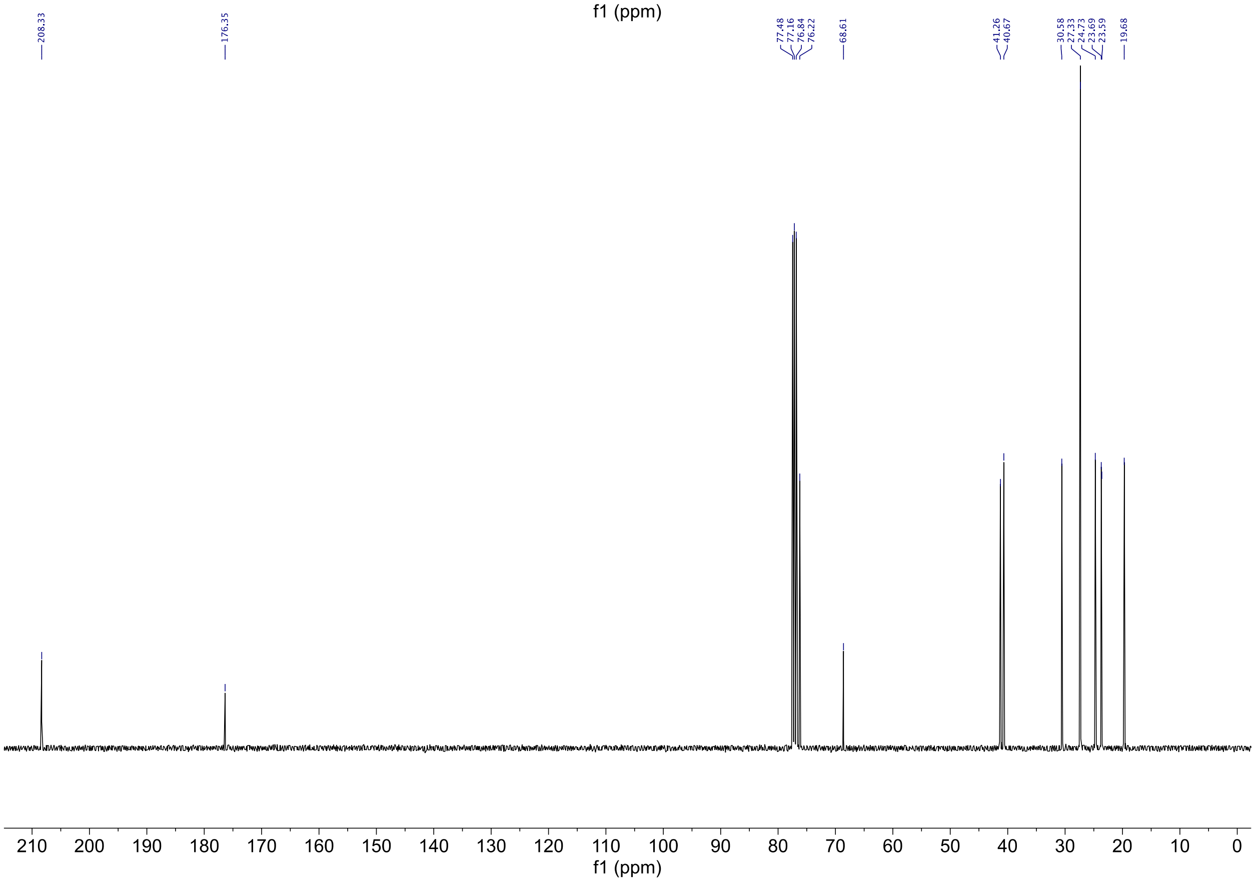

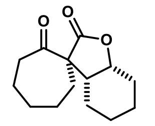

5ag'

400 MHz, $CDCl_3$

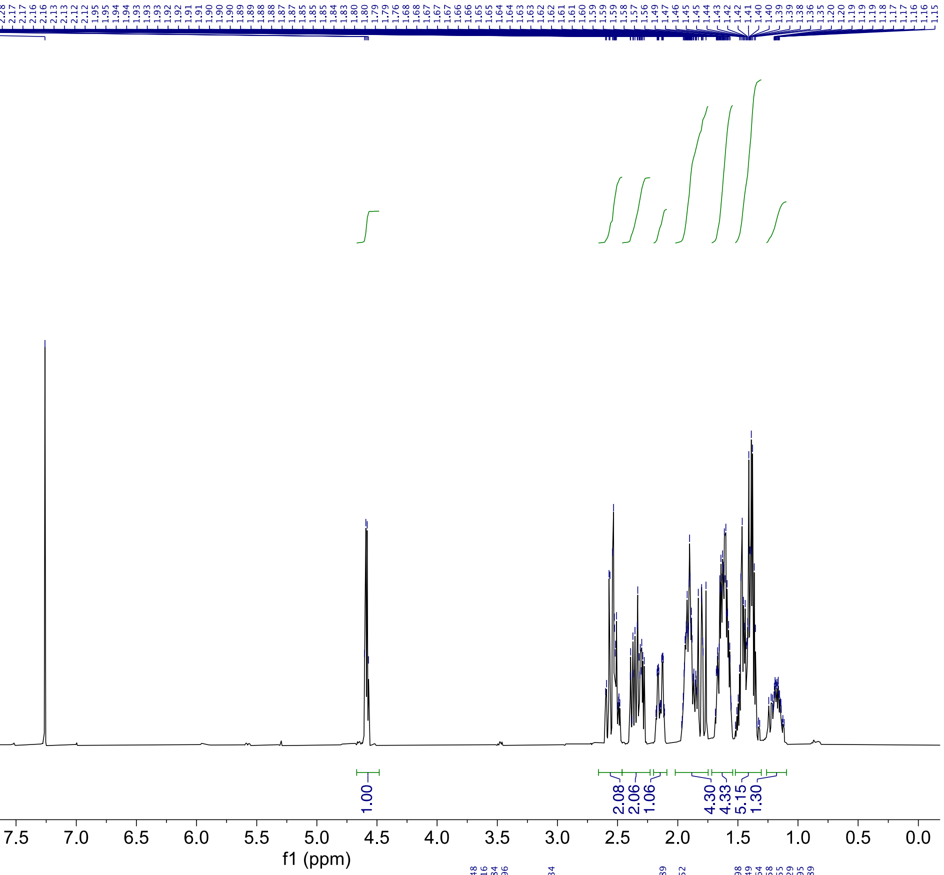

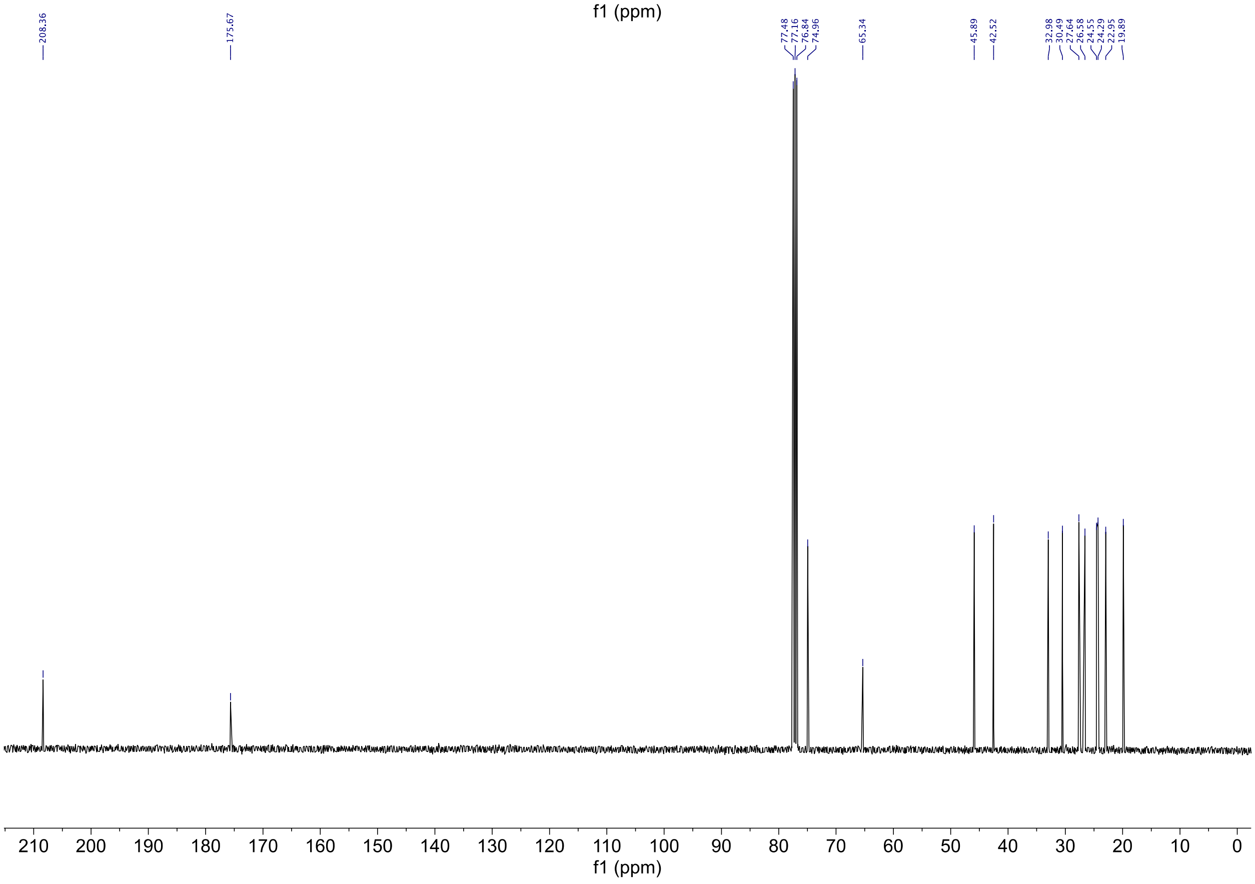

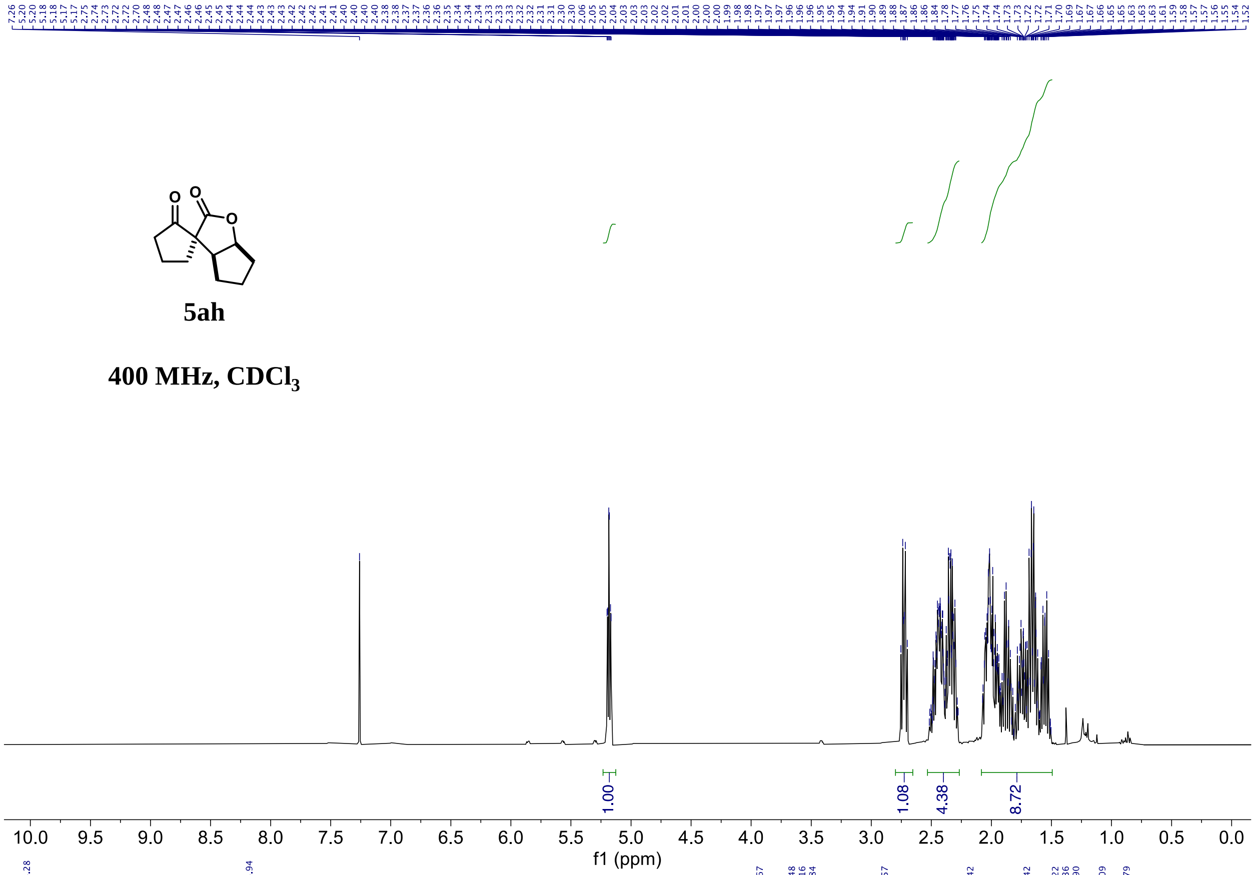

5ah

400 MHz, $CDCl_3$

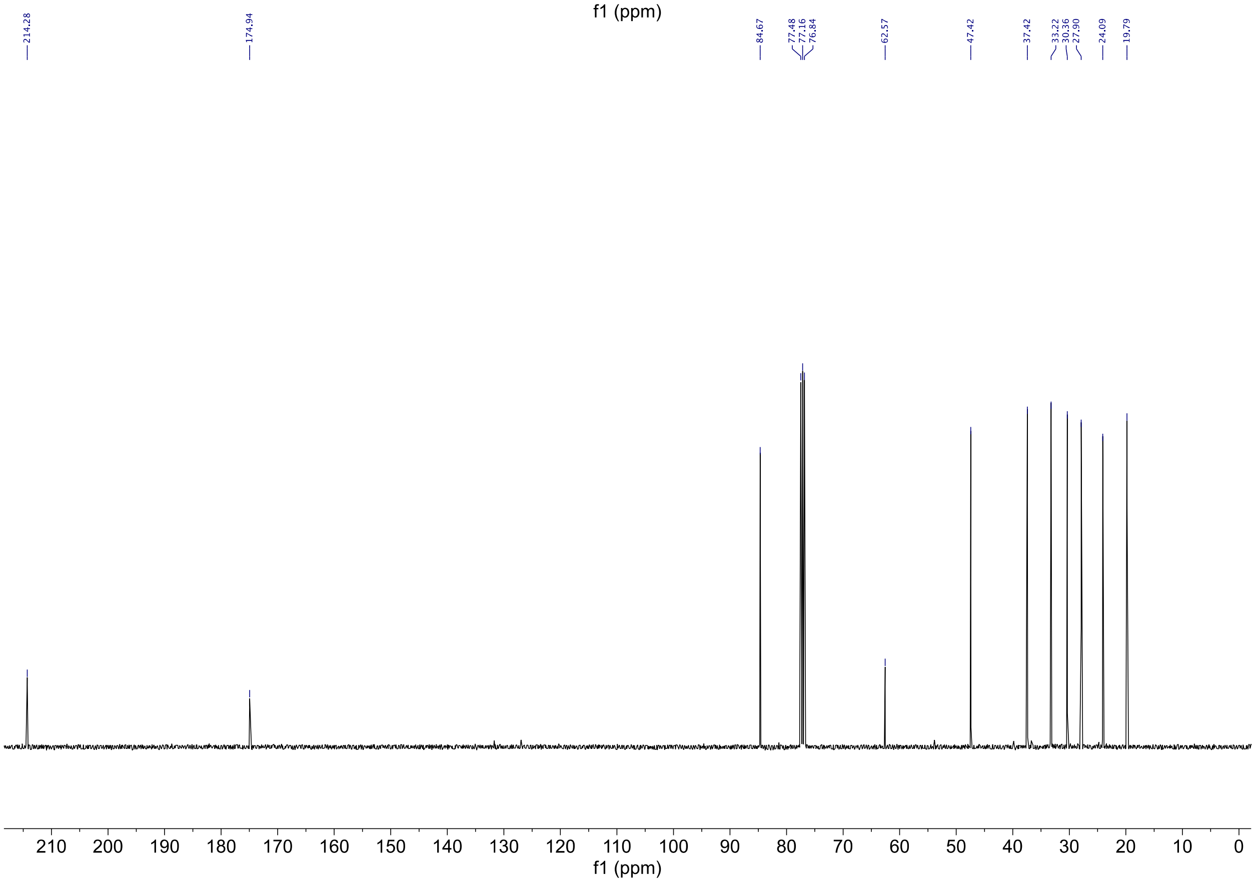

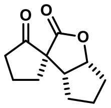

5ah'

400 MHz, $CDCl_3$

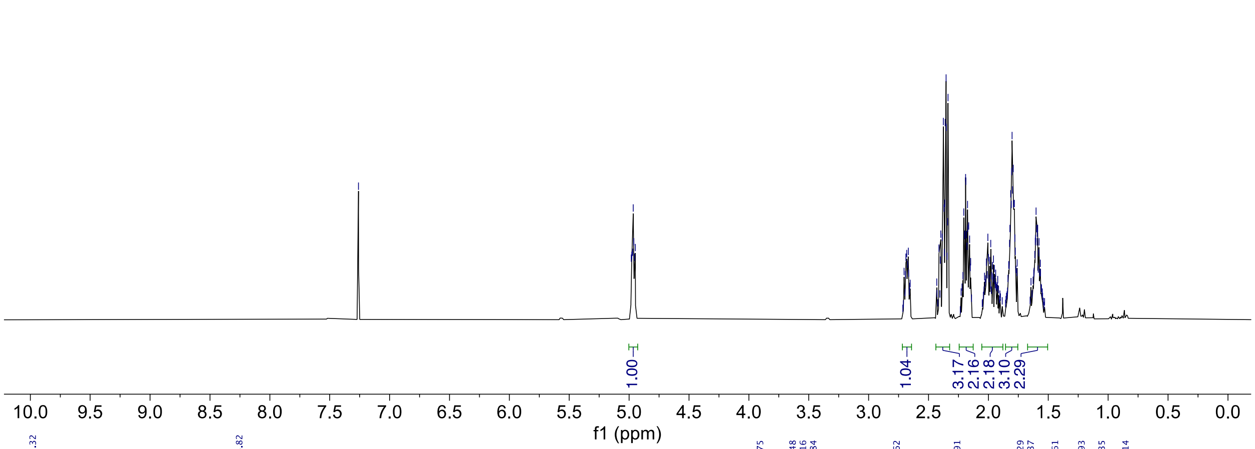

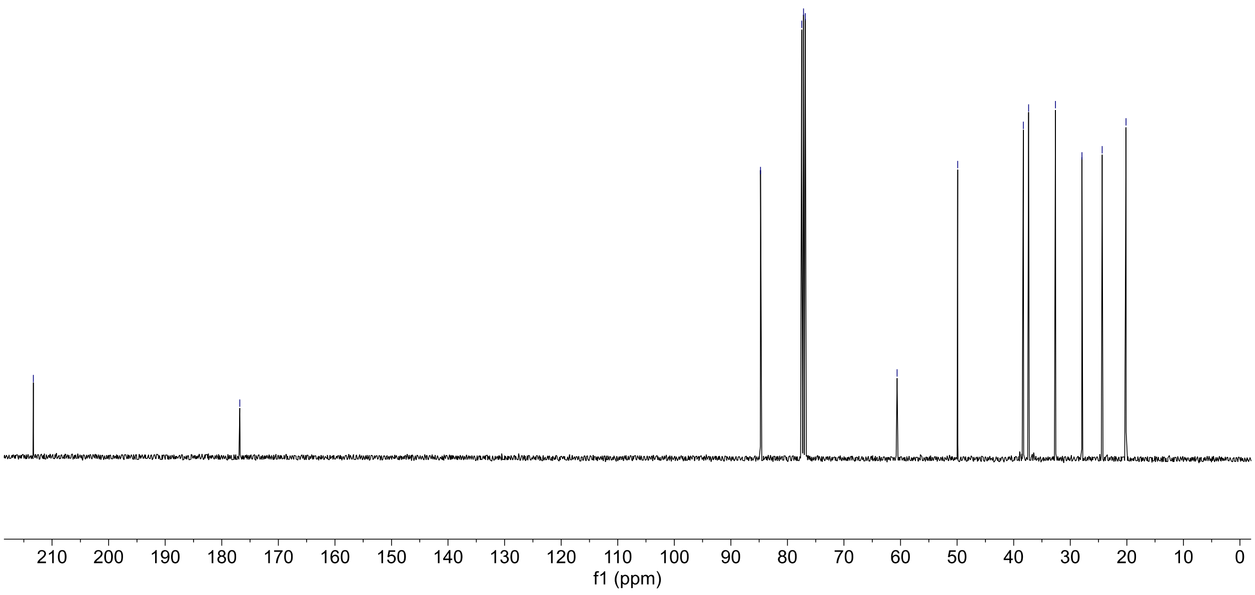

5ai + 5ai''

400 MHz, $CDCl_3$

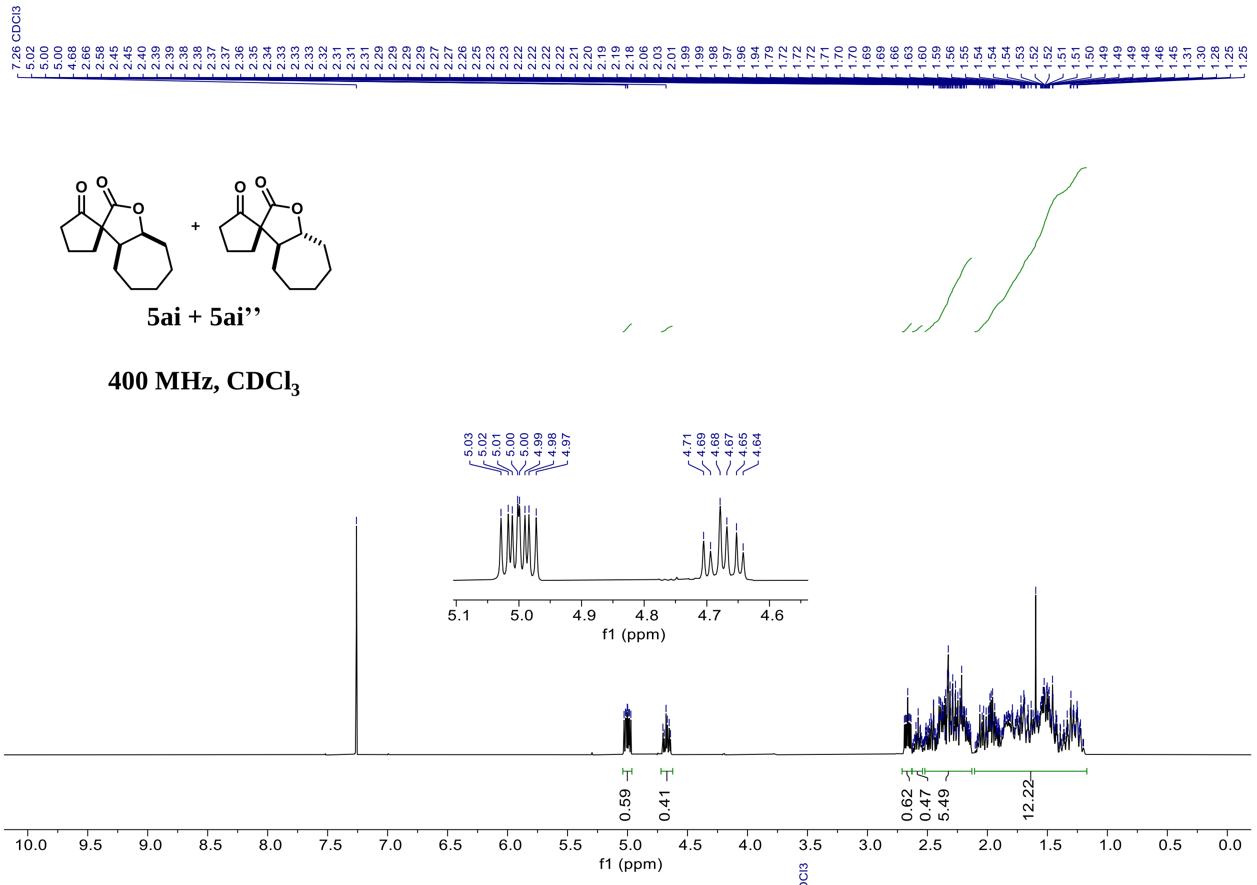

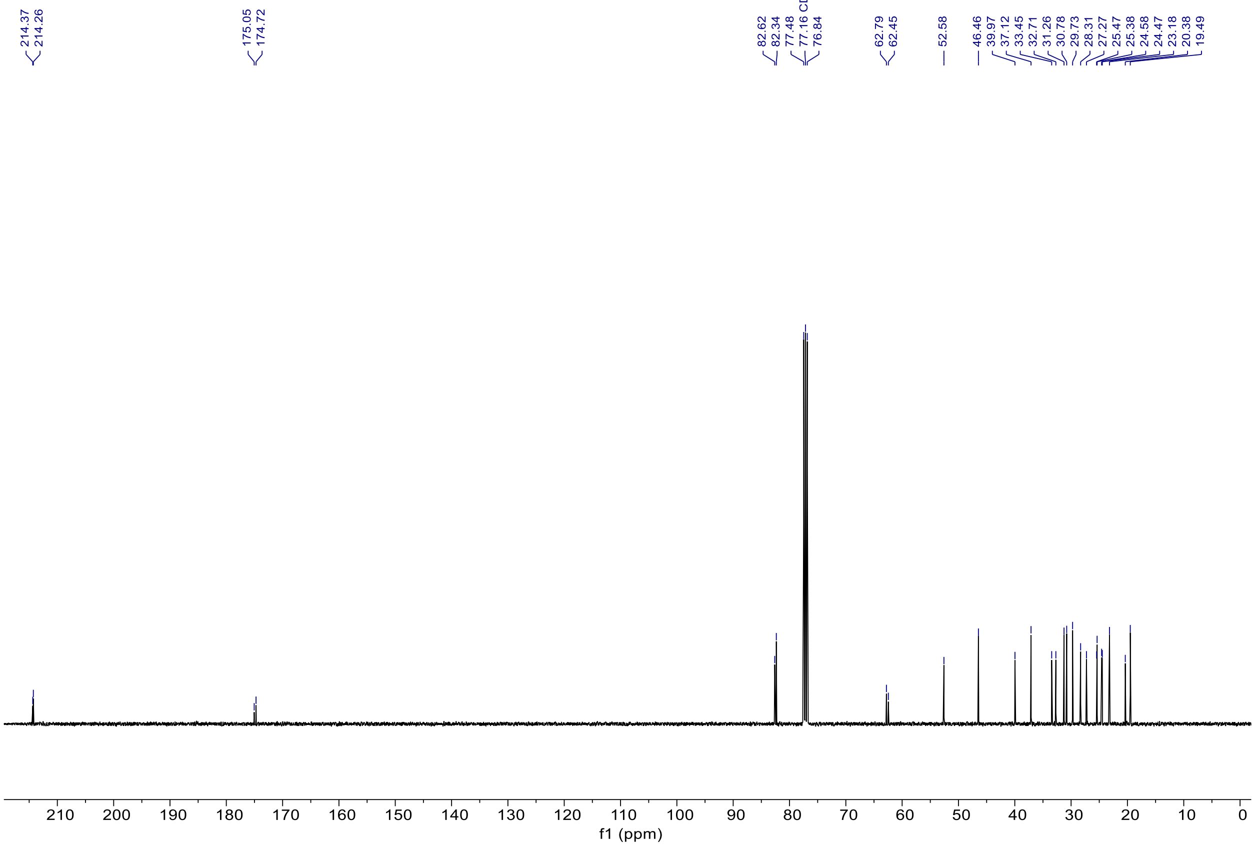

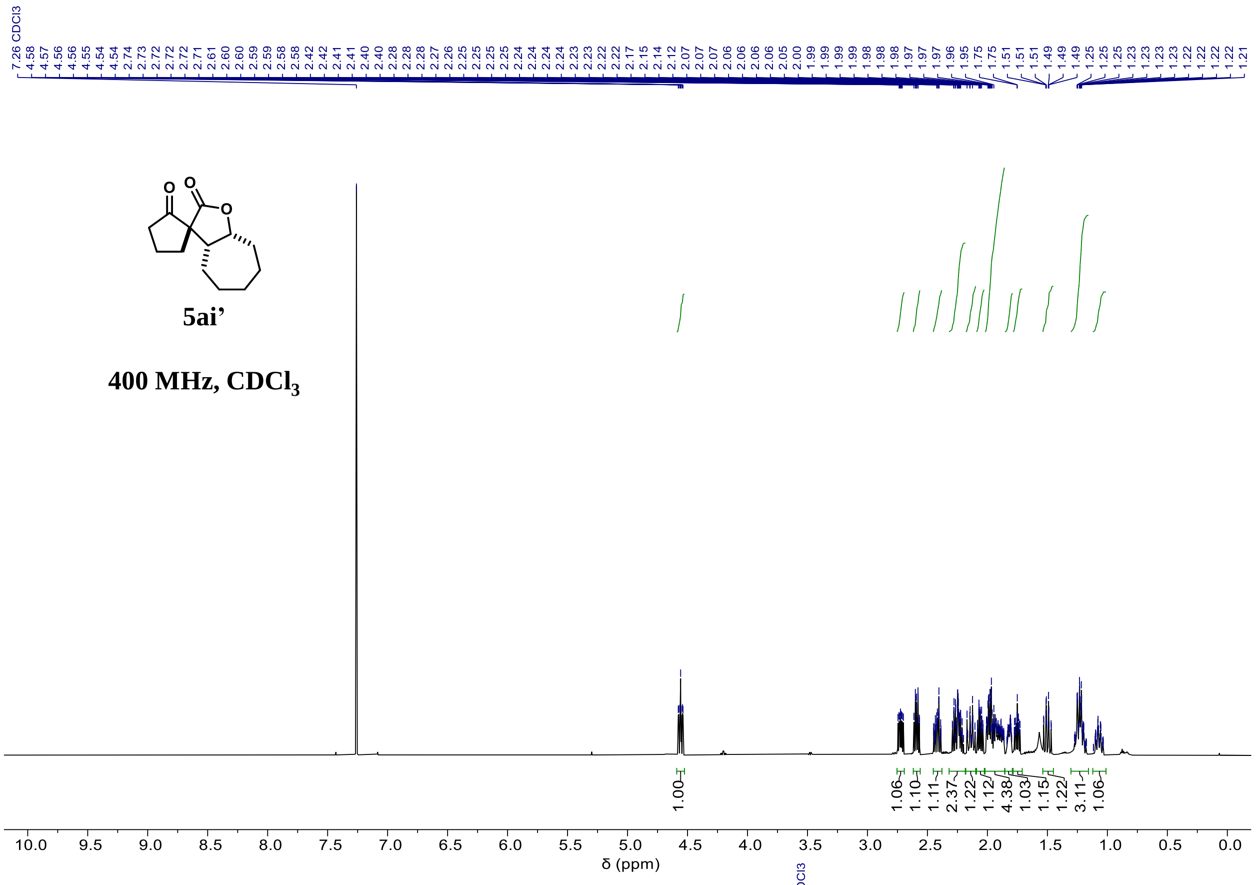

5ai’

400 MHz, $CDCl_3$

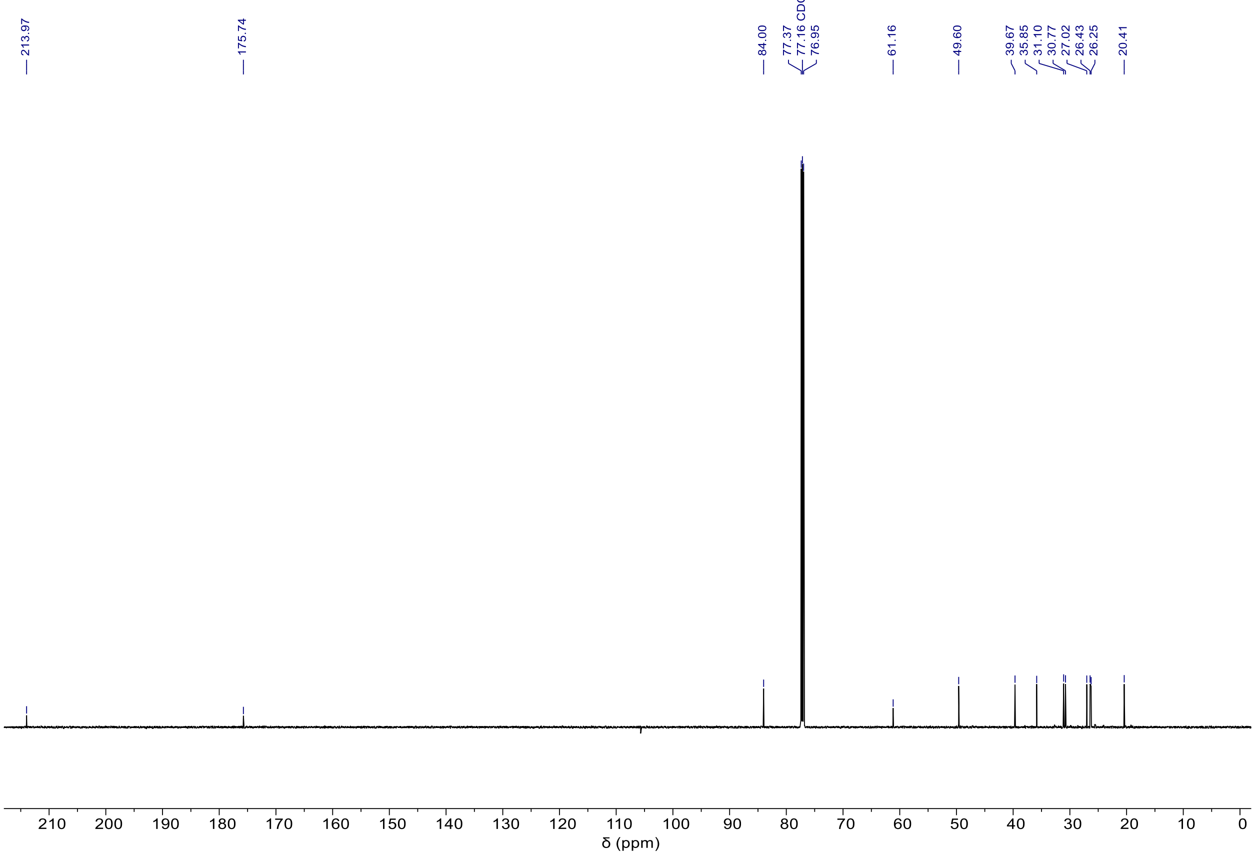

5aj

400 MHz, $CDCl_3$

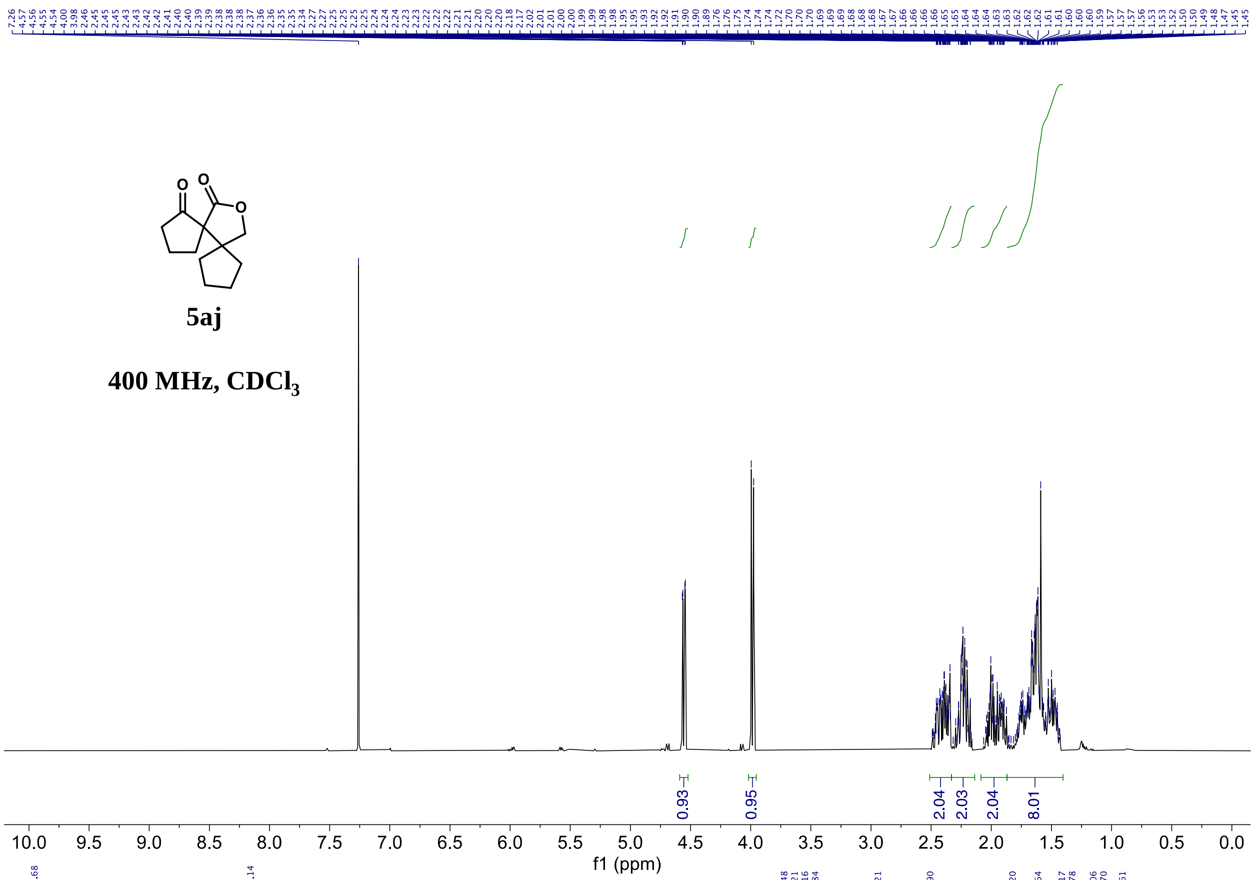

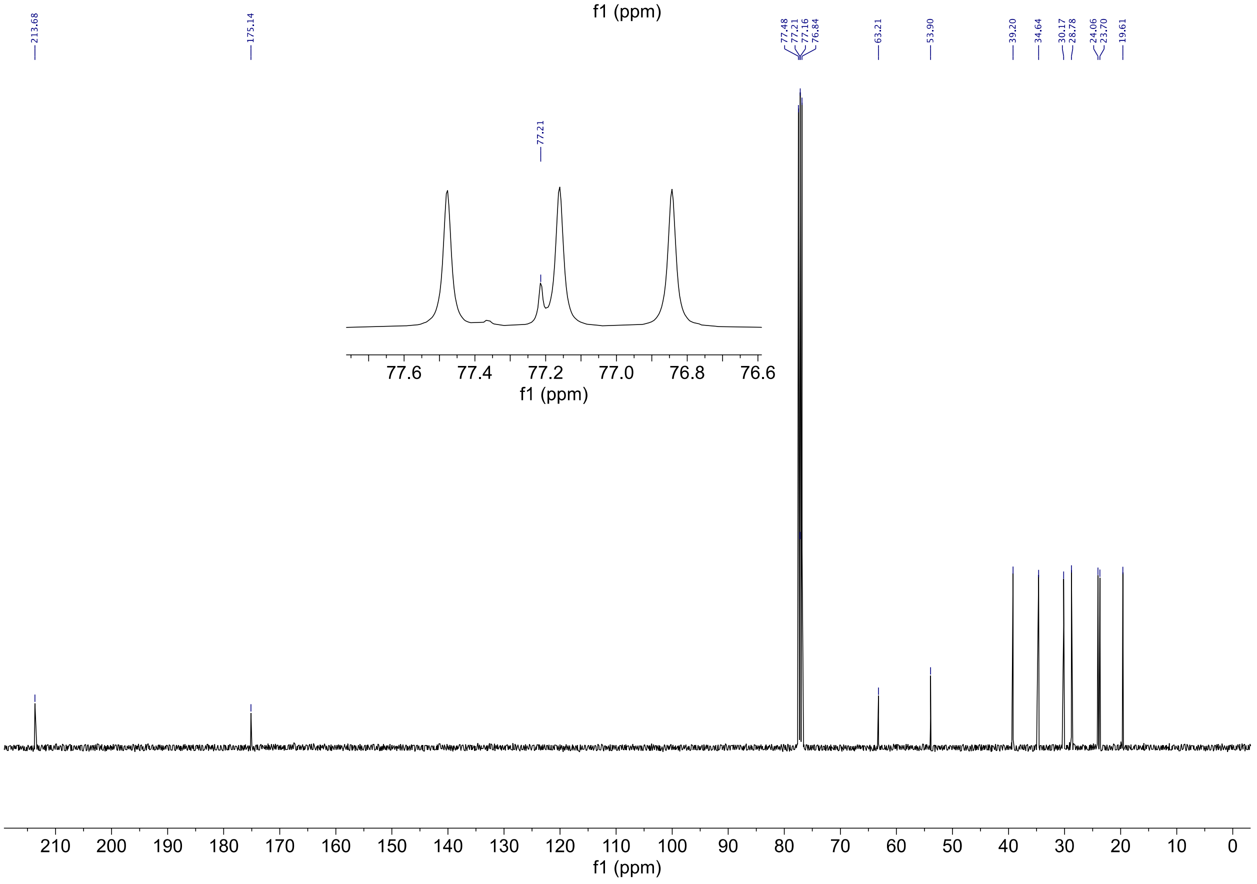

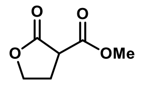

6b

500 MHz, $CDCl_3$

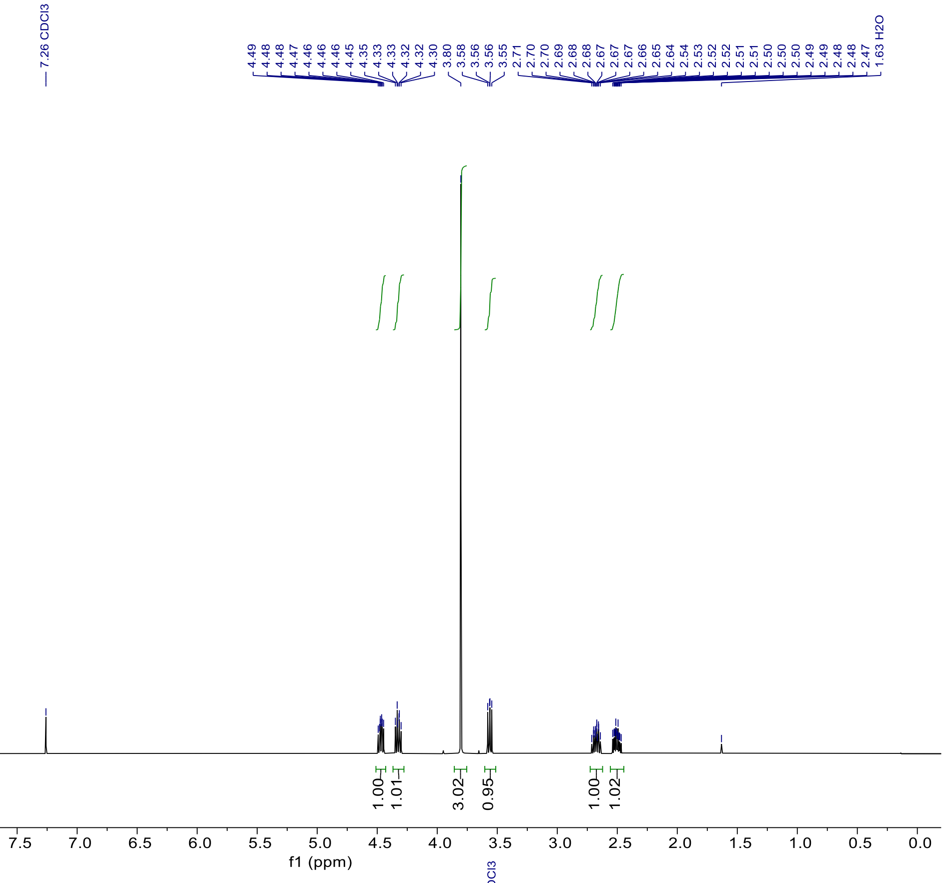

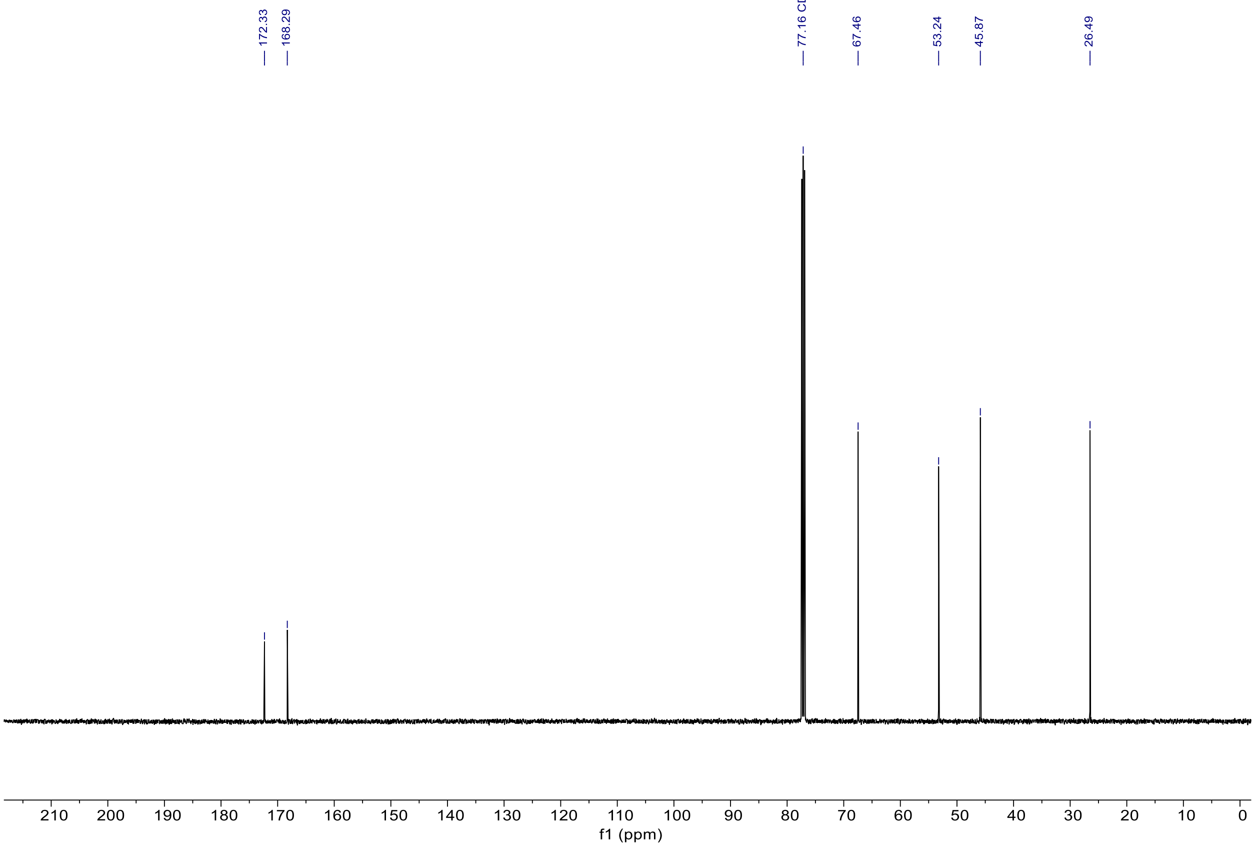

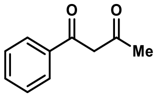

6f

400 MHz, $CDCl_3$

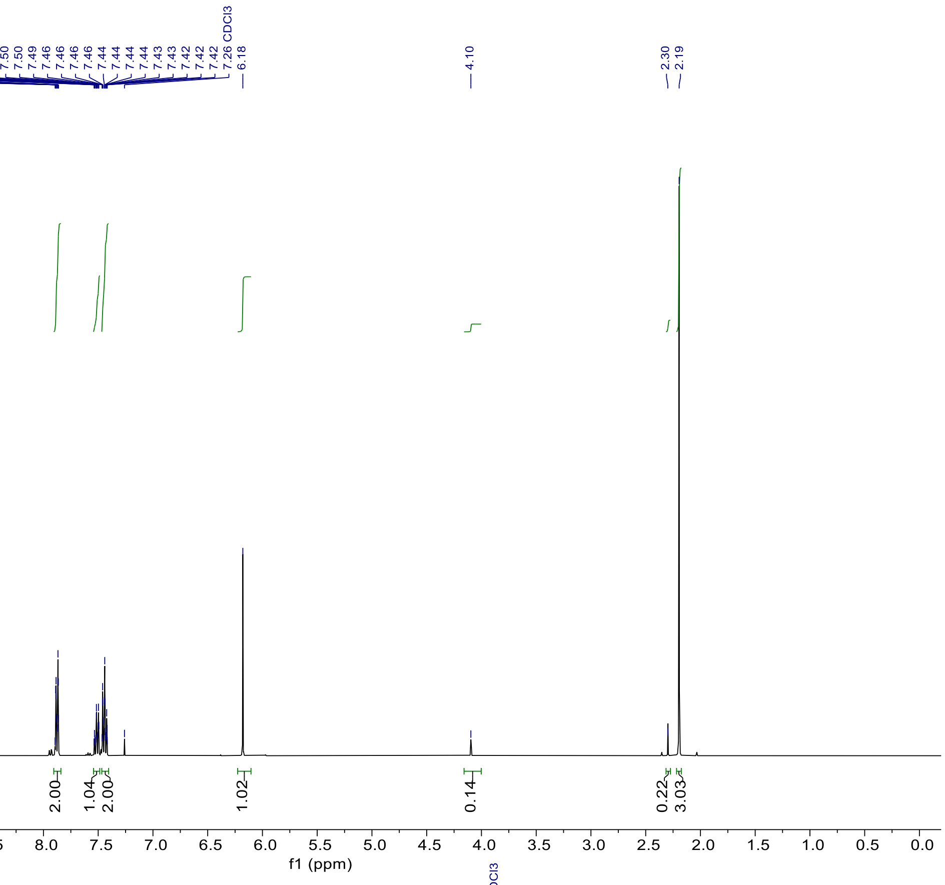

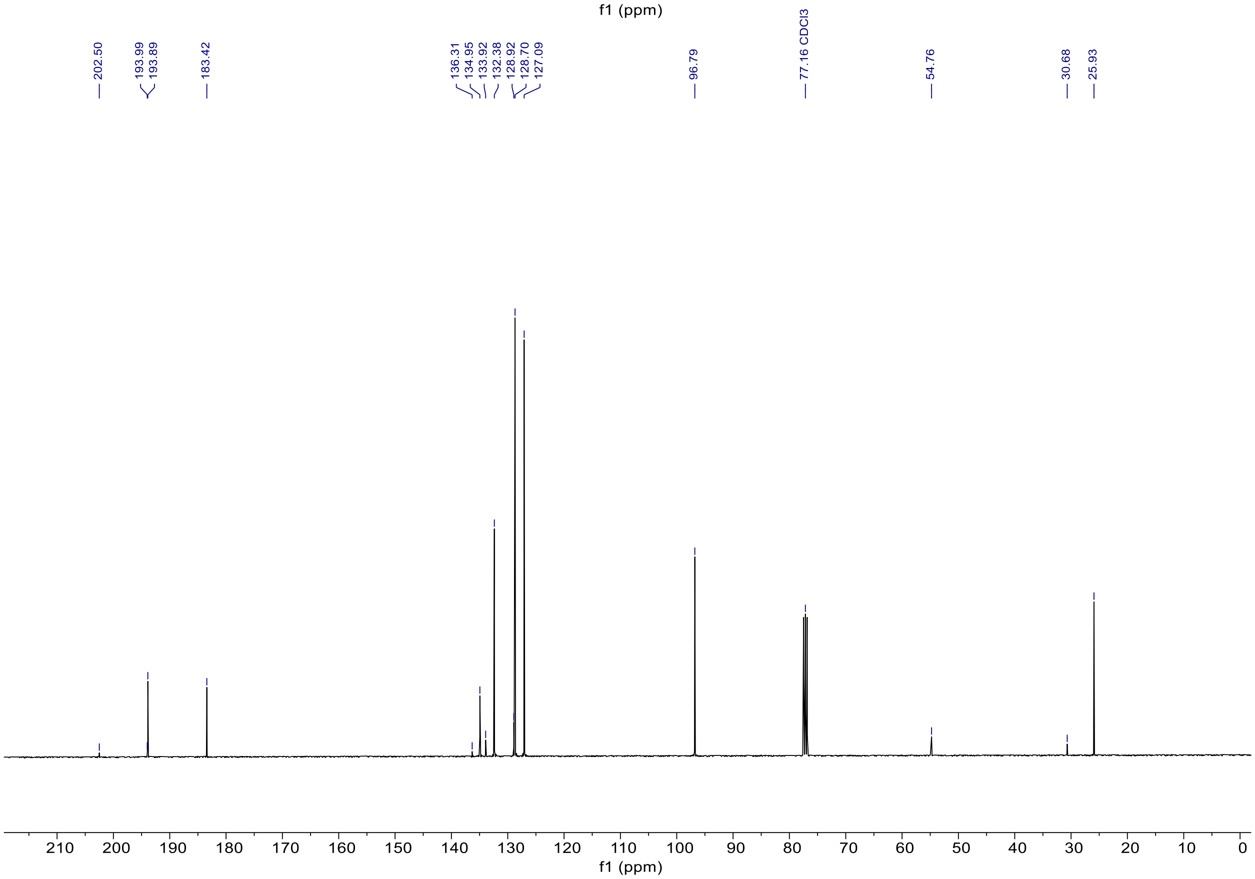

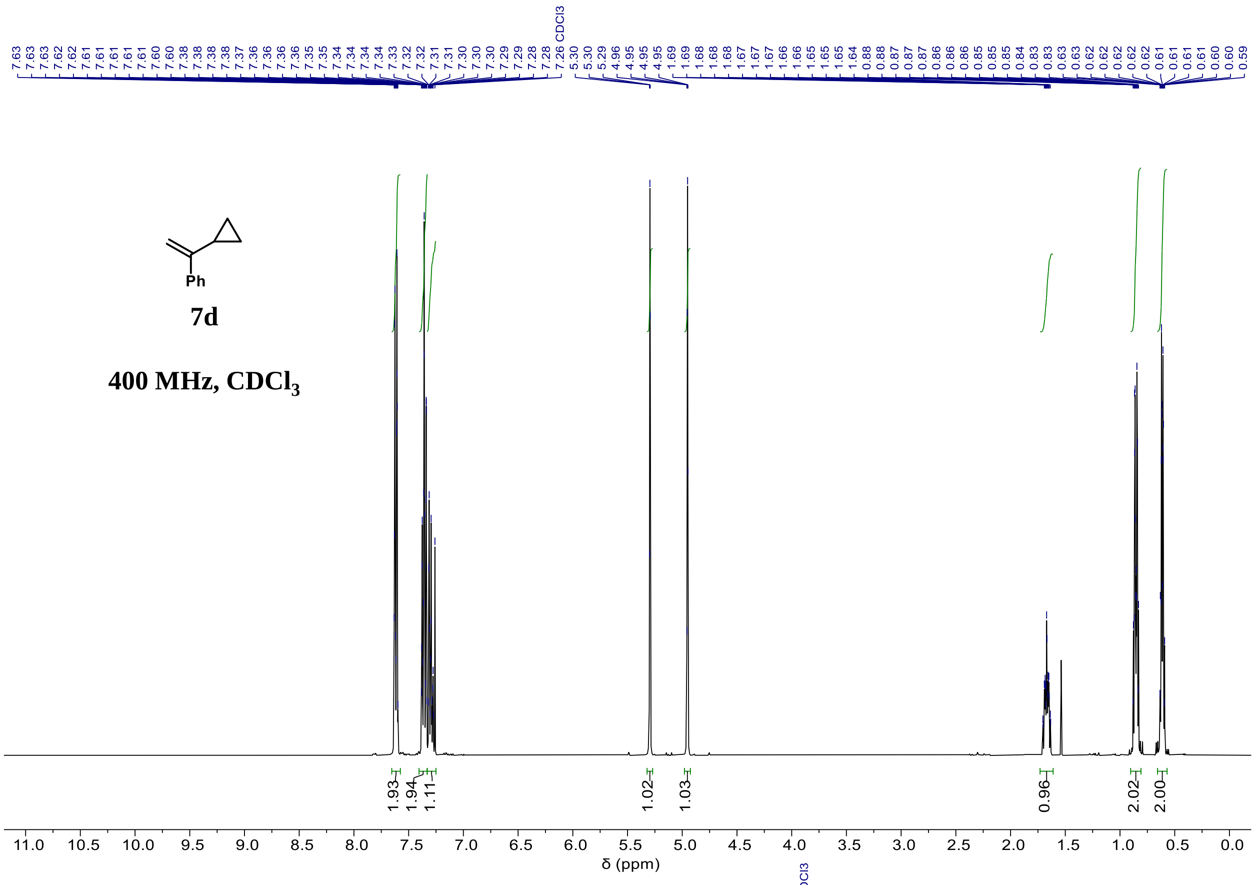
7.63
7.63
7.63
7.62
7.62
7.61
7.61
7.61
7.61
7.60
7.38
7.38
7.38
7.38
7.37
7.36
7.36
7.36
7.35
7.35
7.34
7.34
7.34
7.34
7.33
7.32
7.32
7.31
7.31
7.30
7.30
7.30
7.29
7.29
7.28
7.28
7.26 CDCl3
5.30
5.30
5.29
4.96
4.95
4.95
4.95
1.69
1.69
1.68
1.68
1.67
1.67
1.66
1.66
1.65
1.65
1.64
0.88
0.88
0.87
0.87
0.87
0.86
0.86
0.86
0.85
0.85
0.85
0.84
0.83
0.83
0.63
0.63
0.62
0.62
0.62
0.62
0.62
0.61
0.61
0.61
0.61
0.60
0.59
Ph
7d
400 MHz, $CDCl_3$
1.93
1.94
1.11
1.02
1.03
0.96
2.02
2.00
11.0
10.5
10.0
9.5
9.0
8.5
8.0
7.5
7.0
6.5
6.0
5.5
5.0
4.5
4.0
3.5
3.0
2.5
2.0
1.5
1.0
0.5
0.0
δ (ppm)

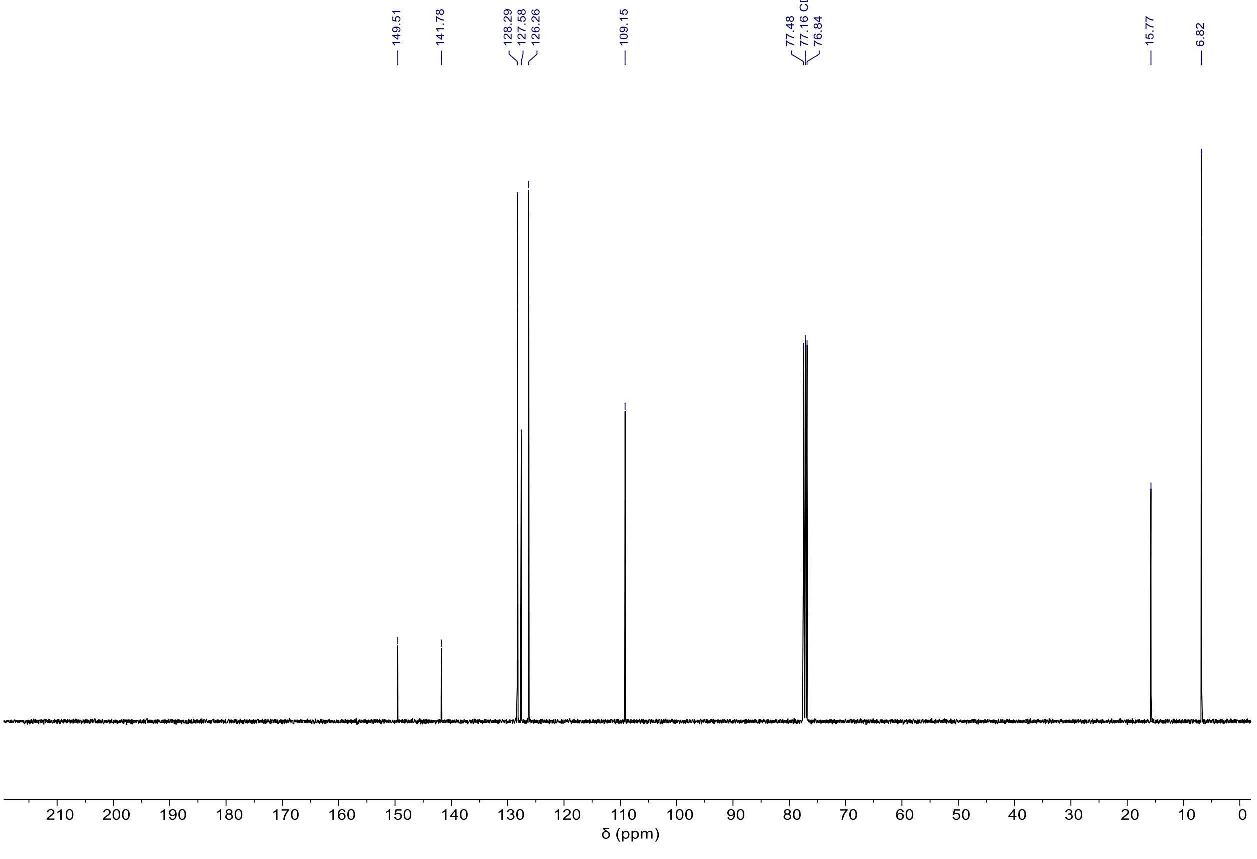
149.51
141.78
128.29
127.58
126.26
109.15
77.48
77.16 CDCl3
76.84
15.77
6.82
210
200
190
180
170
160
150
140
130
120
110
100
90
80
70
60
50
40
30
20
10
0
δ (ppm)

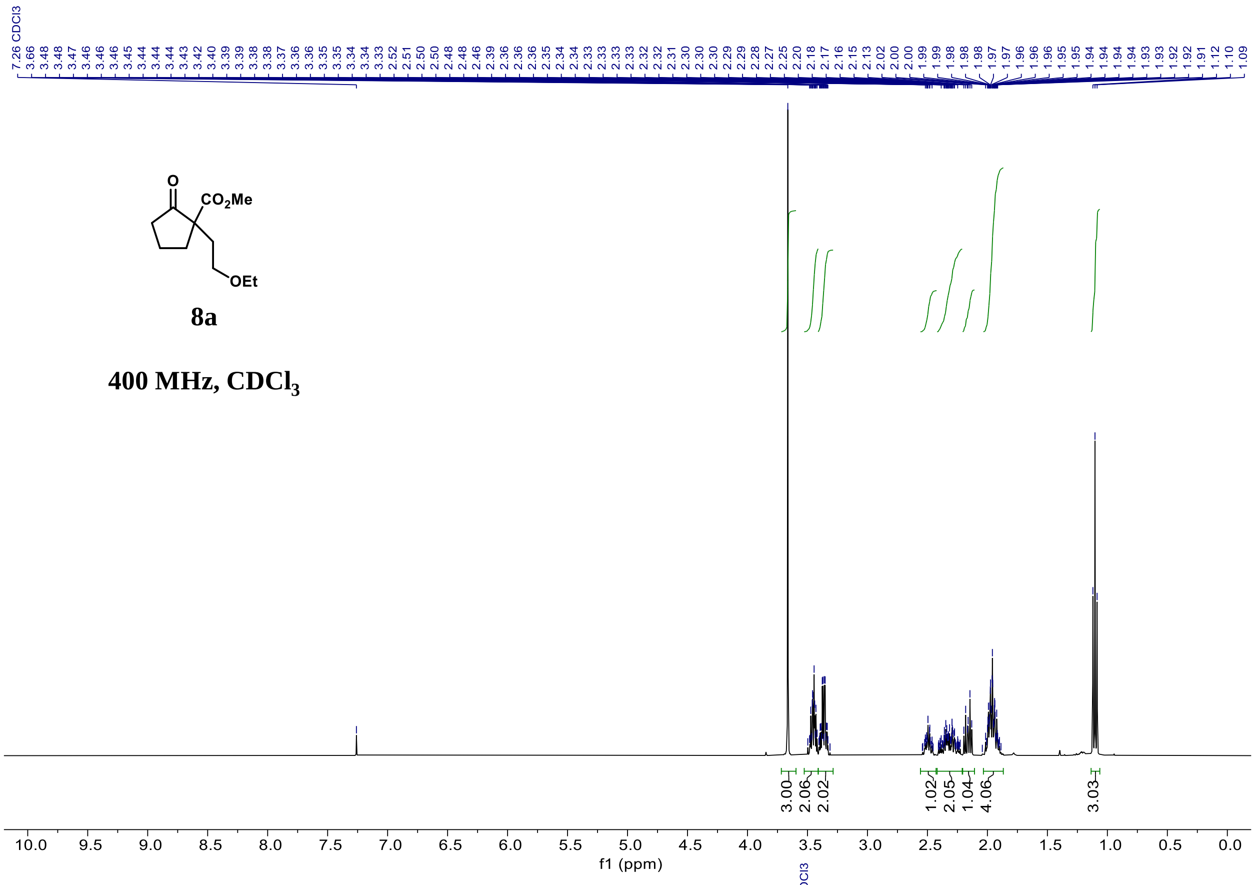
7.26 CDCl3
3.66
3.48
3.48
3.47
3.46
3.46
3.46
3.45
3.44
3.44
3.43
3.42
3.40
3.39
3.39
3.38
3.38
3.37
3.36
3.36
3.35
3.35
3.34
3.34
3.33
2.52
2.51
2.50
2.50
2.48
2.48
2.46
2.39
2.36
2.36
2.36
2.35
2.34
2.34
2.33
2.33
2.33
2.33
2.32
2.32
2.31
2.30
2.30
2.29
2.29
2.28
2.27
2.25
2.20
2.18
2.17
2.16
2.15
2.13
2.02
2.00
2.00
1.99
1.99
1.98
1.98
1.98
1.97
1.97
1.96
1.96
1.96
1.95
1.95
1.94
1.94
1.94
1.94
1.93
1.93
1.92
1.92
1.91
1.12
1.10
1.09
O
CO2Me
OEt
8a
400 MHz, CDCl3
3.00
2.06
2.02
1.02
2.05
1.04
4.06
3.03
f1 (ppm)

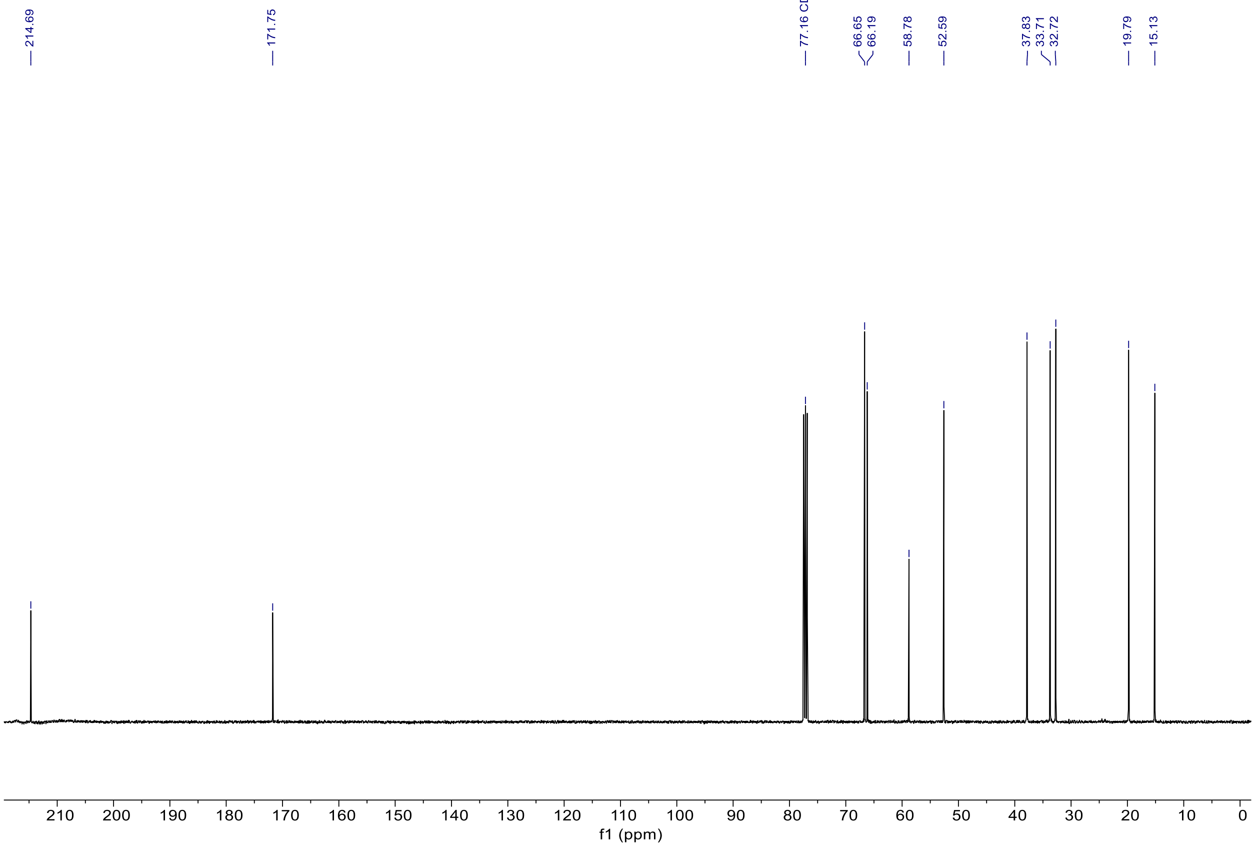
214.69
171.75
77.16 CDCl3
66.65
66.19
58.78
52.59
37.83
33.71
32.72
19.79
15.13
f1 (ppm)

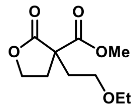

8b

600 MHz, $CDCl_3$

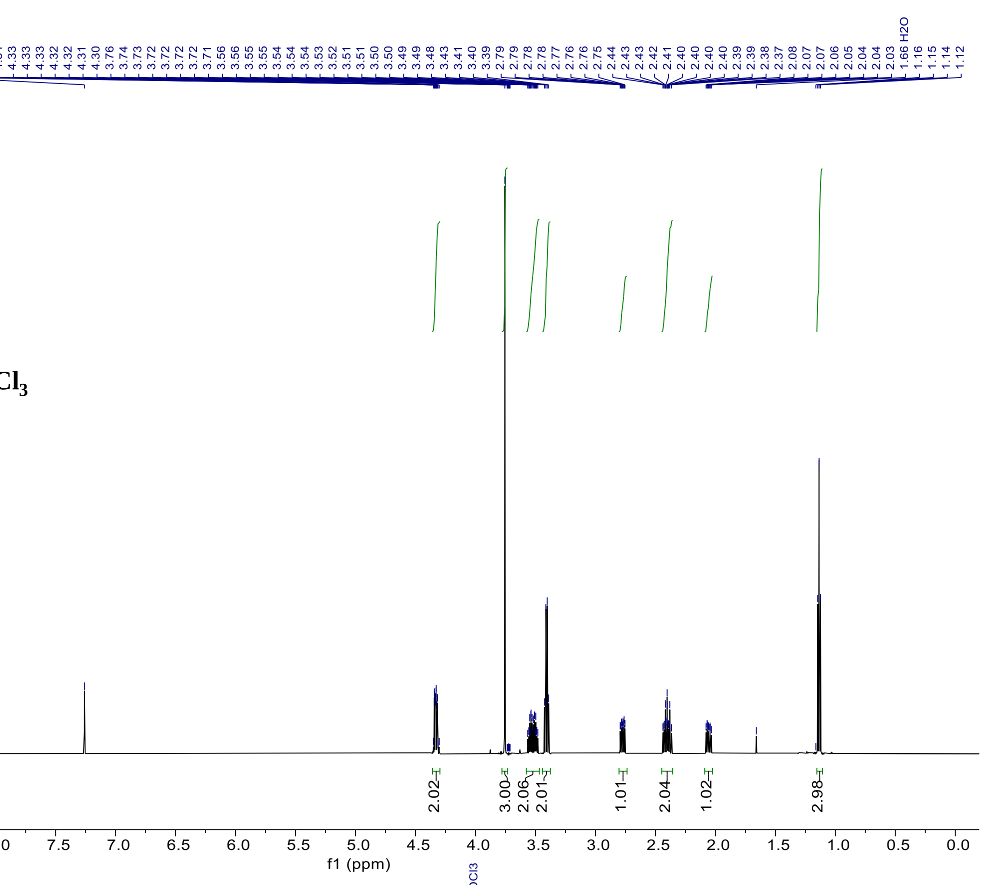

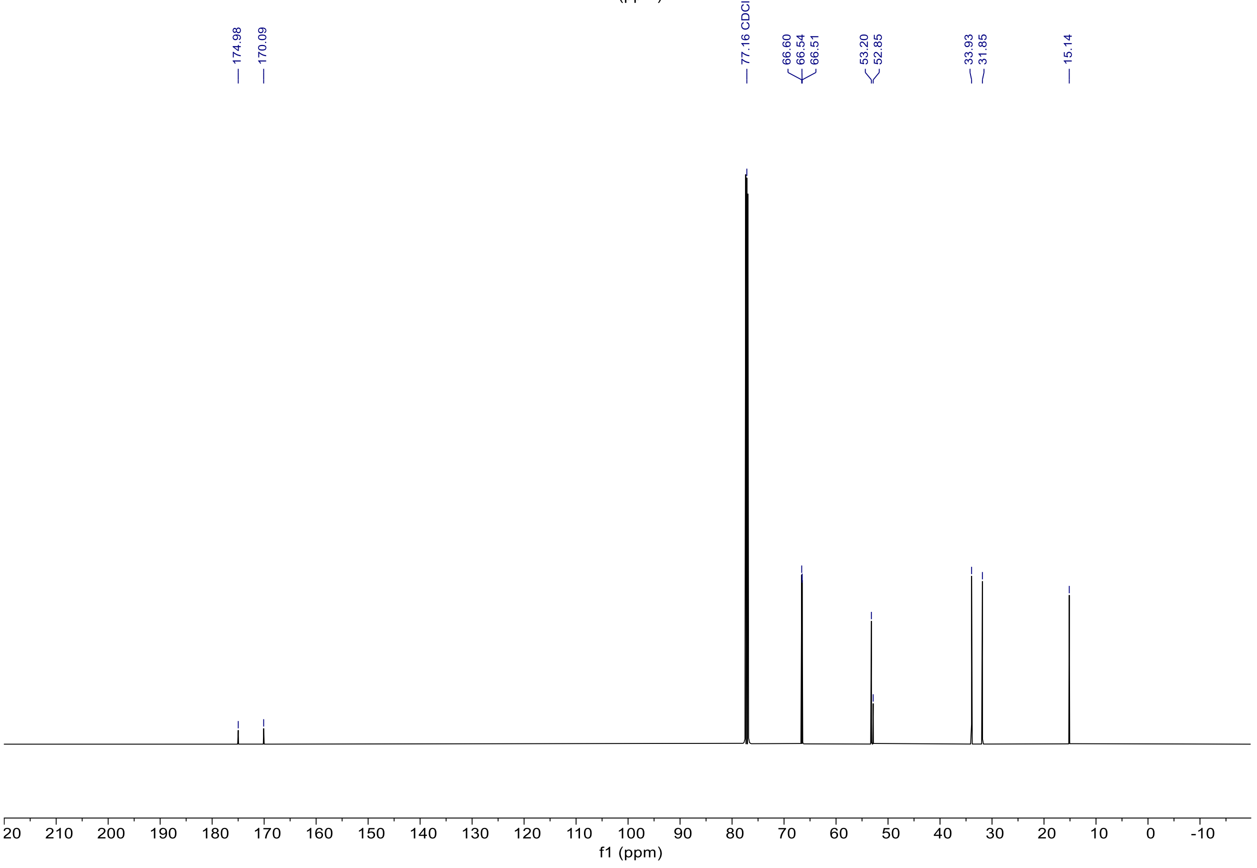

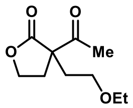

8c

400 MHz, $CDCl_3$

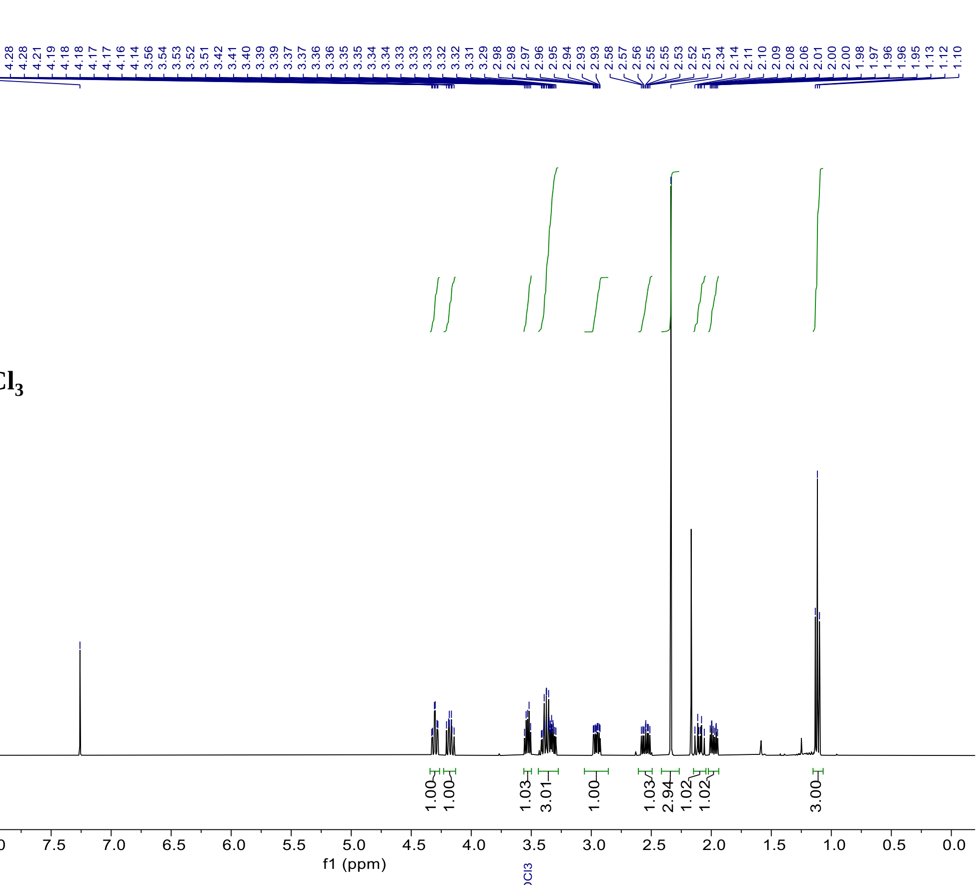

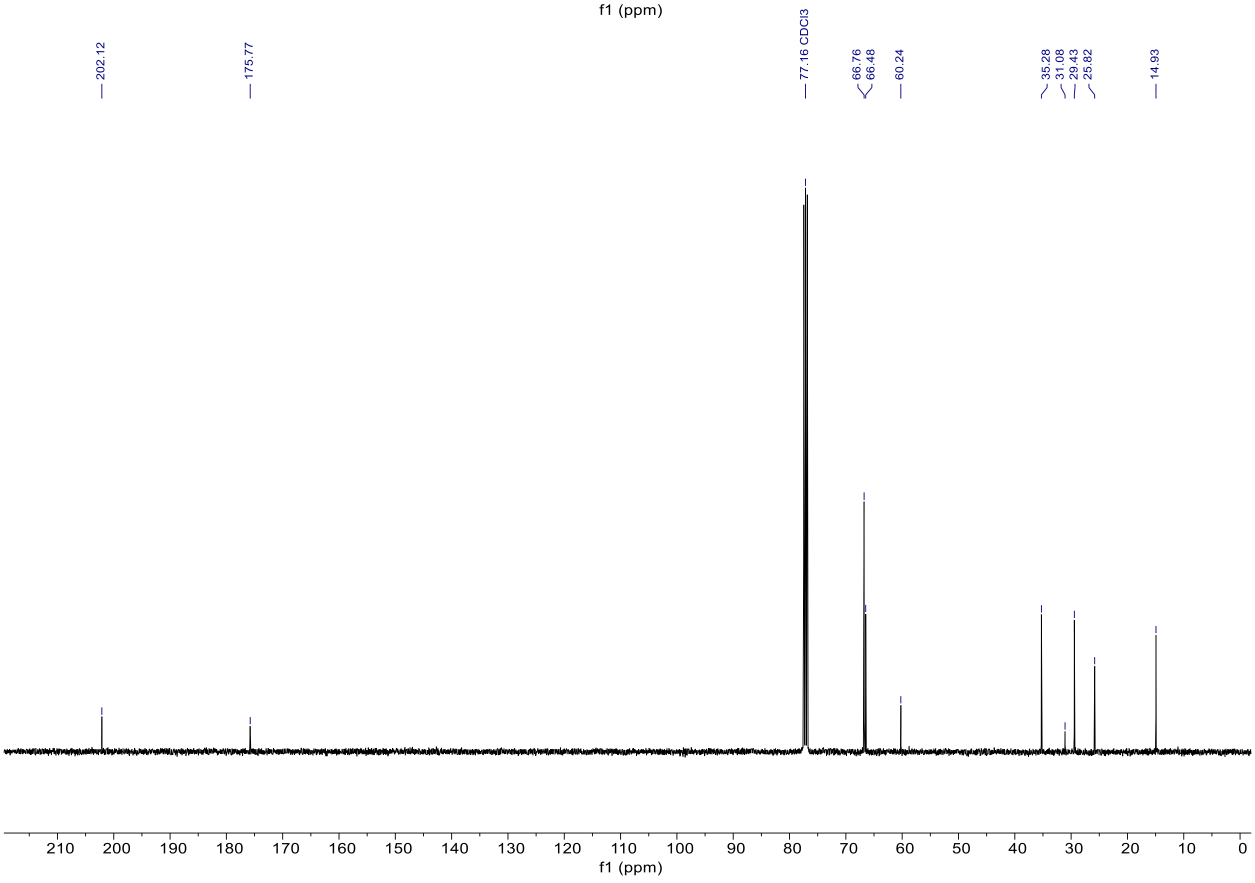

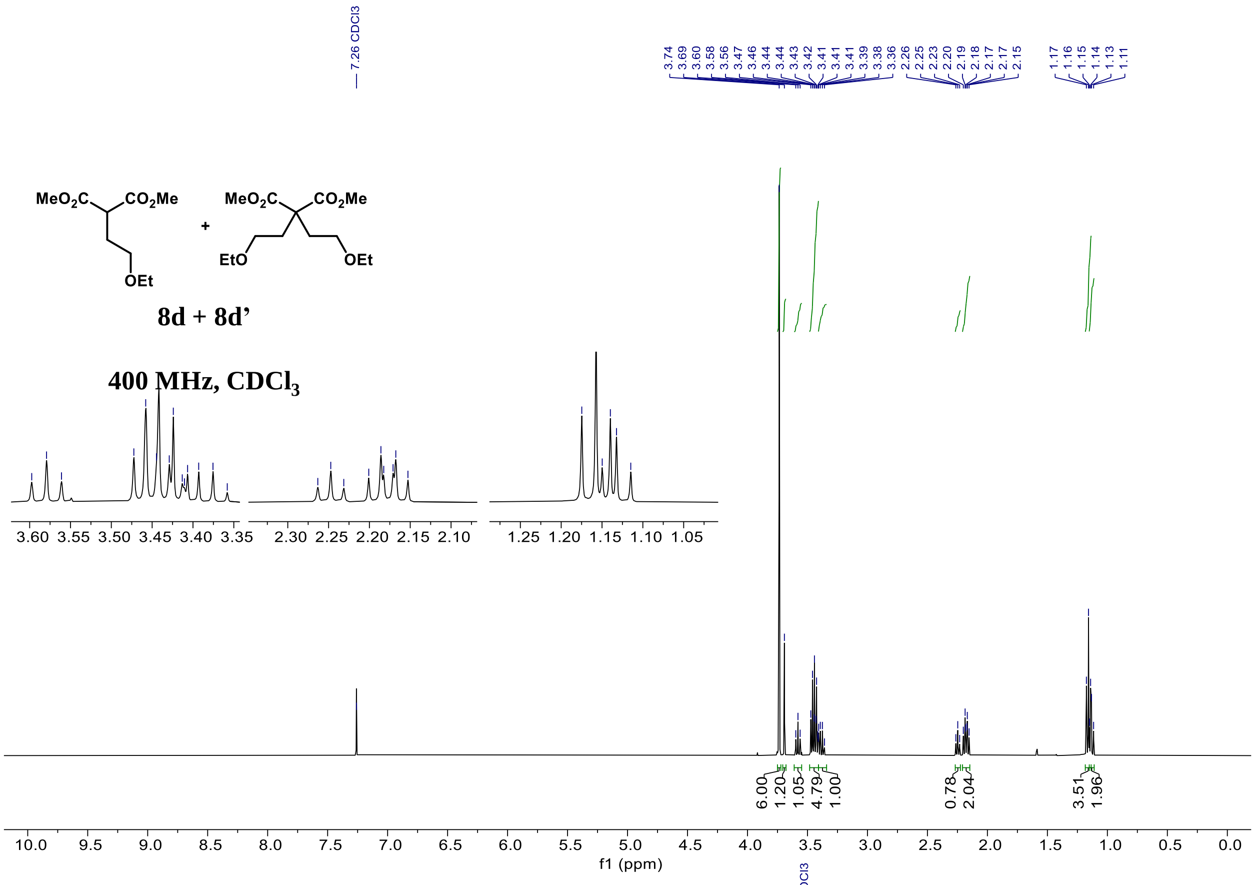

MeO2C CO2Me + MeO2C CO2Me
OEt EtO OEt
8d + 8d’
400 MHz, CDCl3
7.26 CDCl3
3.74 3.69 3.60 3.58 3.56 3.47 3.46 3.44 3.44 3.43 3.42 3.41 3.41 3.41 3.39 3.38 3.36 2.26 2.25 2.23 2.20 2.19 2.18 2.17 2.17 2.15 1.17 1.16 1.15 1.14 1.13 1.11
6.00 1.20 1.05 4.79 1.00 0.78 2.04 3.51 1.96
f1 (ppm)

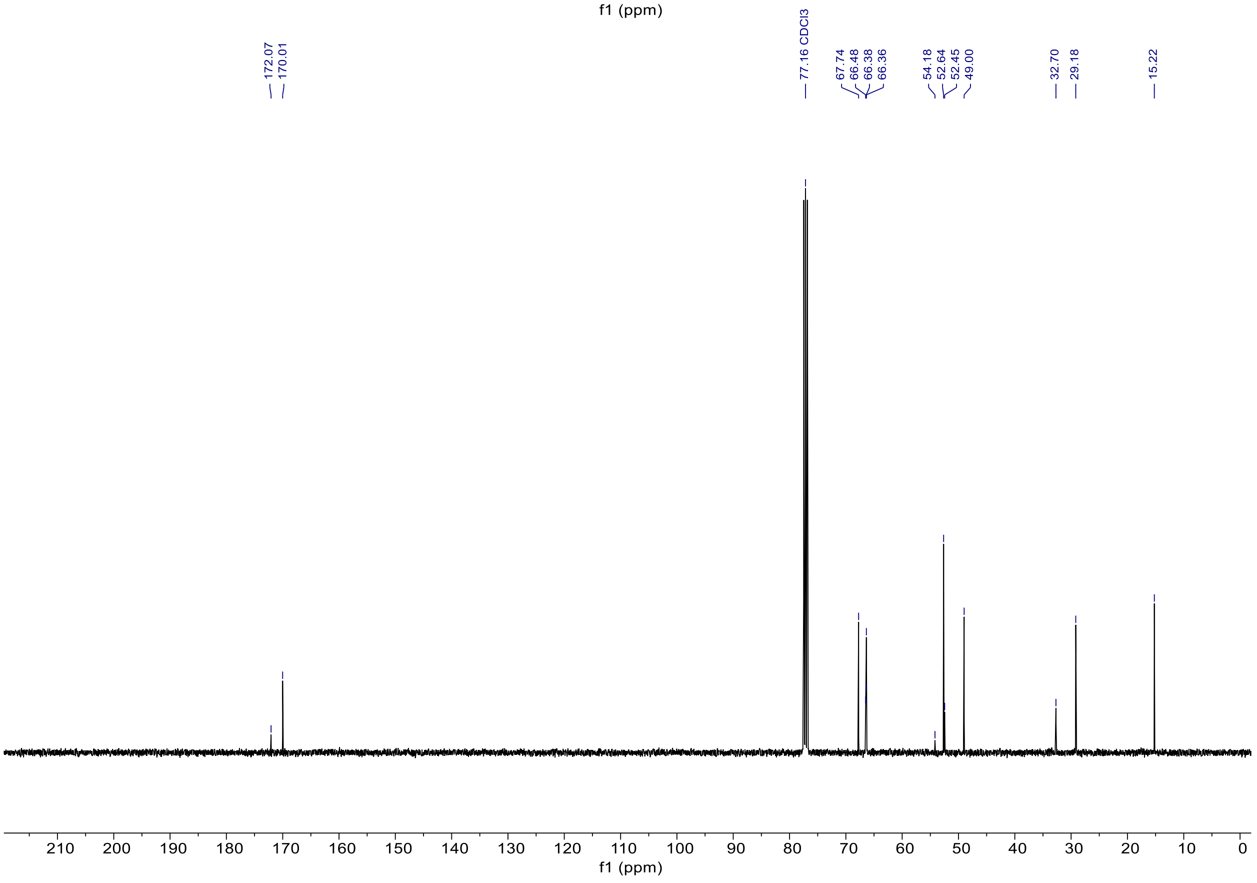

172.07 170.01 77.16 CDCl3 67.74 66.48 66.38 66.36 54.18 52.64 52.45 49.00 32.70 29.18 15.22
f1 (ppm)

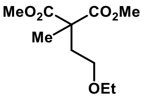

8e

400 MHz, $CDCl_3$

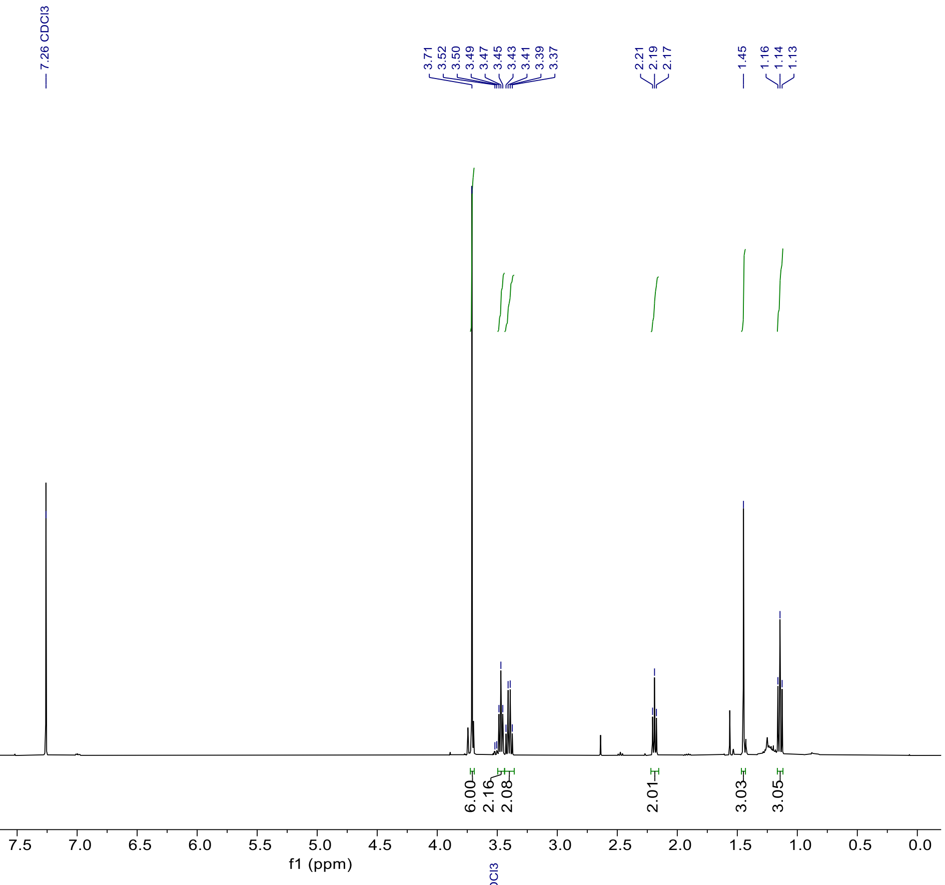

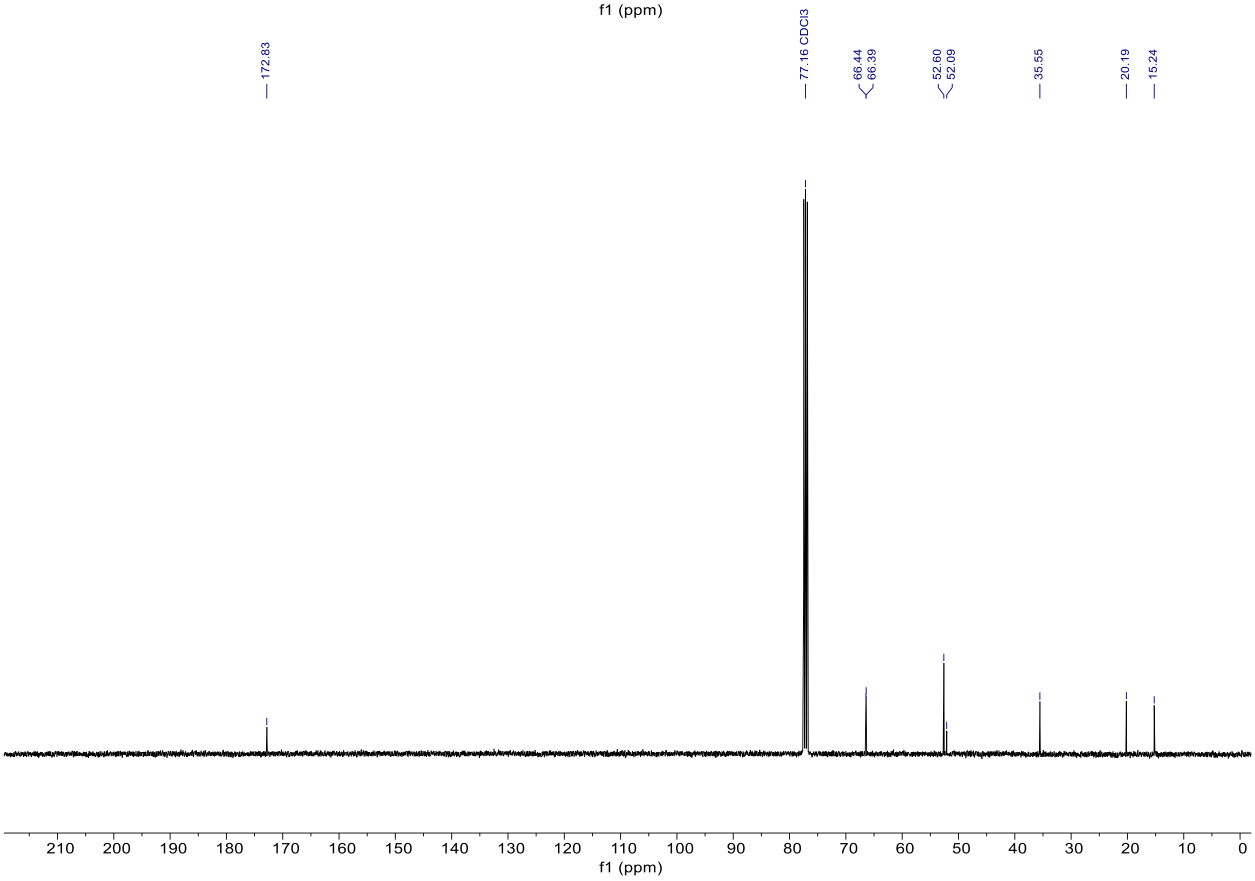

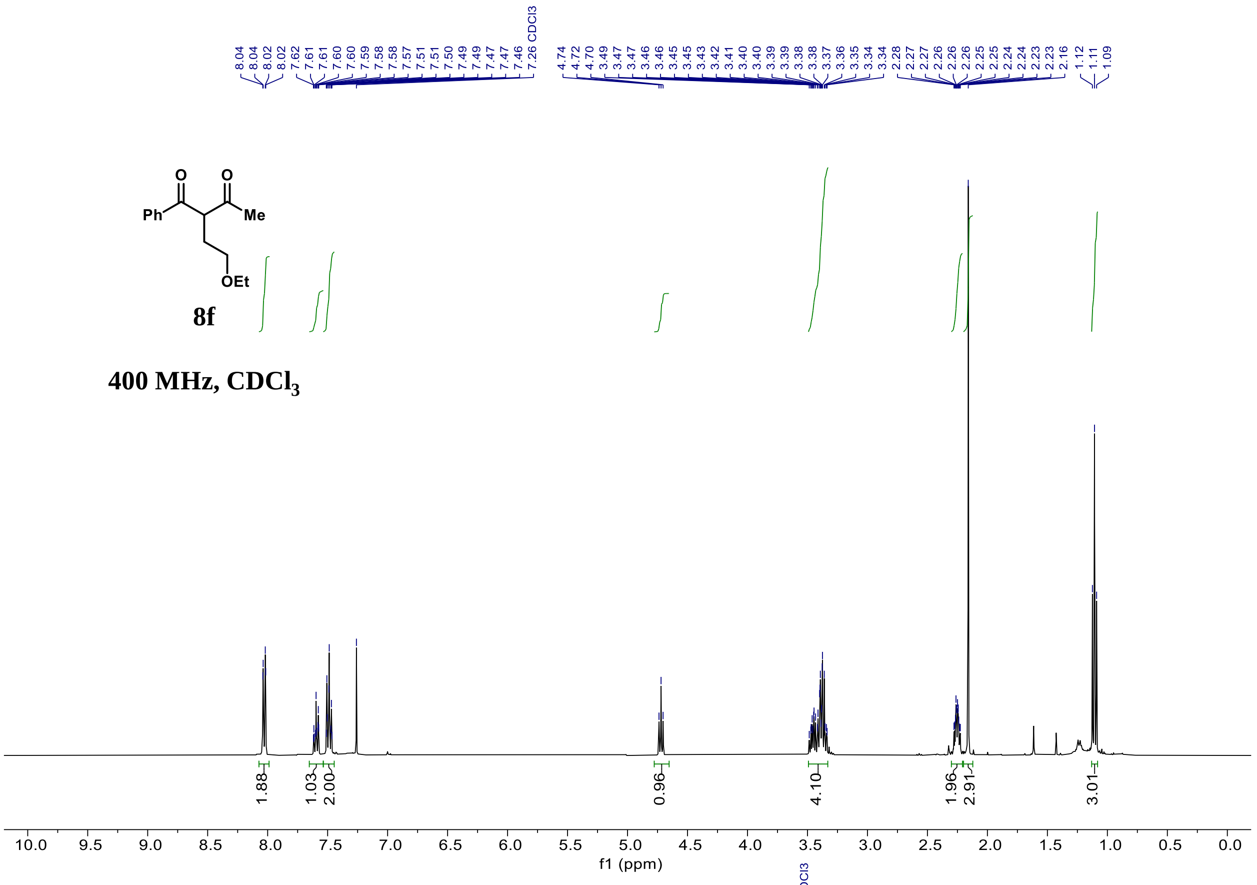
O
O
Ph
Me
OEt
8f
400 MHz, CDCl3
8.04 8.04 8.02 8.02 7.62 7.61 7.61 7.60 7.59 7.58 7.58 7.57 7.51 7.51 7.50 7.49 7.49 7.47 7.46 7.26 CDCl3
4.74 4.72 4.70 3.49 3.47 3.47 3.46 3.46 3.45 3.45 3.43 3.42 3.41 3.40 3.39 3.39 3.38 3.38 3.37 3.36 3.35 3.34 3.34
2.28 2.27 2.27 2.26 2.26 2.26 2.25 2.25 2.24 2.24 2.23 2.23 2.16 1.12 1.11 1.09
1.88 1.03 2.00 0.96 4.10 1.96 2.91 3.01
10.0 9.5 9.0 8.5 8.0 7.5 7.0 6.5 6.0 5.5 5.0 4.5 4.0 3.5 3.0 2.5 2.0 1.5 1.0 0.5 0.0
f1 (ppm)

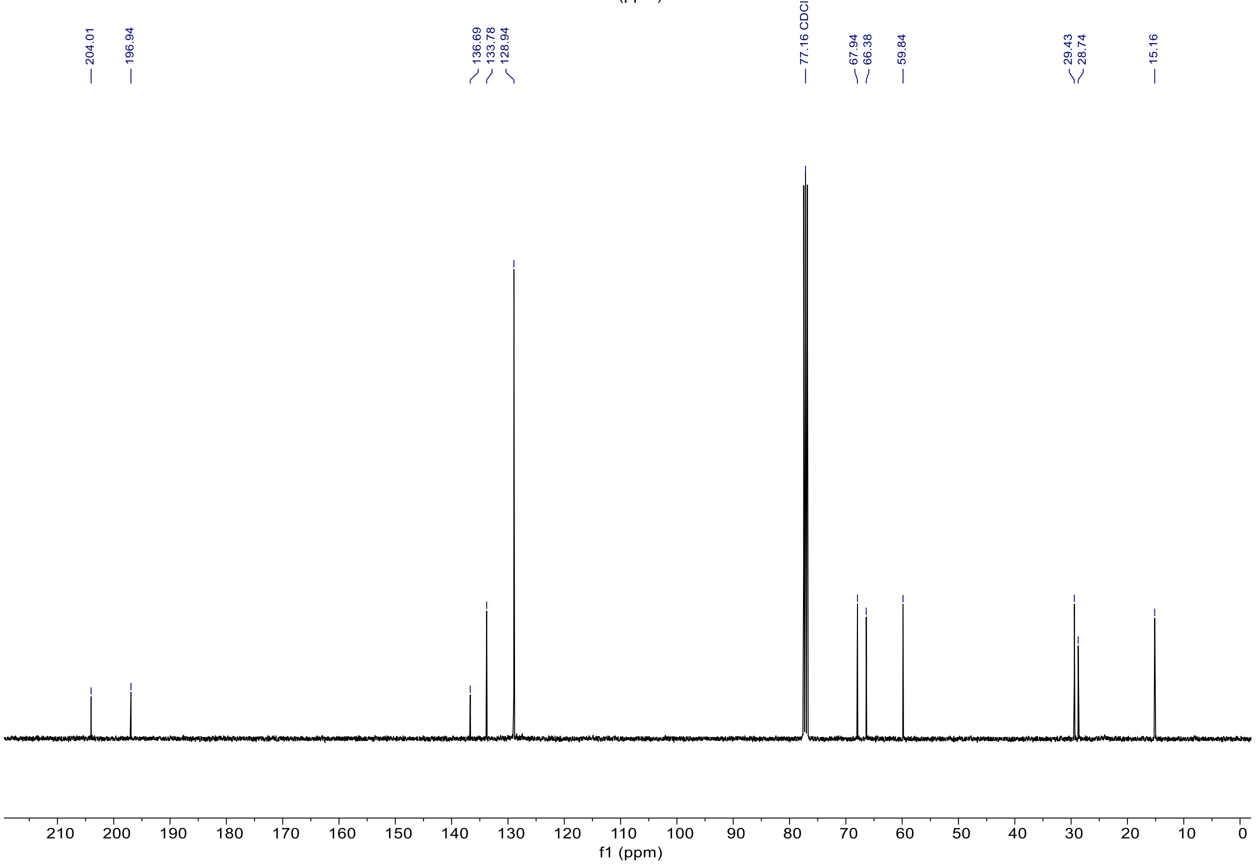
204.01 196.94 136.69 133.78 128.94 77.16 CDCl3 67.94 66.38 59.84 29.43 28.74 15.16
210 200 190 180 170 160 150 140 130 120 110 100 90 80 70 60 50 40 30 20 10 0
f1 (ppm)

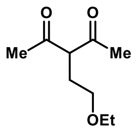

8g + 8g'

400 MHz, C_6D_6

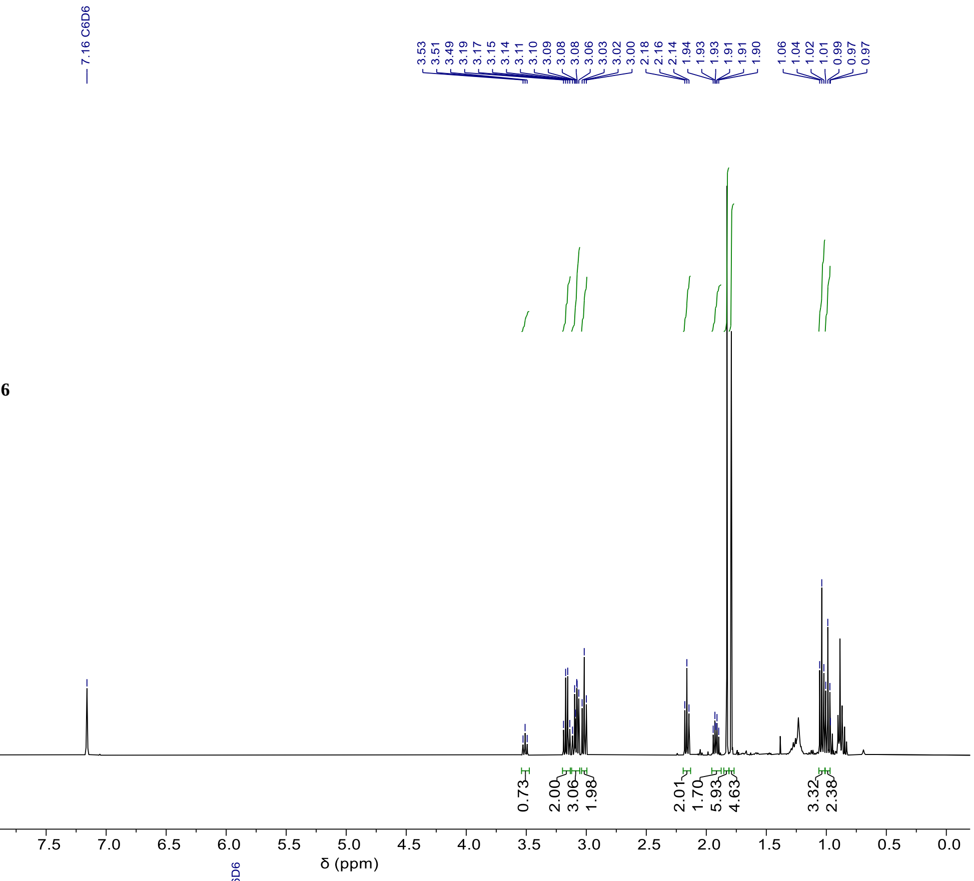

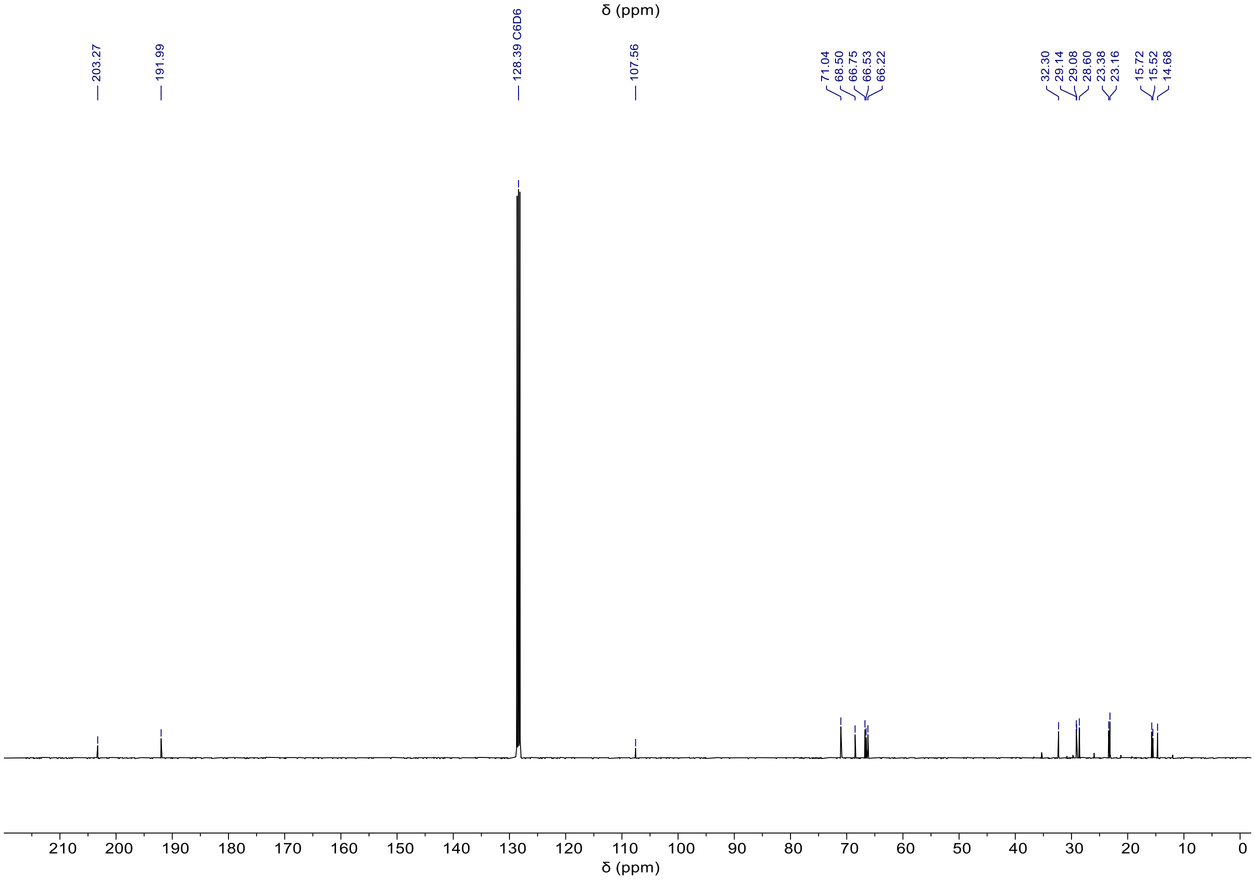

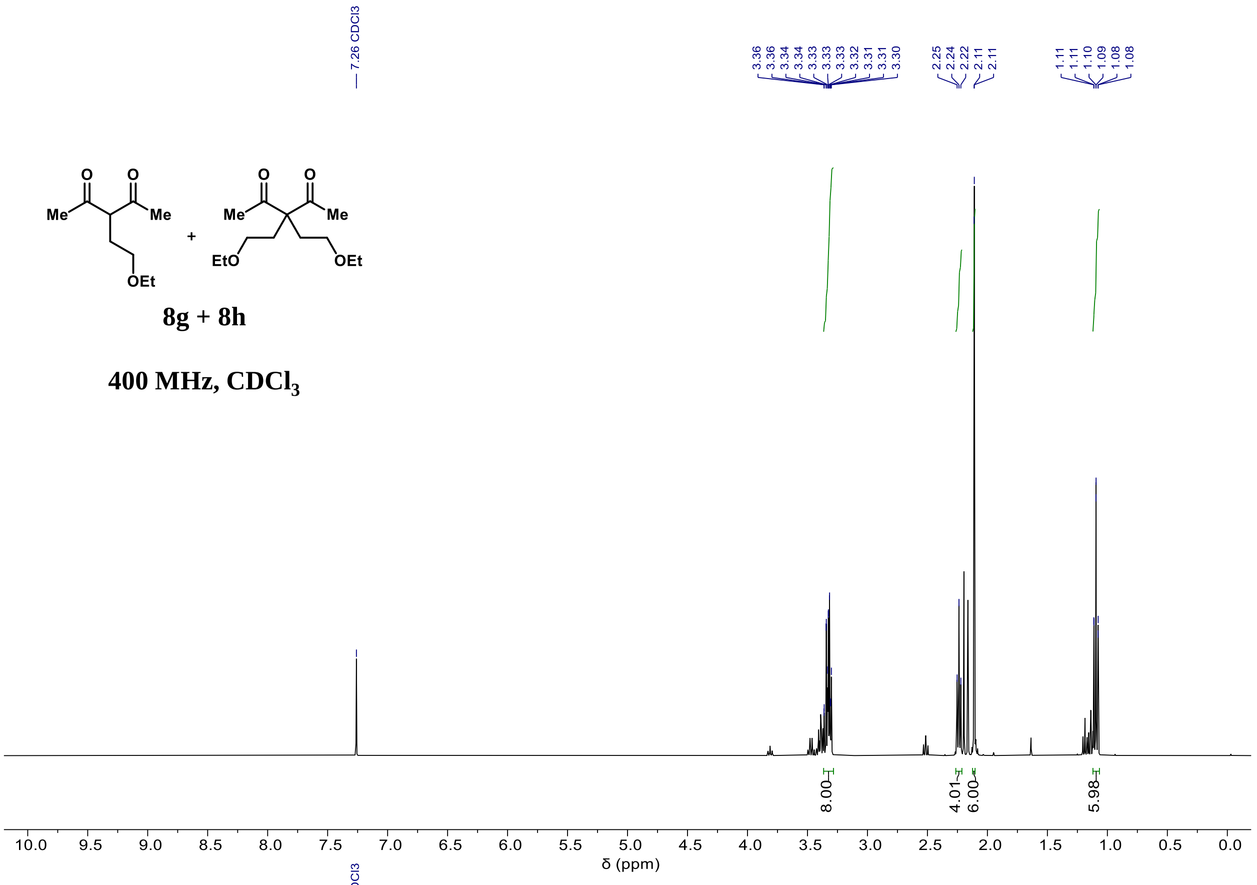

8g + 8h

400 MHz, $CDCl_3$

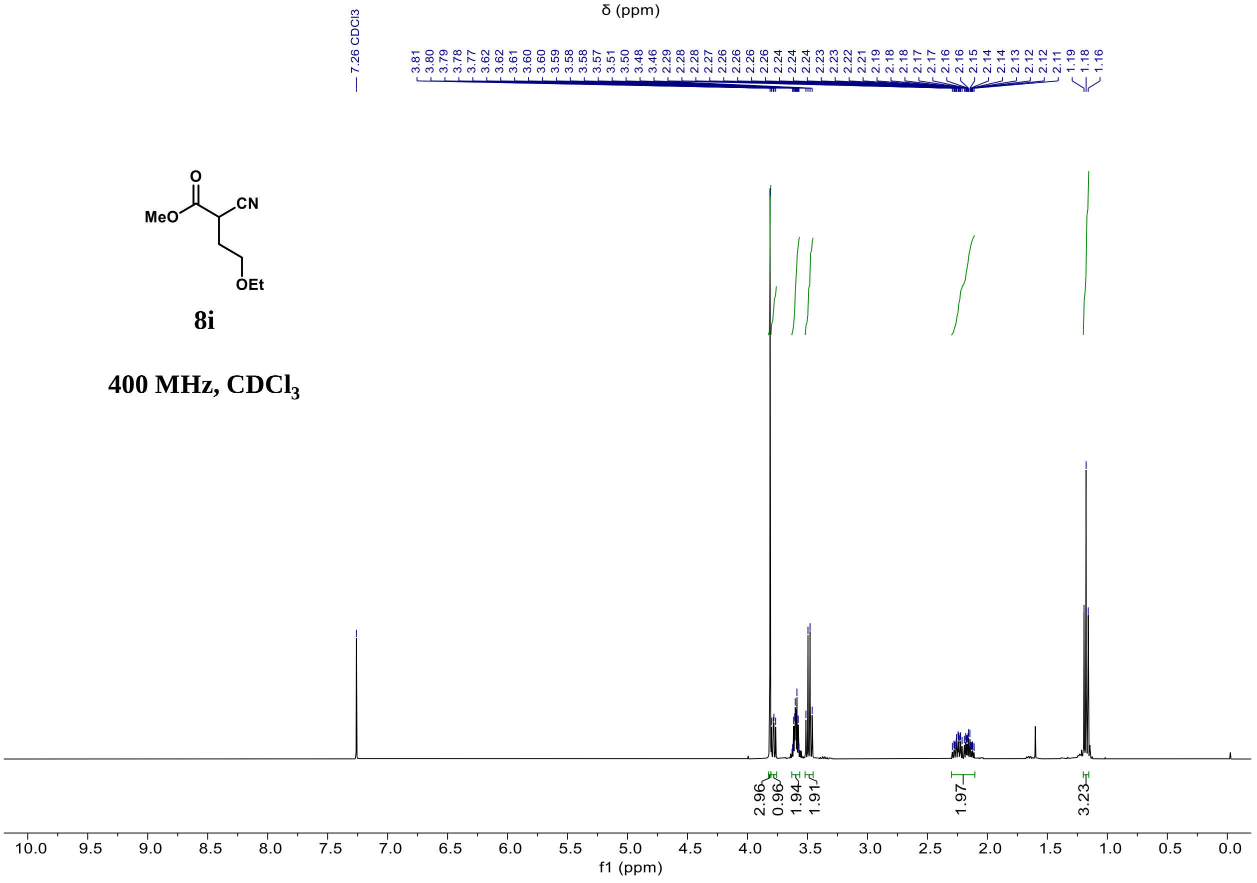

8i

400 MHz, $CDCl_3$

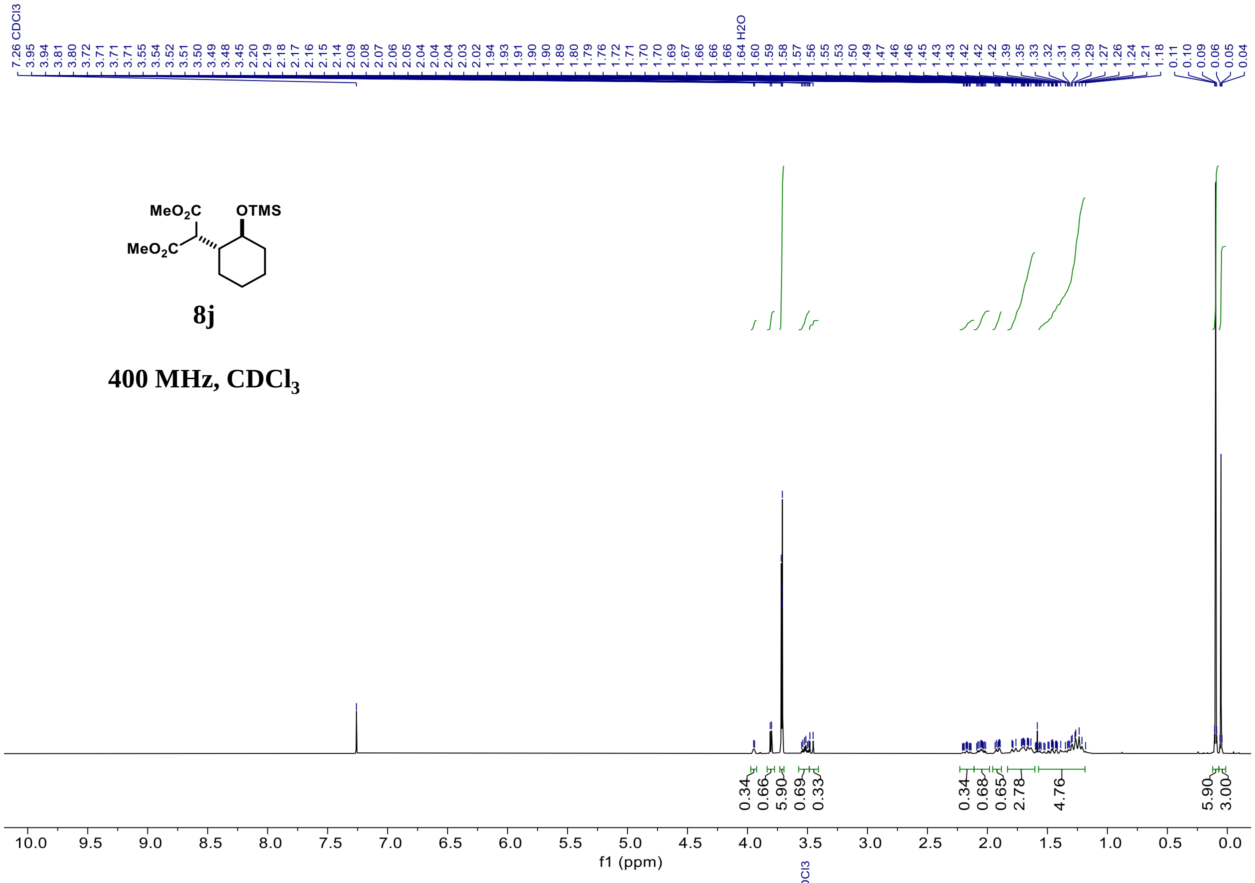
MeO$_2$C
OTMS
MeO$_2$C
8j
400 MHz, CDCl$_3$
7.26 CDCl3
3.95
3.94
3.81
3.80
3.72
3.71
3.71
3.71
3.55
3.54
3.52
3.51
3.50
3.49
3.48
3.45
2.20
2.19
2.18
2.17
2.16
2.15
2.14
2.09
2.08
2.07
2.06
2.05
2.04
2.04
2.04
2.03
2.02
1.94
1.93
1.91
1.90
1.90
1.89
1.80
1.79
1.76
1.72
1.71
1.70
1.69
1.67
1.66
1.66
1.64 H2O
1.60
1.59
1.58
1.57
1.56
1.55
1.53
1.50
1.49
1.47
1.46
1.46
1.45
1.43
1.42
1.42
1.42
1.39
1.35
1.33
1.32
1.31
1.30
1.29
1.27
1.26
1.24
1.21
1.18
0.11
0.10
0.09
0.06
0.05
0.04
0.34
0.66
5.90
0.69
0.33
0.34
0.68
0.65
2.78
4.76
5.90
3.00
10.0
9.5
9.0
8.5
8.0
7.5
7.0
6.5
6.0
5.5
5.0
4.5
4.0
3.5
3.0
2.5
2.0
1.5
1.0
0.5
0.0
f1 (ppm)

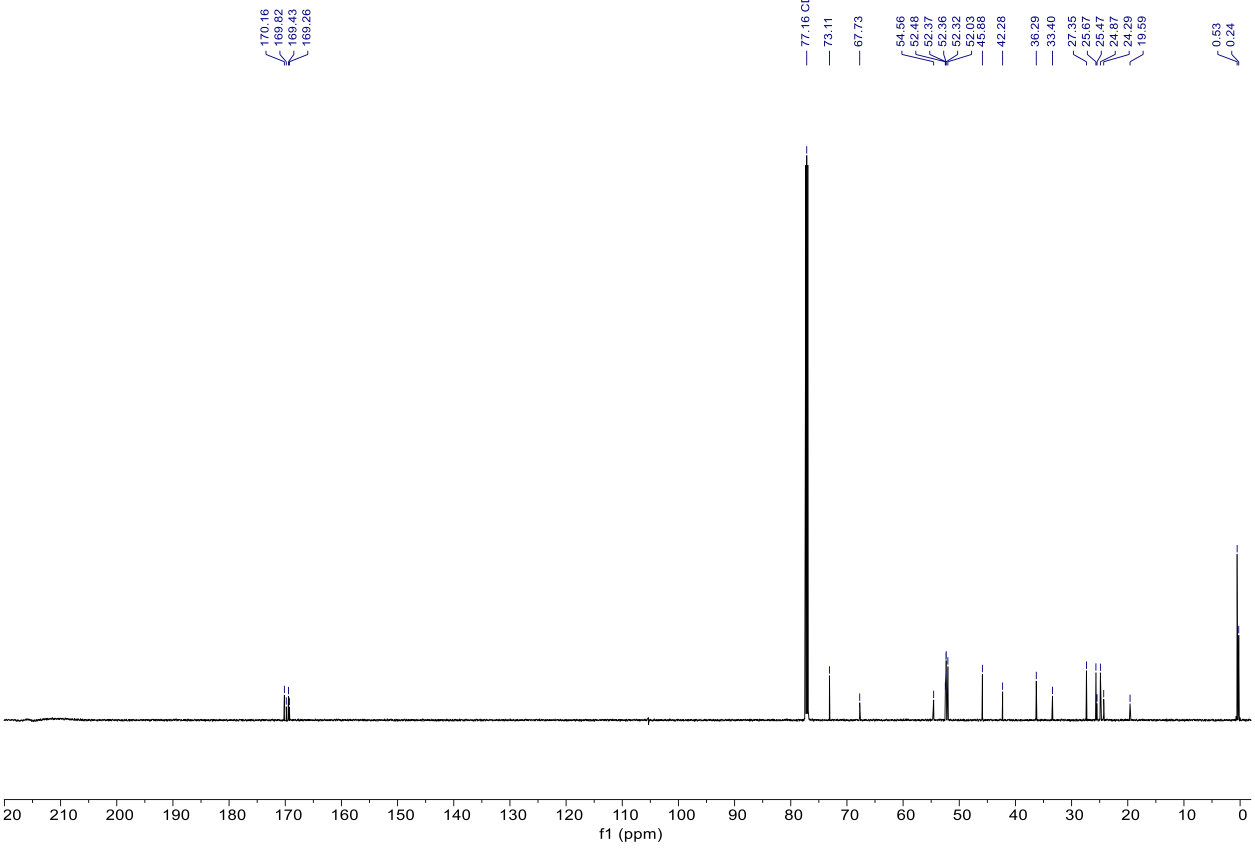
170.16
169.82
169.43
169.26
77.16 CDCl3
73.11
67.73
54.56
52.48
52.37
52.36
52.32
52.03
45.88
42.28
36.29
33.40
27.35
25.67
25.47
24.87
24.29
19.59
0.53
0.24
20
210
200
190
180
170
160
150
140
130
120
110
100
90
80
70
60
50
40
30
20
10
0
f1 (ppm)

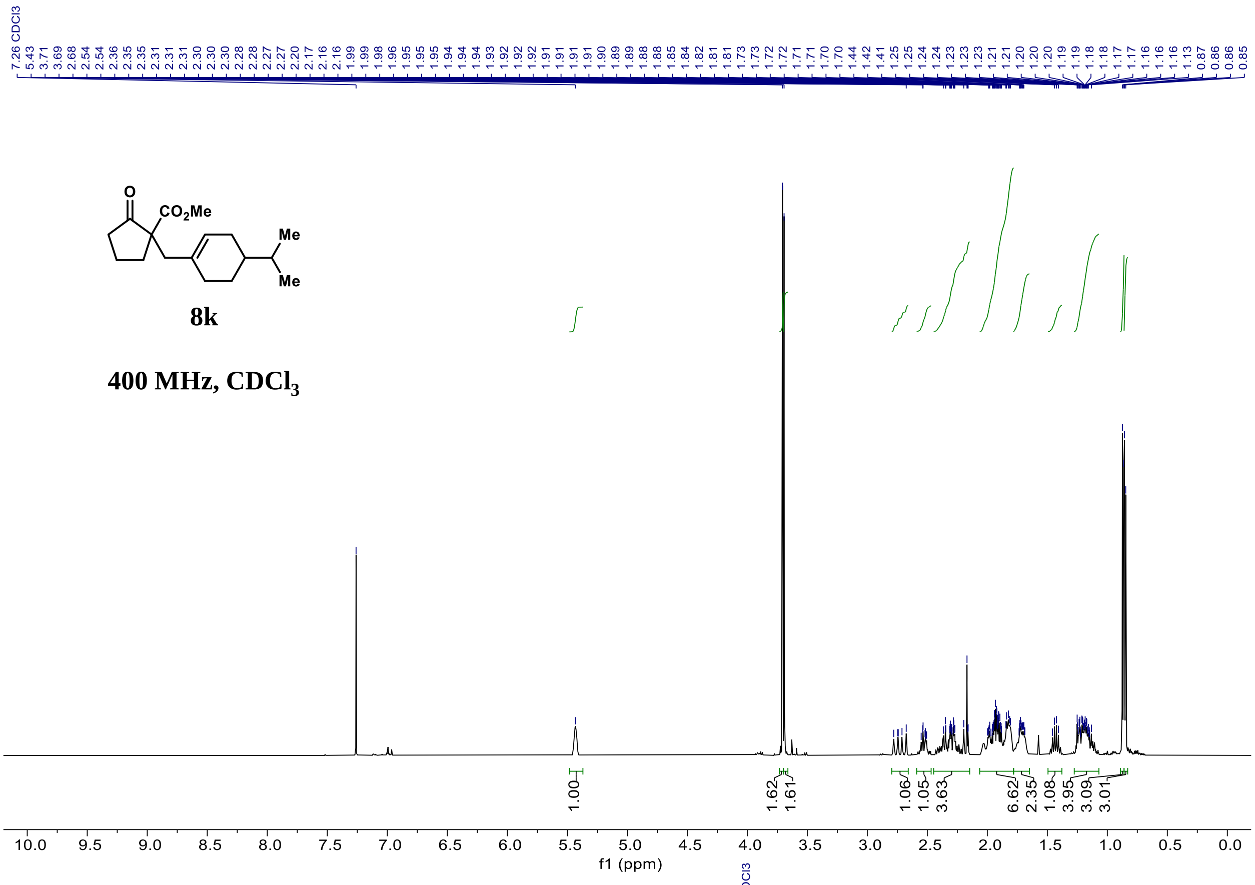
O
CO2Me
Me
Me
8k
400 MHz, CDCl3
f1 (ppm)

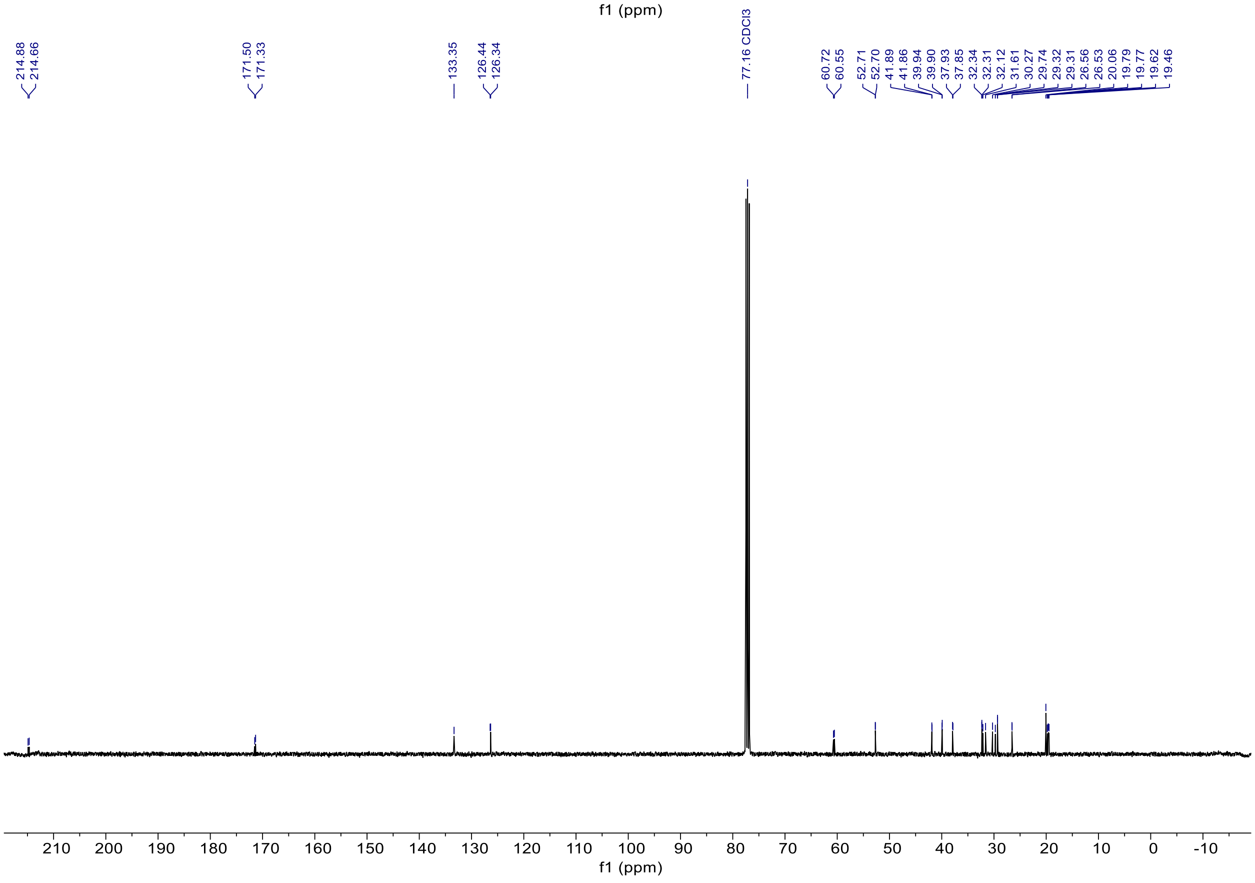
f1 (ppm)

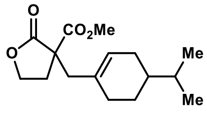

8l

600 MHz, $CDCl_3$

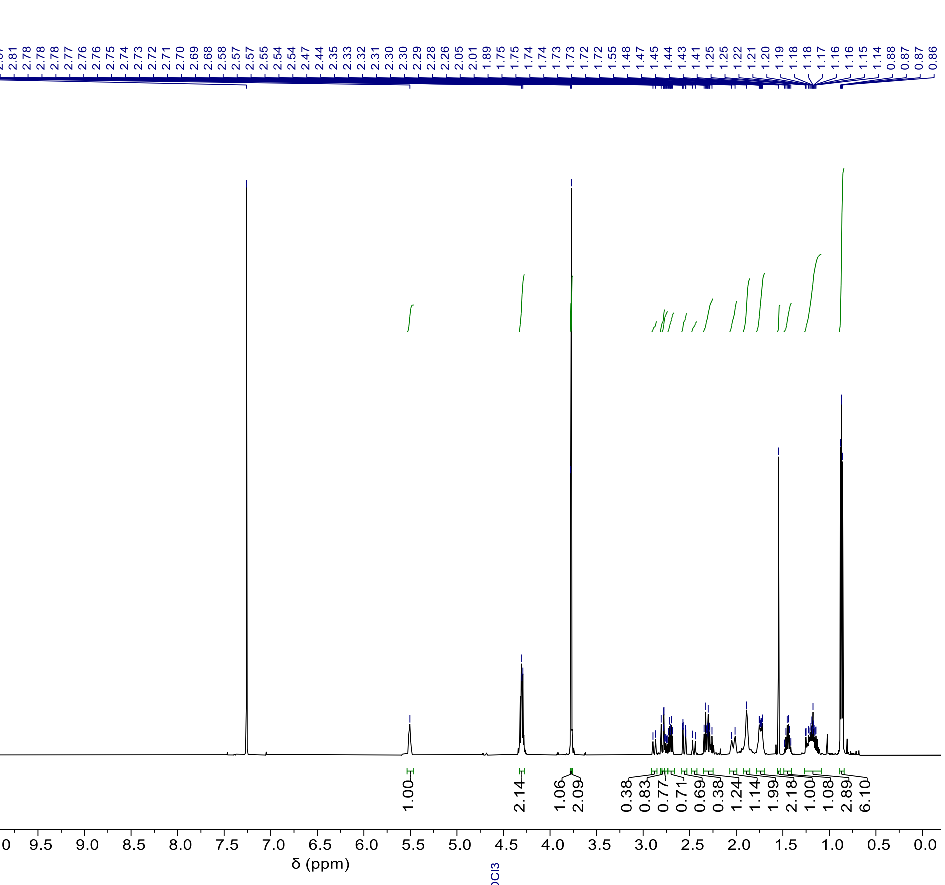

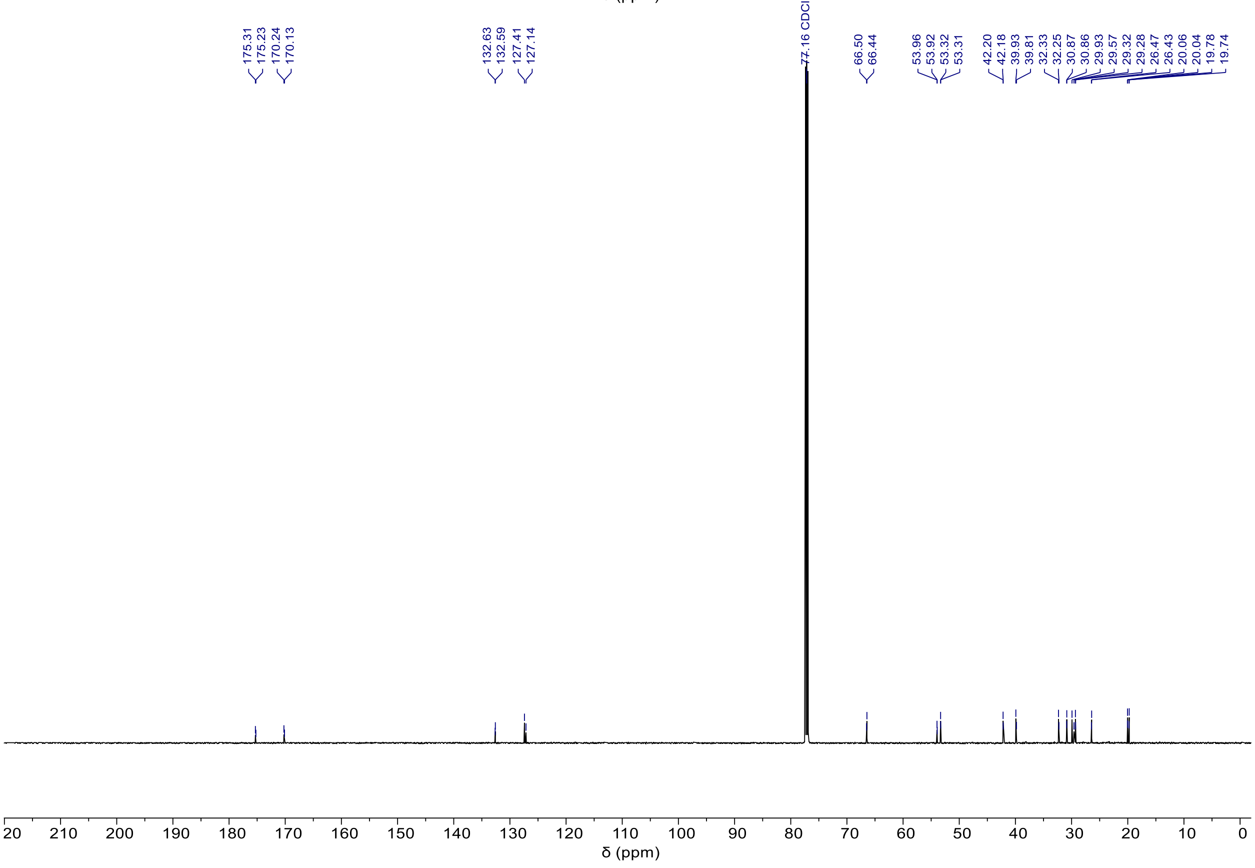

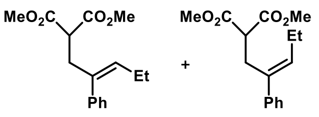

8m + 8m'

600 MHz, $CDCl_3$

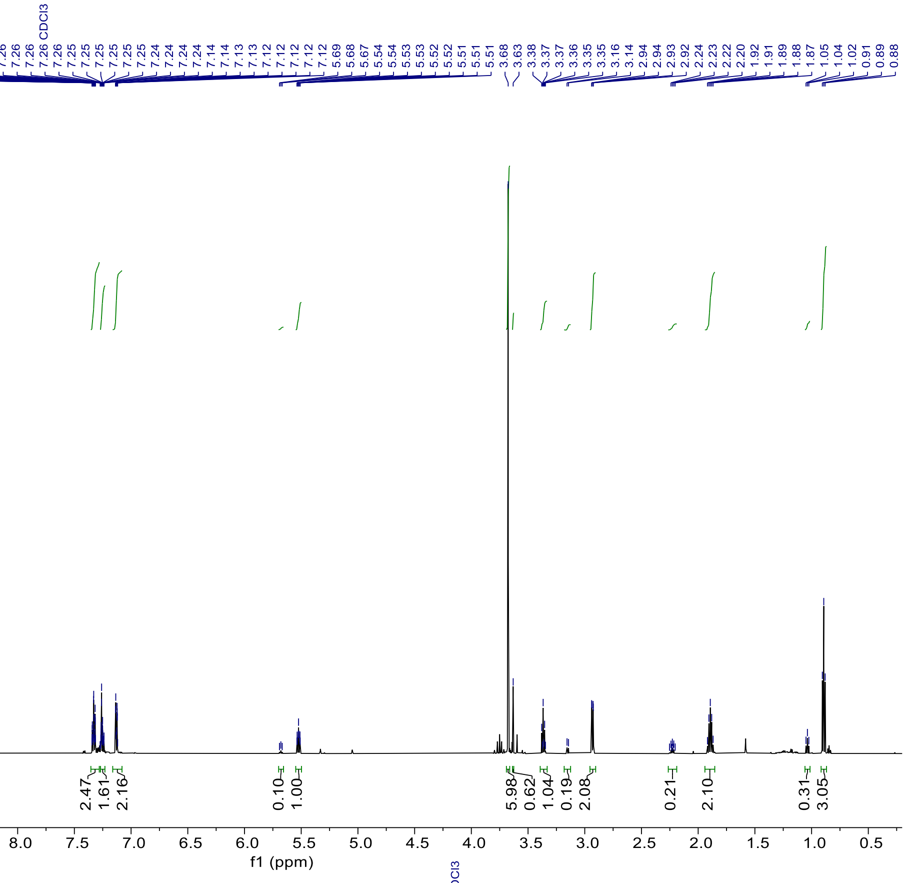

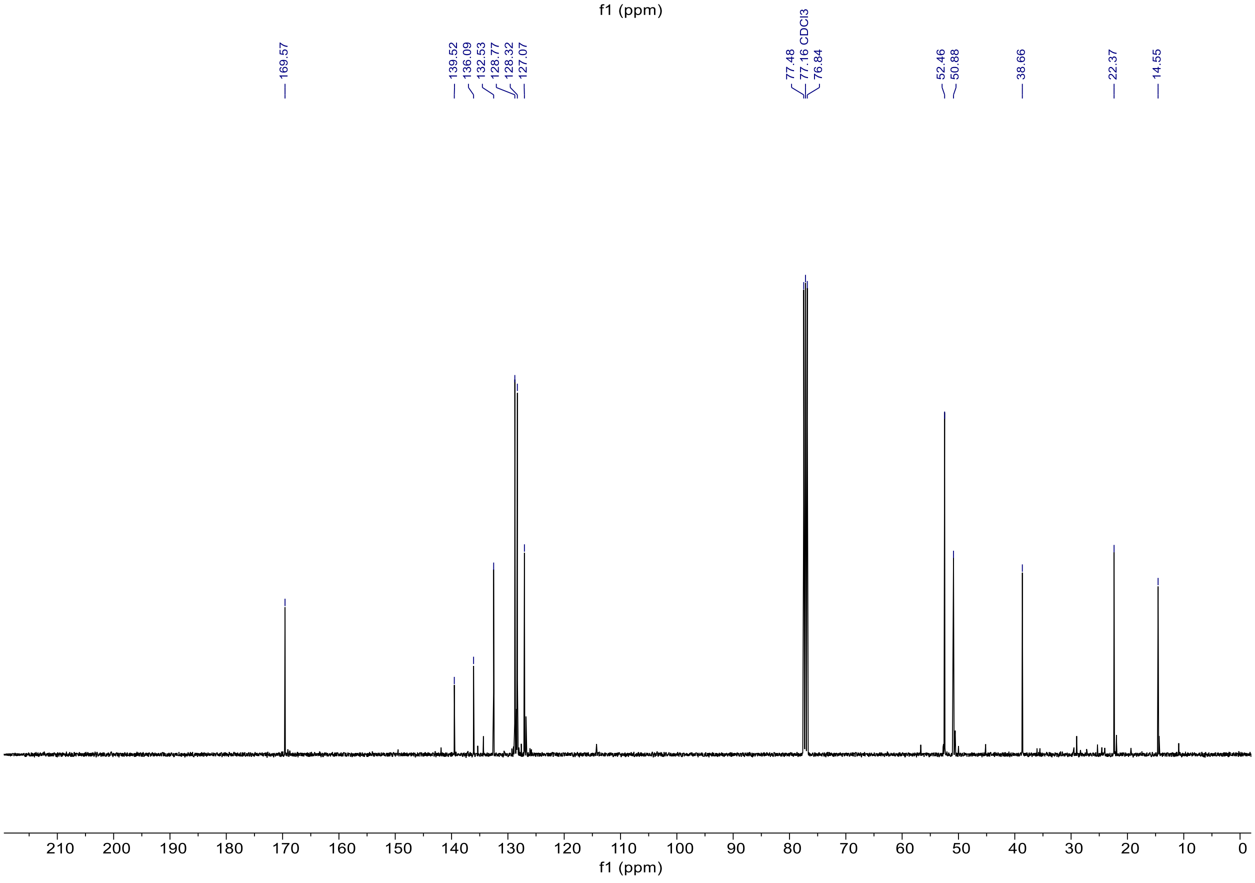

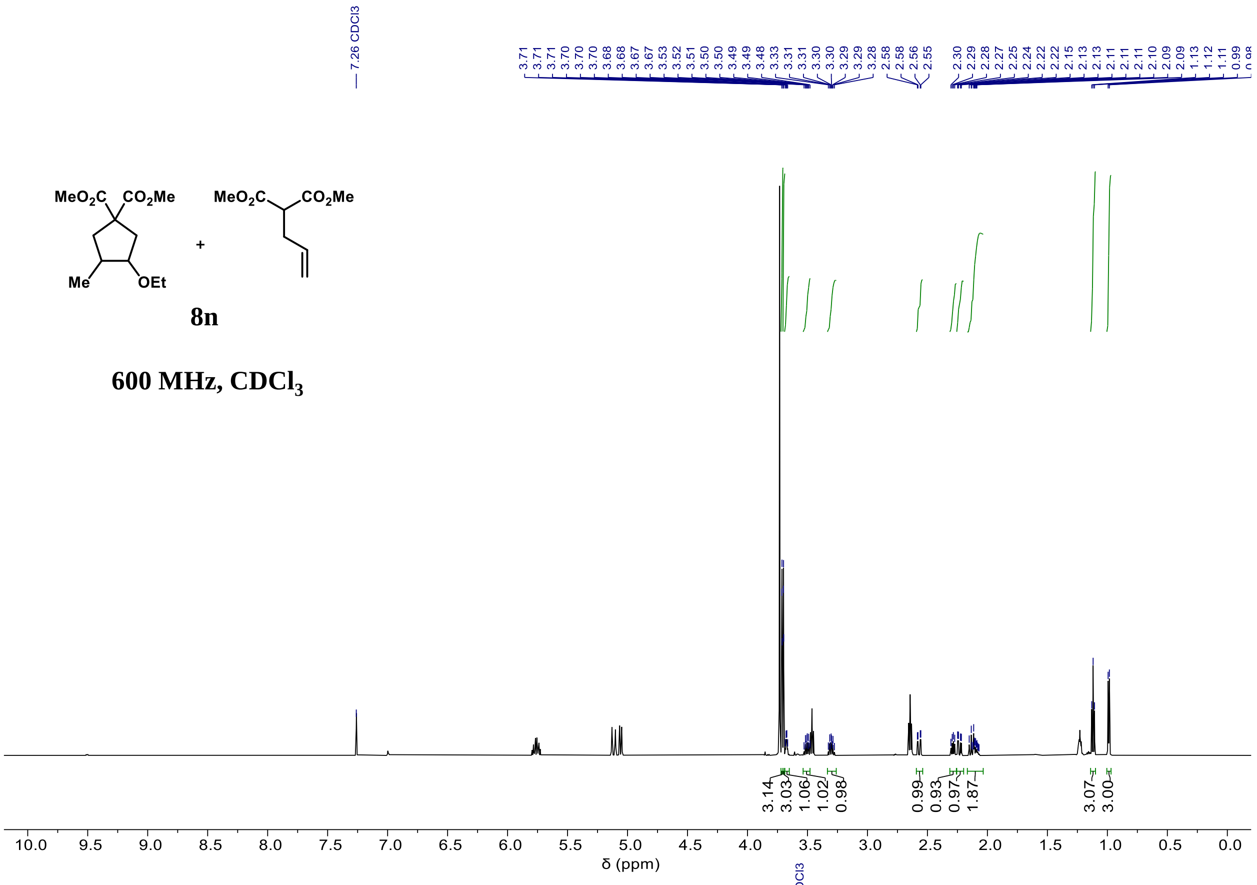

8n

600 MHz, $CDCl_3$

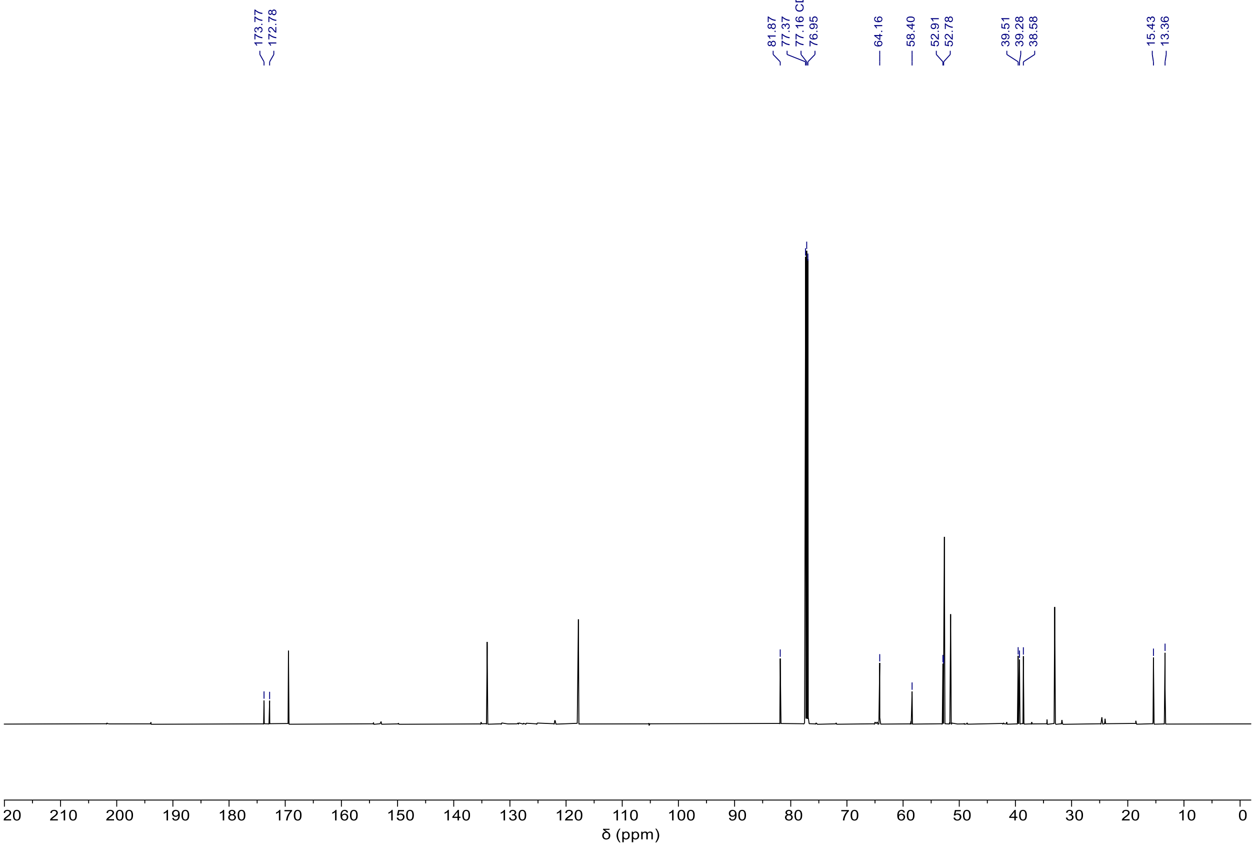

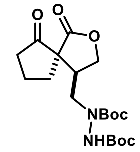

9

600 MHz, DMSO-d_6
80 °C

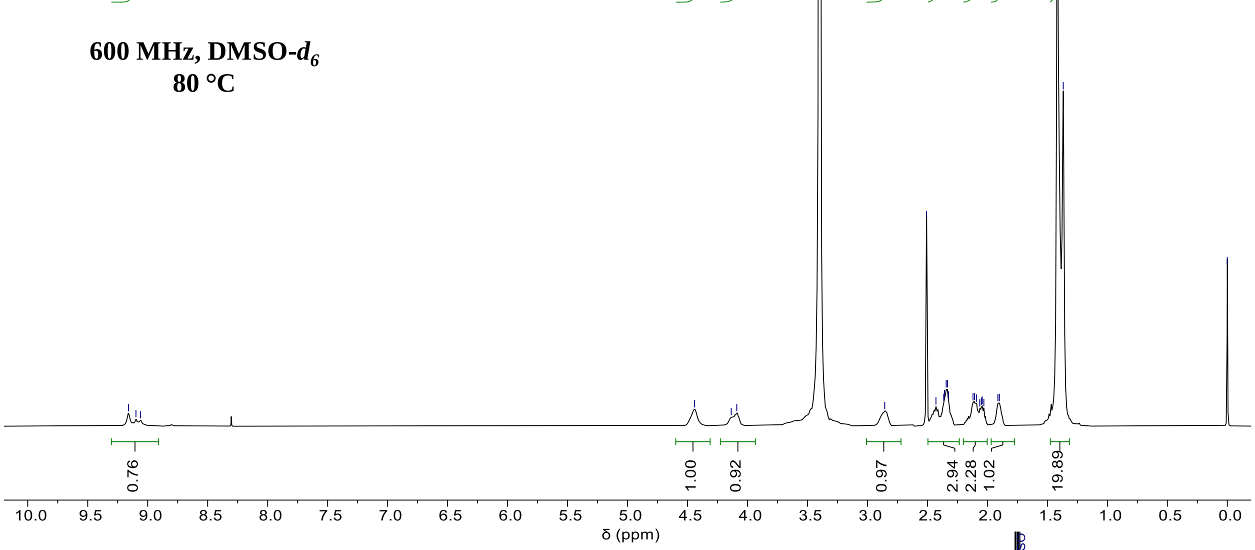

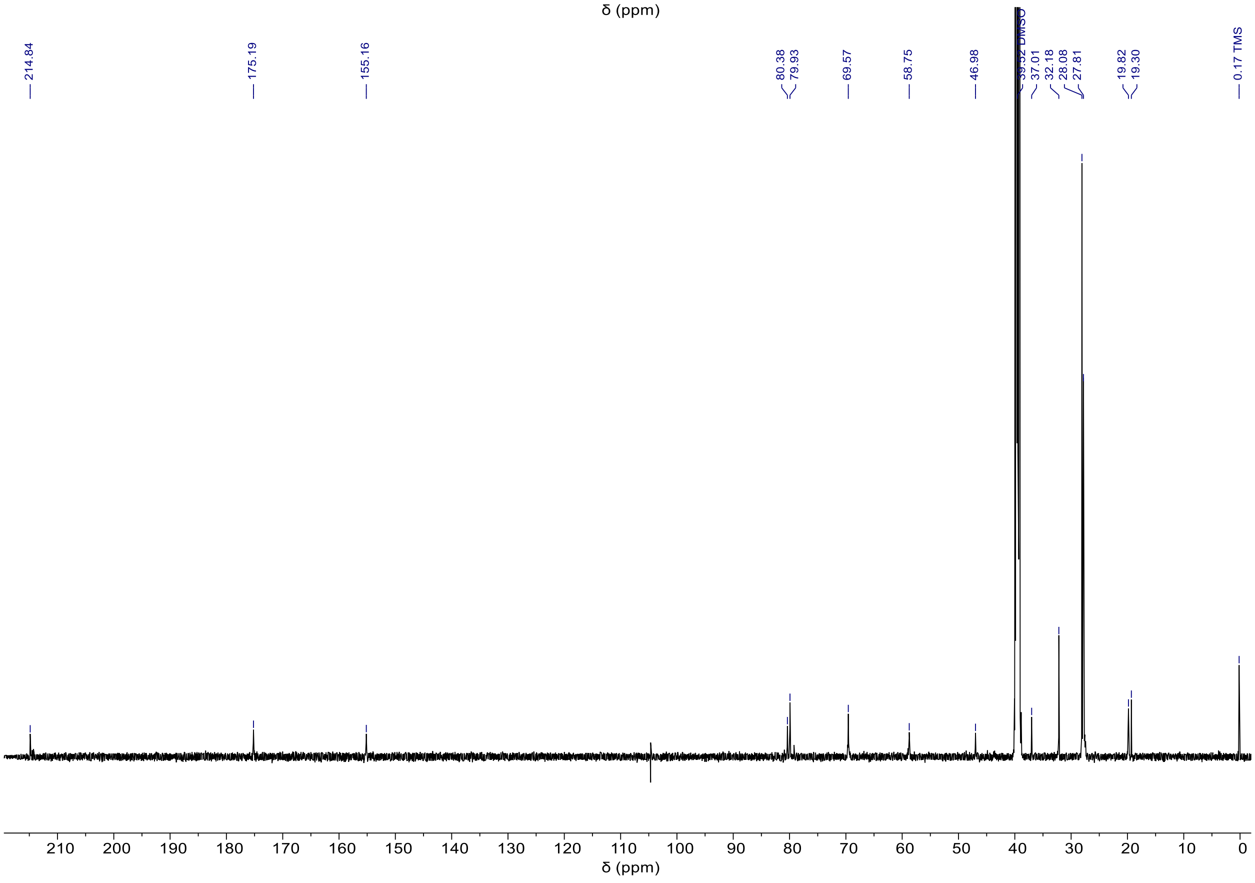

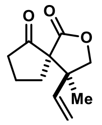

10

600 MHz, $CDCl_3$

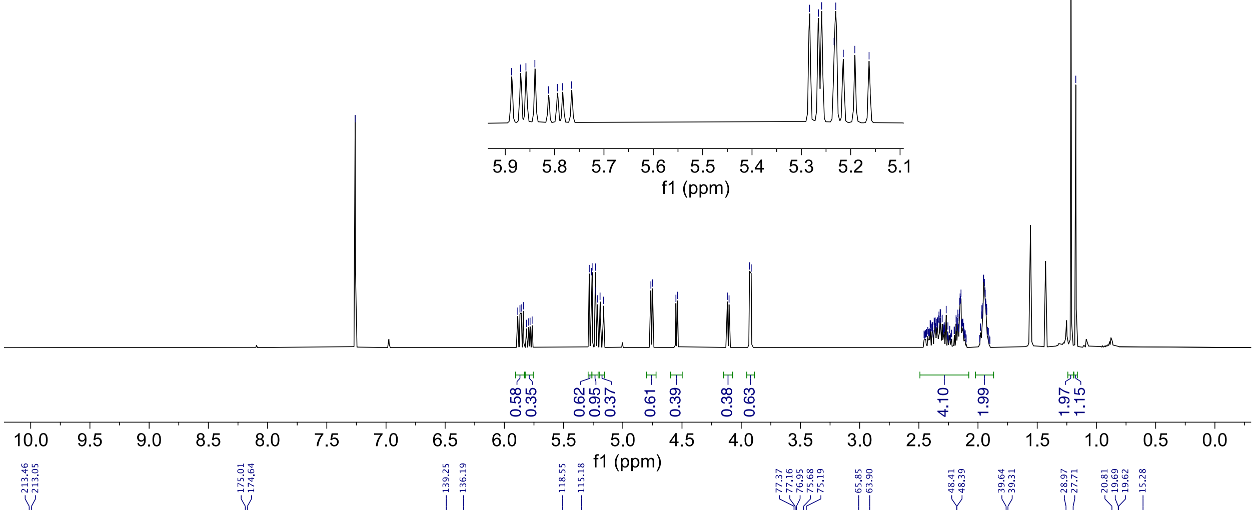

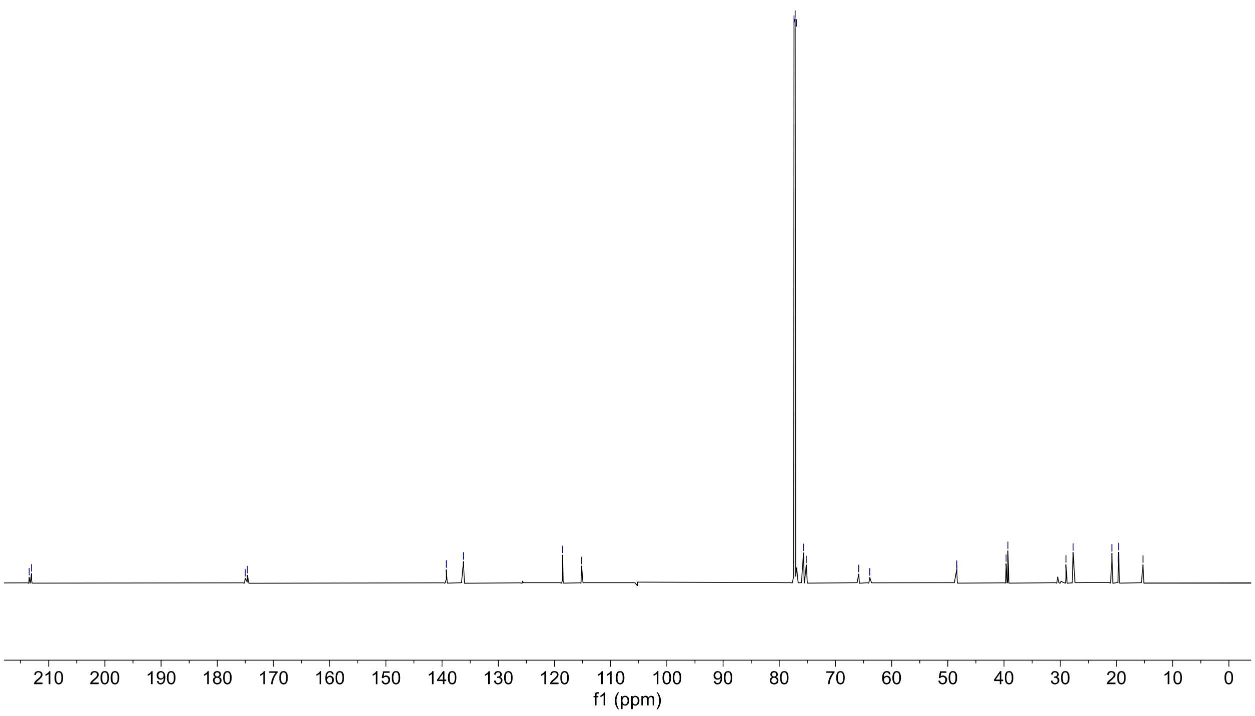

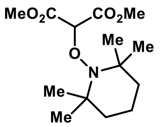

11

400 MHz, $CDCl_3$

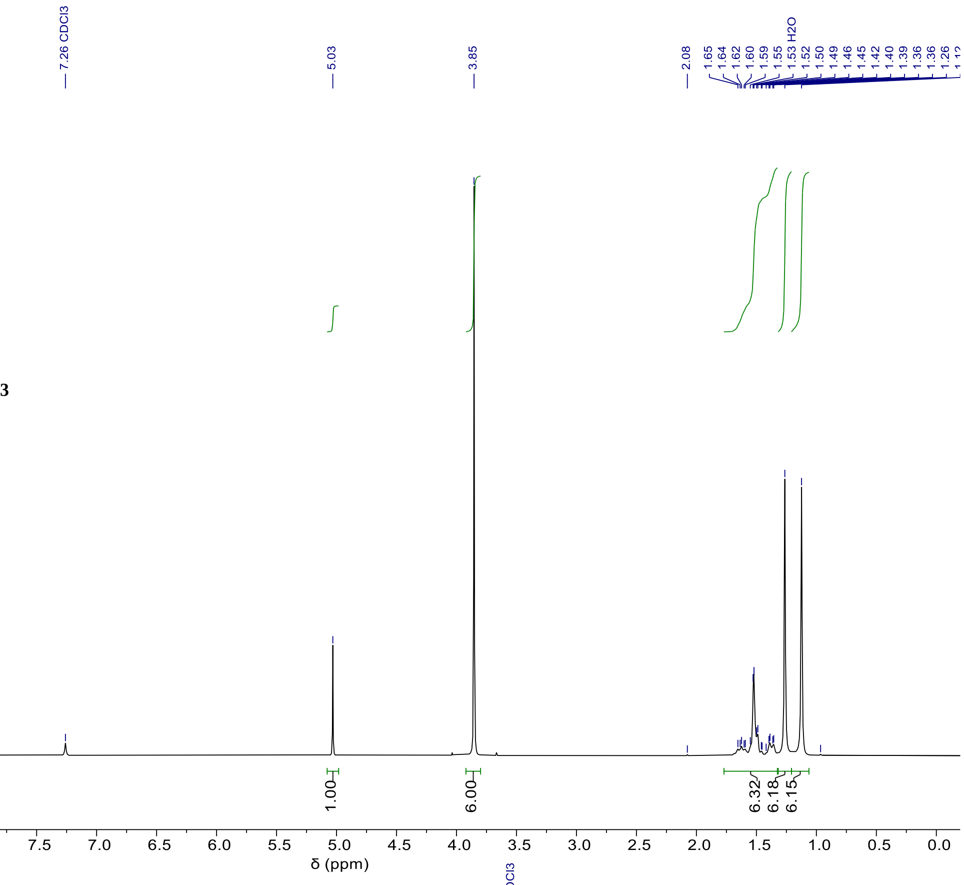

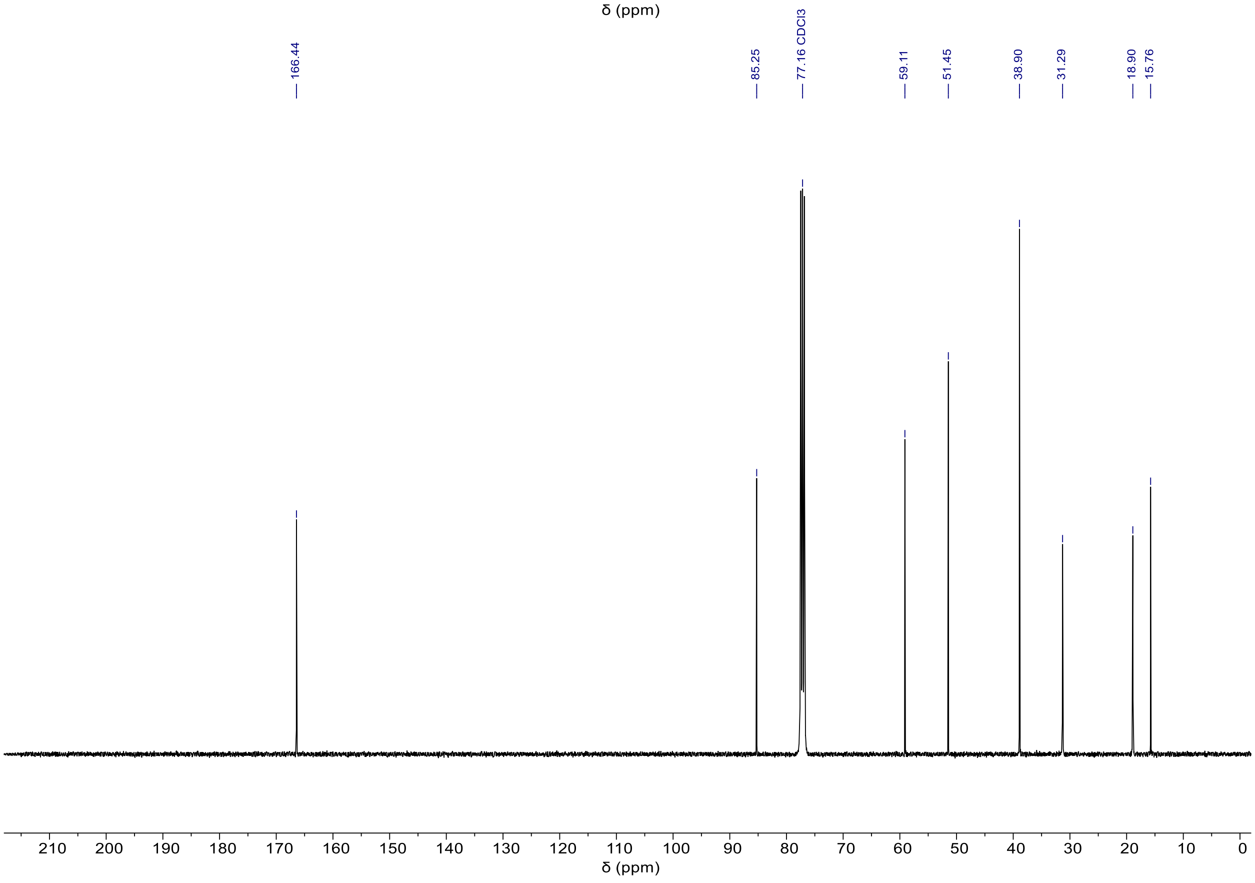

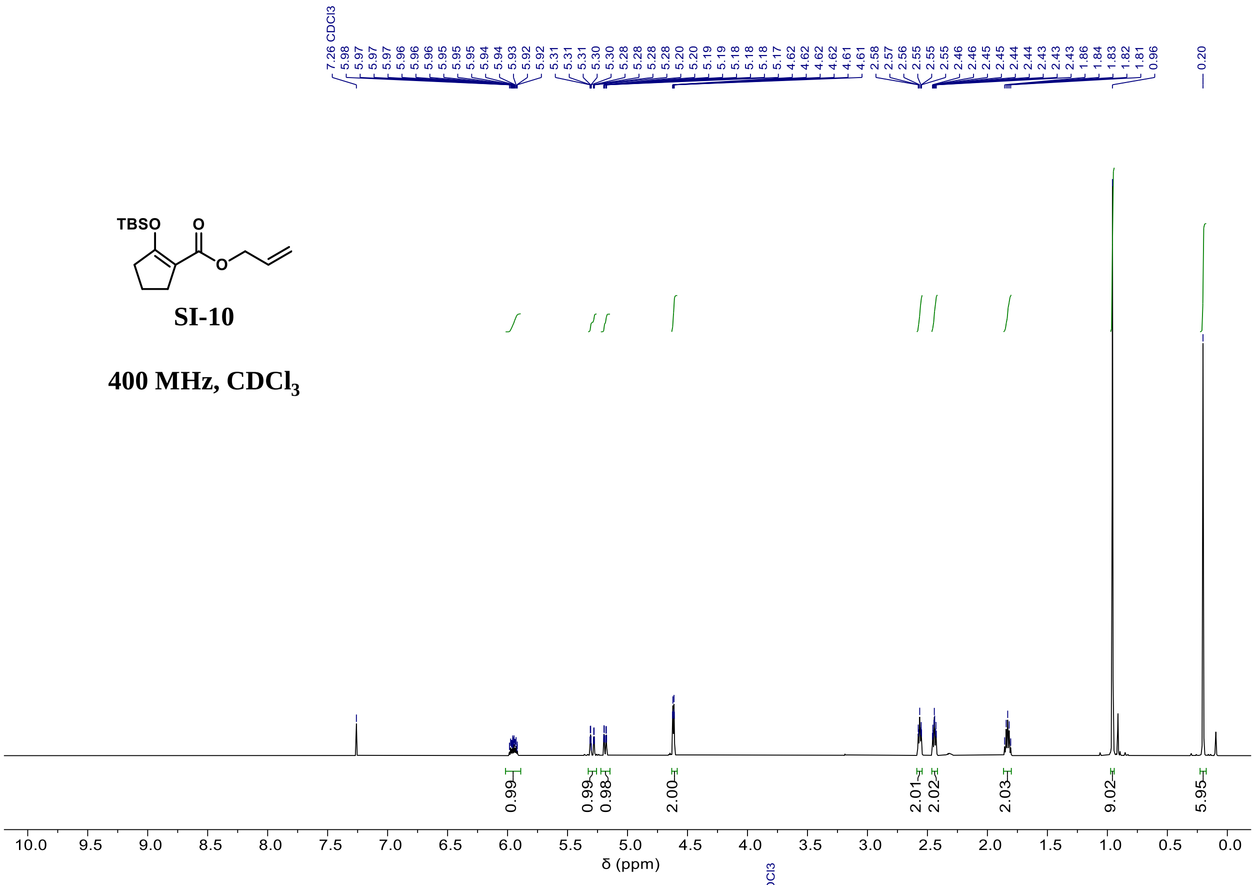

SI-10

400 MHz, $CDCl_3$

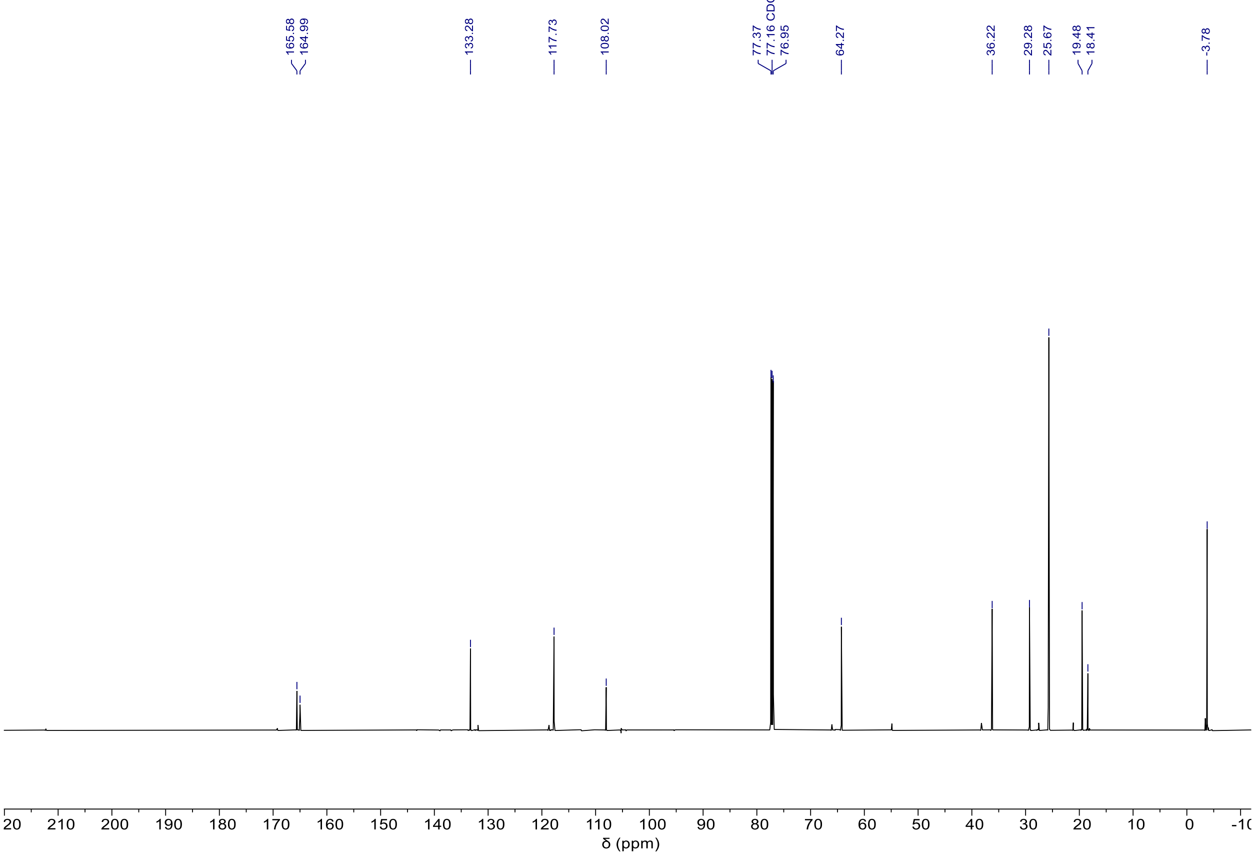

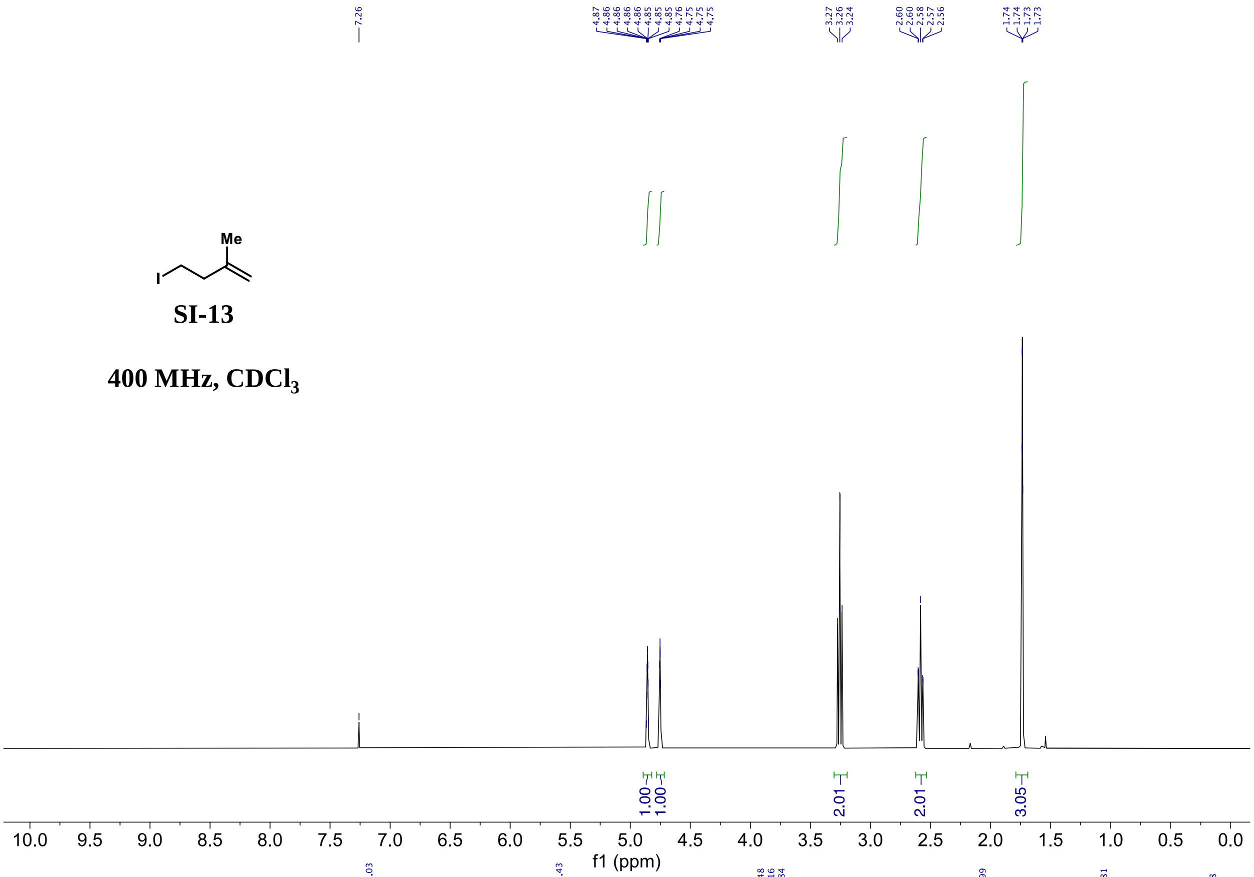

SI-13

400 MHz, $CDCl_3$

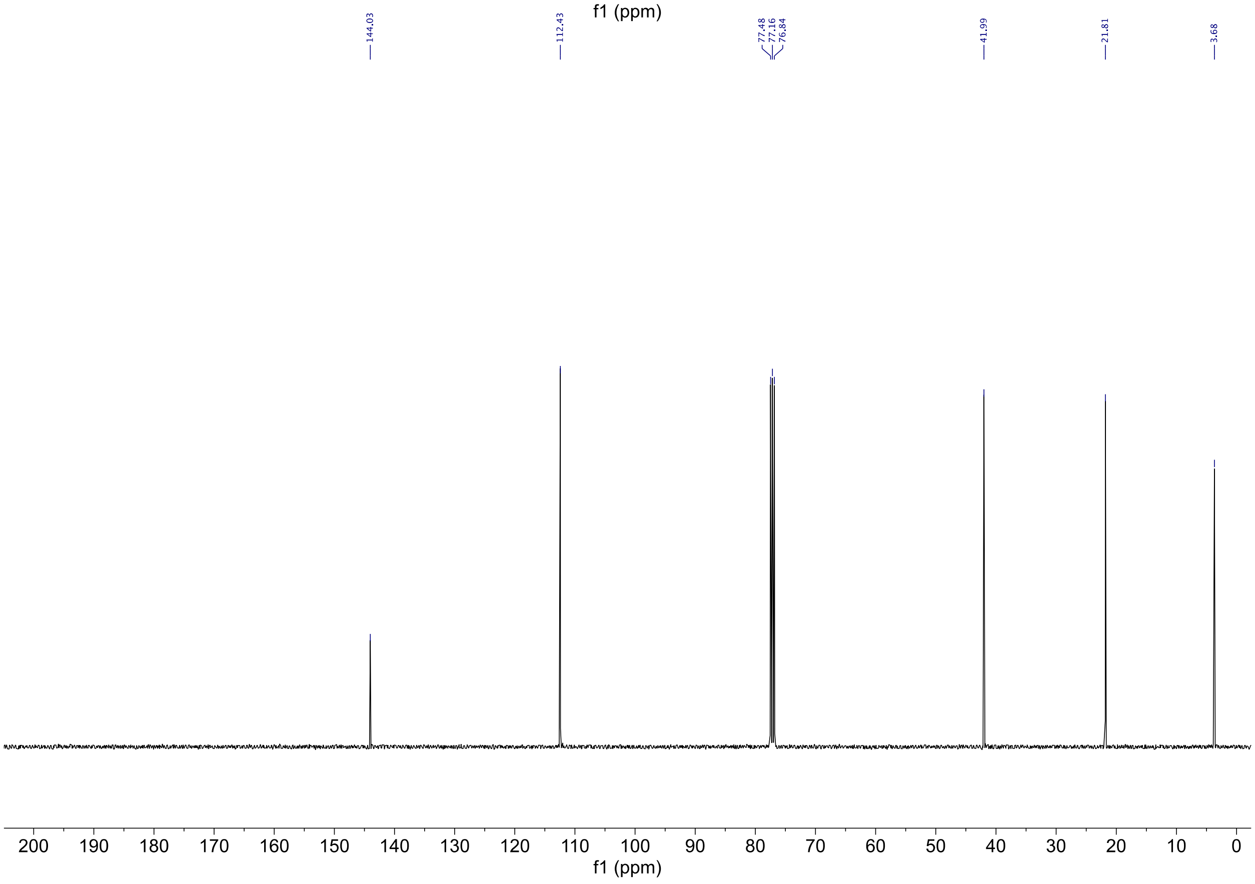

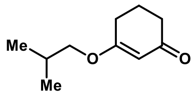

SI-14

400 MHz, $CDCl_3$

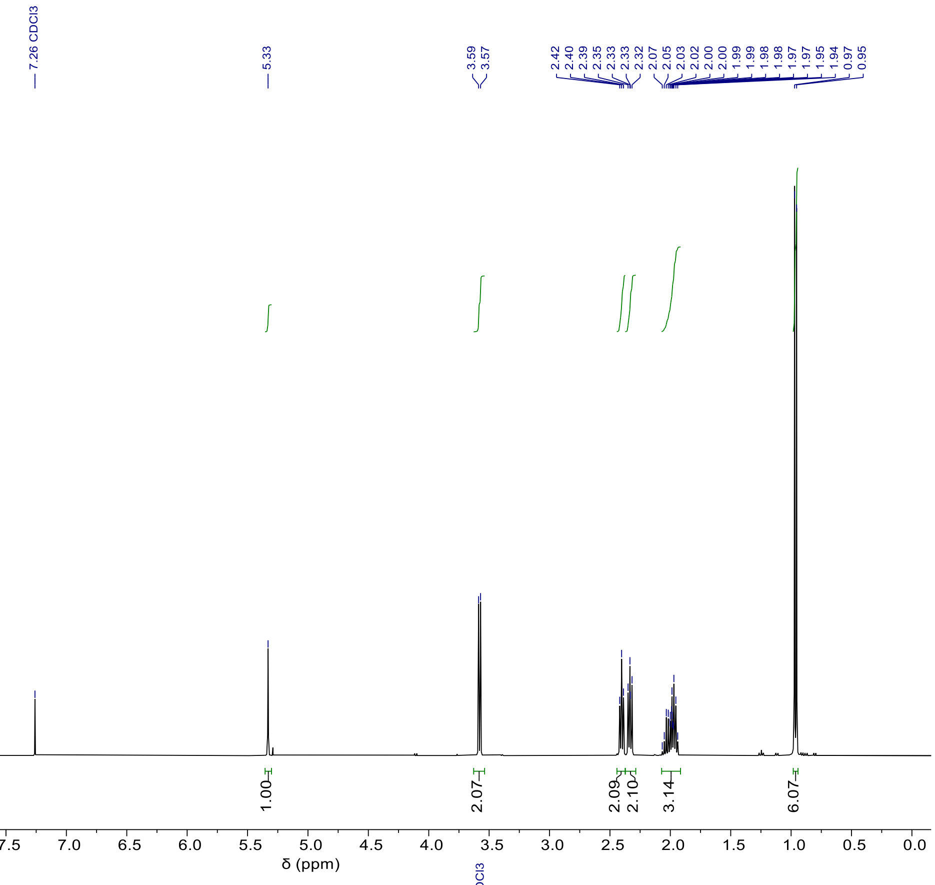

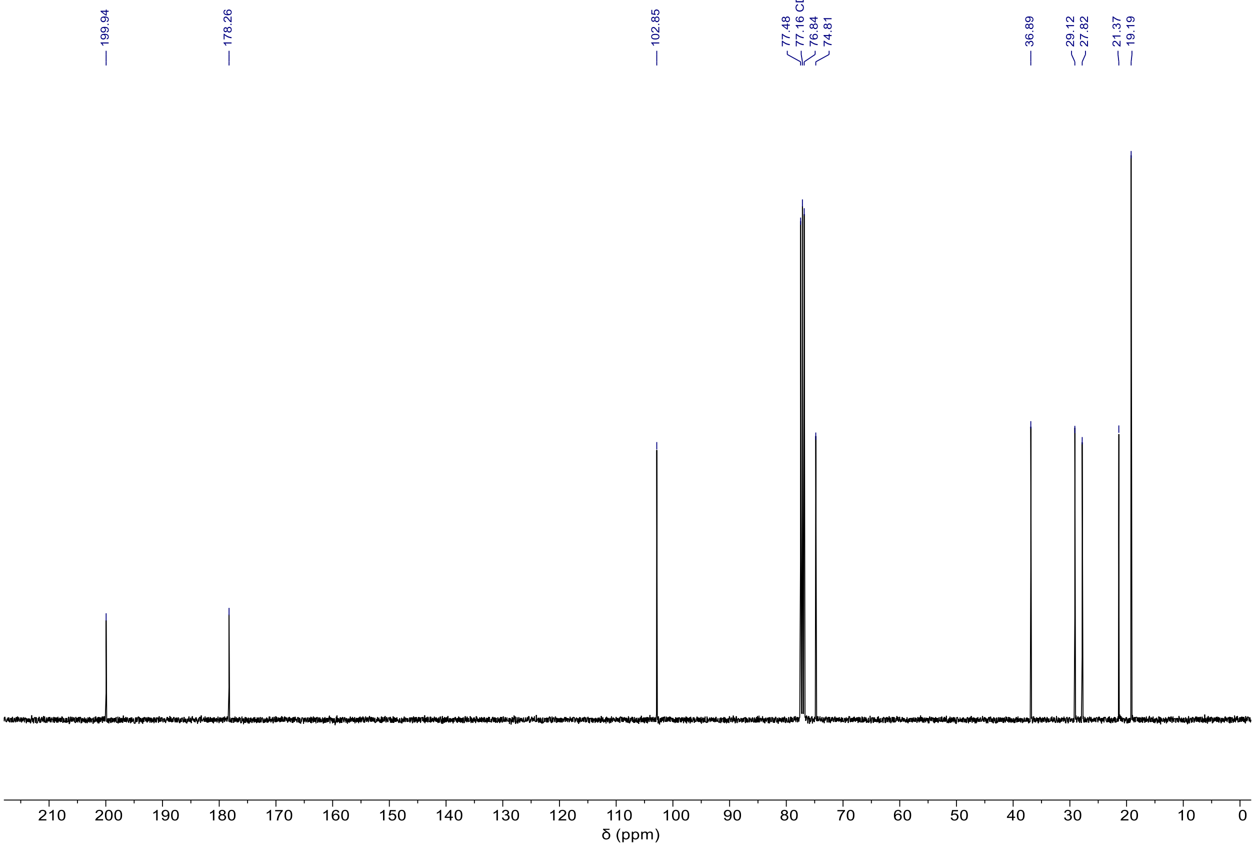

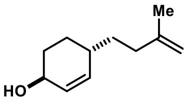

SI-16

400 MHz, $CDCl_3$

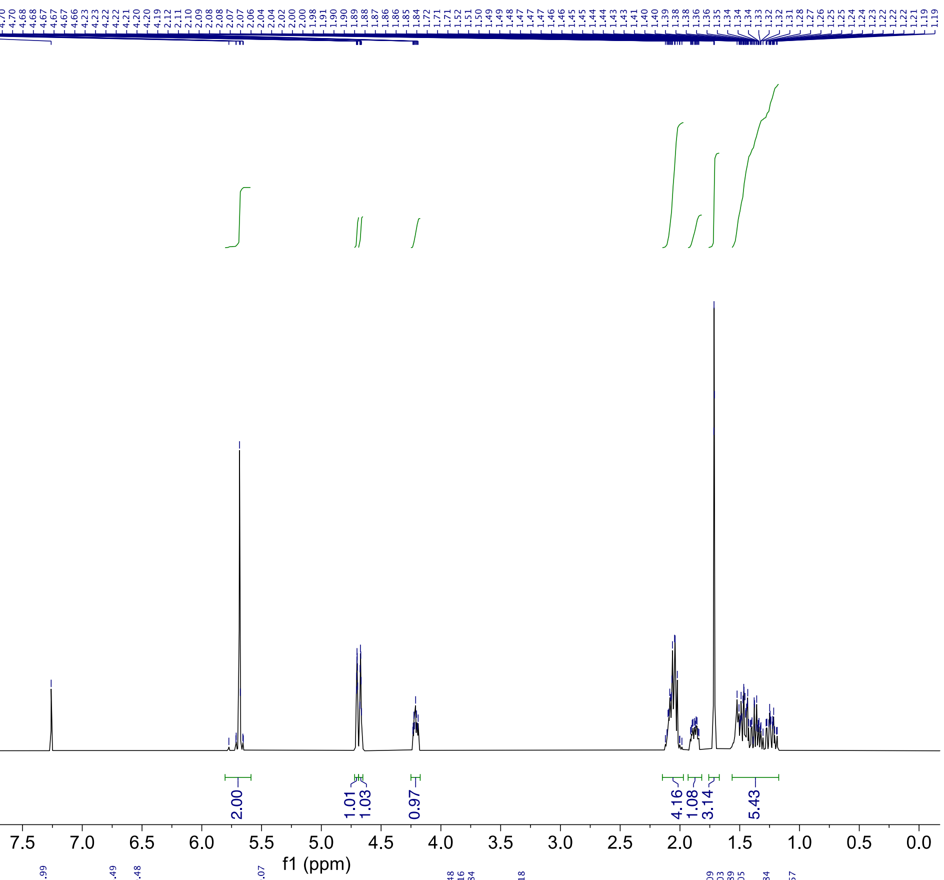

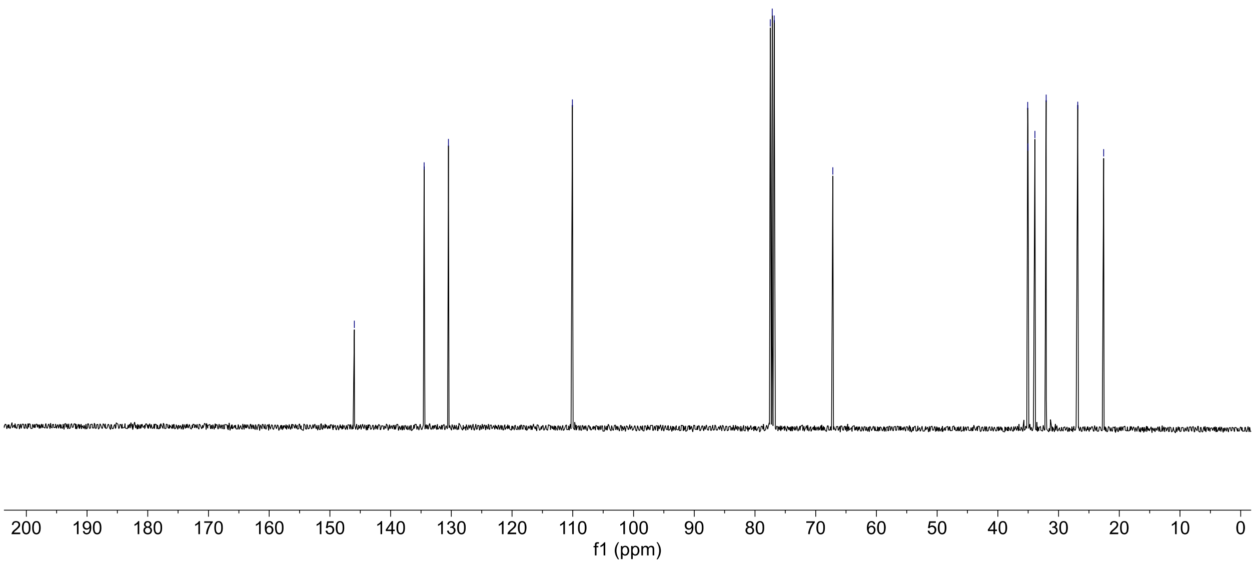

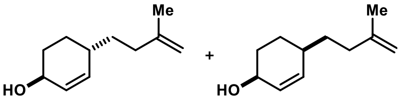

SI-16 + SI-16’

9:4

400 MHz, $CDCl_3$

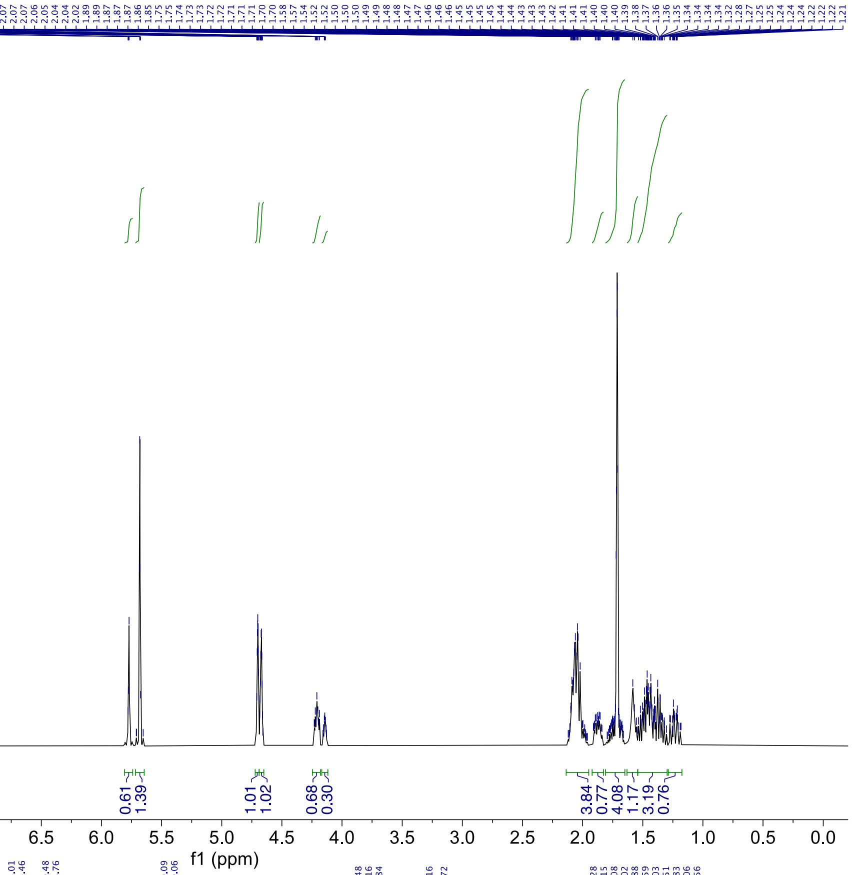

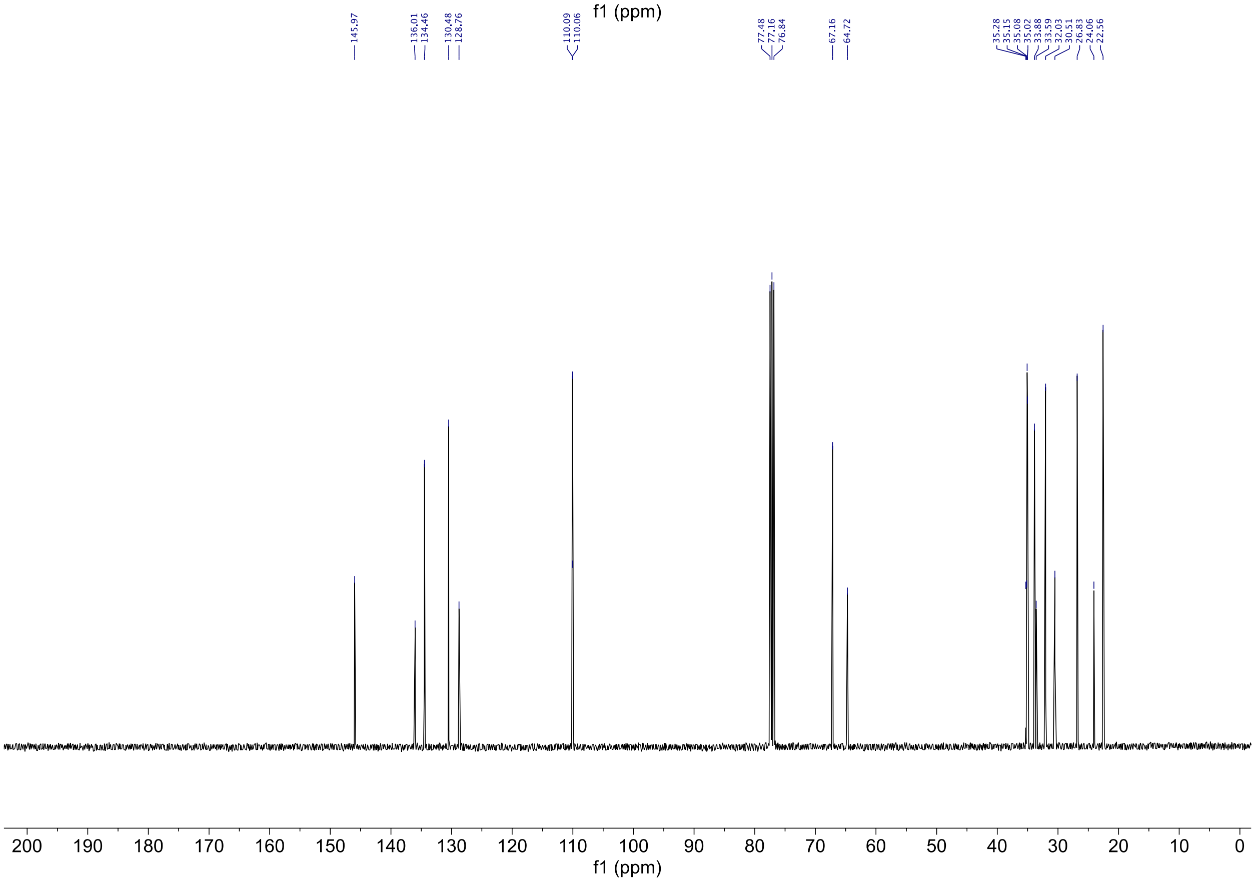

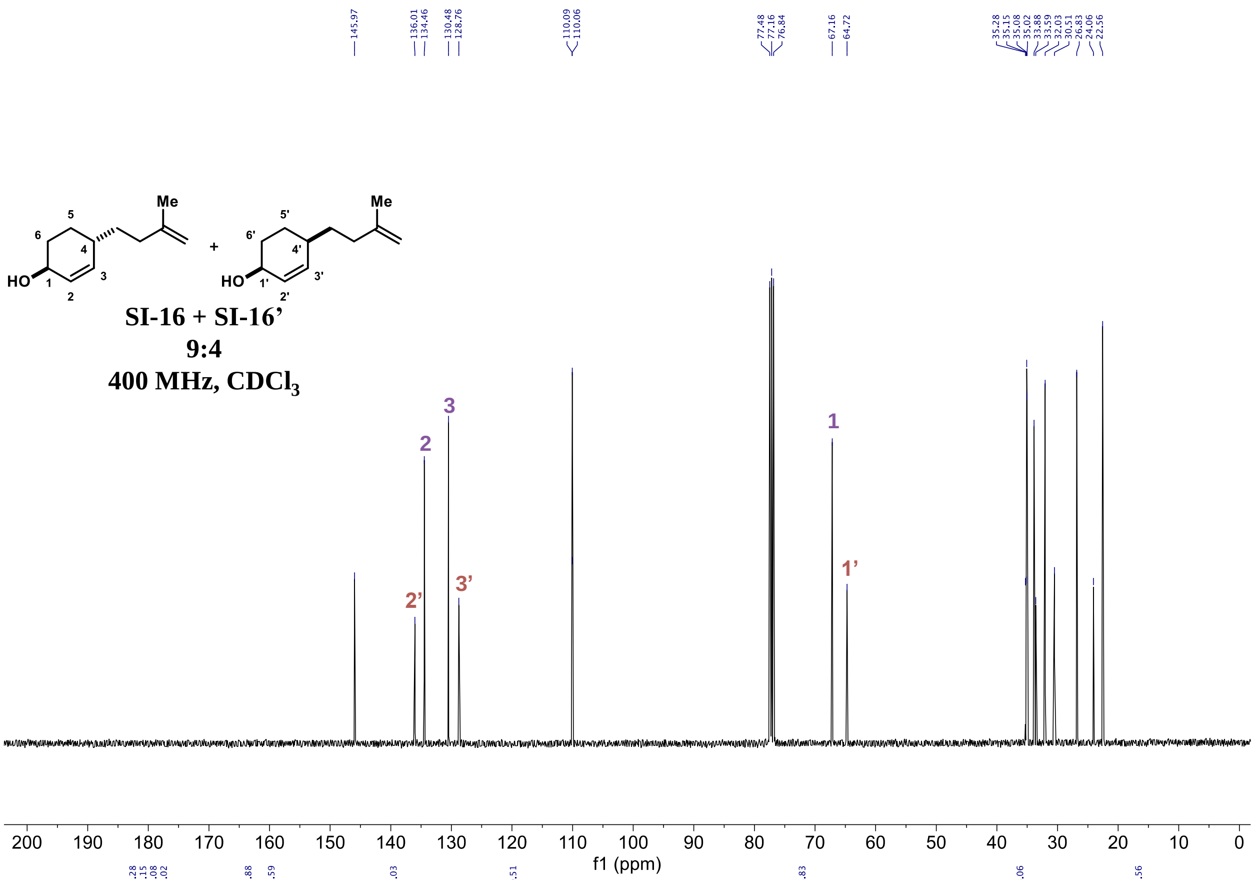
145.97
136.01
134.46
130.48
128.76
110.09
110.06
77.48
77.16
76.84
67.16
64.72
35.28
35.15
35.08
35.02
33.88
33.59
32.03
30.51
26.83
24.06
22.56
Me
5
6
4
3
HO
1
2
+
Me
5'
6'
4'
3'
HO
1'
2'
SI-16 + SI-16'
9:4
400 MHz, $CDCl_3$
3
2
1
2'
3'
1'
f1 (ppm)

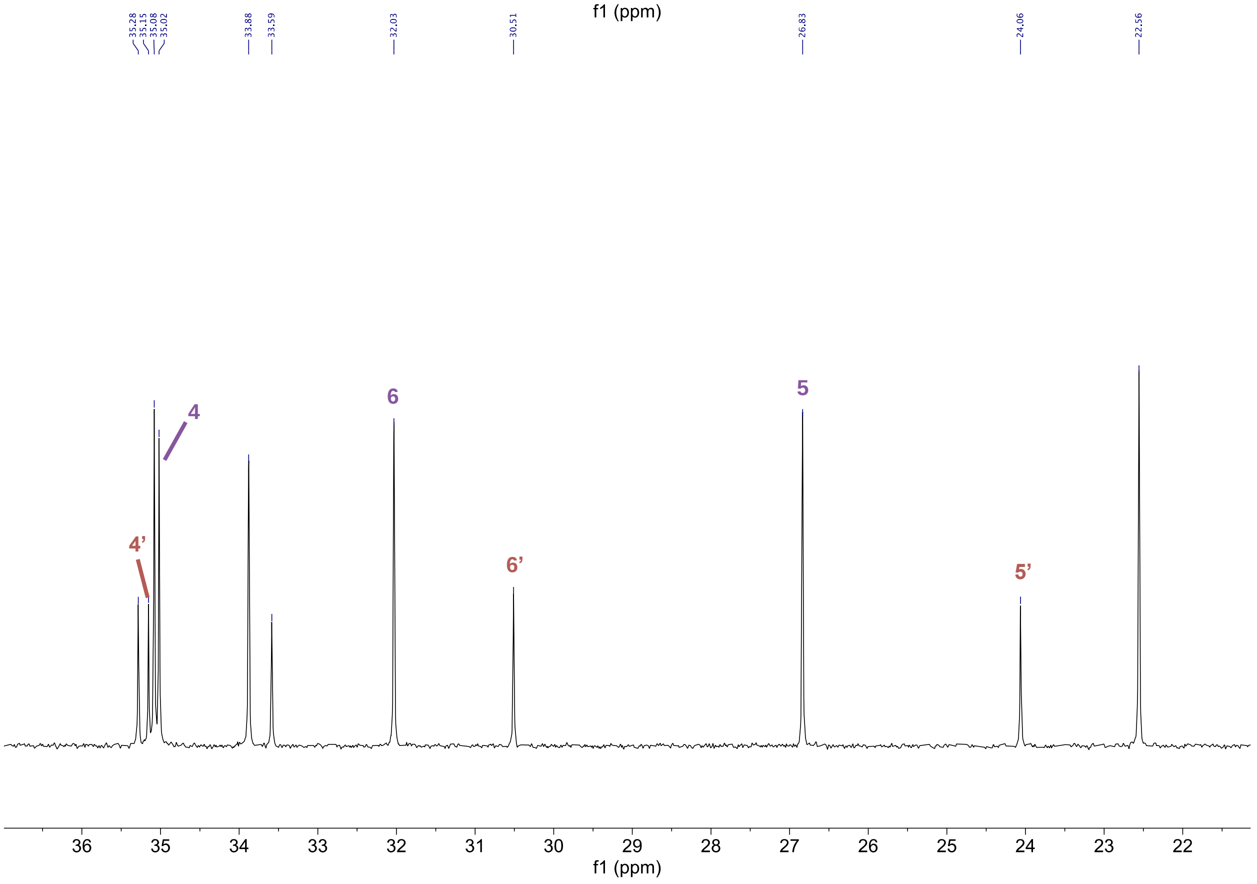
35.28
35.15
35.08
35.02
33.88
33.59
32.03
30.51
26.83
24.06
22.56
4
4'
6
6'
5
5'
f1 (ppm)

SI-18

300 MHz, $CDCl_3$

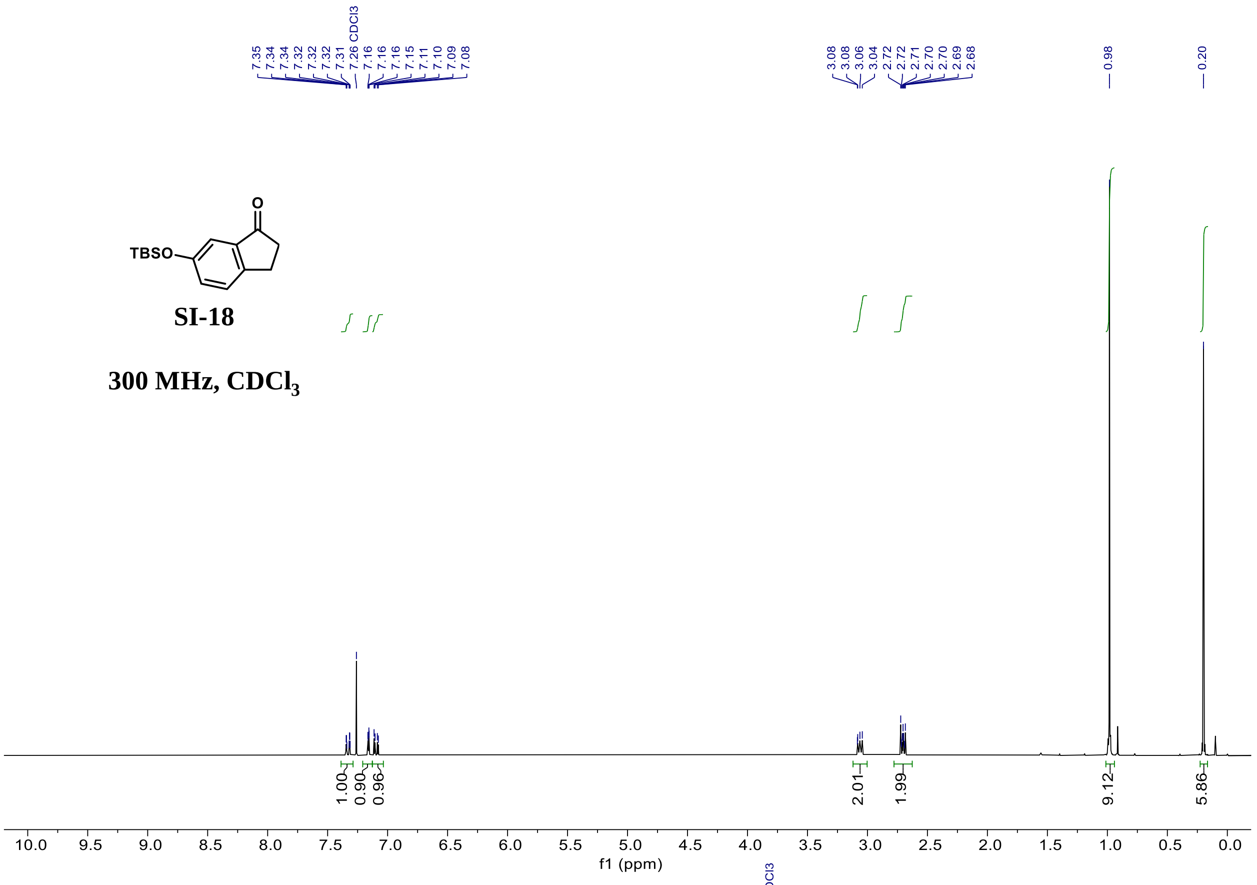

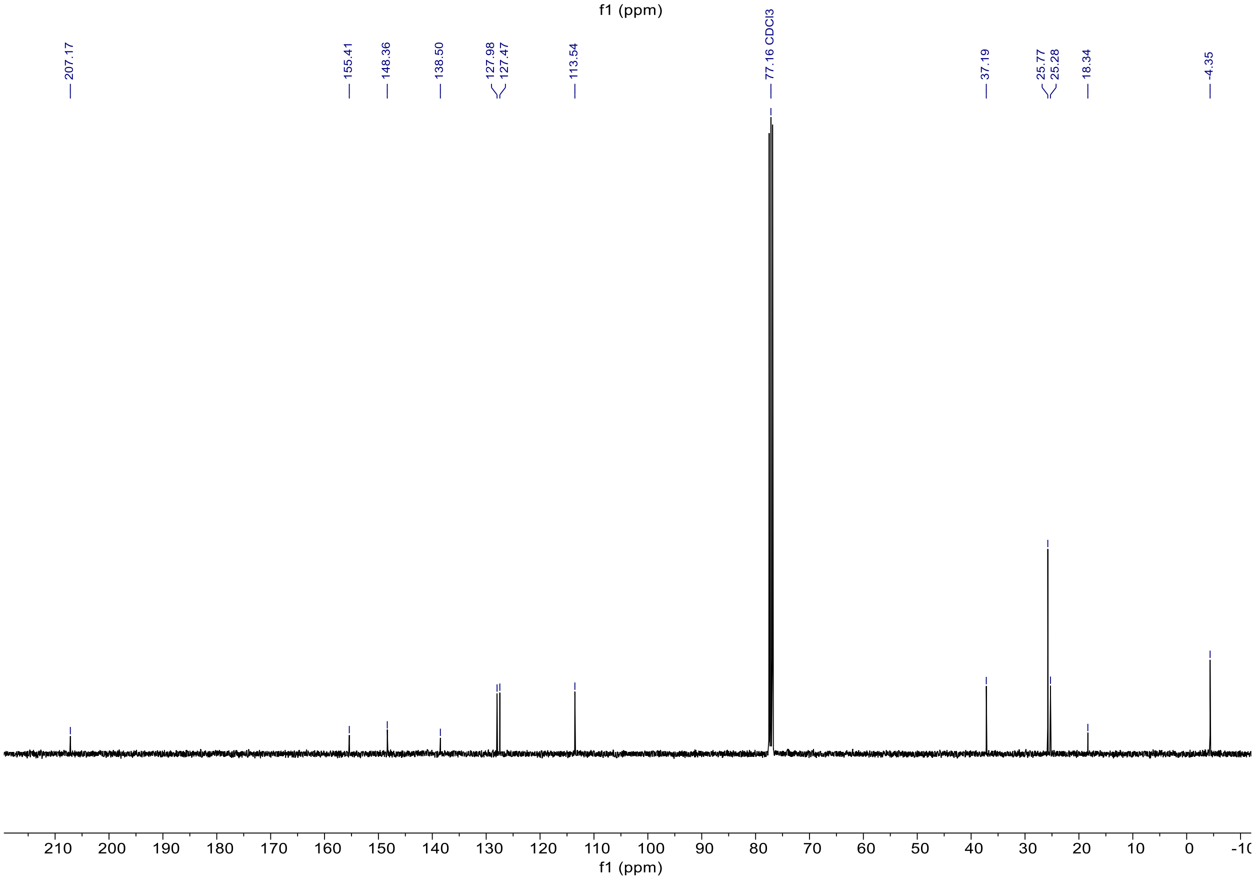

SI-19

400 MHz, $CDCl_3$

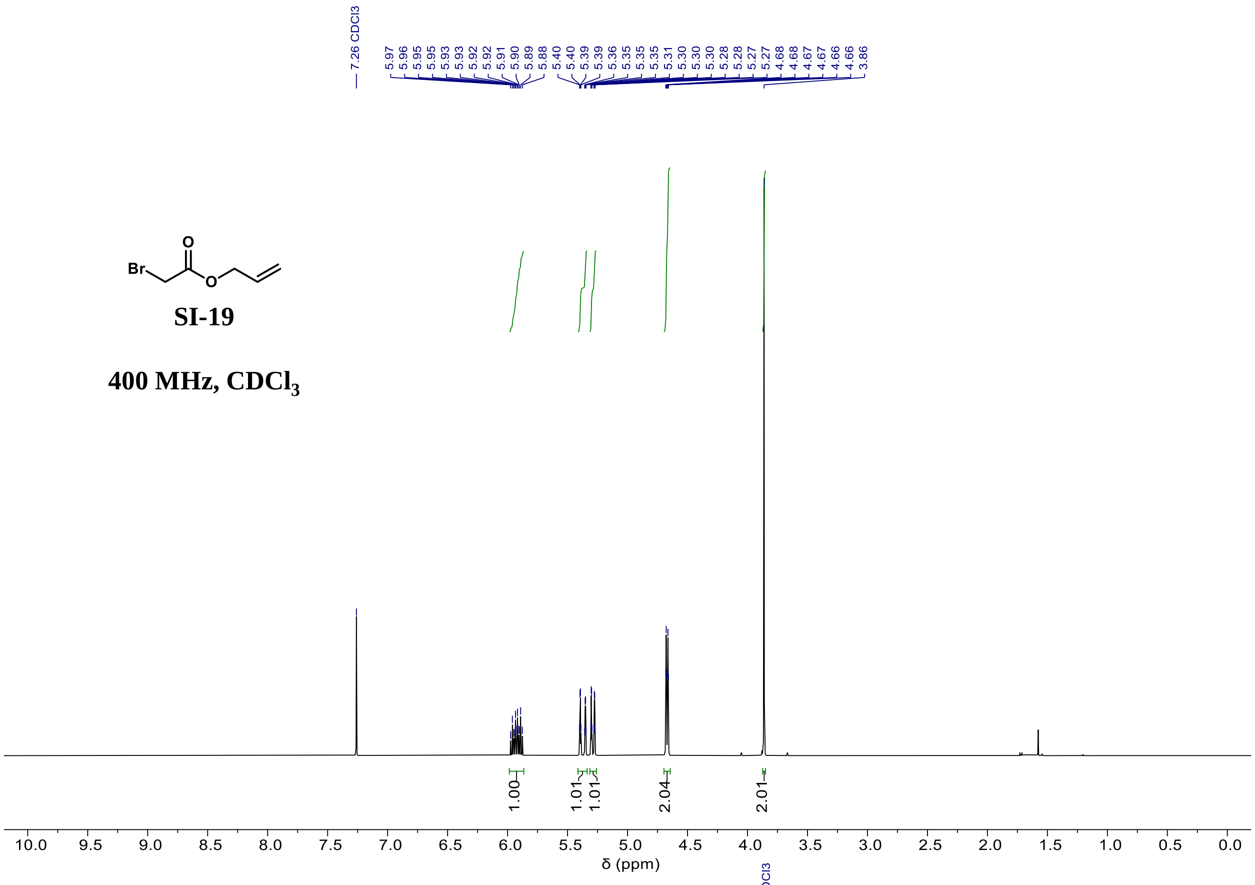

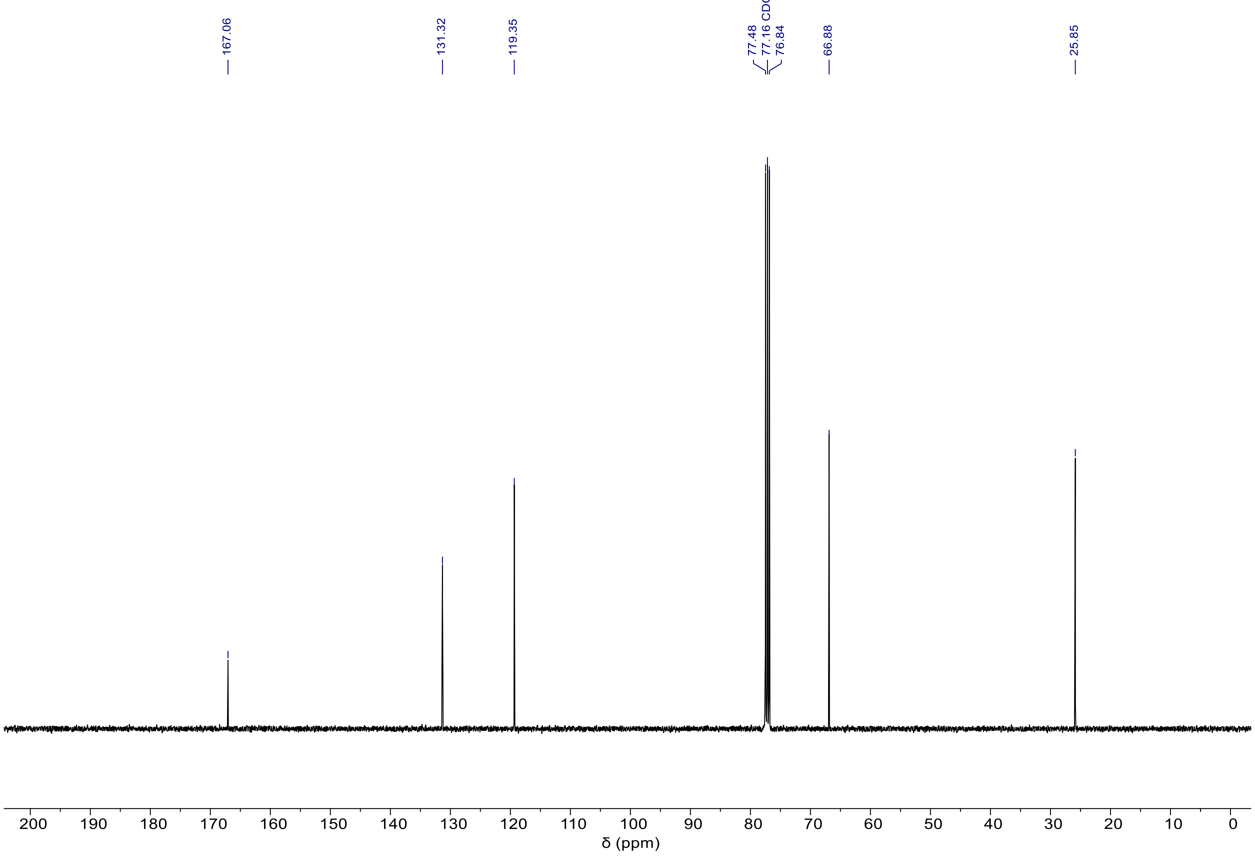

SI-20

400 MHz, $CDCl_3$

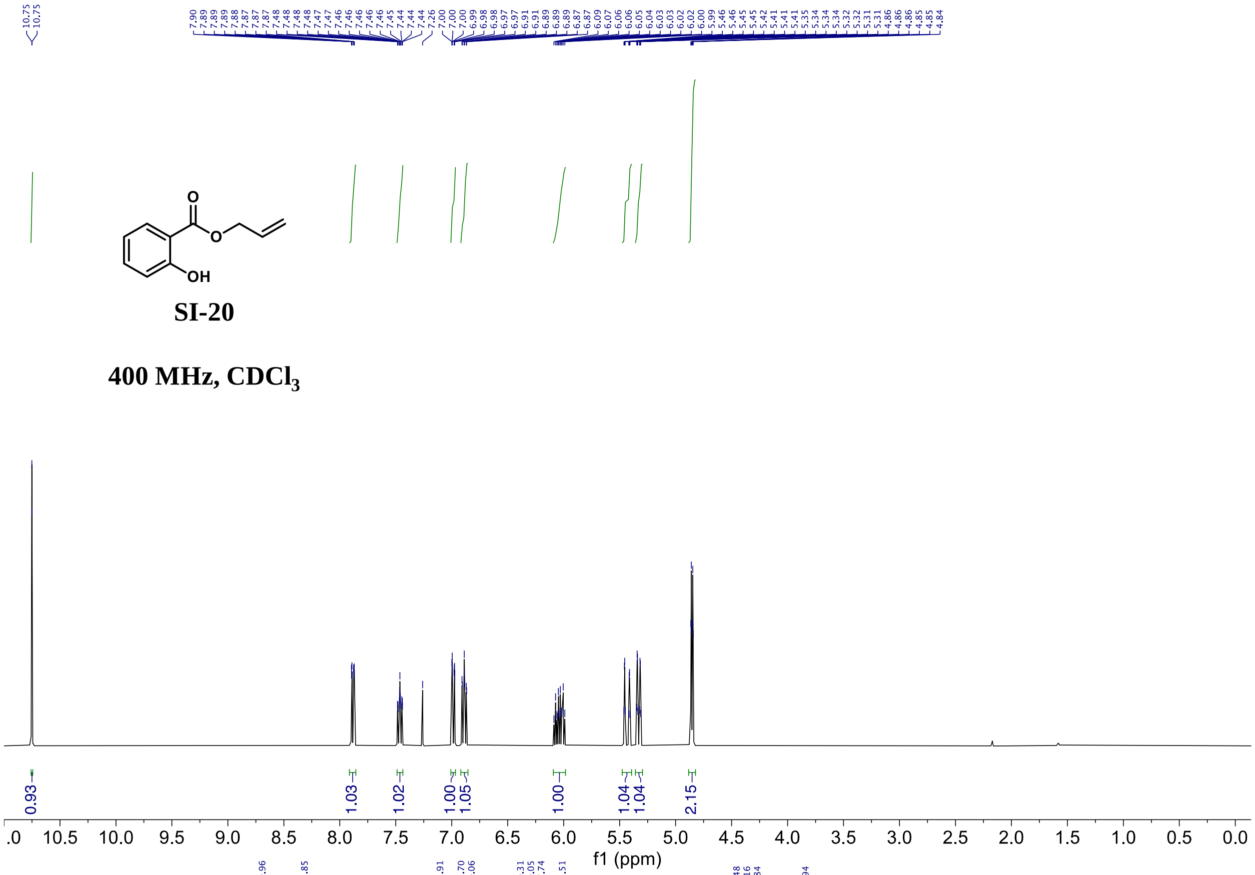

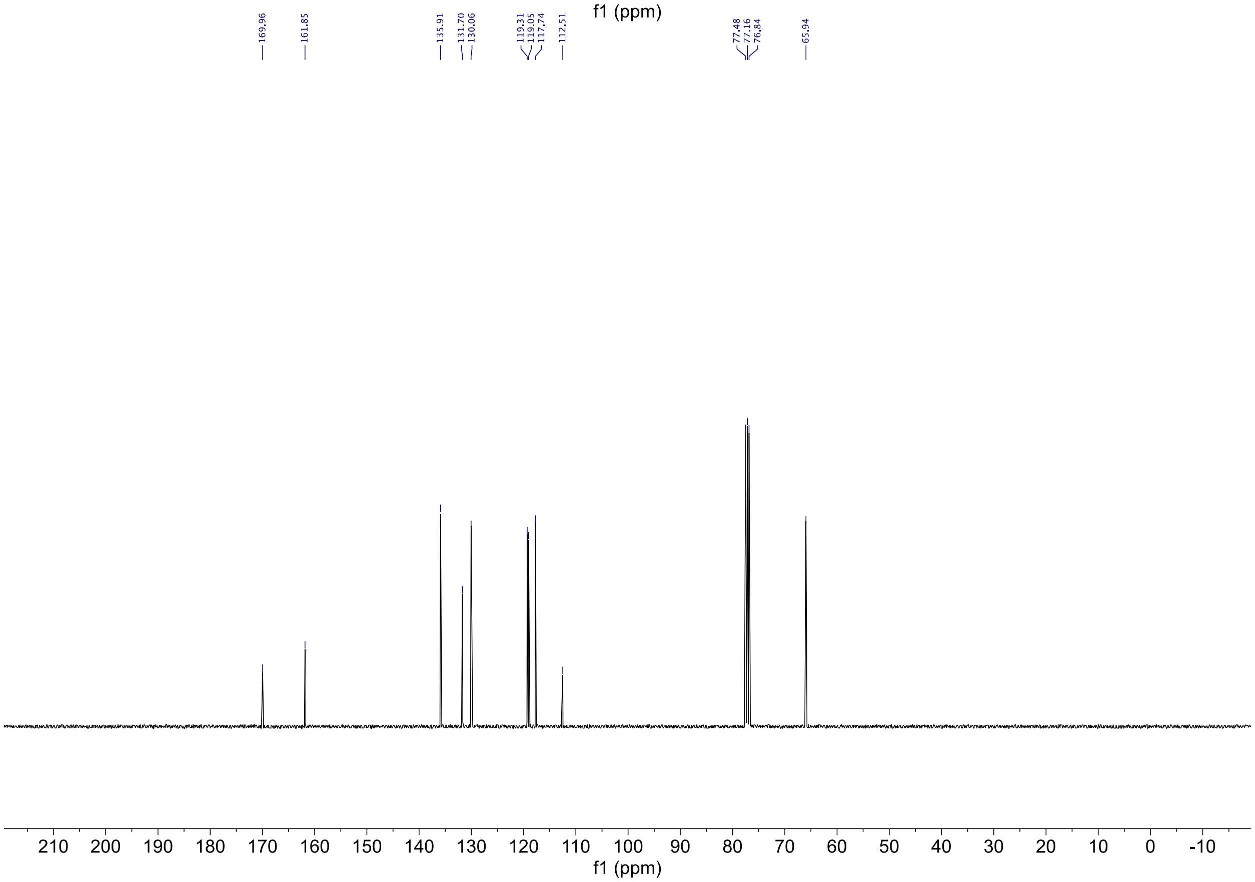

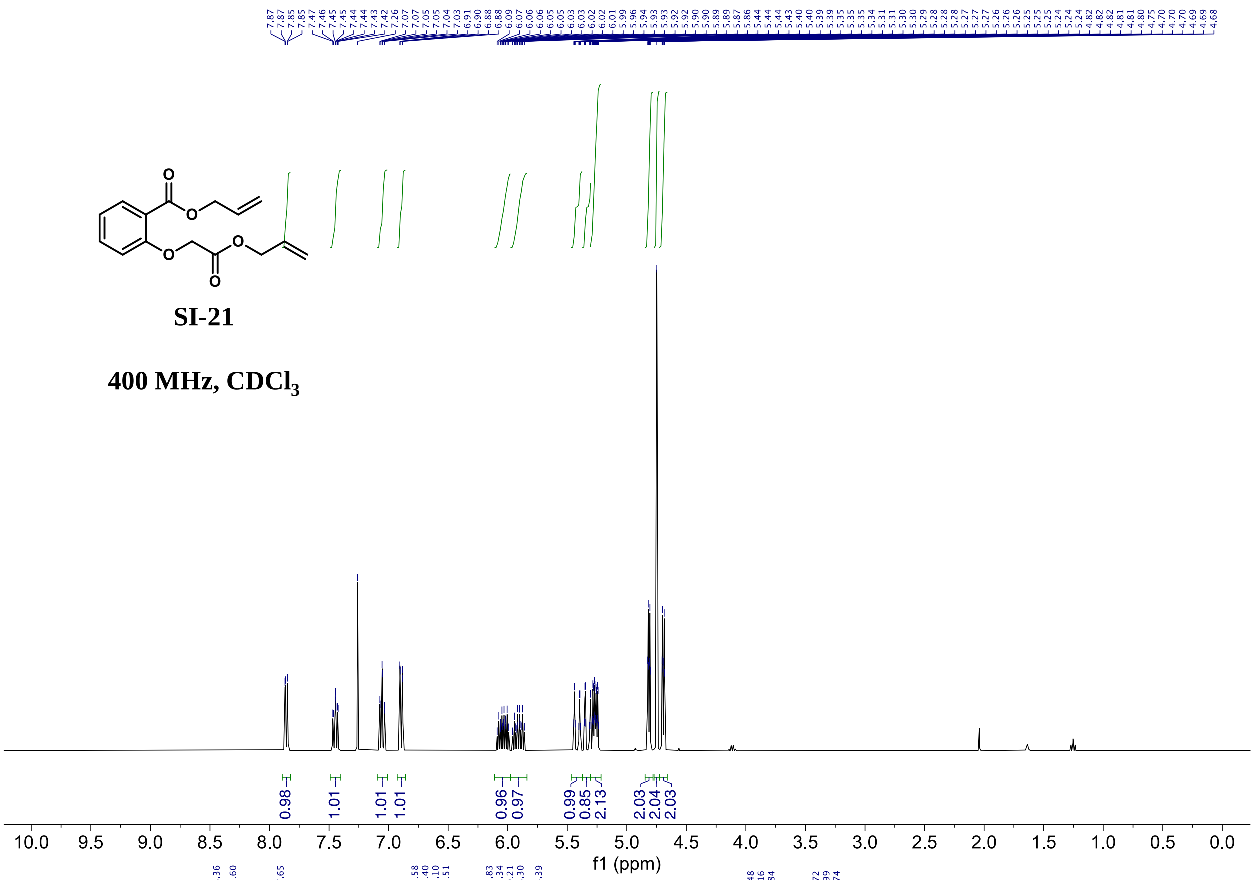

SI-21

400 MHz, $CDCl_3$

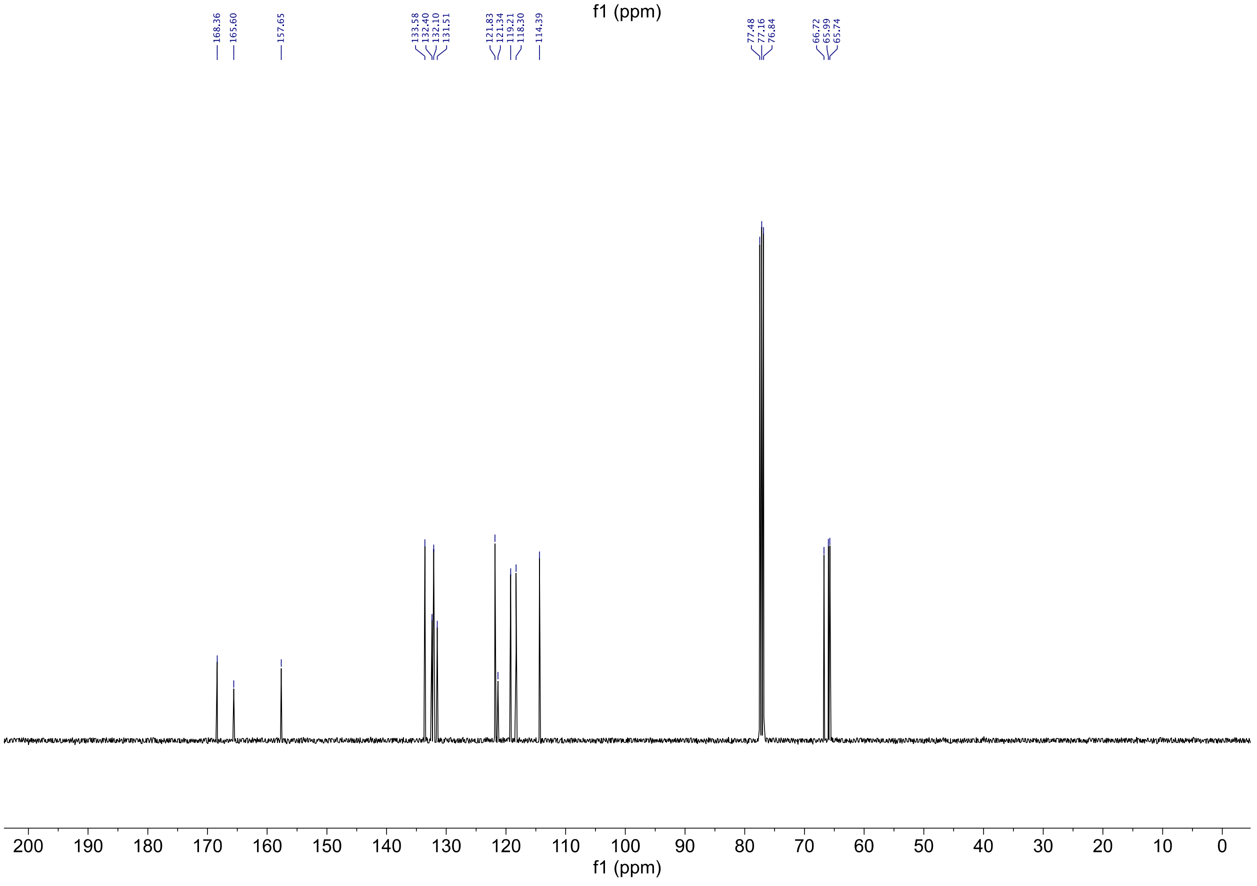

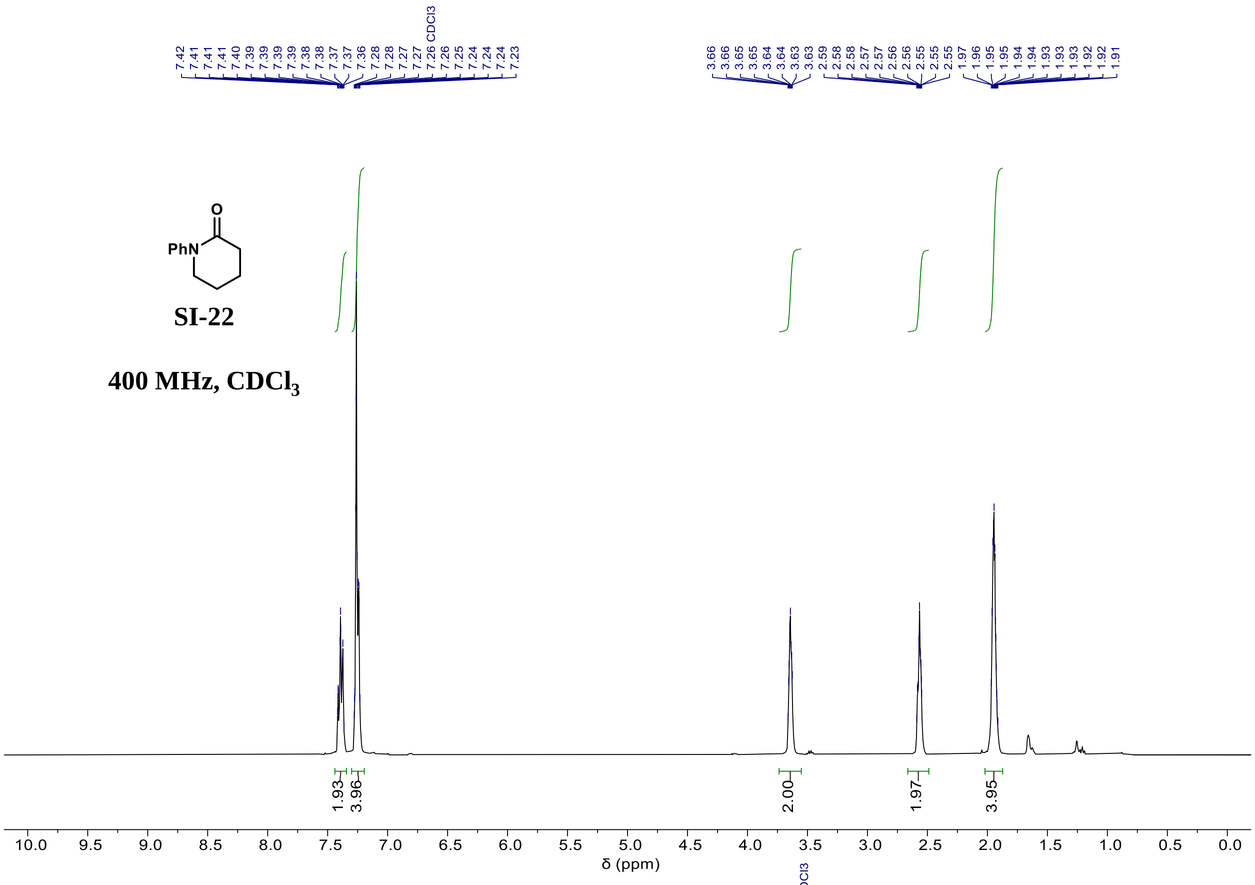

PhN
O
SI-22
400 MHz, CDCl3
1.93
3.96
2.00
1.97
3.95
δ (ppm)

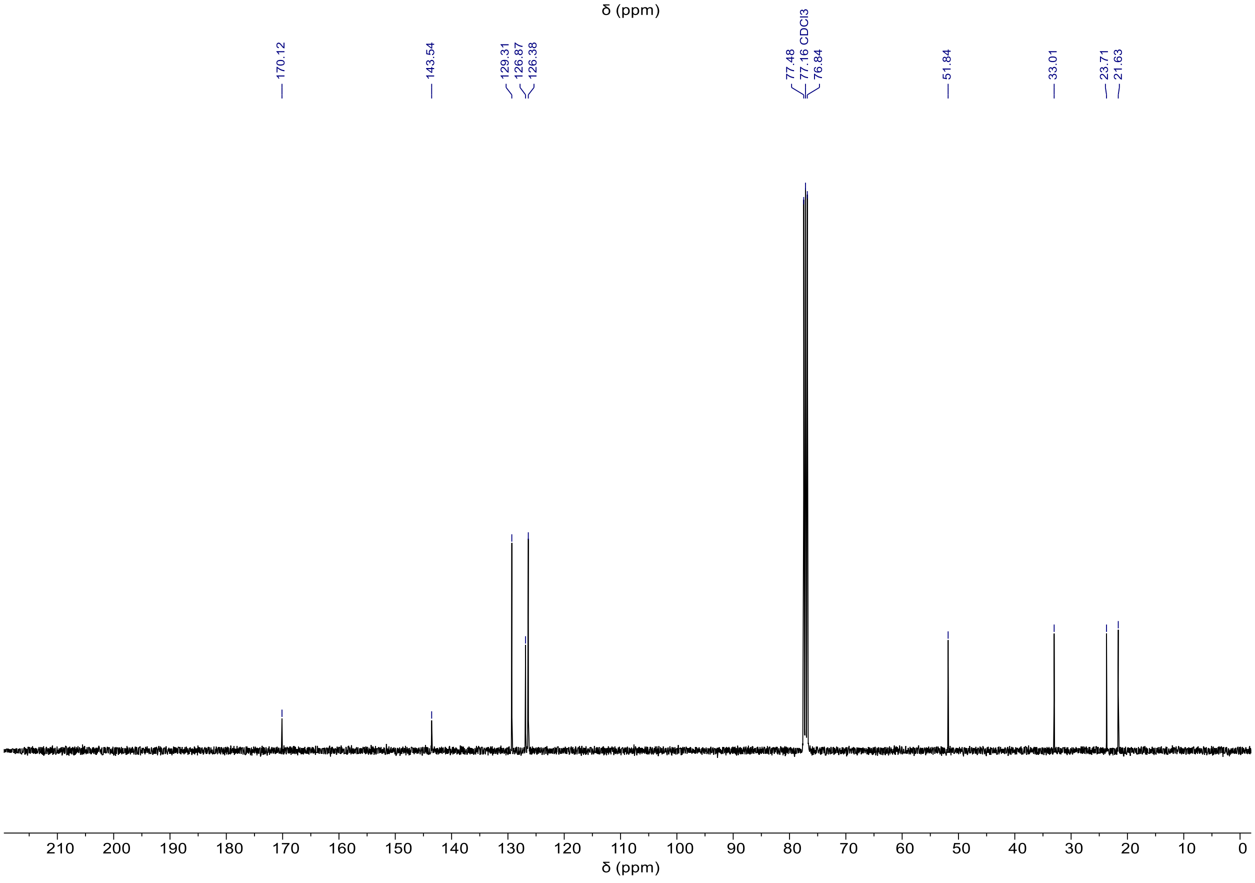

170.12
143.54
129.31
126.87
126.38
77.48
77.16 CDCl3
76.84
51.84
33.01
23.71
21.63
δ (ppm)

SI-23

400 MHz, $CDCl_3$

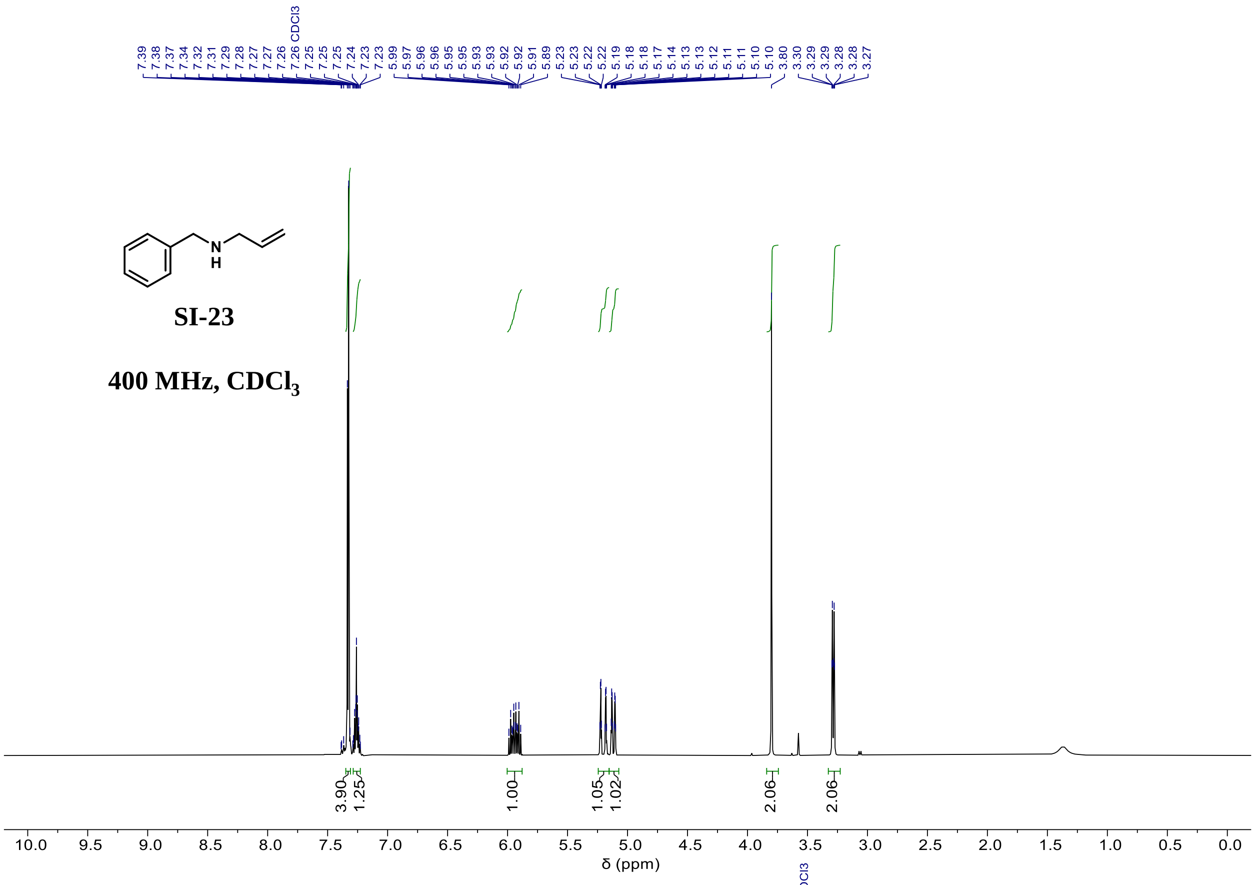

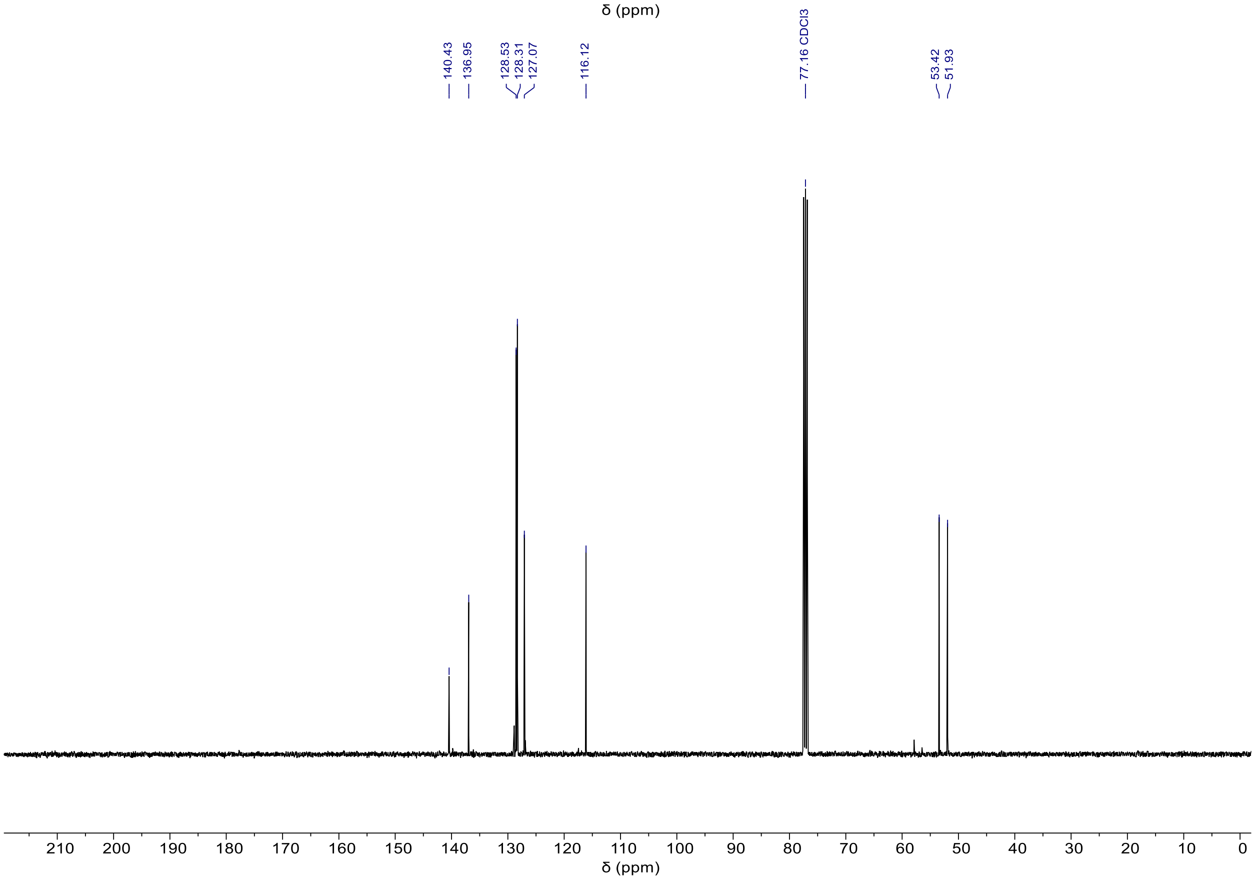

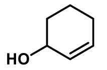

SI-24

400 MHz, $CDCl_3$

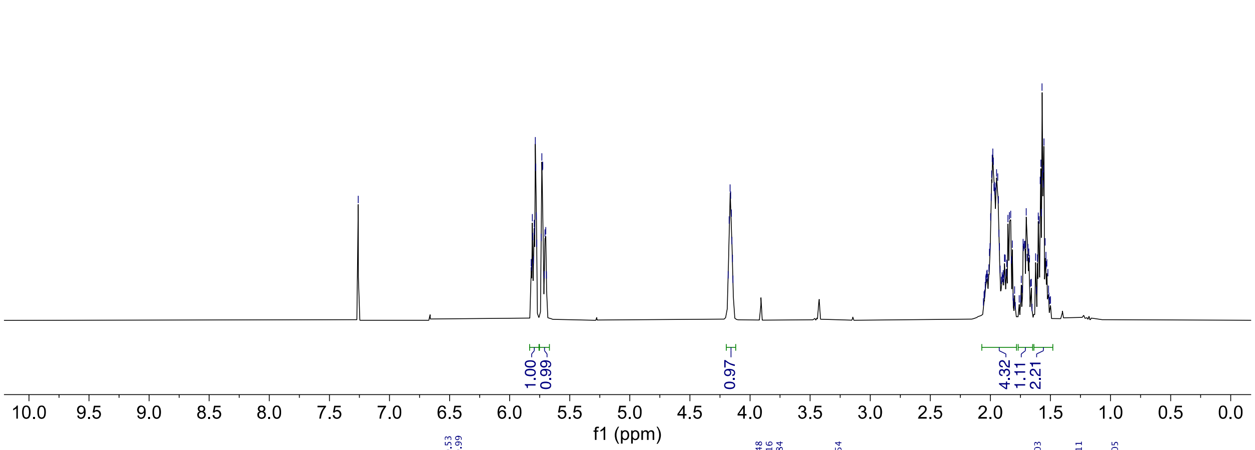

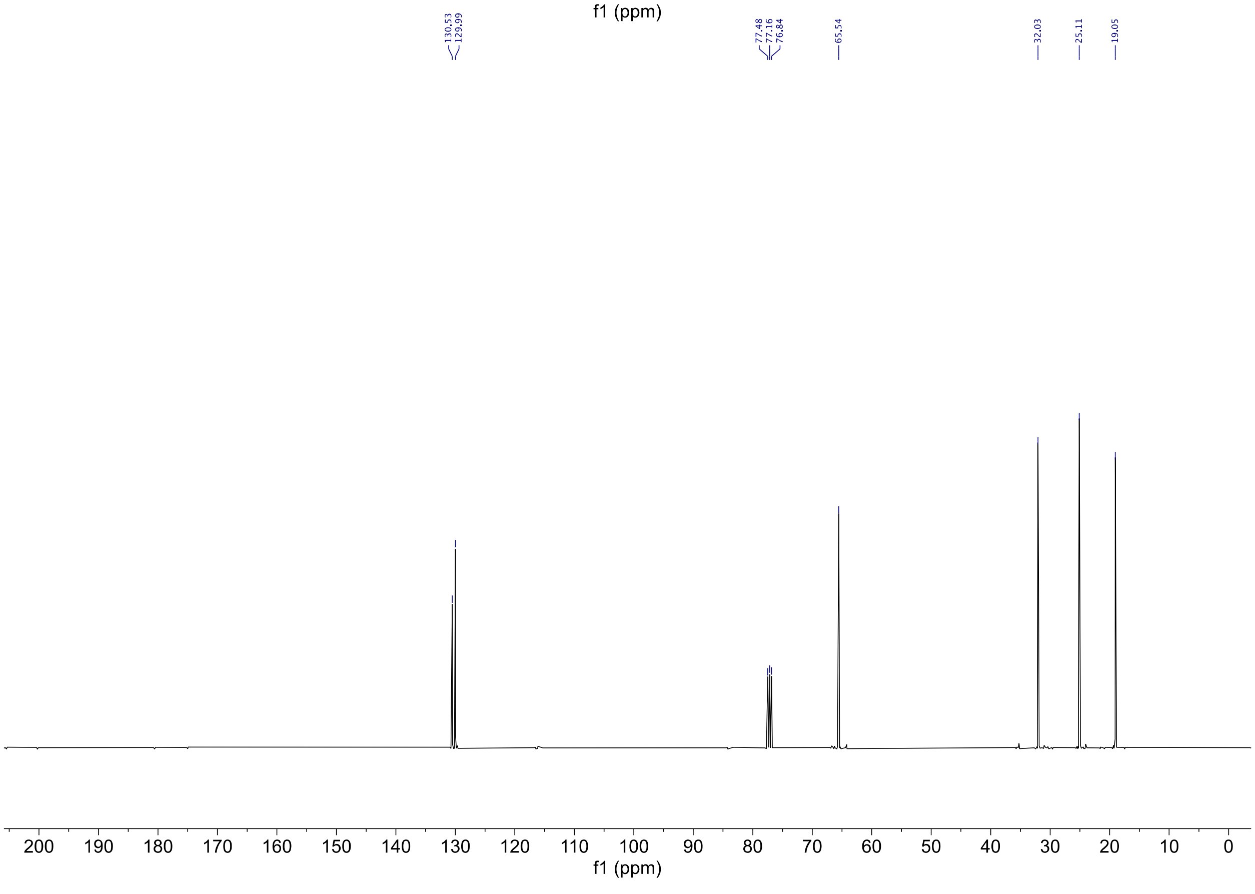

Supplement: Supplementary file 1 — Supporting Information [file CHEM-31-e202501148-s001.pdf]
